# Mechanistic Insights into Regioselective Arene Insertion Using Bis(phosphine) Cobalt(I) Hydrides to Form 1,3-Cyclohexadienes

**DOI:** 10.1021/acs.organomet.6c00013

**Published:** 2026-04-16

**Authors:** Maya J. Lebowitz, Lauren N. Mendelsohn, Hongyu Zhong, Matthew V. Pecoraro, Michael Shevlin, Paul J. Chirik

**Affiliations:** † Department of Chemistry, 6740 Princeton University, Princeton, New Jersey 08544, United States; ‡ Merck & Co., Inc., Rahway, New Jersey 07065, United States

## Abstract

Bis­(phosphine) cobalt allyl complexes were used as isolable precursors to access transient, 14-electron cobalt­(I) hydrides that promoted the insertion of a series of substituted benzenes. The selectivity of the insertion reaction was assayed by analysis of the resulting diamagnetic 18-electron bis­(phosphine)­Co­(η^5^-cyclohexadienyl) complexes by multinuclear NMR spectroscopy. Monitoring the insertion of α,α,α-trifluorotoluene (PhCF_3_) with [(*R,R*)-(^iPr^DuPhos)­CoH] by NMR spectroscopy revealed an initially aselective kinetic product mixture that underwent isomerization to the thermodynamically preferred *ipso*-CF_3_ product. The site selectivity for *ipso*-CF_3_ insertion over alkyl, boryl, and silyl substituents was observed across a series of disubstituted benzenes, motivating an investigation into the kinetic and thermodynamic selectivities of representative alkyl trifluoromethylbenzenes using NMR spectroscopy and X-ray crystallography. Protonation of various cobalt­(I) cyclohexadienyl complexes afforded C­(sp^3^)-CF_3_-substituted cyclohexadienes in high yields, which were trapped by Diels–Alder reactions.

## Graphical abstract

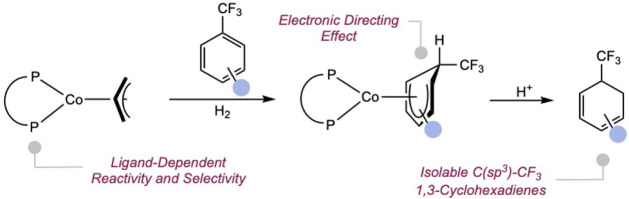

## Introduction

The selective synthesis of cyclohexadienes by partial reduction of benzenes is a powerful approach for constructing value-added building blocks, but is challenged by the aromatic stabilization of arenes.^1^ Significant advances have been made in dearomatization and functionalization of arenes with early transition metals,^2,3^ but site-selective dearomatization remains a challenge, specifically with electron-deficient arenes, where control over the initial η^6^-arene coordination, formation of isomeric mixtures, low-yielding reactions, and limited substrate compatibility persist (Scheme 1A).^4–8^ Harman and coworkers have demonstrated that η^2^-arene coordination of α,α,α-trifluorotoluene (PhCF_3_) to low-valent molybdenum^9^ and tungsten^10^ compounds enables *ortho*-selective protonation and the synthesis of enantioenriched 1,3-disubstituted trifluoromethyl cyclohexadienes. This approach was also successfully applied to phenyl sulfones.^11^ These foundational studies highlight the potential of alternative metal-arene coordination modes to enable the general chemoselective synthesis of electron-deficient cyclohexadienes.

**Scheme 1:**
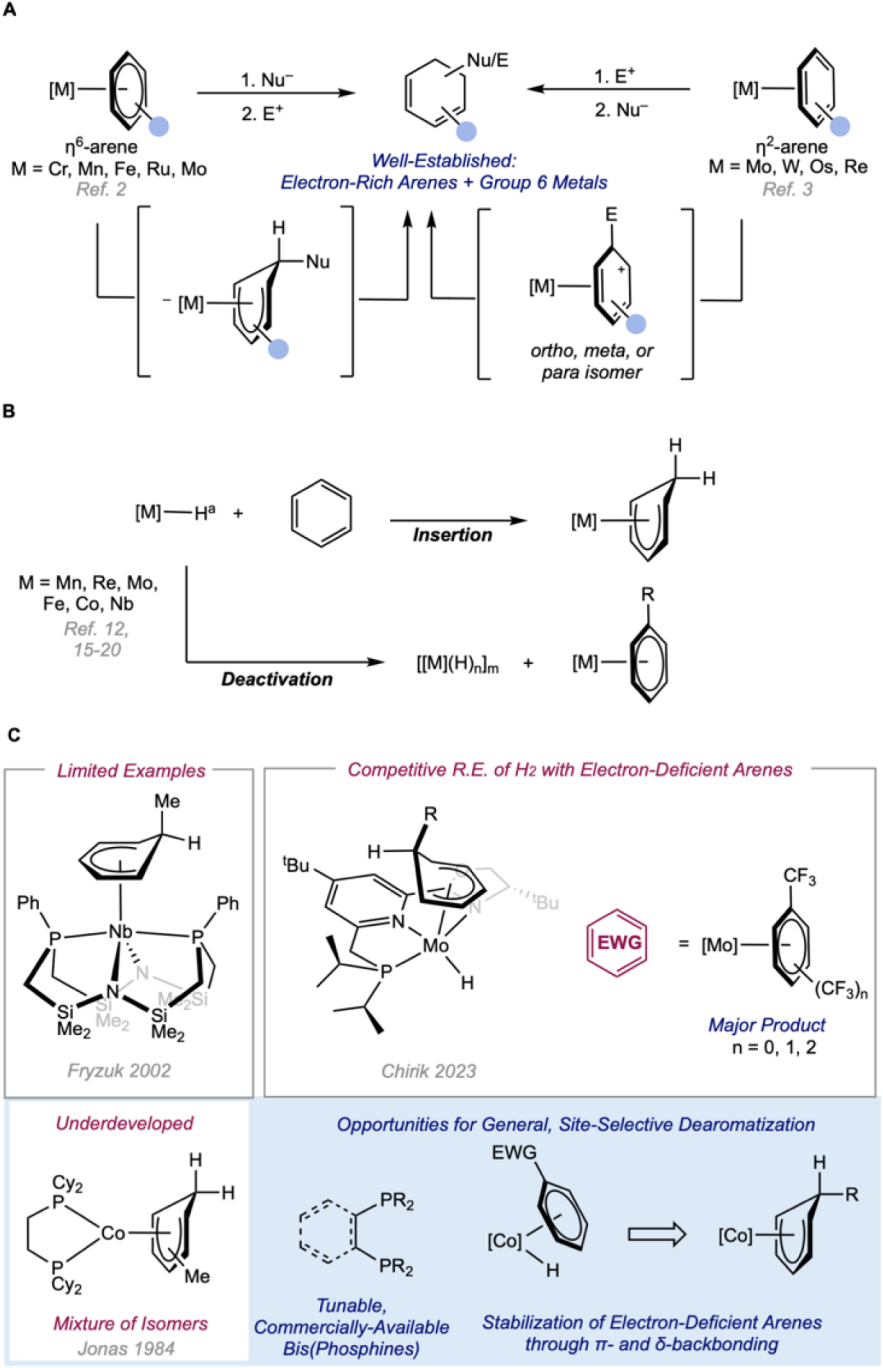
Strategies and Examples of Metal-Mediated Dearomatization of Substituted Benzenes through Addition Reactions and Arene Hydrometallation Strategies. A. Transition Metal-Mediated Dearomatization of Substituted Benzenes. B. Metal–Cyclohexadienyl Complexes Accessible through Arene Insertion into Metal-Hydride Bond. C. Isolable Metal–Cyclohexadienyl Complexes Generated From Dihydrogen^a^

a Including metal dihydrides and polyhydrides for arene insertion.

One promising, yet comparatively less explored, approach for site-selective dearomatization involves the insertion of arenes into metal hydride bonds.^12^ While insertion reactions have been identified as the key selectivity-determining steps in different alkene hydrofunctionalization^13^ and polymerization^14^ reactions, little is known about the reactivity and site selectivity of arene insertion en route to partial hydrogenation products. Access to metal–cyclohexadienyl complexes is rare and typically confined to formally 14-electron metal hydrides (Scheme 1B).^12,15–22^ Examples of arene insertion have been reported with iron,^15^ cobalt,^16^ niobium,^17^ and molybdenum^18^ and are typically limited to alkyl-substituted benzenes (Scheme 1C). A more comprehensive study examining the influence of steric and electronic factors on arene insertion was reported using *in situ*-generated molybdenum phosphino­(oxazoline)­pyridine dihydride complexes (Scheme 1C).^19^ Insertions of electron-rich arenes were more facile, while the corresponding reaction with PhCF_3_ was sluggish and favored reductive elimination of H_2_ and formation of the corresponding molybdenum η^6^-arene complexes. This work highlights the synthetic gap in metal-hydride dearomatization with electronically differentiated arenes.

Transition metals with a higher *d*-electron count are attractive, as both π- and δ-back-bonding may stabilize the incipient η^6^-arene complex prior to insertion.^20^ Seminal reports by Jonas and coworkers described the exposure of (dcype)­Co­(η^3^-C_8_H_12_) (dcype = dicyclohexylphosphinoethane) to H_2_ in the presence of electron-rich arenes, such as benzene, toluene, and *p*-xylene, which formed the corresponding cobalt cyclohexadienyl complex (Scheme 1C).^16^ This system offers a promising strategy for general site-selective dearomatization. Tuning the bis­(phosphine) ligand^21^ can reveal how arene steric and electronic properties govern insertion reactivity, selectivity, and complex deactivation to rationally design a chemoselective dearomatization platform.

To explore this possibility, a series of electron-rich, chiral bis­(phosphine) ligands was evaluated to expand arene compatibility and probe the factors governing selective dearomatization of representative substituted trifluoromethyl benzenes (Scheme 2). *In situ* reaction monitoring revealed distinct kinetic insertion and subsequent isomerization steps. The rates and selectivities were strongly influenced by arene substitution patterns, which were probed through intramolecular competition experiments using disubstituted trifluoromethyl alkylbenzenes, silylbenzenes, and borylbenzenes with differing steric profiles. Protonation of these intermediates was investigated for the release of cyclohexadienes.

**Scheme 2:**
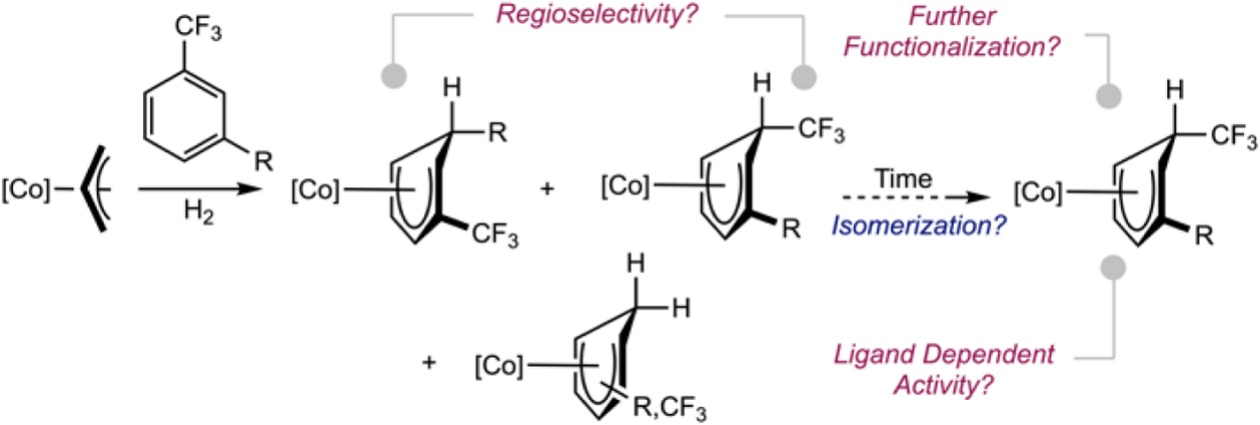
Guidelines for Site-Selective Arene Insertion with Bis­(phosphine) Cobalt Hydrides

## Results and Discussion

### Allyl Synthesis and Reactivity

Bis­(phosphine)­cobalt­(I) allyl complexes were targeted as isolable precursors for the *in situ* generation of the corresponding 14-electron cobalt hydride derivatives designed to promote arene insertion.^16,22^ A modified synthetic route was developed whereby bis­(phosphine)­cobalt­(II) dichloride complexes were treated with (1,4-dioxane)magnesium bis­(allyl) in a reductive allylation sequence (Scheme 3).^22,23^ The use of (1,4-dioxane)­magnesium bis­(allyl) instead of more traditional approaches using allyl Grignard-type reagents sequestered any unproductive halide metathesis reactivity^24,25^ and expanded the scope of the reaction to additional cobalt complexes. Using this approach, (*R,R*)-(^iPr^DuPhos)­Co­(η^3^-C_3_H_5_) (**Co1**), (dcype)­Co­(η^3^-C_3_H_5_) (**Co2**), (*R,R*)-(^iPr^BPE)­Co­(η^3^-C_3_H_5_) (**Co3**), and (*R,R*)-(BenzP*)­Co­(η^3^-C_3_H_5_) (**Co4**) were isolated as diamagnetic, purple, blue-purple, blue, and red solids, respectively, in 58–93% yield (Scheme 3). Each cobalt­(I) allyl complex was characterized by multinuclear NMR spectroscopy and exhibited five diagnostic allylic resonances between 1.15 and 4.50 ppm in the ^1^H NMR spectrum, consistent with an overall *C*_1_ symmetric complex. At ambient temperature, these compounds exhibited a broad resonance between 70.0 and 92.0 ppm in the ^31^P­{^1^H} NMR spectrum, consistent with previous cobalt­(I) allyl reports.^22,26,27^ This spectroscopic behavior is consistent with a dynamic process involving either η^3^-allyl rotation^26,27^ or rapid dissociation to the η^1^ haptomer, rotation, and reassociation of the alkene faster than the NMR time scale.^28^

**Scheme 3:**
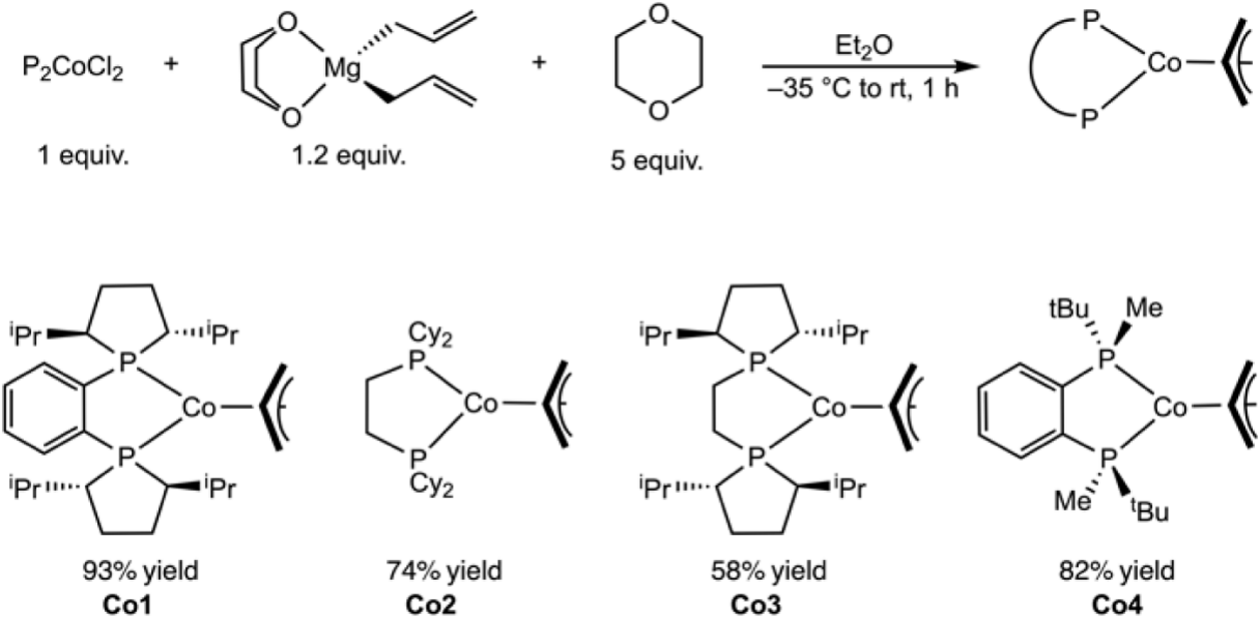
Synthesis of Bis­(phosphine)­Co­(η^3^-C_3_H_5_) Complexes

The solid-state structure of **Co1** was determined by X-ray diffraction and confirmed as a monomeric cobalt­(I) compound with an η^3^-allyl ligand (Figure 1). The Co–C_centroid_ distance of 1.742 Å, as well as individual bond distances of Co–C1 of 2.03(2) Å, Co–C2 of 1.98(2) Å, and Co–C3 of 2.01(1) Å, are similar to those of other previously reported bis­(phosphine) cobalt allyl complexes.^22,23^ The BenzP* derivative, [(*R,R*)-(BenzP*)­Co­(η^3^-C_3_H_5_)]_2_(μ-N_2_) **Co4–N**_**2**_, is dimeric in the solid state with a bridging dinitrogen ligand, and each cobalt in an apparent *anti* configuration around the two-atom bridge. A Co–N–N angle of 169.3(5)° was observed for both cobalt centers. Each cobalt contains an η^3^-allyl with an indistinguishable Co–C_centroid_ distance of 1.743 Å and individual allyl bond distances of Co–C1 of 2.06(1) Å, Co–C2 of 1.95(1) Å, and Co–C3 of 2.07(1) Å. **Co4–N**_**2**_ Co–C_centroid_ bond lengths are consistent with those found in **Co1**. **Co4–N**_**2**_ forms a red-orange solution in methylcyclohexane at −35 °C and undergoes a color change to a dark purple solution upon warming, signaling dissociation of the N_2_ ligand.^29^

**Figure 1:**
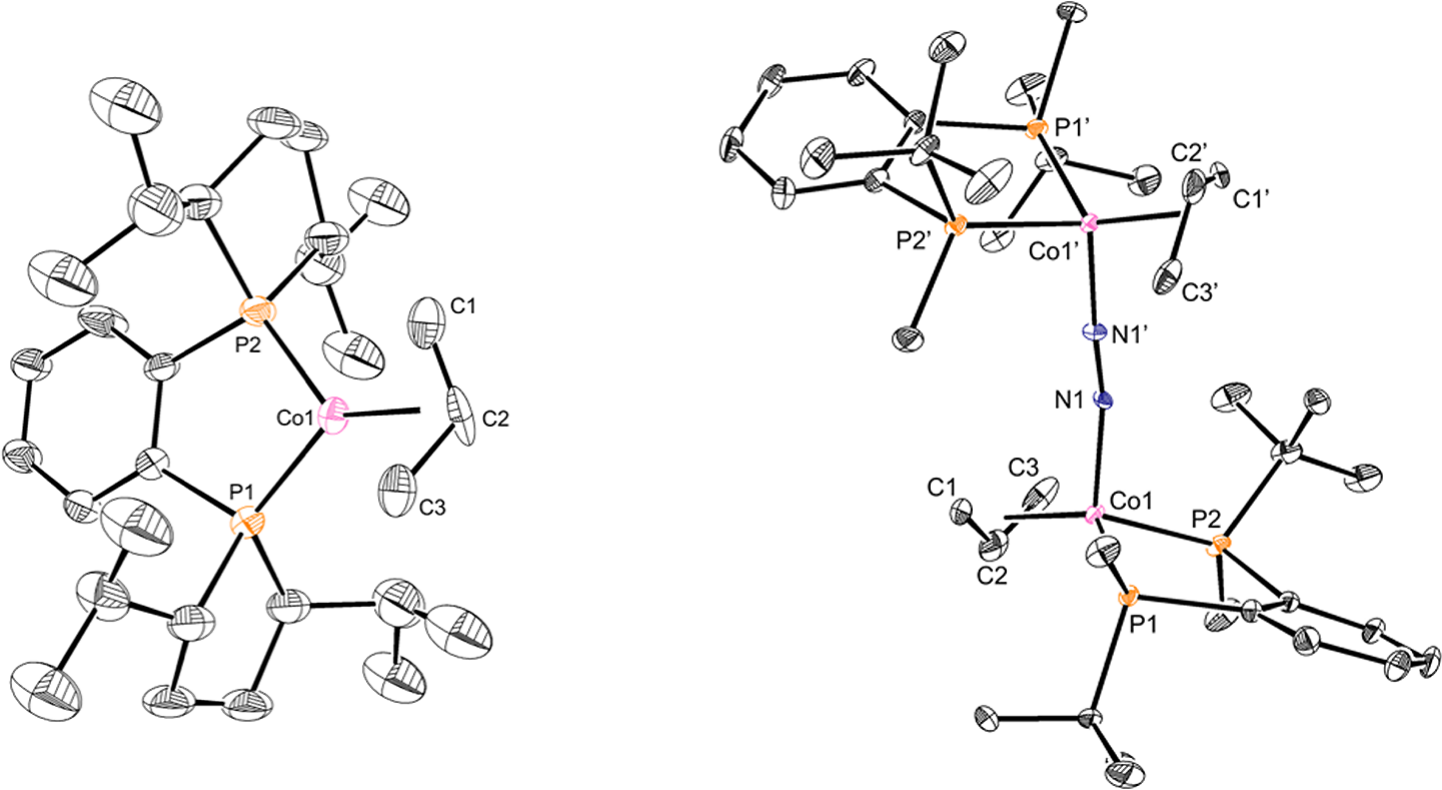
Representations of the solid-state structures of **Co1** (left) and **Co4–N_2_** (right) with 30% probability ellipsoids, with H atoms omitted for clarity.

The hydrogenation reactivity of the cobalt allyl compounds in the absence of arene was assayed to establish the background reactivity. A cyclohexane-*d*_12_ solution of each complex was exposed to 4 atm of H_2_ at 23 °C. In each case, multimetallic cobalt hydride complexes were obtained in 61% to 99% yield along with propane, consistent with previous reports using either **P**_**2**_Co­(I)^16,22^ or **P**_**2**_Co­(II)^30^ precursors (Scheme 4). [(*R*,*R*)-(^iPr^DuPhos)­Co]_2_(μ_2_–H)_3_(H) (**Co1–H**) was reported previously and characterized as a diamagnetic dicobalt­(II) compound^30^ arising from a metal–metal bond between the two cobalt centers.^31^ The achiral compound, [(dcype)­CoH_2_]_*n*_ (**Co2–H;** *n* = 2 or 3), has been observed as both a trimer^16^ or dimer^23^ in solution and in the solid state. The cyclohexane-*d*_12_^1^H NMR spectrum of **Co2–H** at 23 °C is consistent with a diamagnetic compound, supporting a dicobalt­(II) dimer similar to **Co1–H** and previous literature reports using dippp (dippp = 1,3- bis­(diisopropylphosphino)­propane).^22^ Hydrogenation of **Co4** followed by crystallization in a saturated pentane solution at −35 °C resulted in the isolation of a trimeric product, [(*R,R*)-(BenzP*)­CoH_2_]_*n*_ (**Co4–H**; *n* = 2 or 3) (see Supporting Information). Cobalt–cobalt distances of 2.531(1), 2.563(1), and 2.554(1) Å were determined for Co1–Co2, Co2–Co3, and Co1–Co3, respectively, consistent with metal–metal bonds.^31^ The number of hydrides was quantified by a Toepler pump experiment where one equivalent of **Co4–H** was treated with three equivalents of (*R,R*)-(BenzP*)­CoCl_2_, resulting in the formation of [(*R,R*)-(BenzP*)­CoCl]_2_ and H_2_ gas. This experiment produced a 3:1 ratio of combustible gas/cobalt, consistent with six hydrogen atoms per trimer. However, the cyclohexane-*d*_12_^1^H NMR spectrum of **Co4–H** is consistent with a diamagnetic compound. These observations are analogous to the dynamic behavior observed in published reports of **Co2–H**,^16,23^ suggesting equilibration between a trimer and dimer in the solid and solution states, respectively. Hydrogenation of **Co3** generated an intractable mixture of products (**Co3–H**) with no multimetallic metal hydride identified (Scheme 4).

**Scheme 4:**
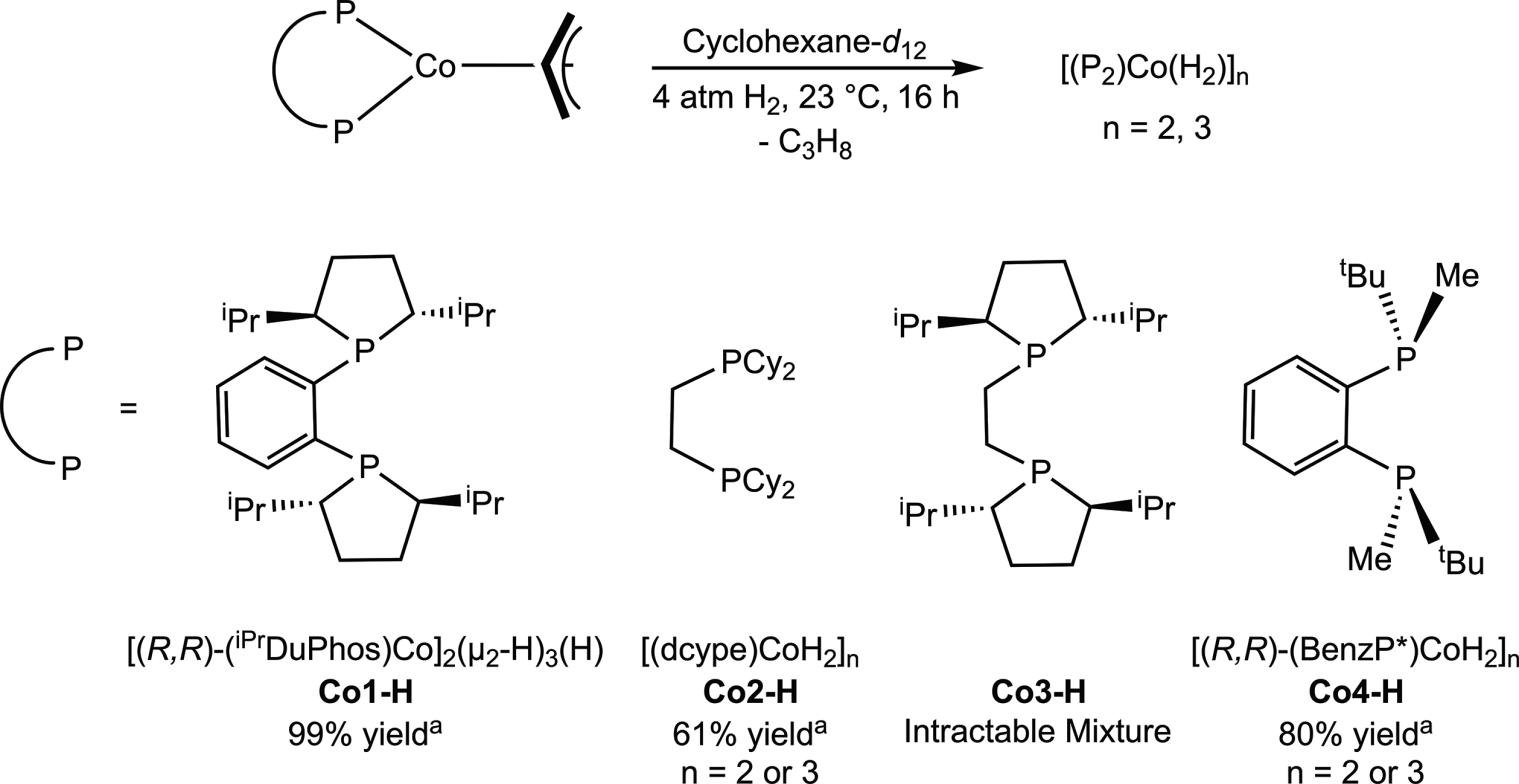
Reactivity of *In Situ*-Formed Bis­(phosphine) Cobalt Hydrides in the Absence of Arene^a^

a Yields determined by empirical formula, mass, and relative integration to the internal standard in the ^1^H NMR spectrum.

Complexes **Co1–H** through **Co4–H** were inactive toward arene insertion (see Supporting Information), consistent with earlier reports that demonstrate arene insertion only occurs from a cobalt­(I) monohydride.^16^ In addition to the identity of the cobalt hydride, the coordination number also plays a role in enabling insertion over deactivation. Previous studies described (η^3^-C_3_H_5_)­Co­(P­(OR)_3_)_3_ as an active precatalyst for arene hydrogenation,^32^ though the formation of nanoparticles is likely.^33^ Under an atmosphere of H_2_ and excess arene, a Co­(I) monohydride, HCo­[P­(OR)_3_]_4_, was observed as the major byproduct and was inactive toward arene hydrogenation.^26,27^ As demonstrated in this and previous work,^16,22,26,27,32^ 14-electron cobalt hydrides are uniquely poised to promote arene dearomatization and further reduction.

### Arene Insertion: Reactivity and Selectivity

Studies into insertion reactivity commenced with PhCF_3_ as this arene has proven incompatible with previous metal-hydride insertion methods^19^ and is of fundamental interest given the electron-deficient nature of the arene. Cyclohexane-*d*_12_ solutions of **Co1**–**Co4** containing 10 equiv of PhCF_3_ were exposed to 4 atm of H_2_, and the reactions were monitored by NMR spectroscopy (Scheme 5).

**Scheme 5:**
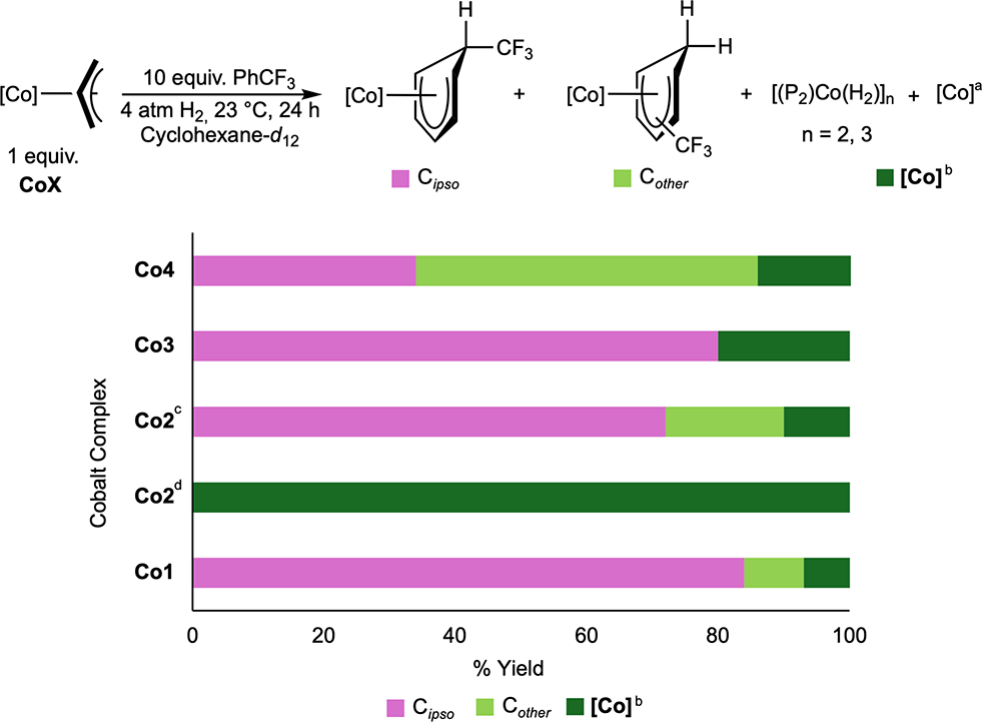
Reactivity of *In Situ*-Formed Bis­(Phosphine) Cobalt Hydrides with PhCF_3_ ^a^ ^b^ ^c^ ^d^

a [Co] = mass unaccounted for.
b Quantified by the difference between the known insertion products and the mass unaccounted for, including the formation of cobalt hydride.
c 0.5 atm H_2_.
d Exclusively cobalt hydride, **Co2–H**, observed.

Notably, the insertion reaction with **Co1** was both facile and selective, producing (*R,R*)-(^iPr^DuPhos)­Co­(η^5^-C_6_H_6_CF_3_) (**Co1-a**) in 93% yield with 84% *ipso*-selectivity after 24 h. The structurally related (*R,R*)-^iPr^BPE complex, **Co3**, also exhibited facile insertion reactivity and generated (*R,R*)-(^iPr^BPE)­Co­(η^5^-C_6_H_6_CF_3_) (**Co3-a**) 90% mass-balance accounted for and with 89% *ipso*-selectivity, highlighting the performance of chiral phospholane-based ligands for site-selective arene insertion. In contrast, the cobalt hydride **Co2–H** was obtained upon treatment of the achiral complex **Co2** with 4 atm of H_2_. Reducing H_2_ pressure from 4 to 0.5 atm resulted in 90% conversion to (dcype)­Co­(η^5^-C_6_H_6_CF_3_) (**Co2-a**), with 80% selectivity for the *ipso*-CF_3_ site. (*R,R*)-(BenzP*)­Co­(η^5^-C_6_H_6_CF_3_) (**Co4-a**) was formed initially in 88% yield, with 48% of **Co4-a** insertion in the *ipso* position after 24 h and 17% loss of mass balance. Varying the H_2_ pressure for the synthesis of **Co1-a**, **Co3-a**, and **Co4-a** did not alter the observed site selectivity of the insertion reaction. Characteristic doublets were observed by ^19^F NMR spectroscopy at −80.60 ppm (^3^ *J*_F–H_ = 7.8 Hz) for **Co1-a**, −80.46 ppm (^3^ *J*_F–H_ = 8.5 Hz) for **Co2-a**, −80.63 ppm (^3^ *J*_F–H_ = 7.5 Hz) for **Co3-a**, and −80.39 (^3^ *J*_F–H_ = 7.9 Hz) for **Co4-a**. Full characterization data for each complex are reported in the Supporting Information.

The role of the supporting phosphine ligand in the site selectivity of the insertion reaction was established. For example, the presence of a sterically demanding *tert*-butyl substituent on the phosphine eroded insertion site selectivity, as evidenced by the isomer distribution of **Co4-a**. Peak broadening was observed in the ^1^H NMR spectra of **Co2-a**, **Co3-a**, and **Co4-a**, suggesting decomposition into paramagnetic and NMR-silent products over time. Such reactivity was not observed with **Co1-a,** demonstrating that the rigid aromatic backbone and distal steric bulk of (*R,R*)-^iPr^DuPhos stabilize the cyclohexadienyl complex (see Supporting Information). Subsequent studies focused on **Co1** to further investigate the steric and electronic effects of arene insertion reactivity and regioselectivity.

Crystallization of **Co1-a** proved challenging due to its physical properties as an amorphous solid at various temperatures ranging from −35 °C to ambient temperature. One-electron oxidation to the corresponding cationic cobalt­(II) by addition of one equivalent of AgSbF_6_ to a diethyl ether solution of **Co1-a** produced a silver mirror and a red product identified as [(*R,R*)-(^iPr^DuPhos)­Co­(η^5^-C_6_H_6_CF_3_)]­[SbF_6_] (**Co1-a** ^**+**^). A THF-*d*_8_ solution magnetic moment of 2.1(6) μ_B_ measured at 23 °C is consistent with one unpaired electron and a low-spin cobalt­(II) complex. The solid-state structure of **Co1-a** ^**+**^ was determined by X-ray diffraction (Figure 2) and confirmed *ispo*-CF_3_ insertion with no close contacts between the cobalt cation and the SbF_6_^–^ anion. The isomer observed in the solid-state structure of **Co1-a** ^**+**^ is consistent with the NMR spectroscopic data and assignments for the diamagnetic cobalt­(I) derivative, **Co1-a**, supporting a lack of isomerization upon oxidation to cobalt­(II).

**Figure 2:**
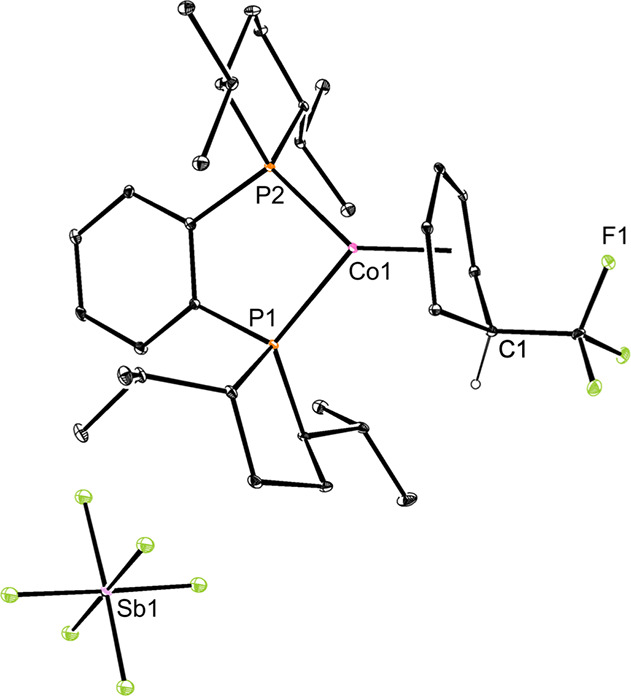
Solid-state structure of **Co1-a^+^** at 30% probability of ellipsoids. Hydrogen atoms, except those on C1, are omitted for clarity.

To investigate the origin of the observed site selectivity, the insertion of PhCF_3_ was monitored over time. A cyclohexane-*d*_12_ solution of **Co1** containing 10 equiv of PhCF_3_ was exposed to 4 atm of H_2_, and the progress of the insertion reaction was monitored by ^1^H and ^19^F NMR spectroscopy (Scheme 6). After 40 min, a mixture of *ortho* (32%), *meta* (20%), and *ipso* (48%) CF_3_ isomers of **Co1-a** was observed. After 4 h at 23 °C, this mixture of isomers converted to the major (87%) *ipso*-CF_3_ product. No additional change in the product ratio was observed upon standing for 4 days, consistent with the formation of the thermodynamic product.

**Scheme 6:**
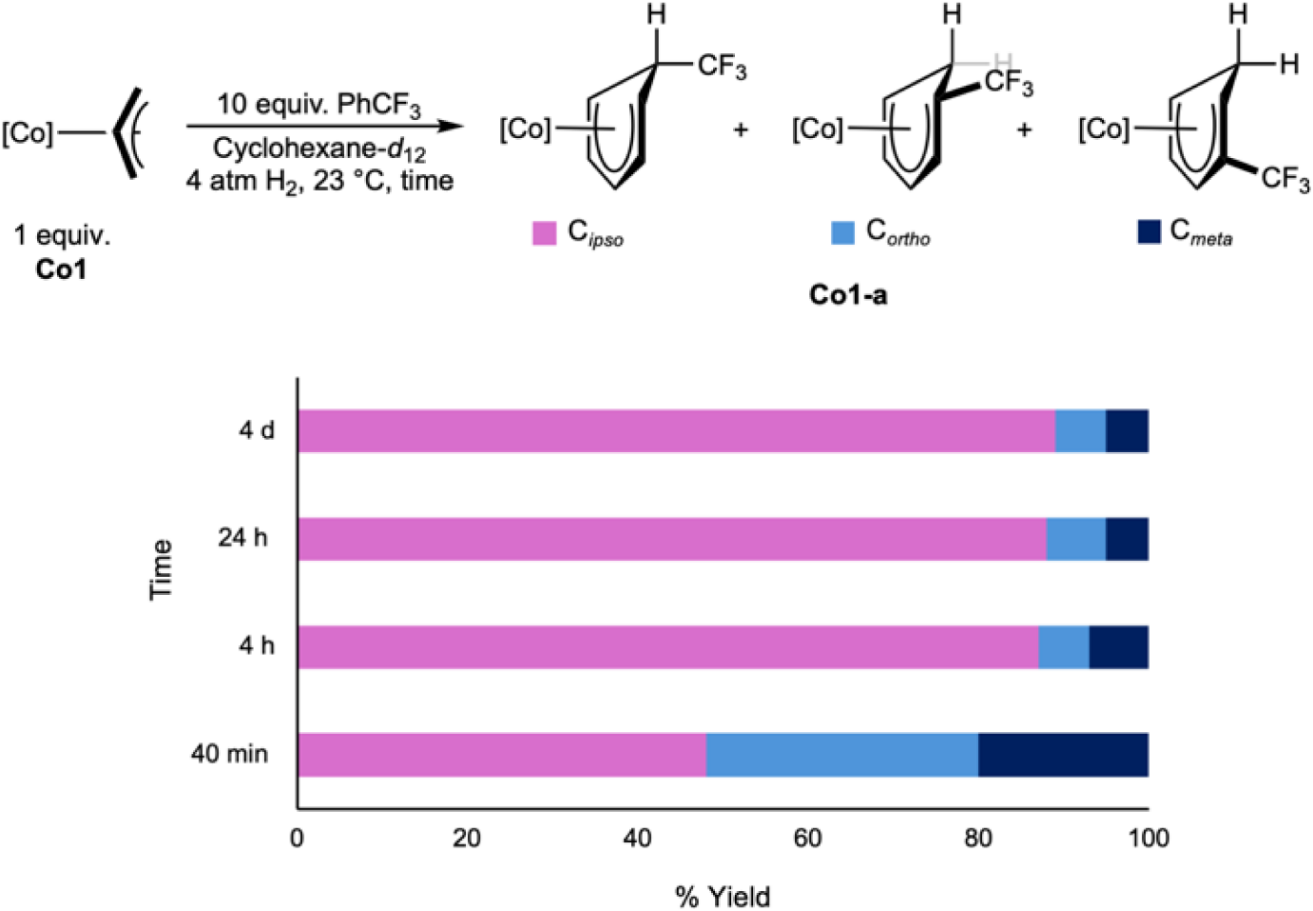
Observation of **Co1-a** Isomers as a Function of Time

These results demonstrate that the kinetic hydrometalation of PhCF_3_ is aselective, producing a mixture of isomers that, through reversible β-hydride elimination, rotation, and reinsertion events, undergo isomerization to the thermodynamic isomer where the C­(sp^3^)-bond is formed at the *ipso* carbon. While this reactivity is established for alkenes using [(η^5^-C_5_H_5_)_2_Zr­(H)]­Cl]_2_ to generate terminal alkyl hydrozirconation products,^34,35^ analogous studies with arenes are underdeveloped, as metal-hydride complexes that insert arenes reversibly are rare.^16–18^ A notable example is a niobium­(III) complex supported by a bis­(phosphine) bis­(silylamido)­(P_2_N_2_) macrocycle, where the transiently generated niobium­(III) monohydride produces a kinetic mixture of toluene insertion products that isomerize to the thermodynamically preferred *ipso*-methyl isomer over time.^17^

To determine if the isomerization of **Co1-a** was inter- or intramolecular, exchange experiments were conducted. Benzene was selected as the representative arene due to the availability of the perdeuterated isotopologue. The corresponding cobalt cyclohexadienyl complex derived from benzene insertion, (*R,R*)-(^iPr^DuPhos)­Co­(η^5^-C_6_H_7_) (**Co1-b**) was synthesized by exposure of a cyclohexane-*d*_12_ solution of **Co1** containing 20 equiv of benzene to 1 atm of H_2_ at ambient temperature (Scheme 7). Isolated **Co1-b** was dissolved in benzene-*d*_6_ and the progress of the reaction was monitored by NMR spectroscopy at 23 °C. After 48 h, 68% conversion to (*R,R*)-(^iPr^DuPhos)­Co­(η^5^-C_6_D_6_H) (**Co1-b**_**D**_) was determined by integration to an internal standard in the^1^H NMR spectrum and the release of C_6_H_6_ by the appearance of a singlet at 128.6 ppm in the ^13^C­{^1^H} NMR spectrum. These results establish slow but reversible insertion that involves the loss of free arene. In the case of PhCF_3_, it is unlikely that arene dissociation occurs following β-hydride elimination from the kinetic products, in agreement with observations with niobium^17^ and achiral bis­(phosphine) cobalt­(I) complexes.^16^

**Scheme 7:**
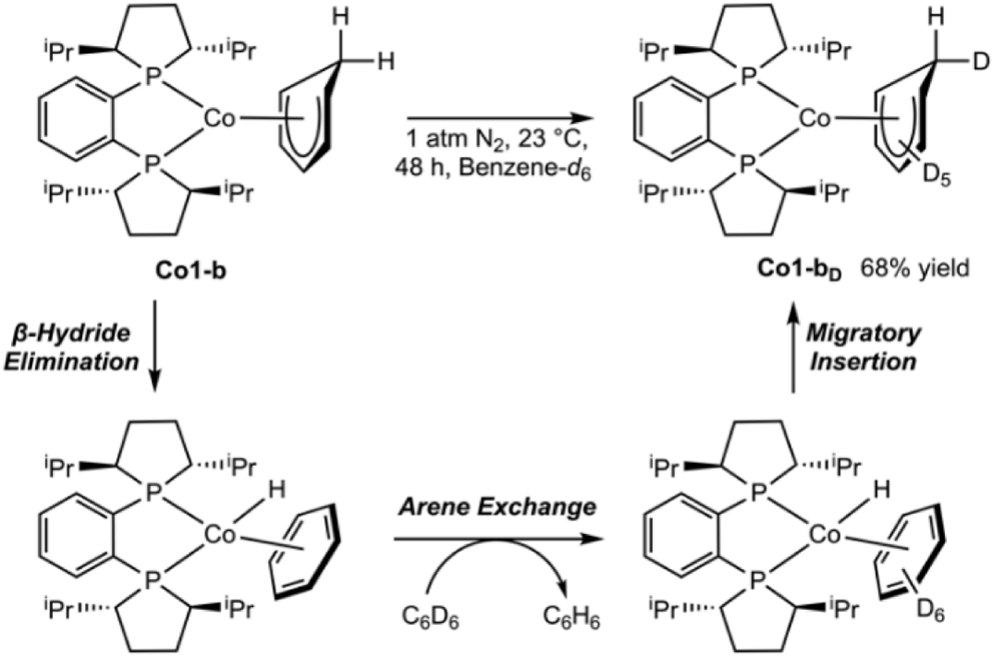
Exchange of Benzene for Benzene-*d*_6_ Observed over Time from Isolated **Co1-b**

To generalize reactivity and further delineate the relative steric and electronic contributions, the site selectivity was examined as a function of the arene substituent. A series of alkyl-substituted benzenes, including methyl, ethyl, isopropyl, and *tert*-butyl benzene, was studied. In each case, a cyclohexane-*d*_12_ solution of **Co1** containing 10 equiv of the arene was prepared, and the reaction mixture was exposed to 4 atm of H_2_ at 23 °C and monitored by NMR spectroscopy (Scheme 8). This protocol minimized the formation of **Co1–H** to <10% and enabled the evaluation of site selectivity as a function of the alkyl substituent. In each case, the selectivity of the insertion reaction was assayed after 16 h.

**Scheme 8:**
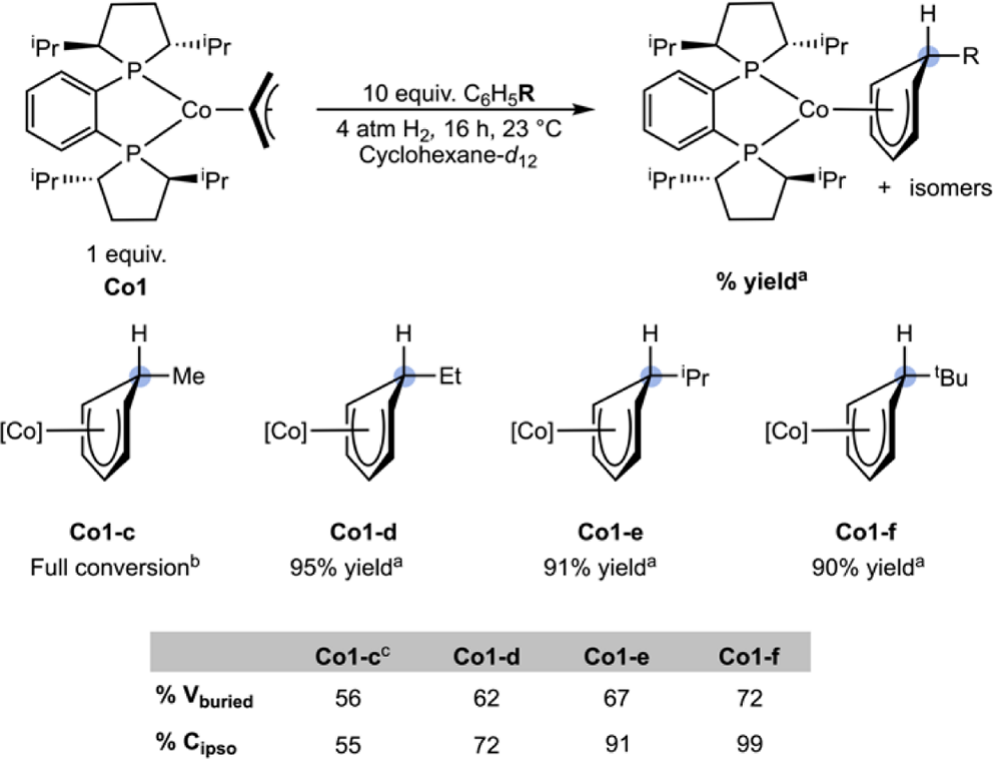
Insertion Reactivity of **Co1** with Alkyl Benzenes^a^ ^b^ ^c^

a % yield reported as a mixture of isomers.
b Full conversion of **Co1** observed.
c %C*_ipso_* assigned by relative integration.

The site selectivity of the arene insertion reaction increased as a function of the size of the alkyl group, as measured by the percent buried volume (%*V*_buried_ = smallest conformation of a functional group within 3.5 Å of the *ipso* carbon center),^36^ consistent with reported molybdenum^19^ and niobium examples.^17^ With toluene, multiple isomers of (*R,R*)-(^iPr^DuPhos)­Co­(η^5^-C_6_H_6_Me) (**Co1-c**) were observed, with the major (55%) product derived from insertion at the *ipso* position. Other regioisomers of the cobalt cyclohexadienyl product were challenging to assign due to overlapping resonances between isomers and with free toluene. Attempts to isolate **Co1-c** produced an intractable mixture, as judged by ^1^H NMR spectroscopy. Increased site selectivity was observed with the insertion of ethylbenzene, as the major isomer of (*R,R*)-(^iPr^DuPhos)­Co­(η^5^-C_6_H_6_Et) (**Co1-d**), constituted 72% of the product mixture and afforded the *ipso* insertion product. Six signals were observed between 3.13 and 5.17 ppm and were assigned to the η^5^-C_6_H_6_Et ligand, with a diagnostic ^1^H NMR resonance between 2.25 and 2.16 ppm correlating to the *ipso*-C­(sp^3^)–H at 42.73 ppm in the ^13^C­{^1^H} NMR spectrum. The insertions of *iso*-propylbenzene and *tert*-butylbenzene were highly selective for the *ipso* position, with (*R,R*)-(^iPr^DuPhos)­Co­(η^5^-C_6_H_6_^i^Pr) (**Co1-e**) and (*R,R*)-(^iPr^DuPhos)­Co­(η^5^-C_6_H_6_^t^Bu) (**Co1-f**) formed with 91% and 99% selectivity, respectively. Complexes **Co1-d**, **Co1-e**, and **Co1-f** were vacuum stable. At early time points in the formation of **Co1-e** and **Co1-f**, a mixture of isomers was observed, which converted to the major isomer over 1 hour at 23 °C (see Supporting Information). No change in the ratio of the products was observed upon additional standing at 23 °C.

Intramolecular competition experiments were conducted with **Co1** using disubstituted arenes bearing alkyl, silyl, boryl, and trifluoromethyl substituents, with representative examples of both 1,2- and 1,3-disubstituted benzenes, to distinguish between steric and electronic effects during the insertion reaction. Reactions were assayed by NMR spectroscopy (Scheme 9). High site selectivity was observed *ipso* to trifluoromethyl groups among 1,3-disubstituted trifluoromethyl benzenes, with near-exclusive *ipso*-CF_3_ insertion with ^i^Pr (**Co1-g**), Me (**Co1-h**), boronate ester (**Co1-i**), CF_3_ (**Co1-j**), SiMe_3_ (**Co1-k**), and ^i^Bu (**Co1-l**) substituents. Near-equimolar mixtures of diastereomers were observed in each case after 24 h, where the identity of the diastereomers was not readily assigned due to the complexity of the spectra. Structural confirmation for the formation and isomer identity of **Co1-h** was obtained by oxidation using the same procedure as for **Co1-a** ^+^. The resulting cobalt­(II) complex, [(*R,R*)-(^iPr^DuPhos)­Co­(η^5^-C_6_H_4_CF_3_Me)]­[SbF_6_] (**Co1-h** ^**+**^), exhibited overall *C*_1_ symmetry as the *re* diastereomer, with the insertion at the *ipso*-CF_3_ position (Figure 3).

**Scheme 9:**
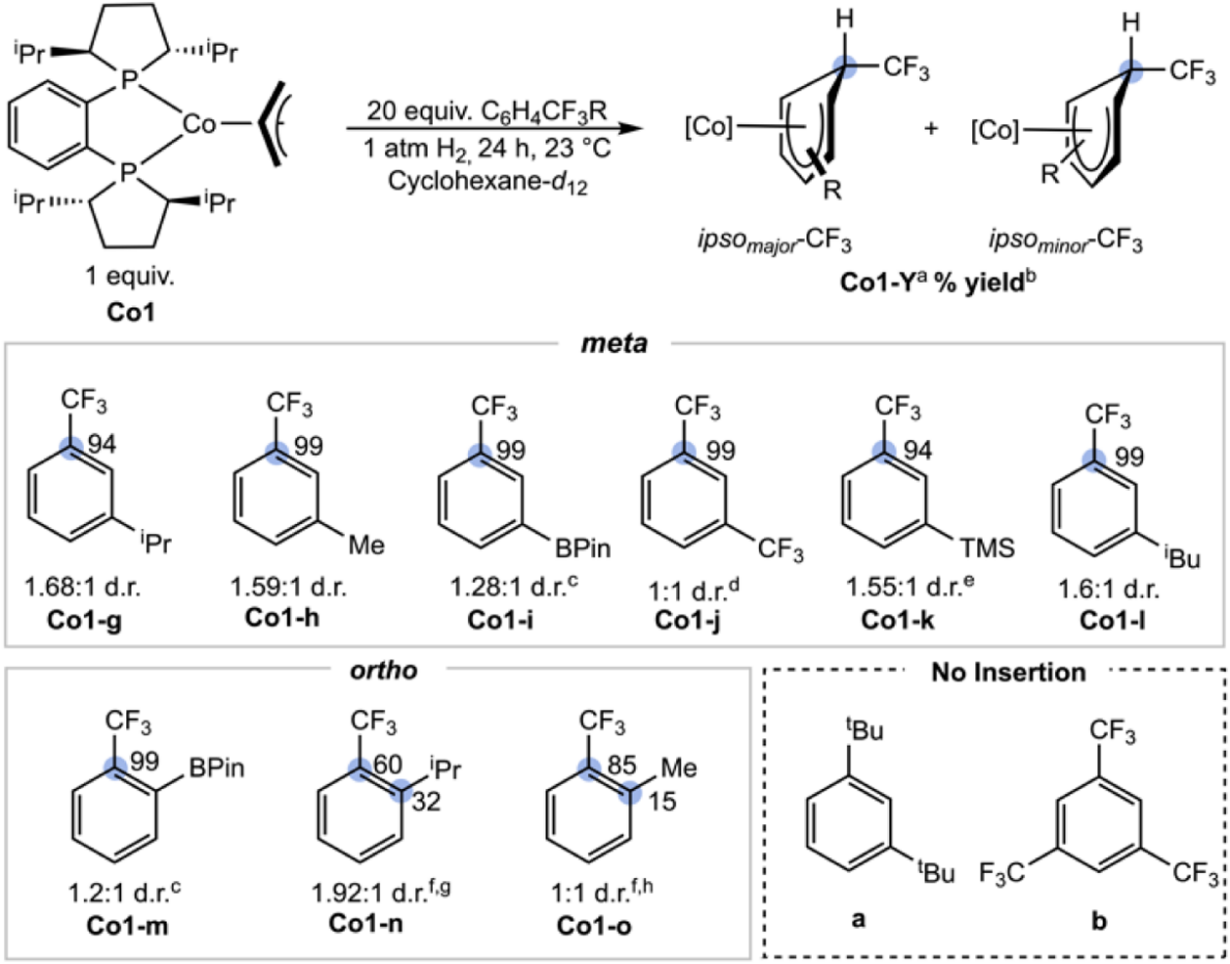
Reactivity of **Co1** with Disubstituted Trifluoromethyl Benzenes^a^ ^b^ ^c^ ^d^ ^e^ ^f^ ^g^ ^h^

a Y = g-o.
b The percent yield is the sum of both diastereomers at the site of insertion, where blue circles depict site selectivity.
c 4 atm H_2_.
d 6% **Co1–H** and 24% mass balance unaccounted.
e 9% unidentified paramagnetic [Co].
f 5 days.
g 12% mass balance unaccounted.
h 11% mass balance unaccounted.

**Figure 3:**
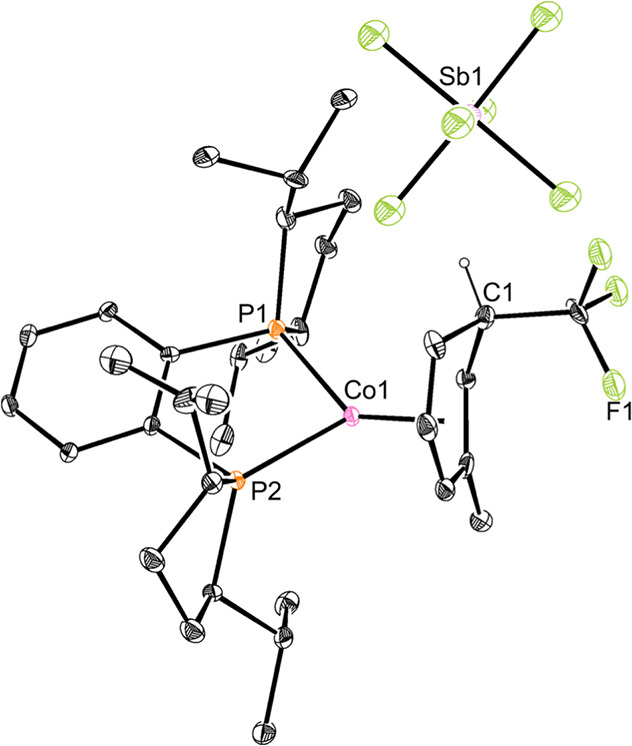
Solid-state structure of **Co1-h^+^** at 30% probability ellipsoids. Hydrogen atoms, except those on C1, are omitted for clarity.

A 1,2-disubstituted benzene bearing a boronate ester (**Co1-m**) retained high (99%) *ipso*-CF_3_ selectivity, although eroded *ipso*-CF_3_ site selectivities of 60% and 85% were observed for alkyl groups, such as ^i^Pr (**Co1-n**) and Me (**Co1-o**), demonstrating that the thermodynamic selectivities were influenced by steric, electronic, and positional effects (Scheme 9). This is a rare example of preferential hydride insertion, *ipso-*CF_3_, even in the presence of more sterically demanding substituents, establishing a significant electronic preference. The high *ipso*-CF_3_ selectivity likely arises from stabilization of the resulting cyclohexadienyl complex by increased metal–carbon bond polarization, which strengthens the resultant metal–carbon bond^37^ as well as stabilization of the negative charge at the β-carbon during insertion.^38^ The position of the arene substituents either amplifies or reduces the electronic effects through competing steric interactions with the *C*_2_-symmetric (*R,R*)-^iPr^DuPhos ligand.

To gain deeper insight into the kinetic and thermodynamic product selectivity as a function of position, as well as steric and electronic effects, the isomerization reactivity of **Co1-g**, **Co1-h**, **Co1-n**, and **Co1-o** was studied over time (Scheme 10). A cyclohexane-*d*_12_ solution containing **Co1** and 20 equiv of arene was prepared, exposed to 1 atm of H_2_, and monitored by ^1^H and ^19^F NMR spectroscopy at 23 °C. Each compound formed a mixture of cobalt cyclohexadienyl products that were identified as both *ipso*-CF_3_ diastereomers (***ipso***_**major**_ **-CF**_**3**_ and ***ipso***_**minor**_ **-CF**_**3**_), the major *ipso-*alkyl diastereomer (***ipso***_***major***_ **-alkyl**), and minor *ipso*-alkyl and unsubstituted C­(sp^3^)-H insertion (***ipso*** **-minor**). Assignments of cobalt facial preferences, denoted as major and minor, are relative and were made through multinuclear and correlation NMR spectroscopic experiments. After 5 min at 23 °C, comparable yields of ***ipso***_**minor**_ **-CF**_**3**_ (32%) and ***ipso***_***major***_ **-alkyl** (42%) were observed for **Co1-g**, with the formation of ***ipso***_**major**_ **-CF**_**3**_ (11%) at 85% conversion of **Co1** (Scheme 10A). Full conversion of **Co1** was achieved at 7.5 h, after which time the yield of ***ipso***_**minor**_ **-CF**_**3**_ (35%) marginally increased, while the yield of ***ipso***_***major***_ **-CF**_**3**_ (59%) increased and ***ipso***_***major***_ **-alkyl** (4%) significantly decreased. The resulting product distributions remained consistent for 5 days with <10% loss of mass balance. The product distribution for **Co1-h** was similar to that for **Co1-g,** with the exception that 11% of the initial insertion occurred at unsubstituted positions. These data support sterically driven kinetic insertion products, and because the %*V*_buried_ for ^i^Pr is greater than CF_3_, the isomerization to the ***ipso***_***major***_ **-CF**_**3**_ position is therefore electronically driven.^39^

**Scheme 10:**
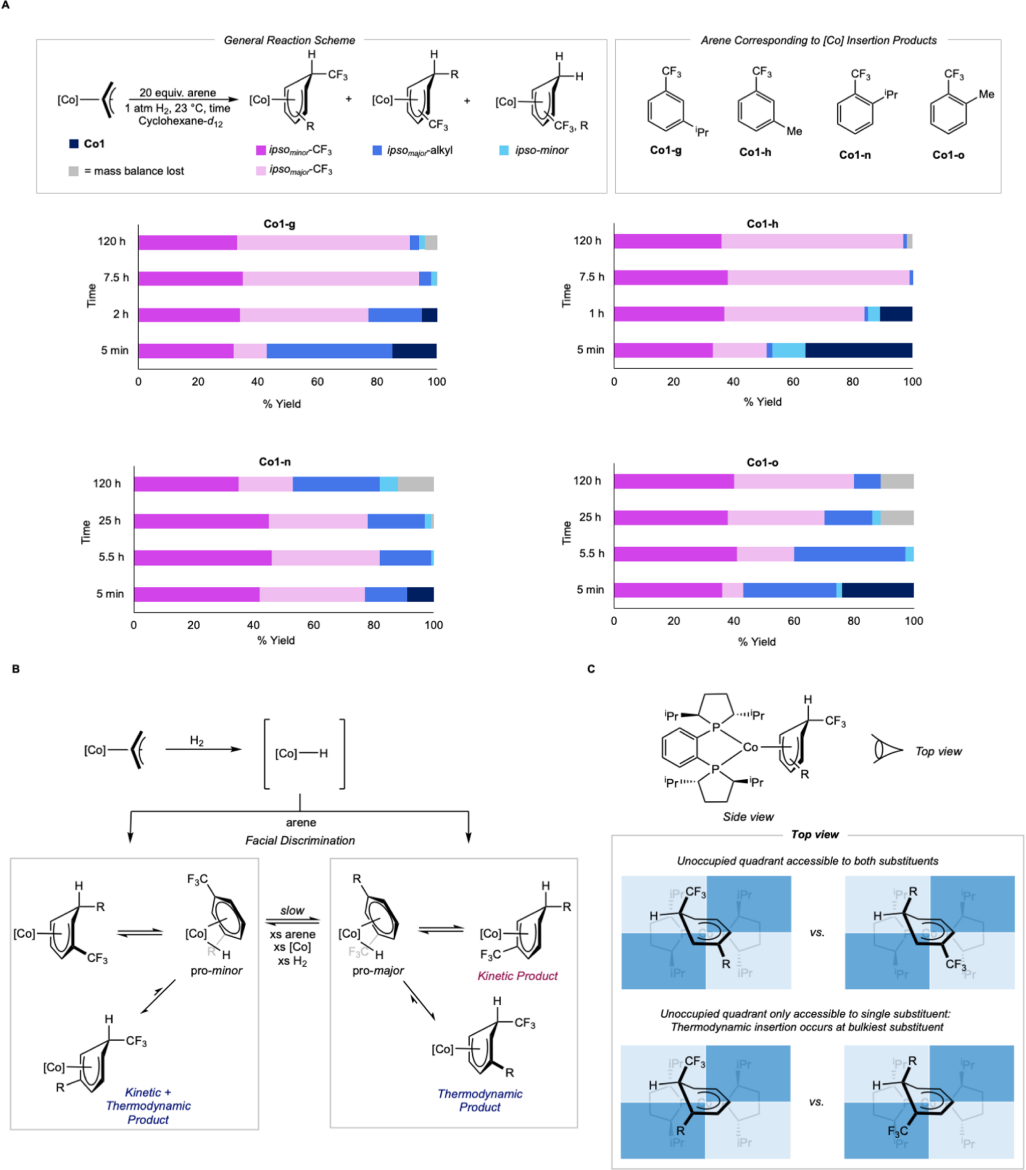
Investigation of Kinetic and Thermodynamic 1,2- and 1,3-Disubstituted Me and ^i^Pr Trifluoromethyl Benzene Insertion Products. A. 1,2- and 1,3-Disubstituted Benzene Time Course. B. Proposed Mechanism of Isomerization. C. Proposed Rationalization of Eroded 1,2-Disubstituted Benzene Selectivity

The steric profile of the substituent influenced both the kinetic and thermodynamic selectivities of the formation of cobalt cyclohexadienyl complexes **Co1-n** and **Co1-o**. At 5 min, both *ipso*-CF_3_ diastereomers of **Co1-n**, ***ipso***_***major***_ **-CF**_**3**_ (42%) and ***ipso***_***minor***_ **-CF**_**3**_ (35%), were observed in comparable yields, with only 14% yield of ***ipso***_***major***_ **-alkyl** observed at 91% conversion of **Co1**. At full conversion after 5.5 h, only minor changes in the product distribution were observed. Continued monitoring of **Co1-n** for 5 days by NMR spectroscopy revealed an increased yield of ***ipso***_***major***_ **-alkyl** to 32%, with a comparable decrease in the amount of ***ipso***_***minor***_ **-CF**_**3**_ (18% yield). At 5 min, **Co1-o** showed similar product distributions to **Co1-g** and **Co1-h,** with products ***ipso***_***minor***_ **-CF**_**3**_ (37%) and ***ipso***_***major***_ **-alkyl** (31%), reflecting a sterically driven insertion, along with low yields of ***ipso***_***major***_ **-CF**_**3**_ (7%) observed at 76% conversion of **Co1**. After 5.5 h and full conversion, ***ipso***_***minor***_ **-CF**_**3**_ maintained similar yields (41%), with an increase in ***ipso***_***major***_ **-CF_3_** (19%) and ***ipso***_***major***_ **-alkyl** (37%) yields. Continuing reaction monitoring for 5 days by NMR spectroscopy revealed diverging reactivity to **Co1-n**, where both **Co1-o** *ipso*-CF_3_ diastereomers— ***ipso***_***major***_ **-CF**_**3**_ (45%) and ***ipso***_***minor***_ **-CF**_**3**_ (40%)—increased relative to *ipso*-alkyl insertion. Increased *ipso*-alkyl insertion for **Co1-n** relative to **Co1-o** corresponds to an increased %V_buried_ of the alkyl substituent. This steric effect is limited to 1,2-disubstituted benzenes.

Based on these data, a mechanism is proposed that accounts for both the formation of the diastereomers and the isomerization to the thermodynamic product, which favors highly selective *ipso*-CF_3_ insertion (Scheme 10B). Following the release of propane, arene coordination to the cobalt occurs from either the *si* or the *re* face (denoted as *major* or *minor*). The observed kinetic products arise from arene coordination to the cobalt through both faces. Previous studies have shown that both alkyl complexes with α-electron-withdrawing groups (EWGs) form strong metal–carbon bonds and that α-EWGs on alkenes are known to slow the rates of β-hydride elimination with late transition metals.^40,41^ The relative rates of alkyl- and trifluoromethylbenzene isomerization are consistent with these trends. When the ***ipso***_***minor***_ **-CF**_**3**_ forms, isomerization is minimal, consistent with ***ipso***_***minor***_ **-CF**_**3**_ being both the kinetic and thermodynamic product formed. The proposed conversion of *ipso*-alkyl to *ipso*-CF_3_ products is likely intramolecular, as benzene-exchange experiments with isolated **Co1-b** with benzene-*d*_6_ revealed only 68% exchange after 48 h (Scheme 7). These results, combined with the increased yield of ***ipso***_***major***_-**CF**_**3**_ corresponding to decreased ***ipso***_***major***_ **-alkyl** over time, support ***ipso***_***major***_-**CF**_**3**_ as the thermodynamic product from the opposite face of the cobalt. The eroding *ipso*-CF_3_ selectivity as a function of substituent size for 1,2-disubstituted benzenes is rationalized by a quadrant model, where only a single substituent occupies an open quadrant compared to two substituents for 1,3-disubstituted benzenes (Scheme 10C). This rationalization is supported by the observation that ***ipso***_***major***_ **-alkyl** selectivity increases in **Co1-n** over time and decreases in **Co1-o.**

### Generation of Free Cyclohexadienes by Protonation

The protonation of the cobalt cyclohexadienyl complexes was explored to release the corresponding cyclohexadiene. Previous studies have shown bis­(phosphine)­Co­(COD) (COD = 1,5-cyclooctadiene) precatalysts form isolable, 18-electron Co­(II) κ^2^-bis­(carboxylates) in the presence of dihydrogen and carboxylic acids.^42^ Treatment of a benzene-*d*_6_ solution of **Co1-a** with 2.5 equiv of benzoic acid resulted in an immediate color change to translucent red and clean formation of 5-(trifluoromethyl)­cyclohexa-1,3-diene (**1**) (Scheme 11A). Compound **1** was isolated in 55% yield following distillation and exhibited four diagnostic alkene resonances between 5.30 and 5.77 ppm in the ^1^H NMR spectrum. A doublet was observed at −72.57 ppm (^3^ *J*_F–H_ = 9.5 Hz) in the ^19^F NMR spectrum and a quartet was observed at 38.32 ppm (^2^ *J*_C–F_ = 27.7 Hz) in the ^13^C­{^1^H} NMR spectrum for the *ipso*-C­(sp^3^)–CF_3_. This peak correlated to a multiplet at 2.68 ppm (^3^ *J*_F–H_ = 9.2 Hz) in the ^1^H NMR spectrum and provided additional support for the proposed structure. The coupling pattern is consistent with a C­(sp^3^)–H *ortho* to two diastereotopic protons, signaling desymmetrization of the cyclohexadiene and supporting the assignment of **1** as the sole product.

**Scheme 11:**
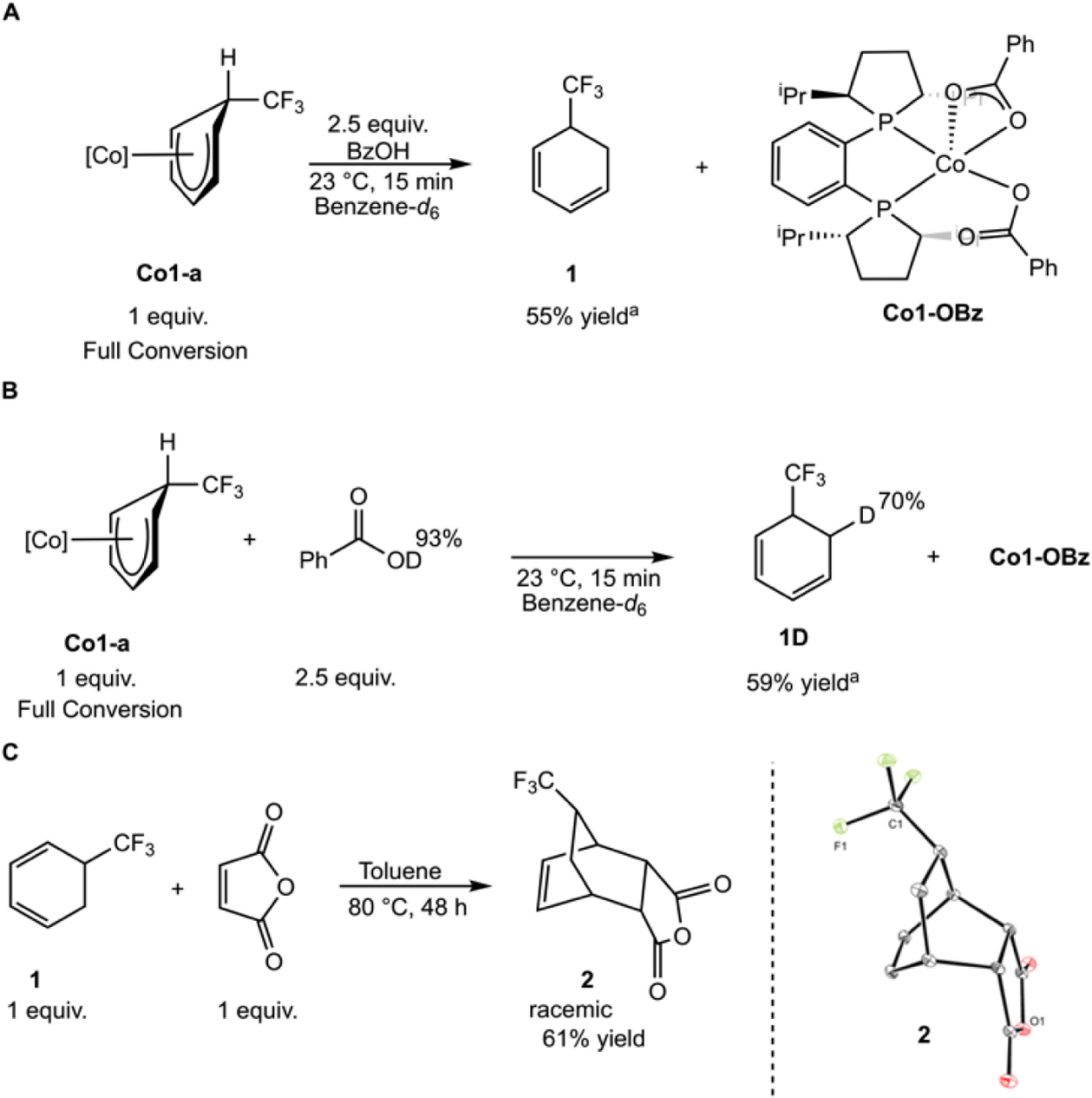
Protonation and Deuteration of **Co1-a**. A. Protonation of **Co1-a** using Benzoic Acid. B. Deuteration of **Co1-a** using Benzoic Acid-*d*. C. Cycloaddition of Isolated **1**

a Exclusive product observed in NMR spectra.

Repeating the protonation reaction with benzoic acid-*d* (Sigma-Aldrich: 98%; 93% observed deuteration from relative integration of the ^1^H NMR spectrum) resulted in 70% deuterium incorporation *ortho* to the CF_3_ substituent (**1D**) with no evidence of deuteration elsewhere in the product (Scheme 11B). The site of deuterium incorporation was established by ^2^H NMR spectroscopy, and a single peak was observed at 1.89 ppm in a benzene solution. The percent deuterium incorporation was determined by integration of the ^1^H NMR spectrum and was confirmed by quantitative ^13^C­{^1^H} NMR spectroscopy. A 1:1:1 triplet of quartets (^*1*^ *J*_C–D_ = 20.4 Hz; ^4^ *J*_F–D_ = 5.7 Hz) was observed at 21.92 ppm in toluene-*d*_8_, consistent with the formation of a C­(sp^3^)–D bond *ortho* to CF_3_. There are several possibilities to account for the lower deuterium incorporation observed in the product than in the starting benzoic acid.^43^ A primary kinetic isotope effect favoring the incorporation of H over D is most likely, as well as other possible exchange pathways that could take place on or off the cobalt. Notably, full conversion of **Co1-a** to **1** was only observed following the addition of two or more equivalents of benzoic acid, where (*R,R*)-(^iPr^DuPhos)­Co­(CO_2_Ph)_2_ ^44^ (**Co1-OBz**) was identified as the sole cobalt product (see Supporting Information). These observations support protonation of the metal center, driven by the formation of a stable, 18-electron cobalt­(II) κ_2_-bis­(carboxylate) product.

Diels–Alder cycloaddition reactions were pursued to further characterize the cyclohexadiene product and aid in the determination of the enantiomeric excess.^45^ Treatment of **1** with maleic anhydride in toluene at 80 °C for 48 h furnished the *endo* product, **2,** in 61% yield (Scheme 11C). X-ray diffraction confirmed the *endo* stereochemistry, and chiral GC analysis established that **2** was nearly racemic, with 5% ee.

To probe the relationship between cobalt cyclohexadienyl diastereomers and the enantiomeric excess of the resulting cyclohexadienes, both **Co1-h** and **Co1-l** were protonated using the same procedure. The use of a longer alkyl chain on the carboxylic acid, such as iso-butyl compared to methyl, was hypothesized to bias the formation of a single diastereomer by restricting the approach to the metal center. The addition of 2.5 equiv of this acid produced a complete reaction within 15–60 min and provided a mixture of constitutional isomers, **3-R** and **4-R** (R = Me or ^i^Bu) in 67% (**3-** ^**i**^ **Bu** + **4-** ^**i**^ **Bu**) and 83% (**3-Me** + **4-Me**) combined yield, along with the corresponding cobalt­(II) bis­(benzoate) complexes (see Supporting Information; Scheme 12A). Following distillation, Diels–Alder trapping with *N*-phenylmaleimide at 78 °C for 36 h provided the corresponding *endo* products **5-R** and **6-R** with <5% exo from products **3-R** and **4-R**, respectively. Diels–Alder products **5-Me** and **6-Me** were formed with 27% ee (**5-Me**) and 18% (**6-Me**), while **5-** ^**i**^ **Bu** and **6-** ^**i**^ **Bu** had similar ee values of 38% (**5-** ^**i**^ **Bu**) and 14% (**6-** ^**i**^ **Bu**). Protonation using other acids, such as diphenylacetic acid, triphenylacetic acid, 1-adamantane carboxylic acid, and 2,4,6-tri*tert*-butylpyridinium triflate, produced similar ee values for **5-R** and **6-R** (see Supporting Information). These data show a correlation between the ratio of diastereomers of the cyclohexadienyl and the enantioenrichment of the free cyclohexadiene. The protonation likely occurs at the metal center, followed by reductive elimination to release the cyclohexadiene (Scheme 12B). Competitive protonation to the deuteration of **1** is also consistent with this observation (Scheme 11B). Because there are two distinct metal faces at which the protonation and subsequent reductive elimination can occur, enantioenrichment is limited to the diastereomeric ratio of insertion products.

**Scheme 12:**
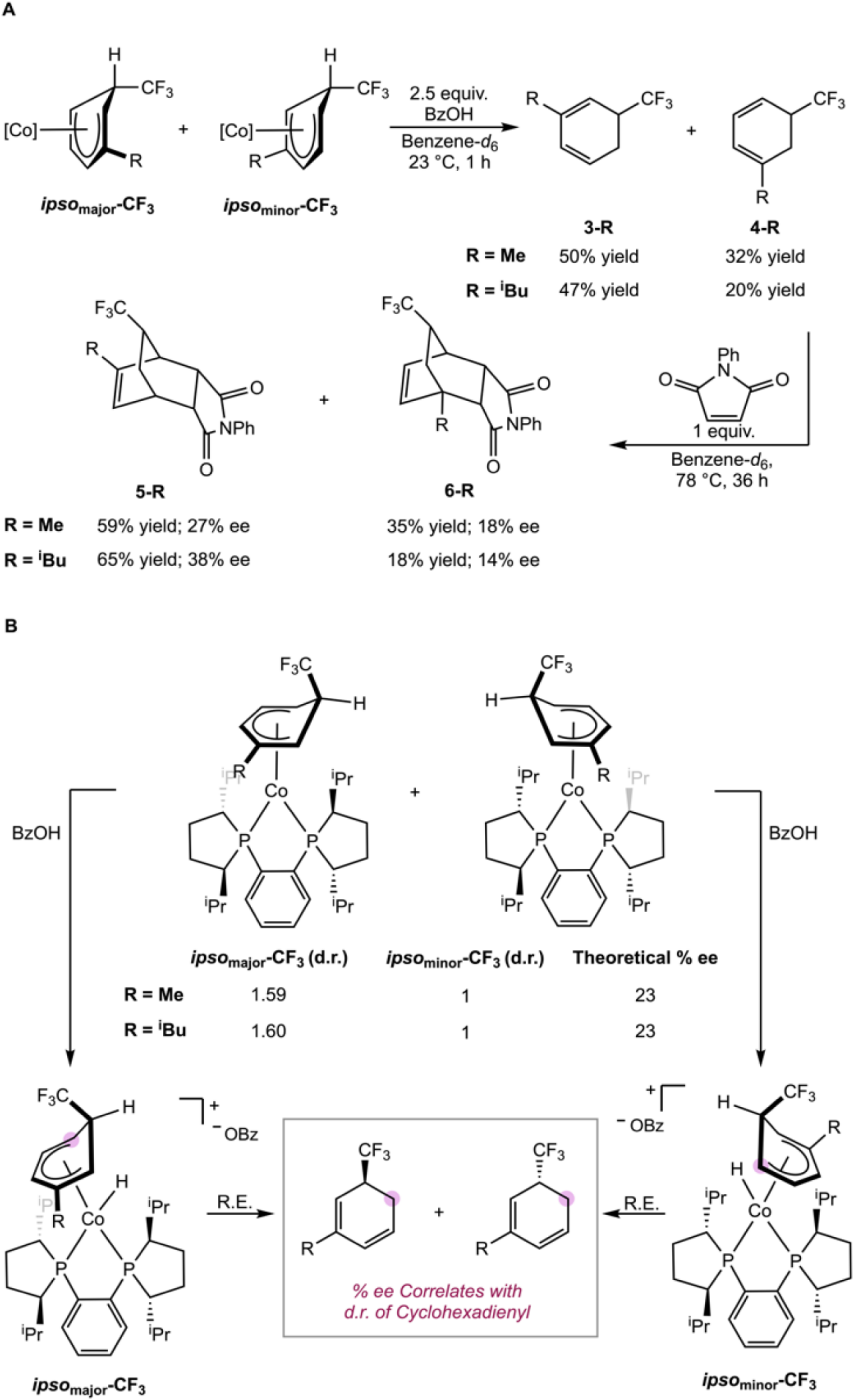
Probing the Relationship between Cyclohexadienyl Diastereomers on Cyclohexadiene Enantioselectivity with **Co1-h** and **Co1-l**. A. Effect of Diastereomers on Cyclohexadiene Synthesis. B. Mechanistic Rationale

## Conclusion

*In situ*-generated cobalt­(I) hydrides supported by bidentate phosphines were studied for the hydrometalation of electronically distinct arenes. The putative cobalt hydride derived from (*R,R*)-^iPr^DuPhos promoted the kinetically aselective insertion of PhCF_3_ that, through reversible β-hydrogen elimination and reinsertion, isomerizes to the thermodynamically preferred *ipso*-CF_3_ isomer. This site selectivity was also observed with disubstituted trifluoromethylbenzenes bearing sterically demanding alkyl, silyl, and boryl substituents, highlighting the electronic effect on the stabilization of the resulting cyclohexadienyl. Sterically encumbered 1,2-disubstituted benzenes reduced *ipso*-CF_3_ selectivity, highlighting the importance of steric interactions between the cyclohexadienyl substituents and the phosphine. Protonation of the cobalt­(I) cyclohexadienyl products with benzoic acid liberated free C­(sp^3^)-CF_3_-substituted 1,3-cyclohexadienes. A correlation between the diastereomeric ratio of the cobalt­(I) cyclohexadienyl and the enantiomeric excess of the resulting cyclohexadienes was observed, supporting protonation at the cobalt center. Through this investigation into the factors governing arene insertion of electron-deficient arenes, a CF_3_-directing effect was established and leveraged toward the chemoselective synthesis of C­(sp^3^)-CF_3_ 1,3-cyclohexadienes. The guiding principles from this study reveal parallels between arene and alkene hydrometalation, informing future transition-metal-mediated, site-selective arene dearomatization and functionalization reactions.

## Supplementary Material



## Data Availability

A preprint of the manuscript was posted on ChemRxiv.^46^

## Notes

Preparation of cobalt complexes; spectroscopic data; general procedure for catalytic transformations; references (PDF)
