# Supplementary material for: Mechanistic Insights into Regioselective Arene Insertion Using Bis(phosphine) Cobalt(I) Hydrides to Form 1,3-Cyclohexadienes
Source: Organometallics. 2026 Apr 16;45(8):978–91. doi: 10.1021/acs.organomet.6c00013 (PMC13126672; doi:10.1021/acs.organomet.6c00013)
Supplement: Supplementary file 1 [file om6c00013_si_001.pdf]

Supporting Information

Mechanistic Insights into Regioselective Arene Insertion using Bis(Phosphine) Cobalt (I) Hydrides to Form 1,3-Cyclohexadienes

Maya J. Lebowitz,[a] Lauren N. Mendelsohn,[a] Hongyu Zhong,[a] Matthew V. Pecoraro,[a] Michael Shevlin,[b] and Paul J. Chirik[a]**

[a]Department of Chemistry, Princeton University, Princeton, New Jersey 08544, United States.
[b]Merck & Co., Inc., Rahway, New Jersey 07065, United States.

pchirik@princeton.edu, michael_shevlin@merck.com

Table of Contents

I. General Considerations S2

II. Preparation and Characterization of Cobalt Allyl Complexes S4

III. Preparation and Characterization of Cobalt Hydride Complexes S7

IV. Preparation and Characterization of Cobalt Cyclohexadienyl Complexes S9

- i. Variable Temperature NMR Spectroscopy S35
- ii. Intramolecular Isomerization Data S38
- iii. Benzene Exchange S51
- iv. Oxidation of **Co1-a** S53

V. NMR Spectroscopic Data of Cobalt Complexes S56

VI. Preparation and Characterization of Cyclohexadienes S206

- i. Preparation and Characterization of Diels-Alder Products S210

VII. Spectroscopic Data of Organic Products S214

VIII. Crystallographic Data for Cobalt and Organic Compounds S266

IX. References S268

I. General Considerations

All air- and moisture-sensitive manipulations were carried out using a vacuum line, Schlenk and cannula techniques, or in an MBraun inert atmosphere (nitrogen) dry box unless otherwise noted. All glassware was stored in a pre-heated oven prior to use. The solvents used for air- and moisture-sensitive manipulations were dried and deoxygenated using literature procedures.[1] Arenes were deoxygenated, stirred over CaH_2 and passed through an alumina plug. Deuterated solvents used in NMR spectroscopic analysis were distilled from sodium metal and stored under an atmosphere of nitrogen. Unless otherwise noted, materials were purchased from commercial vendors and used without further purification.

^{1}H NMR spectra were recorded on either Bruker Avance 400 or 500 spectrophotometers operating at 400 MHz and 500 MHz, respectively. ^{13}C NMR spectra were recorded on either Bruker Avance 400 spectrometers operating at 101 MHz. ^{31}P NMR spectra were recorded on a Bruker Avance 400 spectrometer operating at 161.84 MHz and were referenced to 85% H_3PO_4 as an external standard. ^{19}F NMR spectra were recorded on a Bruker Avance 400 spectrometer operating at 376.5 MHz. All ^{1}H and ^{13}C NMR chemical shifts are reported in ppm relative to $SiMe_4$ using the ^{1}H (cyclohexane-d_{12}: 1.38 ppm; toluene-d_8: 7.09 ppm, 7.00 ppm, 6.98 ppm, 2.09 ppm; chloroform-*d*: 7.26 ppm; benzene-d_6: 7.16 ppm) and ^{13}C (toluene-d_8: 137.86 ppm, 129.24 ppm, 128.33 ppm, 125.49 ppm, 20.4 ppm; benzene-d_6: 128.06 ppm; cyclohexane-d_{12}: 26.43 ppm) chemical shifts of the solvent as a standard. ^{1}H NMR data for diamagnetic compounds are reported as follows: chemical shift, multiplicity (s = singlet, d = doublet, t = triplet, q = quartet, p = pentet, br = broad, m = multiplet, app = apparent, obsc = obscured), coupling constants (Hz), integration, and assignment. ^{1}H NMR data for paramagnetic compounds are reported as follows: chemical shift, integration, peak width at half height (Hz). ^{13}C NMR data for diamagnetic compounds are reported as follows: chemical shift, number of protons attached to carbon (e.g., CH_2), assignment. Magnetic susceptibility measurements in solution were determined by ^{1}H NMR spectroscopy on a Bruker Avance 400 MHz spectrometer using the Evans method.[2] A sealed

capillary containing THF-d_8 was placed in a J. Young NMR tube containing the sample to be measured in THF-d_8.

Elemental analyses were performed at Robertson Microlit Laboratories, Inc., in Ledgewood, NJ. High-resolution mass spectra were obtained at Princeton University mass spectrometry facilities using an Agilent 7200 Accurate-Mass Q-TOF GC/MS. Enantiomeric excesses were determined by Chiral SFC performed by Lotus Separations, LLC or by high-performance liquid chromatography (HPLC) performed on an Agilent 1260 Infinity Series LC equipped with a ChiralPak AD-H (5 μm particle size, 4.6 mm × 250 mm) column. GC analyses were performed using a Shimadzu GC-2010 gas chromatograph equipped with a Shimadzu AOC-20s autosampler and a Supelco 30 m x 0.25 mm BETA DEX 120 capillary column.

Single crystals suitable for X-ray diffraction were coated with polyisobutylene oil in a drybox, transferred to a nylon loop, and then quickly transferred to the goniometer head of a Bruker APEX PHOTON III diffractometer equipped with a Cu X-ray tube (λ = 1.54178 Å). Preliminary data revealed the crystal system. The data collection strategy was optimized for completeness and redundancy using the Bruker COSMO software suite. The space group was identified, and the data were processed using the Bruker SAINT+ program and corrected for absorption using SADABS. The structures were solved using direct methods (SHELXS), completed by subsequent Fourier synthesis and refined by full-matrix least-squares procedures.

II. Preparation of Cobalt Allyl Complexes.

General Procedure A. A 20 mL scintillation vial equipped with a Teflon-coated magnetic stir-bar was charged with 1 equivalent of bis(phosphine)$CoCl_2$ in a 0.04 M Et_2O solution, followed by the addition of 5 equivalents of 1,4-dioxane and cooled for 20 minutes in a –35 °C freezer. In a separate vial, 1.2 equivalents of white, crystalline (1,4-dioxane)Mg(allyl)$_2$[3] was weighed out and dissolved in a 0.2 M Et_2O solution and cooled for 20 minutes in a –35 °C freezer. Once cooled, (1,4-dioxane)Mg(allyl)$_2$ was added dropwise to the ethereal suspension of bis(phosphine)$CoCl_2$ and stirred at room temperature for 4 h. The solution was concentrated, pentane was added, and passed through a pad of Celite, where it was then dried under reduced pressure to yield bis(phosphine)Co(η^3-allyl).

General Procedure B. A 20 mL scintillation vial equipped with a Teflon-coated magnetic stir-bar was charged with 1 equivalent of bis(phosphine)$CoCl_2$ in a 0.04 M Et_2O solution, followed by the addition of 5 equivalents of 1,4-dioxane and cooled for 20 minutes in a –35 °C freezer. In a separate vial, 1.2 equivalents of white, crystalline (1,4-dioxane)Mg(allyl)$_2$ was weighed out and dissolved to form a 0.2 M Et_2O solution and cooled for 20 minutes in a –35° C freezer. Once cooled, (1,4-dioxane)Mg(allyl)$_2$ was added dropwise to the ethereal suspension of bis(phosphine)$CoCl_2$ and stirred at room temperature for 4 h. The solution was concentrated, benzene was added, and passed through a pad of Celite, where it was then dried under reduced pressure to yield bis(phosphine)Co(η^3-allyl).

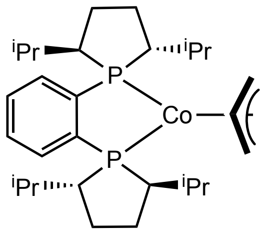

Preparation of Co1. This compound was prepared using General Procedure A described above using 0.72 mmol of (*R,R*)-(^{iPr}DuPhos)$CoCl_2$ and provided 347 mg (93% yield) of (*R,R*)-

(^{iPr}DuPhos)Co(η^3-C_3H_5) as a crystalline, ketyl purple powder identified by ^{1}H, ^{13}C{^{1}H}, and ^{31}P{^{1}H} NMR spectroscopy. Anal Calcd for $C_{29}H_{49}CoP_2$: C, 67.17; H, 9.52. Found: C, 67.31; H, 9.48. ^{1}H NMR (400 MHz, cyclohexane-d_{12}) δ 7.65 – 7.60 (m, 2H, ^{iPr}DuPhos Ar), 7.22 (m, 2H, ^{iPr}DuPhos Ar), 4.43 (tt, J = 13.7, 7.1 Hz, 1H, η^3-C_3H_5), 3.89 (t, J = 7.2 Hz, 1H, η^3-C_3H_5), 3.46 (t, J = 7.0 Hz, 1H, η^3-C_3H_5), 2.45 – 2.26 (m, 4H, overlapping CH + CH_2), 2.18 (m, 4H, overlapping CH + CH_2), 2.02-1.93 (m, 1H, CH), 1.90-1.77 (m, 4H, CH_2), 1.72 (m, 2H, overlapping CH + CH_2), 1.58 (m, 1H, CH), 1.49 (d, J = 13.3 Hz, 1H, η^3-C_3H_5), 1.15 (d, J = 14.4 Hz, 1H, η^3-C_3H_5), 0.96 (d, J = 6.6 Hz, 3H, CH_3), 0.90 (d, J = 6.5 Hz, 3H, CH_3), 0.83 (d, J = 6.5 Hz, 3H, CH_3), 0.76 (d, J = 6.4 Hz, 3H, CH_3), 0.67 (d, J = 6.5 Hz, 3H, CH_3), 0.62 (dd, J = 7.4, 7.0 Hz, 6H, CH_3), 0.52 (d, J = 6.4 Hz, 3H, CH_3). ^{13}C{^{1}H} NMR (101 MHz, cyclohexane-d_{12}) δ 150.63 (m, ^{iPr}DuPhos Ar), 131.27 (t, J = 14.5 Hz, ^{iPr}DuPhos Ar), 128.60 (d, J = 24.3 Hz, ^{iPr}DuPhos Ar), 104.35 (s, η^3-C_3H_5), 56.10 (d, J = 21.1 Hz, CH), 54.75 (app t, η^3-C_3H_5), 53.66 (d, J = 17.7 Hz, CH), 52.88 (d, J = 22.2 Hz, CH), 52.49 (app d, η^3-C_3H_5), 51.94 (d, J = 18.5 Hz, CH), 33.26 (s, CH_2), 32.74 (d, J = 11.7 Hz, CH), 32.53 (s, CH_2), 31.78 (app s, CH), 31.43 (s, CH_2), 31.29 (s, CH_2), 31.03 (d, J = 4.3 Hz, CH), 30.21 (app s, CH), 29.26 (s, CH_2), 26.87 (overlapping d, CH_3), 25.58 (d, J = 4.5 Hz, CH_3), 25.28 (d, J = 4.8 Hz, CH_3), 25.01 (d, J = 6.0 Hz, CH_3), 22.09 (d, J = 6.1 Hz, CH_3), 21.90 (d, J = 7.2 Hz, CH_3), 21.58 (d, J = 6.1 Hz, CH_3), 21.30 (d, J = 8.4 Hz, CH_3). ^{31}P{^{1}H} NMR (162 MHz, cyclohexane-d_{12}) δ 86.69 (app d, 2P).

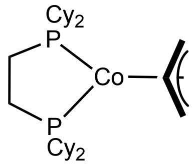

Preparation of Co2. This compound was prepared using General Procedure B described above using 0.27 mmol of (dcype)$CoCl_2$ and provided 105 mg (74% yield) of (dcype)Co(η^3-C_3H_5) as a crystalline, navy-blue powder identified by ^{1}H, ^{13}C{^{1}H} , and ^{31}P{^{1}H} NMR spectroscopy. ^{1}H NMR (400 MHz, cyclohexane-d_{12}) δ 4.24 (dq, J = 13.7, 6.9 Hz, 1H, η^3-C_3H_5), 3.54 (t, J = 6.0 Hz, 2H, η^3-

C_3H_5), 2.00 (dd, *J* = 23.3, 10.9 Hz, 5H, CH + CH_2), 1.89 – 1.63 (m, 25H, CH + CH_2), 1.59 – 1.44 (m, 28H, CH_2), 1.41 – 1.31 (m, 4H, CH_2), 1.29 – 1.00 (m, 8H, CH_2 + η^3-C_3H_5). $^{13}C\{^1H\}$ NMR (101 MHz, Cyclohexane-d_{12}) δ 99.56 (app broad s, η^3-C_3H_5), 51.45 (s, η^3-C_3H_5), 38.53 (m, CH), 37.68 (m, CH), 30.66 (s, CH_2), 30.54 (s, CH_2), 30.36 (s, CH_2), 29.94 (s, CH_2), 28.35 (m, CH_2), 27.51 (s, CH_2), 24.31 – 23.85 (m, CH_2). $^{31}P\{^1H\}$ NMR (162 MHz, cyclohexane-d_{12}) δ 92.86. The data are consistent with a previous report.[4]

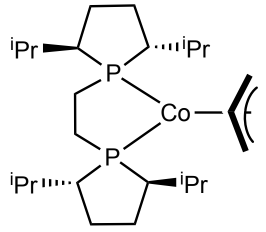

Preparation of Co3. This compound was prepared using General Procedure A described above using 0.16 mmol of (*R,R*)-(^{iPr}BPE)$CoCl_2$ and provided 44 mg (58% yield) of (*R,R*)-(^{iPr}BPE)Co(η^3-C_3H_5) as a crystalline, deep blue powder identified by ^1H, $^{13}C\{^1H\}$, and $^{31}P\{^1H\}$ NMR spectroscopy. ^1H NMR (400 MHz, cyclohexane-d_{12}) δ 4.35 (tt, *J* = 13.7, 7.3 Hz, 1H, η^3-C_3H_5), 3.54 (t, *J* = 6.9 Hz, 1H, η^3-C_3H_5), 3.32 (t, *J* = 7.6 Hz, 1H, η^3-C_3H_5), 2.20 – 2.06 (m, 3H, overlapping CH_2 + CH), 2.06-1.94 (m, 2H, overlapping CH_2 + CH), 1.91-1.74 (m, 3H, overlapping CH_2 + CH), 1.71-1.57 (m, 2H, CH), 1.58-1.43 (m, 6H, overlapping CH_2 + CH), 1.32 (m, 1H, η^3-C_3H_5), 1.14 (m, 1H, η^3-C_3H_5), 1.11 (d, *J* = 13.3 Hz, 3H, CH_3), 0.98 (m, 6H, CH_3), 0.92 (m, 9H, CH_3), 0.83 (dd, *J* = 8.3, 6.6 Hz, 6H, CH_3). $^{13}C\{^1H\}$ NMR (101 MHz, cyclohexane-d_{12}) δ 103.72 (s, η^3-C_3H_5), 53.08 (app m, η^3-C_3H_5), 52.86 (app m, CH), 52.56 (app m, η^3-C_3H_5), 52.33 (d, *J* = 19.4 Hz, CH), 51.33 (d, *J* = 15.7 Hz, CH), 50.81 (d, *J* = 14.7 Hz, CH), 33.01 (d, *J* = 9.5 Hz, CH), 31.91 (m, CH + CH_2), 30.03 (s, CH_2) 29.57 (s, CH_2), 29.38 (s, CH_2), 29.09 (app s, CH), 26.60 (m, overlapping solvent + CH_3), 25.55 (d, *J* = 2.7 Hz, CH_3), 25.32 (d, *J* = 4.5 Hz, CH_3), 25.05 (d, *J* = 7.5 Hz, CH_3), 24.85 (d, *J* = 18.9 Hz, CH_3), 22.80 (dd, *J* = 9.2, 6.5 Hz, CH_3), 20.82 (d, *J* = 6.4 Hz, CH_3), 20.52 (d, *J* = 7.1 Hz, CH_3). $^{31}P\{^1H\}$ NMR (162 MHz, cyclohexane-d_{12}) δ 86.04 (app d, 2P).

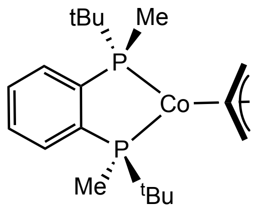

Preparation of Co4. This compound was prepared using General Procedure B described above using 0.21 mmol of (*R,R*)-(BenzP*)$CoCl_2$ and provided 69 mg (82% yield) of (*R,R*)-(BenzP*)Co(η^3-C_3H_5) as a crystalline, red-orange powder identified by ^{1}H, ^{13}C{^{1}H}, and ^{31}P{^{1}H} NMR spectroscopy. The red-orange powder forms a deep purple solution. ^{1}H NMR (400 MHz, cyclohexane-d_{12}) δ 7.64 (m, 2H, BenzP* Ar), 7.28 (m, 2H, BenzP* Ar), 4.41 (tt, *J* = 13.4, 7.1 Hz, 1H, η^3-C_3H_5), 3.88 (dt, *J* = 6.7 Hz, 1H, η^3-C_3H_5), 3.34 (dt, *J* = 7.1, 3.5 Hz, 1H, η^3-C_3H_5), 1.82 (s, 3H, CH_3), 1.63 (s, 3H, CH_3), 1.28 (d, *J* = 7.4 Hz, 2H, η^3-C_3H_5), 1.16 – 0.87 (m, 9H, $C(CH_3)_3$), 0.79 – 0.71 (m, 9H, $C(CH_3)_3$). ^{13}C{^{1}H} NMR (101 MHz, cyclohexane-d_{12}) δ 148.27 (app broad m, BenzP* Ar), 129.79 (app s, BenzP* Ar) , 128.29 (app s, BenzP* Ar), 126.24 (s, BenzP* Ar), 101.02 (s, η^3-C_3H_5), 56.00 (s, η^3-C_3H_5), 50.11 (s, η^3-C_3H_5), 33.44 (s, $C(CH_3)_3$), 33.23 (s, $C(CH_3)_3$), 28.78 (broad s, $(CH_3)_3$), 28.10 (broad s, $(CH_3)_3$), 10.68 (s, CH_3), 9.46 (s, CH_3). ^{31}P NMR (162 MHz, cyclohexane-d_{12}) δ 75.21 (app s, 2P).

III. Preparation of Cobalt Hydride Complexes.

General Procedure A: In a 20 mL scintillation vial, 1 equivalent of cobalt allyl complex was dissolved in a 0.05 M cyclohexane-d_{12} solution and added to a J. Young NMR tube. The contents of the tube were frozen by submerging the tube in liquid nitrogen, followed by the addition of 4 atm of dihydrogen at this temperature. The contents of the tube were thawed carefully and analyzed by ^{1}H NMR spectroscopy to confirm full conversion. For additional analysis, the NMR tube was degassed and brought into a glovebox. The solvent was removed *in vacuo*. Approximately 5 mL of *n*-pentane was used to dissolve the solid which was then passed through

a pad of Celite and dried under reduced pressure to yield a crystalline, powder and analyzed by ^{1}H NMR spectroscopy.

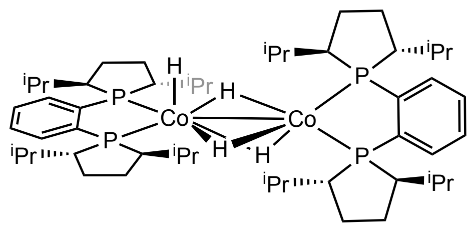

Preparation of Co1-H. This compound was prepared using General Procedure A with 10 mg (0.0194 mmol) of (*R,R*)-(^{iPr}DuPhos)Co(η^3-C_3H_5). The crystalline, deep purple powder obtained in 9 mg (99% yield) was identified as [(*R,R*)-(^{iPr}DuPhos)CoH_2]$_2$, by ^{1}H NMR spectroscopy. ^{1}H NMR (400 MHz, cyclohexane-d_{12}) δ 8.05 – 7.93 (app s, 4H, ^{iPr}DuPhos Ar), 7.28 (app s, 4H, ^{iPr}DuPhos Ar), 3.66 (app s, 4H, Co–H), 1.99 – 1.77 (app s, 4H, CH), 1.78 – 1.68 (m, 8H, overlapping CH_2 + CH), 1.68-1.59 (m, 8H, overlapping CH_2 + CH), 1.56 – 1.40 (m, 5H, overlapping CH_2 + CH), 0.93 - 0.86 (dd, J = 7.7, 7.0 Hz, 6H), 0.79 – 0.74 (app s, 12H, CH_3), 0.69 (d, J = 6.2 Hz, 12H, CH_3), 0.60 (d, J = 6.0 Hz, 12H, CH_3), 0.38 (d, J = 5.0 Hz, 12H, CH_3). No $^{31}P\{^{1}H\}$ NMR signal at 23 °C in cyclohexane-d_{12}. Data are consistent with the published report.[5]

Preparation of Co2-H. This compound was prepared using General Procedure A with 18 mg (0.0343 mmol) of (dcype)Co(η^3-C_3H_5). The crystalline, deep blue powder obtained in 10 mg (61% yield) was identified as [(dcype)CoH_2]$_n$ (n = 2 or 3) by ^{1}H NMR spectroscopy. ^{1}H NMR (400 MHz, cyclohexane-d_{12}) δ 3.38 (app s, 6H, Co–H), 1.95-1.55 (m, 68 H, CH_2 + CH), 1.52-1.05 (m, 94 H, overlapping CH_2), 1.68-1.59 (m, 8H, overlapping CH_2 + CH), 0.95-0.83 (app s, 16H, CH). No $^{31}P\{^{1}H\}$ NMR signal at 23 °C in cyclohexane-d_{12}. Spectrum supports published data.[4,6]

Preparation of Co4-H. This compound was prepared using General Procedure A with 7 mg (0.0182 mmol) of (*R,R*)-(BenzP*)Co(η^3-C_3H_5). The crystalline, deep purple powder obtained in 5

mg (80% yield) was identified as [(*R,R*)-(BenzP*)CoH_2]$_n$ (n = 2 or 3) based on ^1H NMR spectroscopy. Single crystals suitable for X-ray diffraction were obtained from a saturated pentane solution stored at –35 °C overnight, isolated as a trimer, and further supported by Toepler pump experiments revealing a 3:1 ratio of H_2 to cobalt. The ^1H NMR spectrum is consistent with a diamagnetic complex, suggesting a dimeric compound in the solution state and trimeric in the solid state. ^1H NMR (400 MHz, cyclohexane-d_{12}) δ 8.67 (app s, 6H, BenzP* Ar), 7.54 (app s, 6H, BenzP* Ar), 1.37 – 1.26 (m, 18 H, CH_3), 1.07 (app s, 54 H, $C(CH_3)_3$), 0.22 (app s, 6H, Co–H). No $^{31}P\{^1H\}$ NMR signal at 23 °C in cyclohexane-d_{12}.

IV. Preparation and Characterization of Cobalt Cyclohexadienyl Complexes.

General Procedure A. A 20 mL scintillation vial was charged with 1 equivalent of bis(phosphine)Co(η^3-allyl) in a ~0.05 M cyclohexane-d_{12} solution and then added to a J. Young NMR tube, followed by the addition of 10 equivalents of arene and 30 µL of 0.25 M hexamethyldisiloxane (HMDSO) cyclohexane-d_{12} solution. The contents of the tube were frozen by submerging the tube in liquid nitrogen, and 4 atm of dihydrogen was added at this temperature. The contents of the tube were thawed and analyzed by ^1H, $^{13}C\{^1H\}$, ^{19}F, and $^{31}P\{^1H\}$ NMR spectroscopy. Assignments listed at 24 h at 23 °C.

General Procedure B. A 20 mL scintillation vial was charged with 1 equivalent of bis(phosphine)Co(η^3-allyl) in a 0.05 M cyclohexane-d_{12} solution and then added to a J. Young NMR tube, followed by the addition of 20 equivalents of arene and 50 µL of 0.25 M 1,3,5-tris(trifluoromethyl)benzene cyclohexane-d_{12} solution. The contents of the tube were frozen by submerging the tube in liquid nitrogen, and 1 atm of dihydrogen was added at this temperature. The contents of the tube were thawed carefully and analyzed by ^1H, $^{13}C\{^1H\}$, ^{19}F, and $^{31}P\{^1H\}$ NMR spectroscopy. Assignments listed at 24 h at 23 °C.

General Procedure C. A 20 mL scintillation vial was charged with 1 equivalent of bis(phosphine)Co(η^3-allyl) in a 0.2 M arene solution and then added to a 31 mL or 49 mL glass vessel, using ~ 0.5-0.8 mL pentane to ensure solution transfer. The contents of the vessel were frozen by submerging the vessel in liquid nitrogen, and 1- 3 equivalents of dihydrogen were added at this temperature. The contents of the tube were thawed and stirred vigorously for a designated amount of time. The vessel was degassed and brought back into the glovebox. The solvent was removed *in vacuo*. Approximately 5 mL *n*-pentane was added, and the solution evaporated. This dissolution, evaporation procedure was repeated 5 times to ensure complete azeotropic removal of the remaining arene. The remaining solid was dissolved in cyclohexane-d_{12} and transferred to a J. Young NMR tube, followed by the addition of 50 µL of 0.25 M 1,3,5-tris(trifluoromethyl)benzene cyclohexane-d_{12} solution. The contents were further analyzed by ^{1}H, ^{13}C{^{1}H}, ^{19}F, and ^{31}P{^{1}H} NMR spectroscopy.

General Procedure D. A 20 mL scintillation vial was charged with 1 equivalent of bis(phosphine)Co(η^3-allyl) in a ~0.05 M cyclohexane-d_{12} solution and then added to a J. Young NMR tube, followed by the addition of 10 equivalents of arene. A capillary containing 50 µL of 0.15 M hexamethyldisiloxane (HMDSO) and 0.14 M triphenyl phosphine (PPh_3) cyclohexane-d_{12} solution was then added. The contents of the tube were frozen by submerging the tube in liquid nitrogen, and 4 atm of dihydrogen was added at this temperature. The contents of the tube were thawed and analyzed by ^{1}H, ^{13}C{^{1}H}, ^{19}F, and ^{31}P{^{1}H} NMR spectroscopy. Assignments listed at 24 h at 23 °C.

General Procedure E. A 20 mL scintillation vial was charged with 1 equivalent of bis(phosphine)Co(η^3-allyl) in a ~0.05 M cyclohexane-d_{12} solution and then added to a J. Young NMR tube, followed by the addition of 10 equivalents of arene. A capillary containing 50 µL of 0.15 M hexamethyldisiloxane (HMDSO), 0.14 M triphenyl phosphine (PPh_3), 0.2 M 2-

fluorobiphenyl cyclohexane-d_{12} solution was then added. The contents of the tube were frozen by submerging the tube in liquid nitrogen, and 4 atm of dihydrogen was added at this temperature. The contents of the tube were thawed and analyzed by ^{1}H, ^{13}C{^{1}H}, ^{19}F, and ^{31}P{^{1}H} NMR spectroscopy. Assignments listed at 24 h at 23 °C.

General Procedure F. A 20 mL scintillation vial was charged with 1 equivalent of bis(phosphine)Co(η^3-allyl) in a ~0.05 M cyclohexane-d_{12} solution and then added to a J. Young NMR tube, followed by the addition of 10 equivalents of arene. The contents of the tube were frozen by submerging the tube in liquid nitrogen, and 4 atm of dihydrogen was added at this temperature. The contents of the tube were thawed and analyzed by ^{1}H, ^{13}C{^{1}H}, ^{19}F, and ^{31}P{^{1}H} NMR spectroscopy. Assignments listed at 24 h at 23 °C.

1-isopropyl-2-(trifluoromethyl)benzene and 1-isopropyl-3-(trifluoromethyl)benzene were prepared according to literature procedure for 1-(prop-1-en-2-yl)-2-(trifluoromethyl)benzene and 1-(prop-1-en-2-yl)-3-(trifluoromethyl)benzene.[7] Isolated arene was added to a thick-walled vessel with 2 mol% Pd/C (10 w/t %) equipped with a Teflon-coated magnetic stir-bar, followed addition of 4 atm of dihydrogen in neat arene for 12 h at 23 °C. The vessel was depressurized and passed through a pad of silica eluting with hexanes. ^{1}H and ^{19}F NMR spectra are consistent with previously reported values.[8,9]

Facial Assignment of Cobalt Complexes

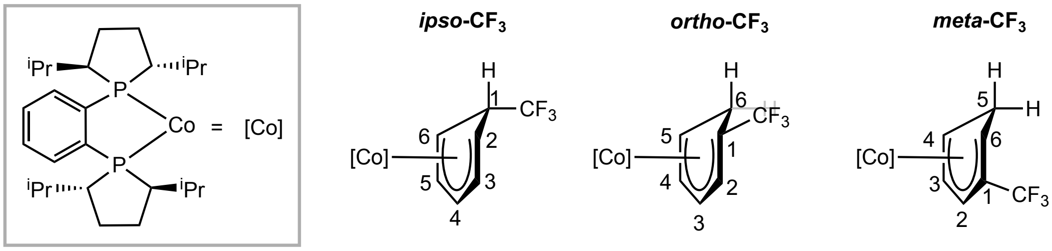

Preparation of Co1-a. This compound was prepared using General Procedure D using 0.0237 mmol of (*R,R*)-(^{iPr}DuPhos)Co(η^3-C_3H_5). The yield of **Co1-a** was 91%, where 84% was *ipso*-CF_3 isomer, 9% *ortho*-CF_3 and *meta*-CF_3 isomers, 6% **Co1-H**, and 3% mass balance lost relative to HMDSO identified by ^{1}H NMR spectroscopy. Using general procedure C with 0.212 mmol **Co1** , **Co1-a** was isolated as a deep red semi-solid in 85% yield (0.180 mmol). Major *ipso*-CF_3 resonances are assigned through ^{1}H, ^{13}C{^{1}H}, ^{19}F, and ^{31}P{^{1}H} NMR spectra. Minor *ortho*-CF_3 and *meta*-CF_3 isomers were assigned by ^{1}H and ^{19}F NMR spectra. ^{1}H NMR (400 MHz, cyclohexane-*d*$_{12}$) δ 7.58 (m, 2H, ^{iPr}DuPhos Ar), 7.23 (m, 2H, ^{iPr}DuPhos Ar), 5.71 (t, *J* = 5.8 Hz, 1H, C^3H_{meta}), 5.42 (d, *J* = 5.4 Hz, 1H, C^2H_{ortho}), 5.29 (t, *J* = 5.9 Hz, 1H, $C^{3/5}H_{ipso}$), 5.13 (app s, 1H, C^4H_{ortho}), 5.12 (app s, 1H, C^4H_{ipso}), 5.04 (app s, 1H, C^4H_{meta}), 4.94 (app s, 1H, C^3H_{ortho}), 4.67 (t, *J* = 6.0 Hz, 1H, $C^{3/5}H_{ipso}$), 4.52 (d, *J* = 3.9 Hz, C^2H_{meta}), 3.61 (t, *J* = 5.4 Hz, 1H, $C^{2/6}H_{ipso}$), 3.45 (app s, 1H, C^5H_{ortho}), 3.07 (dtt, *J* = 8.4, 5.8, 2.4 Hz, 1H, C^1H_{ipso}), 2.84 (t, *J* = 6.3 Hz, 1H, $C^{2/6}H_{ipso}$), 2.74 (m, 1H, C^6H_{meta}), 2.62 (m, 1H, C^6H_{ortho}), 2.45 (m, 1H, C^5H_{meta}), 2.36 (m, 1H, C^6H_{ortho}), 2.34 (m, 1H, C^5H_{meta}), 2.32-2.17 (m, 4H, overlapping CH + CH_2), 2.16– 2.04 (m, 3H, CH_2), 2.03-1.90 (m, 2H, overlapping CH + CH_2), 1.73-1.46 (m, 7H, overlapping CH + CH_2), 1.00 (d, *J* = 6.7 Hz, 6H, CH_3), 0.75 (d, *J* = 6.6 Hz, 6H, CH_3), 0.65 (d, *J* = 6.6 Hz, 6H, CH_3), 0.58 (d, *J* = 6.7 Hz, 6H, CH_3). ^{13}C{^{1}H} NMR (101 MHz, cyclohexane-*d*$_{12}$) δ 149.63 (app s, ^{iPr}DuPhos Ar), 131.14 (d, *J* = 14.6 Hz, ^{iPr}DuPhos Ar), 128.32 (d, *J* = 4.8 Hz, ^{iPr}DuPhos Ar), 121.57 (app broad m, $CF_{3;ipso}$), 91.82 (s, $C^{3/5}_{ipso}$), 90.46 (s, $C^{3/5}_{ipso}$), 76.60 (s, $C^4{}_{ipso}$), 56.60 (d, *J* = 18.8 Hz, CH), 53.10 (d, *J* = 18.9 Hz, CH), 46.65 (s, $C^{2/6}_{ipso}$), 45.17 (s, $C^{2/6}_{ipso}$), 43.53 (q, *J* = 27.4 Hz, $C^1{}_{ipso}$), 32.10 (app d, CH_2), 31.53 (m, overlapping CH + CH_2),

28.89 (app s, CH), 25.32 (d, *J* = 4.7 Hz, CH_3), 25.17 (d, *J* = 6.4 Hz, CH_3), 22.24 (d, *J* = 8.4 Hz, CH_3), 21.23 (d, *J* = 4.2 Hz, CH_3). $^{31}P\{^1H\}$ NMR (162 MHz, cyclohexane-d_{12}) δ 90.16 (broad s, 2P, *ipso*-CF_3). ^{19}F NMR (376 MHz, cyclohexane-d_{12}) δ -62.82 (s, *ortho*-CF_3), -64.18 (s, *meta*-CF_3), -80.60 (d, *J* = 8.3 Hz, *ipso*-CF_3).

Facial Assignment of Cobalt Complexes

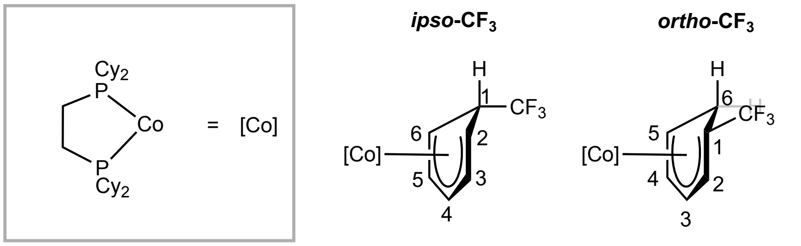

Preparation of Co2-a. This compound was prepared using General Procedure C using 0.030 mmol of (dcype)Co(η^3-C_3H_5). The deep red solid was identified as 90% yield of (dcype)Co(η^5-$C_6H_6CF_3$), where 80% yield of *ipso*-CF_3 isomer and 20% yield of *ortho*-CF_3 was assigned by ^1H and ^{19}F NMR spectroscopy in benzene-d_6. The complex was assigned separately following general procedure B without an internal standard using 0.019 mmol of (dcype)Co(η^3-C_3H_5). ^1H NMR (400 MHz, cyclohexane-d_{12}) δ 5.23 (d, *J* = 5.6 Hz, 1H, C^3H_{ortho}), 5.13 (t, *J* = 5.7 Hz, 1H, C^4H_{ortho}), 5.06 (d, *J* = 5.0 Hz, 1H, C^4H_{ipso}), 4.99 (t, *J* = 5.7 Hz, 2H, $C^{3/5}H_{ipso}$), 4.94 (d, *J* = 5.4 Hz, 1H, $^2H_{ortho}$), 3.14 (t, *J* = 6.0 Hz, 2H, $C^{2/6}H_{ipso}$), 3.01 (q, *J* = 7.3 Hz, 1H, C^1H_{ipso}), 2.83 (app s, 1H, C^5H_{ortho}), 2.67 – 2.54 (m, 1H, C^6H_{ortho}), 2.27 (m, 1H, C^6H_{ortho}), 1.92 – 1.55 (m, 44H, CH_2 + CH), 1.32 – 1.17 (m, 36H, CH_2 + CH), 1.10 – 0.98 (m, 10H, CH_2). $^{13}C\{^1H\}$ NMR (101 MHz, cyclohexane-d_{12}) δ 90.41 (s, C^4_{ortho}), 90.23 (s, $C^{3/5}_{ipso}$), 87.02 (app s, C^2_{ortho}), 76.64 (s, C^4_{ipso}), 76.50 (s, C^3_{ortho}), 43.82 (q, *J* = 28.8 Hz, C^1_{ipso}), 42.73 (s, $C^{2/6}_{ipso}$), 42.16 (s, C^5_{ortho}), 39.58 (d, *J* = 17.3 Hz, CH), 29.74 (s, CH_2), 28.38 (d, *J* = 10.9 Hz, CH_2), 28.15 (s, *J* = 10.0 Hz, CH_2), 27.59 (s, CH_2),. C^6_{ortho}, C^1_{ortho}, and CF_3 peaks could not be accurately assigned due to poor signal resolution and low abundance in solution. $^{31}P\{^1H\}$ NMR (162 MHz, cyclohexane-d_{12}) δ 96.78 (app broad s, 4P, *ipso*-CF_3 + *ortho*-

CF_3). ^{19}F NMR (376 MHz, cyclohexane-d_{12}) δ -63.38 (s, 3F, *ortho*-CF_3), -80.46 (d, J = 8.4 Hz, 3F, *ipso*-CF_3).

Facial Assignment of Cobalt Complexes

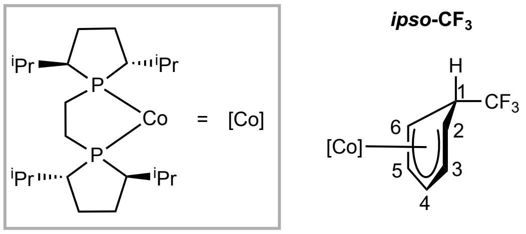

Preparation of Co3-a. This compound was prepared using General Procedure B using 0.0085 mmol of (*R,R*)-(^{iPr}BPE)Co(η^3-C_3H_5). The deep red solution formed was identified as 90% yield of (*R,R*)-(^{iPr}BPE)Co(η^5-$C_6H_6CF_3$), where *ipso*-CF_3 isomer accounted for 89% relative to 1,3,5-tris(trifluoromethyl)benzene assigned through ^{1}H, ^{13}C{^{1}H}, ^{19}F, and ^{31}P{^{1}H} NMR spectra. ^{1}H NMR (400 MHz, cyclohexane-d_{12}) δ 5.10 (t, J = 6.0 Hz, 1H, $C^{3/5}$H), 4.90 (app s, 1H, C^4H), 4.67 (t, J = 5.9 Hz, 1H, $C^{3/5}$H), 3.41 (t, J = 6.3 Hz, 1H, $C^{2/6}$H), 2.95 – 2.89 (m, 1H, C^1H), 2.84 (app s, 1H, $C^{2/6}$H), 2.07 – 2.00 (m, 4H, CH_2), 1.96-1.86 (m, 4H, CH_2), 1.83-1.76 (m, 2H, CH), 1.71-1.61 (m, 2H, CH), 1.60-1.50 (m, 2H, CH_2), 1.38 (m, 4H, overlapping solvent + CH_2 + CH), 1.29 (m, 2H, overlapping solvent + CH_2), 1.14 (app s, 2H, CH), 1.07 (d, J = 6.6 Hz, 12H, CH_3), 0.89 (m, 16H, CH_3). ^{13}C{^{1}H} NMR (101 MHz, cyclohexane-d_{12}) δ 121.46 (app broad m, CF_3), 91.31 (s, $C^{3/5}$), 90.38 (s, $C^{3/5}$), 76.88 (s, C^4), 53.30 (d, J = 14.5 Hz, CH), 52.28 (d, J = 18.7 Hz, CH), 45.13 (s, $C^{2/6}$), 44.17 (s, $C^{2/6}$), 43.56 (q, J = 28.6 Hz, C^1), 31.56 (d, J = 8.7 Hz, CH), 30.97 (s, CH_2), 29.62 (s, CH_2), 28.15 (s, CH_2), 25.00 (dd, J = 19.2, 6.3 Hz, CH_3), 22.57 – 20.97 (m, CH_3). ^{31}P{^{1}H} NMR (162 MHz, cyclohexane-d_{12}) δ 94.53 (s, 2P). ^{19}F NMR (376 MHz, cyclohexane-d_{12}) δ -80.57 (d, J = 8.3 Hz, 3F).

Facial Assignment of Cobalt Complexes

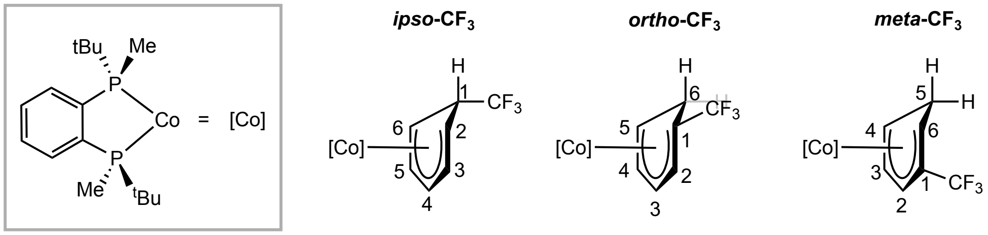

Preparation of Co4-a. This compound was prepared using General Procedure A using 0.018 mmol of (*R,R*)-(BenzP*)Co(η^3-C_3H_5). A deep red solution formed and was identified as of (*R,R*)-(BenzP*)Co(η^5-$C_6H_6CF_3$) in 88% yield at early time points (between 30 min – 1h). At 24 h, **Co4-a** resulted in 17% mass balance lost with 48% yield *ipso*-CF_3 isomer, 37% *ortho*-CF_3 and 15% *meta*-CF_3 isomers relative to HMDSO assigned by ^{1}H, ^{13}C{^{1}H}, ^{19}F, and ^{31}P{^{1}H} NMR spectroscopy. ^{1}H NMR (400 MHz, cyclohexane-d_{12}) δ 7.73 (t, *J* = 6.4 Hz, 2H, BenzP* Ar), 7.57-7.50 (m, 6H, overlapping solvent + BenzP* Ar), 7.29 – 7.24 (m, 4H, overlapping solvent + BenzP* Ar), 5.63 (t, *J* = 5.4 Hz, 1H, C^3H_{meta}), 5.42 (t, *J* = 4.0 Hz, 1H, C^2H_{ortho}), 5.20 (d, *J* = 5.6 Hz, 1H, C^4H_{meta}), 5.16 (t, *J* = 5.8 Hz, 1H, $C^{3/5}H_{ipso}$), 5.08 (d, *J* = 6.0 Hz, 1H, C^4H_{ipso}), 5.03 (dd, *J* = 7.4, 3.8 Hz, 1H, C^4H_{ortho}), 4.98 (t, *J* = 6.1 Hz, 1H, $C^{3/5}H_{ipso}$), 4.93 (d, *J* = 5.4 Hz, 1H, C^3H_{ortho}), 4.56 (t, *J* = 5.8 Hz, 1H, C^2H_{meta}), 3.47 (app s, 1H, C^5H_{ortho}), 3.29 (t, *J* = 5.5 Hz, 1H, $C^{6/2}H_{ipso}$), 3.08 (m, 2H, $C^{6/2}H_{ipso}$ + C^1H_{ipso}), 2.76 (app d, 1H, C^5H_{meta}), 2.66 (m, 1H, C^6H_{ortho}), 2.28 (m, 2H, C^6H_{ortho} + C^5H_{meta}), 2.21 (m, 1H, C^6H_{meta}), 1.91 (dd, *J* = 12.6, 7.4 Hz, 6H, CH_3), 1.65 (app broad m, 6H, CH_3), 1.36 (m, 6H, CH_3 + overlapping solvent), 1.15 (d, *J* = 13.1 Hz, 18H, $C(CH_3)_3$), 0.86 (m, 18H, $C(CH_3)_3$ + overlapping solvent), 0.71 (d, *J* = 12.9 Hz, 18H, $C(CH_3)_3$). ^{13}C{^{1}H} NMR (126 MHz, cyclohexane-d_{12}) δ 147.75 (dd, *J* = 45.1, 39.4 Hz, BenzP* Ar), 146.67 (app m, BenzP* Ar), 145.49 (app t, BenzP* Ar), 129.98 (app broad m , $CF_{3;\ ortho}$), 129.56 (d, *J* = 3.8 Hz, BenzP* Ar), 128.70 (d, *J* = 4.9 Hz, BenzP* Ar), 128.56 (d, *J* = 3.2 Hz, Benzp* Ar), 128.34 (app d, BenzP* Ar), 125.69 (overlapping s, BenzP* Ar), 122.40 (app broad m , $CF_{3;\ ipso}$), 92.09 (s, C^4_{ortho}), 91.16 (s, $C^{3/5}_{ipso}$), 90.32 (s, $C^{3/5}_{ipso}$), 88.34 (s, C^4_{meta}), 87.71 (app br s, C^2_{ortho}), 85.13 (app br s, C^2_{meta}), 84.21 (app s, C^3_{meta}), 76.78 (app d, C^4_{ipso}), 74.37 (app

d, C^3_{ortho}), 58.60 (dd, J = 7.3, 4.0 Hz, C^5_{ortho}), 49.00 (s, $C^{6/2}_{ipso}$), 46.47 (app broad m, C^1_{ortho}), 43.47 (q, J = 28.3 Hz, C^1_{ipso}), 42.79 (s, $C^{2/6}_{ipso}$), 35.86 (d, J = 17.4 Hz, $C(CH_3)_3$), 35.46 (d, J = 18.6 Hz, $C(CH_3)_3$), 34.61 (d, J = 20.3 Hz, $C(CH_3)_3$), 34.25 (app broad m, C^6_{meta}), 29.21 (app broad s, C^5_{meta} + C^6_{ortho}), 28.27 (m, $(CH_3)_3$), 27.95 (m, $(CH_3)_3$), 27.52 (m, $C(CH_3)_3$), 12.22 (d, J = 19.5 Hz, CH_3), 10.48 (d, J = 9.3 Hz, CH_3), 10.24 (d, J = 13.3 Hz, CH_3). C^1_{meta} and $CF_{3;meta}$ peaks could not be accurately assigned due to overlapping signals with the starting material and/or low abundance in solution. $^{31}P\{^1H\}$ NMR (162 MHz, cyclohexane-d_{12}) δ 84.65 (app br s, 2P), 80.32 (app br s, 1P), 68.88 (app br s, 1P), 67.28 (app br s, 2P). Absolute $^{31}P\{^1H\}$ NMR resonance assignments could not be made due to overlapping signals. ^{19}F NMR (376 MHz, cyclohexane-d_{12}) δ -62.42 (s, 3F, *meta*-CF_3), -64.33 (app t, 3F, *ortho*-CF_3), -75.37 (d, J = 7.9 Hz, (trifluoromethyl)cyclohexane, generated from unidentified [Co] side-product following arene insertion), -80.39 (d, J = 7.9 Hz, 3F, *ipso*-CF_3).

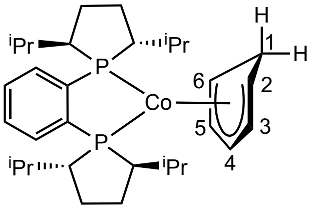

Preparation of Co1-b. This compound was prepared using General Procedure B using 0.0085 mmol of (*R,R*)-(^{iPr}DuPhos)Co(η^3-C_3H_5). A deep red solution formed was identified as (*R,R*)-(^{iPr}DuPhos)Co(η^5-C_6H_7) in 96% as determined relative to 1,3,5-tris(trifluoromethyl)benzene, assigned using ^1H and $^{13}C\{^1H\}$ NMR spectroscopy. ^1H NMR (400 MHz, cyclohexane-d_{12}) δ 7.56 (app s, 2H, ^{iPr}DuPhos Ar), 7.20 (overlapping app s, 2H, ^{iPr}DuPhos Ar), 5.26 (app s, 1H, C^4H), 5.17 (d, J = 6.2, 4.9 Hz, 1H, $C^{3/5}H$), 4.53 (dd, J = 5.7, 6.5 Hz, 1H, $C^{3/5}H$), 3.58 (app t, J = 5.7 Hz, 1H, $C^{2/6}H$), 2.69 (app t, J = 5.5 Hz, 1H, $C^{2/6}H$), 2.60 (m, 1H, C^1H), 2.37 – 2.29 (m, 1H, C^1H), 2.28-2.16 (m, 5H, overlapping CH + CH_2), 2.17-1.89 (m, 5H, overlapping CH + CH_2), 1.70-1.51 (m, 6H, CH_2), 1.00 (d, J = 6.5 Hz, 6H, CH_3), 0.73 (d, J = 6.4 Hz, 6H, CH_3), 0.63 (d, J = 6.7 Hz, 6H, CH_3), 0.55 (d, J = 6.7 Hz, 6H, CH_3). $^{13}C\{^1H\}$ NMR (101 MHz, cyclohexane-d_{12}) δ 150.69 (dd, J = 41.2,

27.1 Hz, ^{iPr}DuPhos Ar), 131.04 (d, *J* = 14.4 Hz, ^{iPr}DuPhos Ar), 128.26 (d, *J* = 3.5 Hz, ^{iPr}DuPhos Ar), 91.86 (s, $C^{3/5}$), 90.79 (s, $C^{3/5}$), 76.06 (s, C^4), 56.36 (d, *J* = 17.2 Hz, CH), 52.72 (app t, $C^{2/6}$), 52.54 (app t, CH), 50.31 (s, $C^{2/6}$), 32.06 (app d, CH_2), 31.47 (m, overlapping CH + CH_2), 31.20 (s, C^1), 28.92 (app broad s, CH), 25.30 (d, *J* = 3.9 Hz, CH_3), 25.20 (d, *J* = 5.0 Hz, CH_3), 22.33 (d, *J* = 8.6 Hz, CH_3), 21.30 (d, *J* = 4.6 Hz, CH_3). No observable $^{31}P\{^1H\}$ NMR signal at 23 °C in cyclohexane-d_{12}.

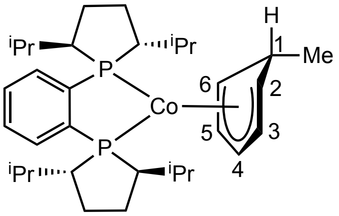

Preparation of Co1-c. This compound was prepared using General Procedure F using 0.0154 mmol of (*R,R*)-(^{iPr}DuPhos)Co(η^3-C_3H_5). A deep red solution formed where 55% of (*R,R*)-(^{iPr}DuPhos)Co(η^5-C_6H_6Me) was observed as the *ipso*-Me isomer relative to 3 (*ortho*, *meta*, *para*) isomers, and assigned using ^{1}H and $^{13}C\{^1H\}$ NMR spectroscopy. *Ipso*-Me isomer is the only isomer assigned. ^{1}H NMR (400 MHz, cyclohexane-d_{12}) δ 7.55 (app s, 2H, ^{iPr}DuPhos Ar), 7.14 (overlapping app s, 2H, ^{iPr}DuPhos Ar), 5.08 (app d, 2H, $C^{3/5}$H + C^4H), 4.45 (app s, 1H, $C^{3/5}$H), 3.85 (t, *J* = 6.1 Hz, 1H, $C^{2/6}$H), 3.06 (m, 1H, $C^{2/6}$H), 2.44 (m, 1H, C^1H), 2.40 – 2.32 (m, 2H, CH_2), 2.24-2.11 (m, 4H, overlapping CH + CH_2), 2.09- 1.93 (m, 4H, overlapping CH + CH_2), 1.72-1.55 (m, 6H, overlapping CH + CH_2), 0.99 (d, *J* = 6.1 Hz, 6H, CH_3), 0.74 (d, *J* = 7.0 Hz, 6H, CH_3), 0.65 (d, *J* = 6.6 Hz, 6H, CH_3), 0.56 (d, *J* = 6.1 Hz, 6H, CH_3), 0.42 (d, *J* = 6.1 Hz, 3H, $CH_{3;\,ipso}$). $^{13}C\{^1H\}$ NMR (101 MHz, cyclohexane-d_{12}) δ 130.92 (app d, ^{iPr}DuPhos Ar), 128.21 (app d, ^{iPr}DuPhos Ar), 89.27 (s, $C^{3/5}$), 88.40 (s, $C^{3/5}$), 75.81 (s, C^4), 60.61 (s, $C^{2/6}$), 59.07 (s, $C^{2/6}$), 56.46 (d, *J* = 18.1 Hz, CH), 52.86 (d, *J* = 18.1 Hz, CH), 35.87 (s, C^1), 32.34 (d, *J* = 6.2 Hz, CH), 32.08 (d, *J* = 6.2 Hz, CH), 31.45 (app m, CH + CH_2), 30.62 (s, CH_2), 28.85 (app broad s, CH), 25.31- 25.18 (app m, CH_3), 22.35 (app m, CH_3), 21.30 (d, *J* = 4.1 Hz, CH_3). Additional ^{iPr}DuPhos Ar and $CH_{3;ipso}$ peaks

could not be assigned due to peak broadening, poor HMBC resolution, and overlapping resonances. An intractable $^{31}P\{^1H\}$ NMR spectrum was observed at 23 °C in cyclohexane-d_{12}.

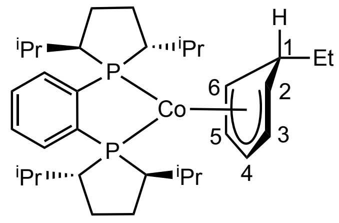

Preparation of Co1-d. This compound was prepared using General Procedure E using 0.0190 mmol of (*R,R*)-(^{iPr}DuPhos)Co(η^3-C_3H_5). A deep red solution formed in 95% total yield that contained 72% yield of (*R,R*)-(^{iPr}DuPhos)Co(η^5-C_6H_6Et) and 6% yield of **Co1-H** relative to HMDSO was determined. The major *ipso*-Et isomer was assigned using ^{1}H and $^{13}C\{^1H\}$ NMR spectroscopy. ^{1}H NMR (400 MHz, cyclohexane-d_{12}) δ 7.54 (dd, *J* = 6.8, 3.3 Hz, 2H, ^{iPr}DuPhos Ar), 7.14 (app m, 2H, overlapping solvent + ^{iPr}DuPhos Ar), 5.11 (t, *J* = 5.8 Hz, 1H, $C^{3/5}$H), 5.07 (m, 1H, C^4H), 4.48 (t, *J* = 6.0 Hz, 1H, $C^{3/5}$H), 3.92 – 3.83 (m, 1H, $C^{2/6}$H), 3.08 (t, *J* = 6.3 Hz, 1H, $C^{2/6}$H), 2.44 – 2.24 (m, 2H, overlapping CH_2+ CH), 2.24- 2.16 (m, 4H, overlapping CH_2 + CH + C^1H), 2.14 – 1.93 (m, 4H, overlapping CH_2 + CH), 1.90 – 1.64 (m, 2H, overlapping CH_2 + CH), 1.60 – 1.42 (m, 4H, overlapping CH_2 + CH), 0.99 (d, *J* = 6.6 Hz, 6H, CH_3), 0.74 (d, *J* = 6.4 Hz, 8H, CH_3 + $CH_{2;ipso}$), 0.67 – 0.63 (d, *J* = 6.6 Hz, 9H, CH_3 + $CH_{3;ipso}$), 0.57 (d, *J* = 6.7 Hz, 6H, CH_3). Additional aryl resonances are associated with PPh_3 and 2-fluoro-biphenyl in a cyclohexane-d_{12} capillary. ^{13}C NMR (101 MHz, cyclohexane-d_{12}) δ 150.71 (dd, *J* = 40.9, 27.4 Hz, ^{iPr}DuPhos Ar), 130.90 (app s, ^{iPr}DuPhos Ar), 128.10 (s, ^{iPr}DuPhos Ar), 89.58 (s, $C^{3/5}$), 88.70 (s, $C^{3/5}$), 76.09 (s, C^4), 58.64 (s, $C^{2/6}$), 57.27 (s, $C^{2/6}$), 56.29 (d, *J* = 17.8 Hz, CH), 52.76 (d, *J* = 17.5 Hz, CH), 42.73 (s, C^1), 35.65 (app t, $CH_{2;ipso}$), 32.06 (d, *J* = 4.8 Hz, CH), 31.56 (s, CH_2), 31.36 (d, *J* = 10.7 Hz, CH), 28.88 (s, CH_2), 25.30 (d, *J* = 5.0 Hz, CH_3), 25.18 (d, *J* = 6.5 Hz, CH_3), 22.33 (d, *J* = 8.4 Hz, CH_3), 21.33 (d, *J* = 4.4 Hz, CH_3), 9.74 (s, $CH_{3;ipso}$). No $^{31}P\{^1H\}$ NMR signal was observed at 23 °C in cyclohexane-d_{12}, resonance at - 5.70 ppm associated with PPh_3 in a cyclohexane-d_{12} capillary.

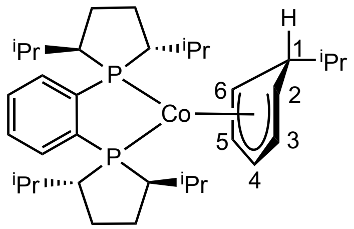

Preparation of Co1-e. This compound was prepared using General Procedure F using 0.0193 mmol of (*R,R*)-(^{iPr}DuPhos)Co(η^3-C_3H_5). A deep red solution formed was identified that contained 91% yield of (*R,R*)-(^{iPr}DuPhos)Co(η^5-$C_6H_6{}^{i}$Pr) and 6% yield of **Co1-H** relative to 3 (*ortho*, *meta*, *para*) isomers. Major *ipso*-^{i}Pr assigned using ^{1}H, ^{13}C{^{1}H}, and ^{31}P{^{1}H} NMR spectroscopy. Using General Procedure B using 0.0187 mmol of **Co1**, the yield of **Co1-e** was 86% yield and <1% **Co1-H** relative to 1,3,5-tris(trifluoromethyl)benzene assigned using ^{1}H NMR spectroscopy. ^{1}H NMR (400 MHz, cyclohexane-d_{12}) δ 7.54 (m, 2H, ^{iPr}DuPhos Ar), 7.14 (overlapping app s, 2H, ^{iPr}DuPhos Ar), 5.13 (t, J = 5.8 Hz, 1H, $C^{3/5}$H), 5.04 (app s, 1H, C^4H), 4.50 (t, J = 6.0 Hz, 1H, $C^{3/5}$H), 3.91 (t, J = 6.4 Hz, 1H, $C^{2/6}$H), 3.11 (t, J = 6.3 Hz, 1H, $C^{2/6}$H), 2.30-2.23 (m, 2H, CH_2), 2.23-2.16 (m, 3H, overlapping CH + CH_2), 2.15– 2.03 (m, 5H, overlapping CH + CH_2), 2.03-1.94 (m, 2H, CH), 1.90 (m, 1H, C^1H), 1.75-1.50 (m, 4H, overlapping solvent + CH_2), 1.00 (d, J = 6.5 Hz, 6H, CH_3), 0.85 (m, 1H, CH_{ipso}), 0.63 (m, 6H, $CH_{3;ipso}$ + overlapping ligand), 0.64 (m, 12H, CH_3), 0.58 (d, J = 6.7 Hz, 6H, CH_3). ^{13}C{^{1}H} NMR (101 MHz, cyclohexane-d_{12}) δ 150.69 (dd, J = 41.1, 27.3 Hz, ^{iPr}DuPhos Ar), 130.94 (d, J = 15.7 Hz, ^{iPr}DuPhos Ar), 128.27 (m, ^{iPr}DuPhos Ar), 89.68 (s, $C^{3/5}$), 88.82 (s, $C^{3/5}$), 76.45 (s, C^4), 57.63 (s, $C^{2/6}$), 56.47 (s, $C^{2/6}$), 56.30 (d, J = 16.4 Hz, CH), 52.87 (d, J = 18.5 Hz, CH), 48.17 (s, C^1), 39.21 (app t, CH_{ipso}), 32.00 (d, J = 4.8 Hz, CH), 31.62 (s, CH_2), 31.30 (d, J = 10.9 Hz, CH), 28.95 (s, CH_2), 25.32 (d, J = 5.7 Hz, CH_3), 25.15 (d, J = 6.4 Hz, CH_3), 22.30 (d, J = 8.3 Hz, CH_3), 21.37 (d, J = 4.5 Hz, CH_3), 18.54 (app d, $CH_{3;ipso}$). ^{31}P{^{1}H} NMR (162 MHz, cyclohexane-d_{12}) δ 89.28 (broad s, 2P).

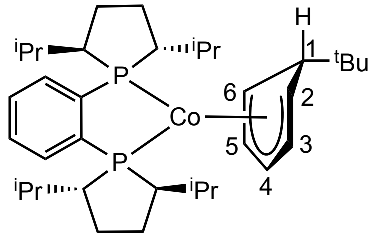

Preparation of Co1-f. This compound was prepared using General Procedure D using 0.0193 mmol of (*R,R*)-(^{iPr}DuPhos)Co(η^3-C_3H_5). A deep red solution formed that contained 90% total yield, where >99% yield of the *ipso* isomer of (*R,R*)-(^{iPr}DuPhos)Co(η^5-$C_6H_6{}^tBu$) as the exclusive isomer and 10% **Co1-H** relative to an HMDSO internal standard assigned by ^{1}H and ^{13}C{^{1}H} NMR spectra. Using General Procedure B, the yield of **Co1-f** was 84% yield, 7% **Co1-H**, and 9% mass balance lost relative to 1,3,5-tris(trifluoromethyl)benzene assigned by ^{1}H, ^{13}C{^{1}H}, and ^{31}P{^{1}H} NMR spectroscopy. ^{1}H NMR (400 MHz, cyclohexane-d_{12}) δ 7.54 (m, 2H, ^{iPr}DuPhos Ar), 7.14 (overlapping app s, 2H, ^{iPr}DuPhos Ar), 5.24 (t, *J* = 5.9 Hz, 1H, $C^{3/5}$H), 4.94 (app s, 1H, C^4H), 4.63 (t, *J* = 6.0 Hz, 1H, $C^{3/5}$H), 3.88 (t, *J* = 6.3 Hz, 1H, $C^{2/6}$H), 3.12 (t, *J* = 6.0 Hz, 1H, $C^{2/6}$H), 2.34 (m, 1H, C^1H), 2.32-2.24 (m, 1H, CH_2), 2.24- 2.18 (m, 2H, overlapping CH + CH_2), 2.16– 2.04 (m, 2H, CH_2), 1.97 (broad s, 1H, CH), 1.81-1.72 (m, 2H, overlapping CH + CH_2), 1.71-1.55 (m, 8H, overlapping CH + CH_2), 0.98 (d, *J* = 6.6 Hz, 6H, CH_3), 0.75 (d, J = 6.6 Hz, 6H, CH_3), 0.66 (d, *J* = 6.5 Hz, 6H, CH_3), 0.62 (s, 9H, $(CH_3)_{3;ipso}$), 0.57 (d, *J* = 6.5 Hz, 6H, CH_3).^{13}C{^{1}H} NMR (101 MHz, cyclohexane-d_{12}) δ 149.82 (dd, *J* = 41.2, 27.5 Hz, ^{iPr}DuPhos Ar), 130.98 (app s, ^{iPr}DuPhos Ar), 128.26 (d, *J* = 4.8 Hz, ^{iPr}DuPhos Ar), 91.46 (s, $C^{3/5}$), 90.95 (s, $C^{3/5}$), 74.69 (s, C^4), 58.29 (s, $C^{2/6}$), 57.30 (s, $C^{2/6}$), 56.30 (d, *J* = 17.6 Hz, CH), 52.72 (d, *J* = 17.3 Hz, CH), 51.31 (s, C^1), 37.40 (app t, $C(CH_3)_{3;ipso}$), 32.18 (app d, CH_2), 31.57 (app broad s, CH_2), 31.30 (d, *J* = 10.0 Hz, CH), 28.85 (app broad s, CH), 25.02 (s, $(CH_3)_{3;ipso}$), 25.33 (d, *J* = 5.1 Hz, CH_3), 25.22 (d, *J* = 6.5 Hz, CH_3), 22.28 (d, *J* = 8.1 Hz, CH_3), 21.33 (d, *J* = 4.4 Hz, CH_3). ^{31}P{^{1}H} NMR (162 MHz, cyclohexane-d_{12}) δ 89.66 (broad s, 2P).

Facial Assignment of Cobalt Complexes

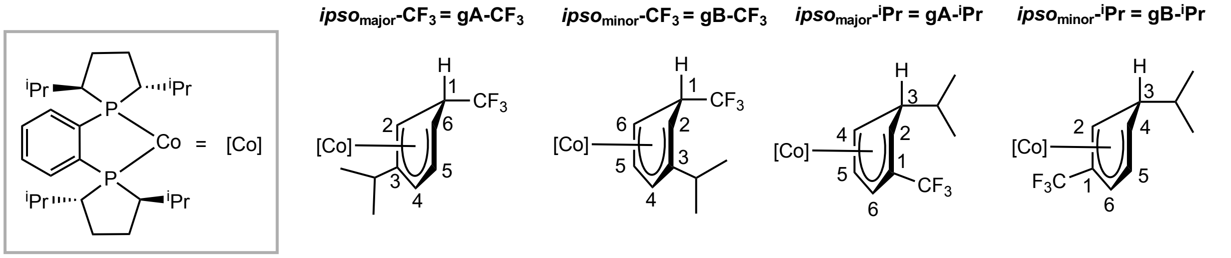

Preparation of Co1-g. This compound was prepared using General Procedure B using 0.0193 mmol of (*R,R*)-(^{iPr}DuPhos)Co(η^3-C_3H_5). A deep red solution formed was identified that contained (*R,R*)-(^{iPr}DuPhos)Co(η^5-$C_6H_5CF_3$iPr) *ipso*-CF_3 in 94% yield and 6% *ipso*-^{i}Pr, relative to 1,3,5-tris(trifluoromethyl)benzene. Using General Procedure C with 0.0579 mmol of (*R,R*)-(^{iPr}DuPhos)Co(η^3-C_3H_5), 92% yield of **Co1-g** was obtained as a red semi-solid and characterized by ^{1}H, ^{13}C{^{1}H}, ^{19}F, and ^{31}P{^{1}H} NMR spectroscopy. Residual arene was observed in the NMR spectra. ***Ipso*$_{major}$-CF$_3$** and ***ipso*$_{minor}$-CF$_3$** are denoted **gA-CF$_3$** and **gB-CF$_3$,** respectively. ***Ipso*$_{major}$-^{i}Pr** and ***ipso*$_{minor}$-^{i}Pr** are denoted **gA-^{i}Pr** and **gB-^{i}Pr**, respectively. **gA-CF$_3$** and **gB-CF$_3$** resonances were assigned using ^{1}H, ^{13}C{^{1}H}, ^{19}F, and ^{31}P{^{1}H} NMR spectroscopy. **gA-^{i}Pr** and **gB-^{i}Pr** resonances were assigned by ^{1}H and ^{19}F NMR spectroscopy due to low abundance in solution. ^{1}H NMR (400 MHz, cyclohexane-d_{12}) δ 7.66-7.51 (m, 6H, ^{iPr}DuPhos Ar), 7.22 (app s, 6H, ^{iPr}DuPhos Ar, overlapping solvent), 5.59 (app s, 1H, C^6H_{gA-iPr}), 5.31 (d, *J* = 5.3 Hz, 1H, C^4H_{gB-CF3}), 5.29 (app s, 1H, C^6H_{gB-iPr}), 5.18 (t, *J* = 6.0 Hz, 1H, C^5H_{gA-CF3}), 5.03 (t, *J* = 3.8 Hz, 1H, C^4H_{gA-CF3}), 5.02 (app s, 1H, C^5H_{gB-iPr}), 4.25 (m, 1H, C^5H_{gB-CF3}), 4.14 (app m, 1H, C^5H_{gA-iPr}), 4.07 (app m, 1H, C^4H_{gA-iPr}), 3.64 (t, *J* = 6.3 Hz, 1H, C^6H_{gB-CF3}), 3.63 (app m, 1H, C^2H_{gB-iPr}), 3.52 (app m, 1H, C^4H_{gB-iPr}), 3.38 (m, 1H, C^2H_{gA-CF3}), 3.12 (m, 1H, C^1H_{gB-CF3}), 3.07 (m, 2H, C^1H_{gA-CF3} + C^2H_{gA-CF3}), 2.87 (app m, 1H, C^2H_{gA-iPr}), 2.55 (m, 1H, C^2H_{gB-CF3}), 2.52-2.44 (m, 2H, CH_2), 2.38 (m, 1H, overlapping solvent + $CH_{;gB-CF3}$), 2.35 (m, 1H, overlapping solvent + $CH_{;gA-CF3}$), 2.29-2.07 (m, 10H, overlapping CH + CH_2), 2.07-1.90 (m, 4H, CH), 1.89 (broad m, 1H, C^3H_{gA-iPr}), 1.88 (broad m, 1H, C^3H_{gB-iPr}), 1.83-1.69 (m, 4H, CH), 1.69-1.45 (m, 12H, CH_2), 1.29 (app broad s, 3H, CH_3), 1.28 (m, 15H, CH_3 +

$CH_{3;gA/B\text{-}CF3}$), 1.08 (m, 6H, $CH_{3;gA/B\text{-}CF3}$), 1.05-0.98 (broad s, 6H, CH_3), 0.98- 0.91 (broad s, 6H, CH_3), 0.75- 0.64 (broad m, 15H, CH_3), 0.64 (broad s, 3H, CH_3), 0.62 (broad s, 3H, $CH_{3;}$), 0.59-0.51 (broad s, 6H, CH_3), 0.35 (broad s, 3H, CH_3). $CH(CH_3)_{2;gA\text{-}iPr}$ and $CH(CH_3)_{2;gB\text{-}iPr}$ could not be identified due to overlapping alkyl resonances. $^{13}C\{^1H\}$ NMR (101 MHz, cyclohexane-d_{12}) δ 149.58 (m, ^{iPr}DuPhos Ar), 131.7-130.39 (broad m, ^{iPr}DuPhos Ar), 126.25 (broad s, ^{iPr}DuPhos Ar), 124.08 (app broad m, CF_3; $_{A\text{-}CF3}$), 121.21 (app broad m, $CF_{3;B\text{-}CF3}$), 115.57 (s, $C^3_{gA\text{-}CF3}$), 114.62 (s, $C^3_{gB\text{-}CF3}$), 91.37 (s, $C^5_{gA+B\text{-}CF3}$), 75.80 (s, $C^4_{gB\text{-}CF3}$), 75.37 (s, $C^4_{gA\text{-}CF3}$), 59.00 (d, J = 14.4 Hz, CH), 56.04 (d, J = 17.6 Hz, CH), 54.31 (broad m, CH), 53.45 (broad m, CH), 50.86 (s, $C^6_{gA\text{-}CF3}$), 48.93 (s, $C^6_{gB\text{-}CF3}$), 44.83 (q, J = 29.0 Hz, $C^1_{gA\text{-}CF3}$), 44.11 (q, J = 26.5 Hz, $C^1_{gB\text{-}CF3}$), 37.33 (s, $C^2H_{gA\text{-}CF3}$), 35.02 (s, $CH_{;gA/B\text{-}CF3}$), 34.49 (s, $CH_{;gA/B\text{-}CF3}$ + $C^2_{gB\text{-}CF3}$), 32.84 (s, CH_2), 32.27 (s, CH_2), 31.84 (s, CH_2), 31.18 (s, CH), 29.98 (s, CH), 28.54 (s, CH_2), 26.16 (overlapping s, $CH_{3;A/B\text{-}CF3}$), 25.61 (app broad s, CH_3), 25.33 (s, $CH_{3;gA/B\text{-}CF3}$), 24.67 (app broad m, CH_3), 22.46 (s, $CH_{3;gA/B\text{-}CF3}$), 22.35 (d, J = 8.4 Hz, CH_3), 21.60 (app broad s, CH_3), 21.22 (app broad s, CH_3), 21.09 (s, $CH_{3;gA/B\text{-}CF3}$), 20.41 (broad s,). $^{31}P\{^1H\}$ NMR (162 MHz, cyclohexane-d_{12}) δ 97.54 (broad s, 1P, gA-CF_3), 95.52 (broad s, 1P, gB-CF_3), 84.57 (broad s, 1P, gB-CF_3), 76.23 (broad s, 1P, gA-CF_3). ^{19}F NMR (376 MHz, cyclohexane-d_{12}) δ -61.85 (s, 3F, gA-^{i}Pr), -61.90 (s, 3F, gB-^{i}Pr), -80.04 (d, J = 8.3 Hz, 3F,gB-CF_3), -80.83 (d, J = 7.6 Hz, 3F, gA-CF_3).

Facial Assignment of Cobalt Complexes

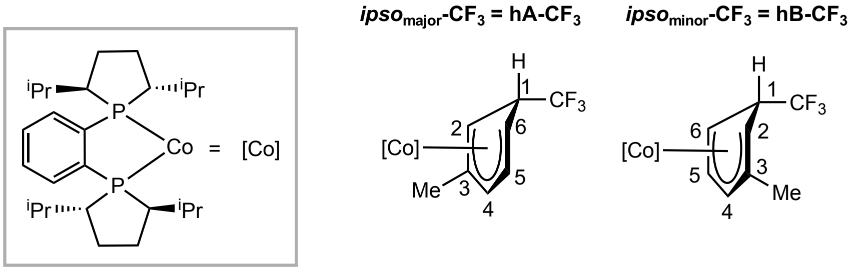

Preparation of Co1-h. This compound was prepared using General Procedure B using 0.0232 mmol of (R,R)-(^{iPr}DuPhos)Co(η^3-C_3H_5). A deep red solution formed that contained 99% yield of (R,R)-(^{iPr}DuPhos)Co(η^5-$C_6H_5CF_3Me$) *ipso*-CF_3 isomer and 1% yield *ipso*-Me isomer relative to

1,3,5-tris(trifluoromethyl)benzene. Using General Procedure A with 0.0193 mmol of (*R,R*)-(^{iPr}DuPhos)Co(η^3-C_3H_5), a deep red solution formed that contained 98% yield of **Co1-h** that was characterized by ^{1}H, ^{13}C{^{1}H}, ^{19}F, and ^{31}P{^{1}H} NMR spectroscopy. ***Ipso*** $\textbf{\textit{Ipso}}_{\textbf{major}}$**-CF$_3$** and $\textbf{\textit{ipso}}_{\textbf{minor}}$**-CF$_3$** are denoted **hA-CF$_3$** and **hB-CF$_3$,** respectively. **hA-CF$_3$** and **hB-CF$_3$** resonances were assigned using ^{1}H, ^{13}C{^{1}H}, ^{19}F, and ^{31}P{^{1}H} NMR spectroscopy. ^{1}H NMR (400 MHz, cyclohexane-*d*$_{12}$) δ 7.61 (m, 4H, ^{iPr}DuPhos Ar), 7.20 (m, 4H, ^{iPr}DuPhos Ar + overlapping solvent), 5.33 (d, *J* = 5.2 Hz, 1H, $C^4H_{hB\text{-}CF3}$), 5.14 (t, *J* = 6.0 Hz, 1H, $C^5H_{hA\text{-}CF3}$), 5.00 (app s, 1H, $C^4H_{hA\text{-}CF3}$), 4.26 (t, *J* = 6.5 Hz, 1H, $C^5H_{hB\text{-}CF3}$), 3.61 (t, J = 6.2 Hz, 1H, $C^6H_{hB\text{-}CF3}$), 3.34 (d, *J* = 5.1 Hz, 1H, $C^2H_{hA\text{-}CF3}$), 3.13 (m, 1H, $C^1H_{hB\text{-}CF3}$), 3.08 (m, 1H, $C^6H_{hA\text{-}CF3}$), 3.06 (m, 1H, $C^1H_{hA\text{-}CF3}$), 2.57 (app s, 1H, $C^2H_{hB\text{-}CF3}$), 2.28-2.18 (m, 8H, overlapping CH + CH_2), 2.16-2.04 (m, 4H, overlapping CH + CH_2), 1.94 (s, 3H, $CH_{3;B\text{-}CF3}$), 1.90 (s, 3H, $CH_{3;A\text{-}CF3}$), 1.86 (broad m, 2H, CH + CH_2), 1.82-1.65 (m, 10H, overlapping CH + CH_2), 1.64-1.51 (m, 8H, CH_2), 1.04 (d, *J* = 6.6 Hz, 6H, CH_3), 0.98 (d, *J* = 5.4 Hz, 6H, CH_3), 0.74-0.64 (td, *J* = 11.4, 6.3 Hz, 24H, CH_3), 0.58 (d, *J* = 6.7 Hz, 12H, CH_3). ^{13}C{^{1}H} NMR (101 MHz, cyclohexane-*d*$_{12}$) δ 149.70 (m, ^{iPr}DuPhos Ar), 131.11 (s, ^{iPr}DuPhos Ar), 128.61 (s, ^{iPr}DuPhos Ar), 124.28 (app broad m, $CF_{3;hB\text{-}CF3}$), 123.79 (app broad m, $CF_{3;hA\text{-}CF3}$), 103.07 (s, $C^3_{hA\text{-}CF3}$), 101.64 (s, $C^3_{hB\text{-}CF3}$), 92.00 (s, $C^5_{hB\text{-}CF3}$), 91.65 (s, $C^5_{hA\text{-}CF3}$), 77.85 (s, $C^4_{hA\text{-}CF3}$), 77.21 (s, $C^4_{hB\text{-}CF3}$), 54.48 (d, *J* = 19.3 Hz, CH), 52.75 (d, *J* = 18.3 Hz, CH), 49.73 (s, $C^6_{hA\text{-}CF3}$), 48.72 (s, $C^6_{hB\text{-}CF3}$), 44.75 (q, *J* = 27.5 Hz, $C^1_{hA\text{-}CF3}$), 44.22 (q, *J* = 27.5 Hz, $C^1_{hB\text{-}CF3}$), 42.70 (s, $C^2_{hA\text{-}CF3}$), 41.97 (s, $C^2_{hB\text{-}CF3}$), 33.78 (s, CH_2), 32.33 (d, *J* = 4.9 Hz, CH), 31.62 (app s, CH+ CH_2), 31.30 (app dd, CH + CH_2), 25.60 (d, *J* = 4.8 Hz, CH_3), 25.26 (app broad m, CH_3), 24.94 (m, CH_3), 23.08 (s, $CH_{3;hB\text{-}CF3}$), 22.88 (s, $CH_{3;hA\text{-}CF3}$), 22.40 (m, CH_3), 21.38 (overlapping broad s, CH_3). ^{31}P{^{1}H} NMR (162 MHz, cyclohexane-*d*$_{12}$) δ 98.32 (broad s, 1P, hA-CF_3), 92.01 (broad s, 2P, hB-CF_3), 77.39 (broad s, 1P, hA-CF_3). ^{19}F NMR (376 MHz, cyclohexane-*d*$_{12}$) δ -80.20 (d, *J* = 8.5 Hz, 3F, hB-CF_3), -80.84 (d, *J* = 7.8 Hz, 3F, hA-CF_3).

Facial Assignment of Cobalt Complexes

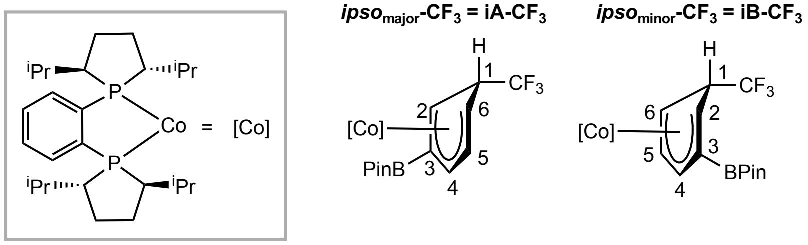

Preparation of Co1-i. This compound was prepared using General Procedure A using 0.0193 mmol of (*R,R*)-(^{iPr}DuPhos)Co(η^3-C_3H_5). A deep red solution formed that contained 97% yield of (*R,R*)-(^{iPr}DuPhos)Co(η^5-$C_6H_5CF_3BPin$). *ipso*-CF_3 was the only observable isomer relative to HMDSO. ***Ipso*$_{\mathbf{major}}$-$\mathbf{CF_3}$** and ***ipso*$_{\mathbf{minor}}$-$\mathbf{CF_3}$** are denoted **iA-$\mathbf{CF_3}$** and **iB-$\mathbf{CF_3}$,** respectively. **iA-$\mathbf{CF_3}$** and **iB-$\mathbf{CF_3}$** resonances were assigned using ^{1}H, ^{13}C{^{1}H}, ^{19}F, and ^{31}P{^{1}H} NMR spectroscopy. ^{1}H NMR (400 MHz, cyclohexane-*d*$_{12}$) δ 7.72 (m, 4H, overlapping solvent + ^{iPr}DuPhos Ar), 7.29 (m, 4H, overlapping solvent + ^{iPr}DuPhos Ar), 5.77 (d, *J* = 5.4 Hz, 1H, $C^4H_{iA\text{-}CF3}$), 5.57 (t, *J* = 4.6 Hz, 1H, $C^4H_{iB\text{-}CF3}$), 5.08 (m, 1H, $C^5H_{iB\text{-}CF3}$), 4.35 (t, *J* = 5.8 Hz, 1H, $C^5H_{iA\text{-}CF3}$), 3.64 (t, J = 6.2 Hz, 1H, $C^6H_{iA\text{-}CF3}$), 3.30 (app s, 1H, $C^6H_{iB\text{-}CF3}$), 3.14 (m, 2H, $C^2H_{iB\text{-}CF3}$ + $C^1{}_{iA\text{-}CF3}$), 2.98 (m, 1H, $C^1{}_{iB\text{-}CF3}$), 2.80 (m, 1H, $C^2H_{iA\text{-}CF3}$), 2.55- 2.41 (m, 1H, CH), 2.37-2.21 (m, 6H, overlapping CH + CH_2), 2.21-2.09 (m, 4H, overlapping CH + CH_2), 2.09-1.93 (m, 2H, CH_2), 1.96- 1.79 (s, 4H, overlapping CH + CH_2), 1.76-1.46 (m, 15H, overlapping CH + CH_2), 1.21 (s, 12H, $CH_{3;iB\text{-}CF3}$), 1.19 (s, 12H, $CH_{3;iA\text{-}CF3}$), 0.99 (d, *J* = 6.0 Hz, 6H, CH_3), 0.90 (overlapping d, 6H, CH_3), 0.76 (d, *J* = 6.5 Hz, 9 H, CH_3), 0.69 (d, *J* = 5.6 Hz, 9H, CH_3), 0.58 (d, *J* = 6.0 Hz, 6H, CH_3), 0.52 (d, *J* = 5.1 Hz, 6H, CH_3), 0.43 (d, *J* = 5.5 Hz, 6H, CH_3). ^{13}C{^{1}H} NMR (101 MHz, cyclohexane-*d*$_{12}$) δ 149.84 (m, ^{iPr}DuPhos Ar), 148.66 (m, ^{iPr}DuPhos Ar), 130.99 (s, ^{iPr}DuPhos Ar), 128.72 (d, *J* = 11.3 Hz, ^{iPr}DuPhos Ar), 123.23 (app m, $CF_{3;iA\text{-}CF3}$), 122.71(app m, $CF_{3;iB\text{-}CF3}$), 94.35/34 (s, $C^5{}_{iA\text{-}CF3}$ + $C^5{}_{iB\text{-}CF3}$), 84.18 (s, $C^4{}_{iA\text{-}CF3}$ + $C^4{}_{iB\text{-}CF33}$), 83.11 (s, $C(O)(C)(CH_3)_{2;iB\text{-}CF3}$), 83.07 (s, $C(O)(C)(CH_3)_{2;iA\text{-}CF3}$), 82.52 (app broad s, $C^3{}_{iB\text{-}CF3}$), 80.65 (app broad s, $C^3{}_{iA\text{-}CF3}$), 57.59 (d, *J* = 20.4 Hz, CH), 56.28 (d, *J* = 15.0 Hz, CH), 54.30 (d, *J* = 21.9 Hz, CH), 52.70 (d, *J* = 16.3 Hz, CH), 47.69 (broad s, $C^2{}_{iB\text{-}CF3}$), 46.19 (s, $C^6{}_{iA\text{-}CF3}$), 43.70 (q, *J* = 29.0

Hz, $C^1_{iA\text{-}CF3}$ + $C^1_{iB\text{-}CF3}$), 38.72 (s, $C^2_{iA\text{-}CF3}$), 37.37 (app t, $C^6_{iB\text{-}CF3}$), 34.34 (broad s, CH_2), 32.26 (d, *J* = 4.6 Hz, CH), 32.00 (broad s, CH_2), 31.07 (d, *J* = 7.2 Hz, CH), 30.75-30.29 (broad m, CH + CH_2), 30.11 (broad s, CH_2), 25.84 (app s, $CH_{3;iA\text{-}CF3}$ + $CH_{3;iB\text{-}CF3}$), 22.89 (app m, CH_3), 22.74-22.55 (app m, CH_3), 21.79 (d, *J* = 4.7 Hz, CH_3), 21.52 (app broad m, CH_3), 21.02 (d, *J* = 4.1 Hz, CH_3), 20.79 (app broad m, CH_3). Additional ^{iPr}DuPhos CH/CH_2 resonances could not be assigned due to overlapping signals with starting material and poor HMBC/HSQC resolution. $^{31}P\{^1H\}$ NMR (162 MHz, cyclohexane-d_{12}) δ 96.71 (broad s, 1P, iB-CF_3), 95.20 (broad s, 1P, iA-CF_3), 89.56 (broad s, 1P, iA-CF_3), 77.76 (broad s, 1P, iB-CF_3). ^{19}F NMR (376 MHz, cyclohexane-d_{12}) δ -79.55 (d, *J* = 8.3 Hz, iA-CF_3), -80.32 (d, *J* = 8.7 Hz, iB-CF_3).

Facial Assignment of Cobalt Complexes

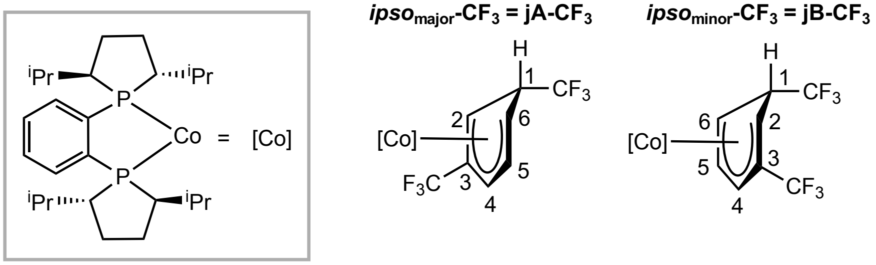

Preparation of Co1-j. This compound was prepared using General Procedure B using 0.0154 mmol of (*R,R*)-(^{iPr}DuPhos)Co(η^3-C_3H_5). A deep red-orange solution formed that contained 70% yield of (*R,R*)-(^{iPr}DuPhos)Co(η^5-$C_6H_5CF_3CF_3$) *ipso*-CF_3 as the only observable isomer, 6% yield of **Co1-H**, and 24% mass balance lost relative to 1,3,5-tris(trifluoromethyl)benzene. ***Ipso*$_{major}$-CF$_3$** and ***ipso*$_{minor}$-CF$_3$** are denoted **jA-CF$_3$** and **jB-CF$_3$,** respectively*.* **jA-CF$_3$** and **jB-CF$_3$** resonances were assigned using ^1H, $^{13}C\{^1H\}$, ^{19}F, and $^{31}P\{^1H\}$ NMR spectroscopy. ^1H NMR (400 MHz, cyclohexane-d_{12}) δ 7.64 (m, 4H, overlapping solvent + ^{iPr}DuPhos Ar), 7.30 (m, 4H, overlapping solvent + ^{iPr}DuPhos Ar), 5.70 (d, *J* = 4.6 Hz, 1H, $C^4H_{jB\text{-}CF3}$), 5.34 (app s, 1H, $C^4H_{jA\text{-}CF3}$), 5.23 (t, *J* = 6.3 Hz, 1H, $C^5H_{jA\text{-}CF3}$), 4.34 (t, *J* = 6.6 Hz, 1H, $C^5H_{jB\text{-}CF3}$), 3.78 (t, *J* = 6.5 Hz, 1H, $C^6H_{jB\text{-}CF3}$), 3.42 (d, *J* = 5.8 Hz, 1H, $C^2H_{jA\text{-}CF3}$), 3.29 (t, *J* = 6.4 Hz, $C^6H_{jA\text{-}CF3}$), 3.16 (m, 1H, $C^1H_{jB\text{-}CF3}$), 3.04 (m, 1H, $C^1H_{jA\text{-}CF3}$), 2.66 (app s, 1H, $C^2H_{jB\text{-}CF3}$), 2.50-2.17 (m, 12H, overlapping CH + CH_2), 2.16-2.04 (m,

4H, overlapping CH + CH_2), 1.79- 1.61 (m, 8H, overlapping CH + CH_2), 1.61- 1.45 (m, 8H, overlapping CH + CH_2), 1.00 (broad m, 9H, CH_3), 0.95 (m, 6H, CH_3), 0.78 (broad s, 6H, CH_3), 0.68-0.51 (m, 27H, CH_3). $^{13}C\{^1H\}$ NMR (101 MHz, cyclohexane-d_{12}) δ 129.21 (app s, ^{iPr}DuPhos Ar + overlapping starting material), 128.65 (app broad s, ^{iPr}DuPhos Ar + overlapping starting material), 122.85 (app m, $C(sp^3)$-$CF_{3;jB\text{-}CF3}$ + overlapping with starting material), 122.42 (app broad m, $C(sp^3)$-$CF_{3;jA\text{-}CF3}$ + overlapping with starting material), 93.45 (s, $C^5_{jA\text{-}CF3}$), 91.67 (s, $C^5_{jB\text{-}CF3}$), 74.52 (s, $C^4_{jB\text{-}CF3}$), 73.50 (s, $C^4_{jA\text{-}CF3}$), 59.56 (app t, CH), 56.51 (d, J = 15.4 Hz, CH), 54.57 (d, J = 19.0 Hz, CH), 52.85 (d, J = 16.3 Hz, CH), 51.03 (s, $C^6_{jA\text{-}CF3}$), 48.70 (s, $C^6_{jB\text{-}CF3}$), 43.96 (q, J = 28.0 Hz, $C^1_{jA\text{-}CF3}$), 43.48 (q, J = 31.6 Hz, $C^1_{jB\text{-}CF3}$), 33.35 (app broad m, CH), 32.13 (s, CH_2), 30.28 (app broad s, CH), 29.45 (app d, CH_3 + $C^2_{jA\text{-}CF3}$), 29.16 (broad s, CH_2), 28.40 (s, $C^2_{jB\text{-}CF3}$), 27.44 (app broad d, CH_2), 25.68 (app broad s, CH_3), 25.04 (d, J = 8.4 Hz, CH_3), 24.56 (app broad s, CH_3), 22.27 (d, J = 10.0 Hz, CH_3). Additional ^{iPr}DuPhos Ar , C^3, C^3-$CF_{3;iA/B\text{-}CF3}$, ^{iPr}DuPhos CH/CH_2/CH_3 resonances could not be assigned due to peak broadening, overlapping signals with starting material, and poor HMBC/HSQC resolution. $^{31}P\{^1H\}$ NMR (162 MHz, cyclohexane-d_{12}) δ 96.70 (broad s, 2P, jA/B-CF_3), 84.14 (broad s, 1P, jB-CF_3), 75.39 (broad s, 1P, jA-CF_3). ^{19}F NMR (376 MHz, cyclohexane-d_{12}) δ -80.05 (d, J = 8.3 Hz, 3F, jA-CF_3), -80.84 (d, J = 8.0 Hz, 3F, jB-CF_3), -62.51 (s, 3F, jA/B-CF_3), -62.56 (s, 3F, jA/B-CF_3), -75.48 (d, J = 7.8 Hz, 1,3-bis(trifluoromethyl)cyclohexane, generated from unidentified [Co] side-product following arene insertion).

Facial Assignment of Cobalt Complexes

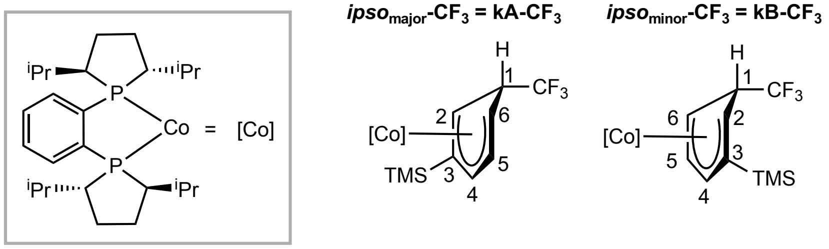

Preparation of Co1-k. This compound was prepared using General Procedure B using 0.0154 mmol of (*R,R*)-(^{iPr}DuPhos)Co(η^3-C_3H_5). A deep red solution formed that contained 91% yield of (*R,R*)-(^{iPr}DuPhos)Co(η^5-$C_6H_5CF_3$TMS), where *ipso*-CF_3 insertion was the only observable isomer and 9% yield of unidentified [Co] products relative to 1,3,5-tris(trifluoromethyl)benzene. ***Ipso*$_{major}$-CF$_3$** and ***ipso*$_{minor}$-CF$_3$** are denoted **kA-CF$_3$** and **kB-CF$_3$,** respectively. **kA-CF$_3$** and **kB-CF$_3$** resonances were assigned using ^{1}H, ^{13}C{^{1}H}, ^{19}F, and ^{31}P{^{1}H} NMR spectroscopy. ^{1}H NMR (400 MHz, cyclohexane-*d*$_{12}$) δ 7.60 (m, 4H, overlapping solvent + ^{iPr}DuPhos Ar), 7.26 (m, 4H, overlapping solvent + ^{iPr}DuPhos Ar), 5.49 (app s, 1H, $C^4H_{kB\text{-}CF3}$), 5.23 (app m, 2H, $C^4H_{kA\text{-}CF3}$ + $C^5H_{kA\text{-}CF3}$), 4.31 (app s, $C^5H_{kB\text{-}CF3}$), 3.77 (t, *J* = 4.8 Hz, 1H, $C^6H_{kB\text{-}CF3}$), 3.22 (app s, 2H, $C^6H_{kA\text{-}CF3}$ + $C^2H_{kA\text{-}CF3}$), 3.13 (app s, 1H, $C^1H_{kB\text{-}CF3}$), 3.00 (m, 1H, $C^1H_{kA\text{-}CF3}$), 2.55- 2.41 (m, 2H, overlapping CH + CH_2), 2.46 (m, 1H, $C^2H_{kB\text{-}CF3}$), 2.40-2.00 (m, 12, overlapping CH + CH_2), 1.99-1.83 (m, 4H, CH_2), 1.78- 1.67 (m, 6H, overlapping CH + CH_2), 1.66- 1.49 (m, 8H, overlapping CH + CH_2), 1.11 (d, *J* = 5.9 Hz, 3H, CH_3), 1.03 (d, *J* = 6.9 Hz, 3H, CH_3), 0.94 (overlapping d, 6H, CH_3), 0.78- 0.71 (m, 9H, CH_3), 0.71-0.65 (d, *J* = 6.5 Hz, 12H, CH_3), 0.58 (d, *J* = 6.4 Hz, 3H, CH_3), 0.50 (d, *J* = 5.5 Hz, 6H, CH_3), 0.41 (m, 6H, CH_3), 0.21 (overlapping s, 18H, $Si(CH_3)_{3;\ kA/B\text{-}CF3}$). ^{13}C{^{1}H} NMR (101 MHz, cyclohexane-*d*$_{12}$) δ 149.89-149.00 (m, ^{iPr}DuPhos Ar), 131.03(overlapping m, ^{iPr}DuPhos Ar), 128.88 (app m, ^{iPr}DuPhos Ar), 124.42 (app broad m, $CF_{3;kB\text{-}CF3}$), 123.82 (app broad m, $CF_{3;kA\text{-}CF3}$), 97.16 (s, $C^3_{kA/B\text{-}CF3}$), 94.59 (s, $C^5_{kA\text{-}CF3}$), 94.48 (s, $C^3_{kA/B\text{-}CF3}$), 94.12 (s, $C^5_{kB\text{-}CF3}$), 82.80 (s, $C^4_{kB\text{-}CF3}$), 80.79 (s, $C^4_{kA\text{-}CF3}$), 58.91 (app d, CH_2), 54.50 (d, *J* = 17.5 Hz, CH), 54.03 (d, *J* = 21.7 Hz, CH), 53.82 (d, *J* = 22.0 Hz, CH), 53.30 (s, CH_2), 52.21 (d, *J* = 9.0 Hz, CH), 50.34 (d, *J* = 14.8 Hz, CH),

50.93 (s, C^6_{kA-CF3}), 47.83 (s, C^6_{kB-CF3}), 43.53 (q, *J* = 29.4 Hz, C^1_{kA-CF3}), 43.05 (q, *J* = 28.9 Hz, C^1_{kB-CF3}), 38.53 (app t, C^2_{kA-CF3}), 36.37 (app t, C^2_{kB-CF3}), 33.53 (app broad s, CH), 32.65 (s, CH_2), 32.33 (s, CH_2), 32.08 (d, *J* = 5.6 Hz, CH), 31.79 (app m, overlapping CH +_CH_2), 30.72 (m, overlapping CH +_CH_2), 29.91 (m, m, overlapping CH +_CH_2), 26.44 (overlapping m, CH_3), 25.62 (d, *J* = 6.5 Hz, CH_3), 25.32 (d, *J* = 6.5 Hz, CH_3), 24.77 (app t, CH_3), 24.41 (d, *J* = 6.9 Hz, CH_3), 22.82 (d, *J* = 8.3 Hz, CH_3), 22.54 (d, *J* = 8.3 Hz, CH_3), 22.34 (d, *J* = 8.2 Hz, CH_3), 22.12 (d, *J* = 6.9 Hz, CH_3), 21.71 (d, *J* = 5.8 Hz, CH_3), 21.48 (d, *J* = 3.4 Hz, CH_3), 20.88 (app s, CH_3), -0.32 (s, $Si(CH_3)_{3;kA/B-CF3}$). $^{31}P\{^1H\}$ NMR (162 MHz, cyclohexane-d_{12}) δ 98.25 (broad s, 1P, kA-CF_3), 95.66 (broad s, 1P, kB-CF_3), 83.89 (broad s, 1P, kB-CF_3), 74.67 (broad s, 1P, kA-CF_3). ^{19}F NMR (376 MHz, cyclohexane-d_{12}) δ -79.51 (d, *J* = 8.5 Hz, 3F, kB-CF_3), -80.40 (d, *J* = 8.4 Hz, 3F, kA-CF_3).

Facial Assignment of Cobalt Complexes

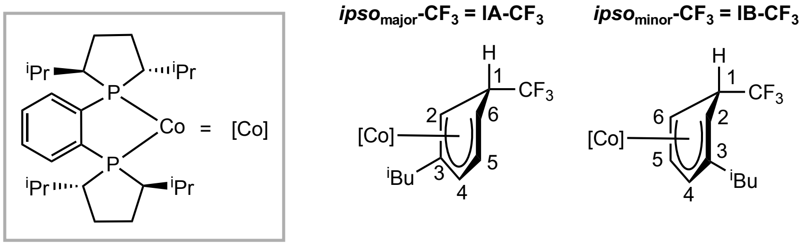

Preparation of Co1-I. This compound was prepared using General Procedure B using 0.0073 mmol of (*R,R*)-(^{iPr}DuPhos)Co(η^3-C_3H_5). A deep red solution formed that contained 94% yield of (*R,R*)-(^{iPr}DuPhos)Co(η^5-$C_6H_5CF_3{}^iBu$), where *ipso*-CF_3 insertion was the only observable isomer, and a 6% yield of unidentified [Co] products relative to 1,3,5-tris(trifluoromethyl)benzene. ***Ipso*$_{major}$-CF$_3$** and ***ipso*$_{minor}$-CF$_3$** are denoted **IA-CF$_3$** and **IB-CF$_3$,** respectively. **IA-CF$_3$** and **IB-CF$_3$** resonances were assigned using ^1H, $^{13}C\{^1H\}$, ^{19}F, and $^{31}P\{^1H\}$ NMR spectroscopy. ^1H NMR (400 MHz, cyclohexane-d_{12}) δ 7.58 (app m, 4H, ^{iPr}DuPhos Ar + overlapping solvent), 7.39-7.30 (app m, 4H, overlapping solvent + ^{iPr}DuPhos Ar), 5.32 (d, *J* = 5.2 Hz, 1H, C^4H_{IB-CF3}), 5.16 (t, *J* = 5.9 Hz, C^5H_{IA-CF3}), 4.98 (app s, 1H, C^4H_{IA-CF3}), 4.28 (app s, 1H, C^5H_{IB-CF3}), 3.62 (t, *J* = 6.4 Hz, 1H, C^6H_{IB-CF3}), 3.34 (app s, 1H, C^2H_{IA-CF3}), 3.16 (app s, 1H, C^1H_{IB-CF3}), 3.09 (m, 2H, C^6H_{IA-CF3} + C^1H_{IA-CF3}),

2.57 (app s, 1H, $C^2H_{IB\text{-}CF3}$), 2.39 – 2.19 (m, 17H, CH + CH_2 + $CH_{;IA/B\text{-}CF3}$), 2.06 (d, 2H, $CH_{2;IA/B\text{-}CF3}$), 1.99 (m, 2H, $CH_{2;IA/B\text{-}CF3}$), 1.83 (m, 1H, overlapping solvent + $CH_{IA/B\text{-}CF3}$ + $CH_{2;IA/B\text{-}CF3}$), 1.69-1.54 (m, 20H, CH_2 + CH), 1.12 – 1.03 (m, 16H, CH_3 + $CH_{3;IA/B\text{-}CF3}$), 0.98 (m, 16H, CH_3 + $CH_{3;IA/B\text{-}CF3}$), 0.71 (m, 27H, CH_3), 0.58 (m, 12H, CH_3). $^{13}C\{^1H\}$NMR (101 MHz, cyclohexane-d_{12}) δ 131.05 (d, J = 14.3 Hz, ^{iPr}DuPhos Ar), 128.58 (app s, ^{iPr}DuPhos Ar), 123.64 (app broad m, $CF_{3;IA\text{-}CF3}$), 121.30 (app broad m, $CF_{3;IB\text{-}CF3}$), 106.58 (s, $C^3_{IA\text{-}CF3}$), 105.32 (s, $C^3_{IB\text{-}CF3}$), 92.09 (s, $C^5_{IA\text{-}CF3}$), 91.98 (s, $C^5_{IB\text{-}CF3}$), 78.55 (s, $C^4_{IA\text{-}CF3}$), 77.67 (s, $C^4_{IB\text{-}CF3}$), 54.47 (d, J = 19.5 Hz, CH), 52.91 (d, J = 17.5 Hz, CH), 49.88 (s, $C^6_{IA\text{-}CF3}$), 49.07 (s, $CH_{2;IA/B\text{-}CF3}$), 48.80 (s, $CH_{2;IA/B\text{-}CF3}$), 48.43 (s, $C^6_{IB\text{-}CF3}$), 44.52 (q, J = 25.4 Hz, $C^1_{IA/B\text{-}CF3}$), 42.37 (s, $C^2_{IA\text{-}CF3}$), 42.16 (s, $C^2_{IB\text{-}CF3}$), 32.69 (s, $CH_{;IA/B\text{-}CF3}$), 32.45 (s, $CH_{;IA/B\text{-}CF3}$), 32.36 (d, J = 5.5 Hz, CH), 31.61 (app broad s, CH), 30.62 (s, CH_2), 25.64 (d, J = 4.7 Hz, CH_3), 24.90 (app broad s, CH_3), 23.21 (s, $CH_{3;IA/B\text{-}CF3}$), 22.50 – 22.12 (s, $CH_{3;IA/B\text{-}CF3}$ + overlapping solvent), 21.33 (d, J = 3.2 Hz, CH_3). Additional ^{iPr}DuPhos Ar and ^{iPr}DuPhos CH/CH_2/CH_3 resonances could not be assigned due to peak broadening, overlapping signals with starting material, and poor HMBC/HSQC resolution. $^{31}P\{^1H\}$ NMR (162 MHz, cyclohexane-d_{12}) δ 98.45 (broad s, 2P, IA/B-CF_3), 76.54 (broad s, 2P, IA/B-CF_3). ^{19}F NMR (376 MHz, cyclohexane-d_{12}) δ -79.85 (d, J = 8.6 Hz, IB-CF_3), -80.48 (d, J = 7.9 Hz, IA-CF_3).

Facial Assignment of Cobalt Complexes

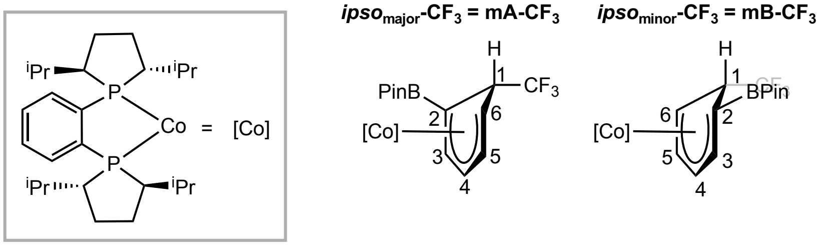

Preparation of Co1-m. This compound was prepared using General Procedure A using 0.0193 mmol of (*R,R*)-(^{iPr}DuPhos)Co(η^3-C_3H_5). A deep red solution formed that contained 94% yield of (*R,R*)-(^{iPr}DuPhos)Co(η^5-$C_6H_5CF_3$BPin), where *ipso*-CF_3 insertion as only observable isomer and 5% of **Co1-H** relative to HMDSO. ***Ipso*$_{\mathbf{major}}$-CF$_3$** and ***ipso*$_{\mathbf{minor}}$-CF$_3$** are denoted **mA-CF$_3$** and **mB-**

CF_3, respectively. **mA-CF_3** and **mB-CF_3** resonances were assigned using ^{1}H, ^{13}C{^{1}H}, ^{19}F, and ^{31}P{^{1}H} NMR spectroscopy. ^{1}H NMR (400 MHz, cyclohexane-d_{12}) δ 7.56 (m, 4H, overlapping solvent + ^{iPr}DuPhos Ar), 7.23 (m, 4H, overlapping solvent + ^{iPr}DuPhos Ar), 5.59 (t, *J* = 5.4 Hz, 1H, $C^4H_{mA\text{-}CF3}$), 5.43 (d, *J* = 5.4 Hz, 1H, $C^3H_{mB\text{-}CF3}$), 5.21 (t, J = 5.6 Hz, 1H, $C^5H_{mB\text{-}CF3}$), 5.14 (t, *J* = 5.6 Hz, 1H, $C^5H_{mA\text{-}CF3}$), 4.96 (app s, 1H, $C^4H_{mB\text{-}CF3}$), 4.84 (d, *J* = 5.4 Hz, 1H, $C^3{}_{mA\text{-}CF3}$), 3.19 (m, 1H, $C^1H_{mA\text{-}CF3}$), 3.12 (m, 1H, $C^1H_{mB\text{-}CF3}$), 3.06 (m, 1H, $C^6H_{mB\text{-}CF3}$), 2.50 (app s, 1H, $C^6H_{mA\text{-}CF3}$), 2.23-2.05 (m, 12H, overlapping CH + CH_2), 1.92 – 1.78 (m, 4H, overlapping CH + CH_2), 1.74-1.45 (m, 16H, overlapping CH + CH_2), 1.17 (app d, 12H, $CH_{3;mB\text{-}CF3}$), 1.15 (app d, 12H, $CH_{3;mA\text{-}CF3}$), 1.03 (broad s, 9H, CH_3), 0.94 (broad m, 9H, CH3), 0.68 (m, 9H, CH_3), 0.60 (broad s, 9H, CH_3), 0.55 (d, *J* = 6.6 Hz, 9H, CH_3). ^{13}C{^{1}H} NMR (101 MHz, cyclohexane-d_{12}) δ 131.08 (d, *J* = 14.4 Hz, ^{iPr}DuPhos Ar), 128.74 (app s, ^{iPr}DuPhos Ar), 124.32 (app broad m, $CF_{3;mA\text{-}CF3}$), 123.76 (app broad m, $CF_{3;mB\text{-}CF3}$), 99.30 (s, $C^3{}_{mB\text{-}CF3}$), 98.90 (s, $C^3{}_{mA\text{-}CF3}$), 88.73 (s, $C^5{}_{mB\text{-}CF3}$), 88.07 (s, $C^5{}_{mA\text{-}CF3}$), 82.89 (s, $C(O)(CH_3)_{2;mA/B\text{-}CF3}$), 82.73 (s, $C(O)(CH_3)_{2;mA/B\text{-}CF3}$), 82.48 (s, $C^4{}_{mB\text{-}CF3}$), 81.28 (s, $C^4{}_{mA\text{-}CF3}$), 52.56 (broad s, CH), 47.33 (app broad m, $C^2{}_{mB\text{-}CF3}$), 46.53 (app broad m, $C^2{}_{mA\text{-}CF3}$), 41.89 (q, *J* = 27.7 Hz, $C^1{}_{mB\text{-}CF3}$), 41.44 (q, *J* = 27.7 Hz, $C^1{}_{mA\text{-}CF3}$), 37.71 (s, $C^6{}_{mA\text{-}CF3}$), 37.28 (s, $C^6{}_{mB\text{-}CF3}$), 31.13 (app d, CH_2), 26.55 (overlapping m, $CH_{3;mA/B\text{-}CF3}$), 25.83 (s, $CH_{3;mA/B\text{-}CF3}$), 22.39 (broad m, CH_3), 21.32 (broad s, CH_3). Additional ^{iPr}DuPhos Ar and ^{iPr}DuPhos CH/CH_2/CH_3 resonances could not be assigned due to peak broadening, overlapping signals with starting material, and poor HMBC/HSQC resolution. ^{31}P{^{1}H} NMR (162 MHz, cyclohexane-d_{12}) δ 96.51(broad s, 1P, mB-CF_3), 94.11 (broad s, 1P, mA-CF_3), 84.24 (broad s, 1P, mB-CF_3), 83.12 (broad s, 1P, mA-CF_3). ^{19}F NMR (376 MHz, cyclohexane-d_{12}) δ -78.33 (d, *J* = 8.6 Hz, 3F, mB-CF_3), -79.46 (d, *J* = 8.3 Hz, 3F, mA-CF_3).

Facial Assignment of Cobalt Complexes

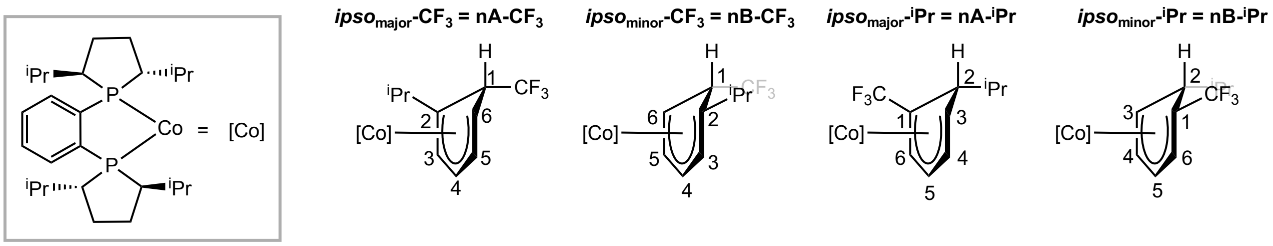

Preparation of Co1-n. This compound was prepared using General Procedure B using 0.021 mmol of (*R,R*)-(^{iPr}DuPhos)Co(η^3-C_3H_5). A deep red solution formed that contained 88% yield of (*R,R*)-(^{iPr}DuPhos)Co(η^5-$C_6H_5CF_3{}^iPr$), where 78% yield was *ipso*-CF_3 isomers, 21% *ipso*-alkyl isomers, and 12% mass balance unaccounted for relative to 1,3,5-tris(trifluoromethyl)benzene after 24 h. Over the course of 5 days, *ipso*-CF_3 yield decreased to 60% and *ipso*-alkyl yield increased to 32%. Using General Procedure C with 0.0568 mmol of (*R,R*)-(^{iPr}DuPhos)Co(η^3-C_3H_5), 86% yield of **Co1-n** was obtained as a red semi-solid and characterized by ^{1}H, ^{13}C{^{1}H}, ^{19}F, and ^{31}P{^{1}H} NMR spectroscopy. Residual arene was observed in the NMR spectra. ***Ipso*$_{major}$-CF_3**, ***ipso*$_{minor}$-CF_3**, ***ipso*$_{major}$-^{i}Pr** and ***ipso*$_{minor}$-^{i}Pr** are denoted **nA-CF_3**, **nB-CF_3**, **nA-^{i}Pr**, and **nB-^{i}Pr**, respectively. **nA-CF_3**, **nB-CF_3**, and **nA-^{i}Pr** resonances were assigned using ^{1}H, ^{13}C{^{1}H}, ^{19}F, and ^{31}P{^{1}H} NMR spectroscopy. **nB-^{i}Pr** resonances were assigned by ^{19}F NMR spectroscopy due to low abundance in solution. ^{1}H NMR (400 MHz, cyclohexane-d_{12}) δ 7.53 (m, 6H, overlapping solvent + ^{iPr}DuPhos Ar), 7.21 (overlapping app s, 6H, overlapping solvent + ^{iPr}DuPhos Ar), 5.51 (d, *J* = 5.5 Hz, 1H, $C^6H_{nA\text{-}iPr}$), 5.37 (t, *J* = 5.0 Hz, 1H, $C^4H_{nB\text{-}CF3}$), 5.20 (d, *J* = 4.3 Hz, 1H, $C^3H_{nB\text{-}CF3}$), 5.10 (t, *J* = 6.0 Hz, 1H, $C^4H_{nA\text{-}iPr}$), 4.88- 4.83 (app dd, 2H, $C^5H_{nA\text{-}CF3}$ + $C^5H_{nB\text{-}CF3}$), 4.76 (app s, 1H, $C^4H_{nA\text{-}CF3}$), 4.71 (app m, 1H, $C^5H_{nA\text{-}iPr}$), 4.47 (d, *J* = 4.3 Hz, 1H, $C^3H_{nA\text{-}CF3}$), 3.67 (t, *J* = 5.8 Hz, 1H, $C^3H_{nA\text{-}iPr}$), 3.26-3.18 (app t, 2H, $C^6H_{nB\text{-}CF3}$ + $C^1H_{nA\text{-}CF3}$), 3.13 (m, 1H, $C^1H_{nB\text{-}CF3}$), 2.75 (app s, 1H, $C^2H_{nA\text{-}iPr}$), 2.51 (app s, 1H, $C^6H_{nA\text{-}CF3}$), 2.34-2.20 (m, 12H, overlapping solvent, CH_2 + CH), 2.19 (m, 1H, $CH_{;nA\text{-}CF3}$), 2.16 (m, 1H, $CH_{;nB\text{-}CF3}$), 2.15-2.03 (m, 6H, overlapping solvent, CH_2 + CH), 1.91 – 1.79(m, 6H, CH), 1.77-1.69 (m, 4H, CH_2), 1.66-1.49 (m, 20H, CH_2), 1.23 (m, 1H, $CH_{;nA\text{-}iPr}$), 1.13 (d, *J* = 2.8 Hz, 6 H, $CH_{3;nA/B\text{-}CF3}$), 1.10 (d, *J* = 1.9 Hz, 6 H, $CH_{3;nA/B\text{-}CF3}$), 1.11 (app s, 9H, CH_3),

1.05 (app d, 9H, CH_3), 0.99 (broad s, 9H, CH_3), 0.83- 75 (broad m, 12H, CH_3), 0.72 (m, 6H, CH_3), 0.69 (d, J = 6.8 Hz, 3H, $CH_{3;nA/B\text{-}iPr}$), 0.65 (d, J = 6.8 Hz, 3H, $CH_{3;nA/B\text{-}iPr}$), 0.55 (overlapping d, 27H, CH_3). $^{13}C\{^1H\}$ NMR (101 MHz, cyclohexane-d_{12}) δ 149.29 (app broad m, ^{iPr}DuPhos Ar), 130.85 (d, J = 14.4 Hz, ^{iPr}DuPhos Ar), 129.71 (app broad m, $CF_{3;nA\text{-}iPr}$), 128.54 (s, ^{iPr}DuPhos Ar), 123.47 (app broad m, $CF_{3;nA\text{-}CF3}$), 122.91 (app broad m, $CF_{3;nA\text{-}CF3}$), 95.93 (s, $C^3_{nB\text{-}CF3}$), 94.94 (s, $C^3_{nA\text{-}CF3}$), 90.56 (q, J = 5.3 Hz, $C^6_{nA\text{-}iPr}$), 89.73 (s, $C^4_{nA\text{-}iPr}$), 85.92 (s, $C^2_{nB\text{-}CF3}$), 85.49 (s, $C^5_{nA/B\text{-}CF3}$), 84.22 (s, $C^5_{nA/B\text{-}CF3}$), 80.98 (s, $C^2_{nA\text{-}CF3}$), 75.76 (s, $C^5_{nA\text{-}iPr}$), 74.78 (s, $C^4_{nA\text{-}CF3}$), 73.70 (s, $C^4_{nB\text{-}CF3}$), 53.75 (app broad m, CH), 52.48 (app broad m, CH), 50.93 (s, $C^3_{nA\text{-}iPr}$), 45.93 (s, $C^2_{nA\text{-}iPr}$), 45.64 (q, J = 28.4 Hz, $C^1_{nA\text{-}CF3}$), 44.65 (q, J = 28.4 Hz, $C^1_{nB\text{-}CF3}$), 38.22 (s, $C^6_{nB\text{-}CF3}$), 36.60 (s, $C^6_{nA\text{-}CF3}$), 35.83 (d, J = 7.8 Hz, $CH_{nA\text{-}iPr}$), 34.16 (overlapping s, $CH_{nB\text{-}CF3}$), 34.06 (s, CH_2), 33.50 (s, CH_2), 33.60 (overlapping s, $CH_{nA\text{-}CF3}$), 32.33 (broad m, CH), 31.47 (app d, CH_2), 30.45 (app s, CH), 29.63 (broad s, CH_2), 26.31 (s, $CH_{3;nB\text{-}CF3}$), 25.68 (app broad s, CH_3), 25.08 (s, $CH_{3;nA\text{-}CF3}$), 24.75 (d, J = 6.3 Hz, CH_3), 22.86 (app broad s, CH_3), 22.24 (d, J = 8.5 Hz, CH_3), 21.88 (s, $CH_{3;nB\text{-}CF3}$), 21.25 (d, J = 6.3 Hz, CH_3), 20.77 (s, $CH_{3;nA\text{-}CF3}$), 18.66 (s, $CH_{3;nA\text{-}iPr}$), 17.32 (s, $CH_{3;nA\text{-}iPr}$). $^{31}P\{^1H\}$ NMR (162 MHz, cyclohexane-d_{12}) δ 93.92 (broad s, 3P, overlapping nA-CF_3, nB-CF_3, nA-^{i}Pr), 80.28 (broad s, 3P, overlapping nA-CF_3, nB-CF_3, nA-^{i}Pr). ^{19}F NMR (376 MHz, cyclohexane-d_{12}) δ -56.76 (s, 3F, nB-^{i}Pr), -58.38 (s, 3F, nA-^{i}Pr), -74.18 (d, J = 8.4 Hz, 3F, nB-CF_3), -75.27 (d, J = 7.9 Hz, 3F, nA-CF_3).

Facial Assignment of Cobalt Complexes

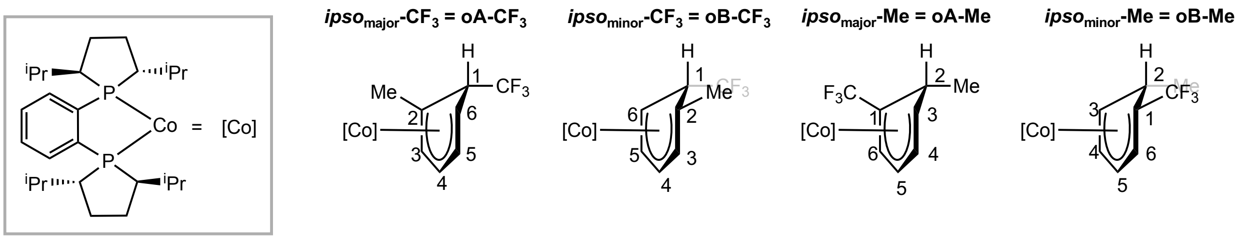

Preparation of Co1-o. This compound was prepared using General Procedure B using 0.022 mmol of (R,R)-(^{iPr}DuPhos)Co(η^3-C_3H_5). A deep red solution formed that contained 89% yield of

(*R,R*)-(^{iPr}DuPhos)Co(η^5-$C_6H_5CF_3Me$), where 70% yield was *ipso*-CF_3 isomers, 19% *ipso*-alkyl isomers, and 11% mass balance unaccounted for relative to 1,3,5-tris(trifluoromethyl)benzene after 24 h. Over the course of 5 days, *ipso*-CF_3 yield increased to 85% and *ipso*-alkyl yield decreased to 15%. Using General Procedure A with 0.0180 mmol of (*R,R*)-(^{iPr}DuPhos)Co(η^3-C_3H_5), a deep red solution formed identified as 75% yield of **Co1-n** that was characterized by ^{1}H, ^{13}C{^{1}H}, ^{19}F, and ^{31}P{^{1}H} NMR spectroscopy. **Co1-n** decomposition was observed when subjected to general procedure C for isolation. ***Ipso*$_{major}$-CF_3**, ***ipso*$_{minor}$-CF_3**, ***ipso*$_{major}$-Me** and ***ipso*$_{minor}$-Me** are denoted **oA-CF_3**, **oB-CF_3**, **oA-Me** and **oB-Me,** respectively. **oA-CF_3**, **oB-CF_3**, and **oA-Me** resonances were assigned using ^{1}H, ^{13}C{^{1}H}, ^{19}F, and ^{31}P{^{1}H} NMR spectroscopy. **oB-Me** resonances were assigned by ^{19}F NMR spectroscopy. ^{1}H NMR (400 MHz, cyclohexane-*d*$_{12}$) δ 7.61 (m, 6H, ^{iPr}DuPhos Ar + overlapping solvent), 7.21 (m, 6H, ^{iPr}DuPhos Ar + overlapping solvent), 5.34 (d, *J* = 5.5 Hz, 1H, C^6H_{oA-Me}), 5.19 (app s, 1H, C^4H_{oB-CF3}), 5.06 (app d, 2H, C^3H_{oA-CF3}+C^5H_{oB-CF3}), 4.98 (broad m, 1H, C^4H_{oA-Me}), 4.86 (t, *J* = 5.8 Hz, 1H, C^5H_{oA-CF3}), 4.81 (app s, 2H, C^5H_{oA-Me} + C^4H_{oA-CF3}), 4.37 (d, *J* = 5.3 Hz, 1H, C^3H_{oB-CF3}), 3.69 (app m, 1H, C^3H_{oA-Me}), 3.27 (t, *J* = 6.2 Hz, 1H, C^6H_{oA-CF3}), 3.16 (m, 1H, C^1H_{oB-CF3}), 2.98 (m, 1H, C^1H_{oA-CF3}), 2.62-2.59 (m, 2H, C^2H_{oA-Me} + C^6H_{oB-CF3}), 2.33- 2.15 (m, 12H, overlapping CH_2 + CH), 2.14- 2.04 (m, 8H, overlapping CH_2 + CH), 1.90-1.74 (m, 8H, overlapping CH_2 + CH), 1.70 (s, 3H, $CH_{3;oB-CF3}$), 1.67-1.67-1.59 (m, 10H, overlapping CH + CH_2), 1.59 (s, 3H, $CH_{3;oA-CF3}$), 1.57-1.46 (m, 10H, overlapping CH + CH_2), 1.03 (broad m, 9H, CH_3), 0.98 (broad m, 15H, CH_3), 0.77 (broad m, 15H, CH_3), 0.69 (m, 9H, CH_3), 0.55 (broad s, 3H, $CH_{3;oA-Me}$), 0.58- 0.46 (broad m, 24H, CH_3). ^{13}C{^{1}H} NMR (101 MHz, cyclohexane-*d*$_{12}$) δ 130.98 (s, ^{iPr}DuPhos Ar), 128.73 (s, ^{iPr}DuPhos Ar), 122.01 (app broad m, $CF_{3;oA-CF3}$), 96.55 (s, C^3_{oB-CF3}), 95.78 (s, C^3_{oA-CF3} or C^4_{oB-CF3}), 87.83 (q, *J* = 4.5 Hz, C^6_{oA-Me}), 87.56 (s, C^4_{oA-Me}), 86.30 (s, C^5_{oA-CF3}), 84.88 (s, C^3_{oA-CF3} or C^4_{oB-CF3}), 76.94 (s, C^5_{oA-Me} or C^4_{oA-CF3}), 75.16 (s, C^5_{oA-Me} or C^4_{oA-CF3}), 72.70 (s, C^4_{oB-CF3}), 69.39 (s, C^2_{oB-CF3}), 63.48 (s, C^2_{oA-CF3}), 61.17 (app broad m, C^1_{oA-Me}), 54.64 (s, C^3_{oA-Me}), 52.87 (d, *J* = 18.3 Hz, CH), 49.15 (app m, C^1_{oA-CF3}), 48.64 (app m, C^1_{oB-CF3}), 39.68 (s, C^6_{oA-CF3}), 39.16 (s, C^2_{oA-Me} or C^6_{oB-CF3}), 35.20 (s, C^6_{oB-CF3}), 34.47 (app s, s, C^2_{oA-Me}), 33.77 (broad

app s, CH), 31.73 (m, overlapping CH + CH_2), 25.09 (d, J = 5.5 Hz, CH_3), 24.43 (s, $CH_{3;oA\text{-}CF3}$), 23.91 (s, $CH_{3;oB\text{-}CF3}$), 22.35 (app broad m, CH_3), 21.29 (q, J = 3.5 Hz, $CH_{3;oA\text{-}Me}$). Additional ^{iPr}DuPhos Ar, $CF_{3;oB\text{-}CF3}$, $CF_{3;oA\text{-}Me}$, and ^{iPr}DuPhos CH/CH_2 resonances could not be assigned due to peak broadening and poor HMBC/HSQC resolution. $^{31}P\{^1H\}$ NMR (162 MHz, cyclohexane-d_{12}) δ 95.64 (broad s, 3P, overlapping oA-CF_3, oB-CF_3, oA-Me), 79.97 (broad s, 3P, overlapping oA-CF_3, oB-CF_3, oA-Me). ^{19}F NMR (376 MHz, cyclohexane-d_{12}) δ -57.70 (s, 3F, oB-Me), -58.92 (s, 3F, oA-Me), -75.63 (d, J = 8.5 Hz, 3F, oB-CF_3), -76.60 (d, J = 7.9 Hz, 3F, oA-CF_3).

i. Variable Temperature NMR Spectroscopy

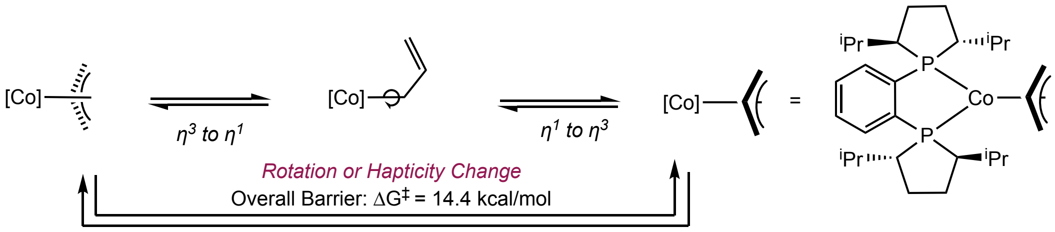

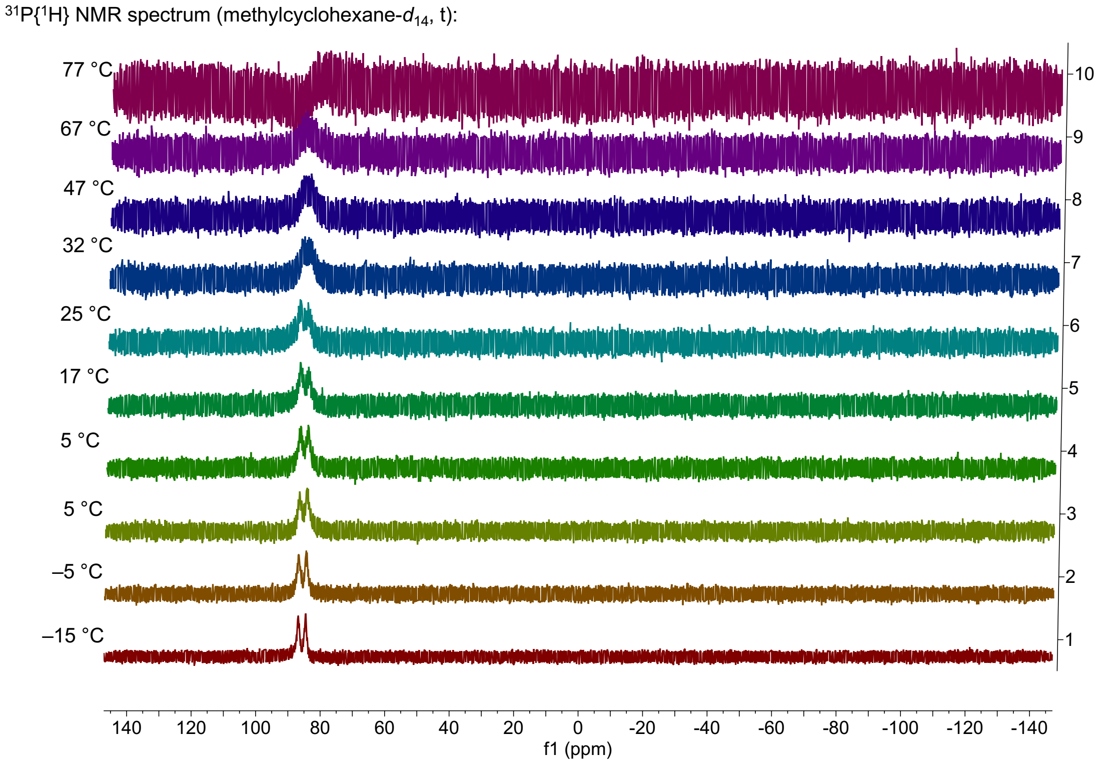

Figure S1. Variable Temperature $^{31}P\{^1H\}$ NMR spectra (202 MHz, methylcyclohexane-d_{14}) of **Co1**.

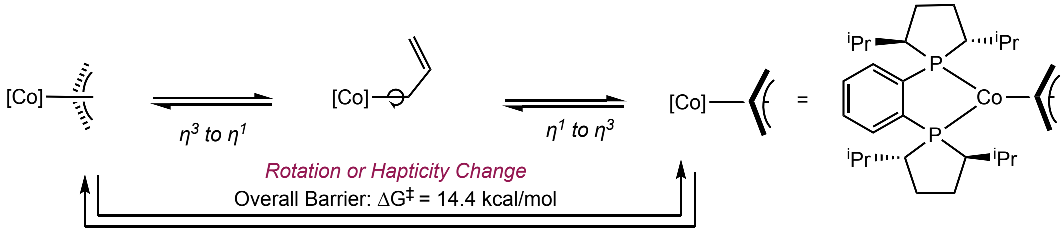

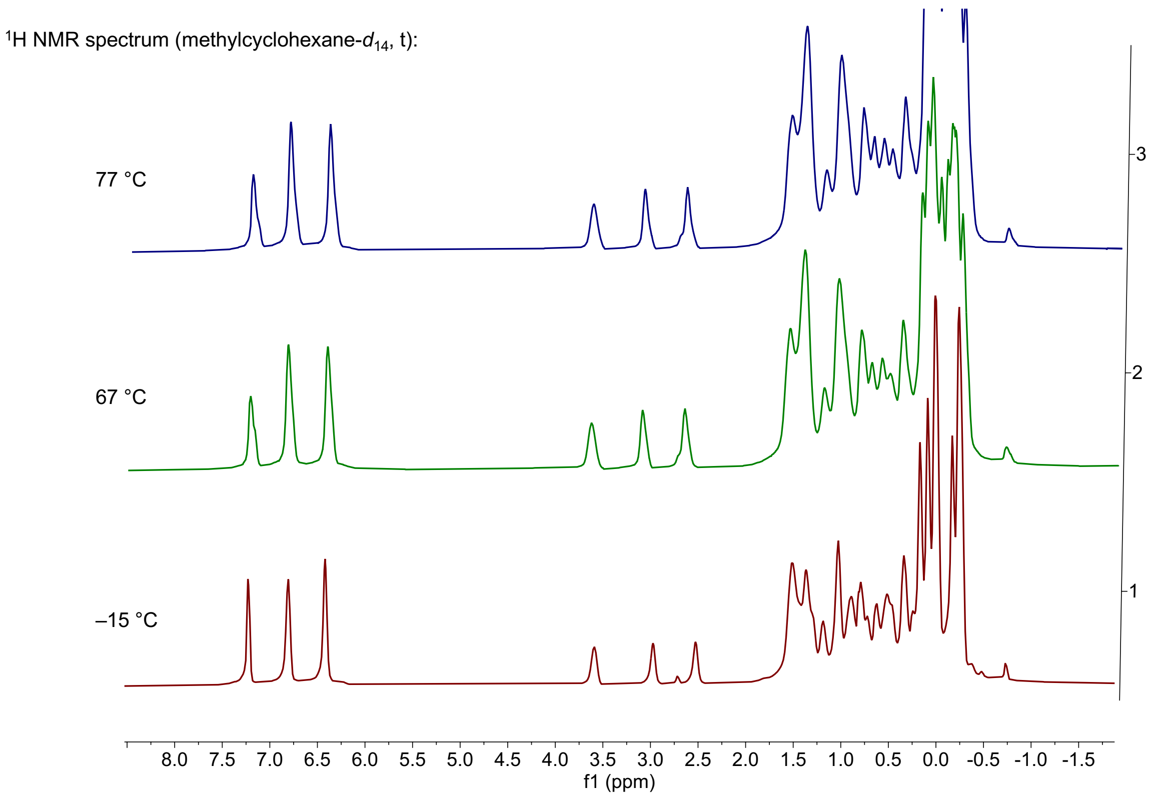

Figure S2. Variable Temperature ^{1}H NMR spectra (500 MHz, methylcyclohexane-d_{14}) of **Co1**.

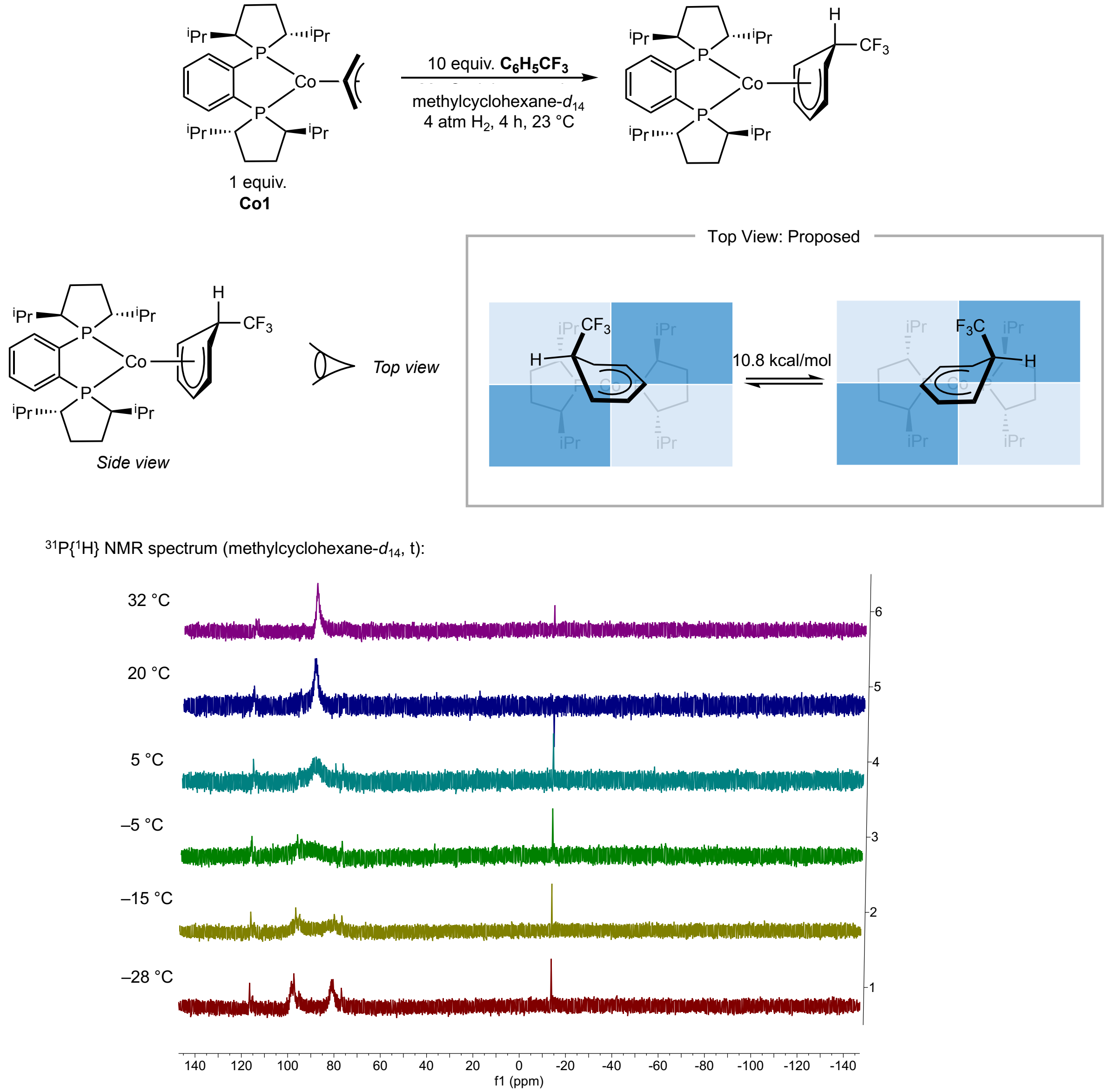

Figure S3. Variable Temperature $^{31}P\{^{1}H\}$ NMR spectra (202 MHz, methylcyclohexane-d_{14}) of **Co1-a**.

ii. Intramolecular Isomerization Data

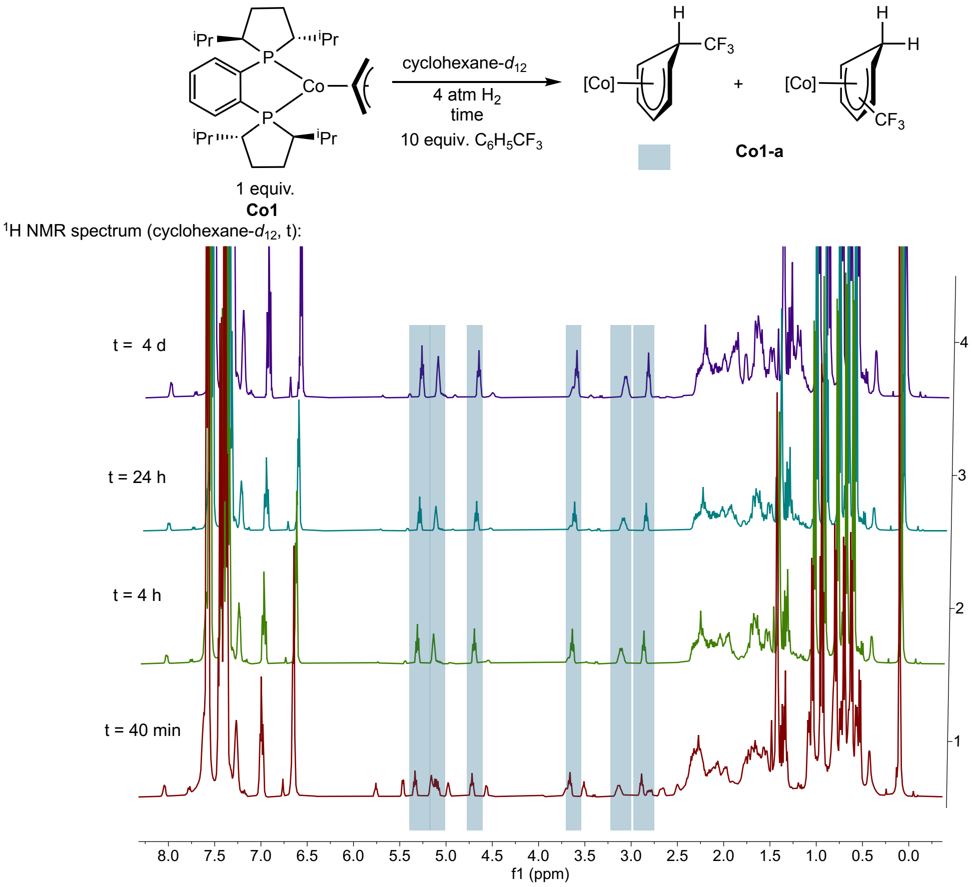

Figure S4. ^{1}H NMR spectra (400 MHz, cyclohexane-d_{12}, 23 °C) of insertion reactions of **Co1** with α,α,α,-trifluoromethyl benzene as a function of time.

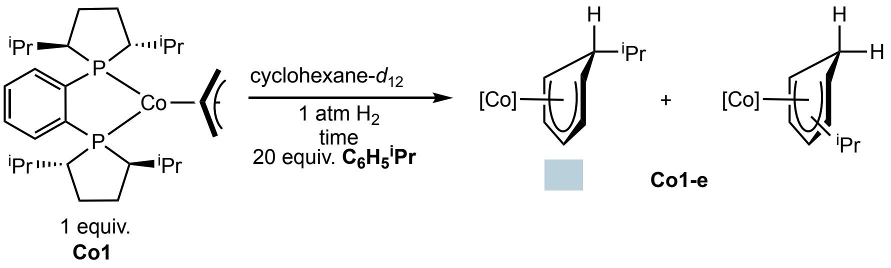

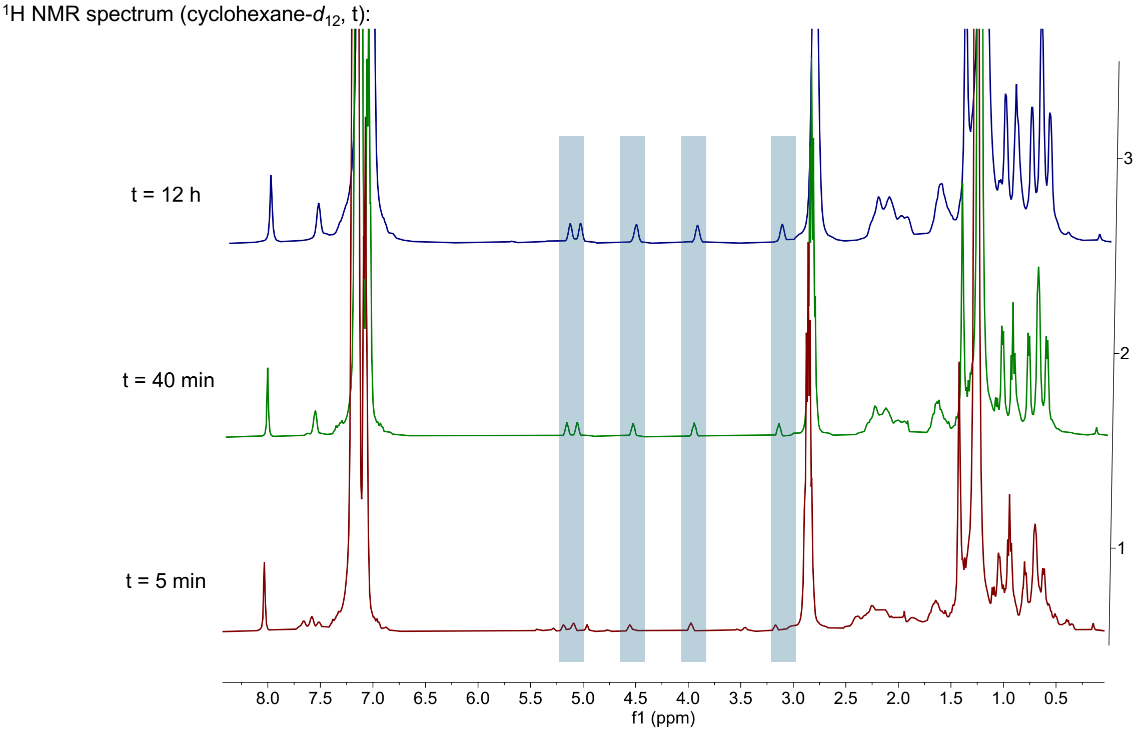

Figure S5. ^{1}H NMR spectra (400 MHz, cyclohexane-d_{12}, 23 °C) of insertion reactions of **Co1** with isopropyl benzene as function of time.

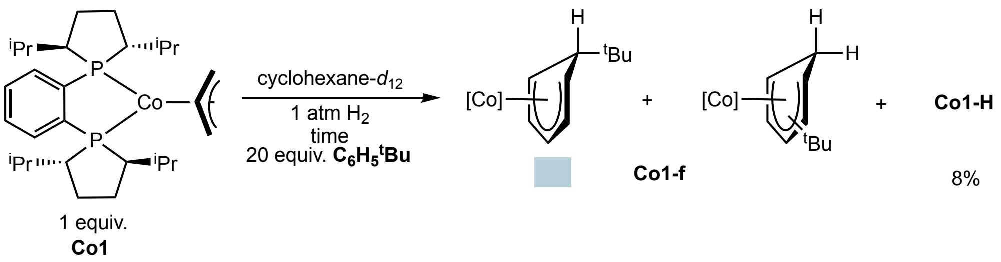

^{1}H NMR spectrum (cyclohexane-d_{12}, t):

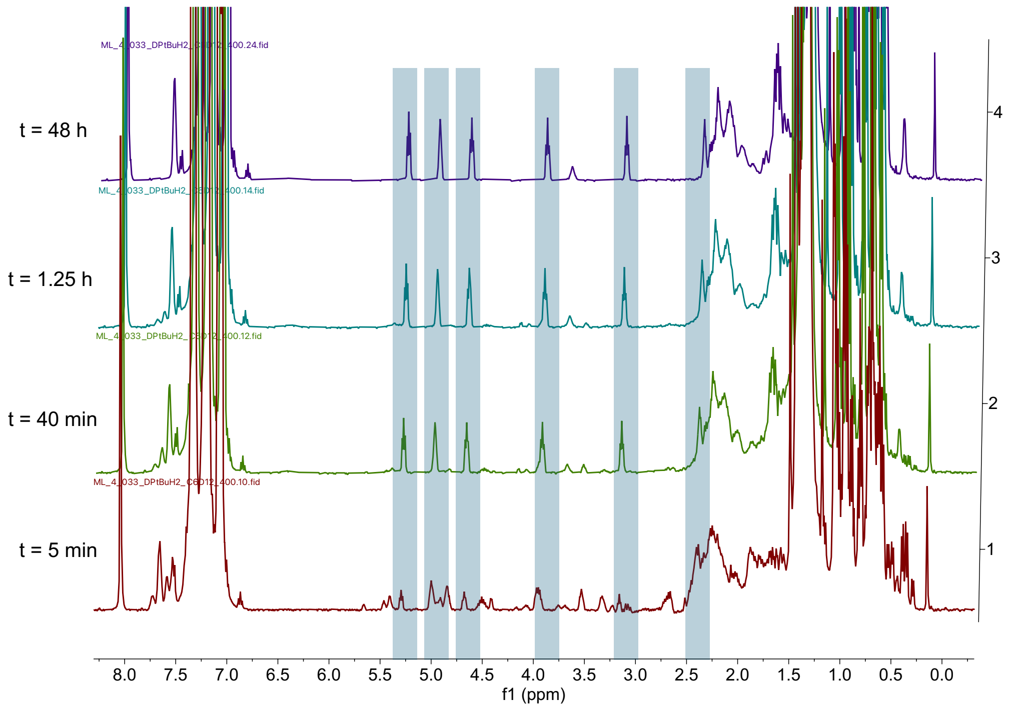

Figure S6. ^{1}H NMR spectra (400 MHz, cyclohexane-d_{12}, 23 °C) of insertion reactions of **Co1** with tert-butyl benzene as a function of time.

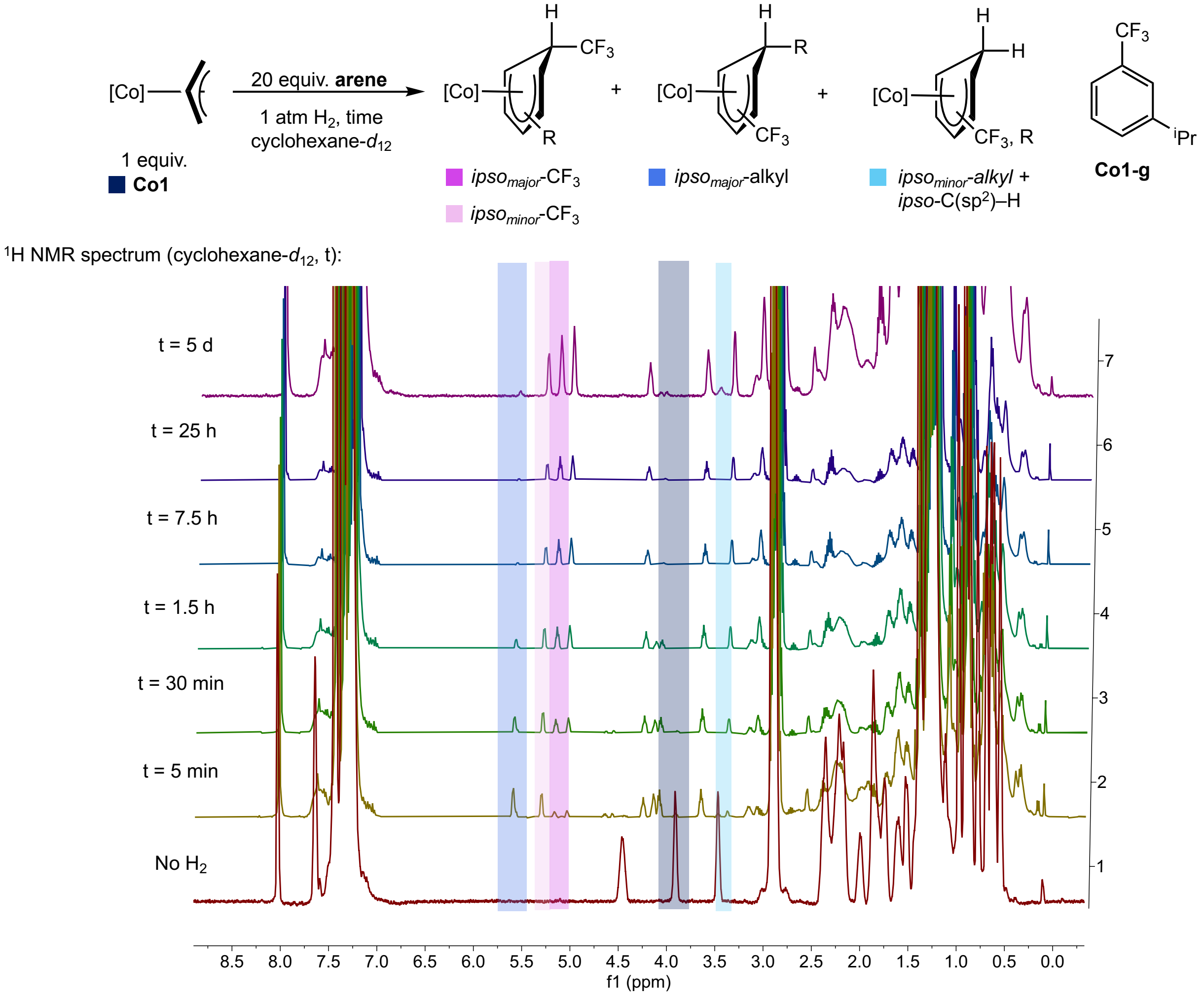

Figure S7. ^{1}H NMR spectra (400 MHz, cyclohexane-d_{12}, 23 °C) of insertion reactions of **Co1** with 1-isopropyl-3-(trifluoromethyl)benzene as a function of time.

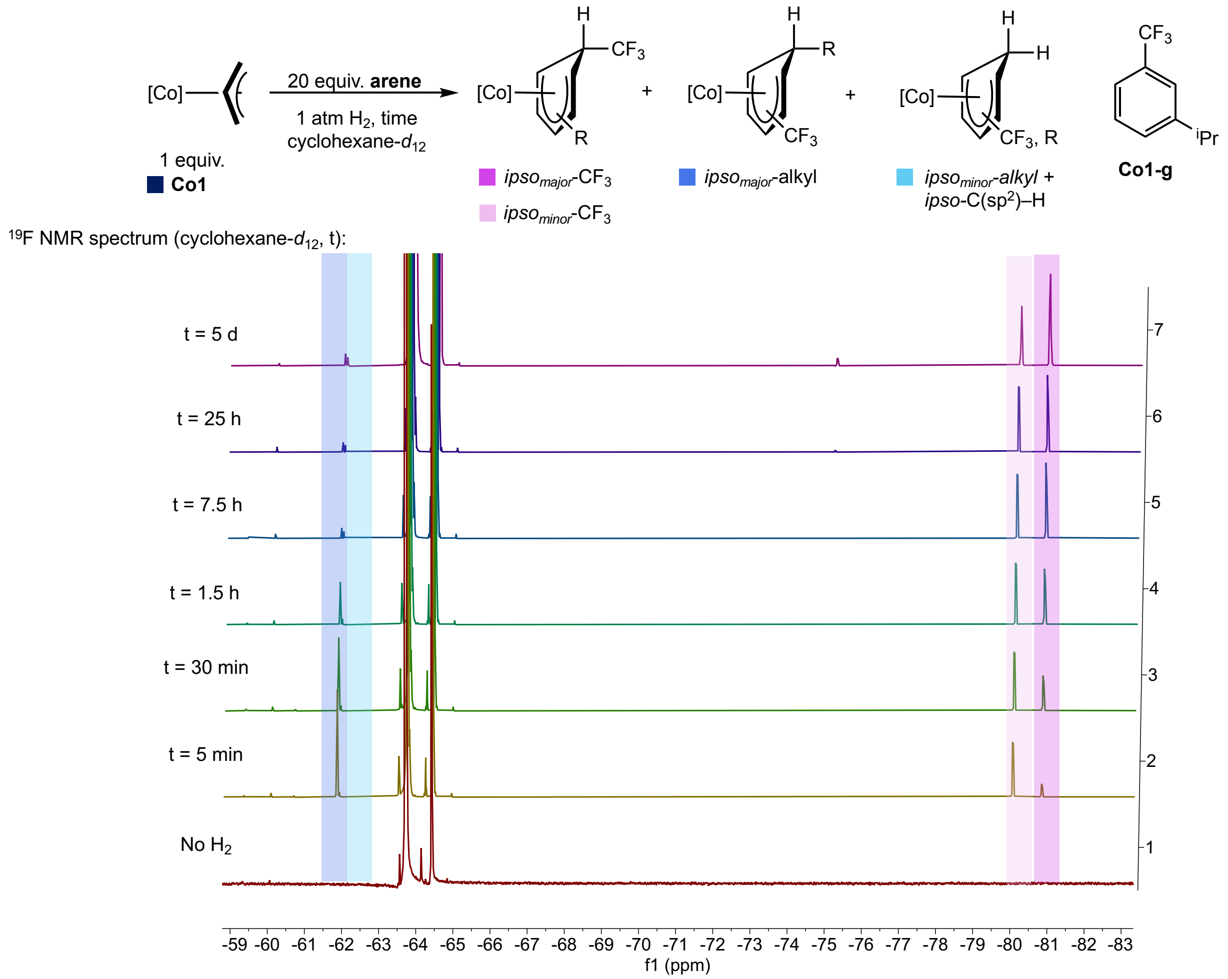

Figure S8. ^{19}F NMR spectra (376 MHz, cyclohexane-d_{12}, 23 °C) of insertion reactions of **Co1** with 1-isopropyl-3-(trifluoromethyl)benzene as a function of time.

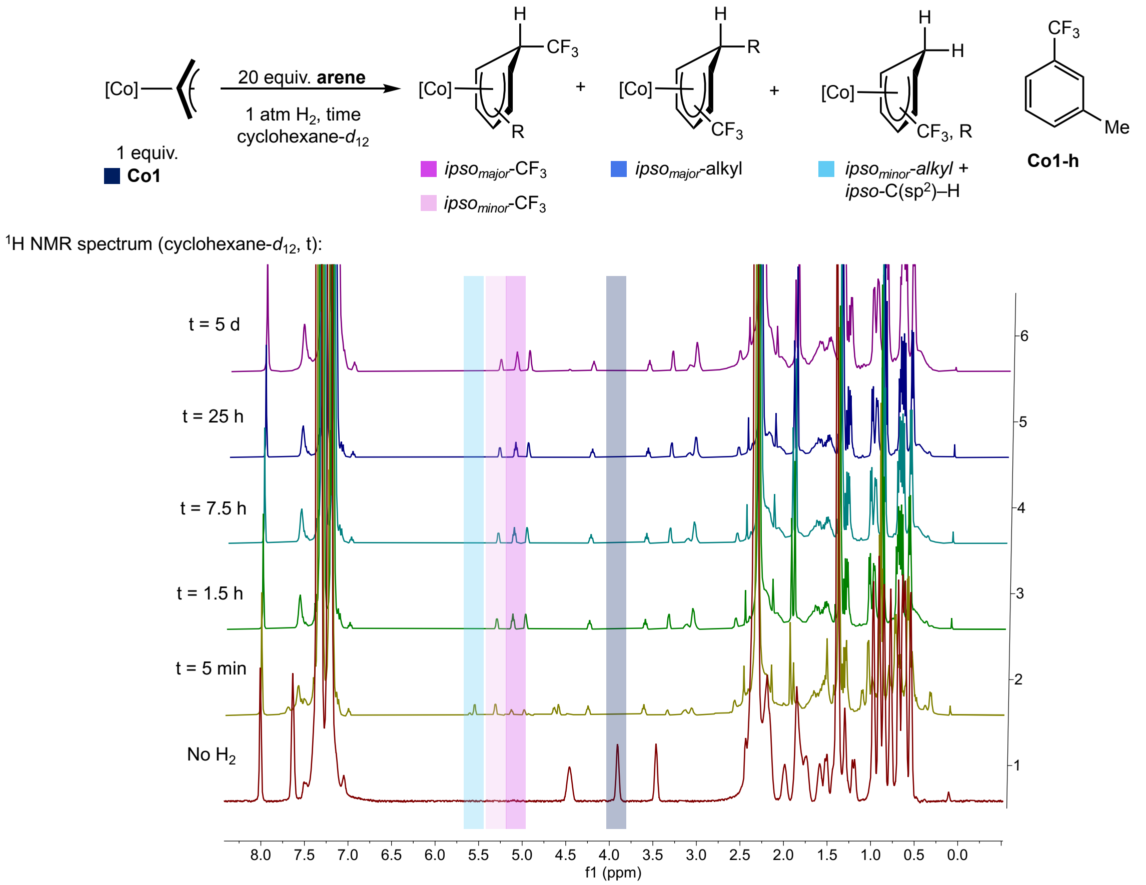

Figure S9. ^{1}H NMR spectra (400 MHz, cyclohexane-d_{12}, 23 °C) of insertion reactions of **Co1** with 1-methyl-3-(trifluoromethyl)benzene as a function of time.

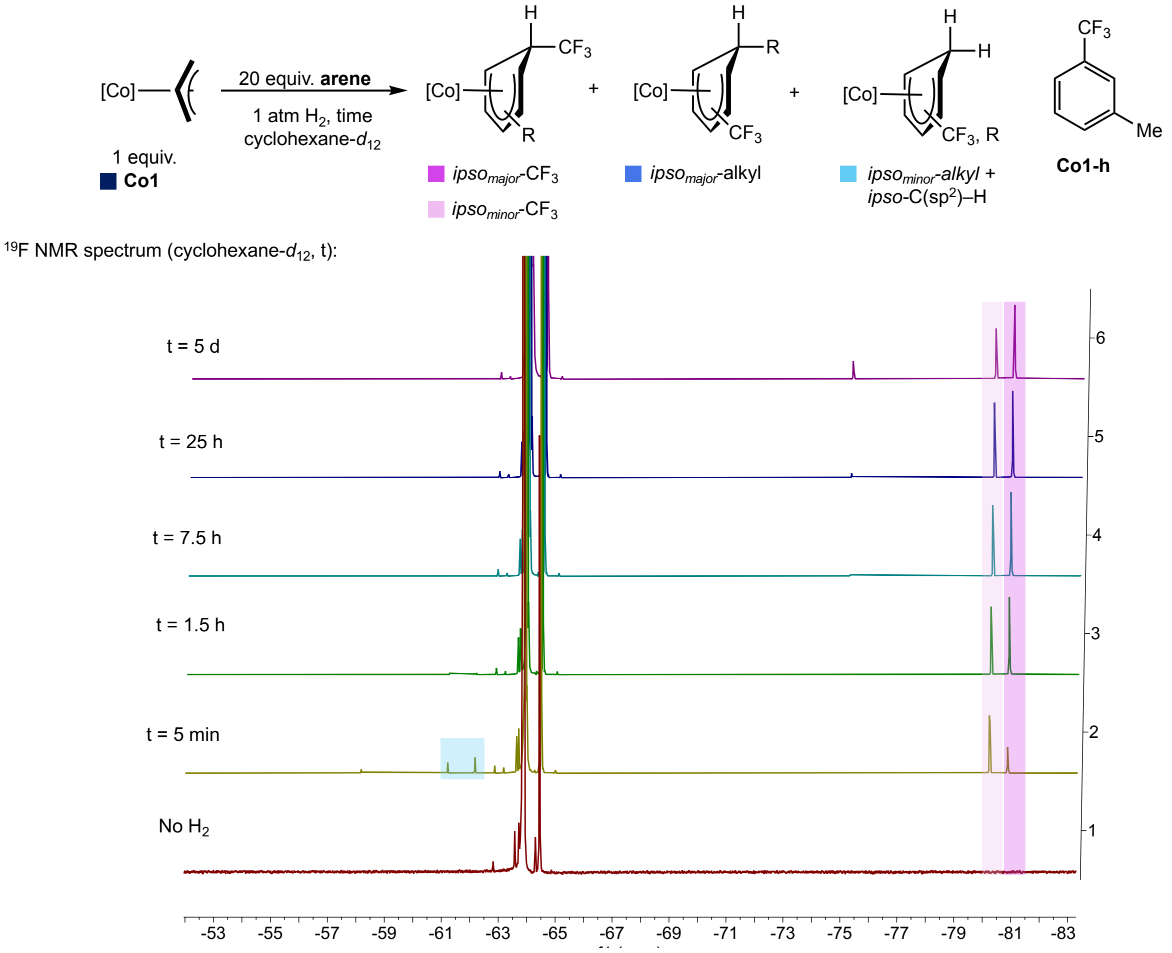

Figure S10. ^{19}F NMR spectra (376 MHz, cyclohexane-d_{12}, 23 °C) of insertion reactions of **Co1** with 1-methyl-3-(trifluoromethyl)benzene as a function of time.

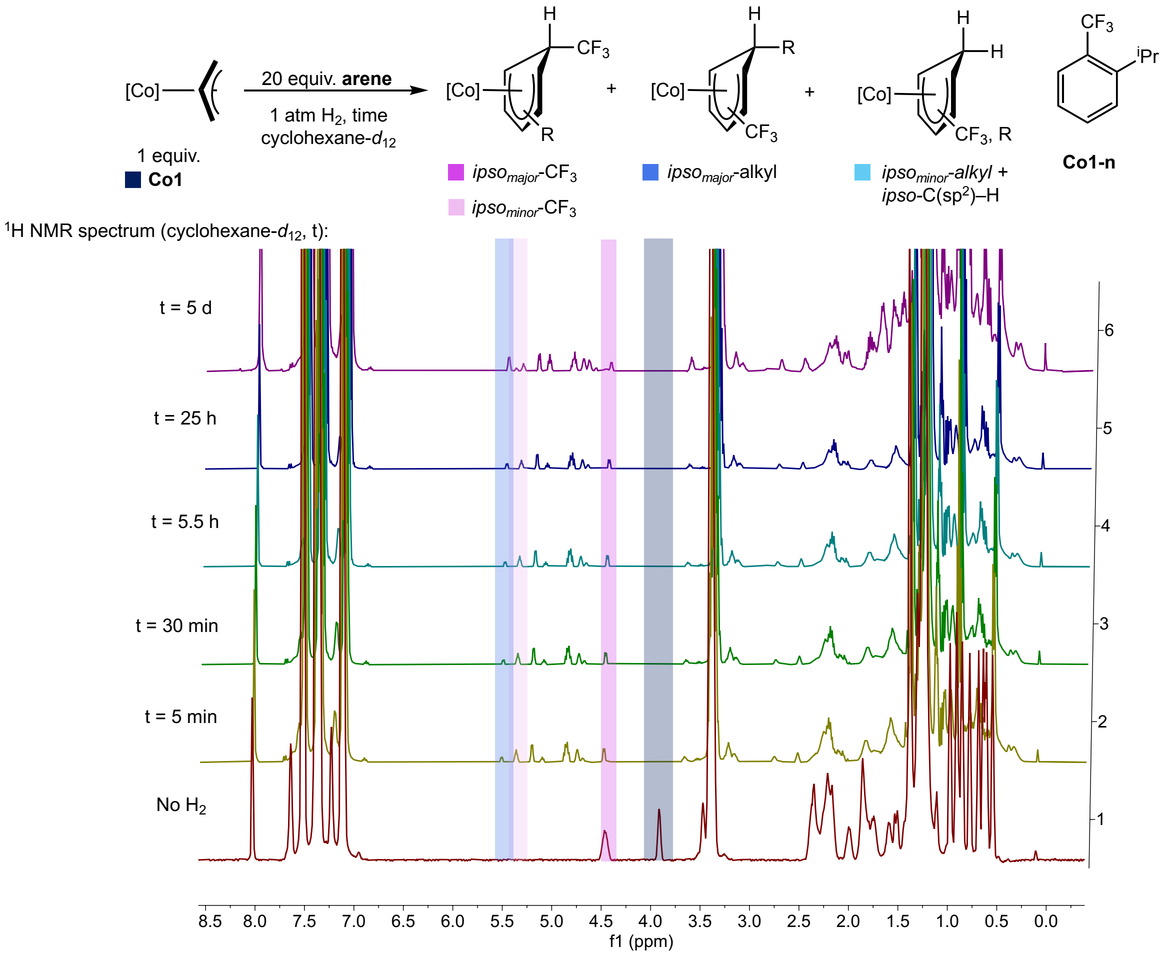

Figure S11. ^{1}H NMR spectra (400 MHz, cyclohexane-d_{12}, 23 °C) of insertion reactions of **Co1** with 1-isopropyl-2-(trifluoromethyl)benzene as a function of time.

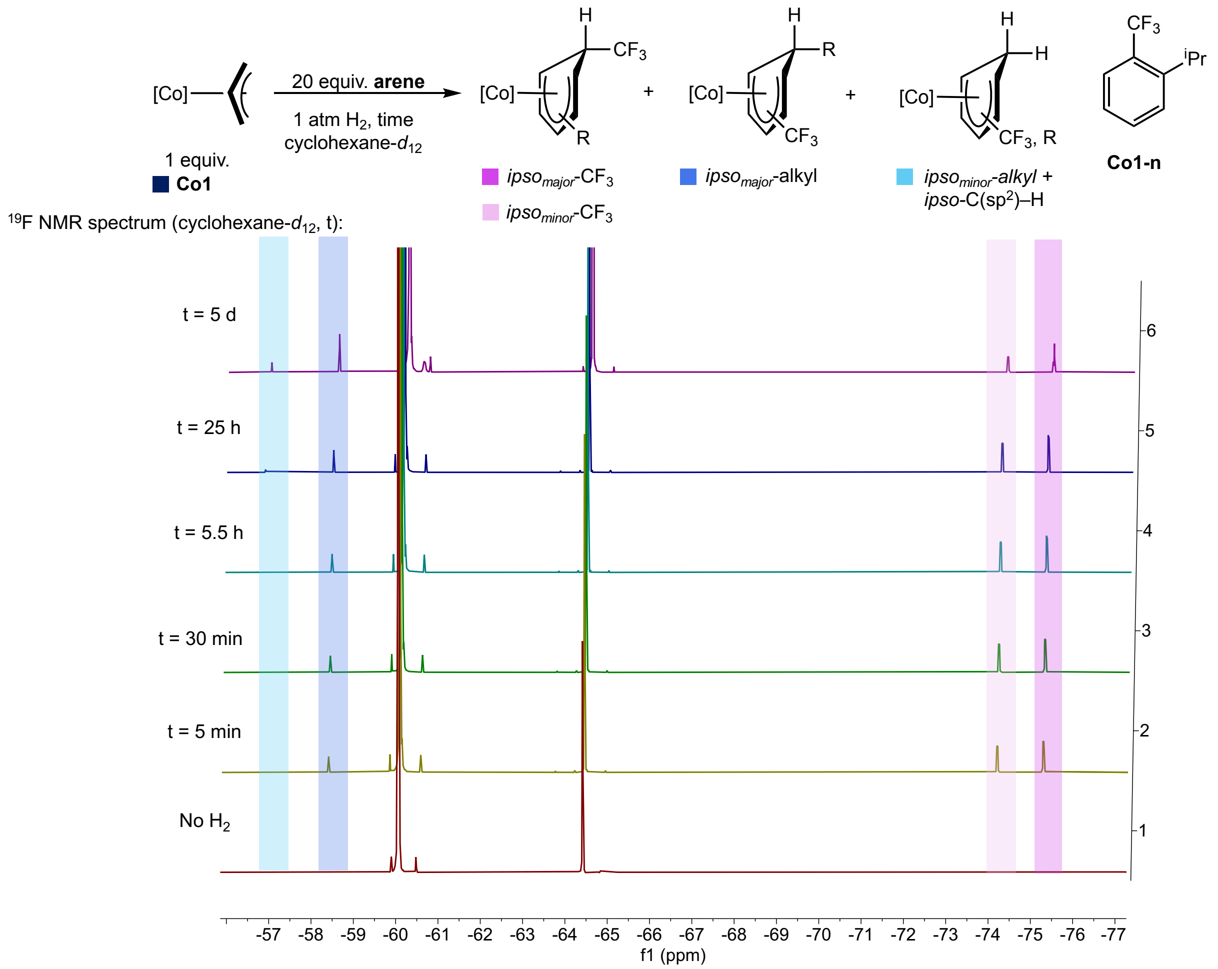

Figure S12. ^{19}F NMR spectra (376 MHz, cyclohexane-d_{12}, 23 °C) of insertion reactions of **Co1** with 1-isopropyl-2-(trifluoromethyl)benzene as a function of time.

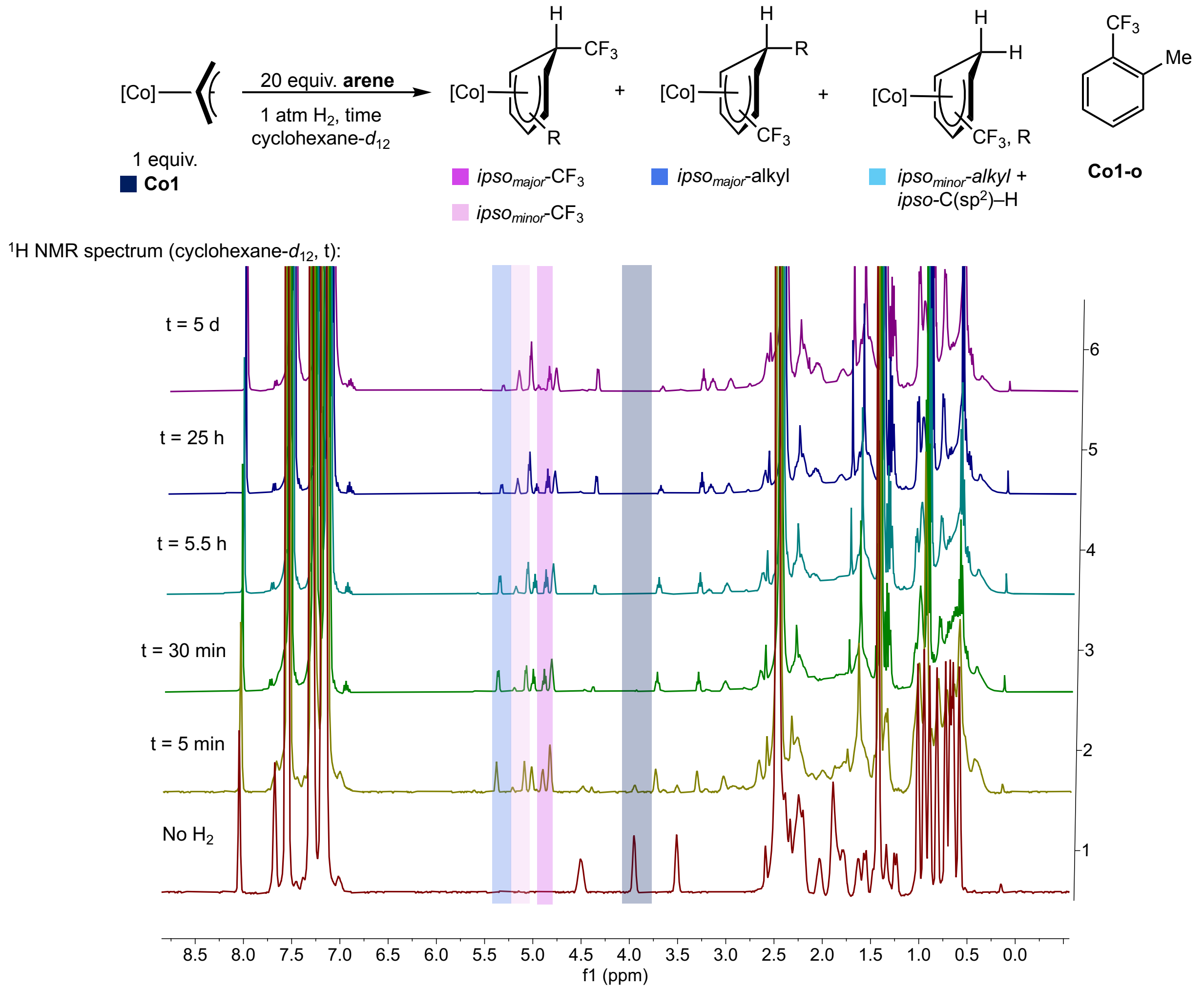

Figure S13. ^{1}H NMR spectra (400 MHz, cyclohexane-d_{12}, 23 °C) of insertion reactions of **Co1** with 1-methyl-2-(trifluoromethyl)benzene as a function of time.

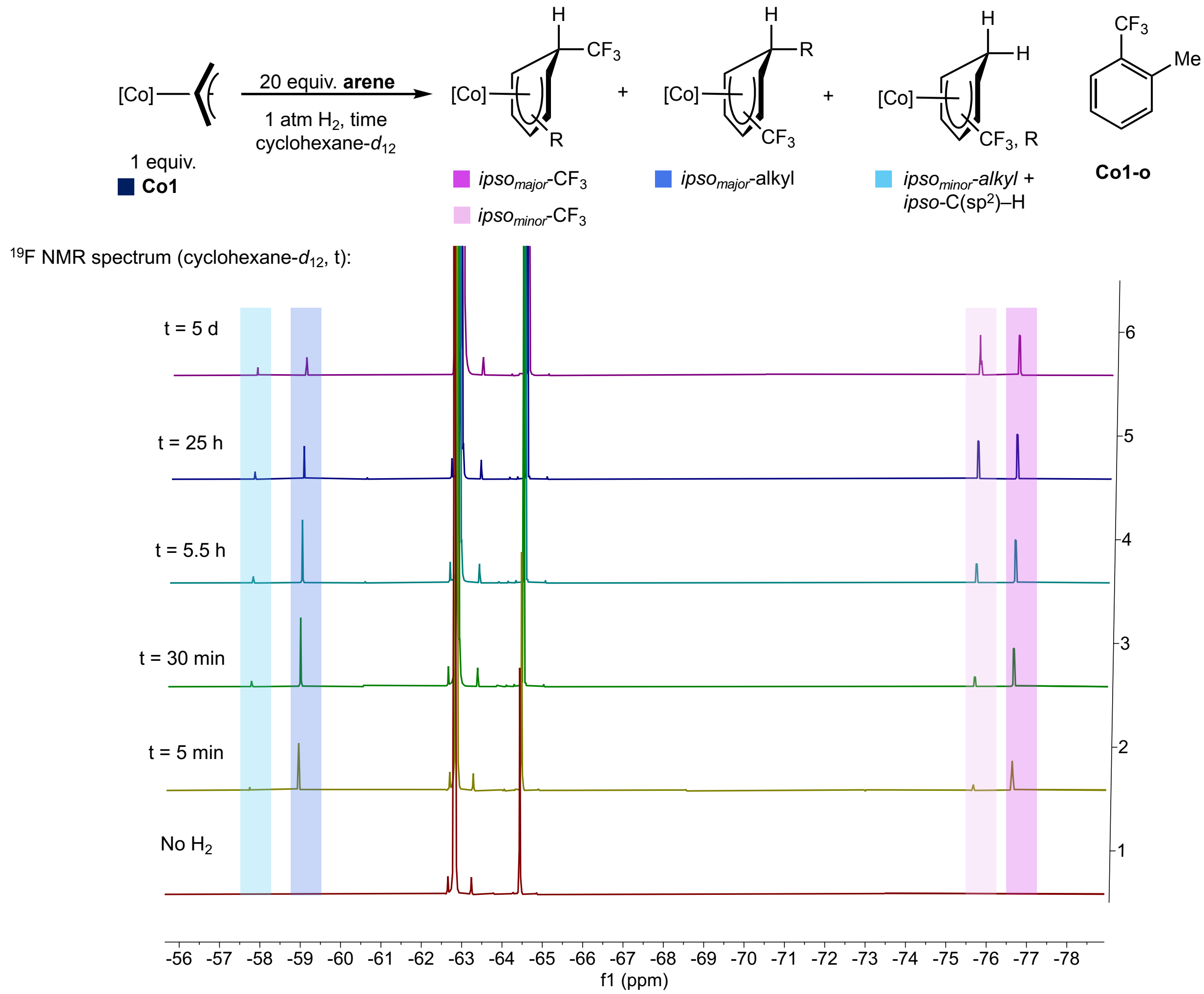

Figure S14. ^{19}F NMR spectra (376 MHz, cyclohexane-d_{12}, 23 °C) of insertion reactions of **Co1** with 1-methyl-2-(trifluoromethyl)benzene as a function of time.

Co1-a

Time	*ipso* (% yield)	*ortho* (% yield)	*meta* (% yield)
40 min	45	32	23
4 h	89	6	5
24 h	89	6	5
4 d	89	6	5

Table S1. % yield over time associated with **Co1-a** in Figure S4 determined through relative integration of ^{1}H NMR spectra.

**Co1-a**					
Time	*ipso* (% yield)	*ortho* (% yield)	*meta* (% yield)	**Co1-H** (% yield)	Mass balance lost (% yield)
40 min	41	29	21	6	3
4 h	80	6	5	7	3
24 h	84	6	4	6	-
4 d	82	6	4	8	-

Table S2. % yield over time associated with **Co1-a** in Figure S4 and Scheme 6 determined through integration of ^{1}H NMR spectra to HMDSO internal standard.

Cobalt complex	*ipso* (% yield)	**[Co]**[a] + Mass balance lost (% yield)
**Co2-a**	72	28
**Co3-a**	79	21
**Co4-a**[b]	40	60
**Co4-a**	35	65

Table S3. % yield over time associated with **Co2-a**, **Co3-a**, and **Co4-a** in Scheme 5 determined through integration of ^{1}H NMR spectra to HMDSO internal standard. [a][Co] represents overlapping resonances associated with insertion isomers, broad cobalt hydride resonances, as well as mass balance unaccounted for. [b]Within one hour of data collection.

**Co1-e**			
Time	*ipso* (% yield)	other (% yield)	**[Co]**[a] + Mass balance lost (% yield)
5 min	34	-	66
40 min	88	-	12
3 h	88	-	12
12 h	86	-	14

Table S4. % yield over time associated with **Co1-e** in Figure S5. [a][Co] represents overlapping resonances associated with insertion isomers, broad cobalt hydride resonances, as well as mass balance unaccounted for.

Co1-f insertion

Time	ipso (% yield)	other (% yield)	**Co1-H** (% yield)	mass lost (% yield)
5 min	21	-	2	-
40 min	64	28	4	4
1.25 h	69	17	6	8
3 h	83	2	7	8
48 h	84	0	7	9

Table S5. % yield over time associated with **Co1-f** in Figure S6.

Co1-g insertion

Time	***ipso$_{minor}$-CF$_3$*** *(% yield)*	***ipso$_{major}$-CF$_3$*** *(% yield)*	***ipso$_{major}$-alkyl*** *(% yield)*	*C$_{other}$* *(% yield)*	***Co1*** *(% yield)*	*mass lost* *(% yield)*
5 min	32	11	42	0	15	0
2 h	34	43	18	0	6	0
7.5 h	35	59	4	2	0	6
120 h	33	58	3	2	0	6

Table S6. % yield over time associated with **Co1-g** in Scheme 10A and Figures S7 and S8.

Co1-h insertion

Time	***ipso$_{minor}$-CF$_3$*** *(% yield)*	***ipso$_{major}$-CF$_3$*** *(% yield)*	***ipso$_{major}$-alkyl*** *(% yield)*	*C$_{other}$* *(% yield)*	***Co1*** *(% yield)*	*mass lost* *(% yield)*
5 min	33	18	2	11	36	0
1 h	37	47	1	4	11	0
7.5 h	38	61	1.3	0	0	0
120 h	36	61	1	0	0	2

Table S7. % yield over time associated with **Co1-h** in Scheme 10A and Figure S9 and S10.

Co1-n insertion

Time	***ipso$_{major}$-CF$_3$*** *(% yield)*	***ipso$_{minor}$-CF$_3$*** *(% yield)*	***ipso$_{major}$-alkyl*** *(% yield)*	*C$_{other}$* *(% yield)*	***Co1*** *(% yield)*	*mass lost* *(% yield)*
5 min	42	35	14	0	9	0
5.5 h	46	36	17	1	0	0
25 h	45	33	19	2	0	1
120 h	35	18	29	6	0	12

Table S8. % yield over time associated with **Co1-n** in Scheme 10A and Figure S11 and S12.

Co1-o insertion						
Time	***ipso$_{minor}$-CF$_3$*** *(% yield)*	***ipso$_{major}$-CF$_3$*** *(% yield)*	***ipso$_{major}$-alkyl*** *(% yield)*	*C$_{other}$* *(% yield)*	***Co1*** *(% yield)*	*mass lost* *(% yield)*
5 min	36	7	31	2	24	0
5.5 h	41	19	37	3	0	0
25 h	38	32	16	3	0	11
120 h	40	40	9	0	0	11

Table S9. % yield over time associated with **Co1-o** in Scheme 10A and Figure S13 and S14.

iii. Benzene Exchange

A 20 mL scintillation vial was charged with 1 equivalent of bis(phosphine)Co(η^5-C_6H_7) in a 0.05 M benzene-d_6 solution and then added to a J. Young NMR tube followed by addition of 50 μL of 0.25 M 1,3,5-tris(trifluoromethyl)benzene cyclohexane-d_{12} solution. The contents of the tube were analyzed by ^{1}H and ^{13}C{^{1}H} NMR spectroscopy over 48 h.

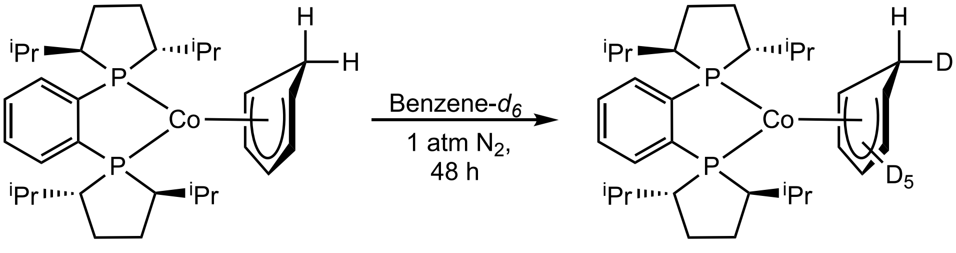

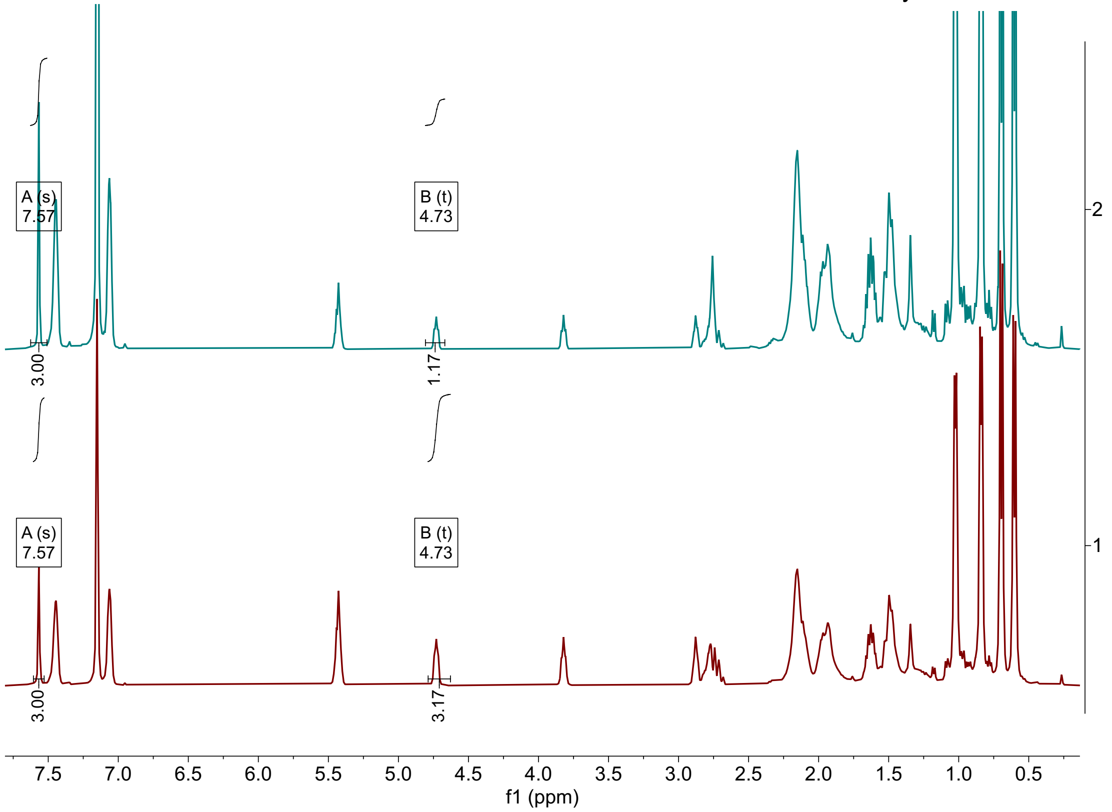

Figure S15. ^{1}H NMR spectra (400 MHz, benzene-d_6, 23 °C) of 68% conversion of 0.0396 mmol **Co1-b** to **Co1-b$_D$** over 48 h.

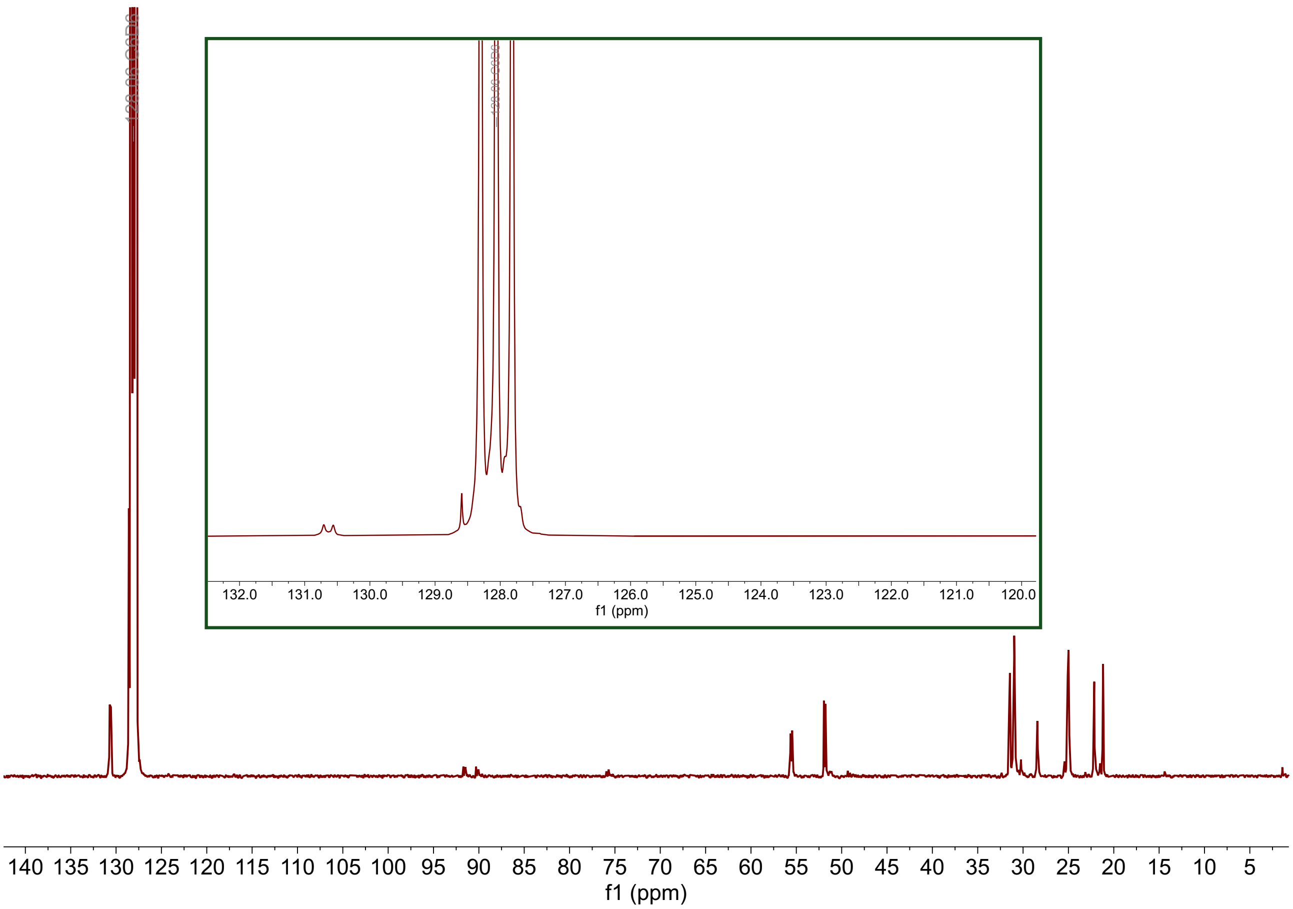

Figure S16. $^{13}C\{^{1}H\}$ NMR spectrum (101 MHz, benzene-d_6, 23 °C) of 68% conversion of 0.0396 mmol **Co1-b** to **Co1-b$_D$** over 48 h showing free benzene.

iv. Oxidation of Co1-a

A 20 mL scintillation vial was charged with 12 mg of **Co1-a** (0.0192 mmol, 1 equiv.) in a 0.02 M Et_2O solution. A separate 20 mL vial was charged with 6.6 mg of $AgSbF_6$ (0.0192 mmol, 1 equiv.) in a 0.02 M Et_2O solution. The $AgSbF_6$ slurry was added dropwise to the **Co1-a** solution and stirred at 23 °C for 15 minutes generating a red and silver precipitate. The slurry was concentrated, brought into THF (~5 mL) and passed through a celite plug. The volatiles were removed under reduced pressure and washed with pentane yielding 61% **Co1-a$^+$** as a red powder. Single-crystals suitable for x-ray diffraction were grown through a layered solution of *o*-

difluorobenzene and pentane. Anal Calcd for $C_{33}H_{50}CoF_9P_2Sb$: C, 46.07; H, 5.86. Found: C, 46.11; H, 5.39. Magnetic Susceptibility (Evans method, THF-d_8, 23 ºC): μ_{eff} = 2.1(6) μ_B. ^{1}H NMR (400 MHz, THF-d_8) δ 17.77 (194 Hz), 10.28 (182 Hz), 3.39 – 3.12 (59 Hz), 2.16 (27 Hz), 1.46 (5.6 Hz), 1.41 (6.8 Hz), 1.26 (25 Hz), 1.12 (15 Hz), 0.87 (57 Hz), 0.51 (35 Hz), 0.08 (12 Hz), -0.42 (13 Hz), -1.03 (20 Hz). ^{19}F NMR (376 MHz, THF-d_8) δ -95.62 (178 Hz).

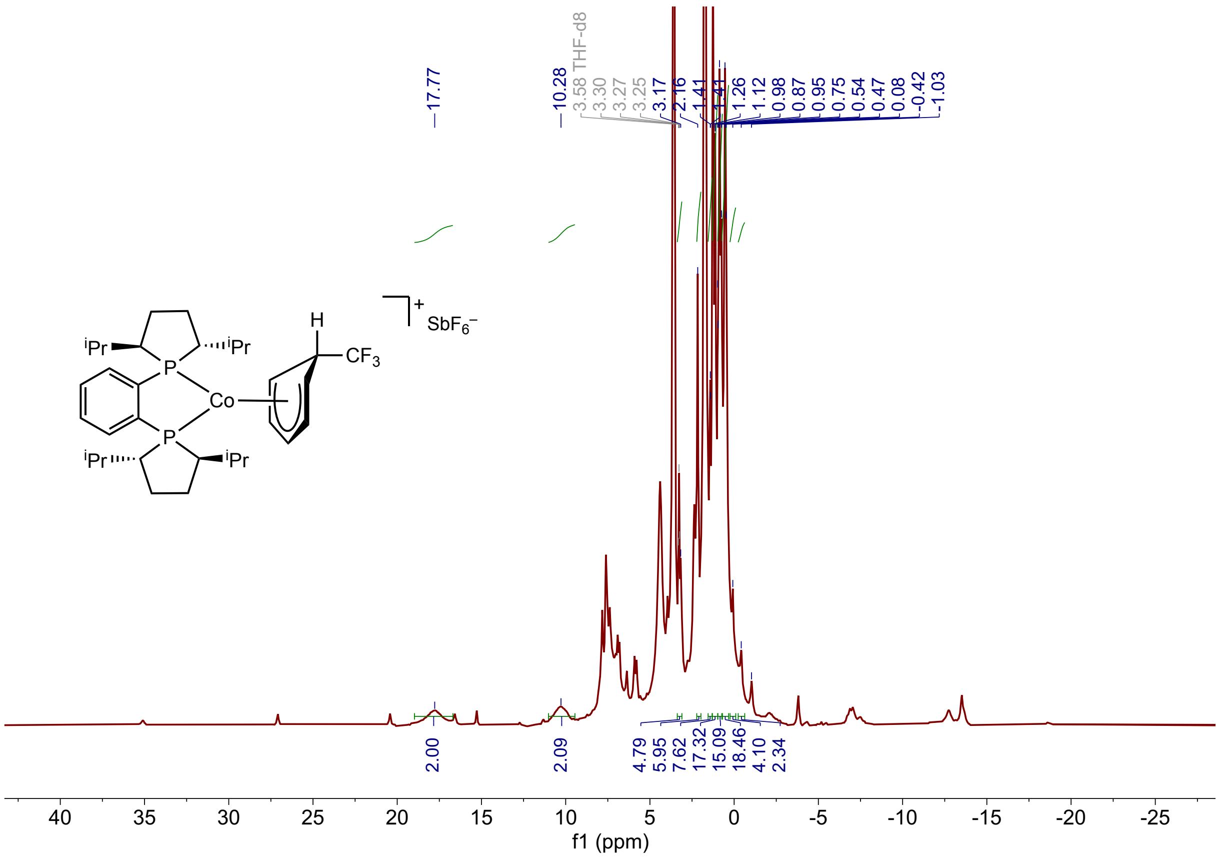

Figure S17. ^{1}H NMR spectrum (400 MHz, THF-d_8, 23 °C) of **Co1-a$^+$**. A minor unidentified paramagnetic compound was also observed.

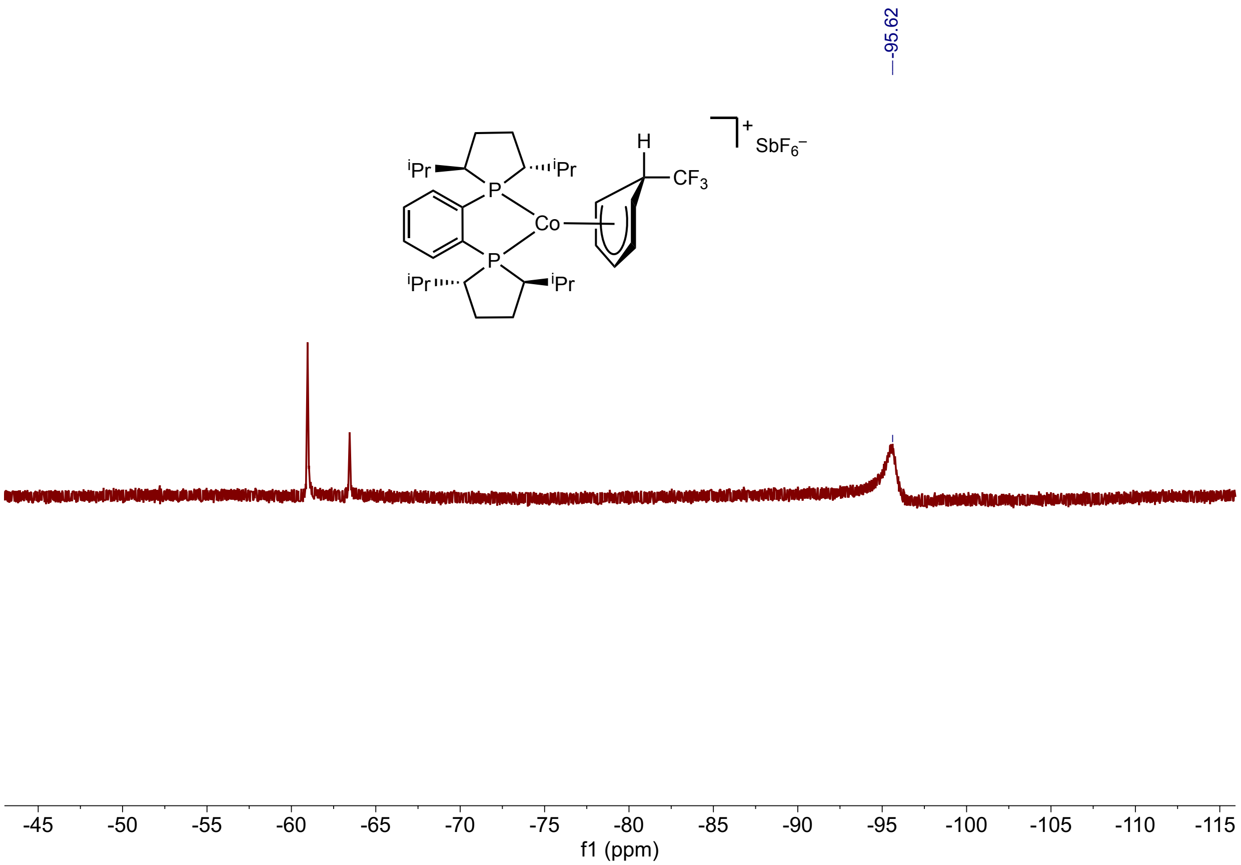

Figure S18. ^{19}F NMR spectrum (376 MHz, THF-d_8, 23 °C) of **Co1-a$^+$**.

IV. NMR Spectroscopic Data of Cobalt Complexes

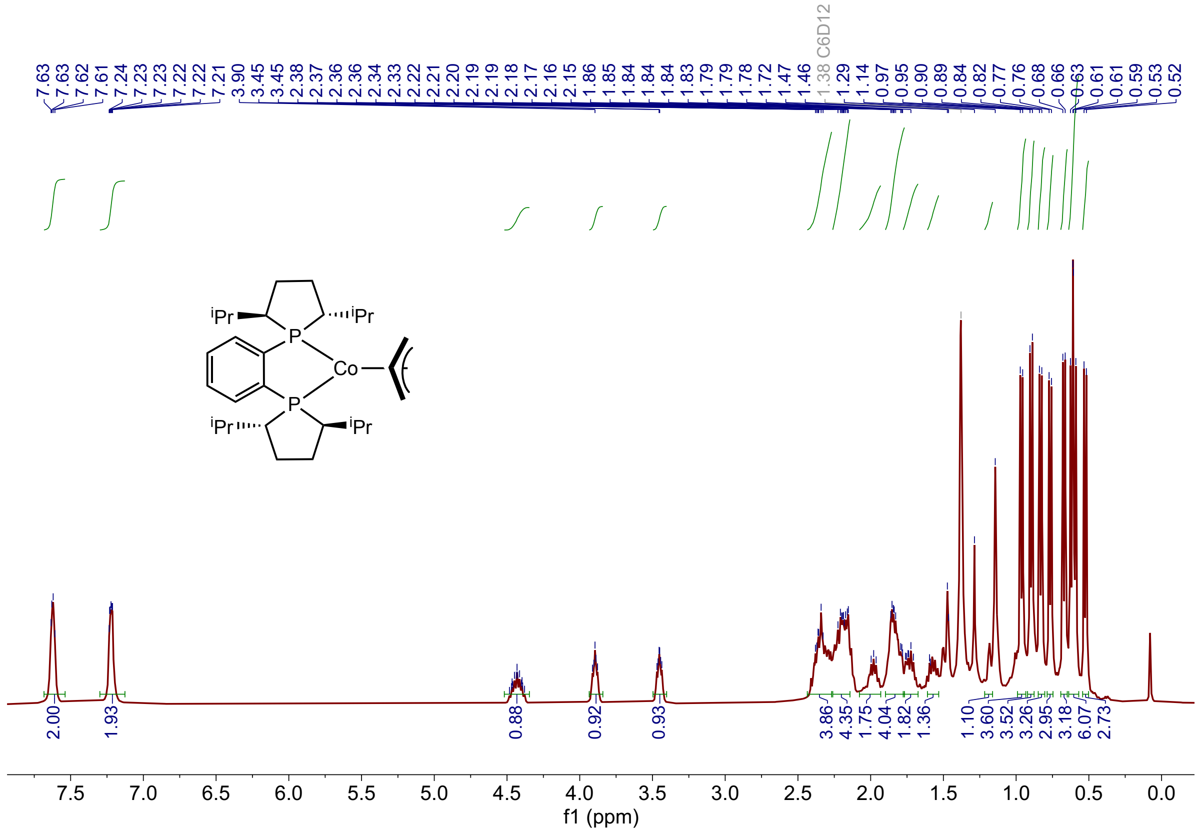

Figure S19. ^{1}H NMR spectrum (400 MHz, cyclohexane-d_{12}, 23 °C) of **Co1**.

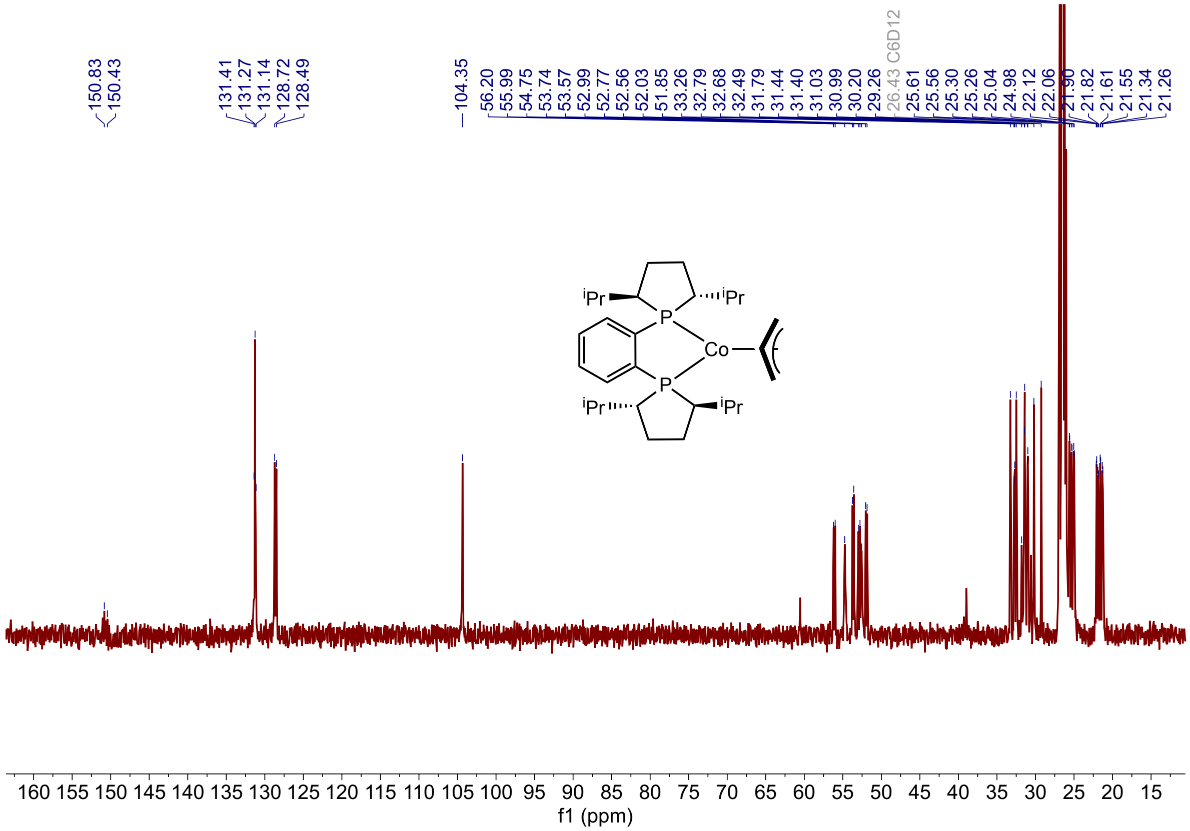

Figure S20. $^{13}C\{^1H\}$ NMR spectrum (101 MHz, cyclohexane-d_{12}, 23 °C) of **Co1**.

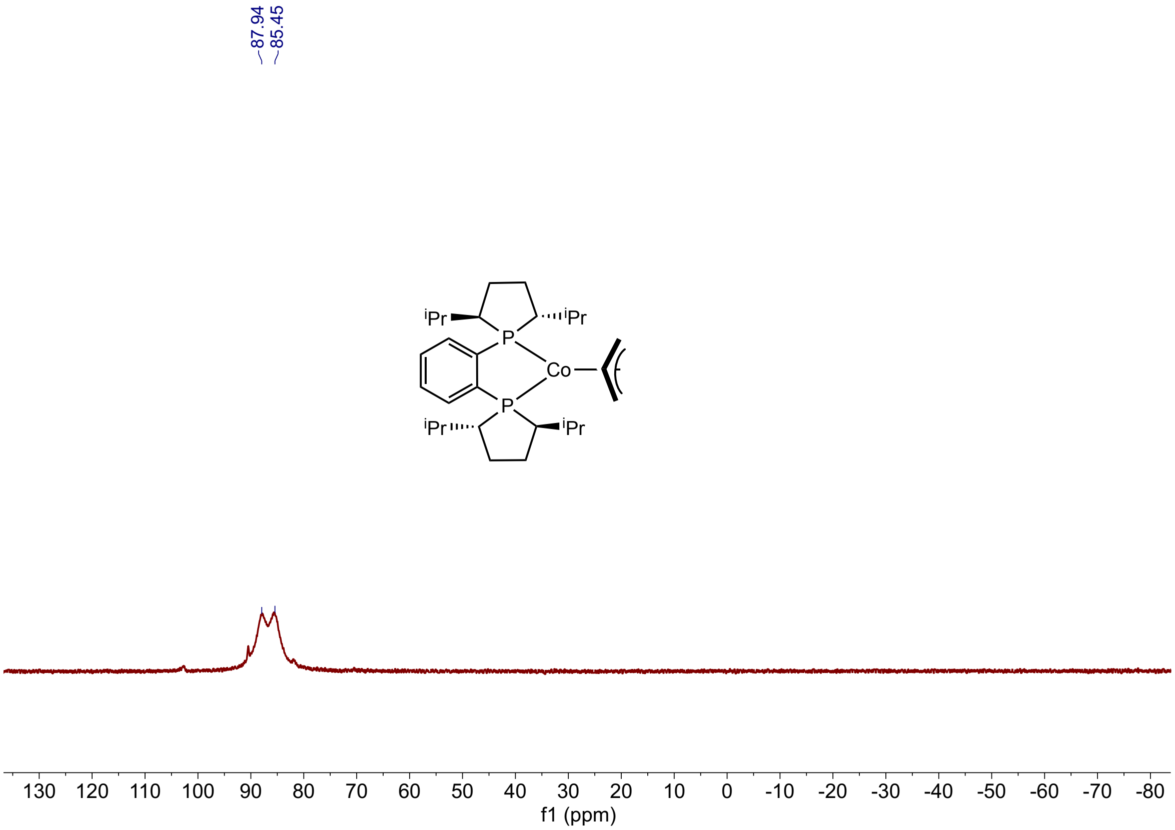

Figure S21. $^{31}P\{^{1}H\}$ NMR spectrum (162 MHz, cyclohexane-d_{12}, 23 °C) of **Co1**.

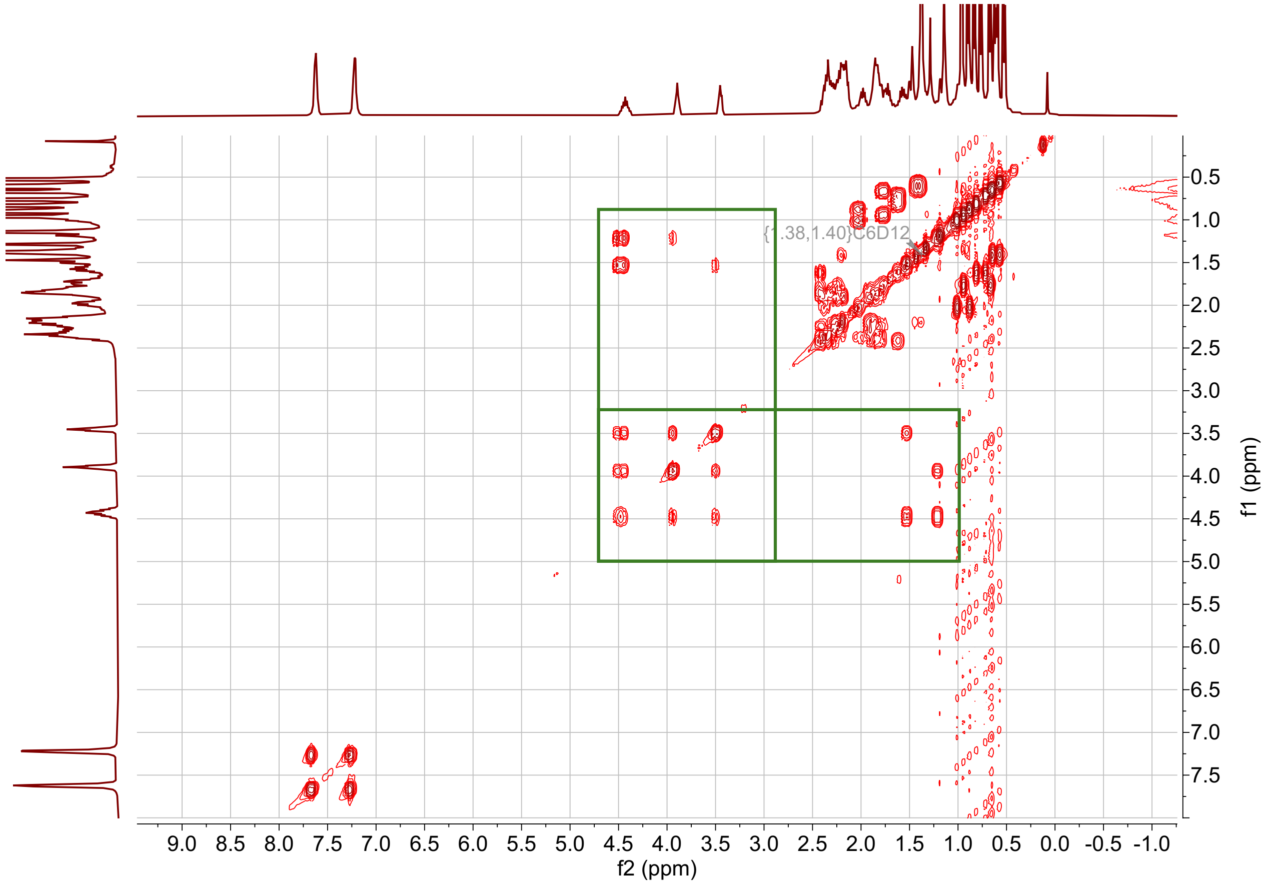

Figure S22. ^{1}H–^{1}H COSY NMR spectrum (cyclohexane-d_{12}, 23 °C) of **Co1**. Inset: ^{1}H–^{1}H correlation between η^5-cyclohexadienyl signals.

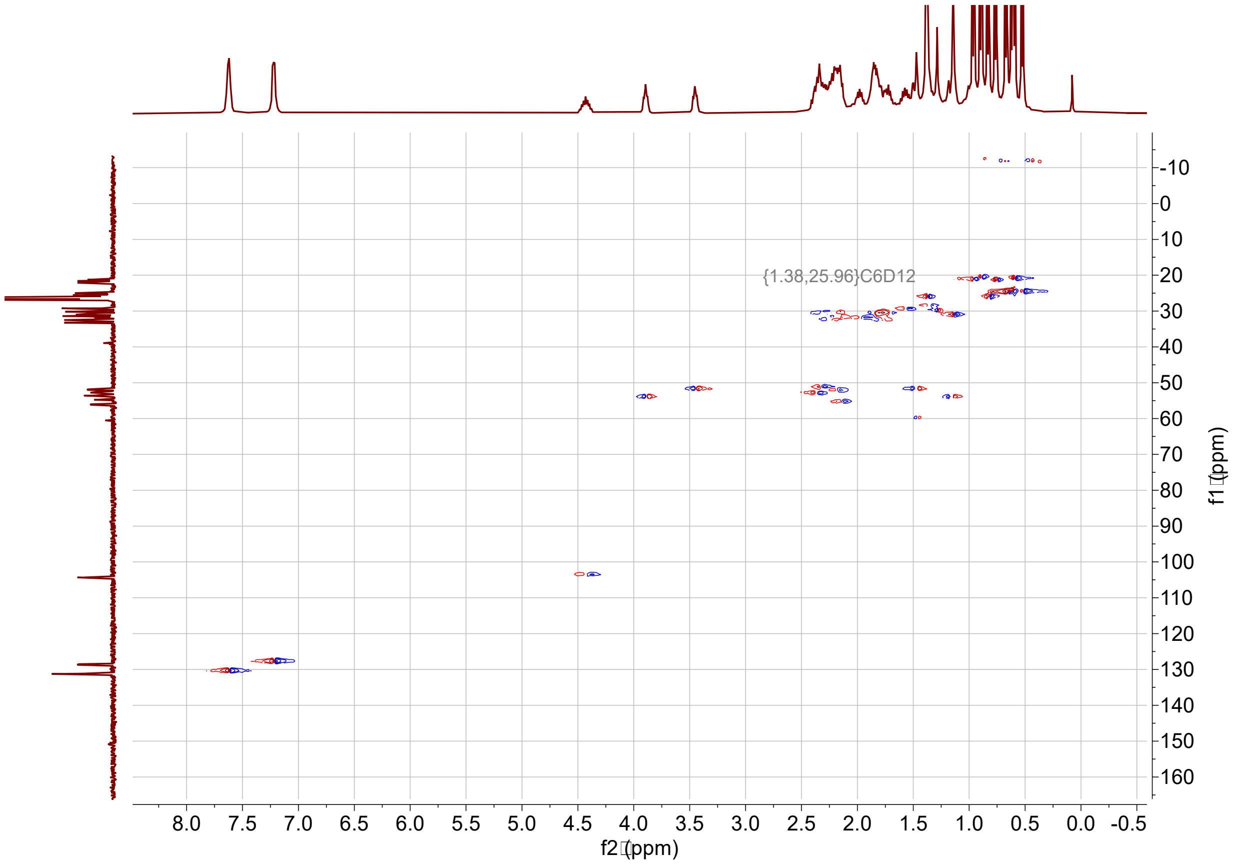

Figure S23. ^{1}H–$^{13}C\{^{1}H\}$ HSQC NMR spectrum (cyclohexane-d_{12}, 23 °C) of **Co1**.

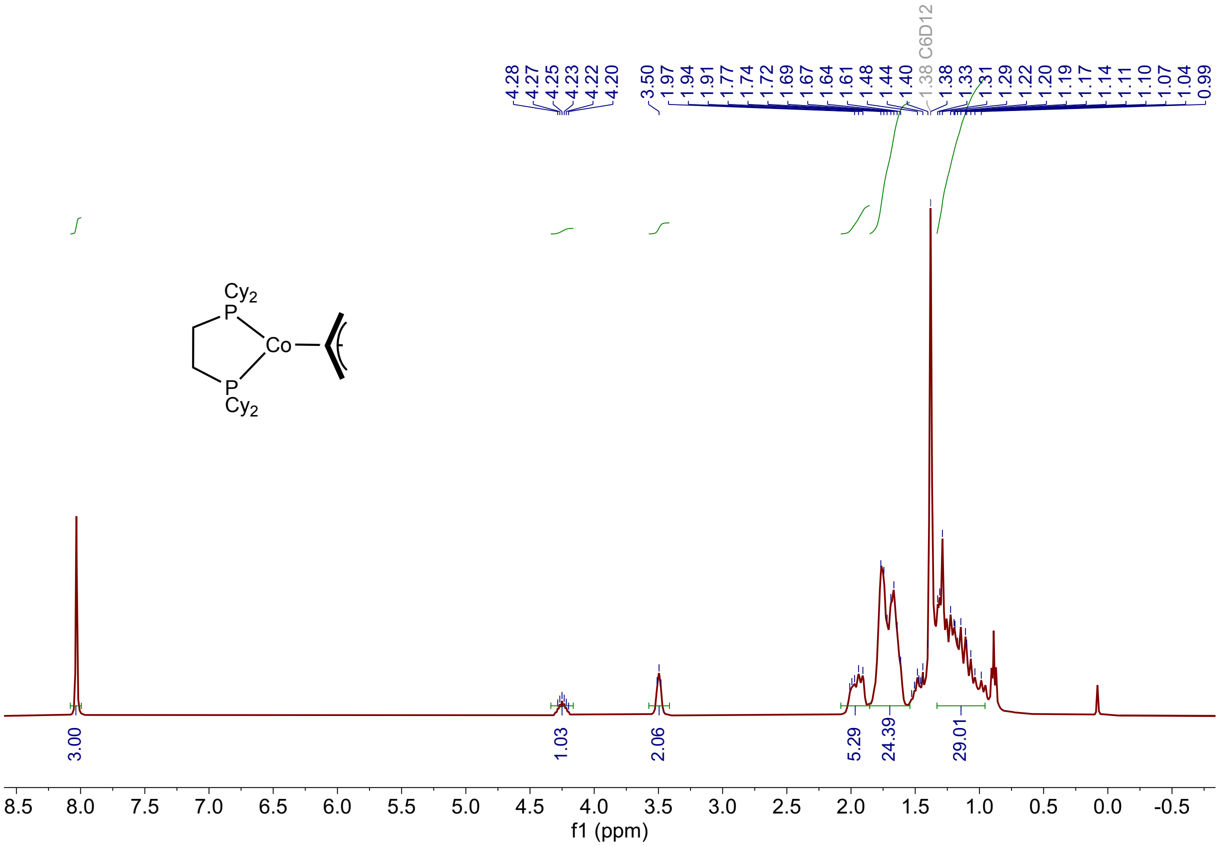

Figure S24. ^{1}H NMR spectrum (cyclohexane-d_{12}, 23 °C) of **Co2** with 1,3,5-tris(trifluoromethyl)benzene internal standard (δ 8.03 ppm).

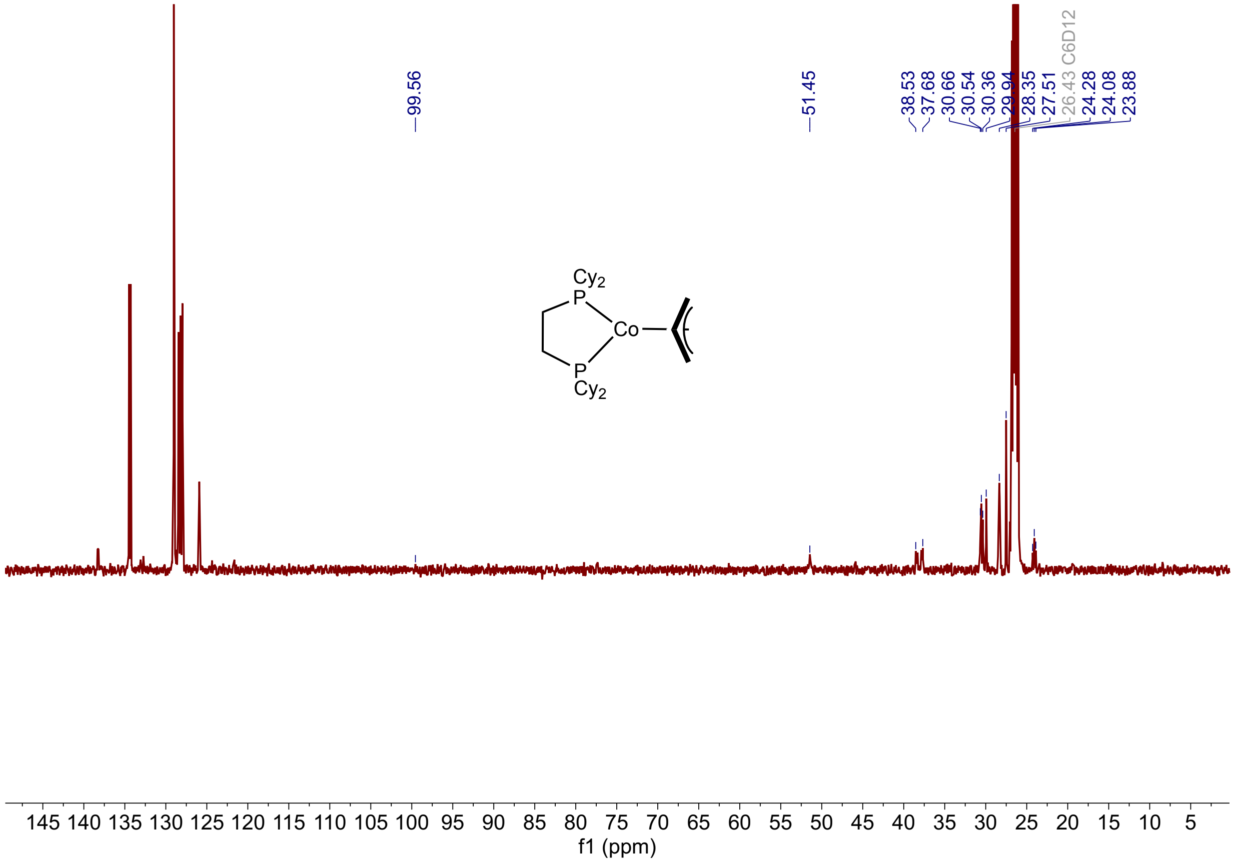

Figure S25. $^{13}C\{^1H\}$ NMR spectrum (cyclohexane-d_{12}, 23 °C) of **Co2** with 1,3,5-tris(trifluoromethyl)benzene (10 µmol) and triphenyl phosphine (10 µmol) internal standard associated with unassigned resonances.

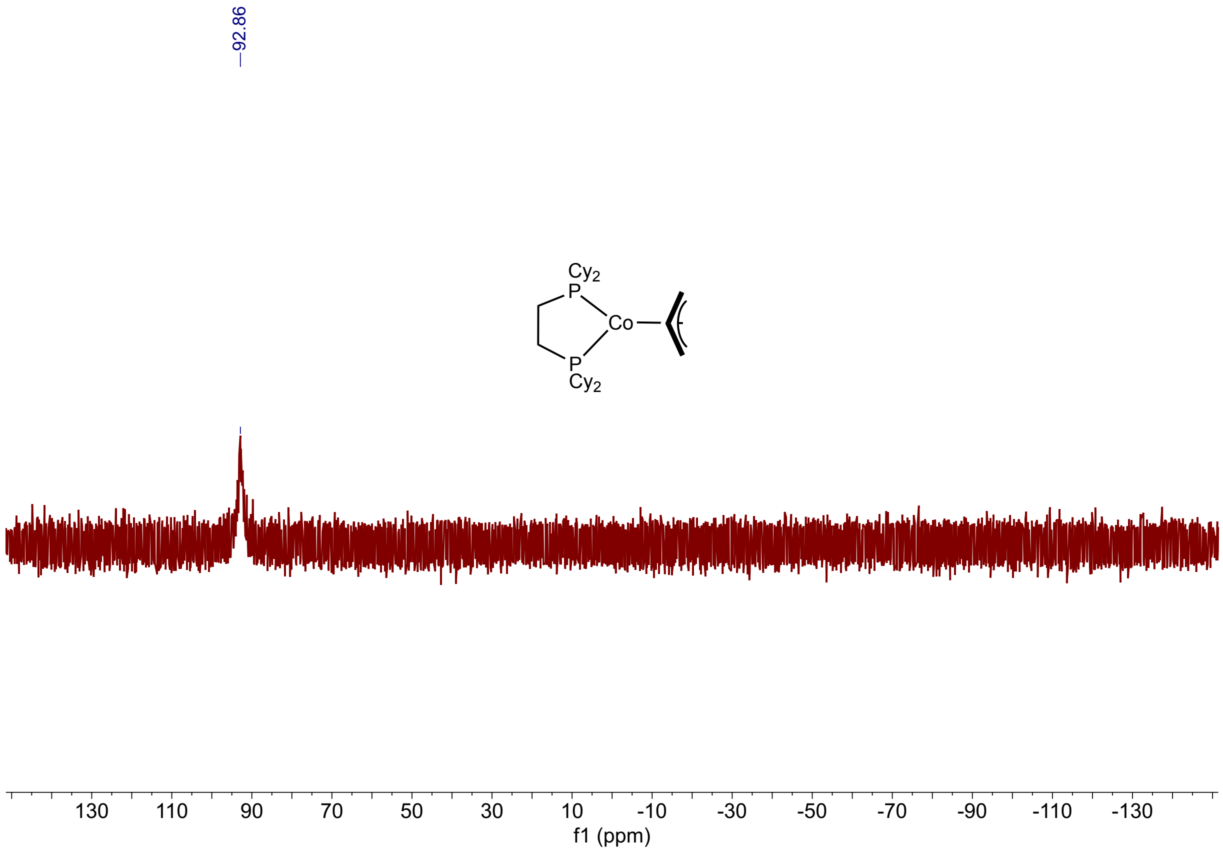

Figure S26. ^{31}P NMR spectrum (cyclohexane-d_{12}, 23 °C) of **Co2**.

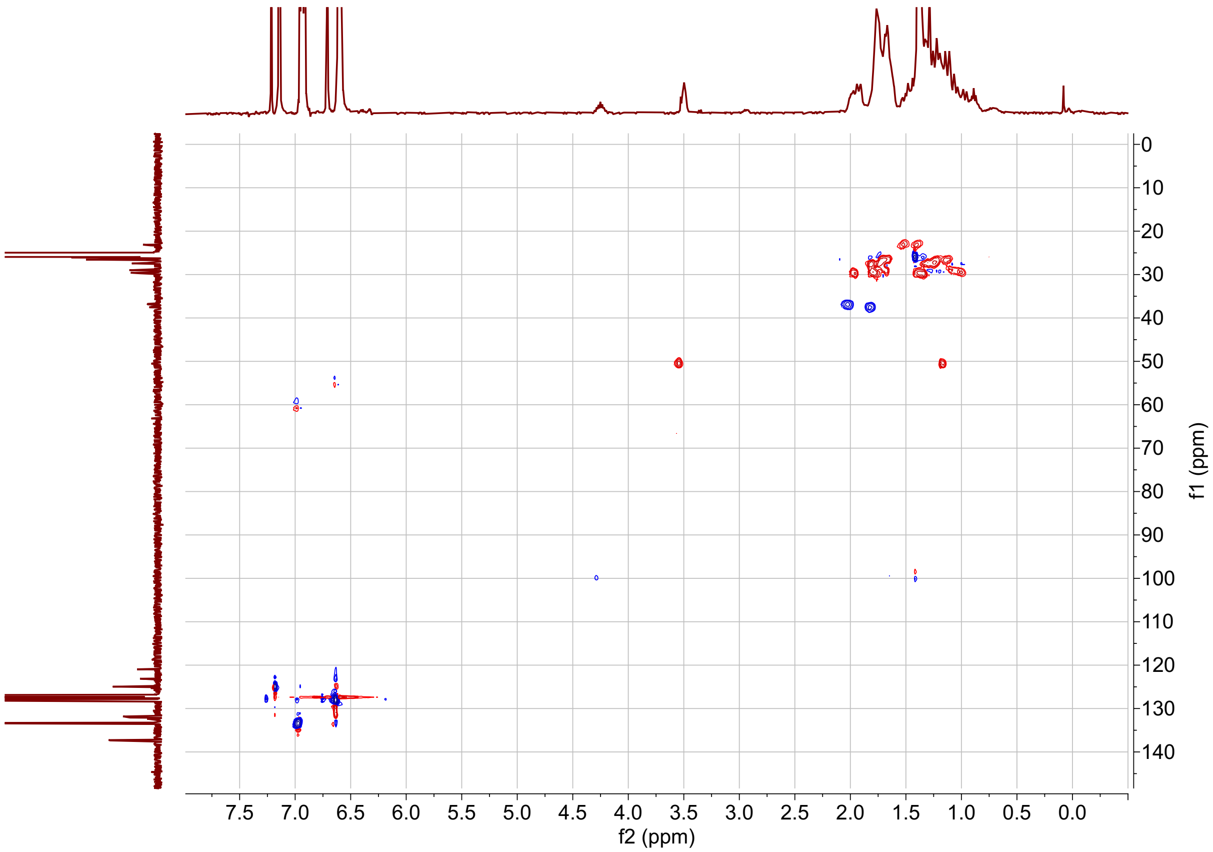

Figure S27. ^{1}H–$^{13}C\{^{1}H\}$ HSQC NMR spectrum (cyclohexane-d_{12}, 23 °C) of **Co2** with 1,3,5-tris(trifluoromethyl)benzene (10 μmol) and triphenyl phosphine (10 μmol) internal standard associated with unassigned resonances.

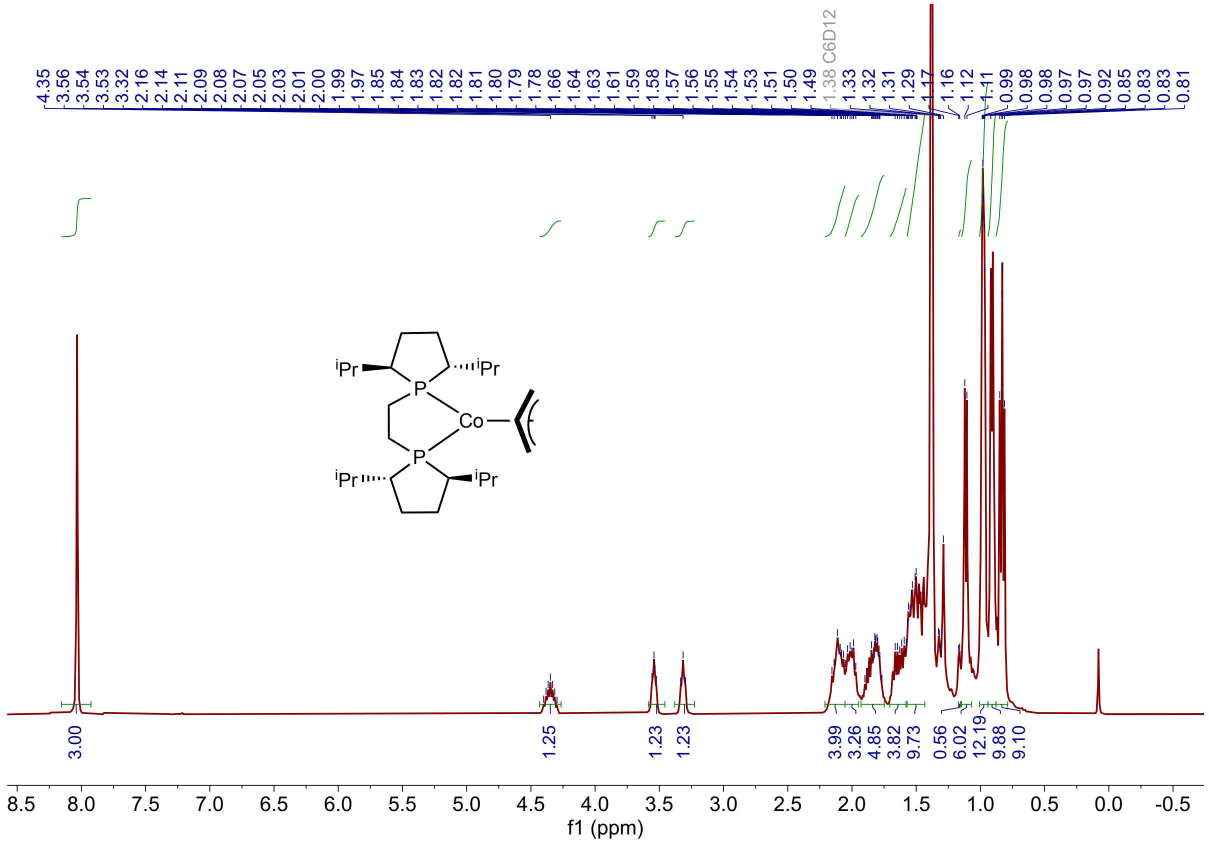

Figure S28. ^{1}H NMR spectrum (400 MHz, cyclohexane-d_{12}, 23 °C) of **Co3** with 1,3,5-tris(trifluoromethyl)benzene internal standard (δ 8.03 ppm).

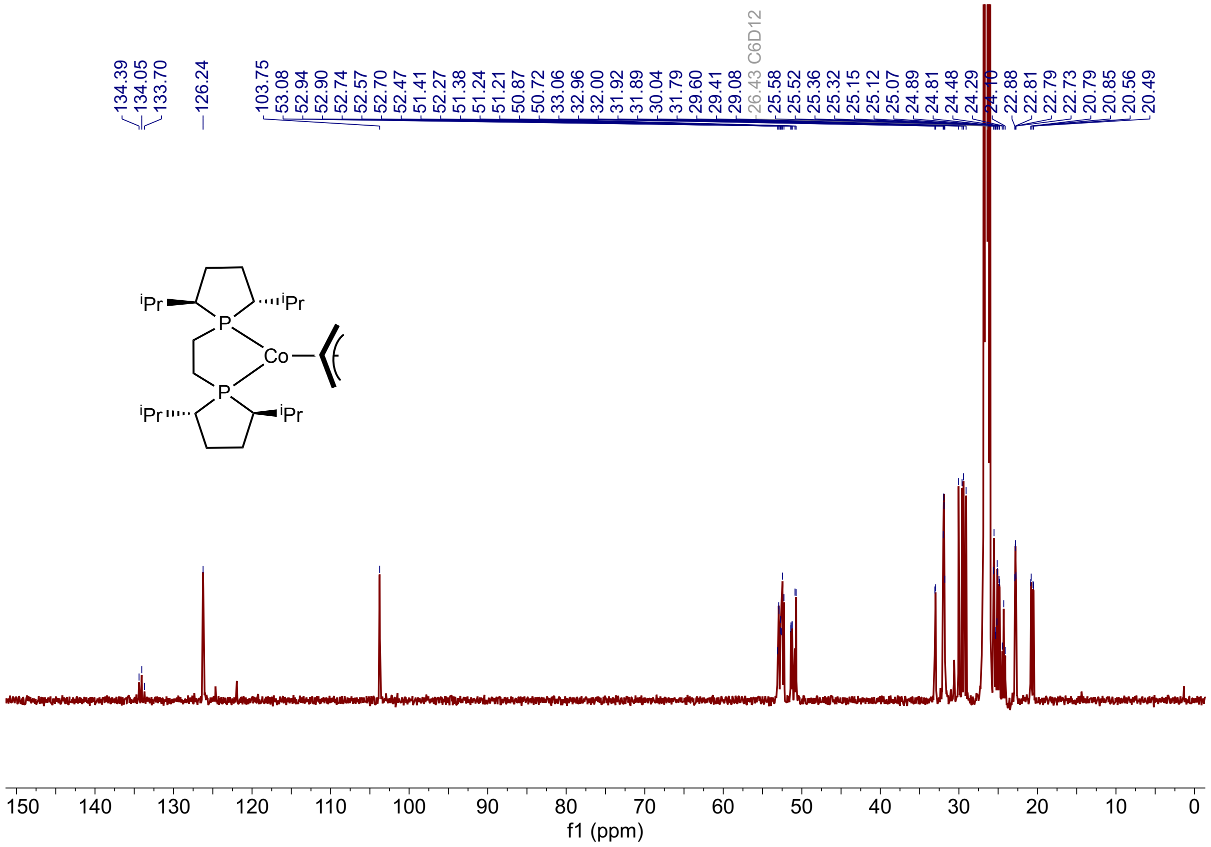

Figure S29. $^{13}C\{^1H\}$ NMR spectrum (101 MHz, cyclohexane-d_{12}, 23 °C) of **Co3** with 1,3,5-tris(trifluoromethyl)benzene.

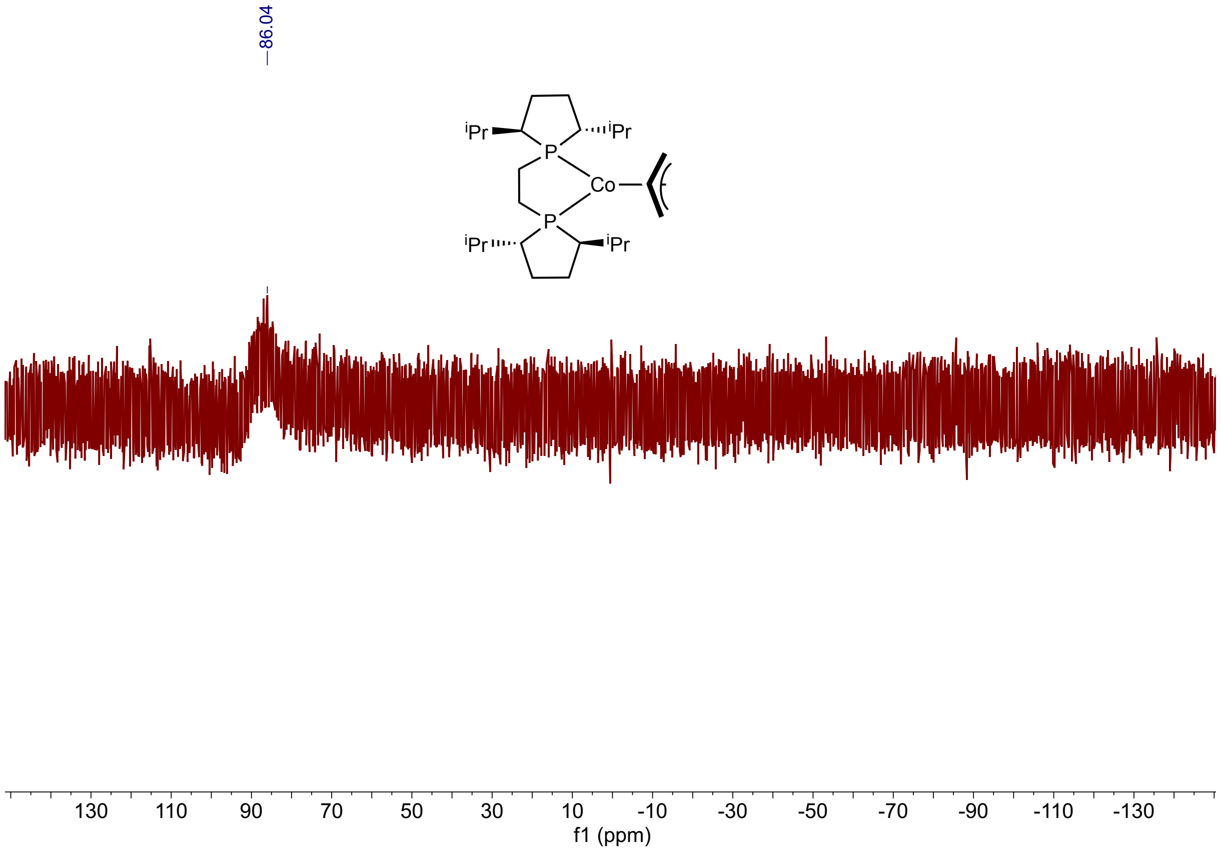

Figure S30. $^{31}P\{^{1}H\}$ NMR spectrum (162 MHz, cyclohexane-d_{12}, 23 °C) of **Co3**.

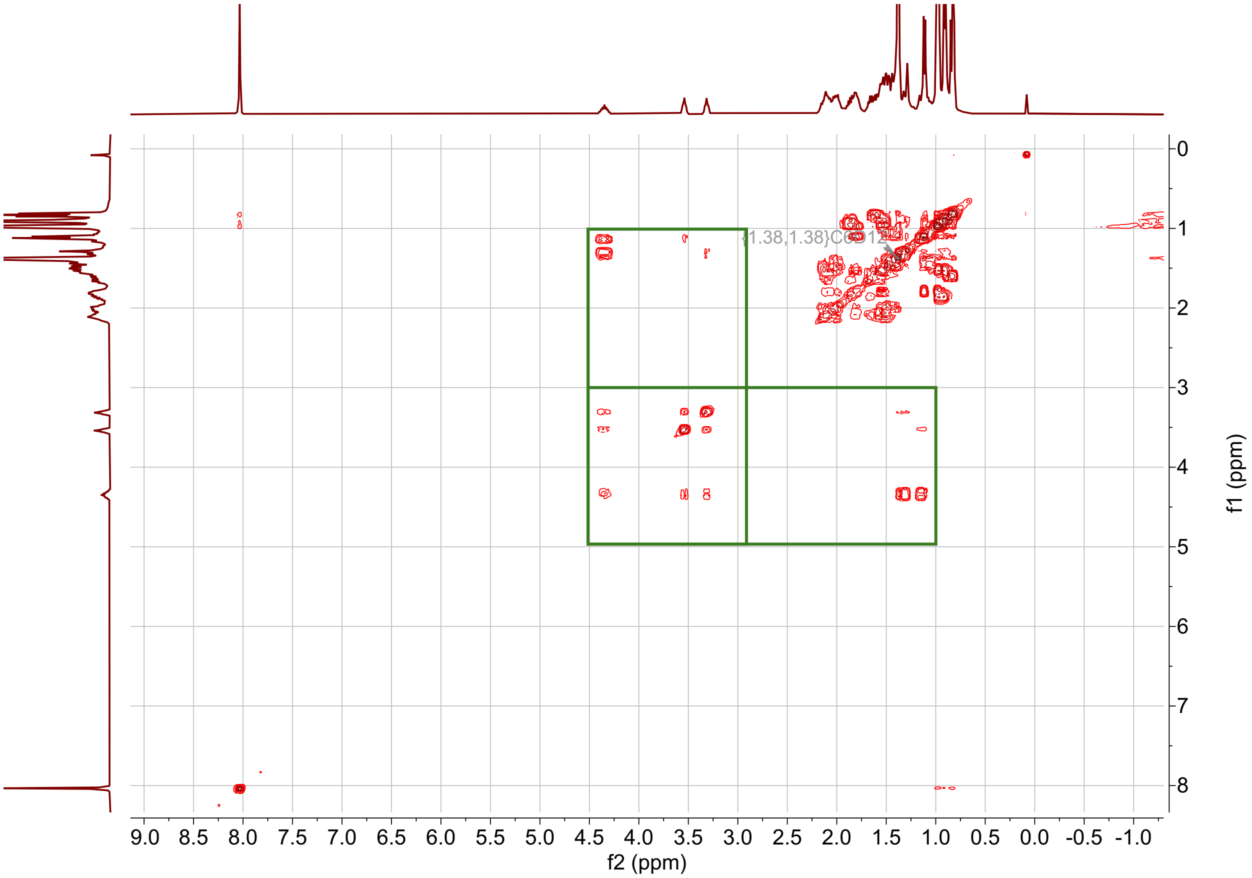

Figure S31. ^{1}H–^{1}H COSY NMR spectrum (cyclohexane-d_{12}, 23 °C) of **Co3** with 1,3,5-tris(trifluoromethyl)benzene. Inset: ^{1}H–^{1}H correlation between η^{5}-cyclohexadienyl signals.

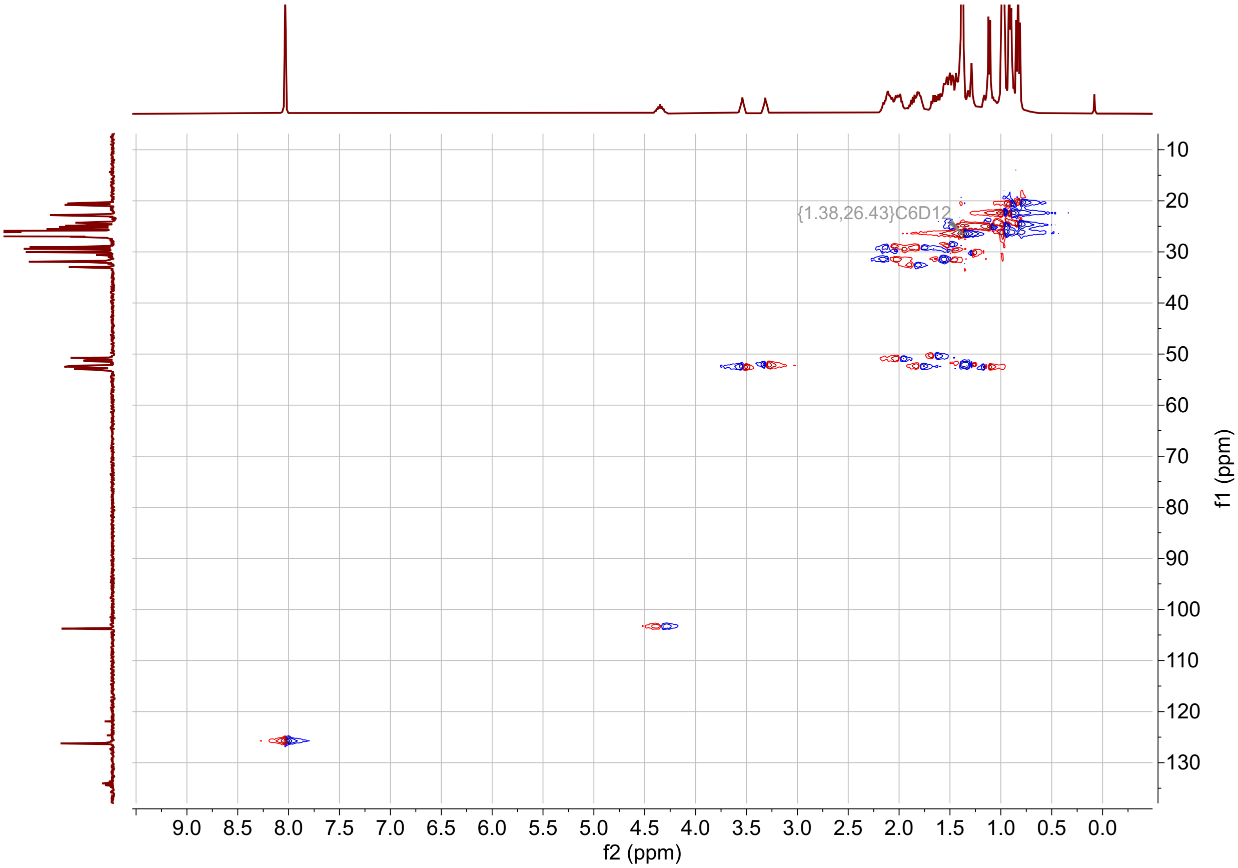

Figure S32. $^1H–^{13}C\{^1H\}$ HSQC NMR spectrum (cyclohexane-d_{12}, 23 °C) of **Co3** with 1,3,5-tris(trifluoromethyl)benzene.

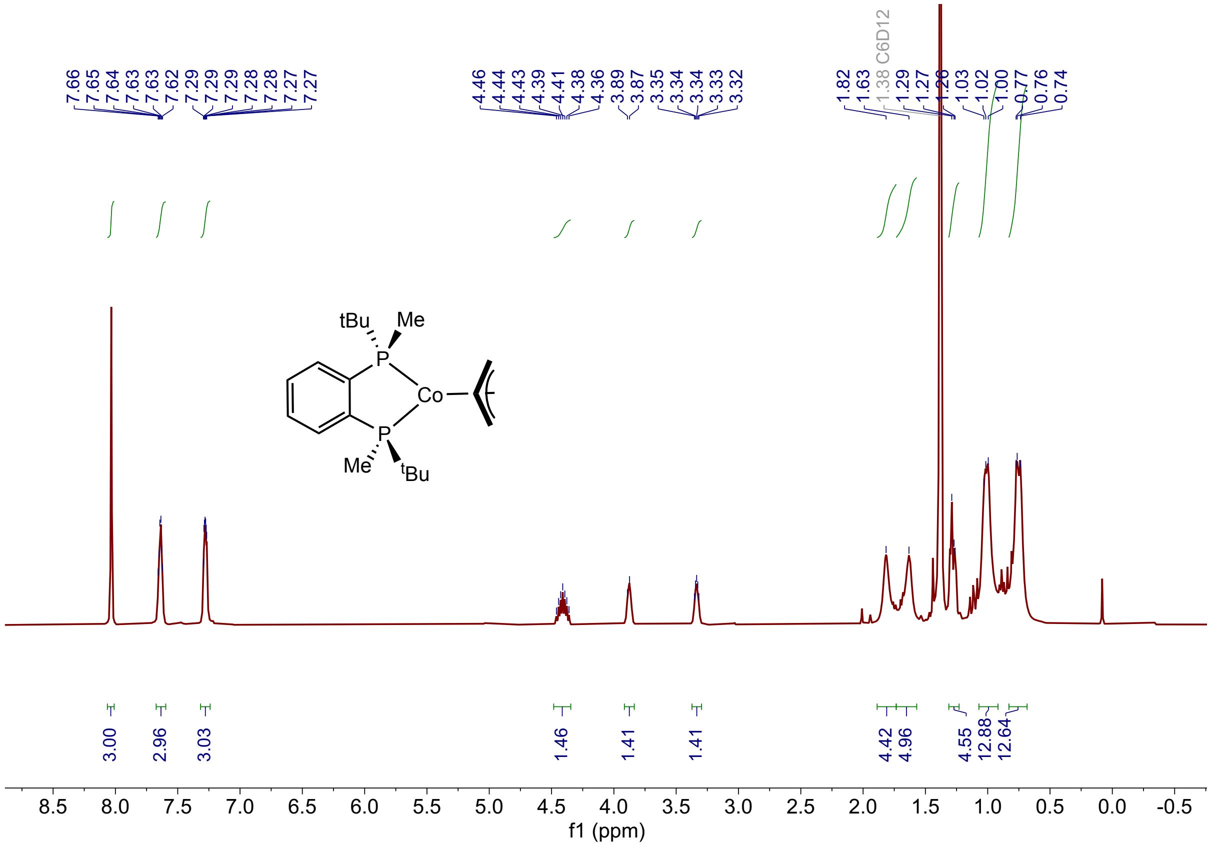

Figure S33. ^{1}H NMR spectrum (400 MHz, cyclohexane-d_{12}, 23 °C) of **Co4** with 1,3,5-tris(trifluoromethyl)benzene internal standard (δ 8.03 ppm).

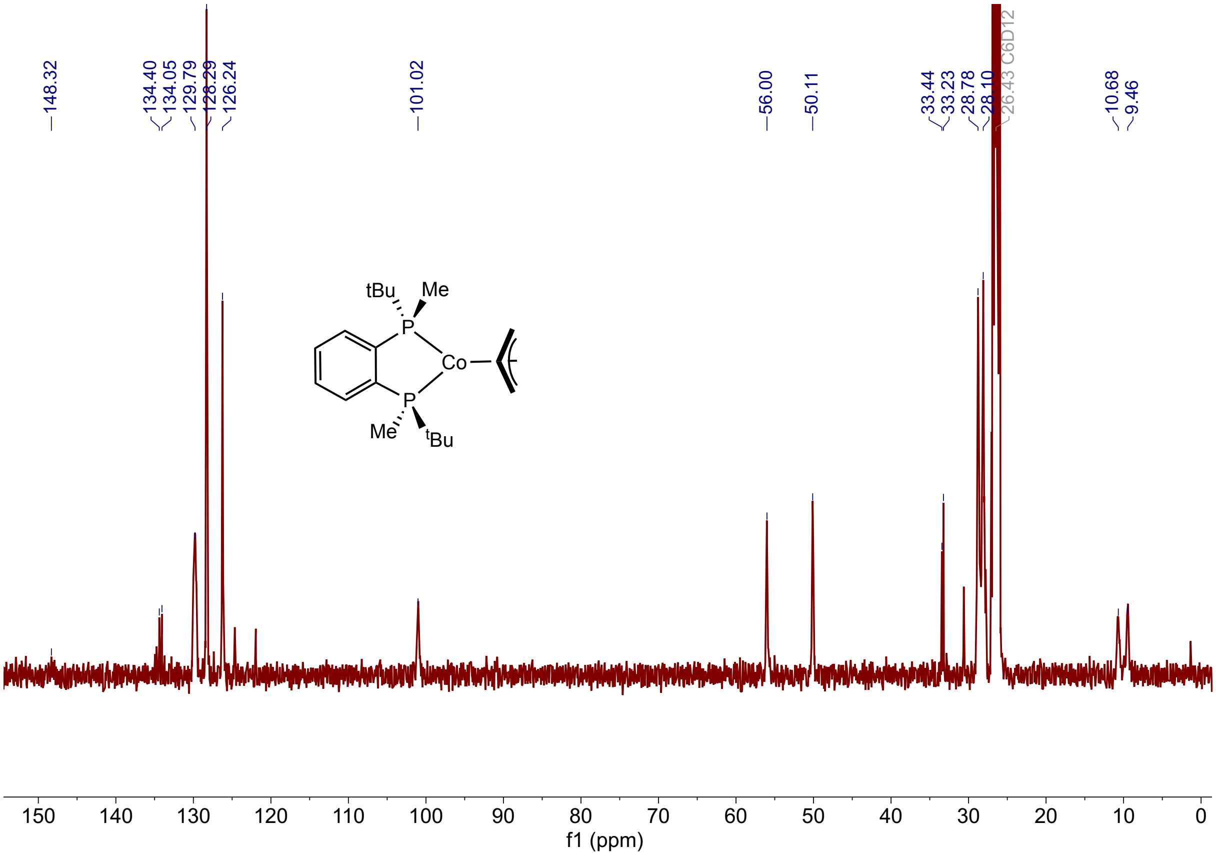

Figure S34. $^{13}C\{^1H\}$ NMR spectrum (101 MHz, cyclohexane-d_{12}, 23 °C) of **Co4** with 1,3,5-tris(trifluoromethyl)benzene.

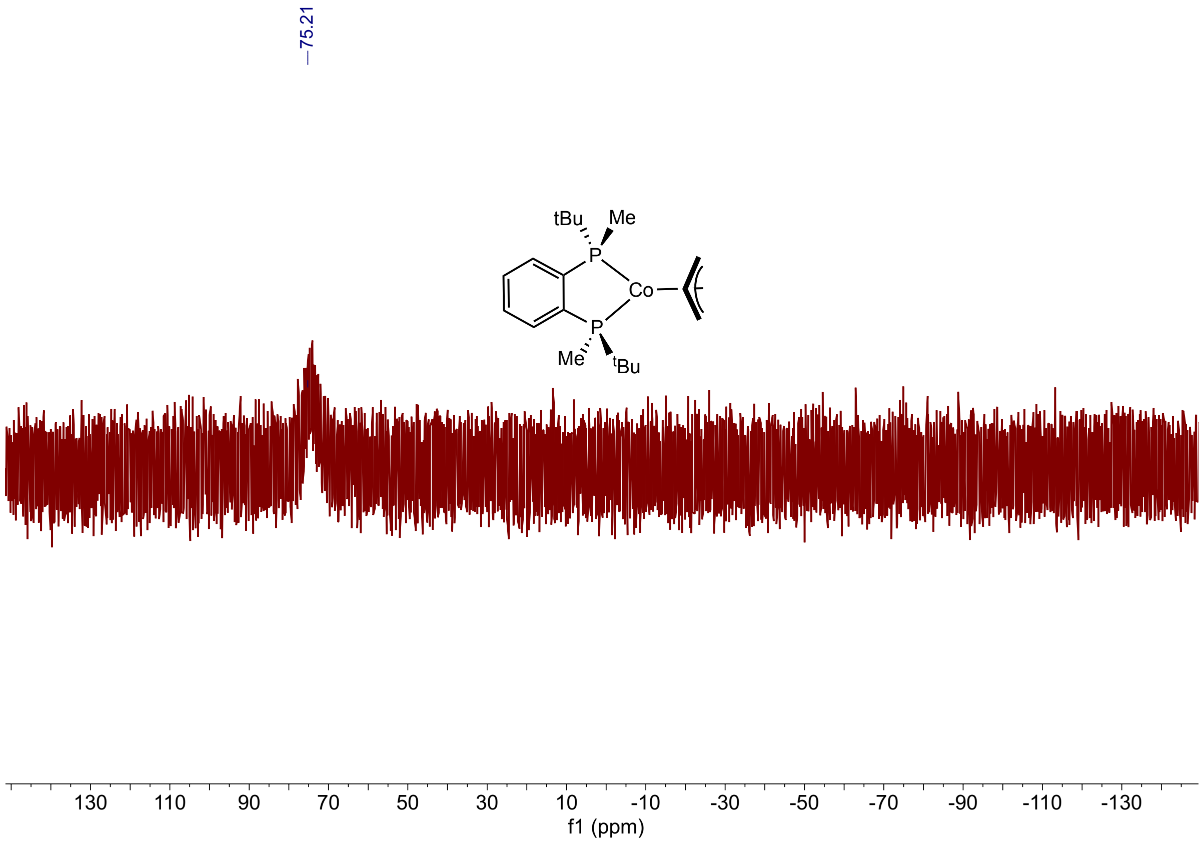

Figure S35. $^{31}P\{^1H\}$ NMR spectrum (162 MHz, cyclohexane-d_{12}, 23 °C) of **Co4**.

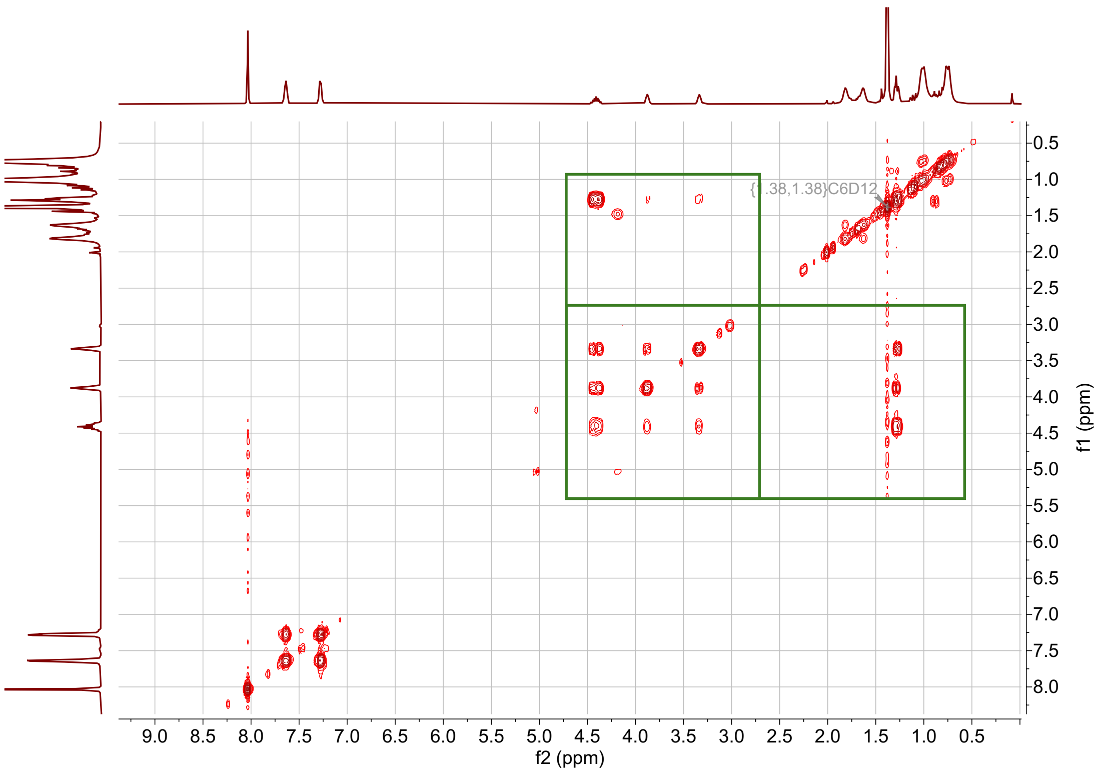

Figure S36. ^1H–^1H COSY NMR spectrum (cyclohexane-d_{12}, 23 °C) of **Co4** with 1,3,5-tris(trifluoromethyl)benzene. Inset: ^1H–^1H correlation between η^5-cyclohexadienyl signals.

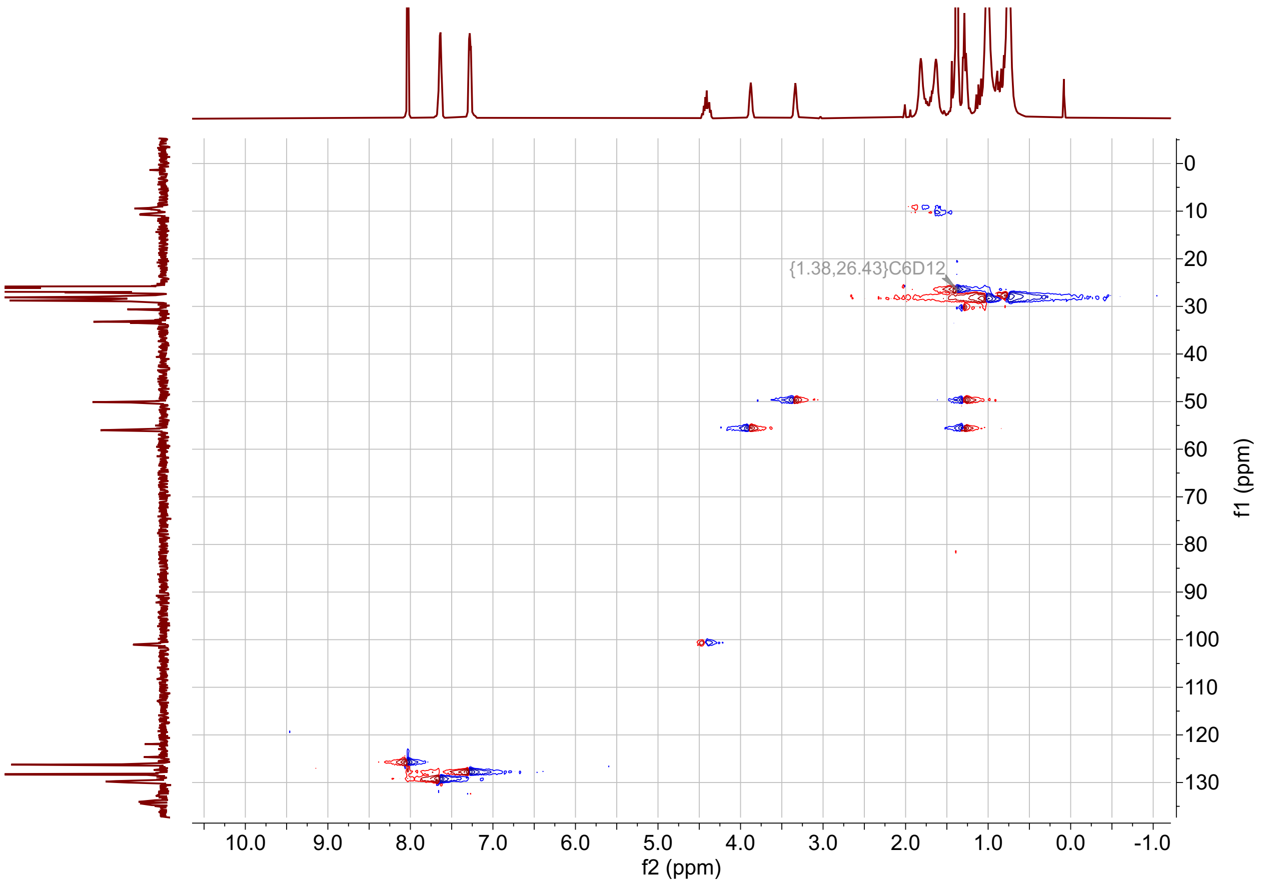

Figure S37. ^{1}H–$^{13}C\{^{1}H\}$ HSQC NMR spectrum (cyclohexane-d_{12}, 23 °C) of **Co4** with 1,3,5-tris(trifluoromethyl)benzene.

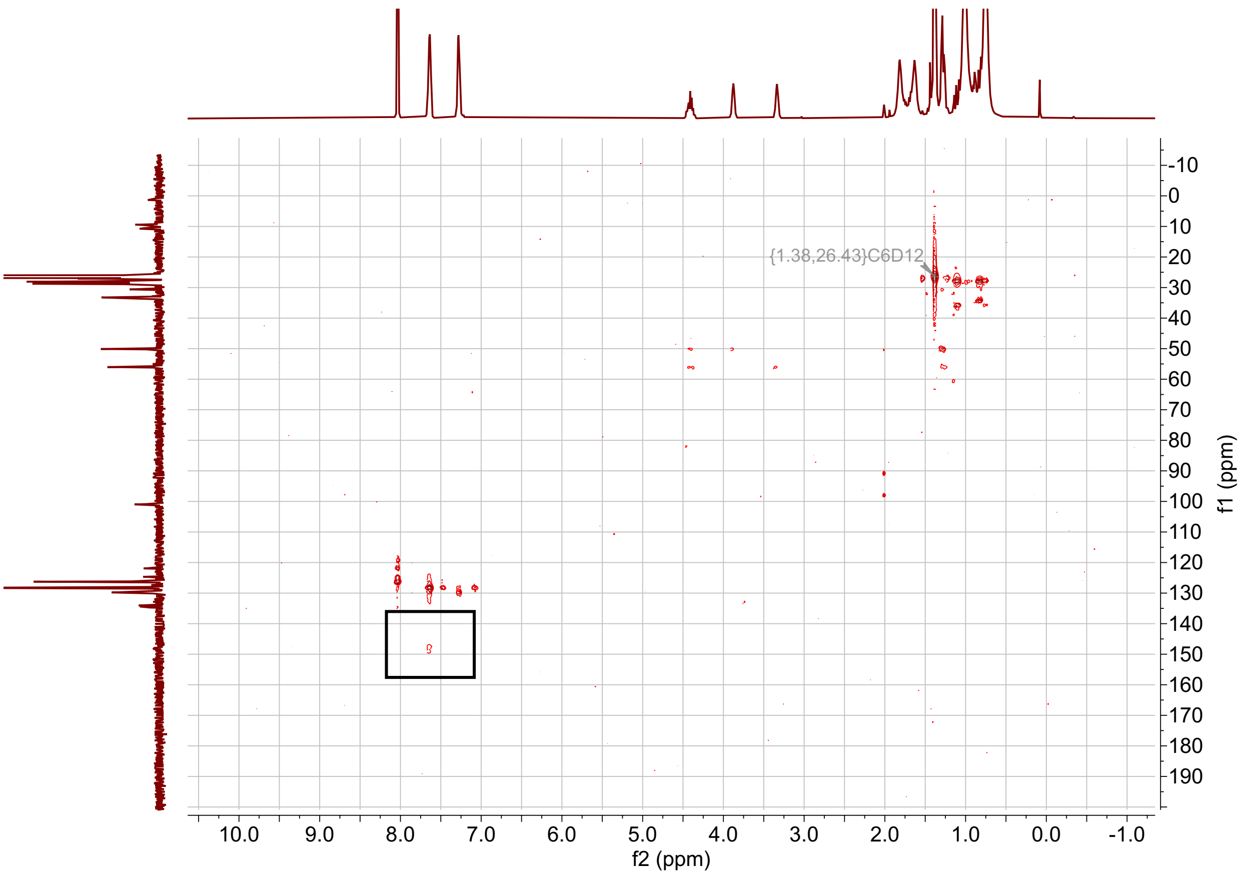

Figure S38. $^{1}H–^{13}C\{^{1}H\}$ HMBC NMR spectrum (cyclohexane-d_{12}, 23 °C) of **Co4**. Inset: $^{1}H–^{13}C$ correlation between BenzP* Ar quaternary carbon signals.

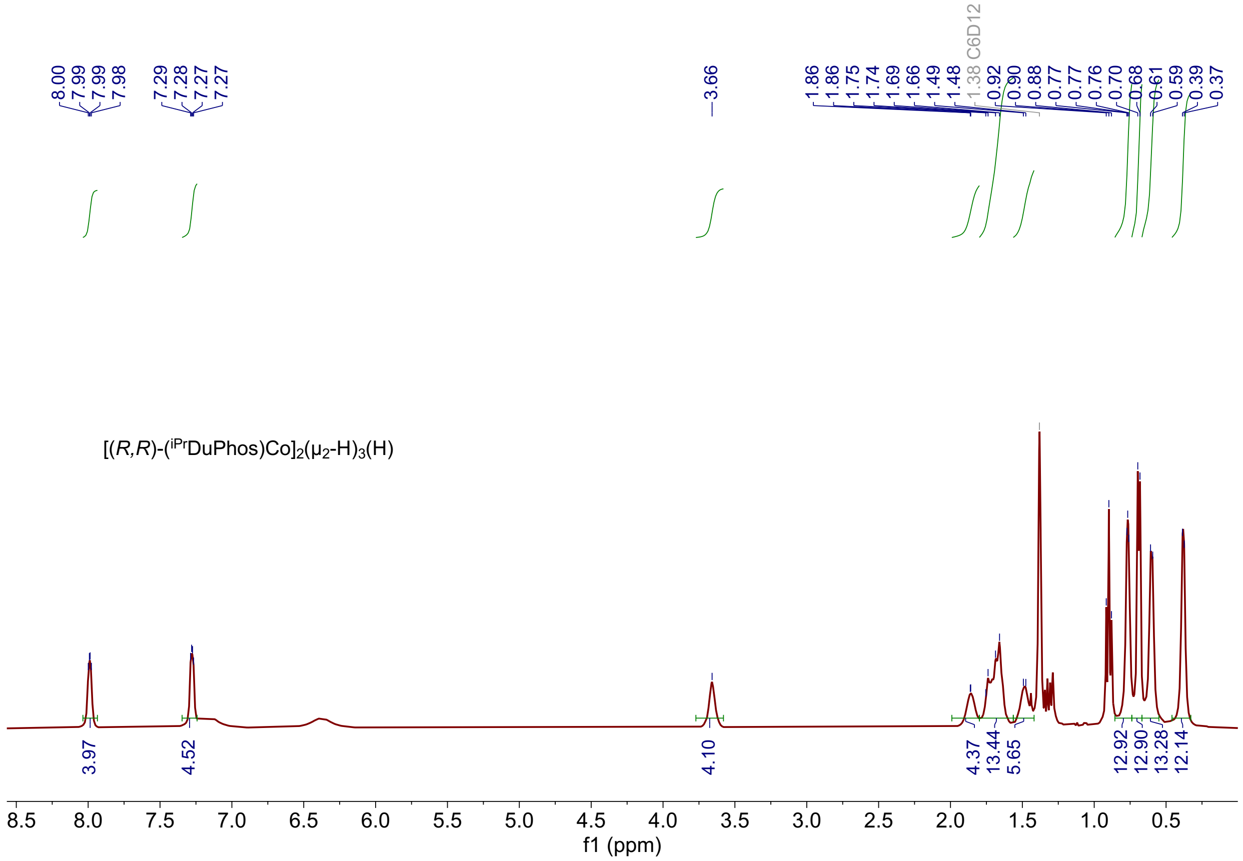

Figure S39. ^{1}H NMR spectrum (cyclohexane-d_{12}, 23 °C) of **Co1-H**.

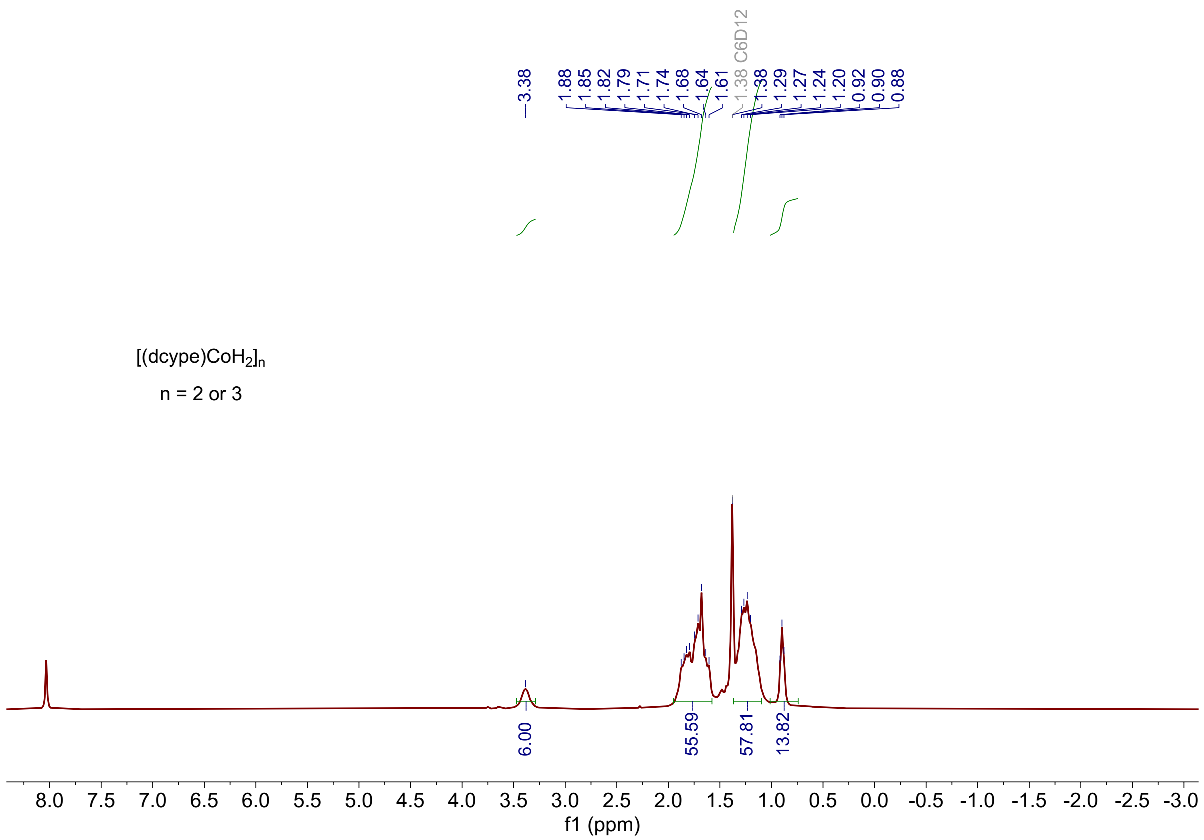

Figure S40. ^{1}H NMR spectrum (400 MHz, cyclohexane-d_{12}, 23 °C) of **Co2-H** with 1,3,5-tris(trifluoromethyl)benzene internal standard (δ 8.03 ppm).

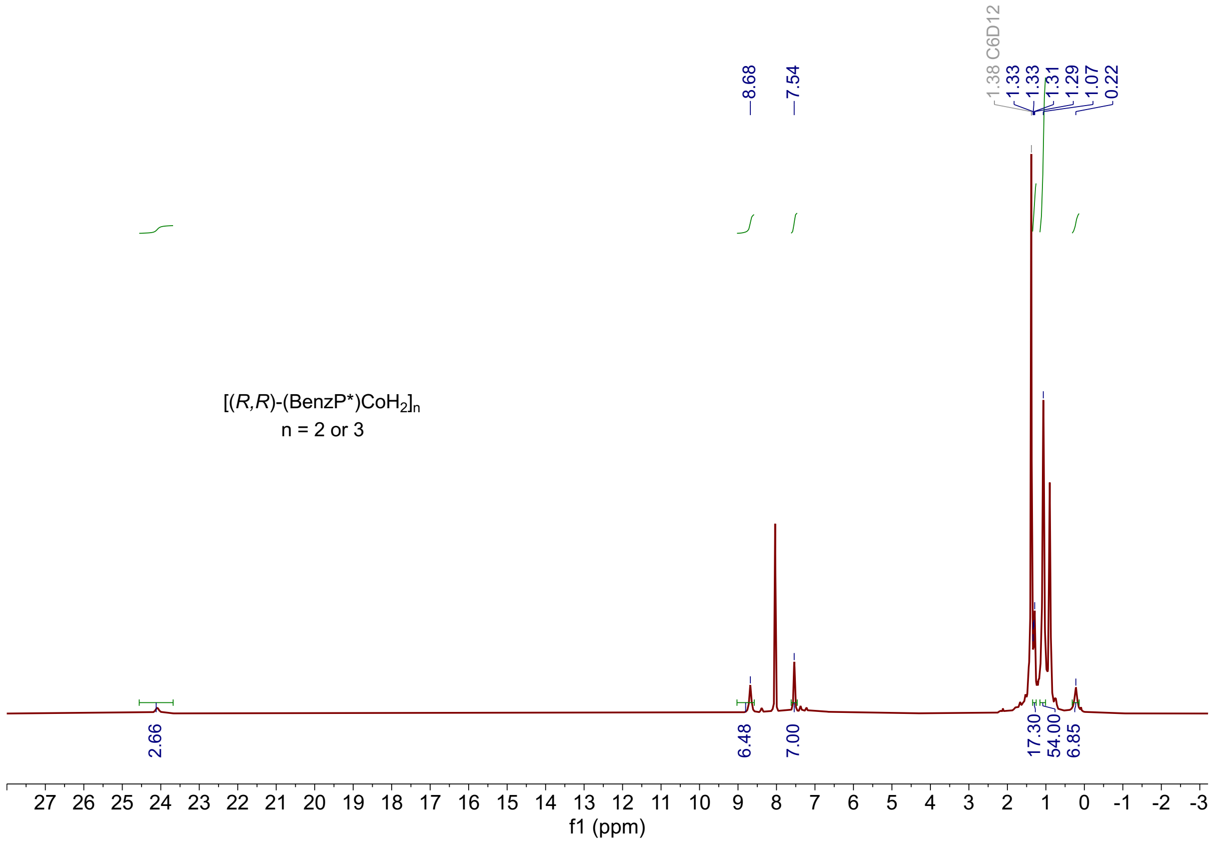

Figure S41. ^{1}H NMR spectrum (400 MHz, cyclohexane-d_{12}, 23 °C) of **Co4-H** with 1,3,5-tris(trifluoromethyl)benzene internal standard (δ 8.03 ppm).

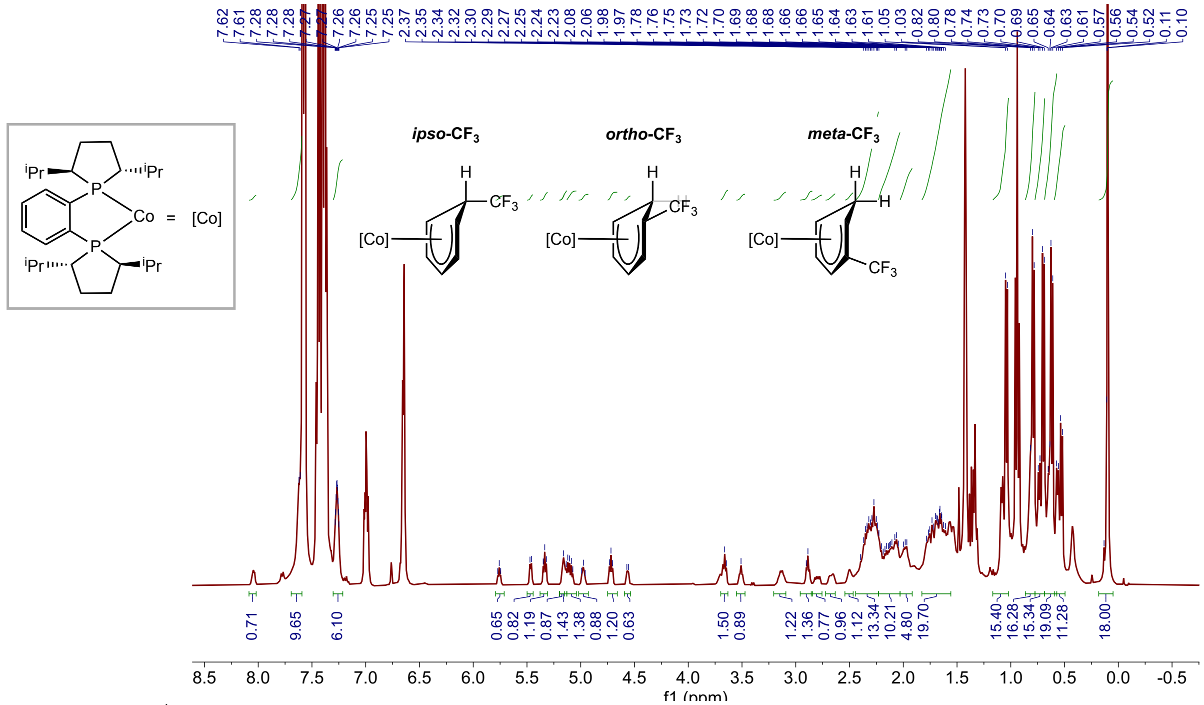

Figure S42. ^{1}H NMR spectrum (400 MHz, cyclohexane-d_{12}, 23 °C) of kinetic products of **Co1-a** in excess $PhCF_3$. Unassigned resonances associated with PPh_3 (δ 6.94, 6.59 ppm) and HMDSO (δ 0.10 ppm) capillary in cyclohexane-d_{12}.

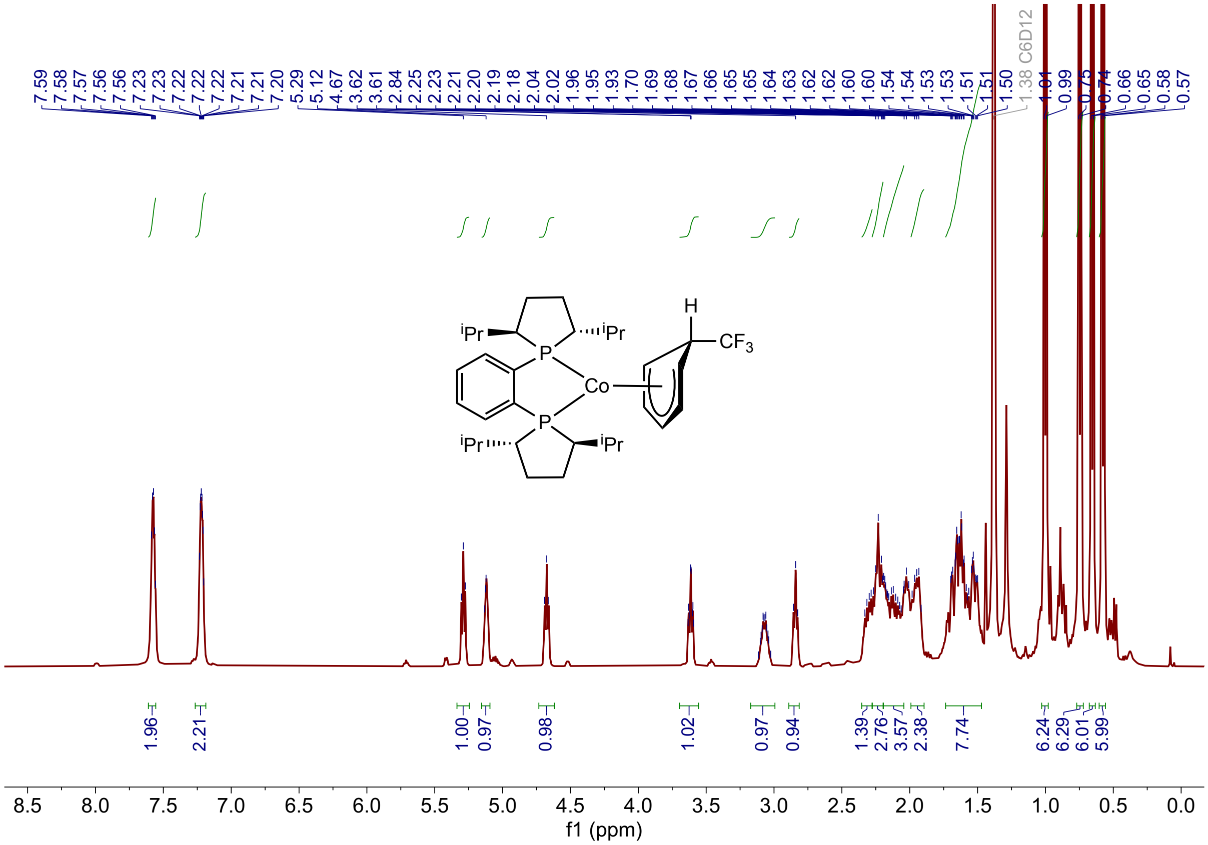

Figure S43. ^{1}H NMR spectrum (400 MHz, cyclohexane-d_{12}, 23 °C) of isolated **Co1-a**.

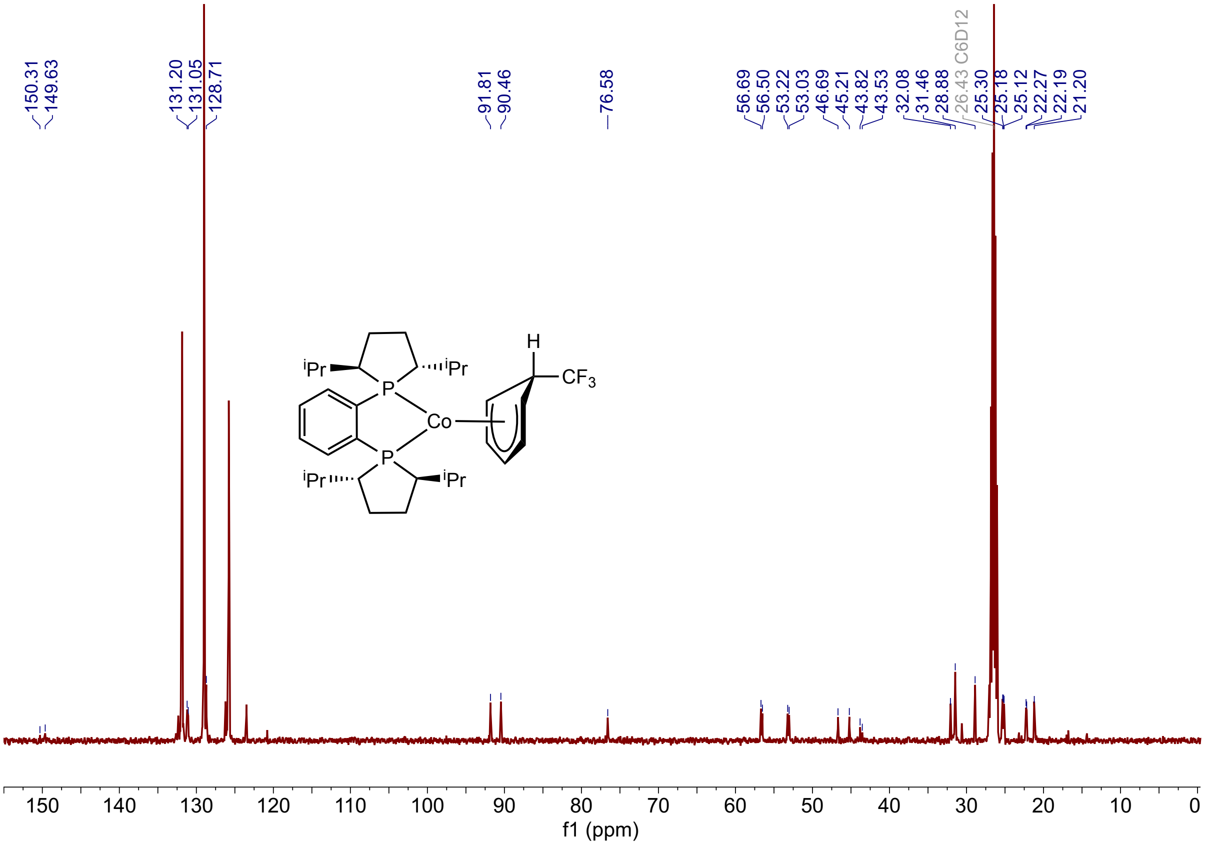

Figure S44. $^{13}C\{^1H\}$ NMR spectrum (101 MHz, cyclohexane-d_{12}, 23 °C) of **Co1-a** in excess $PhCF_3$.

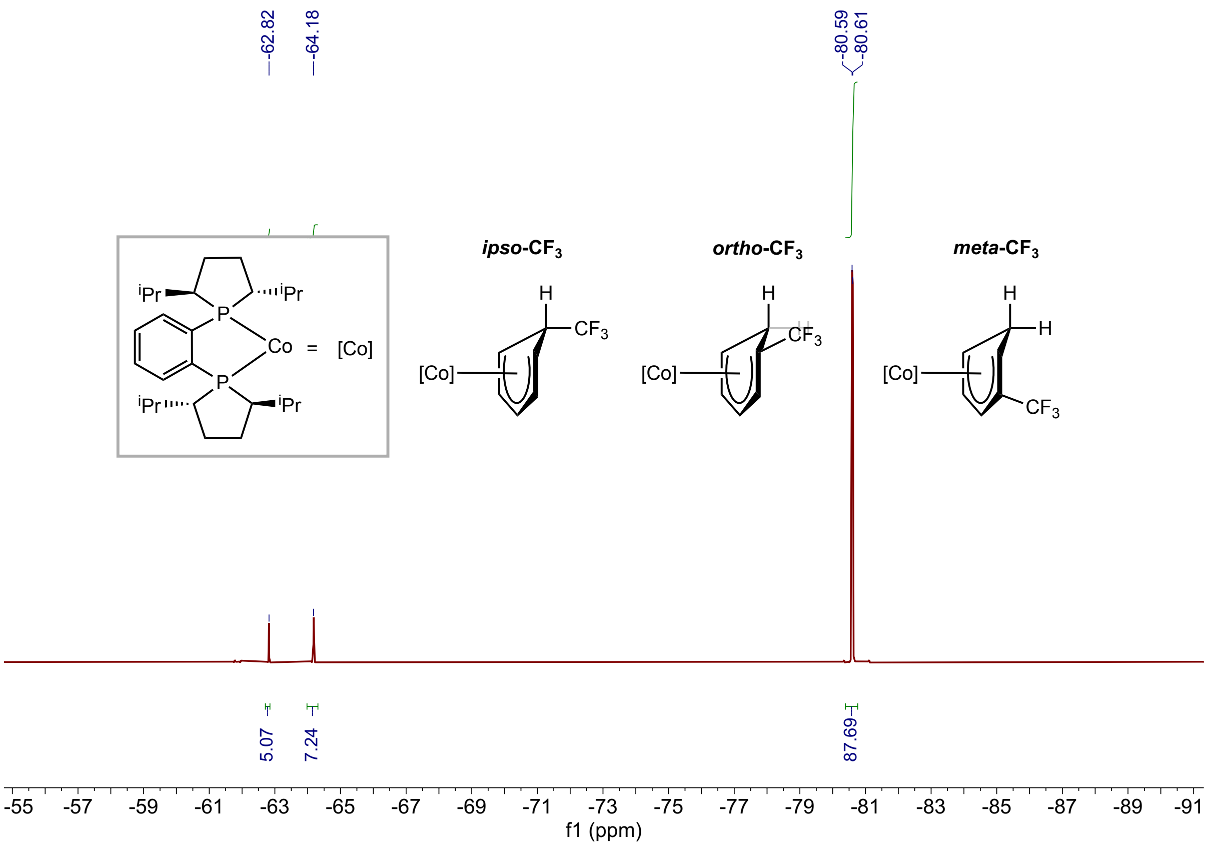

Figure S45. ^{19}F NMR spectrum (376 MHz, cyclohexane-d_{12}, 23 °C) of isolated **Co1-a**.

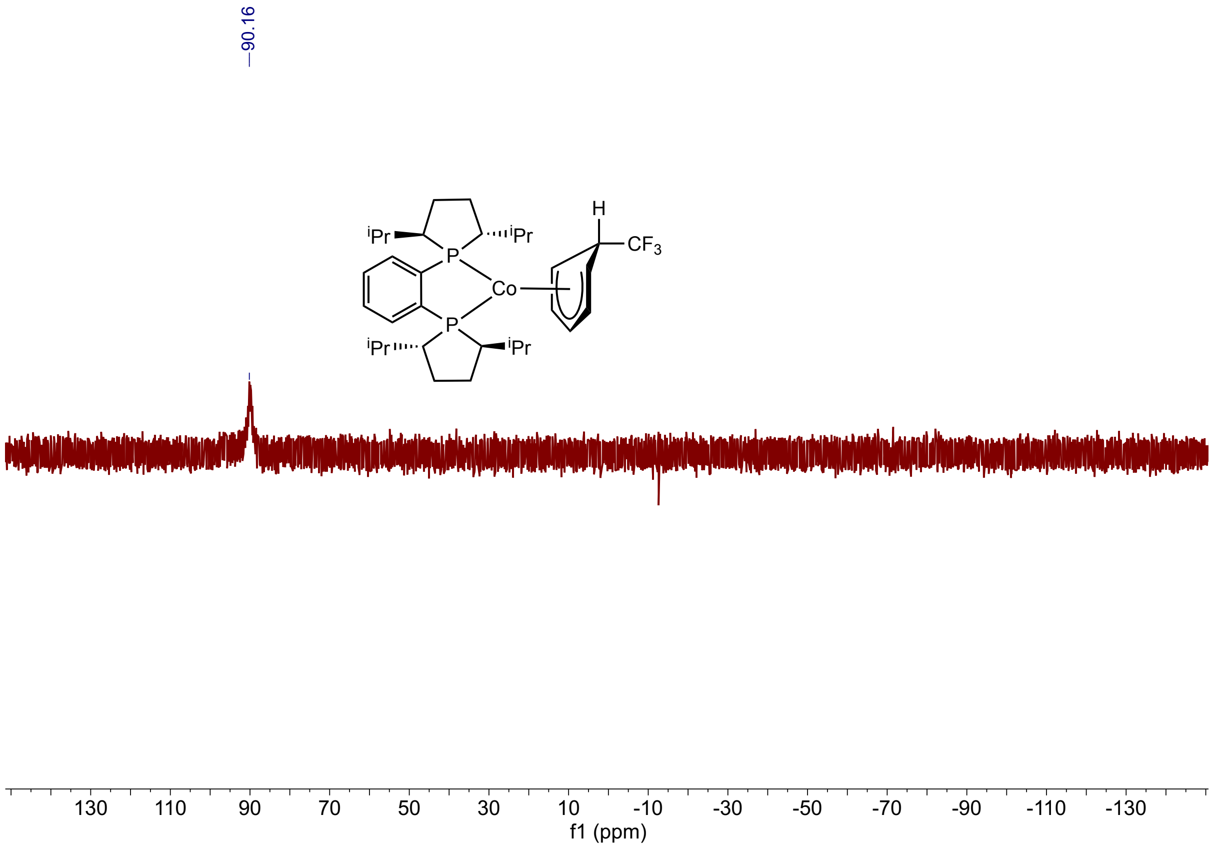

Figure S46. $^{31}P\{^{1}H\}$ NMR spectrum (162 MHz, cyclohexane-d_{12}, 23 °C) of isolated **Co1-a**.

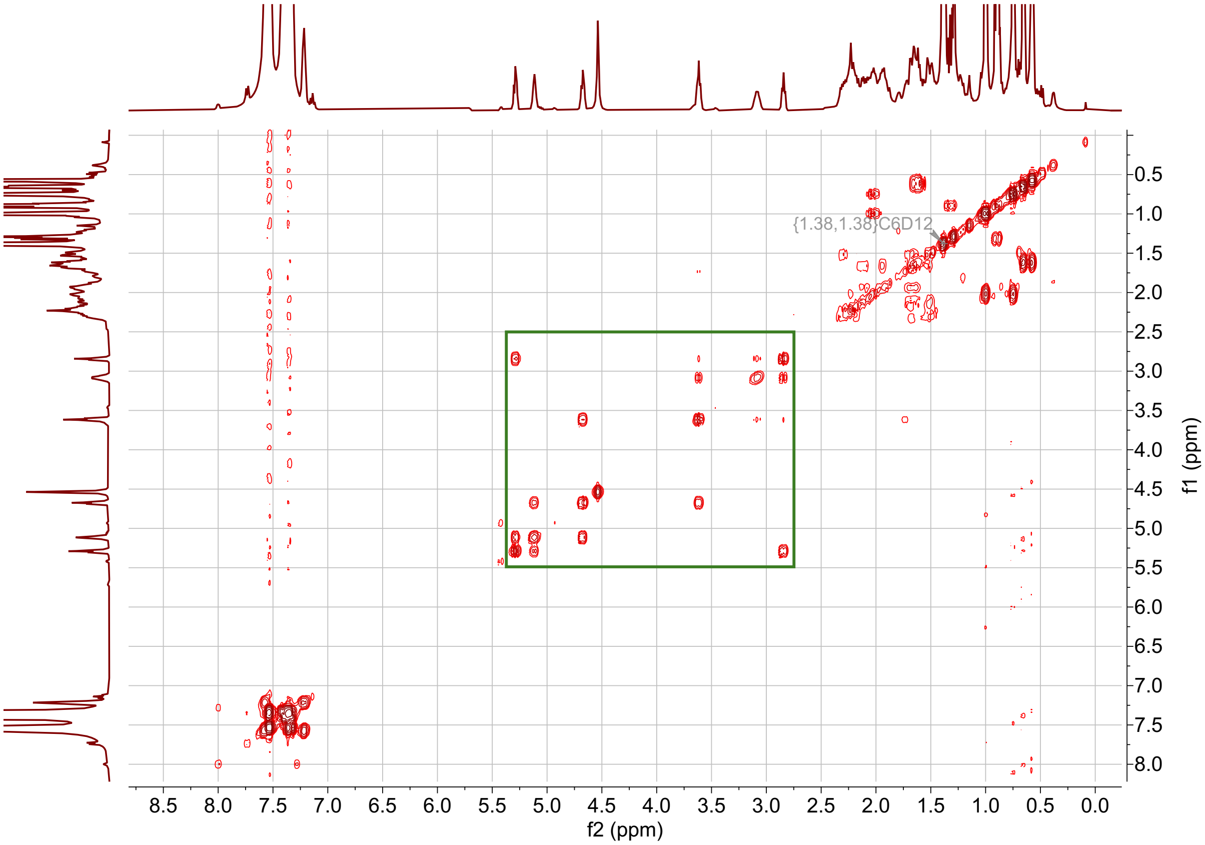

Figure S47. ^1H–^1H COSY NMR spectrum (cyclohexane-d_{12}, 23 °C) of **Co1-a** in excess $PhCF_3$.

Inset: ^1H–^1H correlation between η^5-cyclohexadienyl signals.

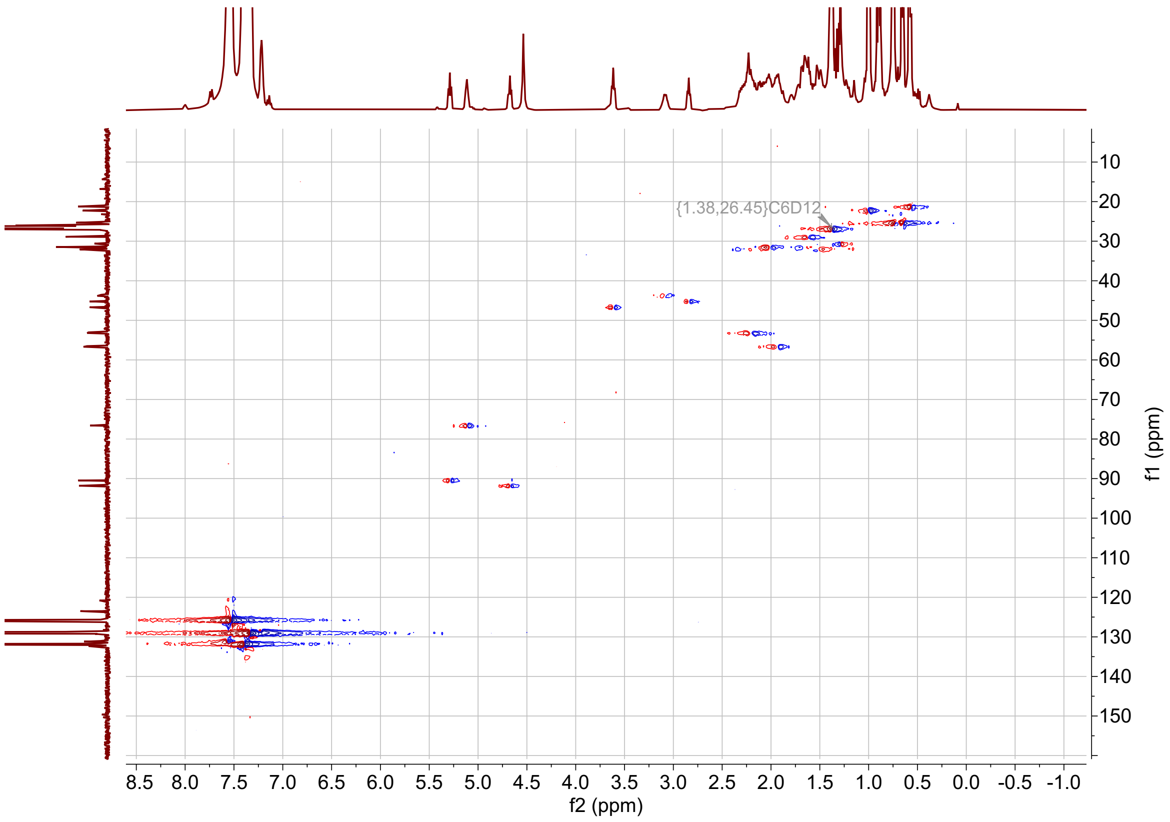

Figure S48. ^{1}H–^{13}C{^{1}H} HSQC NMR spectrum (cyclohexane-d_{12}, 23 °C) of **Co1-a** in excess $PhCF_3$.

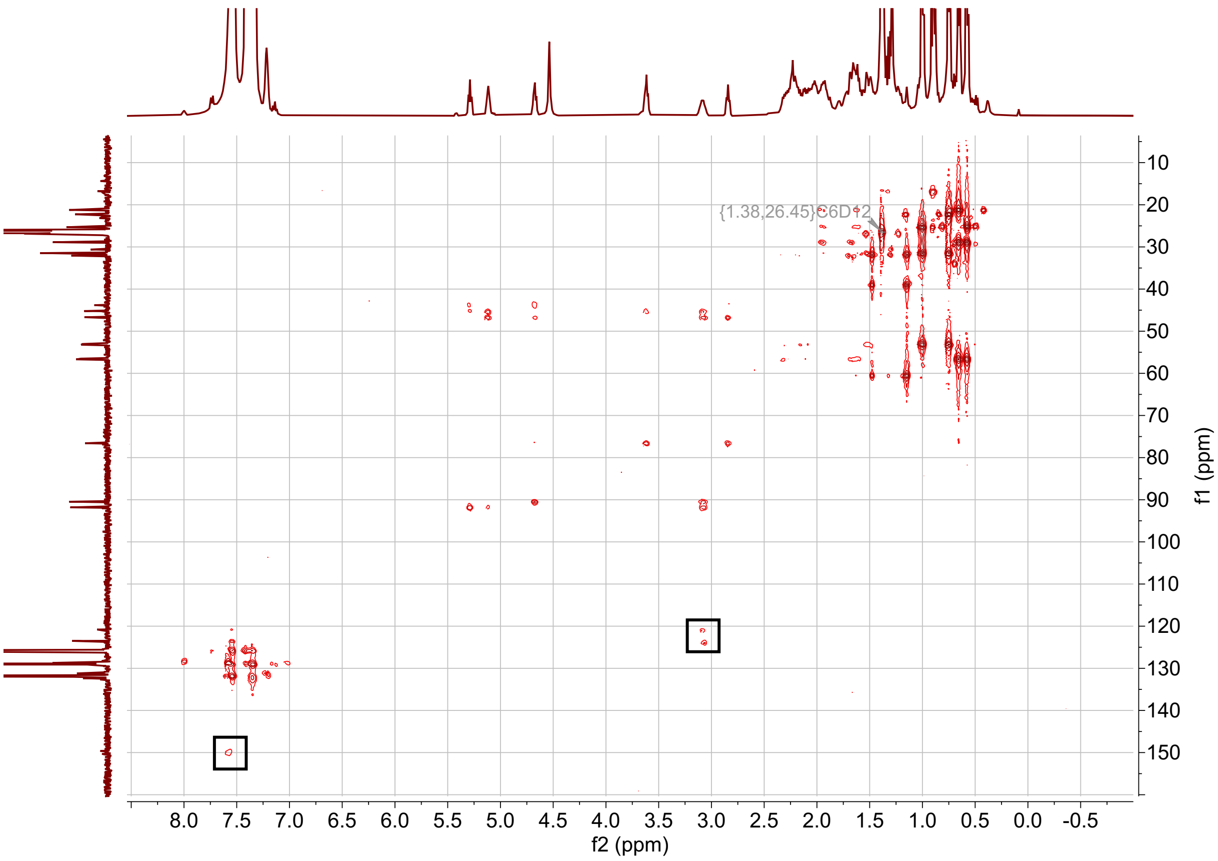

Figure S49. ^{1}H–$^{13}C\{^{1}H\}$ HMBC NMR spectrum (cyclohexane-d_{12}, 23 °C) of **Co1-a** in excess $PhCF_3$. Inset: assignment of quaternary ^{iPr}DuPhos Ar and $CF_{3;ipso}$ resonances.

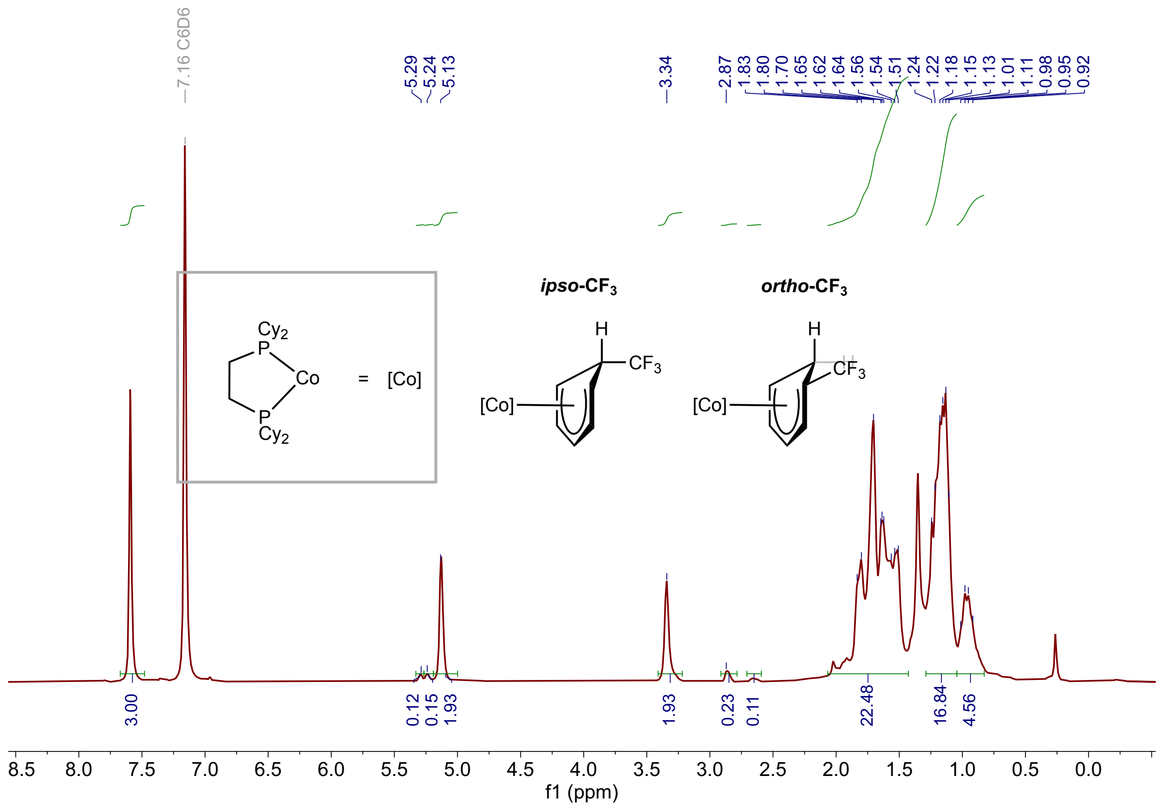

Figure S50. ^{1}H NMR spectrum (400 MHz, benzene-d_6, 23 °C) of isolated **Co2-a** with 1,3,5-tris(trifluoromethyl)benzene internal standard (δ 7.59 ppm) in cyclohexane-d_{12} (δ1.34 ppm).

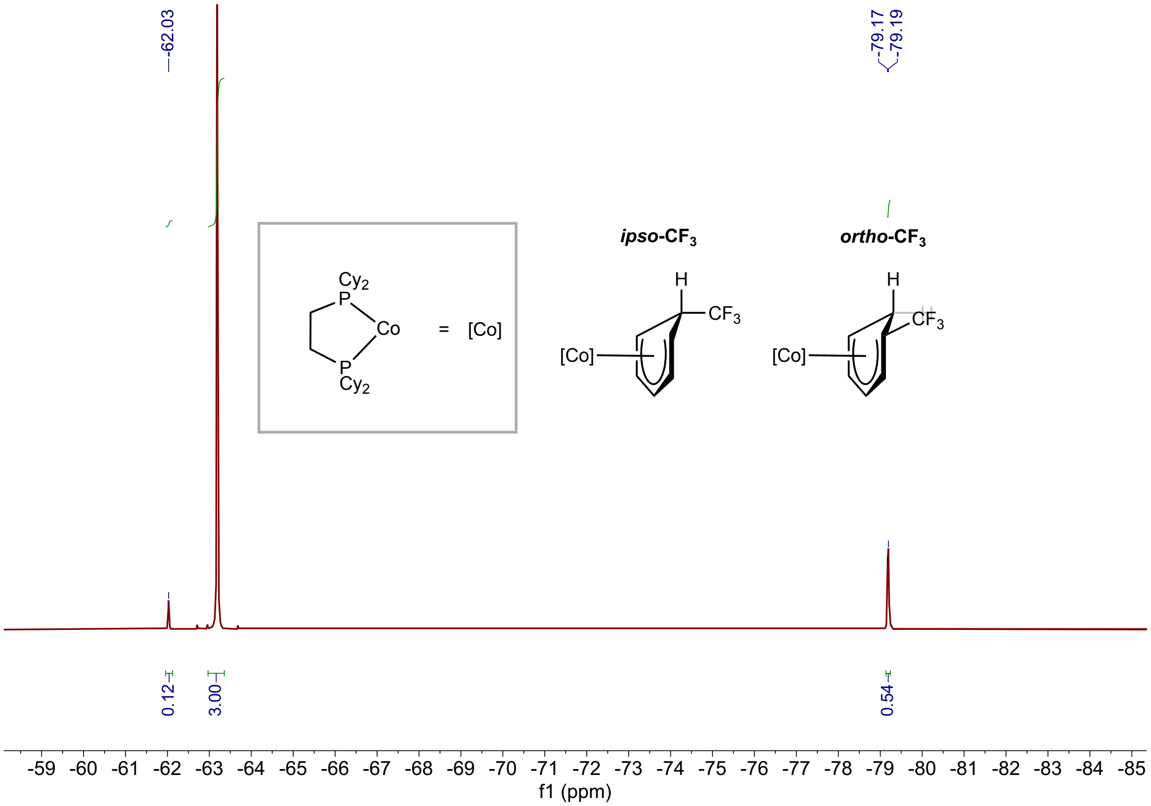

Figure S51. ^{19}F NMR spectrum (376 MHz, benzene-d_6, 23 °C) of isolated **Co2-a** with 1,3,5-tris(trifluoromethyl)benzene internal standard (δ -63.18).

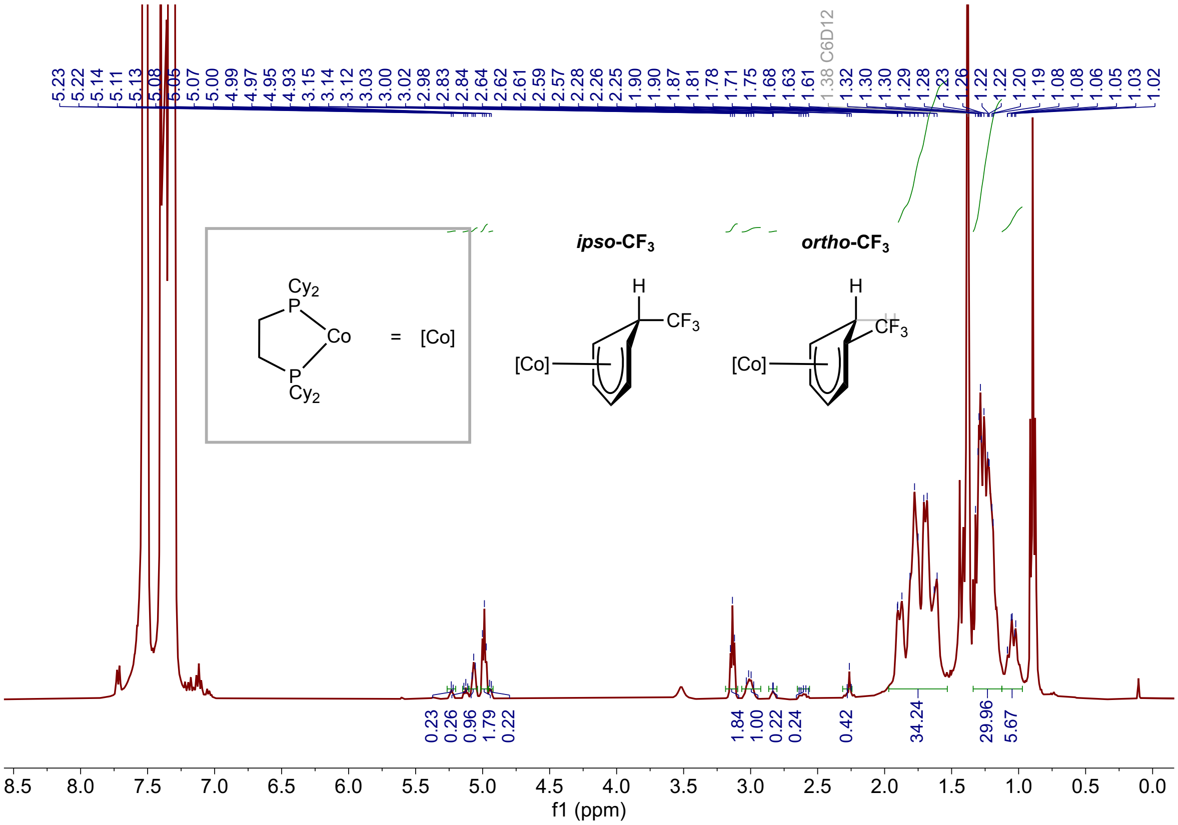

Figure S52. ^{1}H NMR spectrum (400 MHz, cyclohexane-d_{12}, 23 °C) of **Co2-a** in excess $PhCF_3$ and pentane.

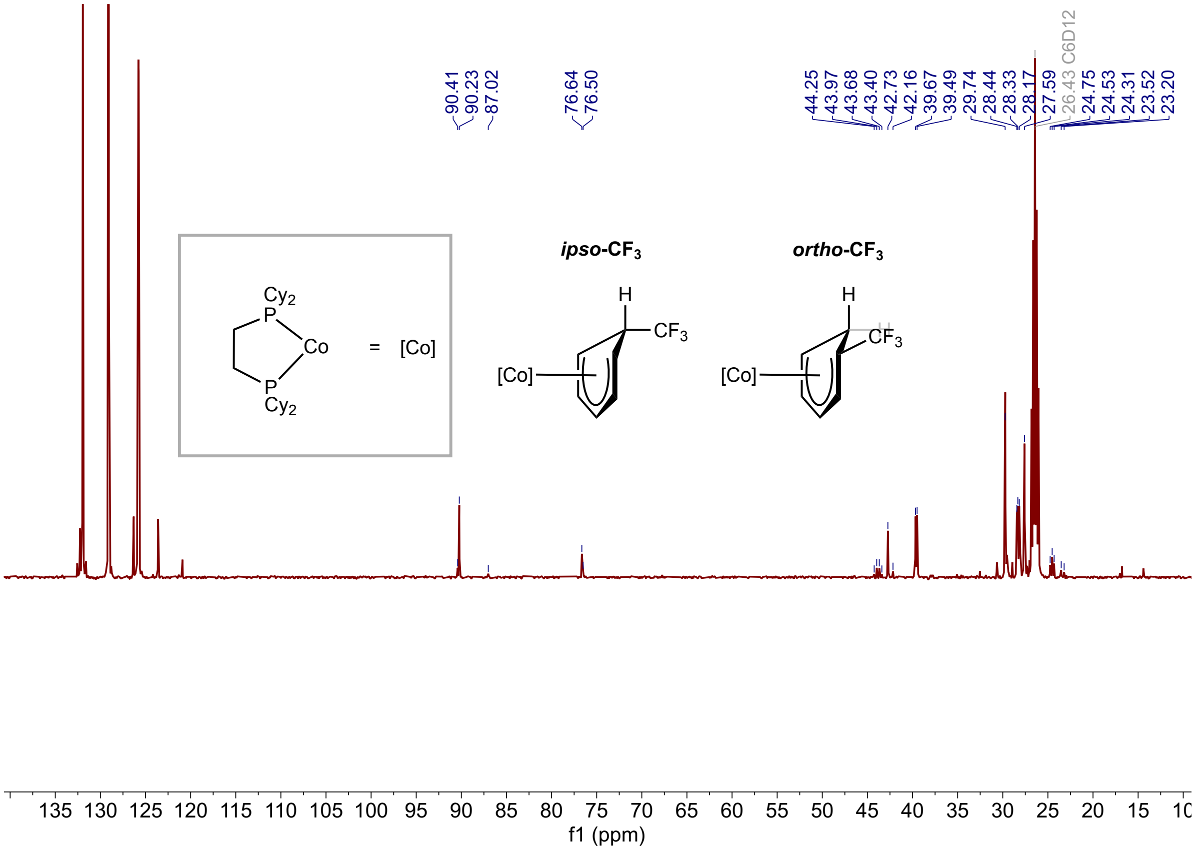

Figure S53. $^{13}C\{^{1}H\}$ NMR spectrum (101 MHz, cyclohexane-d_{12}, 23 °C) of **Co2-a** in excess $PhCF_3$ and pentane.

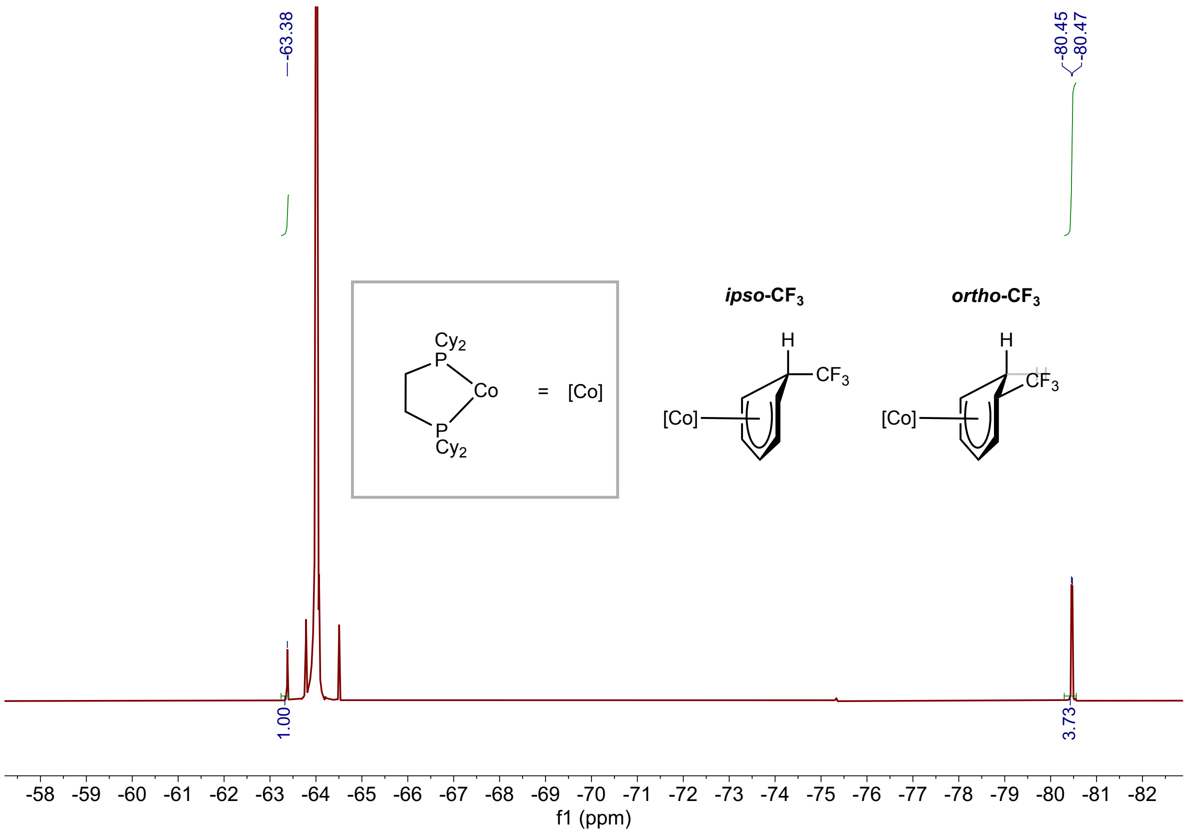

Figure S54. ^{19}F NMR spectrum (376 MHz, cyclohexane-d_{12}, 23 °C) of **Co2-a** in excess $PhCF_3$.

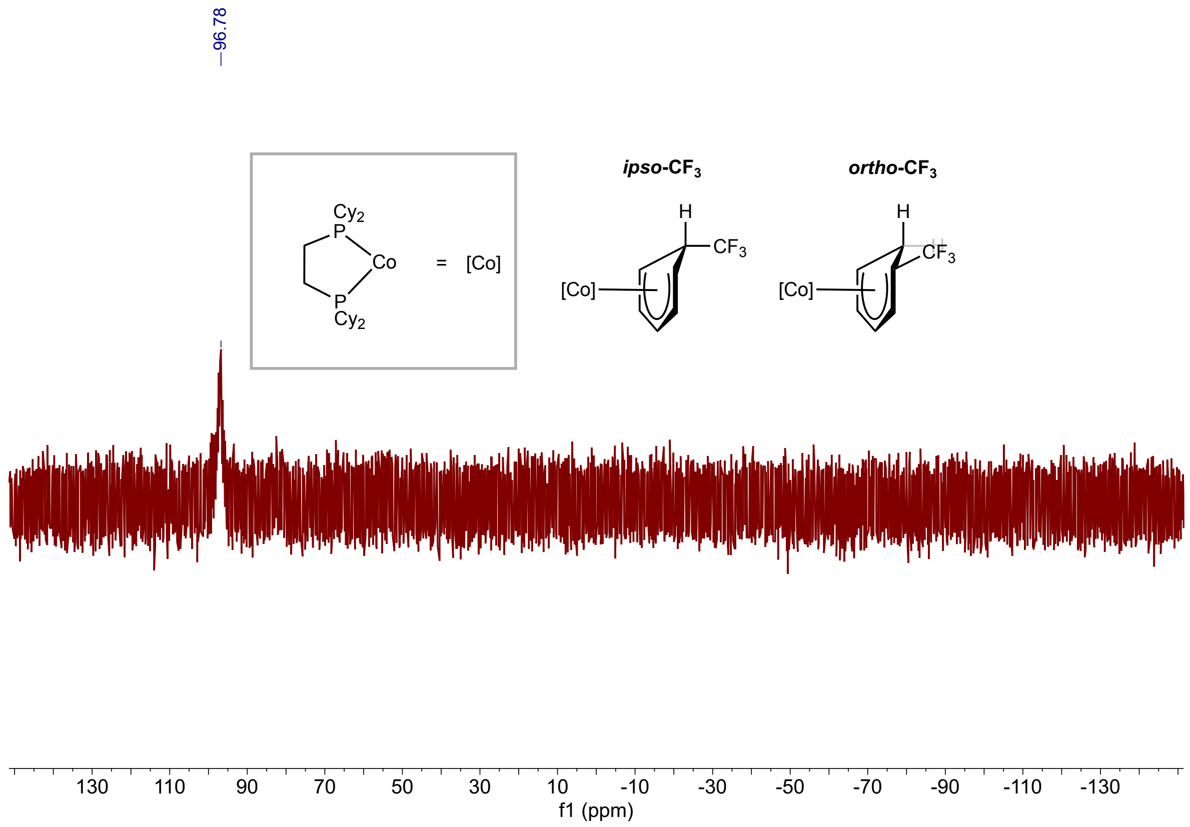

Figure S55. $^{31}P\{^{1}H\}$ NMR spectrum (cyclohexane-d_{12}, 23 °C) of **Co2-a**.

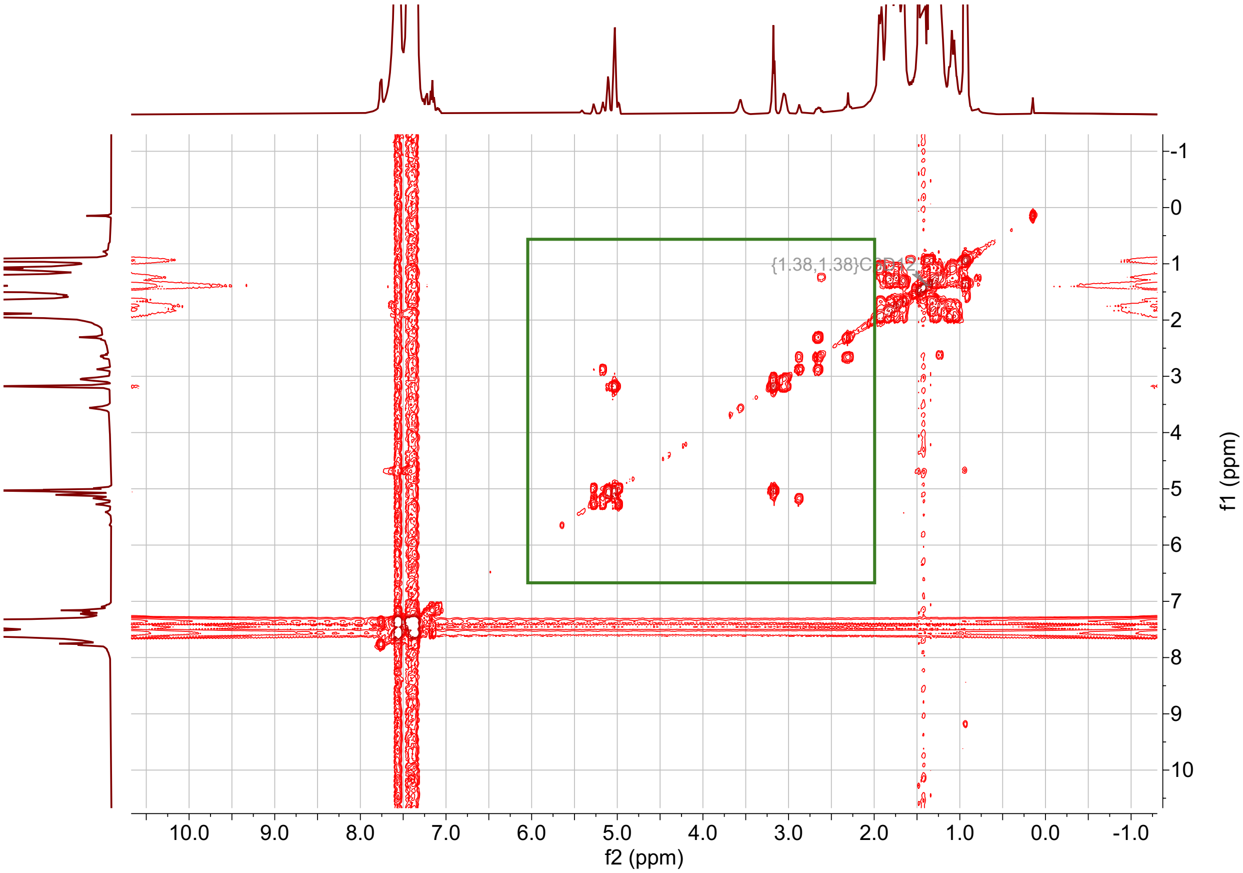

Figure S56. ^{1}H–^{1}H COSY NMR spectrum (cyclohexane-d_{12}, 23 °C) of **Co2-a** in excess $PhCF_3$ and pentane. Inset: ^{1}H–^{1}H correlation between η^{5}-cyclohexadienyl signals.

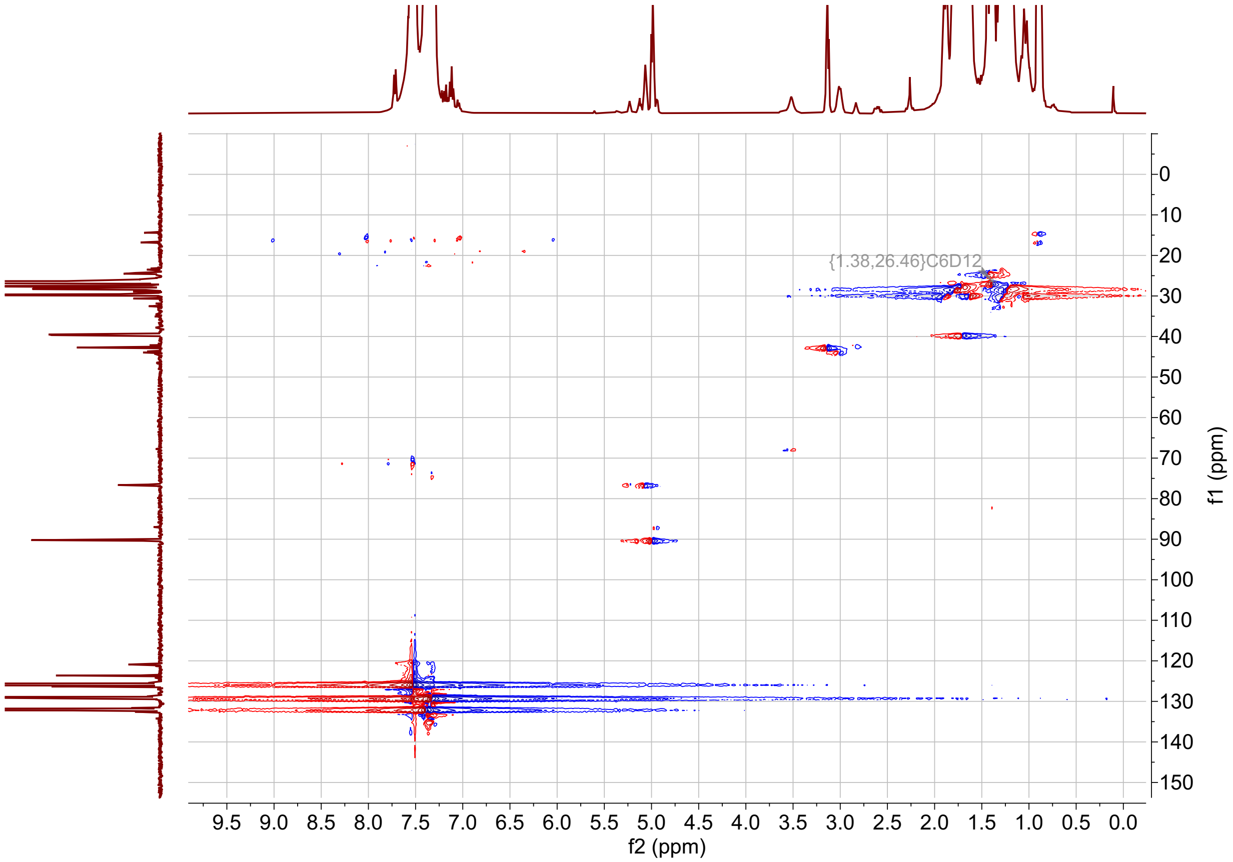

Figure S57. ^{1}H–$^{13}C\{^{1}H\}$ HSQC NMR spectrum (cyclohexane-d_{12}, 23 °C) of **Co2-a** in excess $PhCF_3$ and pentane.

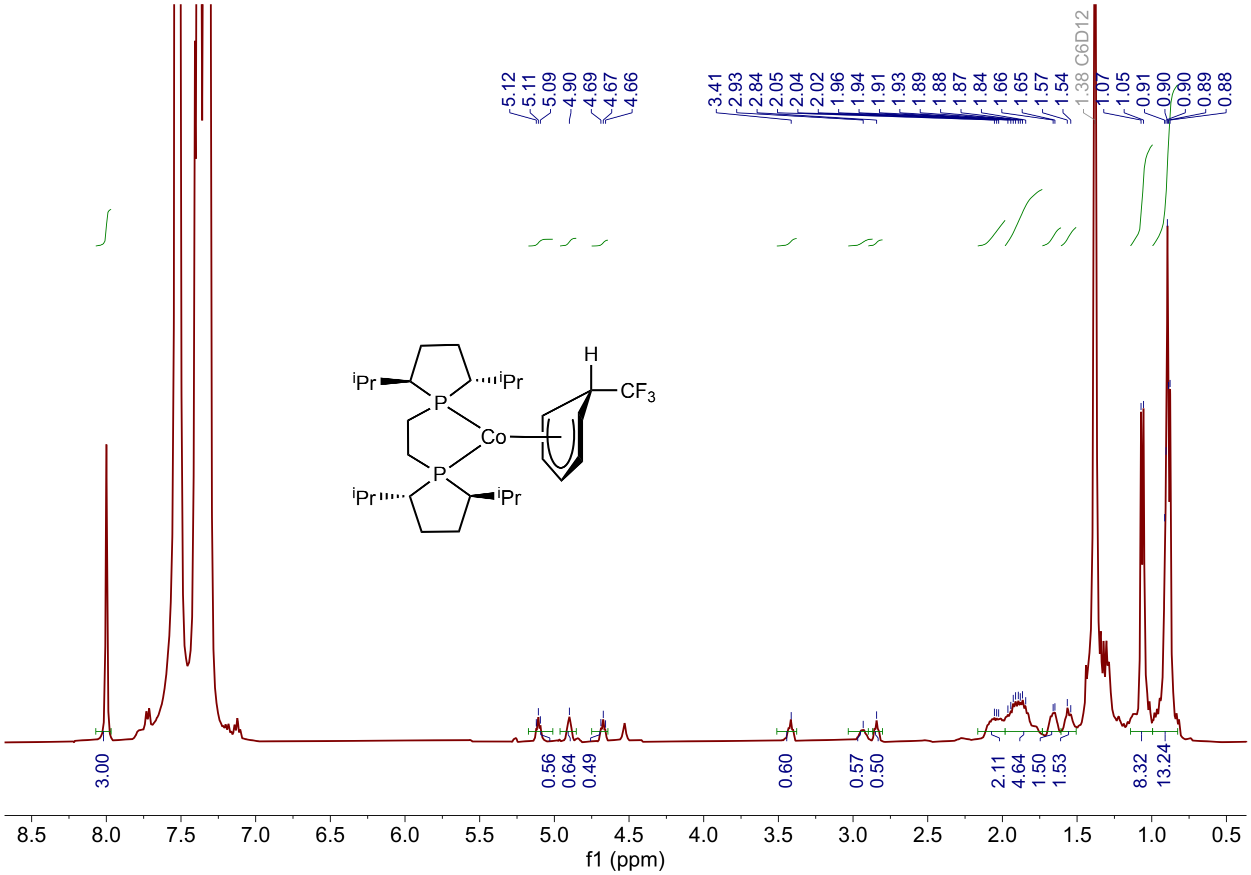

Figure S58. ^{1}H NMR spectrum (cyclohexane-d_{12}, 23 °C) of **Co3-a** with 1,3,5-tris(trifluoromethyl)benzene internal standard (δ 8.03 ppm) and excess $PhCF_3$.

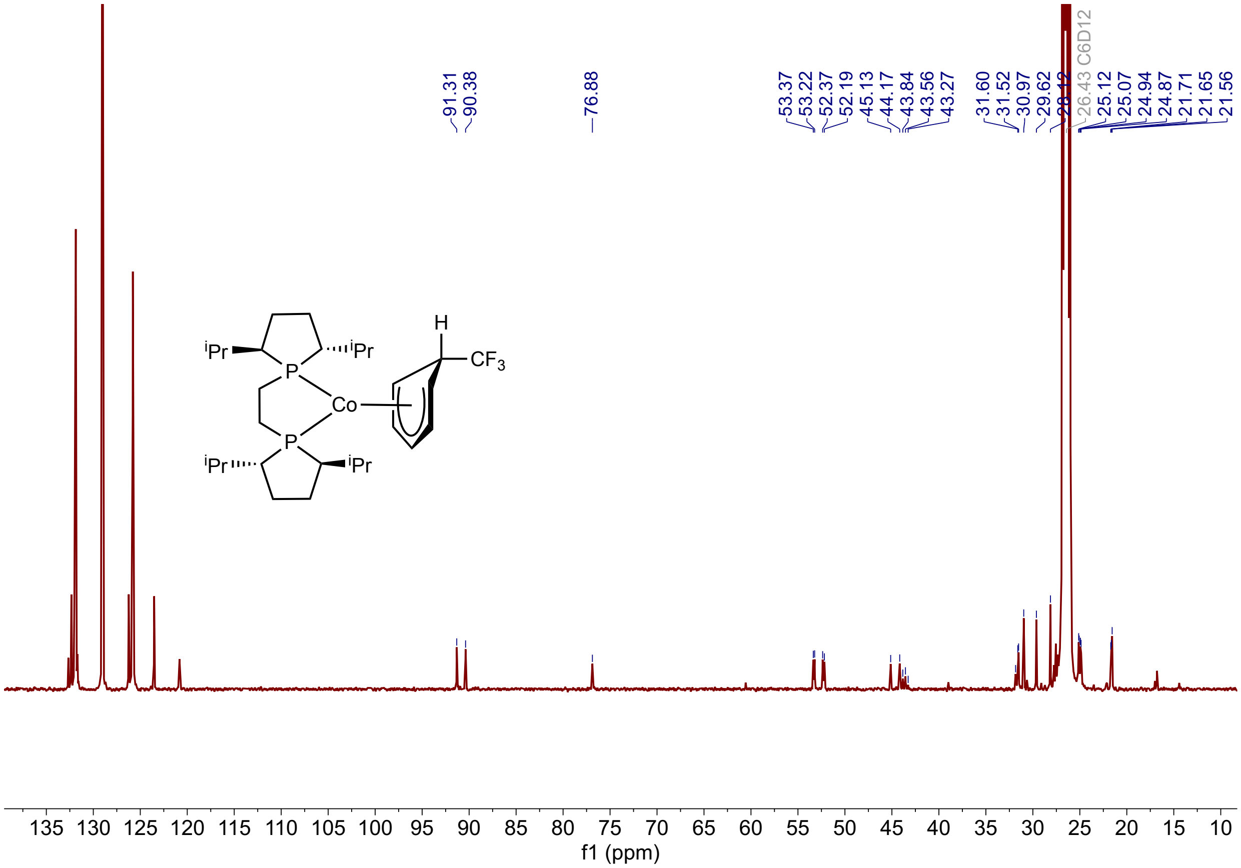

Figure S59. $^{13}C\{^1H\}$ NMR spectrum (101 MHz, cyclohexane-d_{12}, 23 °C) of **Co3-a** excess $PhCF_3$.

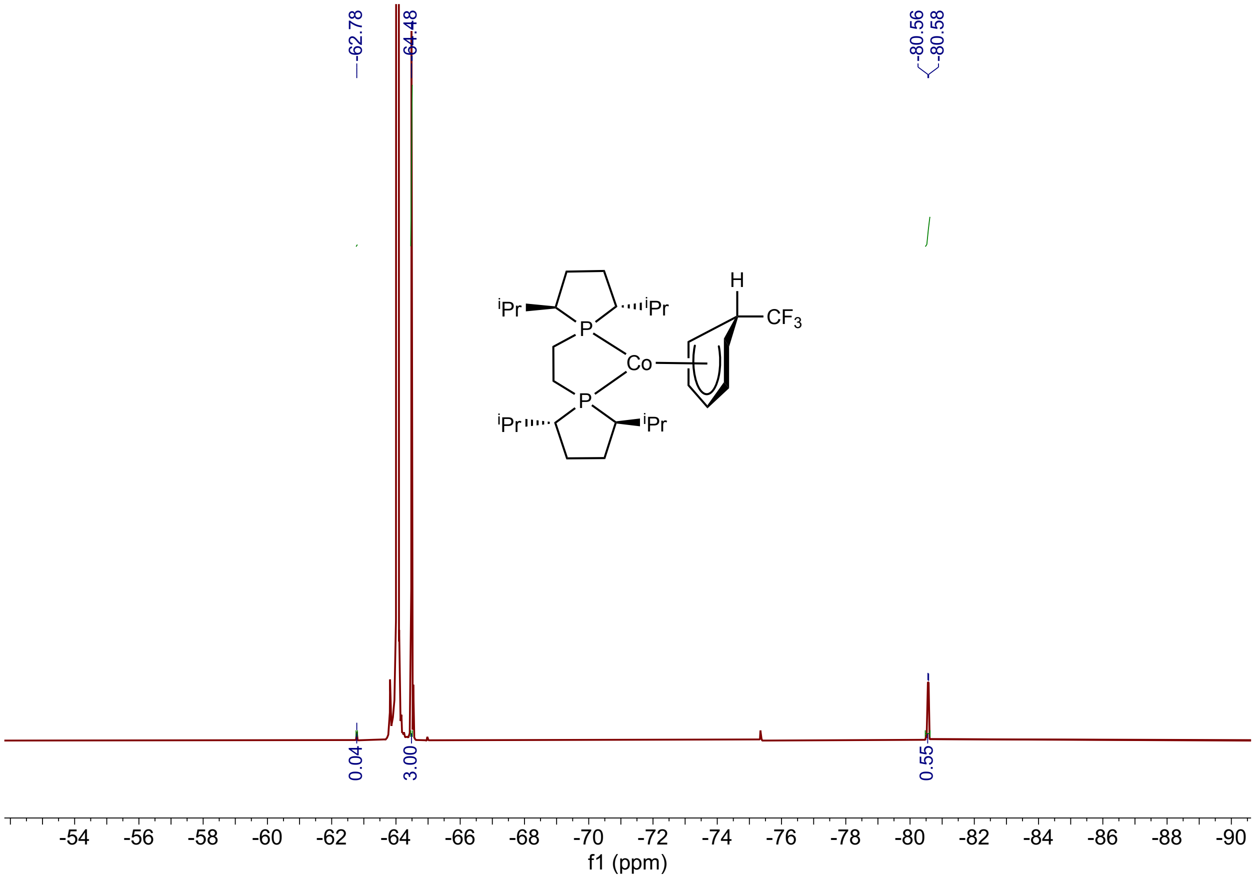

Figure S60. ^{19}F NMR spectrum (376 MHz, cyclohexane-d_{12}, 23 °C) of **Co3-a** with 1,3,5-tris(trifluoromethyl)benzene internal standard (δ -64.48 ppm).

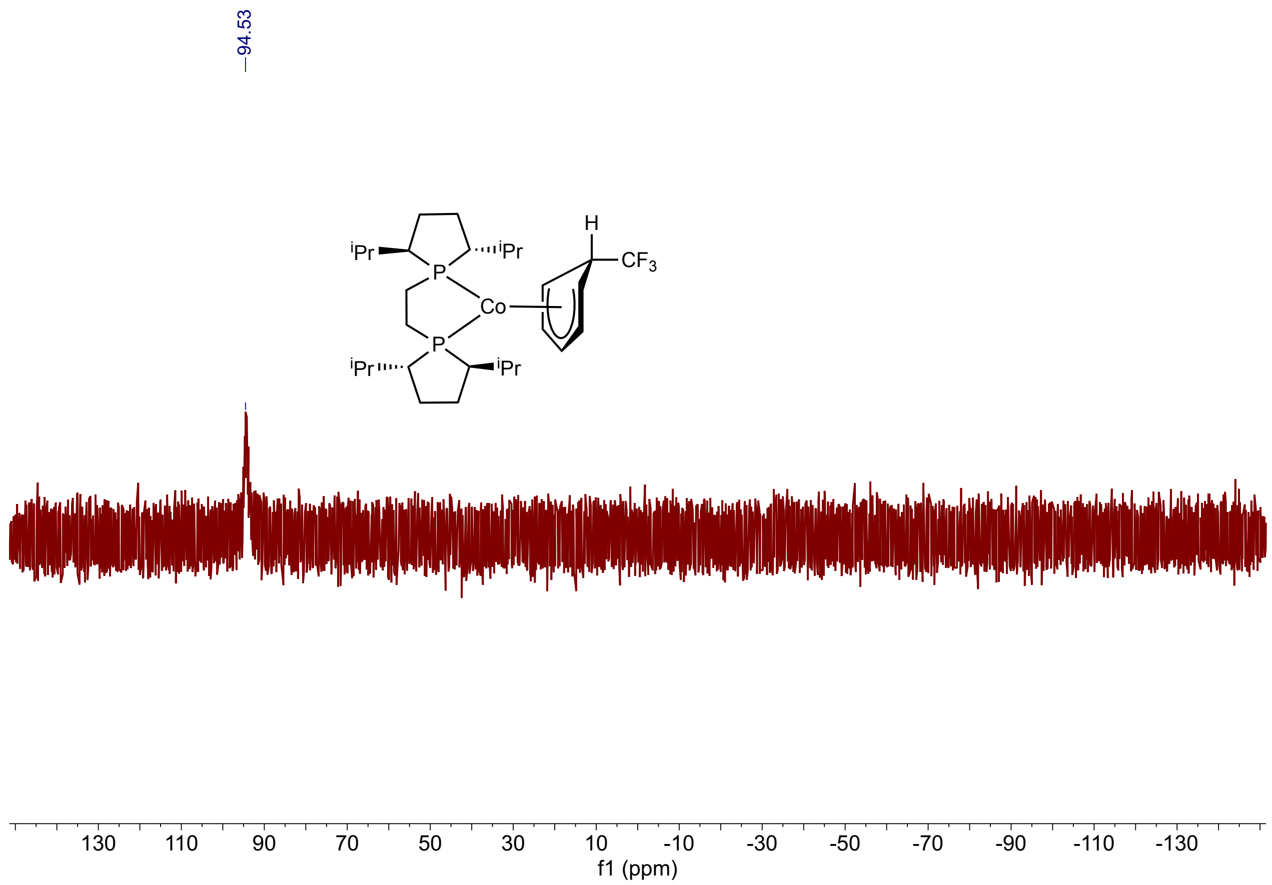

Figure S61. $^{31}P\{^{1}H\}$ NMR spectrum (162 MHz, cyclohexane-d_{12}, 23 °C) of **Co3-a**.

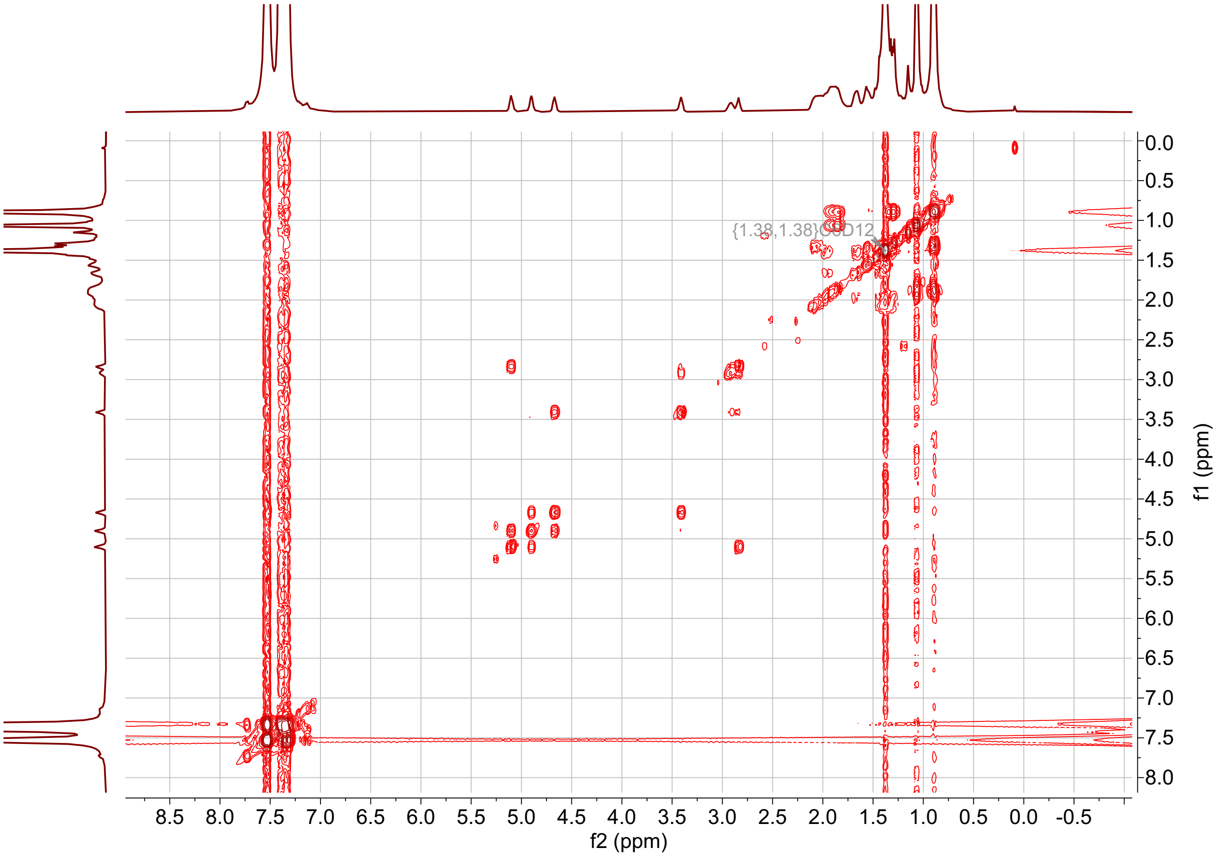

Figure S62. ^{1}H–^{1}H COSY NMR spectrum (cyclohexane-d_{12}, 23 °C) of **Co3-a** in excess $PhCF_3$.

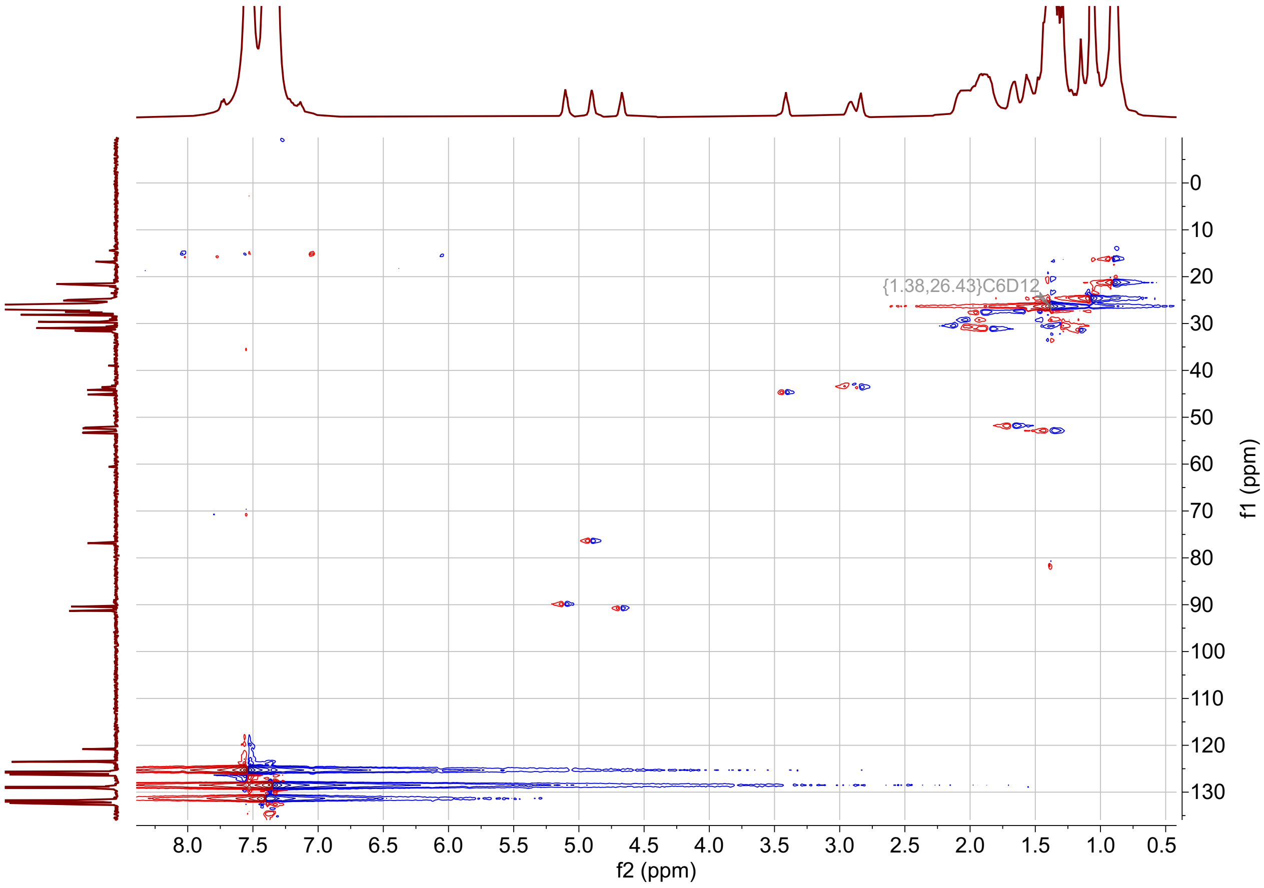

Figure S63. ^{1}H–$^{13}C\{^{1}H\}$ HSQC NMR spectrum (cyclohexane-d_{12}, 23 °C) of **Co3-a** in excess $PhCF_3$.

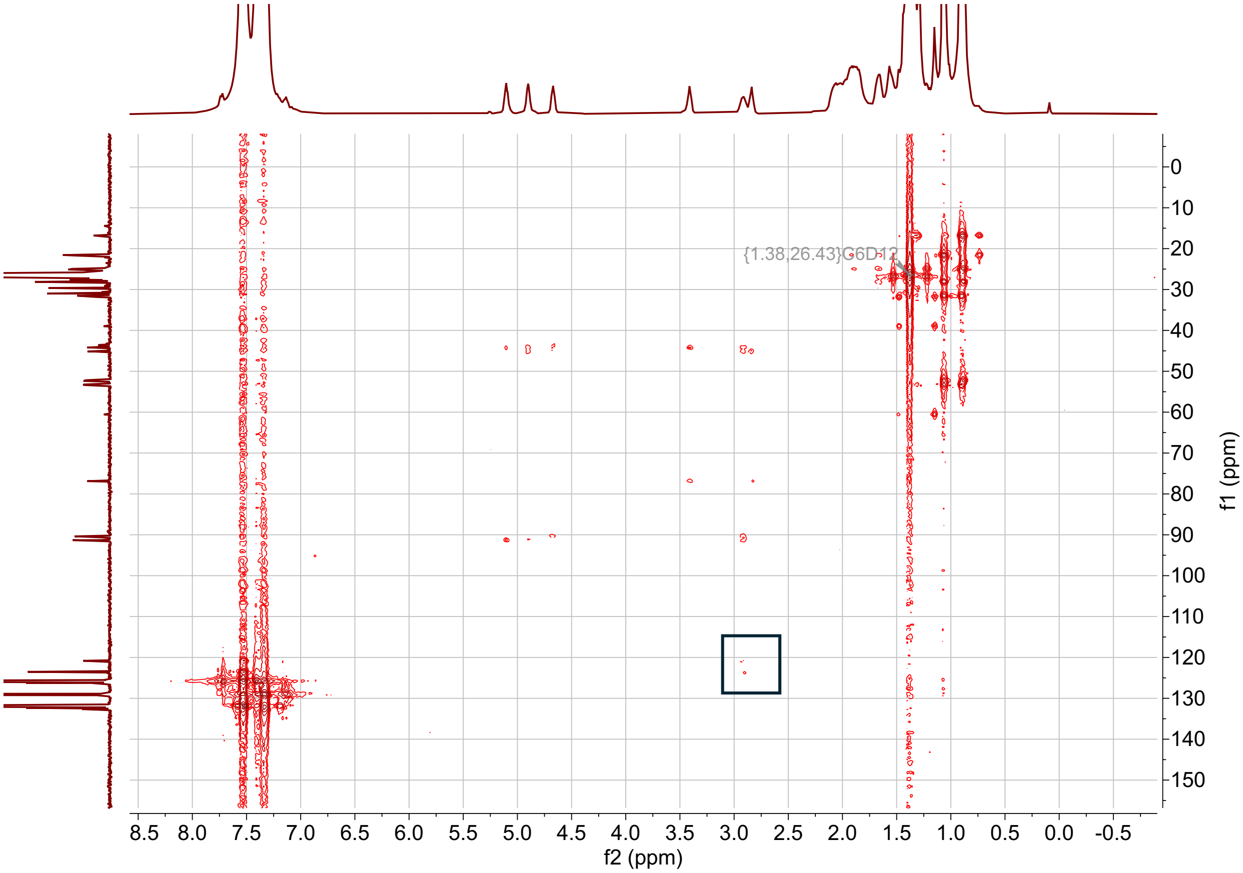

Figure S64. ^{1}H–$^{13}C\{^{1}H\}$ HMBC NMR spectrum (cyclohexane-d_{12}, 23 °C) of **Co3-a** in excess $PhCF_3$. Inset: assignment of quaternary carbons.

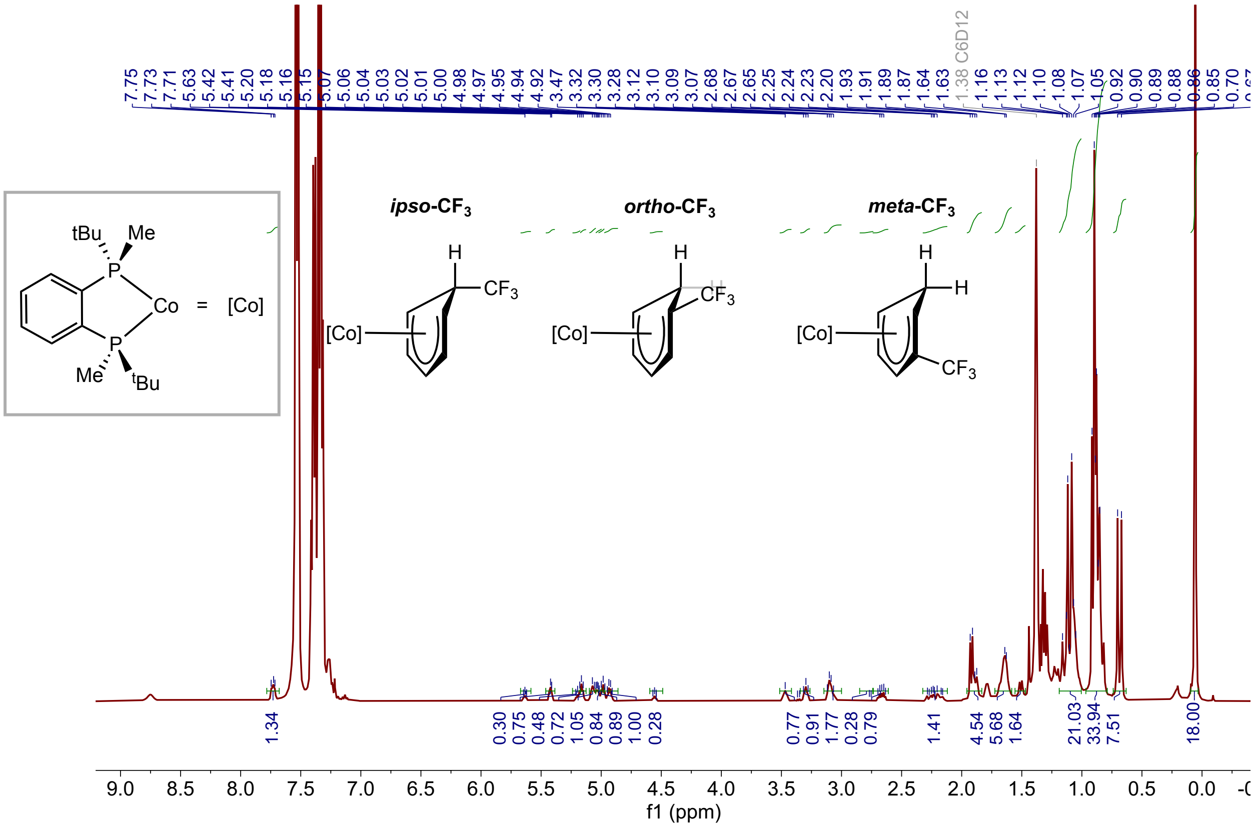

Figure S65. ^{1}H NMR spectrum (400 MHz, cyclohexane-d_{12}, 23 °C) of **Co4-a** with a HMDSO (δ 0.10 ppm) internal standard in excess $PhCF_3$ at 1 h.

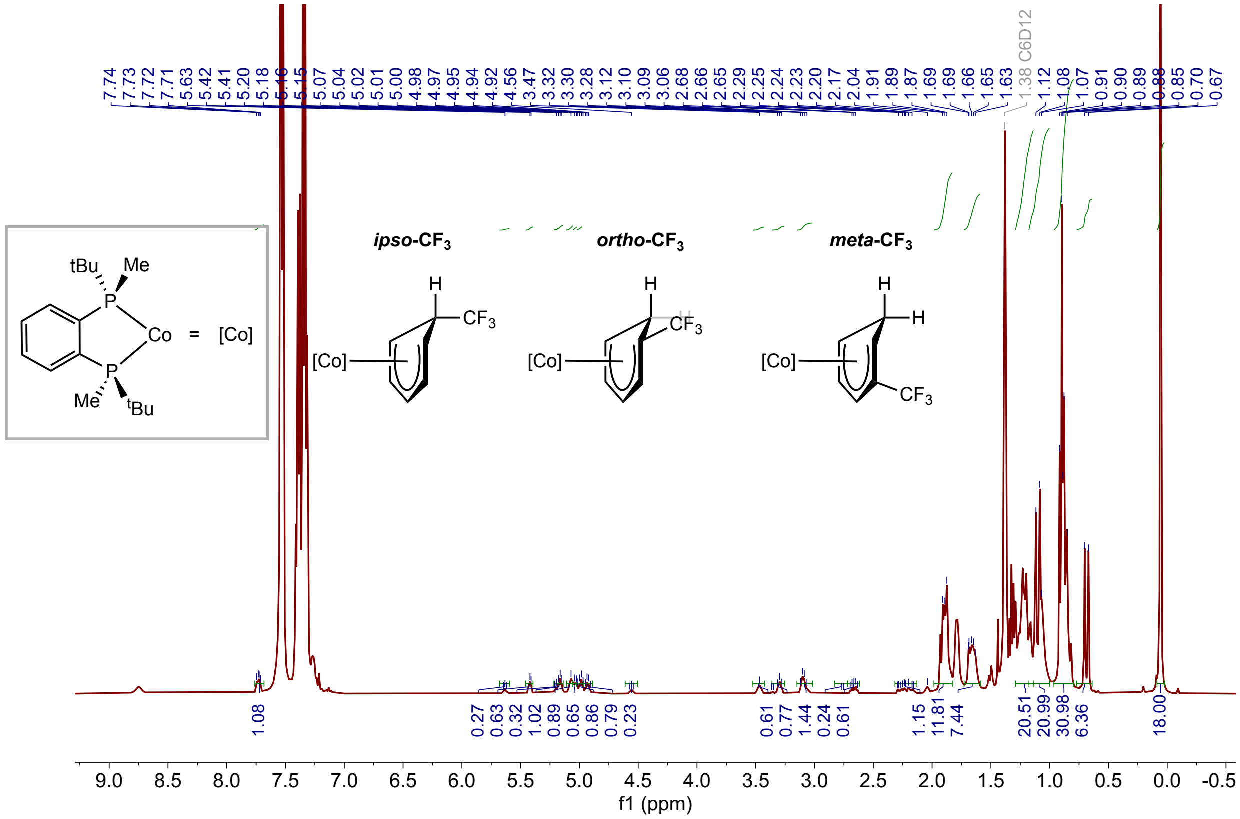

Figure S66. ^{1}H NMR spectrum (400 MHz, cyclohexane-d_{12}, 23 °C) of **Co4-a** with a HMDSO (δ 0.10 ppm) internal standard in excess $PhCF_3$ at 24 h.

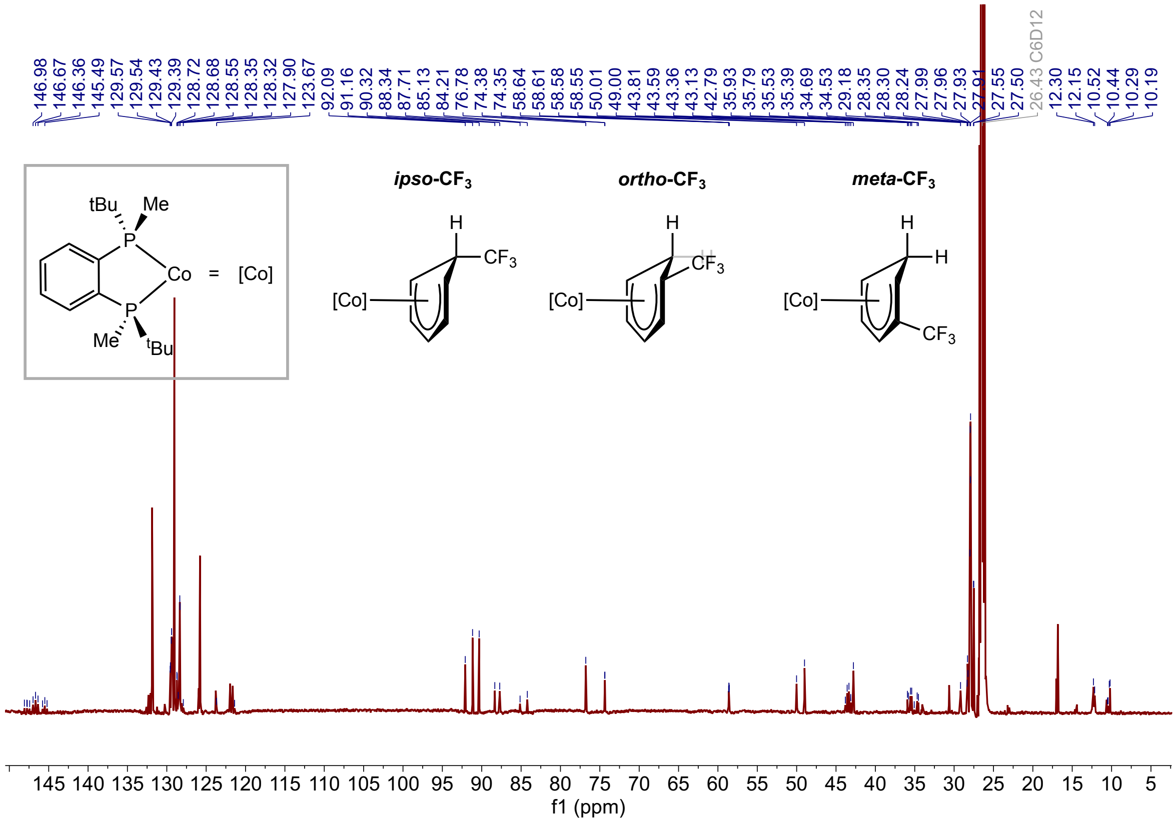

Figure S67. $^{13}C\{^{1}H\}$ NMR spectrum (101 MHz, cyclohexane-d_{12}, 23 °C) of **Co4-a** in excess $PhCF_3$.

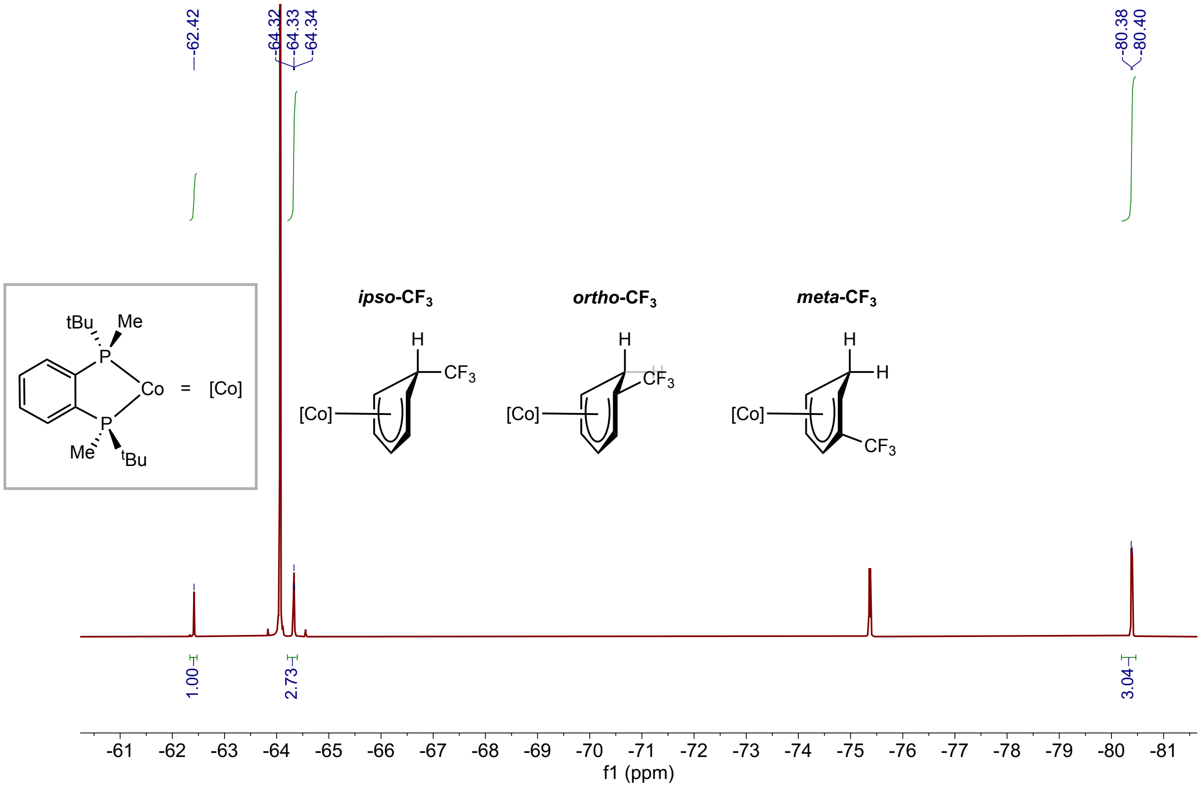

Figure S68. ^{19}F NMR spectrum (376 MHz, cyclohexane-d_{12}, 23 °C) of **Co4-a** in excess $PhCF_3$.

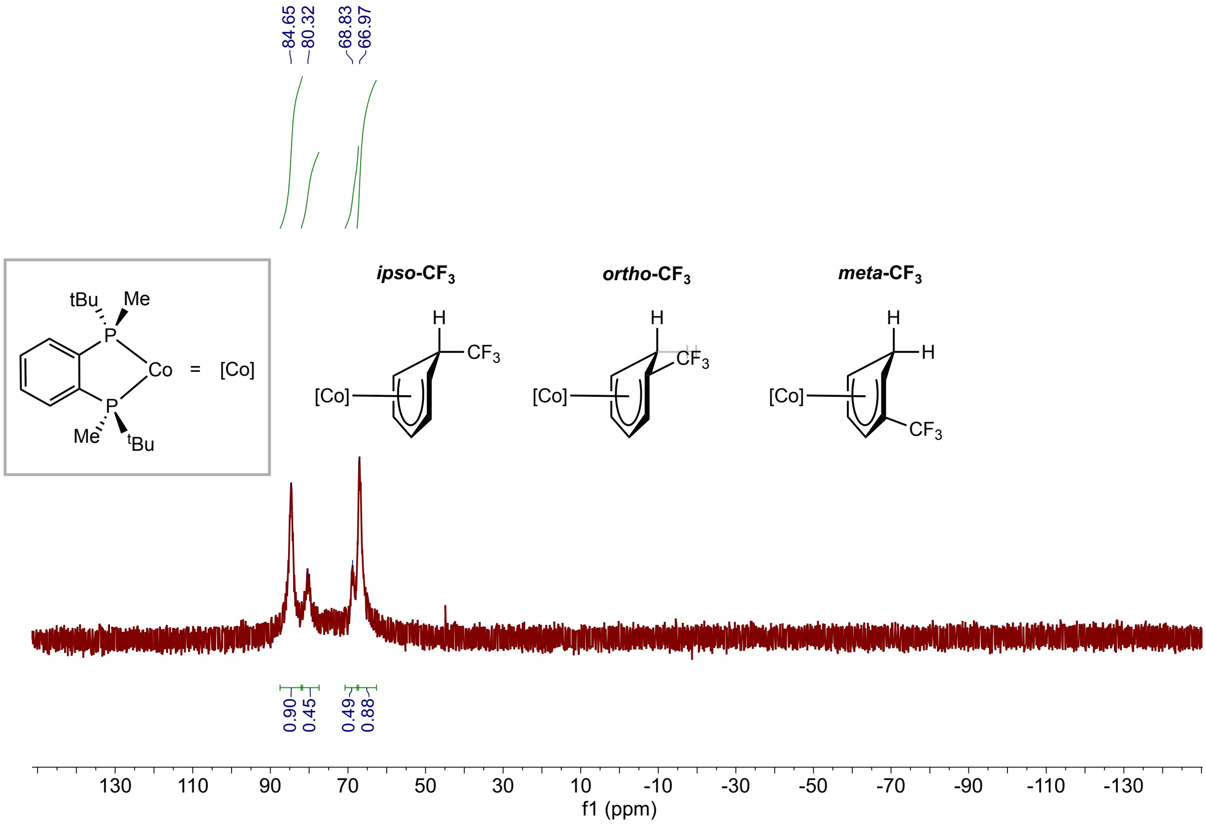

Figure S69. $^{31}P\{^1H\}$ NMR spectrum (162 MHz, cyclohexane-d_{12}, 23 °C) of **Co4-a**.

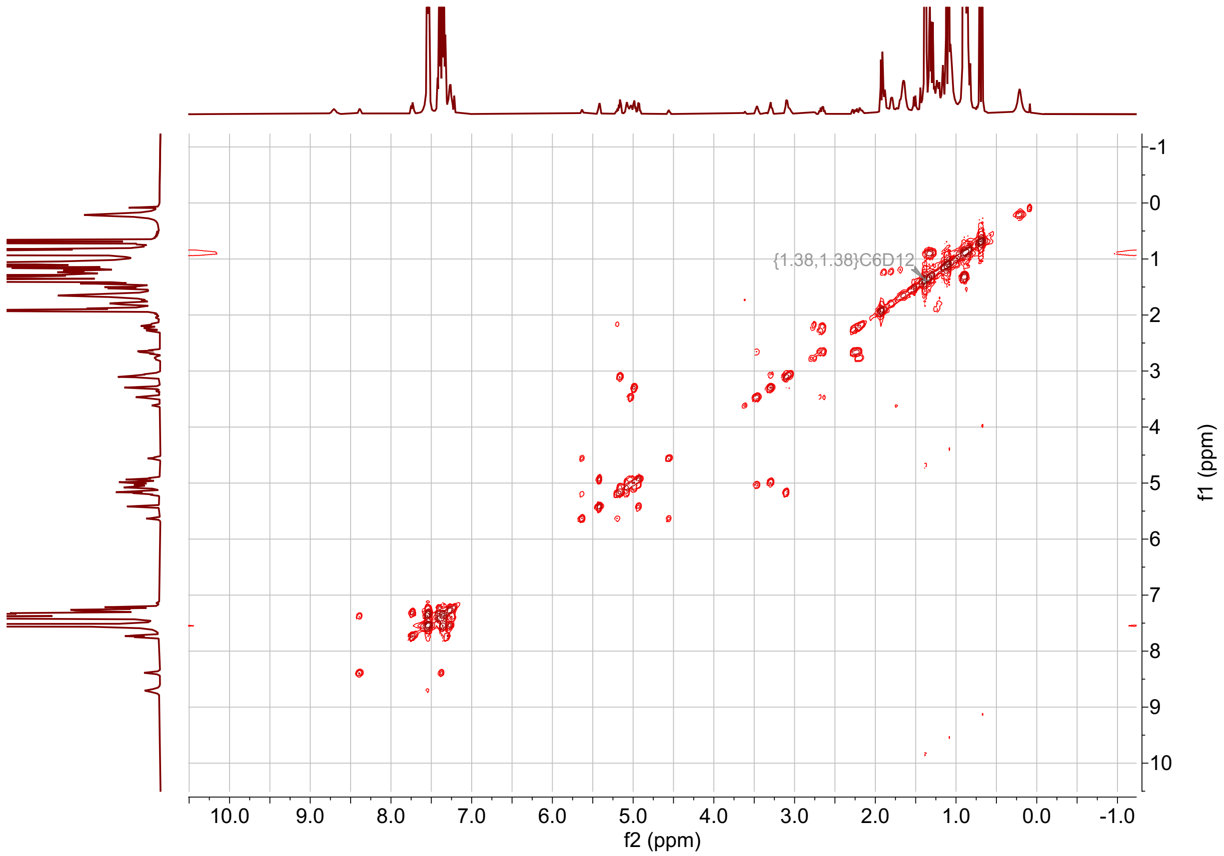

Figure S70. $^1H–^1H$ COSY NMR spectrum (cyclohexane-d_{12}, 23 °C) of **Co4-a** in excess $PhCF_3$.

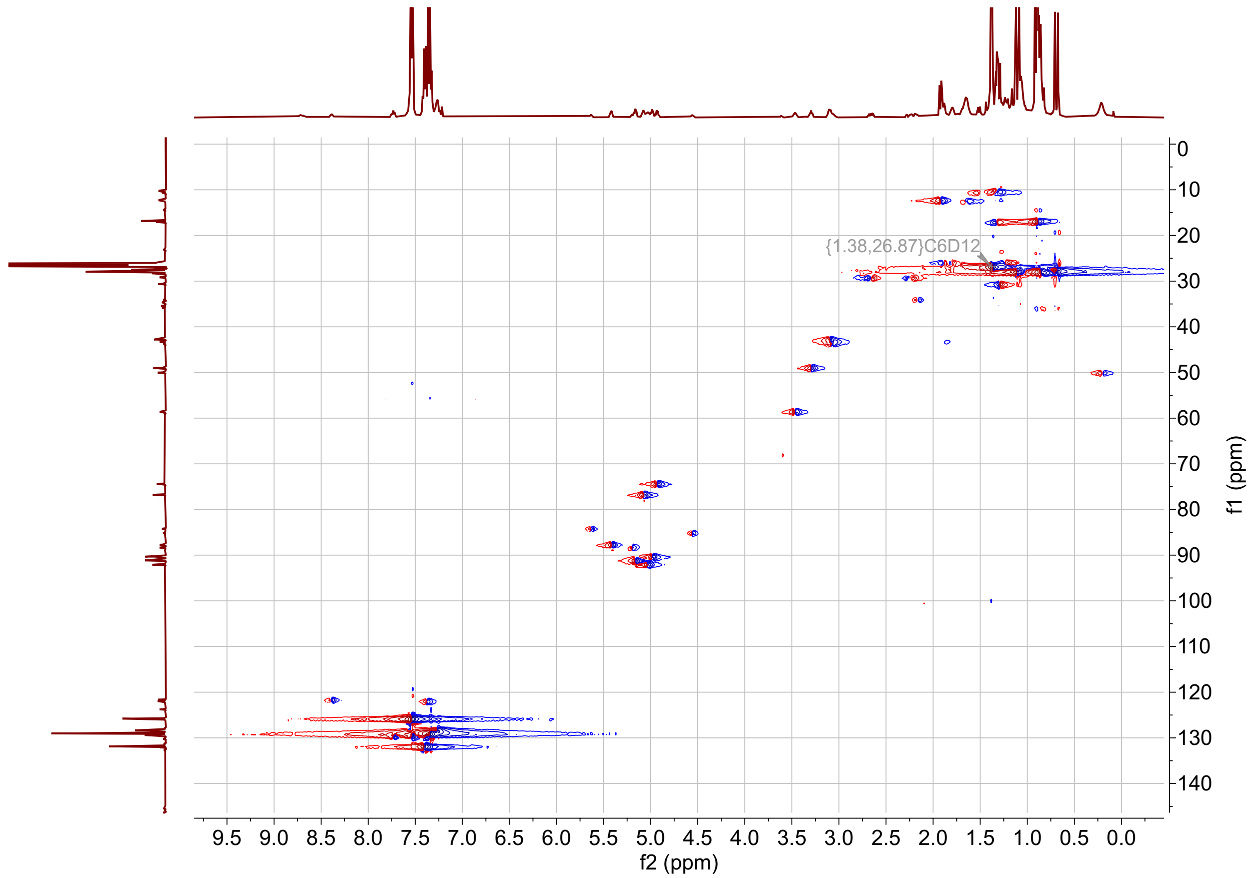

Figure S71. ^{1}H–^{13}C{^{1}H} HSQC NMR spectrum (cyclohexane-d_{12}, 23 °C) of **Co4-a** in excess PhCF$_3$.

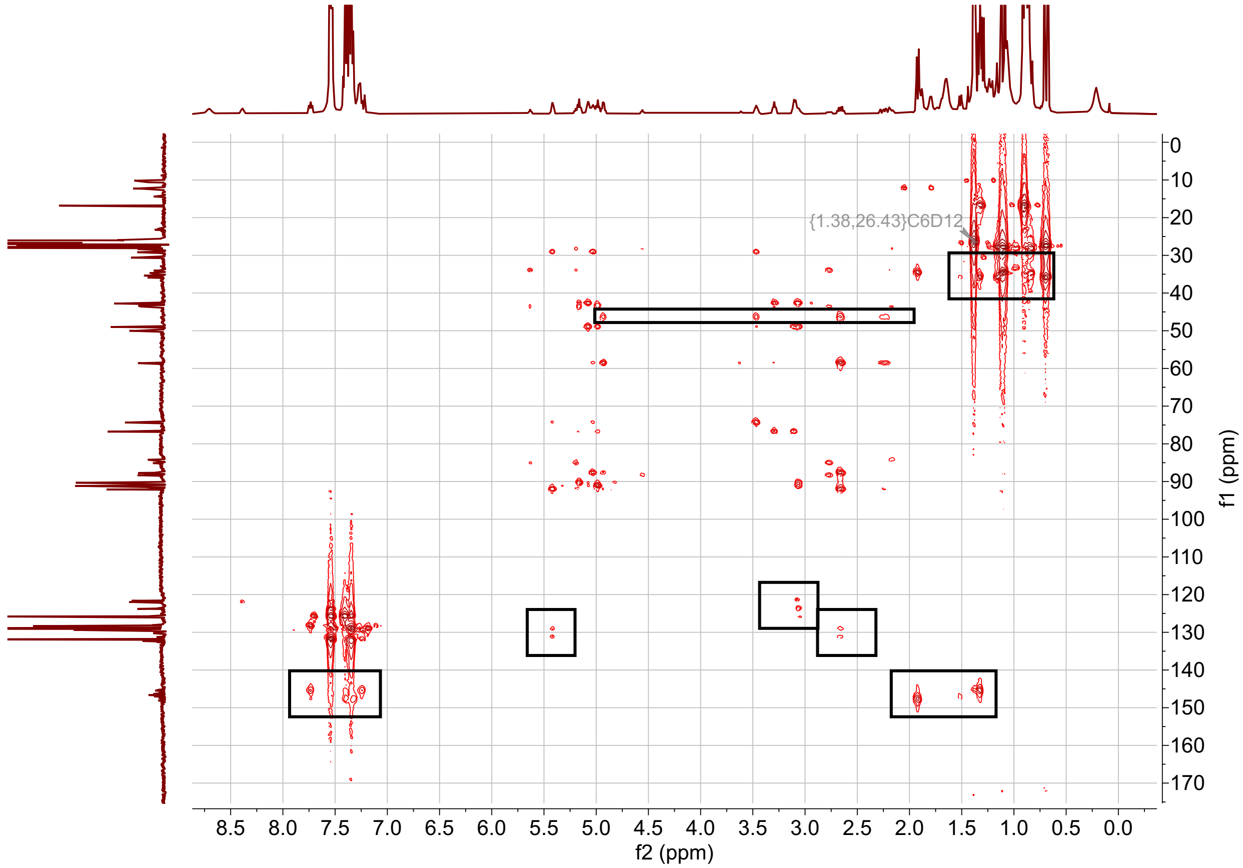

Figure S72. ^{1}H–$^{13}C\{^{1}H\}$ HMBC NMR spectrum (cyclohexane-*d*$_{12}$, 23 °C) of **Co4-a** in excess $PhCF_3$. Inset: assignment of quaternary carbon resonances.

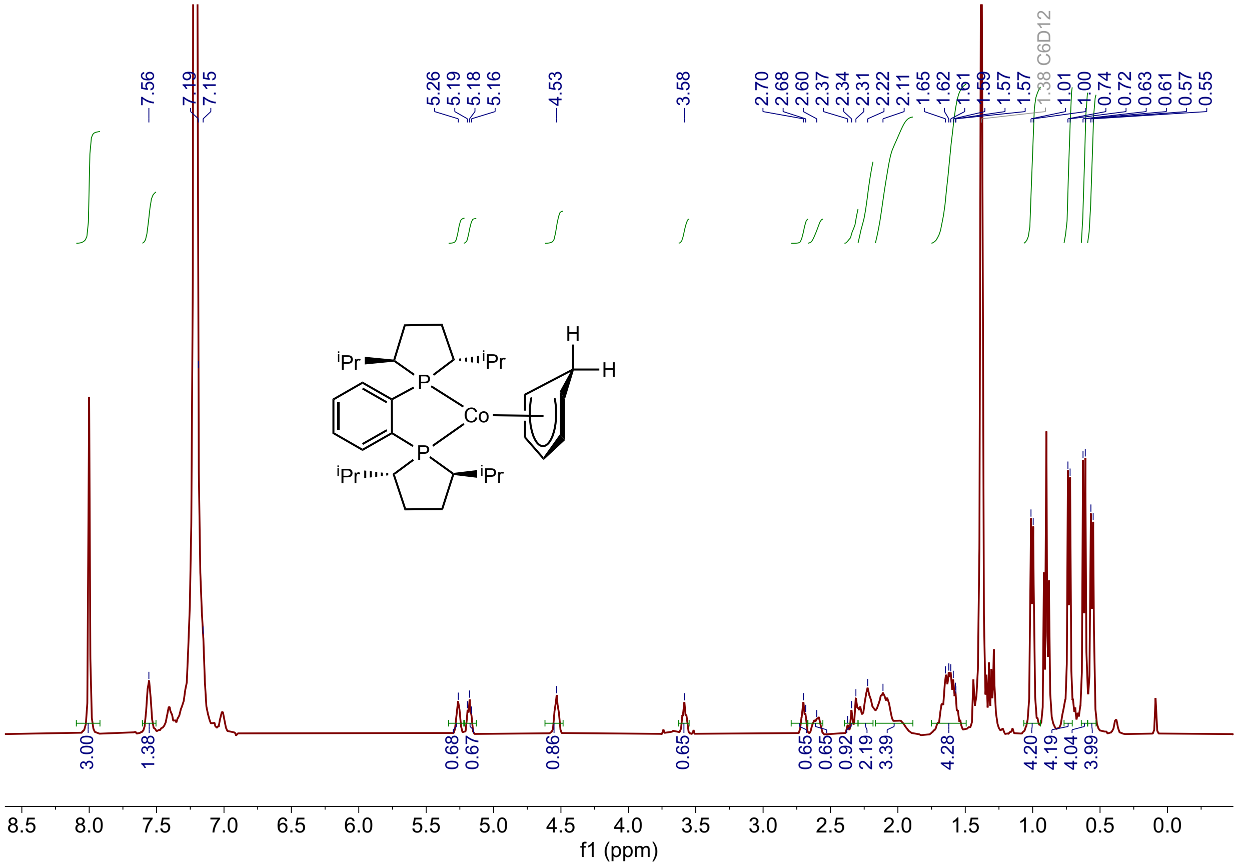

Figure S73. ^{1}H NMR spectrum (400 MHz, cyclohexane-d_{12}, 23 °C) of **Co1-b** with 1,3,5-tris(trifluoromethyl)benzene internal standard (δ 8.03 ppm) and excess PhH.

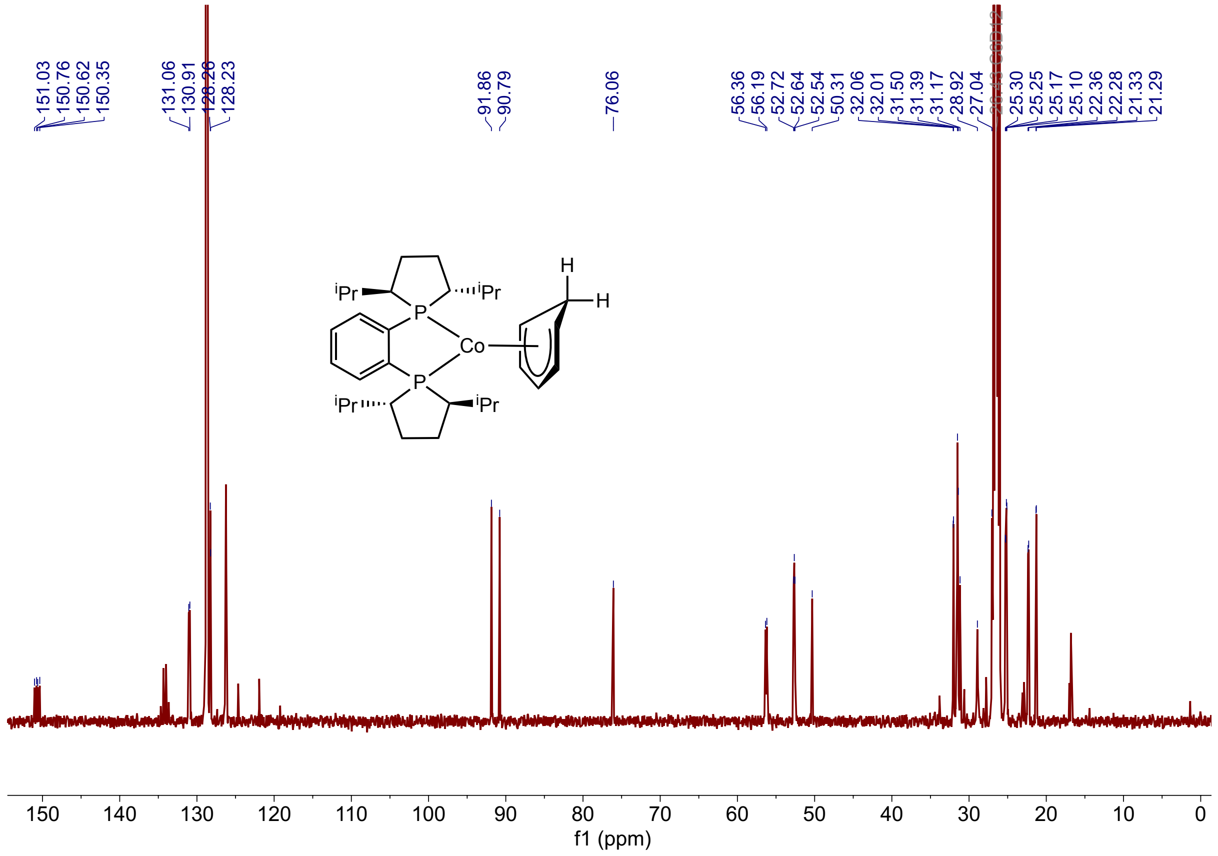

Figure S74. $^{13}C\{^{1}H\}$ NMR spectrum (101 MHz, cyclohexane-d_{12}, 23 °C) of **Co1-b** with 1,3,5-tris(trifluoromethyl)benzene and excess PhH.

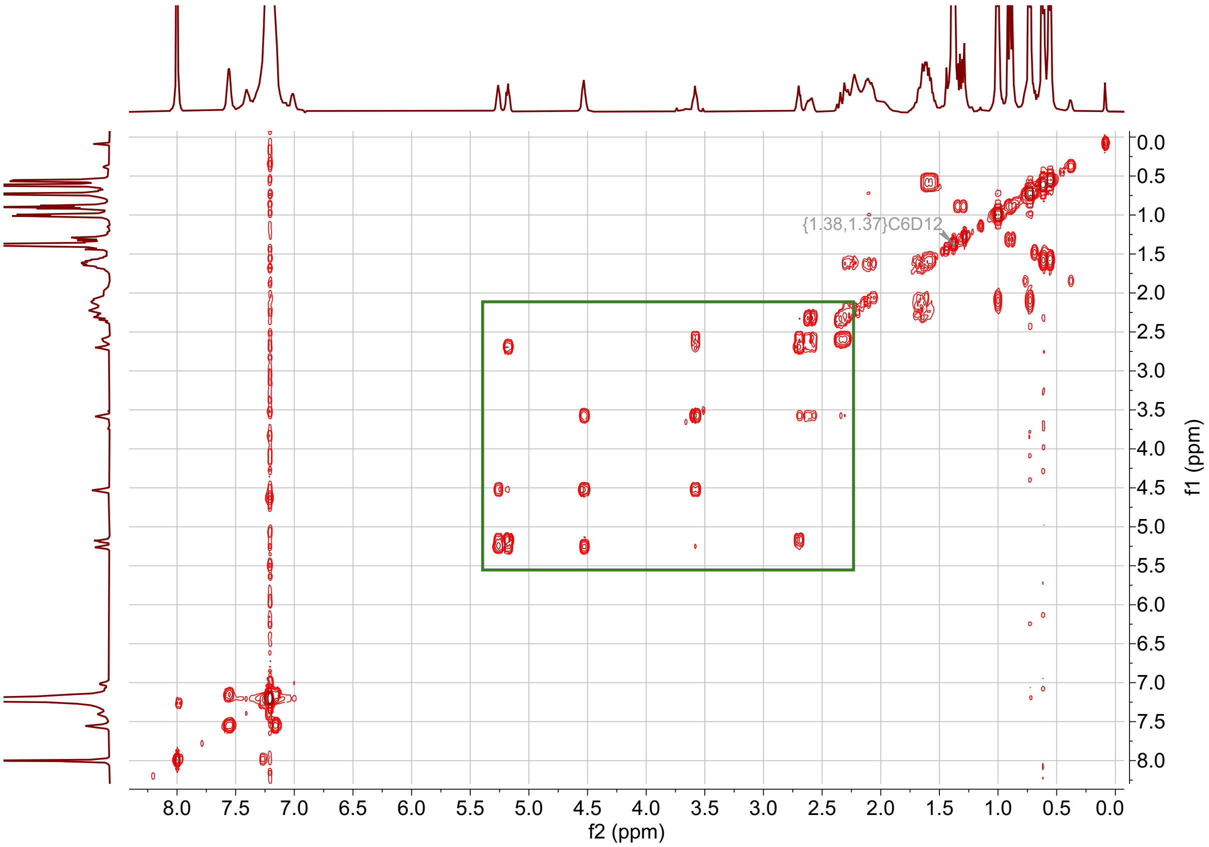

Figure S75. ${}^{1}H$–${}^{1}H$ COSY NMR spectrum (cyclohexane-d_{12}, 23 °C) of **Co1-b** with 1,3,5-tris(trifluoromethyl)benzene and excess PhH. Inset: ${}^{1}H$–${}^{1}H$ correlation between η^5-cyclohexadienyl signals.

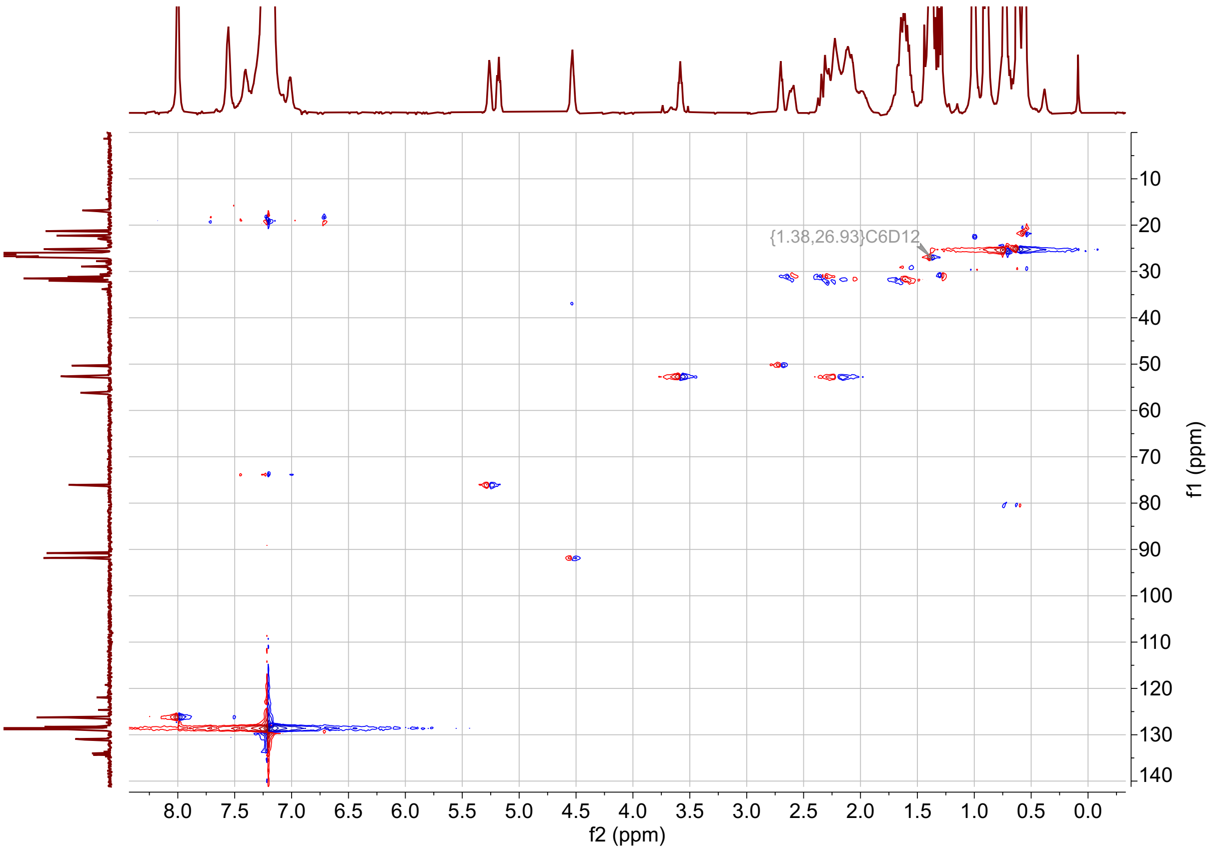

Figure S76. ^{1}H–$^{13}C\{^{1}H\}$ HSQC NMR spectrum (cyclohexane-d_{12}, 23 °C) of **Co1-b** with 1,3,5-tris(trifluoromethyl)benzene internal standard and excess PhH.

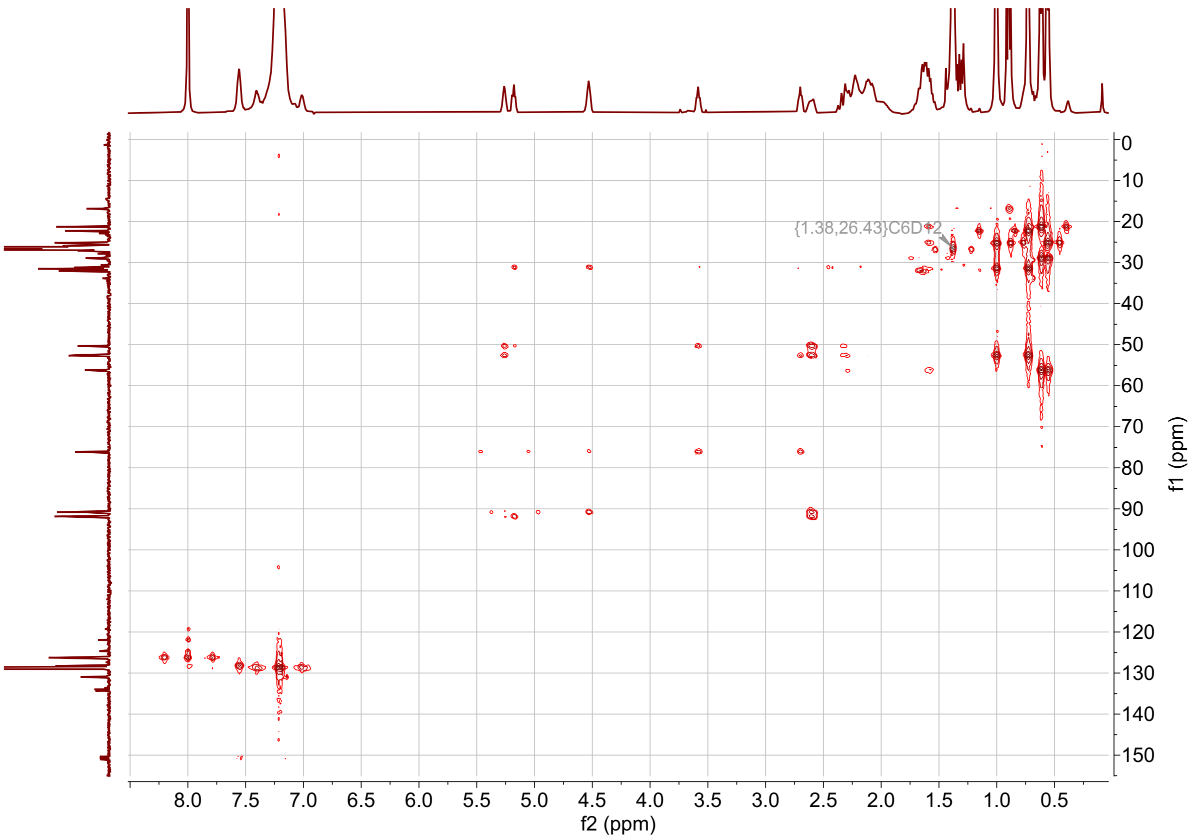

Figure S77. ^{1}H–^{13}C{^{1}H} HMBC NMR spectrum (cyclohexane-d_{12}, 23 °C) of **Co1-b** with 1,3,5-tris(trifluoromethyl)benzene internal standard and excess PhH.

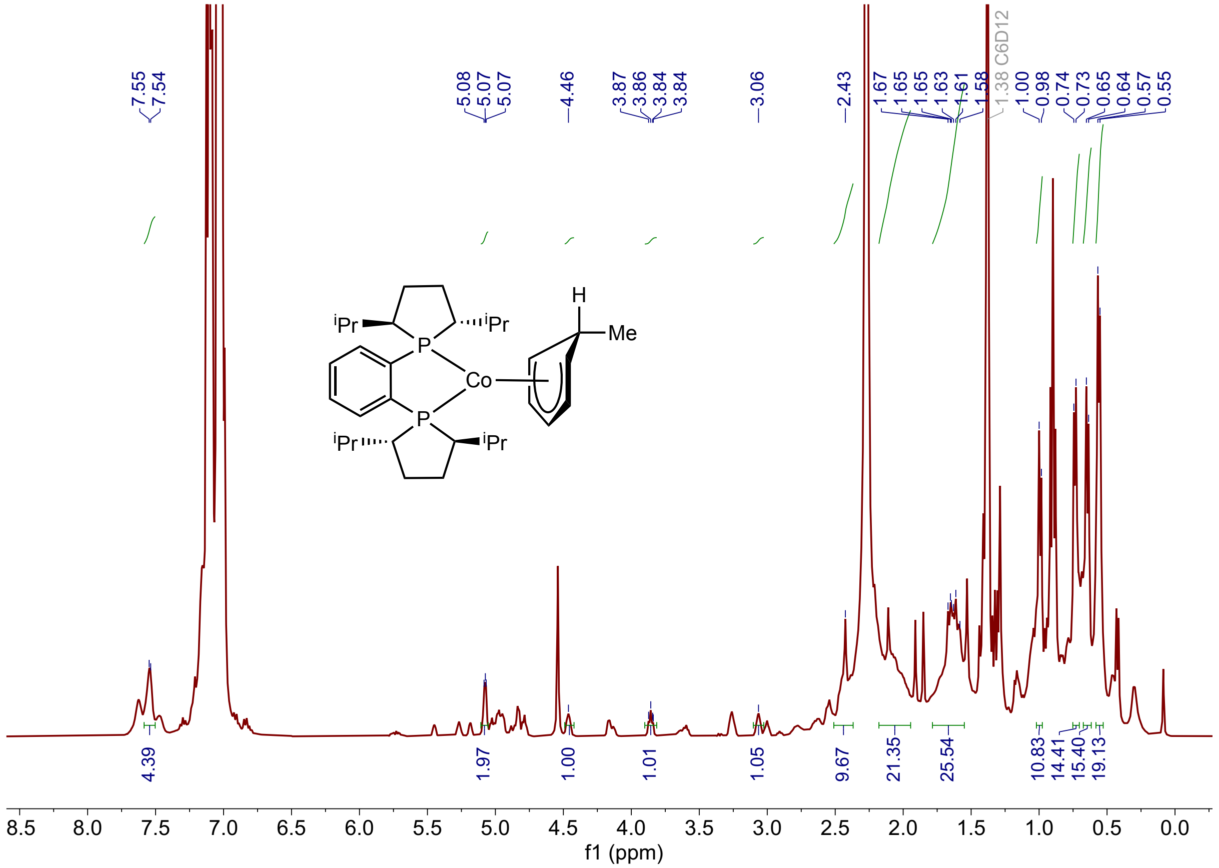

Figure S78. ^{1}H NMR spectrum (400 MHz, cyclohexane-d_{12}, 23 °C) of **Co1-c** in excess PhMe. *Ipso*-Me resonances assigned.

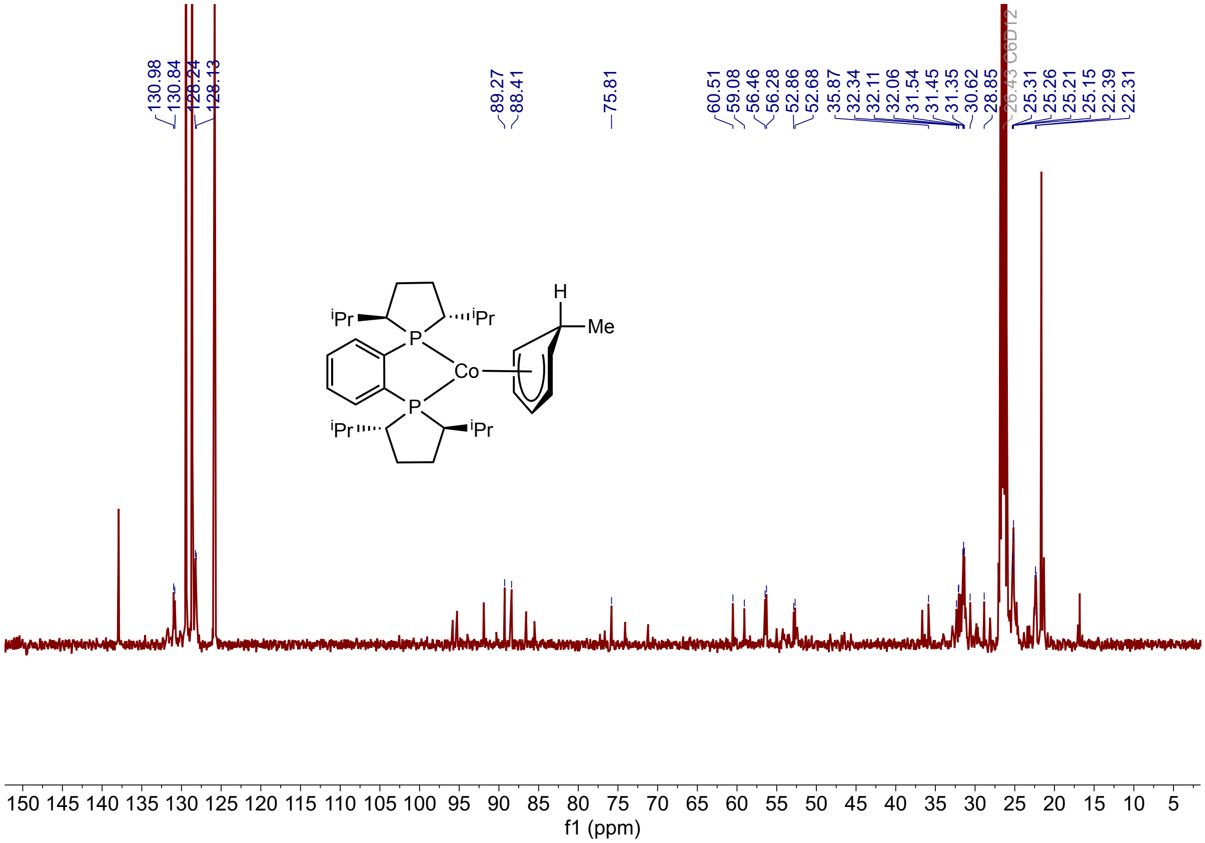

Figure S79. $^{13}C\{^{1}H\}$ NMR spectrum (101 MHz, cyclohexane-d_{12}, 23 °C) of **Co1-c** in excess PhMe. *Ipso*-Me resonances assigned.

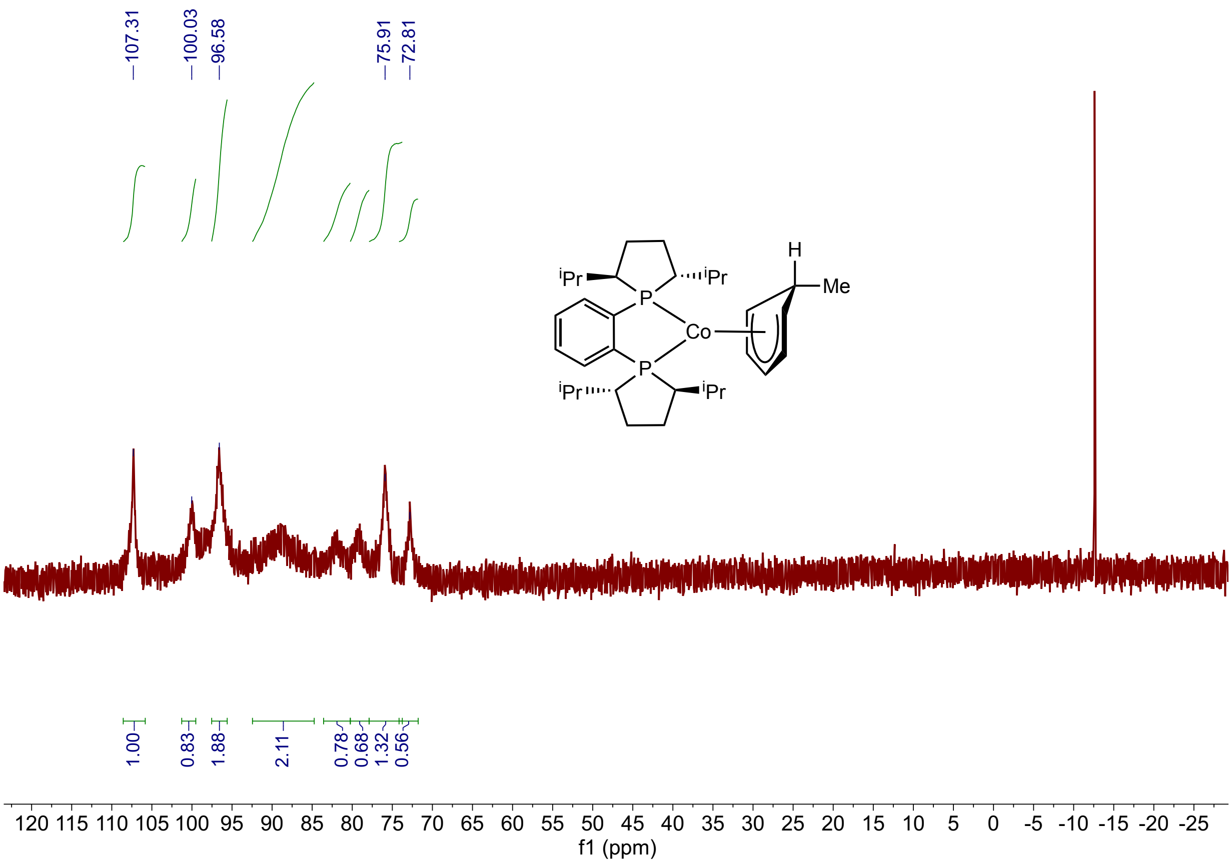

Figure S80. $^{31}P\{^{1}H\}$ NMR spectrum (162 MHz, cyclohexane-d_{12}, 23 °C) of **Co1-c** in excess PhMe. Free ligand observed at -12.61 ppm.

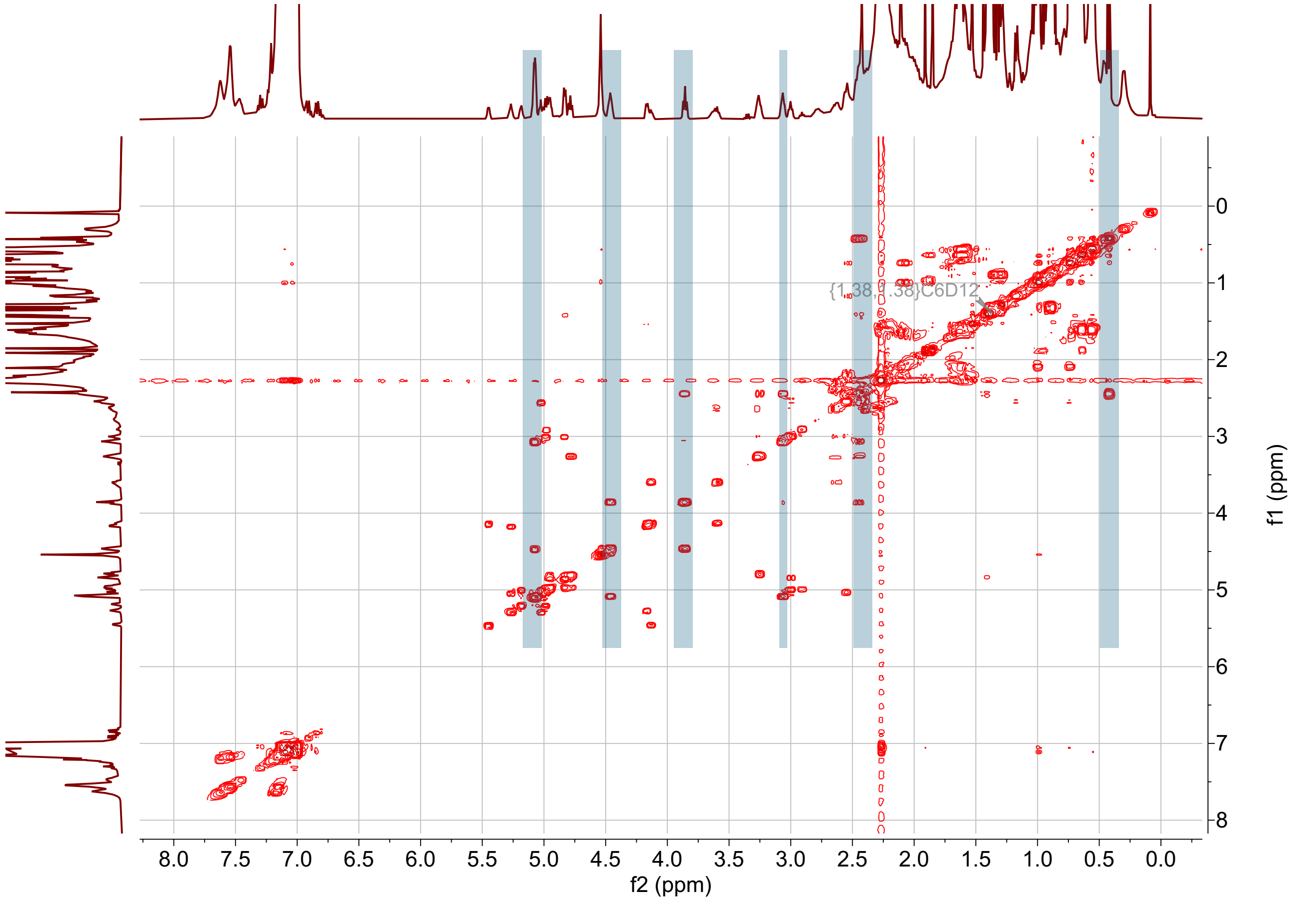

Figure S81. ^1H–^1H COSY NMR spectrum (cyclohexane-d_{12}, 23 °C) of **Co1-c** in excess PhMe. *Ipso*-Me resonances assigned.

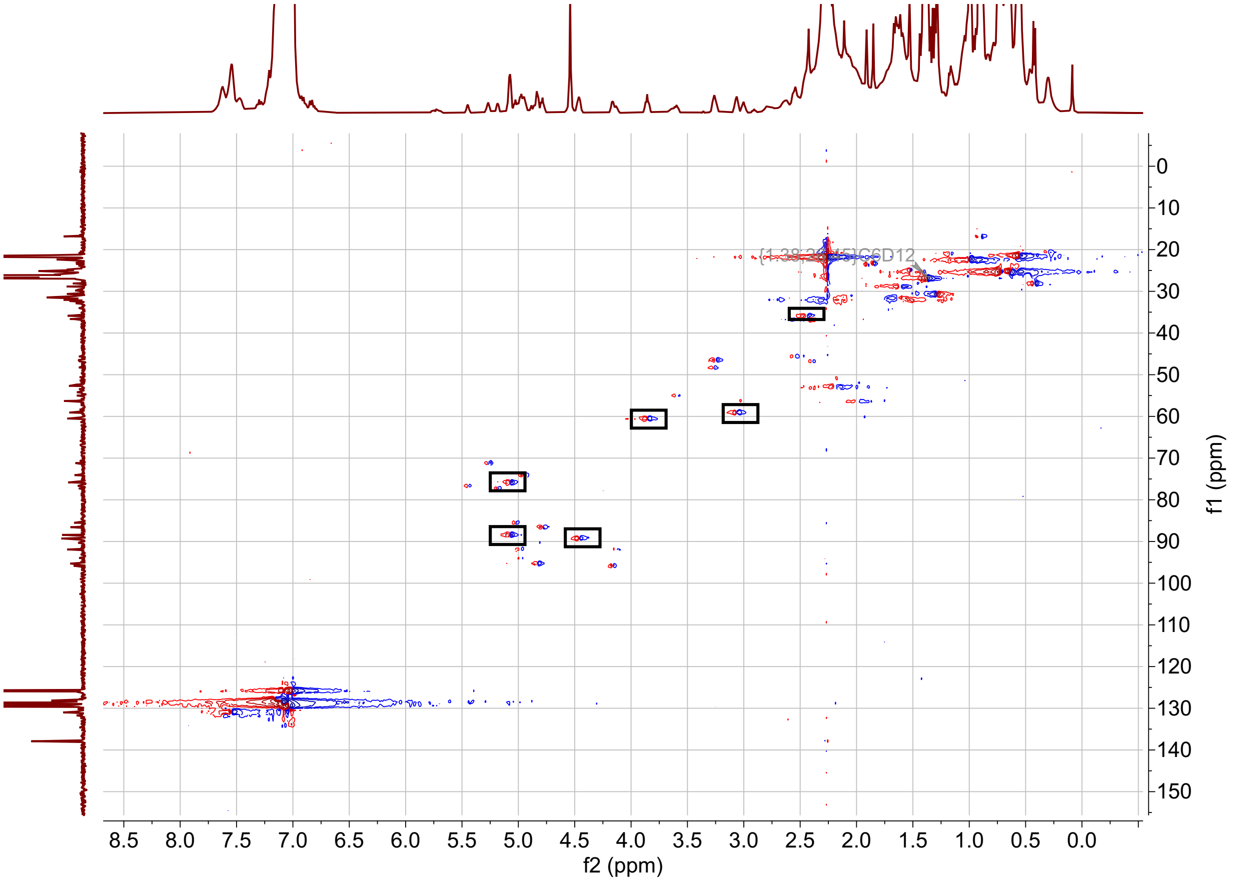

Figure S82. ^{1}H–$^{13}C\{^{1}H\}$ HSQC NMR spectrum (cyclohexane-d_{12}, 23 °C) of **Co1-c** in excess PhMe. Inset: assignment of *ipso*-Me resonances.

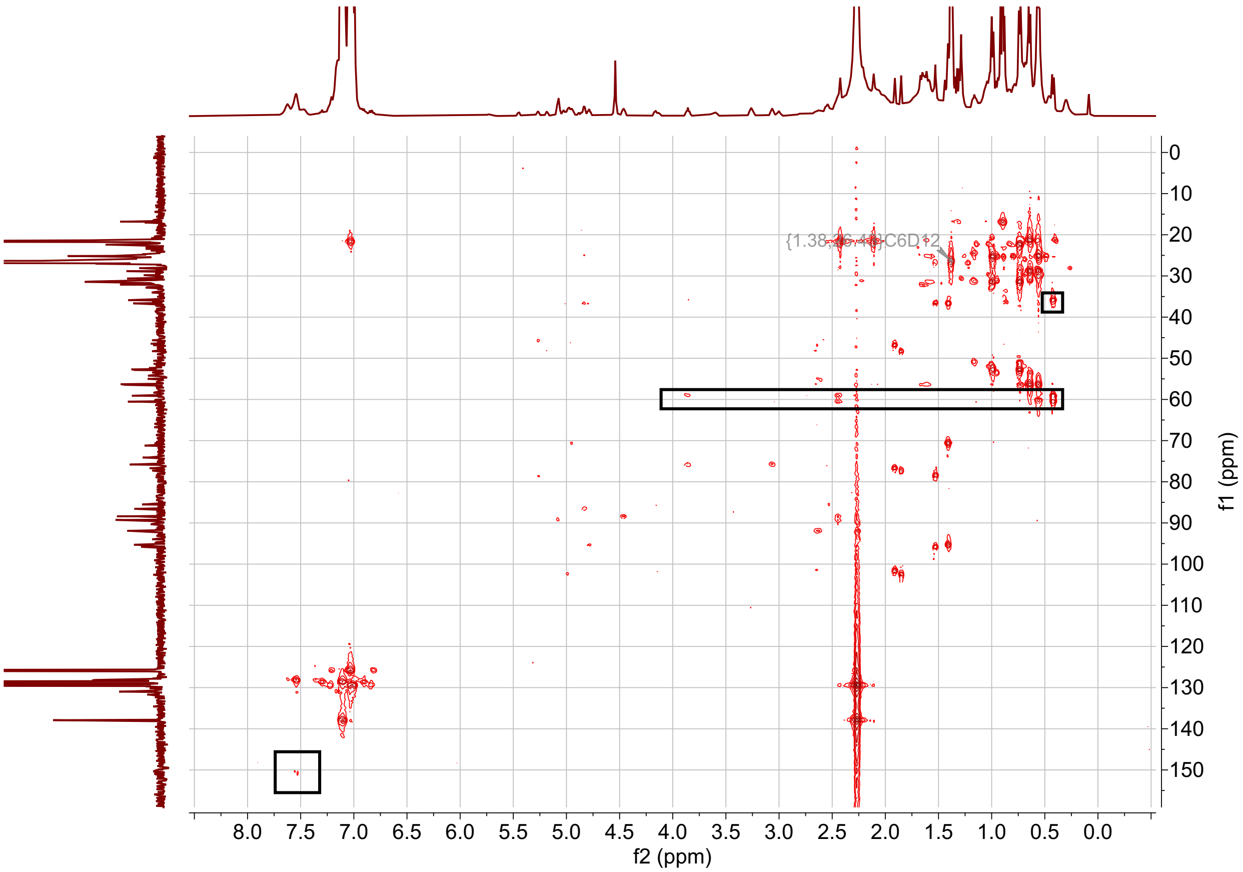

Figure S83. ${}^{1}H$–${}^{13}C\{{}^{1}H\}$ HMBC NMR spectrum (cyclohexane-d_{12}, 23 °C) of **Co1-c** in excess PhMe. Inset: assignment of quaternary carbons and *ipso*-Me–H correlation.

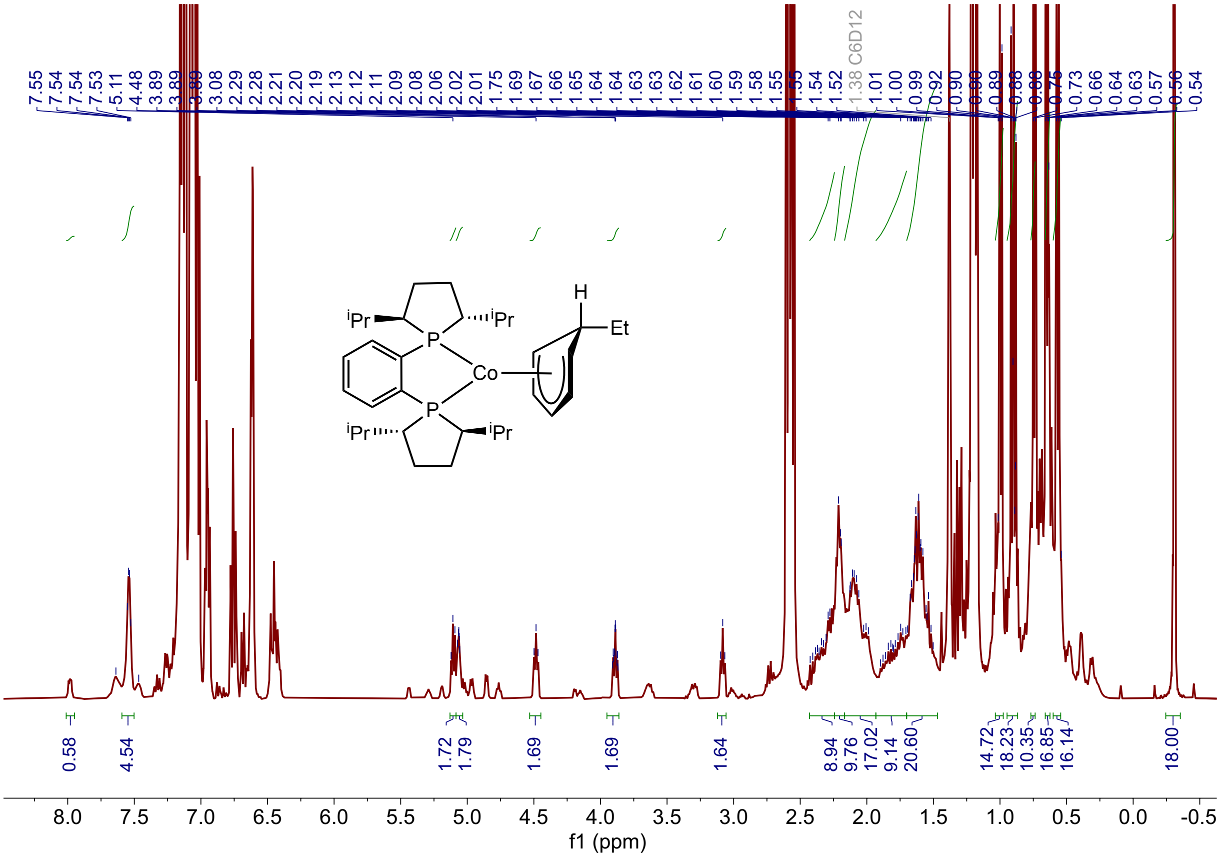

Figure S84. ^{1}H NMR spectrum (400 MHz, cyclohexane-d_{12}, 23 °C) of **Co1-d** in excess PhEt. *Ipso*-Et resonances assigned with 2-fluoro-biphenyl (δ 6.75, 6.67, 6.49 ppm), PPh_3 (δ 6.94, 6.59 ppm), and HMDSO (δ 0.10 ppm) capillary in cyclohexane-d_{12}.

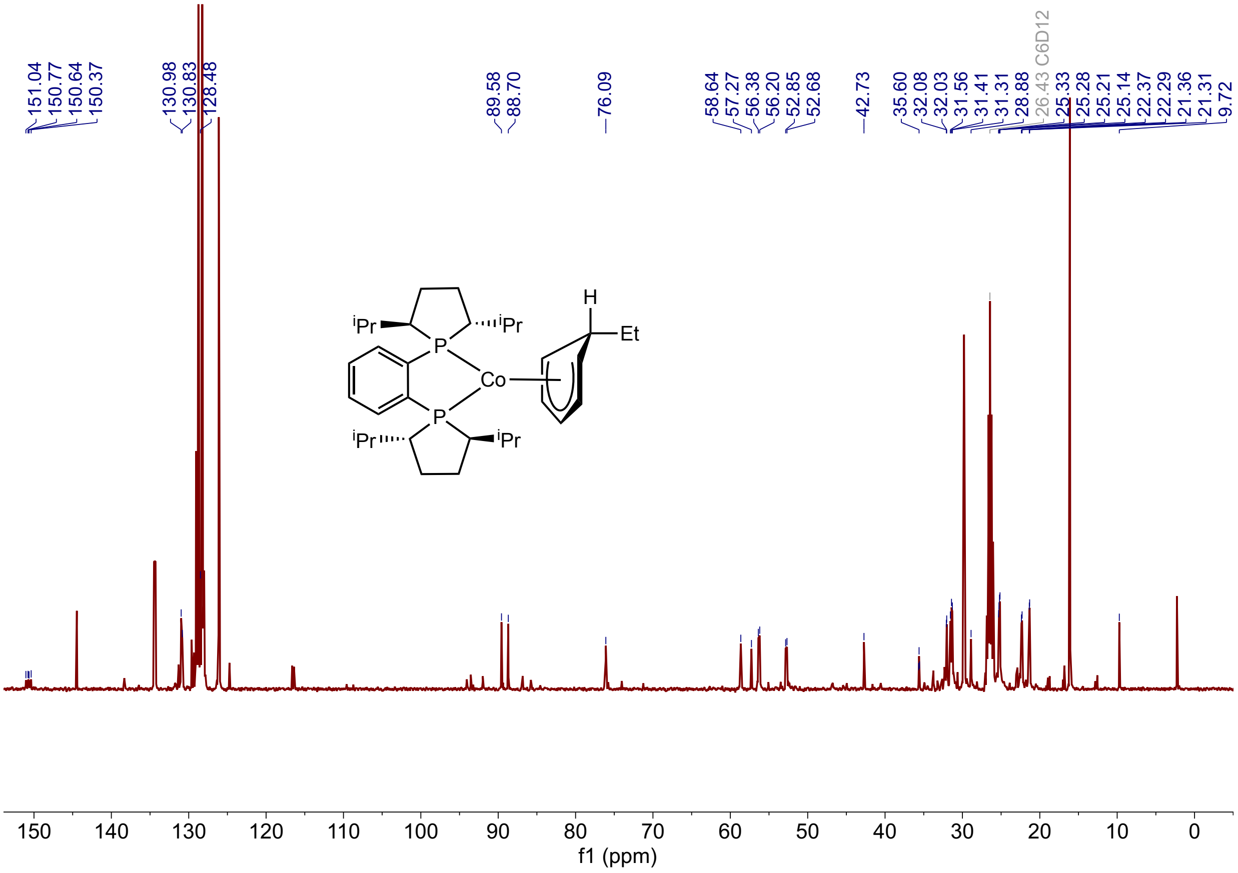

Figure S85. $^{13}C\{^{1}H\}$ NMR spectrum (101 MHz, cyclohexane-d_{12}, 23 °C) of **Co1-d** in excess PhEt. *Ipso*-Et resonances assigned. Additional resonances correspond to PhEt, 2-fluoro-biphenyl, PPh_3, and HMDSO.

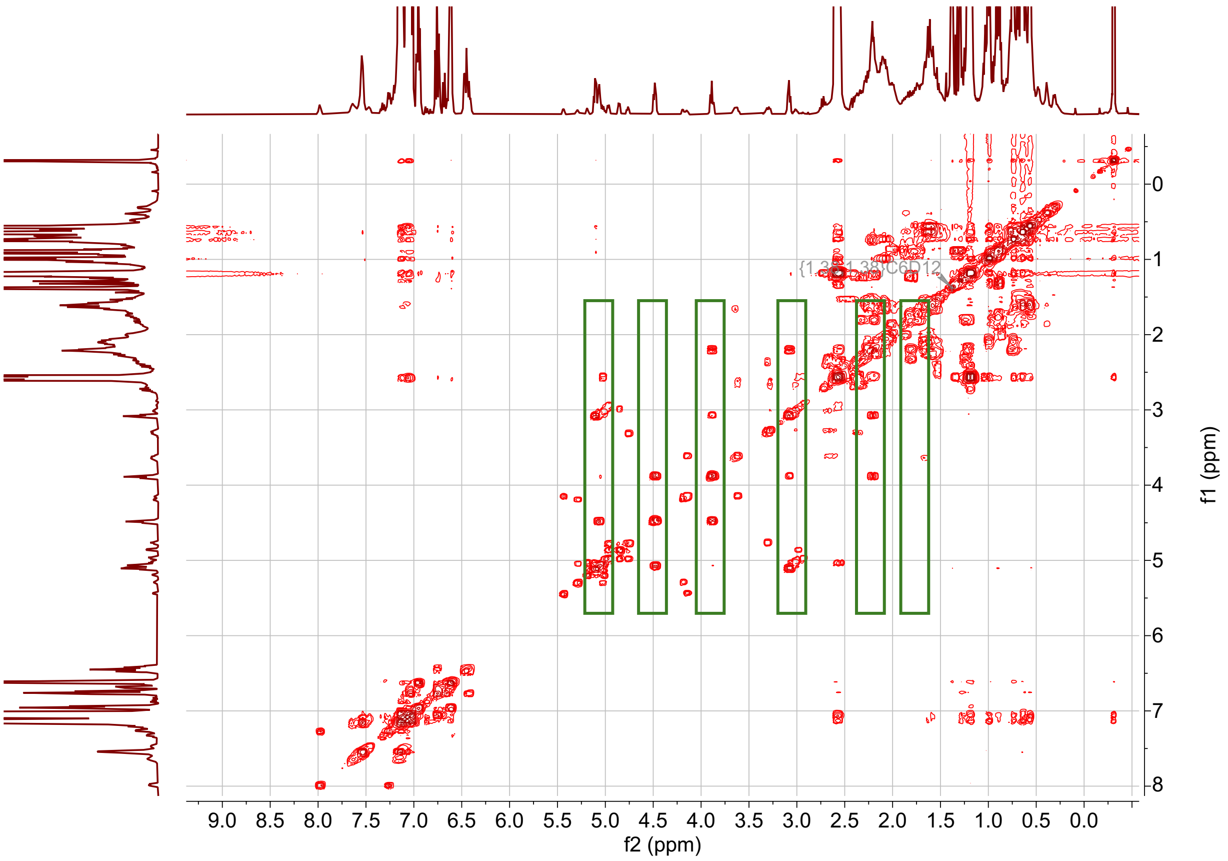

Figure S86. ^{1}H–^{1}H COSY NMR spectrum (cyclohexane-d_{12}, 23 °C) of **Co1-d** in excess PhEt. *Ipso*-Et resonances assigned. Additional resonances correspond to PhEt, 2-fluoro-biphenyl, PPh_3, and HMDSO. Inset: ^{1}H–^{1}H correlation between η^5-cyclohexadienyl signals.

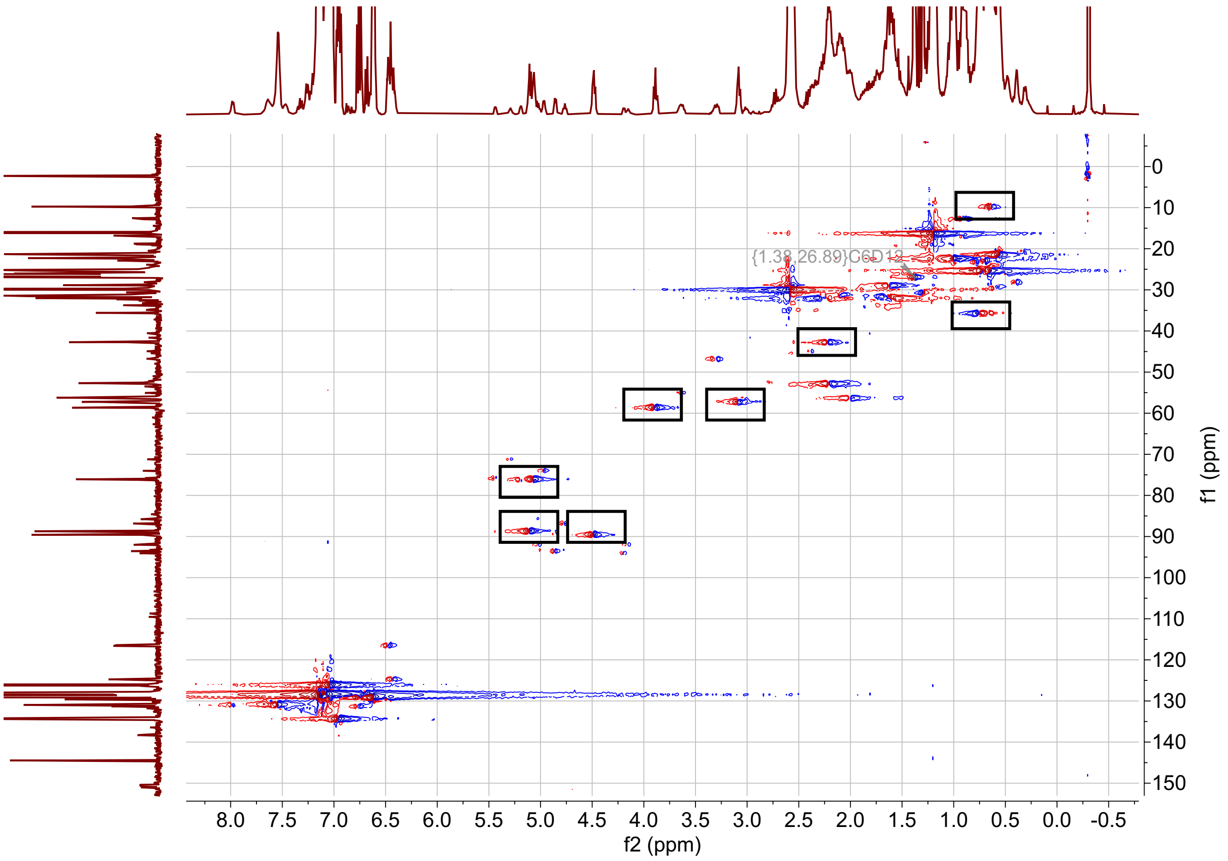

Figure S87. ${}^{1}H$–${}^{13}C\{{}^{1}H\}$ HSQC NMR spectrum (cyclohexane-d_{12}, 23 °C) of **Co1-d** in excess PhEt. Additional resonances assigned with 2-fluoro-biphenyl, PPh_3, and HMDSO. Inset: assignment of *ipso*-Et η^5-cyclohexadienyl resonances.

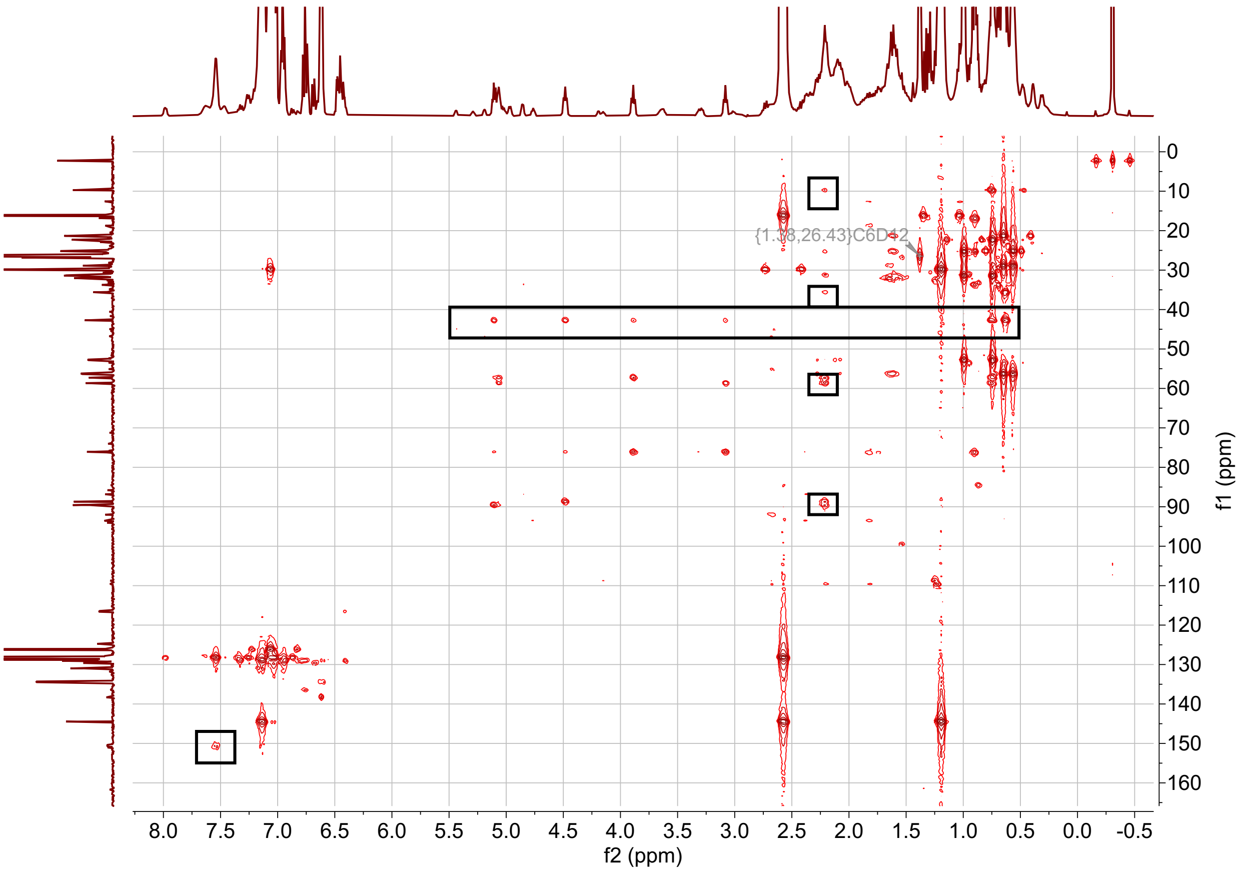

Figure S88. ${}^{1}H$–${}^{13}C\{{}^{1}H\}$ HMBC NMR spectrum (cyclohexane-d_{12}, 23 °C) of **Co1-d** in excess PhEt. Additional resonances assigned with 2-fluoro-biphenyl, PPh_3, and HMDSO capillary in cyclohexane-d_{12}. Inset: Assignment of quaternary carbons and *ipso*-Et–H correlation.

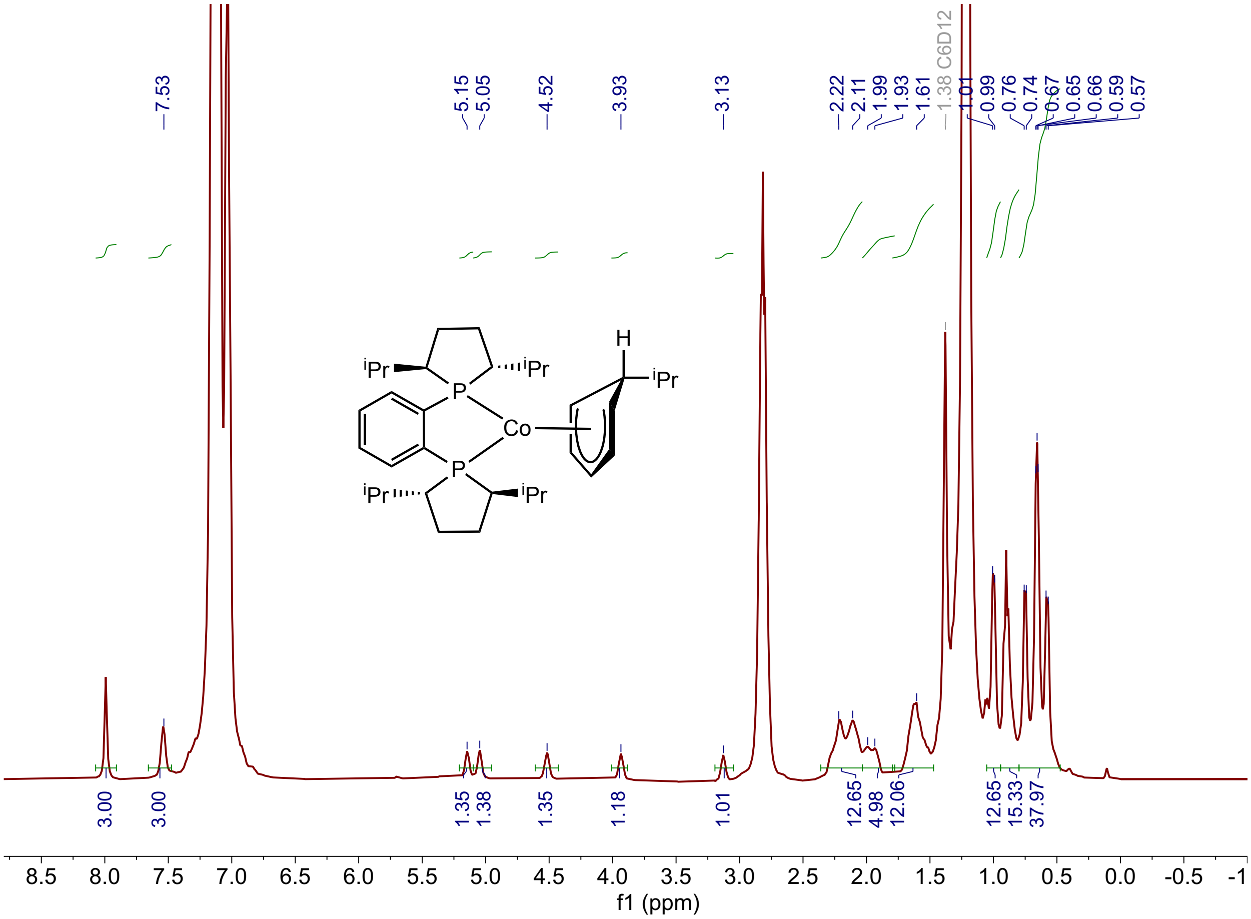

Figure S89. ^{1}H NMR spectrum (400 MHz, cyclohexane-d_{12}, 23 °C) of **Co1-e** with 1,3,5-tris(trifluoromethyl)benzene internal standard (δ 8.03 ppm) and excess Ph^{i}Pr.

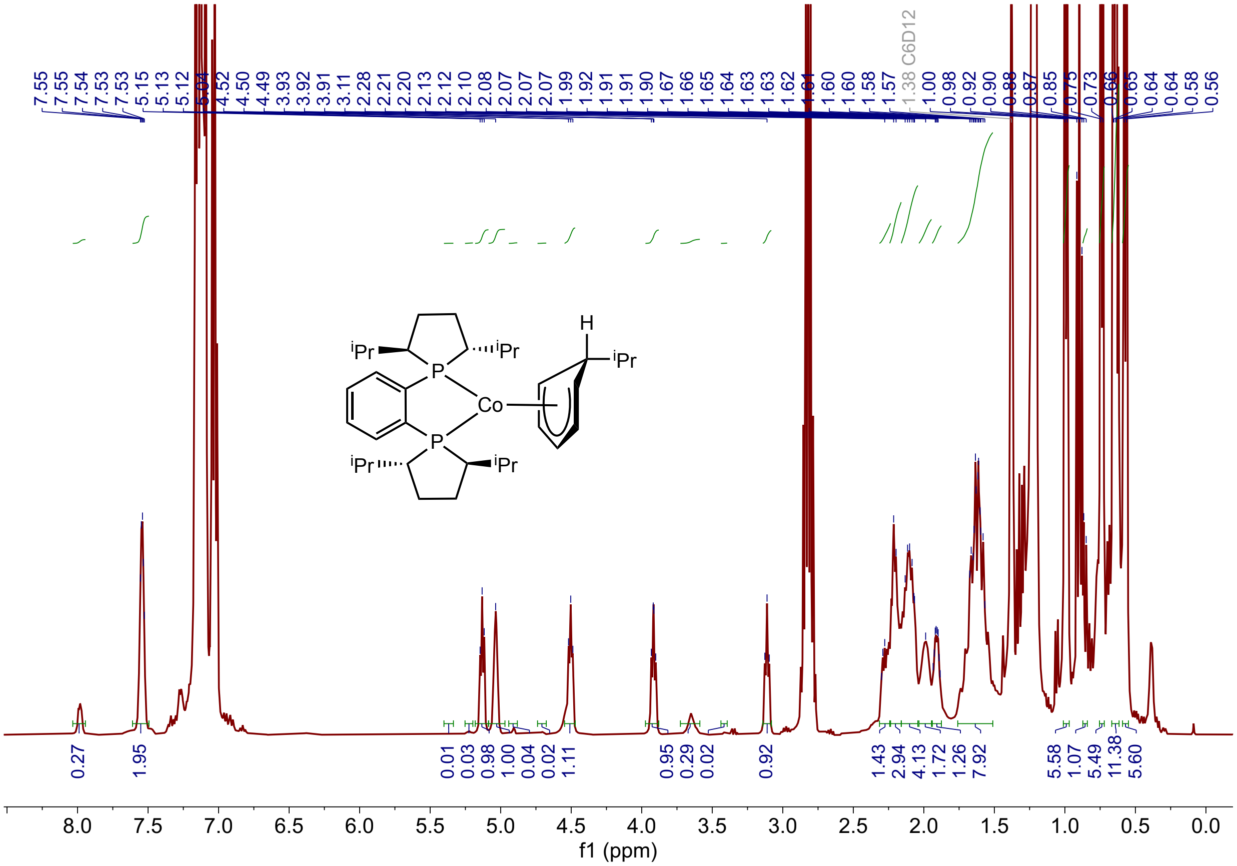

Figure S90. ^{1}H NMR spectrum (400 MHz, cyclohexane-d_{12}, 23 °C) of **Co1-e** in excess Ph^{i}Pr.

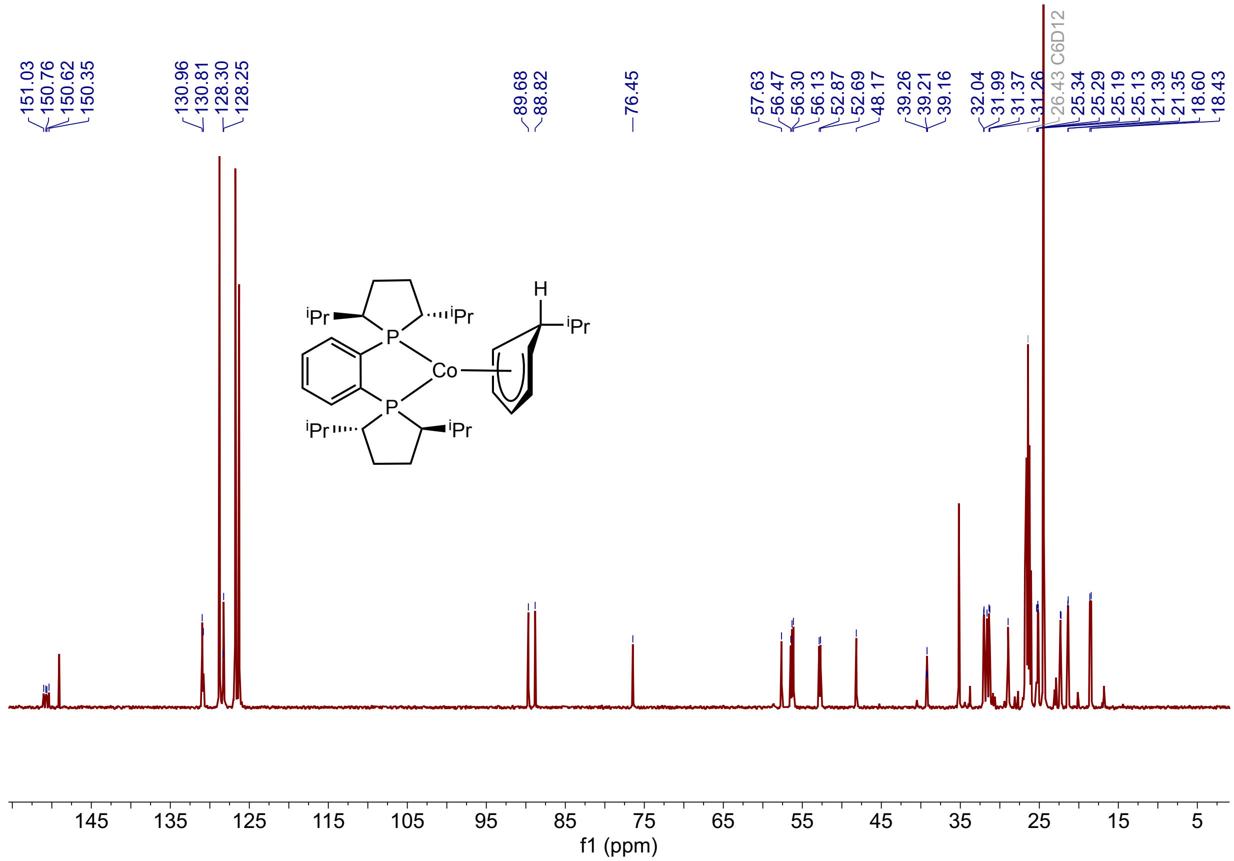

Figure S91. $^{13}C\{^{1}H\}$ NMR spectrum (101 MHz, cyclohexane-d_{12}, 23 °C) of **Co1-e** in excess Ph^{i}Pr.

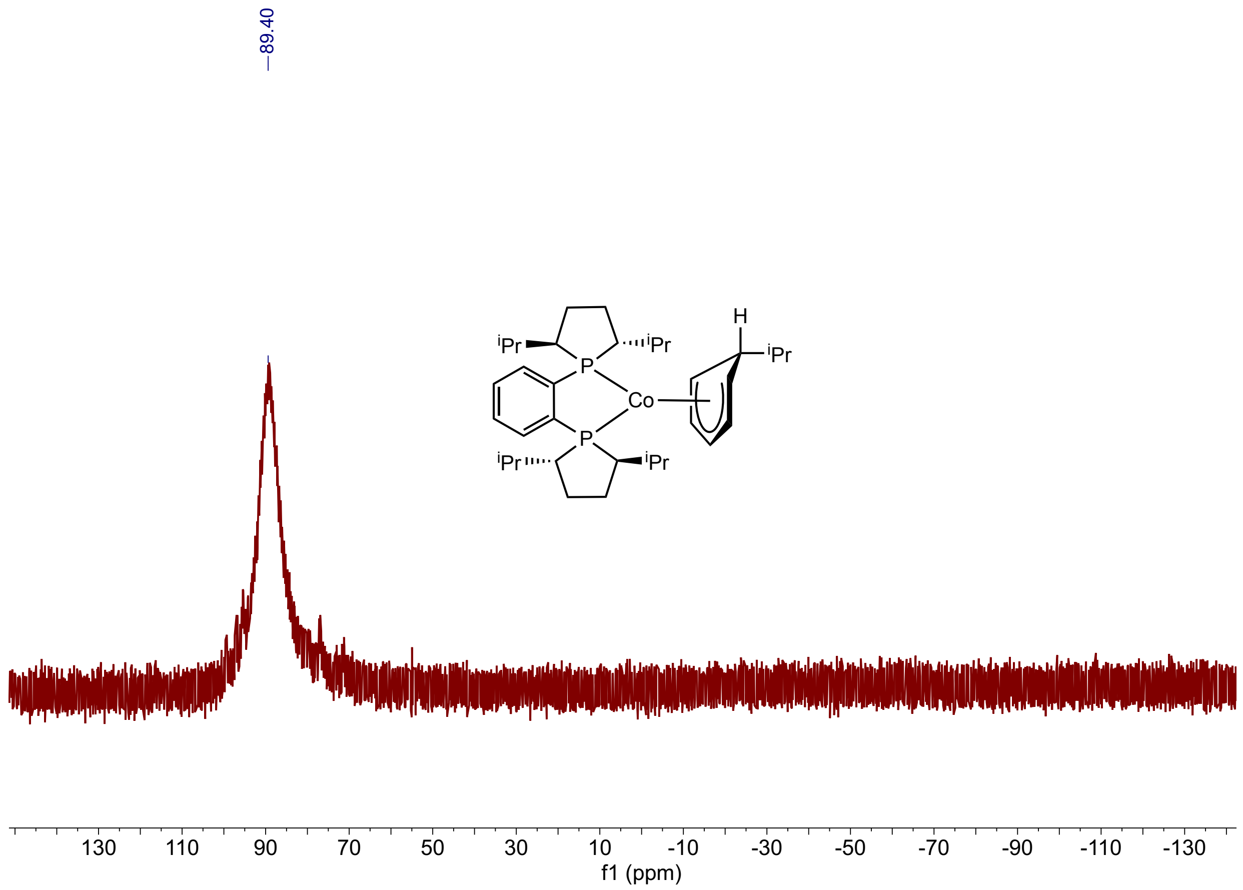

Figure S92. $^{31}P\{^{1}H\}$ NMR spectrum (162 MHz, cyclohexane-d_{12}, 23 °C) of **Co1-e**.

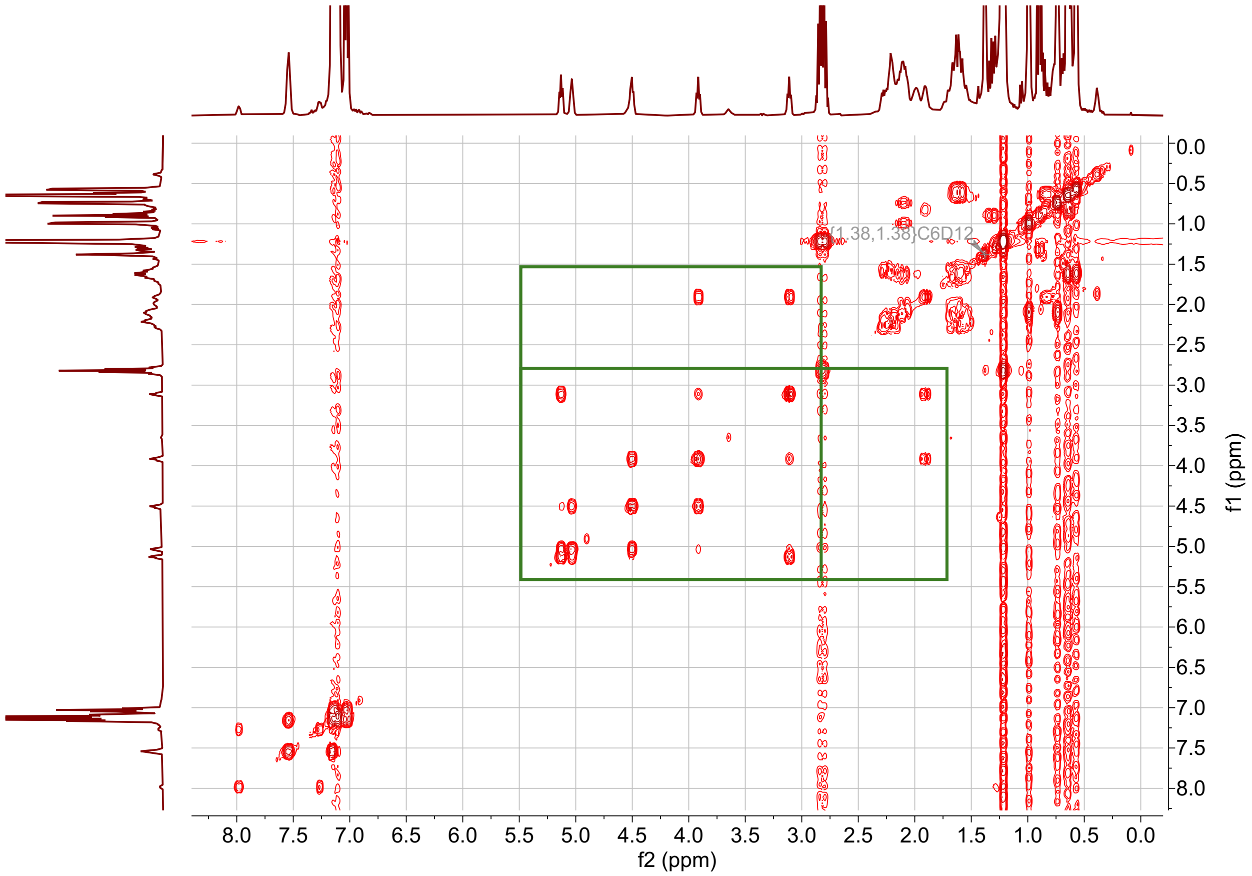

Figure S93. ^1H–^1H COSY NMR spectrum (cyclohexane-d_{12}, 23 °C) of **Co1-e** in excess Ph^{i}Pr. Inset: ^1H–^1H correlation between η^5-cyclohexadienyl signals.

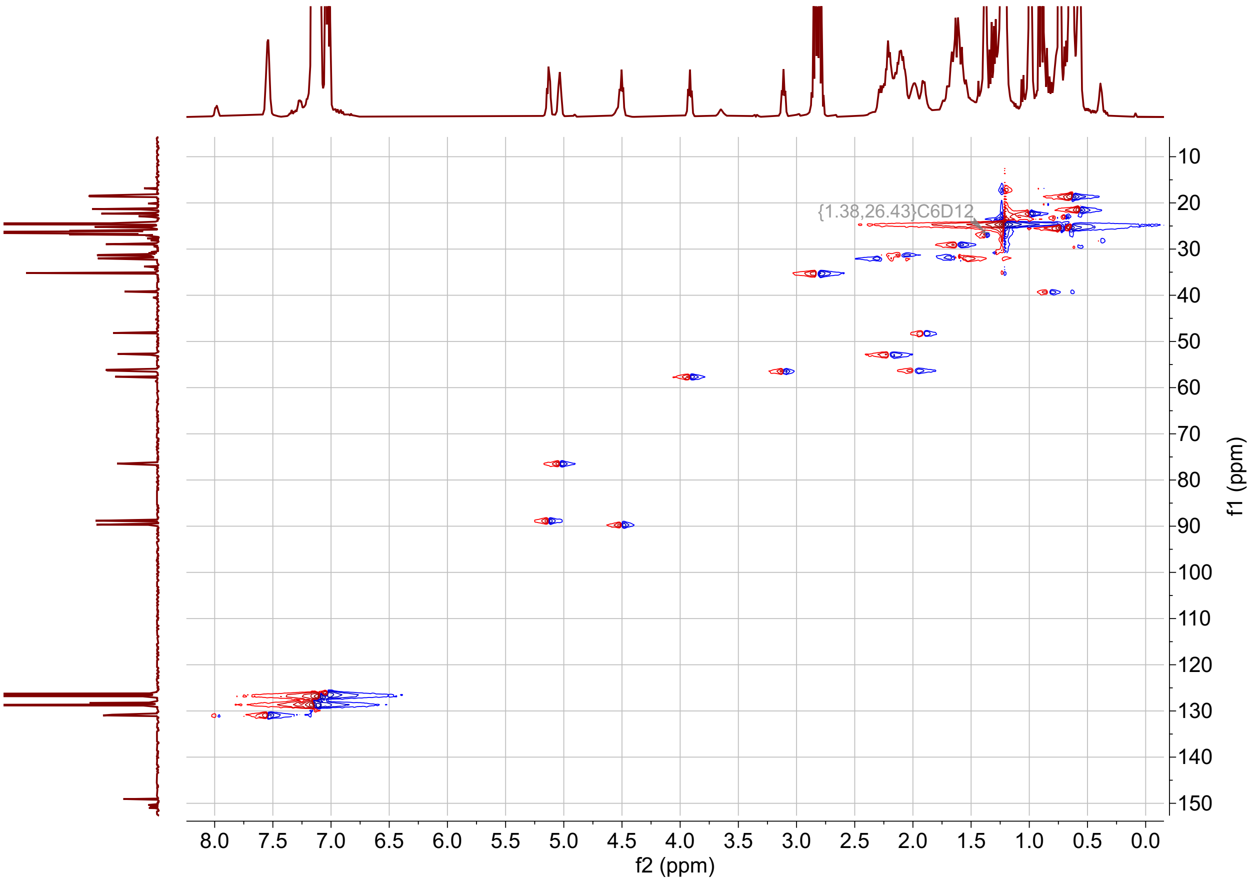

Figure S94. ^{1}H–$^{13}C\{^{1}H\}$ HSQC NMR spectrum (cyclohexane-d_{12}, 23 °C) of **Co1-e** in excess $Ph^{i}Pr$.

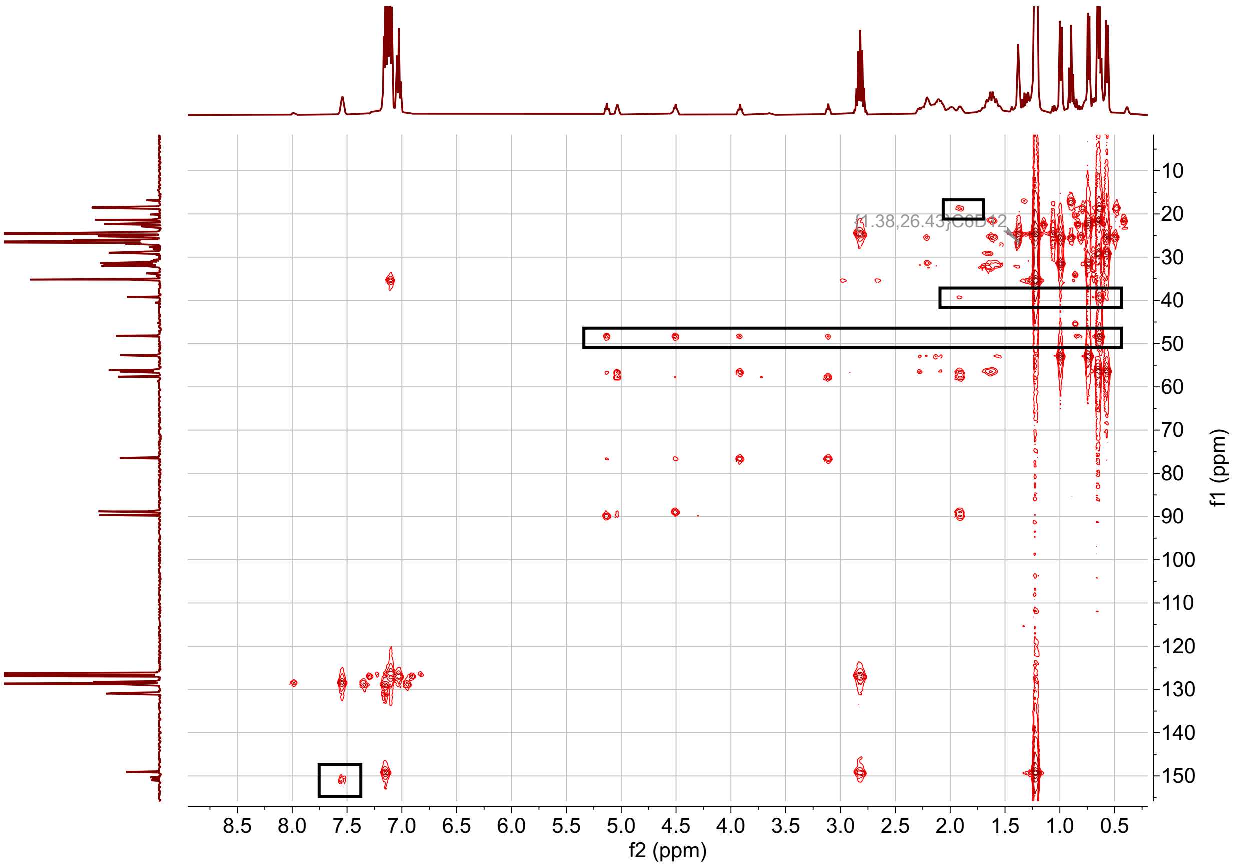

Figure S95. ^{1}H–$^{13}C\{^{1}H\}$ HMBC NMR spectrum (cyclohexane-d_{12}, 23 °C) of **Co1-e** in excess Ph^{i}Pr. Inset: Assignment of quaternary carbons and *ipso*-^{i}Pr–H correlation.

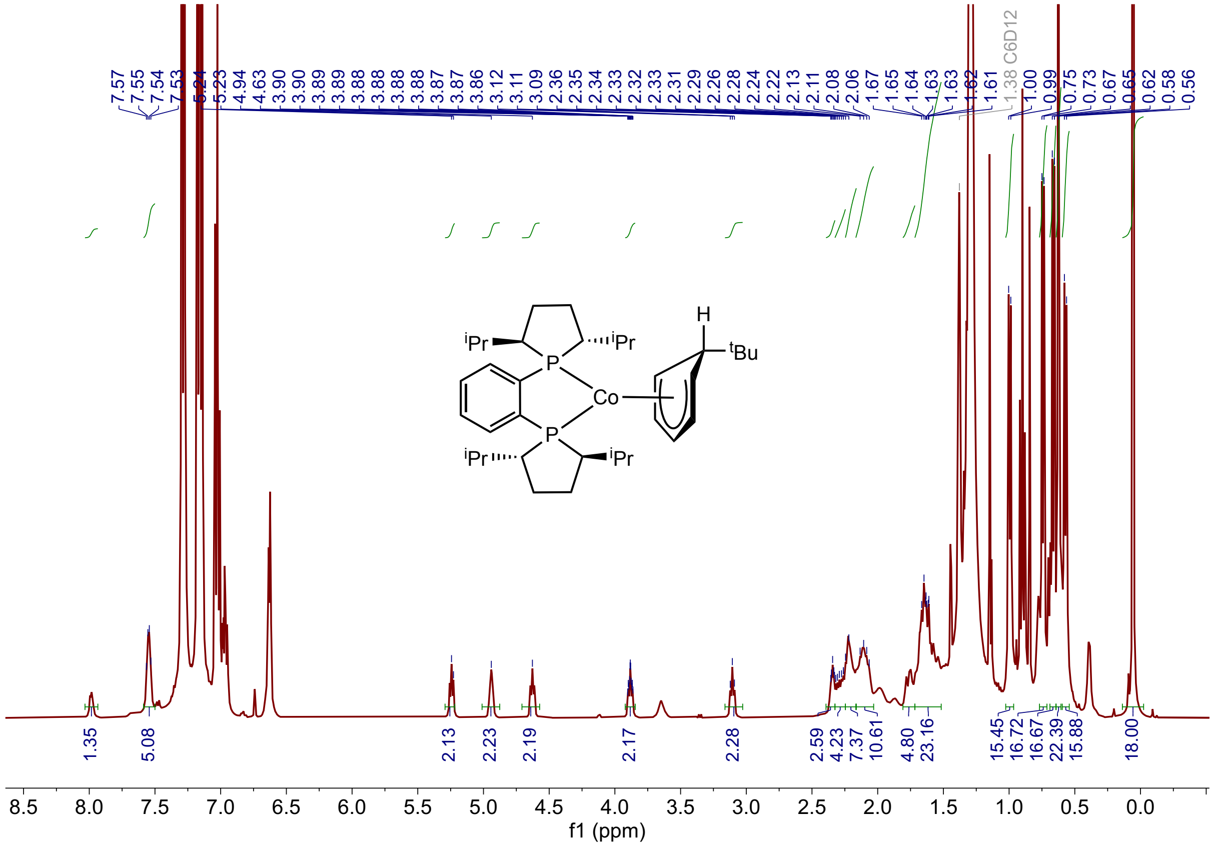

Figure S96. ^{1}H NMR spectrum (400 MHz, cyclohexane-d_{12}, 23 °C) of **Co1-f** in excess Ph^{t}Bu. Unassigned resonances correspond to PPh_3 (δ 6.94, 6.59 ppm) and HMDSO (δ 0.10 ppm) capillary in cyclohexane-d_{12} used as external standard.

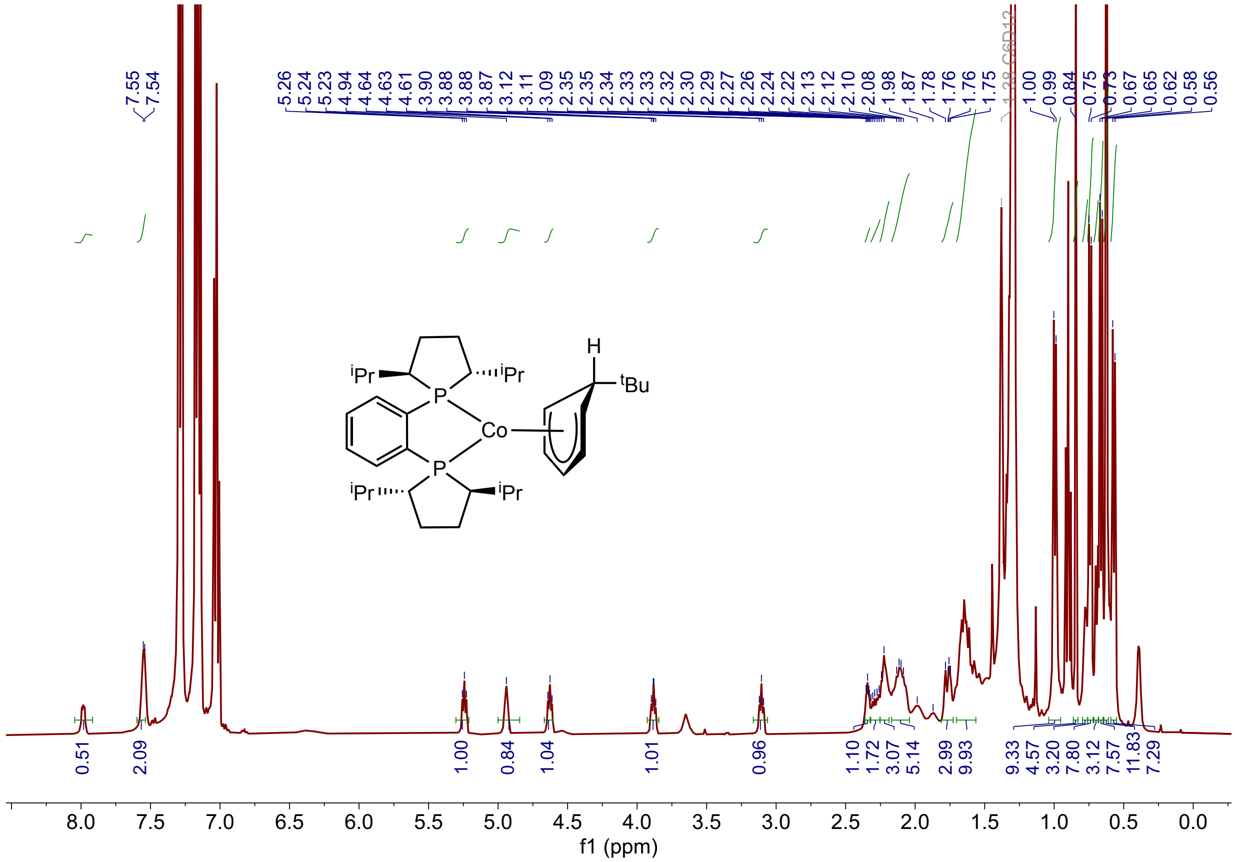

Figure S97. ^{1}H NMR spectrum (400 MHz, cyclohexane-d_{12}, 23 °C) of **Co1-f** in excess Ph^{t}Bu.

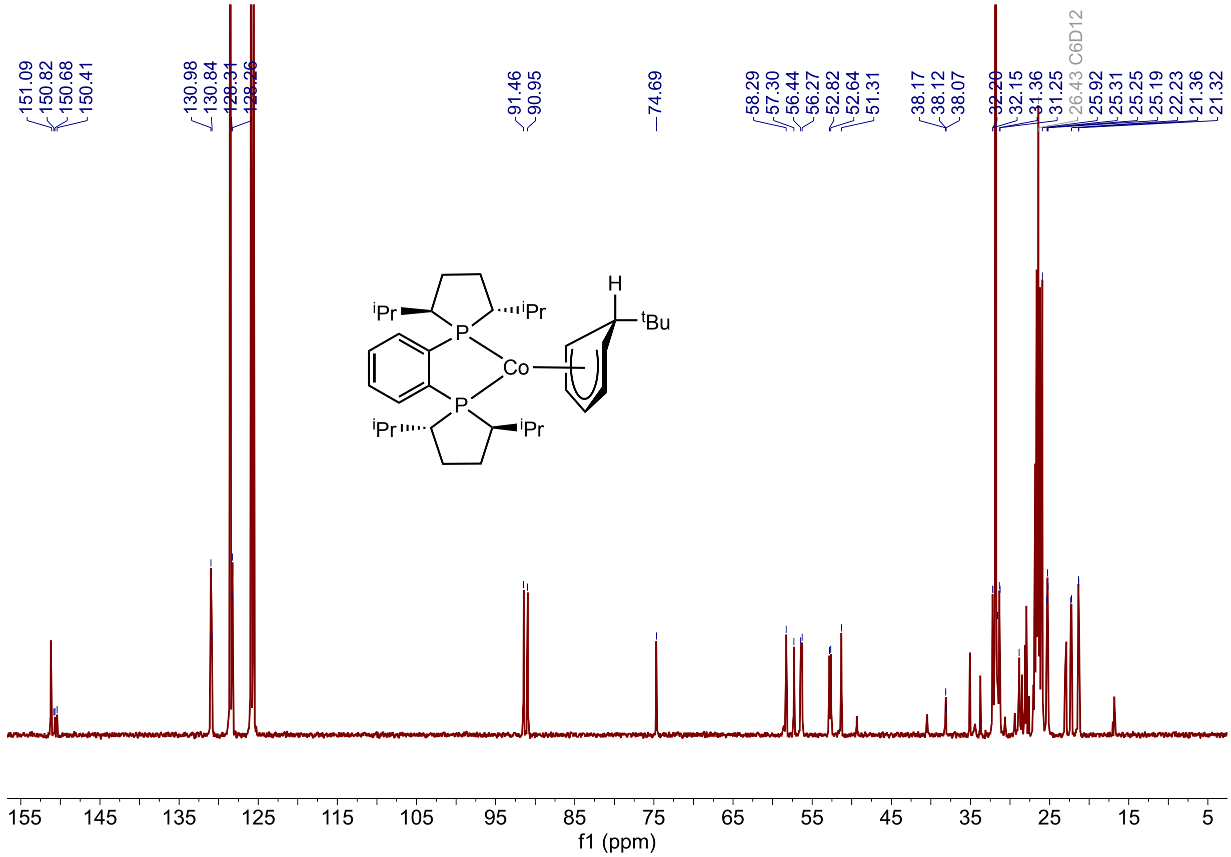

Figure S98. $^{13}C\{^1H\}$ NMR spectrum (101 MHz, cyclohexane-d_{12}, 23 °C) of **Co1-f** in excess Ph^{t}Bu.

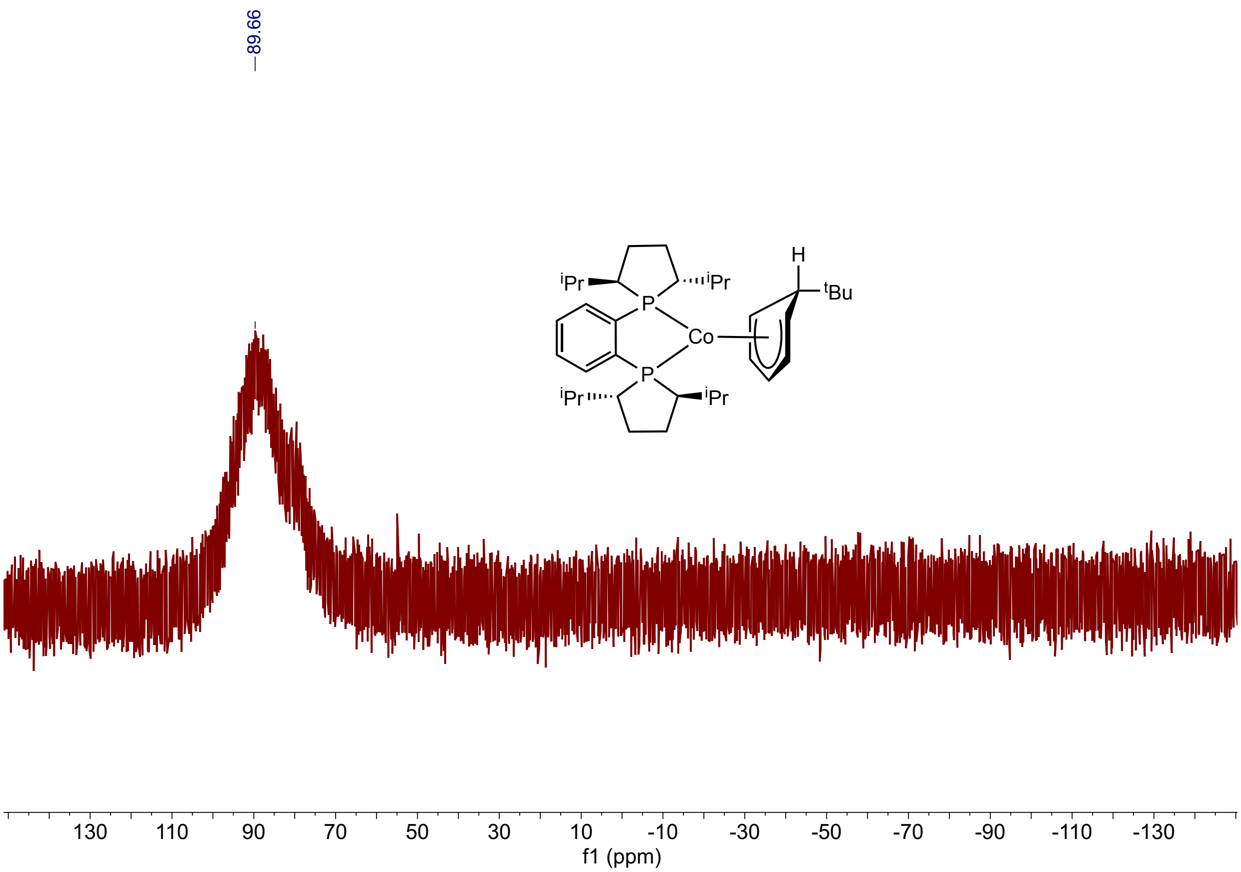

Figure S99. $^{31}P\{^{1}H\}$ NMR spectrum (162 MHz, cyclohexane-d_{12}, 23 °C) of **Co1-f** in excess $Ph^{t}Bu$.

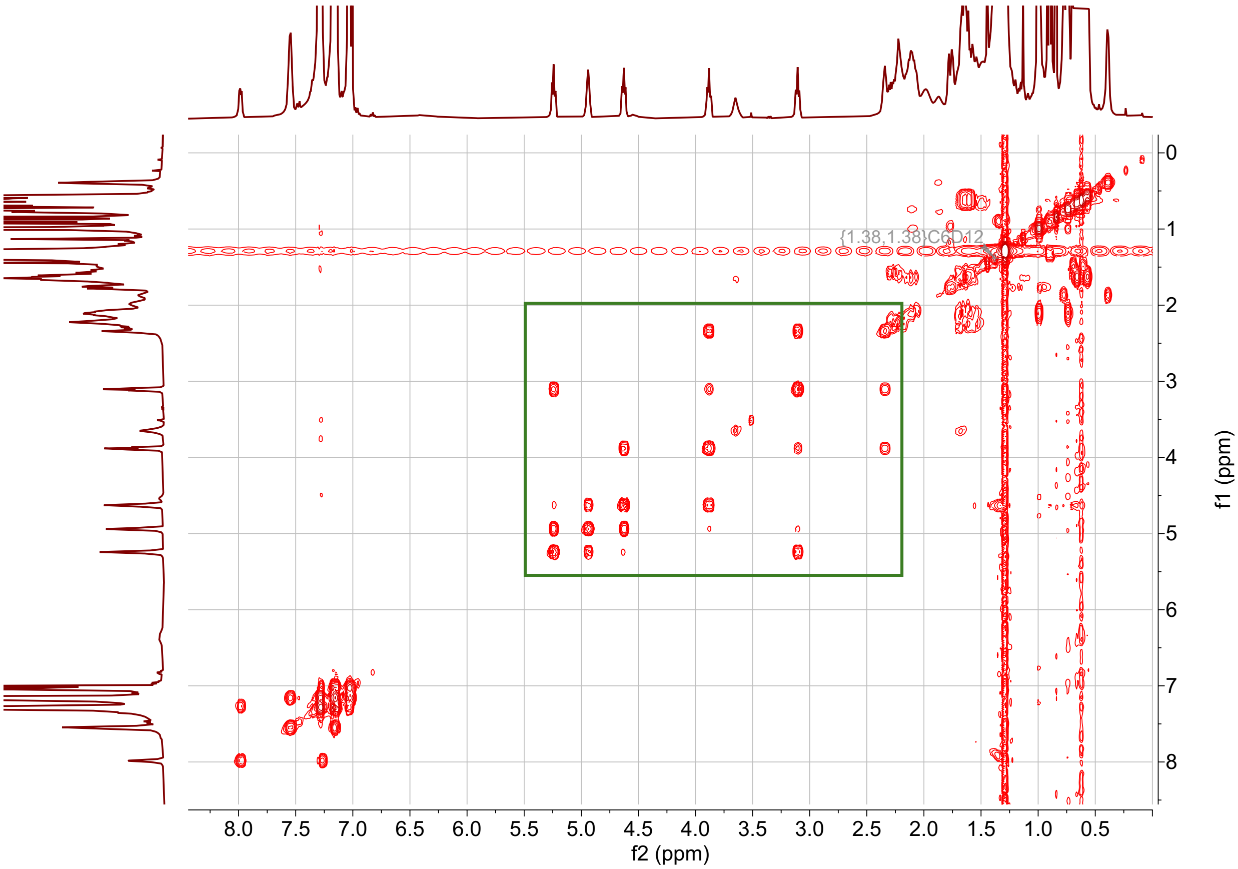

Figure S100. ^{1}H–^{1}H COSY NMR spectrum (cyclohexane-d_{12}, 23 °C) of **Co1-f** in excess Ph^{t}Bu.

Inset: ^{1}H–^{1}H correlation between η^5-cyclohexadienyl signals.

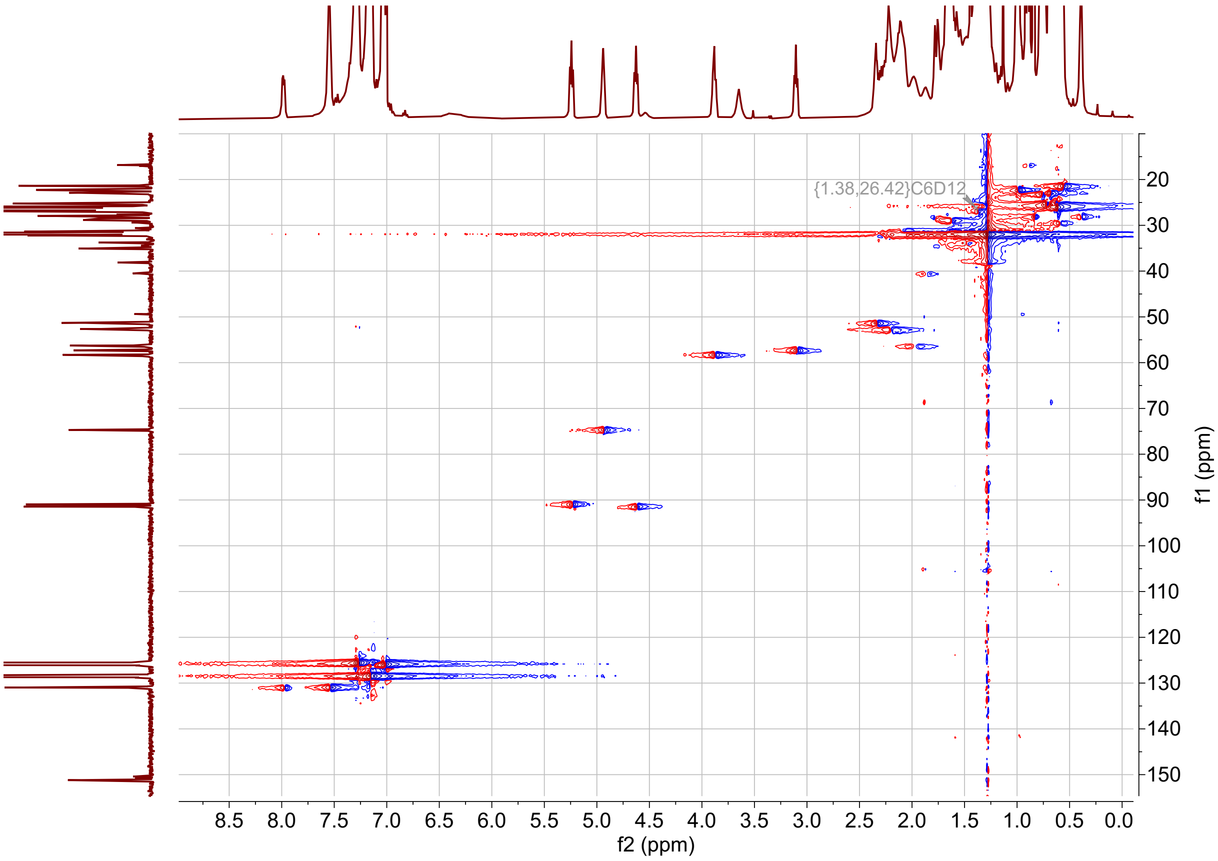

Figure S101. $^{1}H–^{13}C\{^{1}H\}$ HSQC NMR spectrum (cyclohexane-d_{12}, 23 °C) of **Co1-f** in excess $Ph^{t}Bu$.

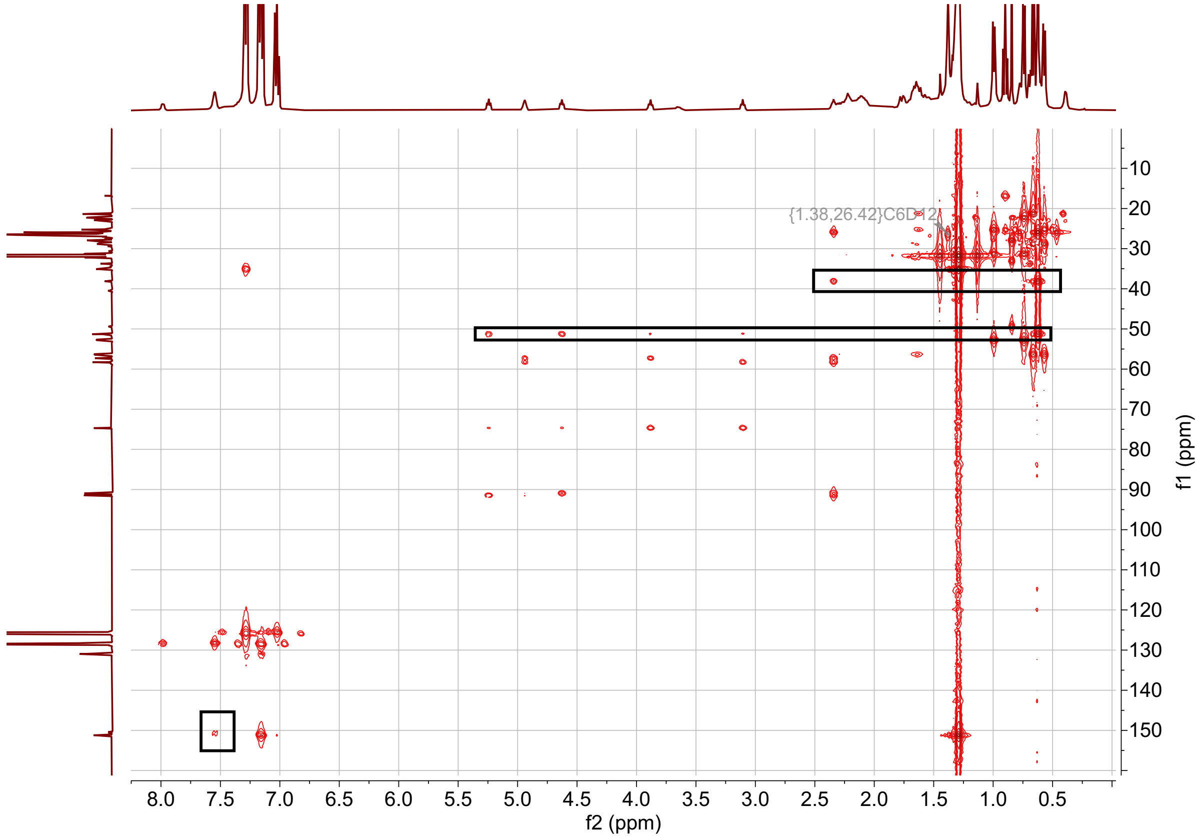

Figure S102. ^{1}H–$^{13}C\{^{1}H\}$ HMBC NMR spectrum (cyclohexane-d_{12}, 23 °C) of **Co1-f** in excess $Ph^{t}Bu$. Inset: Assignment of quaternary carbons and *ipso*-^{t}Bu–H correlation.

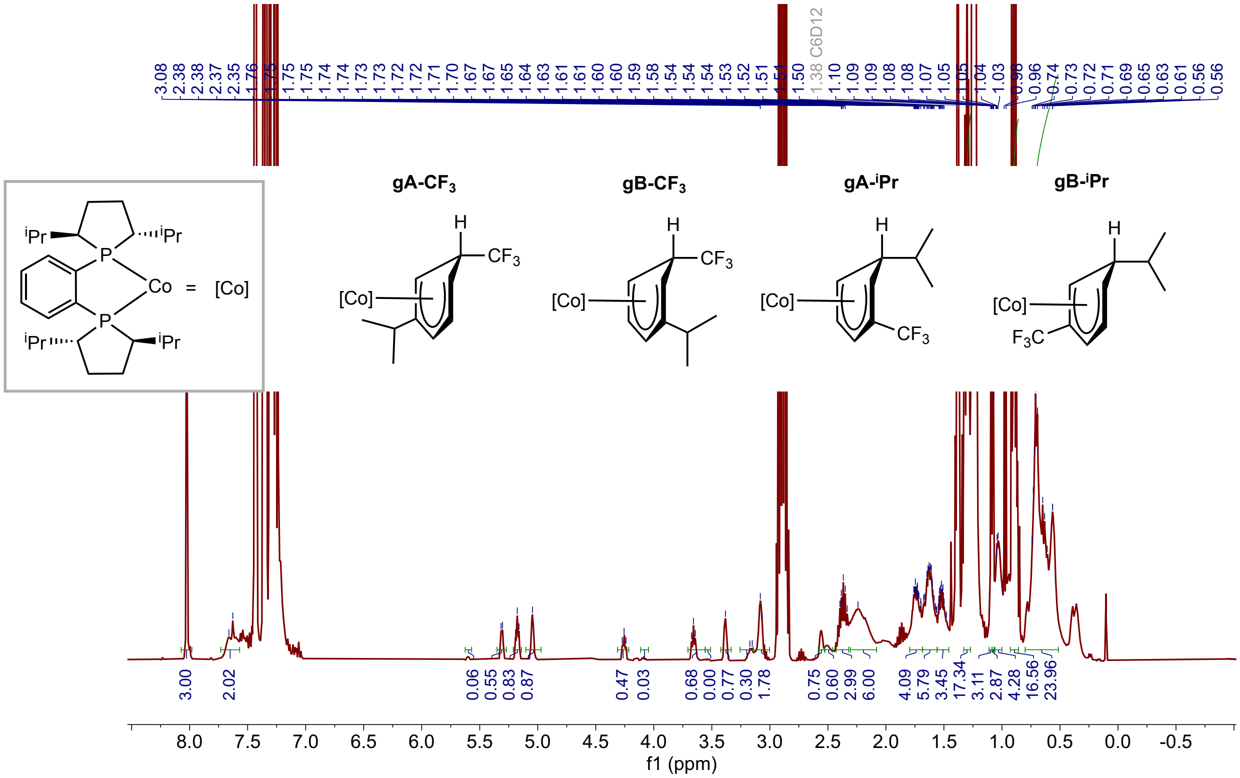

Figure S103. ^{1}H NMR spectrum (400 MHz, cyclohexane-d_{12}, 23 °C) of **Co1-g** in excess arene and 1,3,5-tris(trifluoromethyl)benzene internal standard (δ 8.03 ppm).

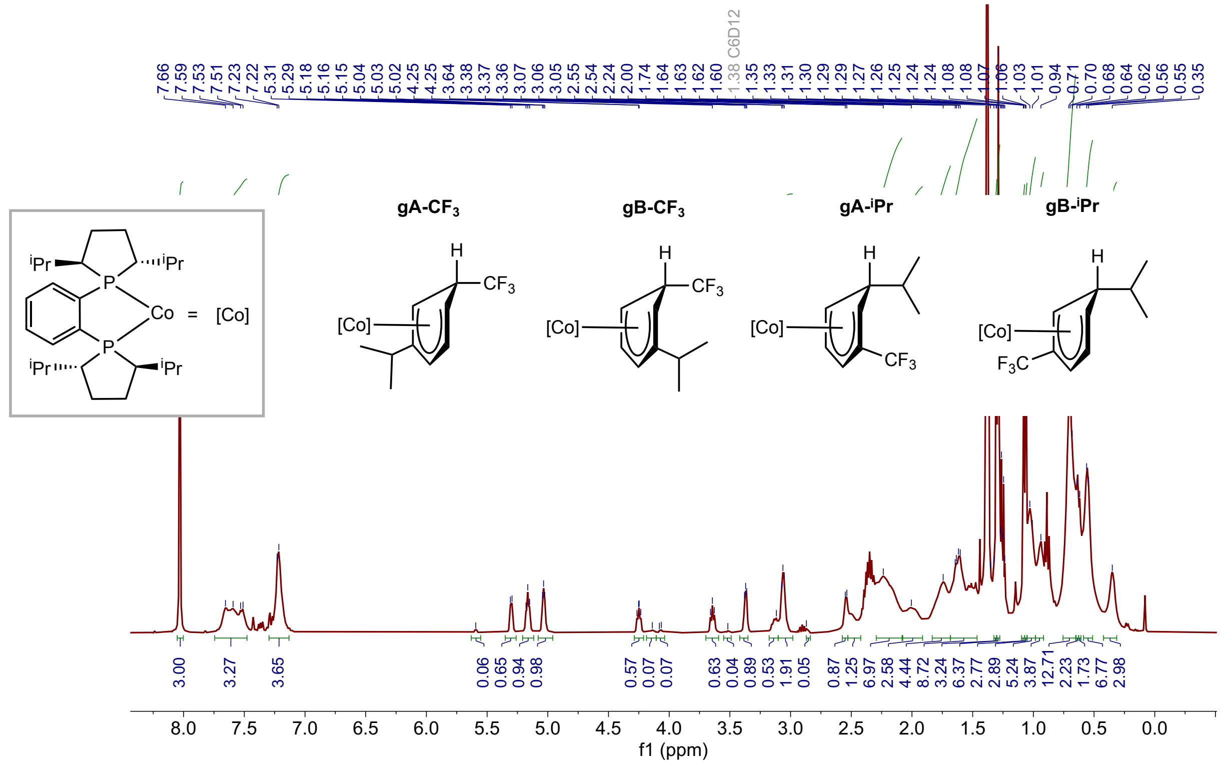

Figure S104. ^{1}H NMR spectrum (400 MHz, cyclohexane-d_{12}, 23 °C) of isolated **Co1-g** and 1,3,5-tris(trifluoromethyl)benzene internal standard (δ 8.03 ppm).

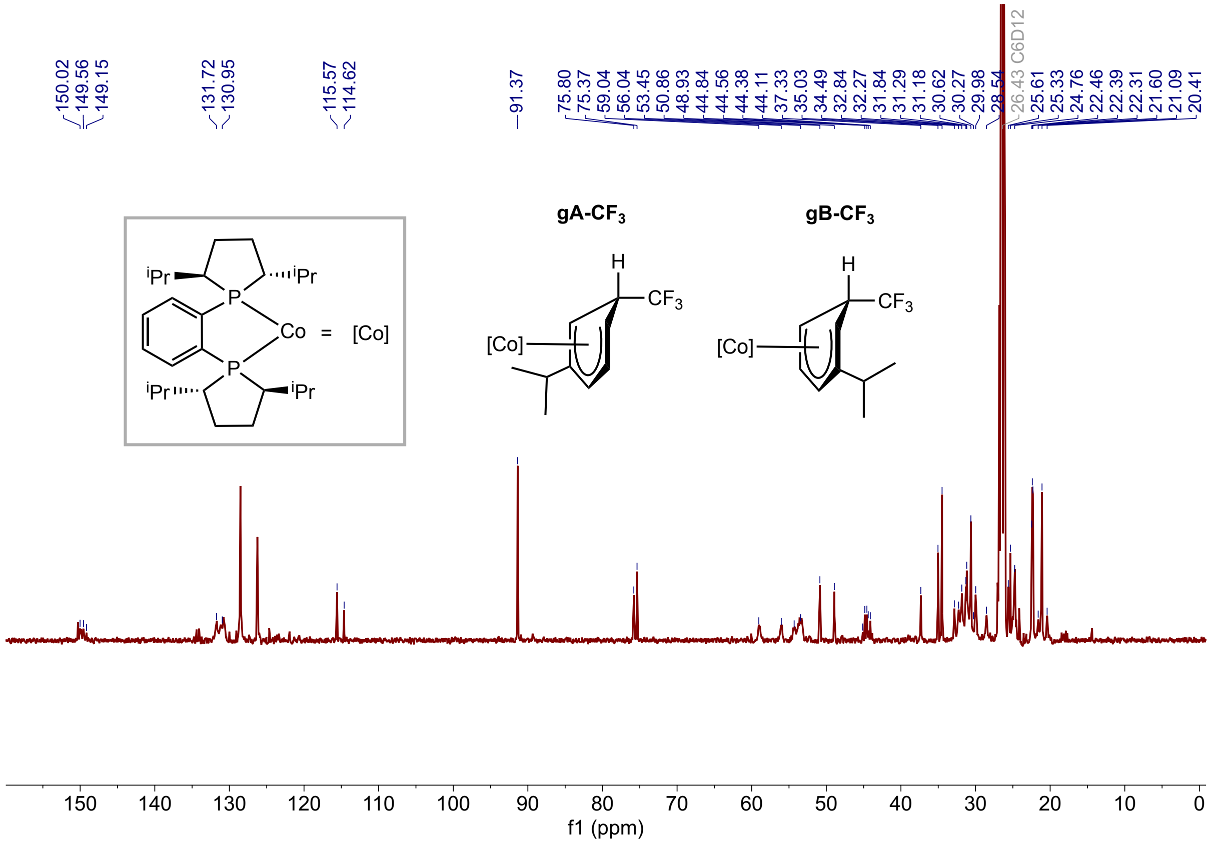

Figure S105. $^{13}C\{^{1}H\}$ NMR spectrum (101 MHz, cyclohexane-d_{12}, 23 °C) of isolated **Co1-g** with 1,3,5-tris(trifluoromethyl)benzene.

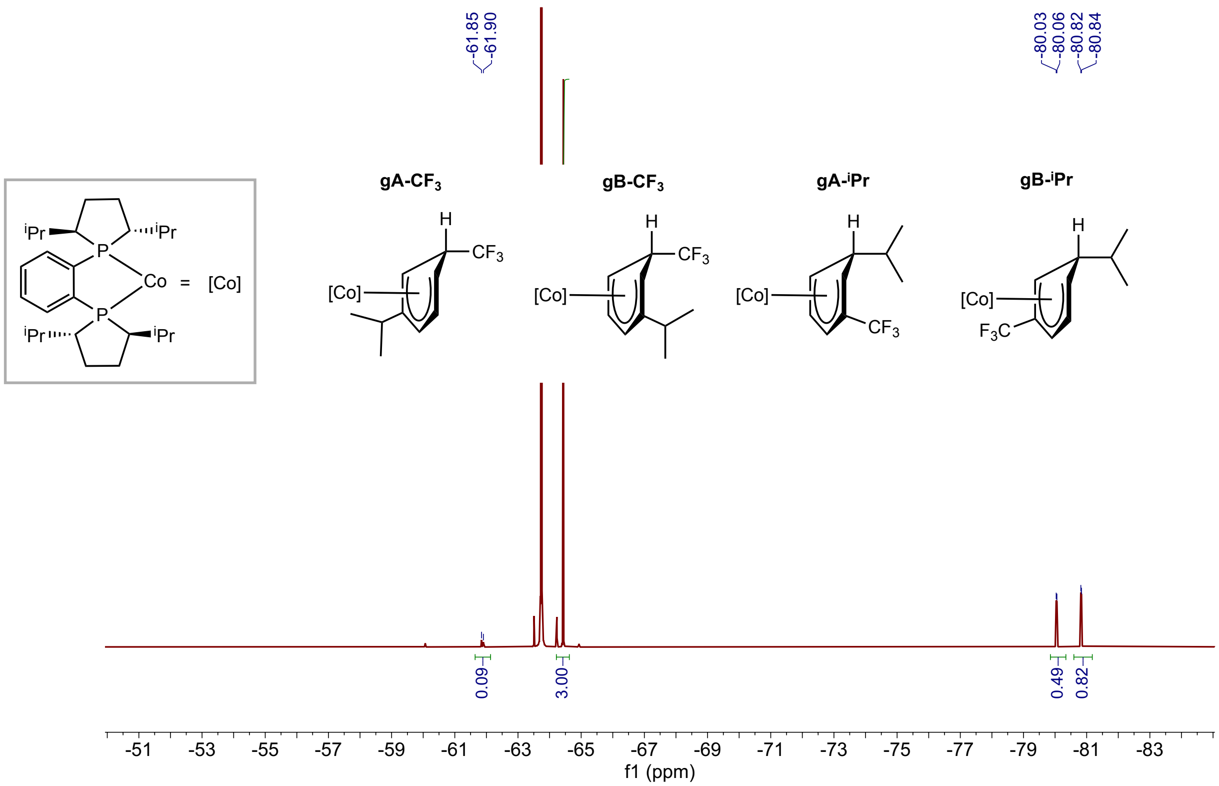

Figure S106. ^{19}F NMR spectrum (376 MHz, cyclohexane-d_{12}, 23 °C) of **Co1-g** with 1,3,5-tris(trifluoromethyl)benzene internal standard (δ -64.43 ppm) and excess arene (δ -63.75 ppm).

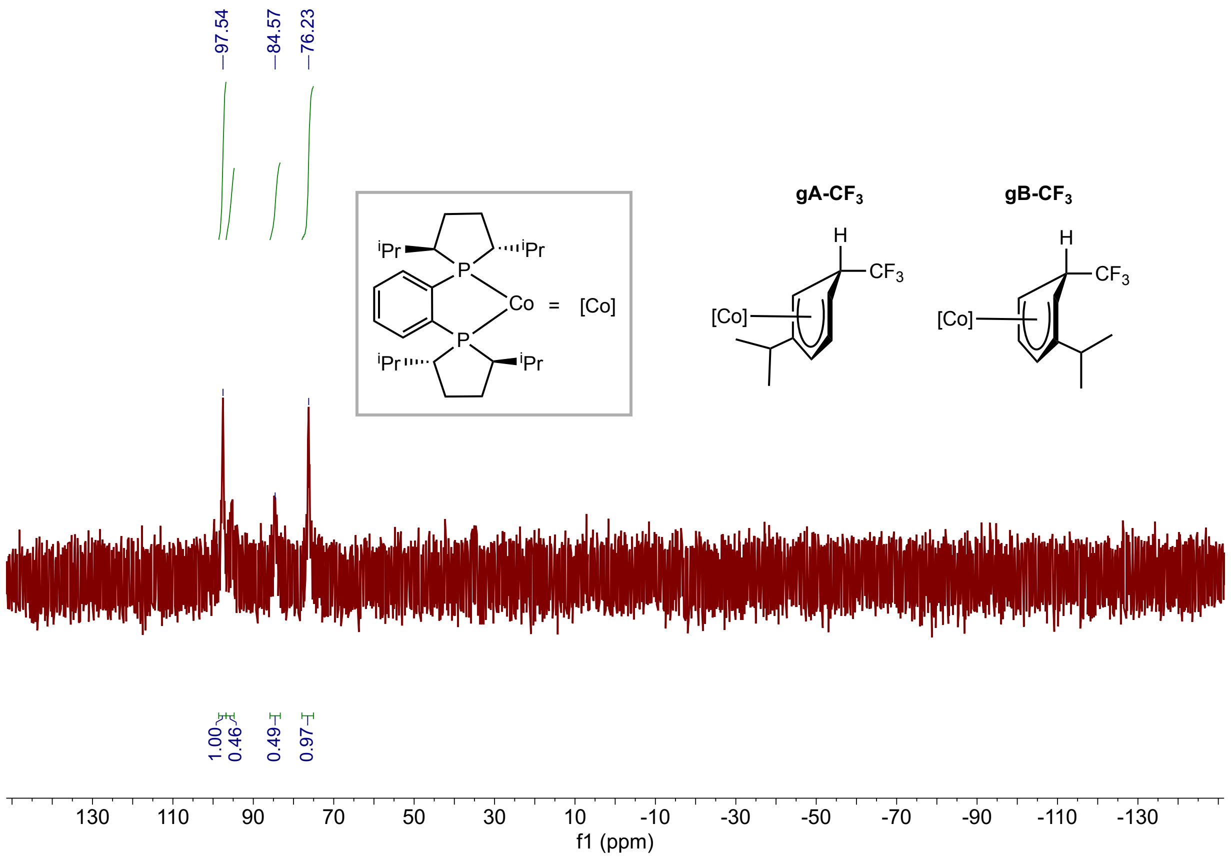

Figure S107. $^{31}P\{^{1}H\}$ NMR spectrum (162 MHz, cyclohexane-d_{12}, 23 °C) of isolated **Co1-g**.

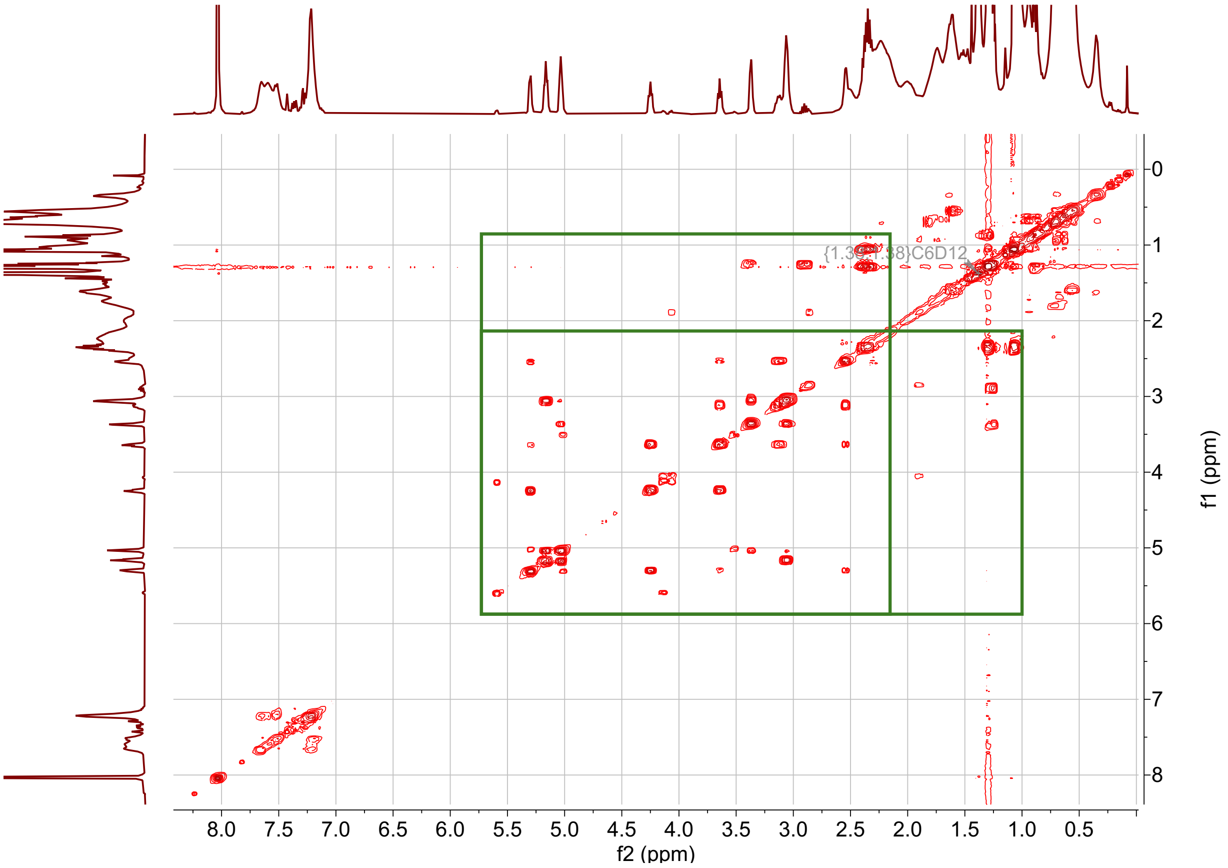

Figure S108. ^1H–^1H COSY NMR spectrum (cyclohexane-d_{12}, 23 °C) of isolated **Co1-g** with 1,3,5-tris(trifluoromethyl)benzene. Inset: ^1H–^1H correlation between η^5-cyclohexadienyl signals.

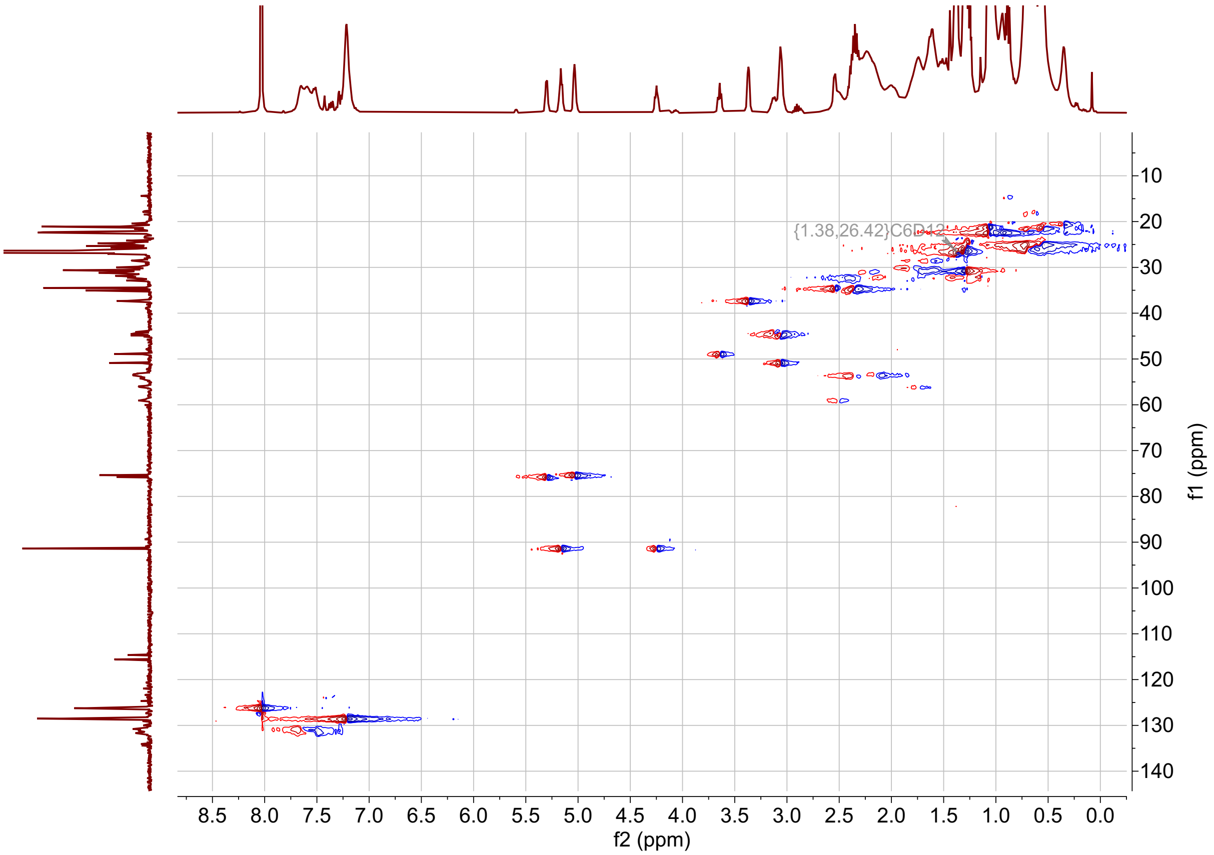

Figure S109. $^{1}H–^{13}C\{^{1}H\}$ HSQC NMR spectrum (cyclohexane-d_{12}, 23 °C) of isolated **Co1-g** with 1,3,5-tris(trifluoromethyl)benzene.

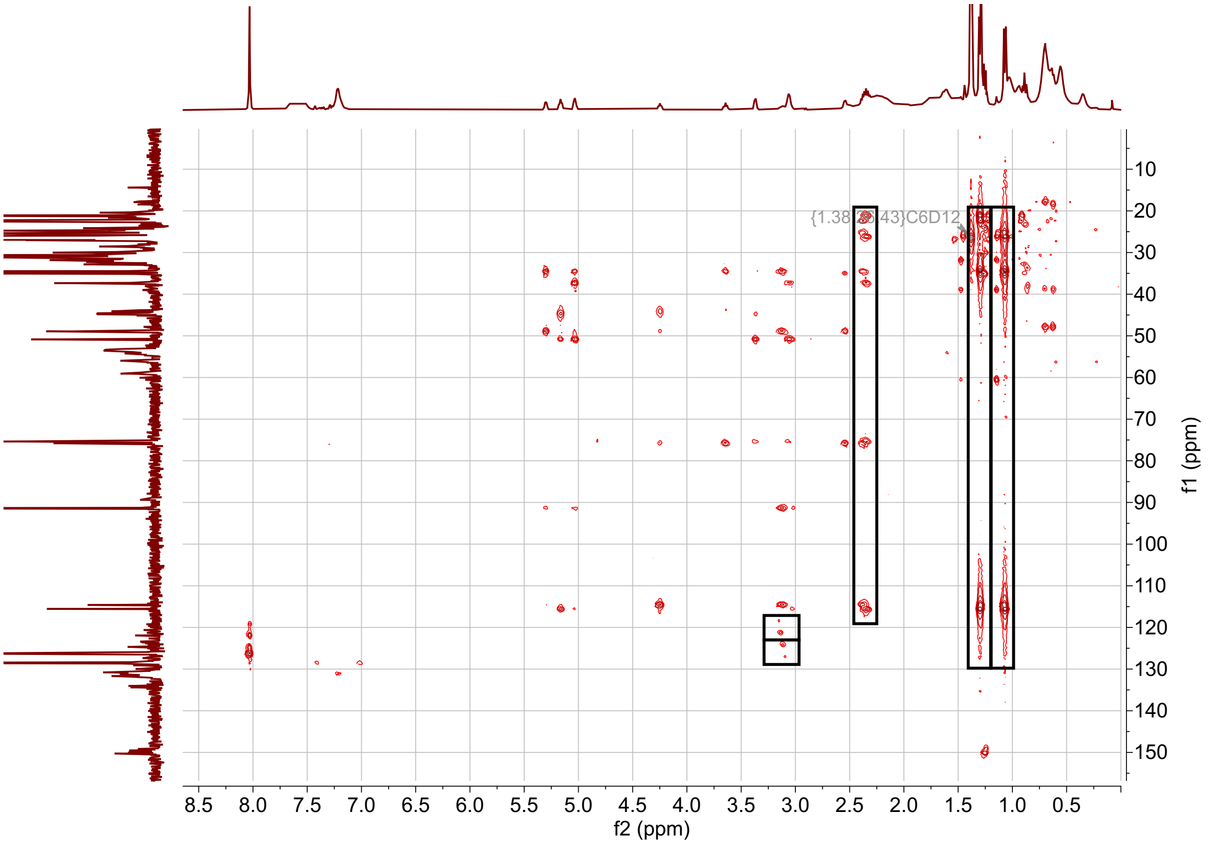

Figure S110. ^{1}H–$^{13}C\{^{1}H\}$ HMBC NMR spectrum (cyclohexane-d_{12}, 23 °C) of **Co1-g** with 1,3,5-tris(trifluoromethyl)benzene. Inset: assignment of quaternary carbons and C(sp^{2})-^{i}Pr correlation.

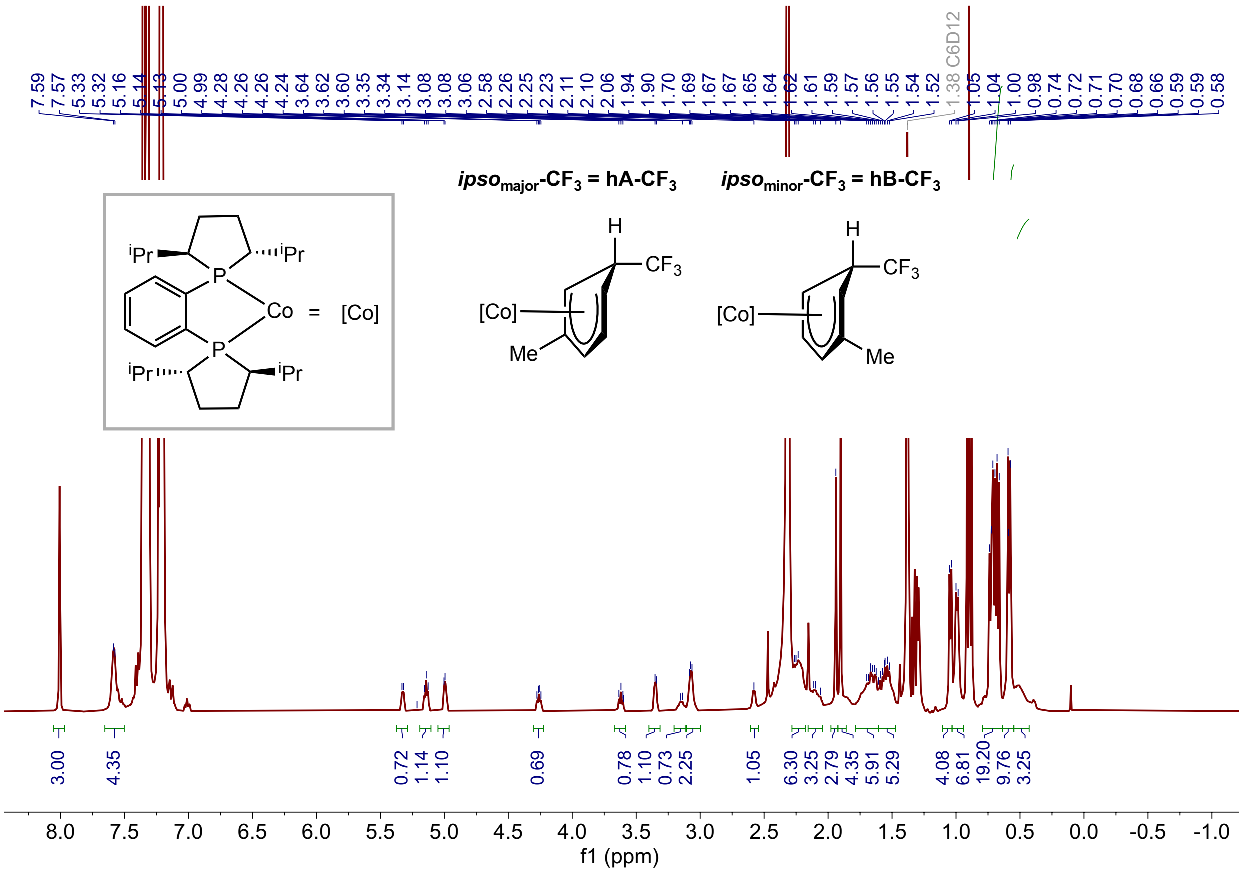

Figure S111. ^{1}H NMR spectrum (400 MHz, cyclohexane-d_{12}, 23 °C) of **Co1-h** with 1,3,5-tris(trifluoromethyl)benzene internal standard (δ 8.03 ppm) and excess arene.

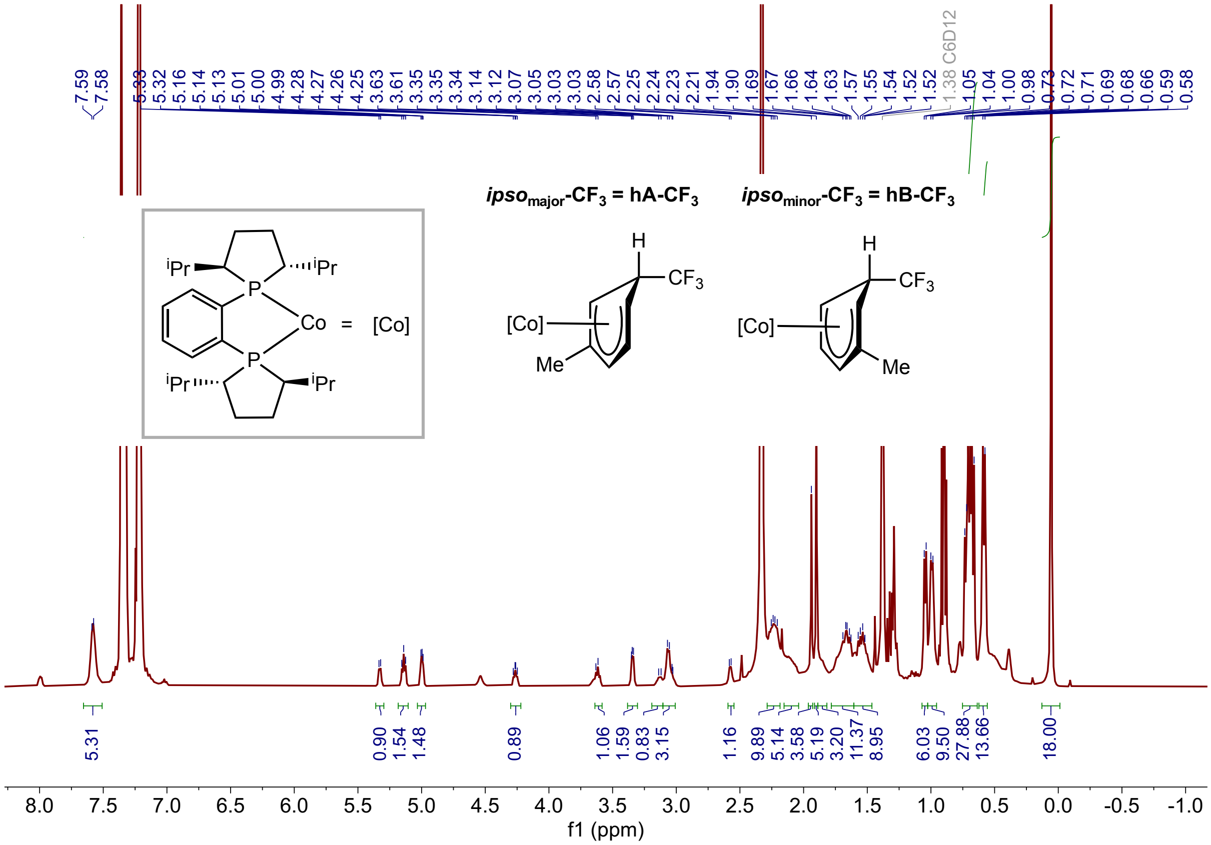

Figure S112. ^{1}H NMR spectrum (400 MHz, cyclohexane-d_{12}, 23 °C) of **Co1-h** with HMDSO internal standard (δ 0.10 ppm) and excess arene.

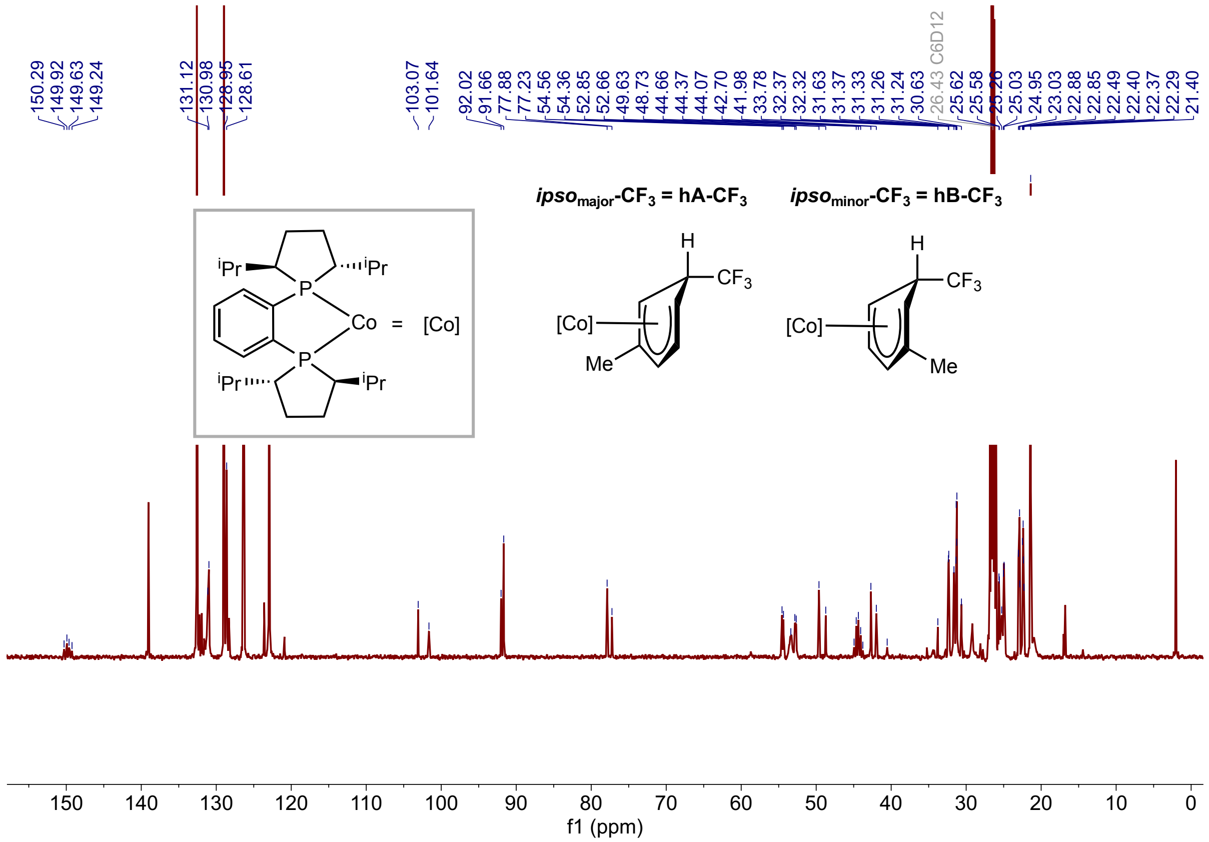

Figure S113. ${}^{13}C\{{}^{1}H\}$ NMR spectrum (101 MHz, cyclohexane-d_{12}, 23 °C) of **Co1-h** with HMDSO internal standard and excess arene.

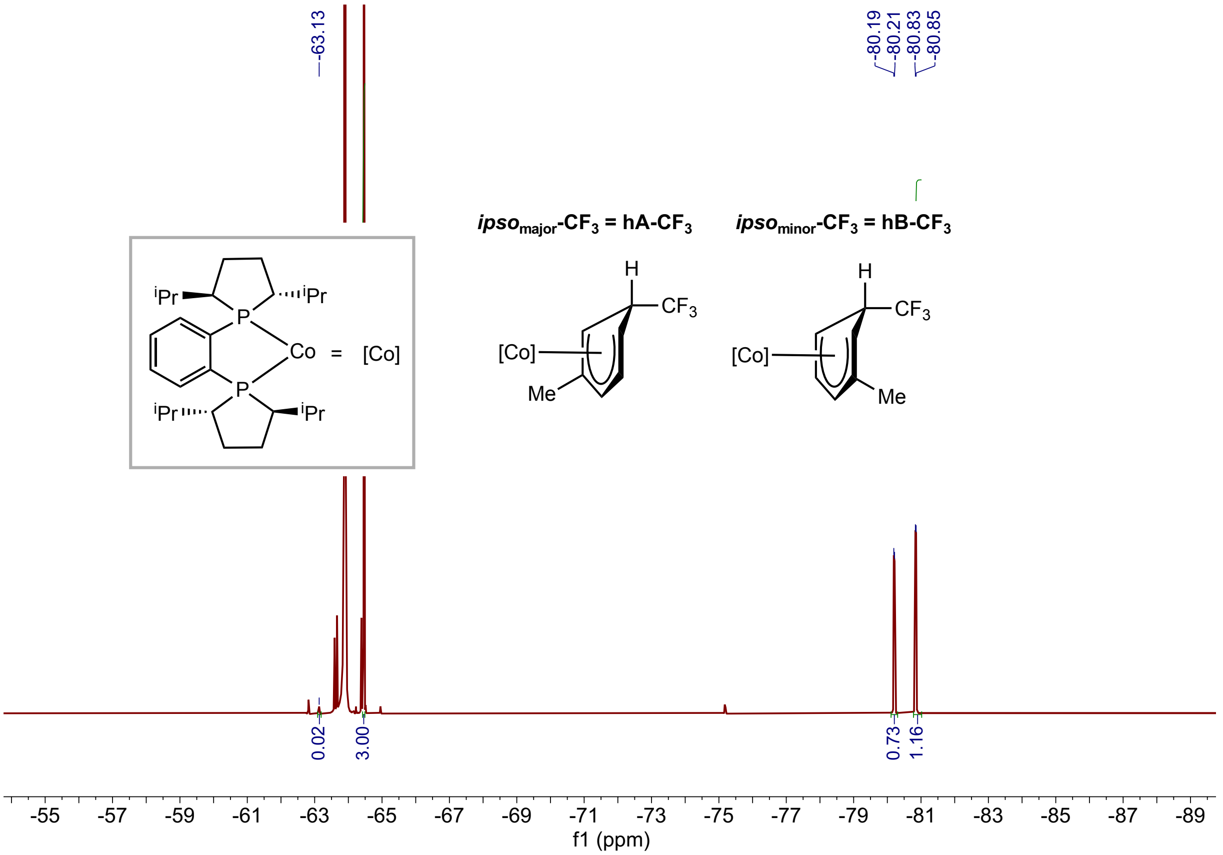

Figure S114. ^{19}F NMR spectrum (376 MHz, cyclohexane-d_{12}, 23 °C) of **Co1-h** with 1,3,5-tris(trifluoromethyl)benzene internal standard (δ -64.46 ppm) and excess arene (δ -63.90 ppm).

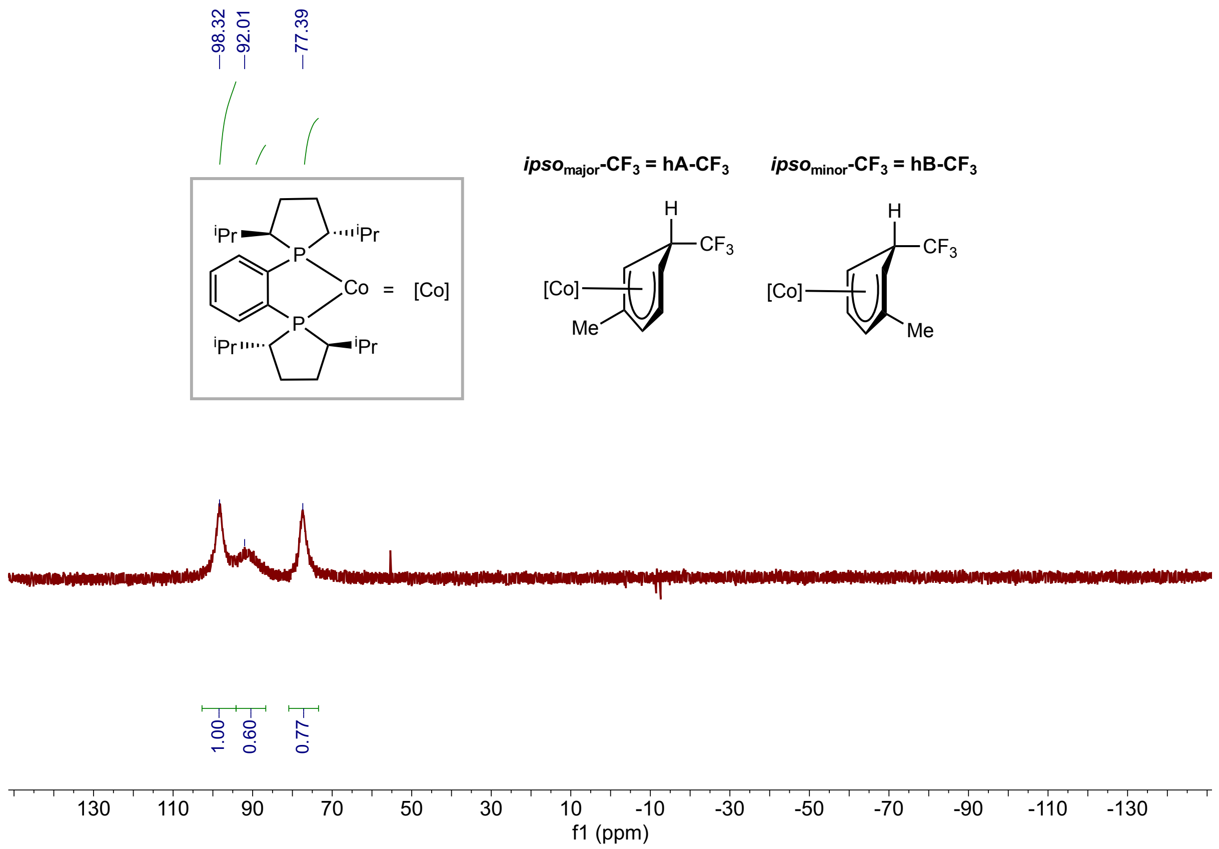

Figure S115. $^{31}P\{^{1}H\}$ NMR spectrum (162 MHz, cyclohexane-d_{12}, 23 °C) of **Co1-h**.

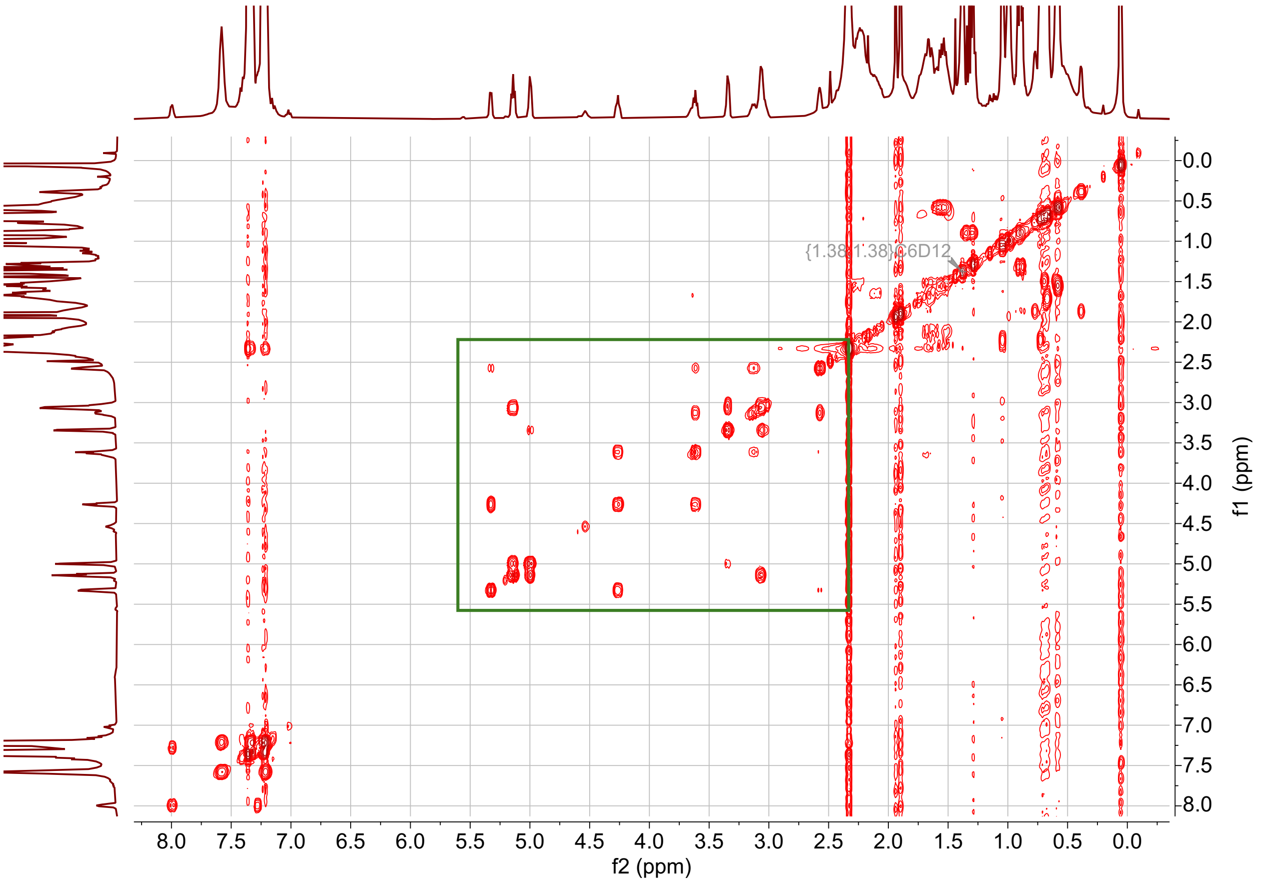

Figure S116. ^{1}H–^{1}H COSY NMR spectrum (cyclohexane-d_{12}, 23 °C) of **Co1-h** with HMDSO and excess arene. Inset: ^{1}H–^{1}H correlation between η^5-cyclohexadienyl signals.

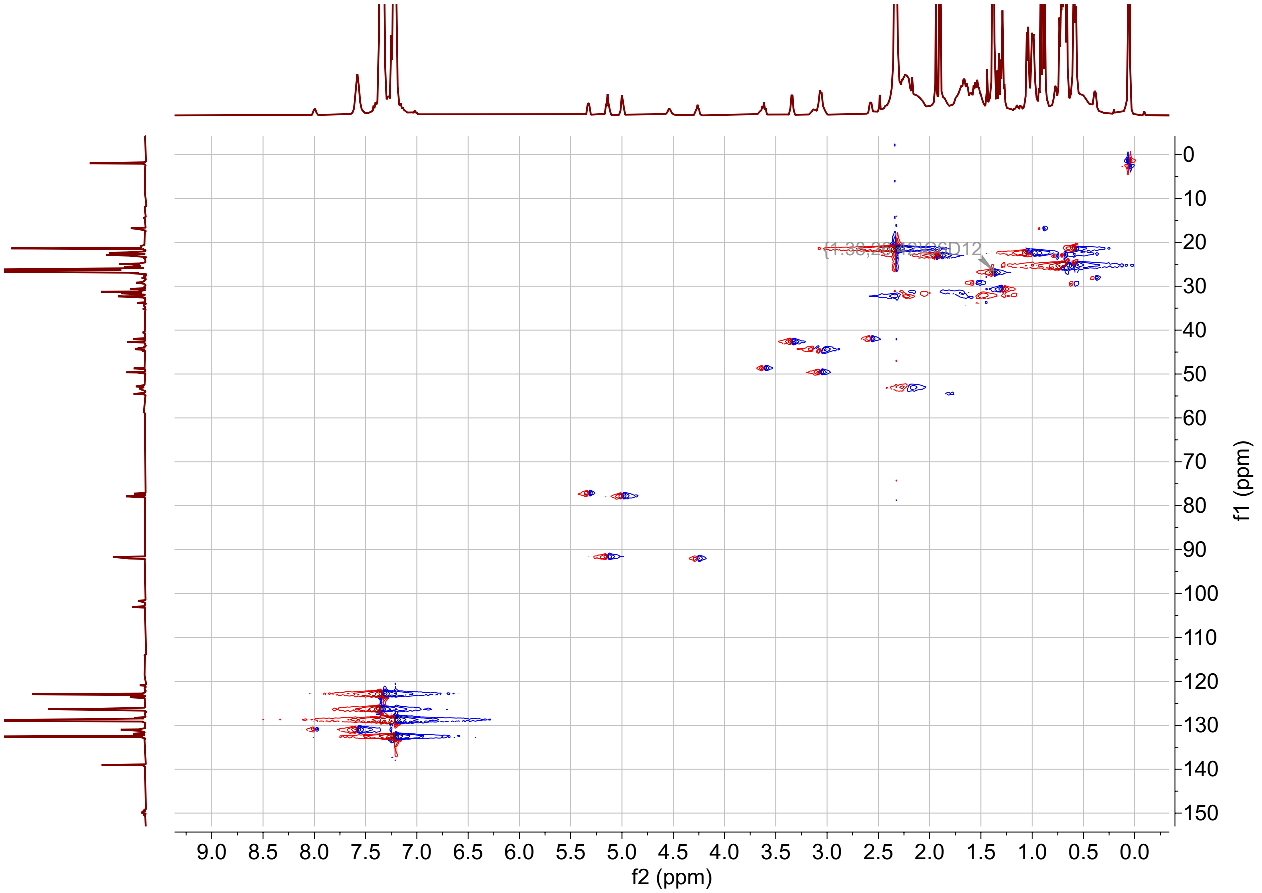

Figure S117. ^{1}H–$^{13}C\{^{1}H\}$ HSQC NMR spectrum (cyclohexane-d_{12}, 23 °C) of **Co1-h** with HMDSO and excess arene.

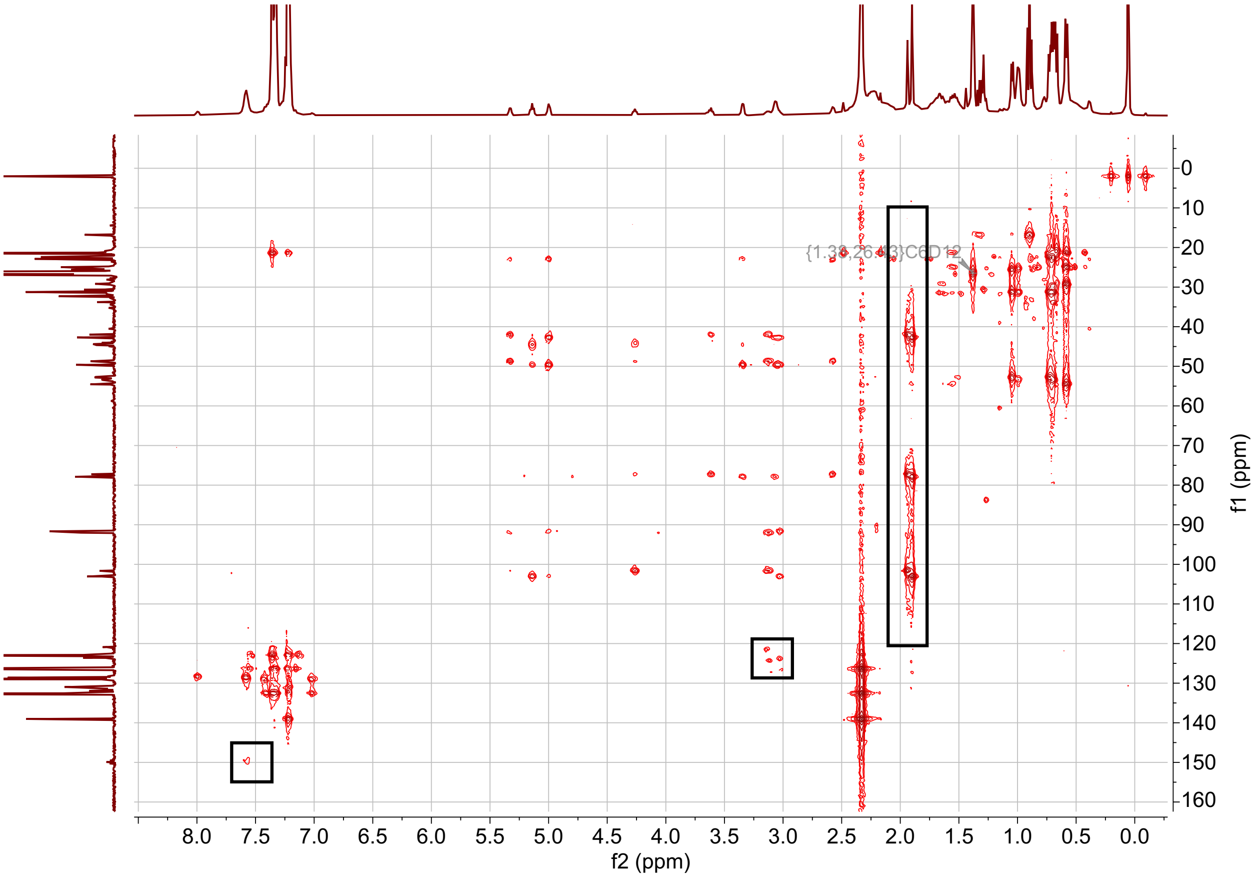

Figure S118. ^{1}H–$^{13}C\{^{1}H\}$ HMBC NMR spectrum (cyclohexane-d_{12}, 23 °C) of **Co1-h** with HMDSO and excess arene. Inset: assignment of quaternary carbons and C(sp^{2})-Me correlation.

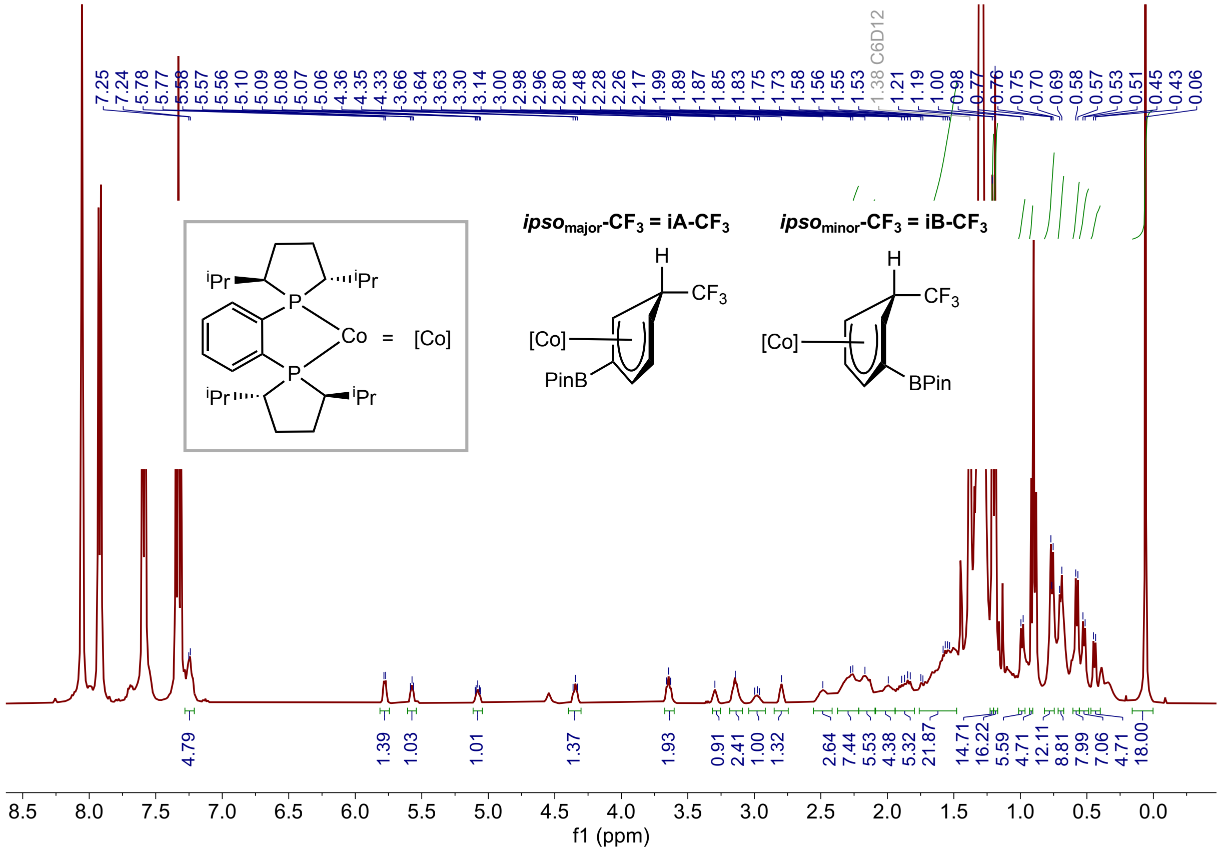

Figure S119. ^{1}H NMR spectrum (400 MHz, cyclohexane-d_{12}, 23 °C) of **Co1-i** with HMDSO internal standard (δ 0.10 ppm) and excess arene.

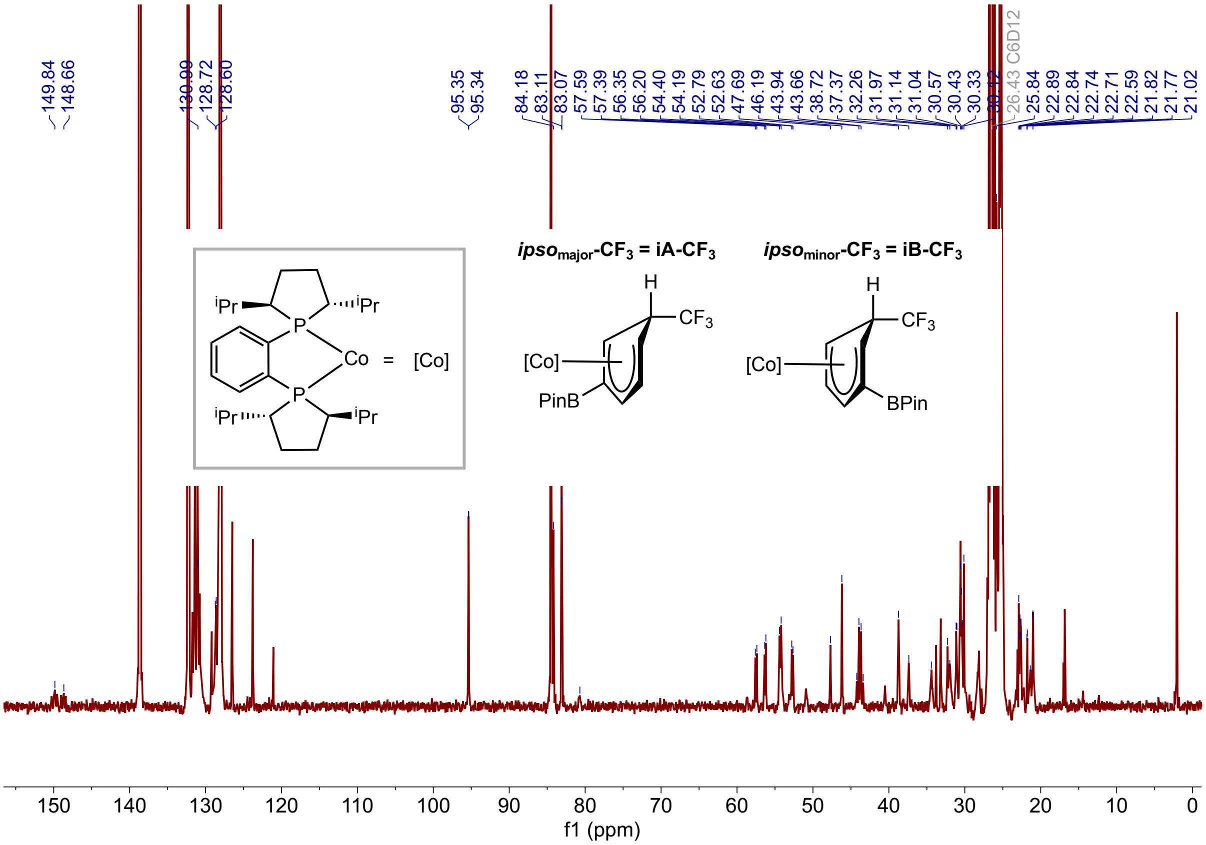

Figure S120. $^{13}C\{^{1}H\}$ NMR spectrum (101 MHz, cyclohexane-d_{12}, 23 °C) of **Co1-i** with HMDSO and excess arene.

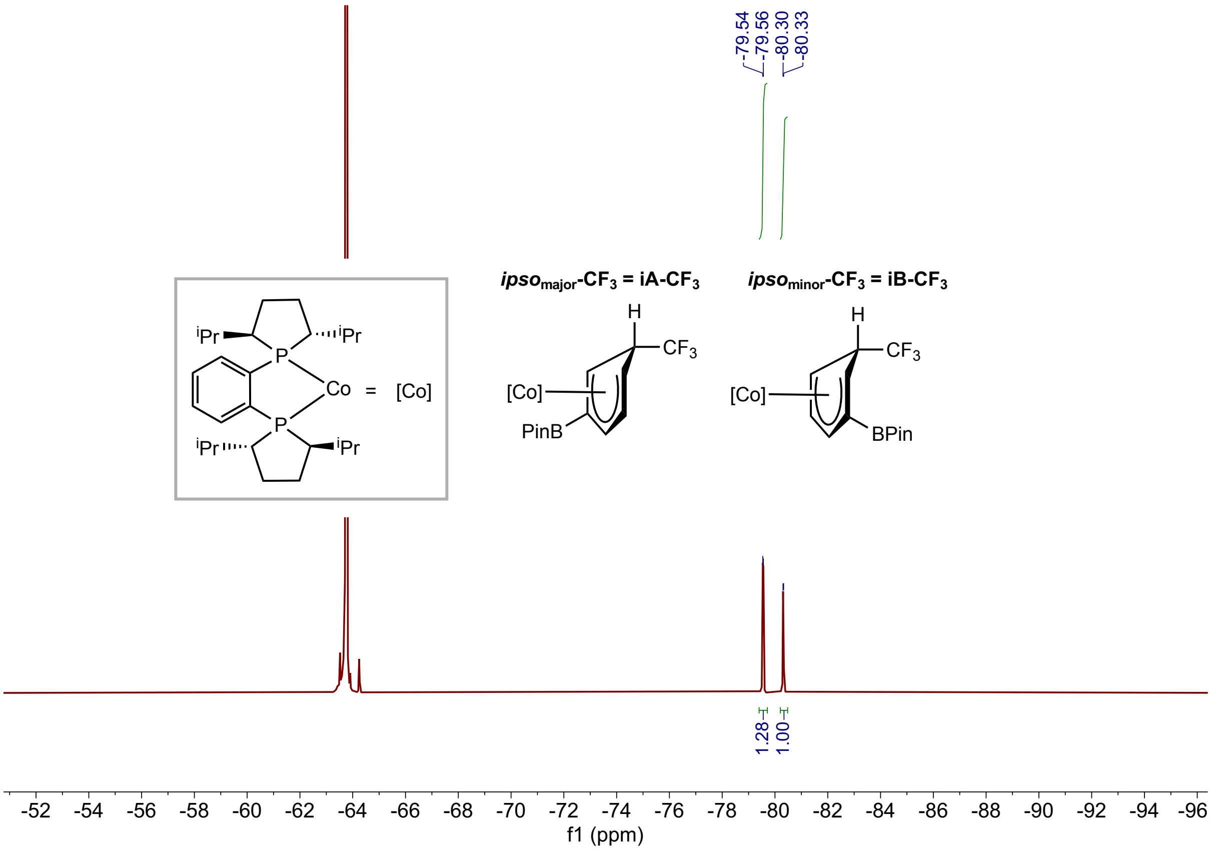

Figure S121. ^{19}F NMR spectrum (376 MHz, cyclohexane-d_{12}, 23 °C) of **Co1-i** in excess arene (δ -63.75 ppm).

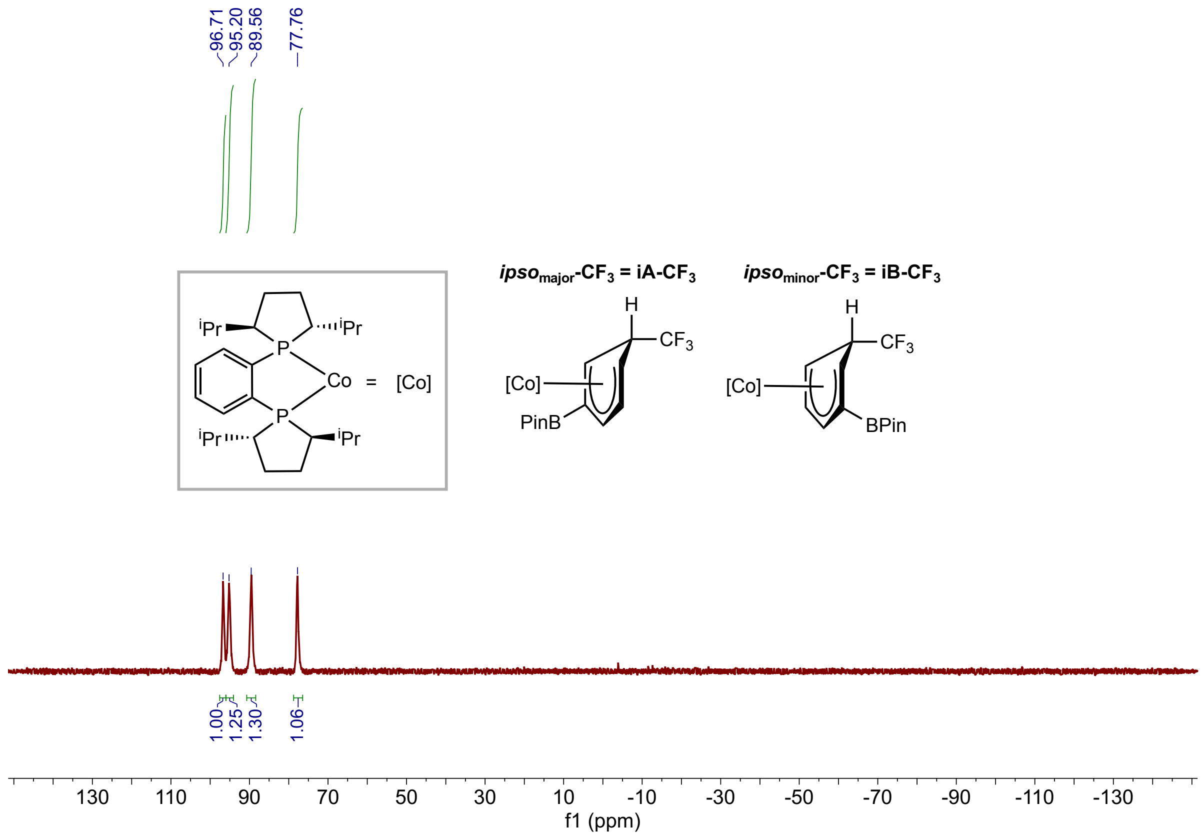

Figure S122. $^{31}P\{^{1}H\}$ NMR spectrum (162 MHz, cyclohexane-d_{12}, 23 °C) of **Co1-i**.

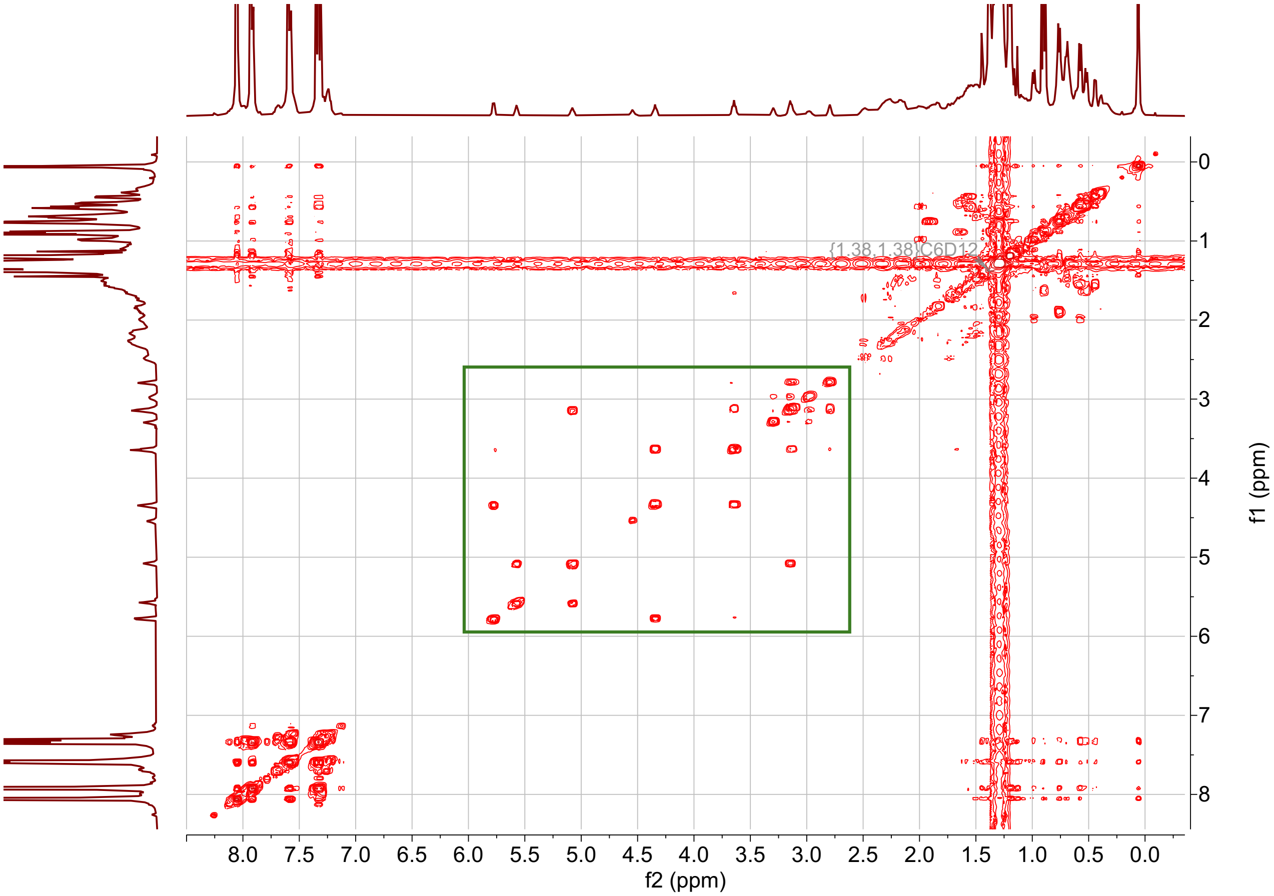

Figure S123. $^1H–^1H$ COSY NMR spectrum (cyclohexane-d_{12}, 23 °C) of **Co1-i** with HMDSO and excess arene. Inset: $^1H–^1H$ correlation between η^5-cyclohexadienyl signals.

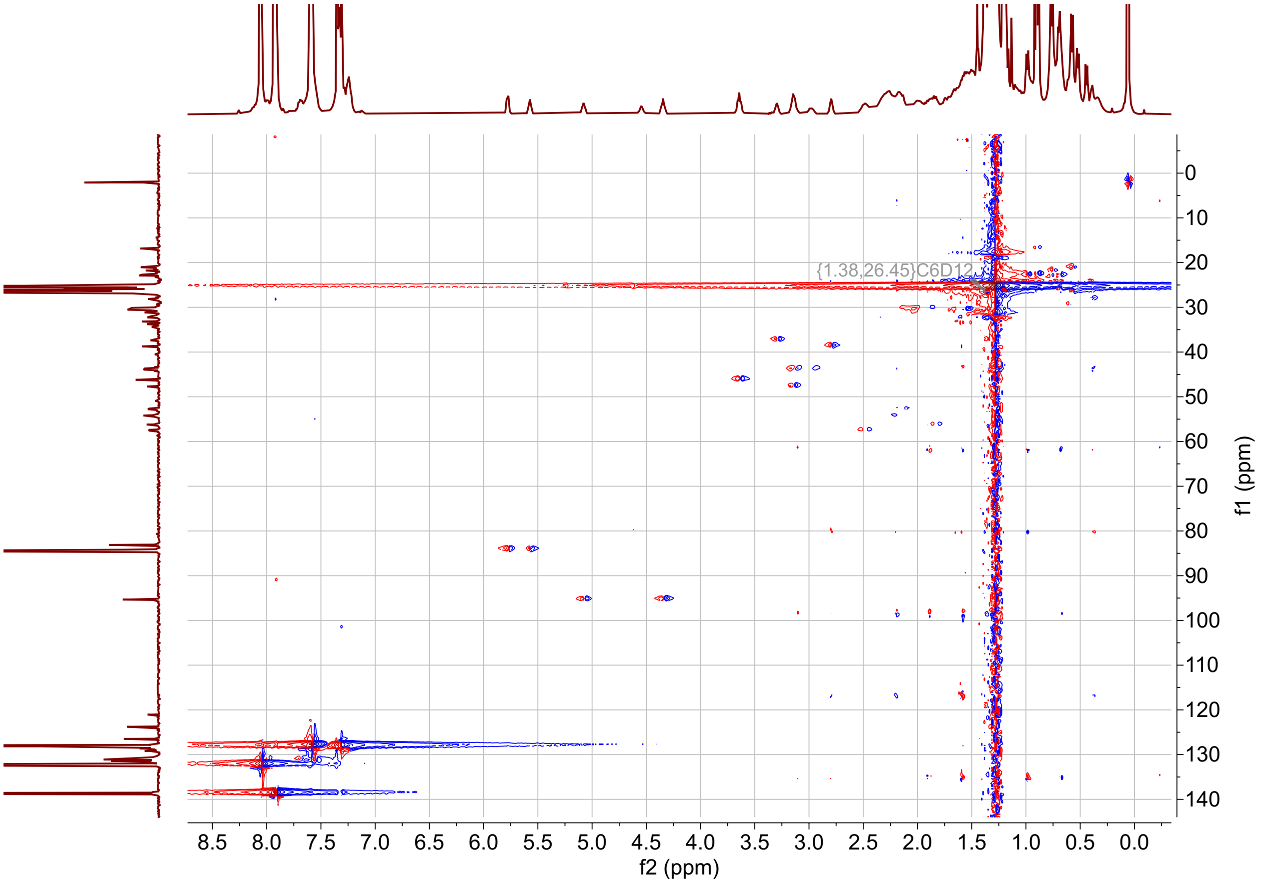

Figure S124. $^{1}H–^{13}C\{^{1}H\}$ HSQC NMR spectrum (cyclohexane-d_{12}, 23 °C) of **Co1-i** with HMDSO and excess arene.

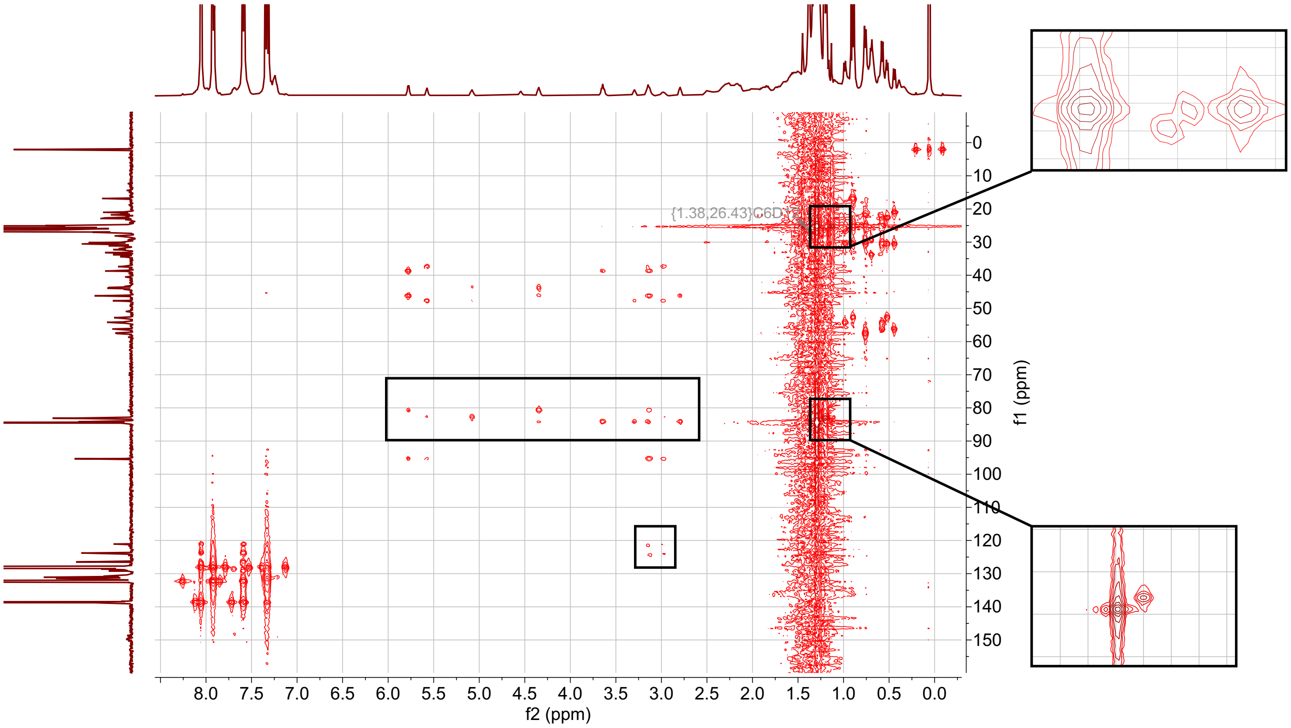

Figure S125. ^{1}H–$^{13}C\{^{1}H\}$ HMBC NMR spectrum (cyclohexane-d_{12}, 23 °C) of **Co1-i** with HMDSO and excess arene. Inset: assignment of quaternary carbons and C(sp^{2})-BPin correlation.

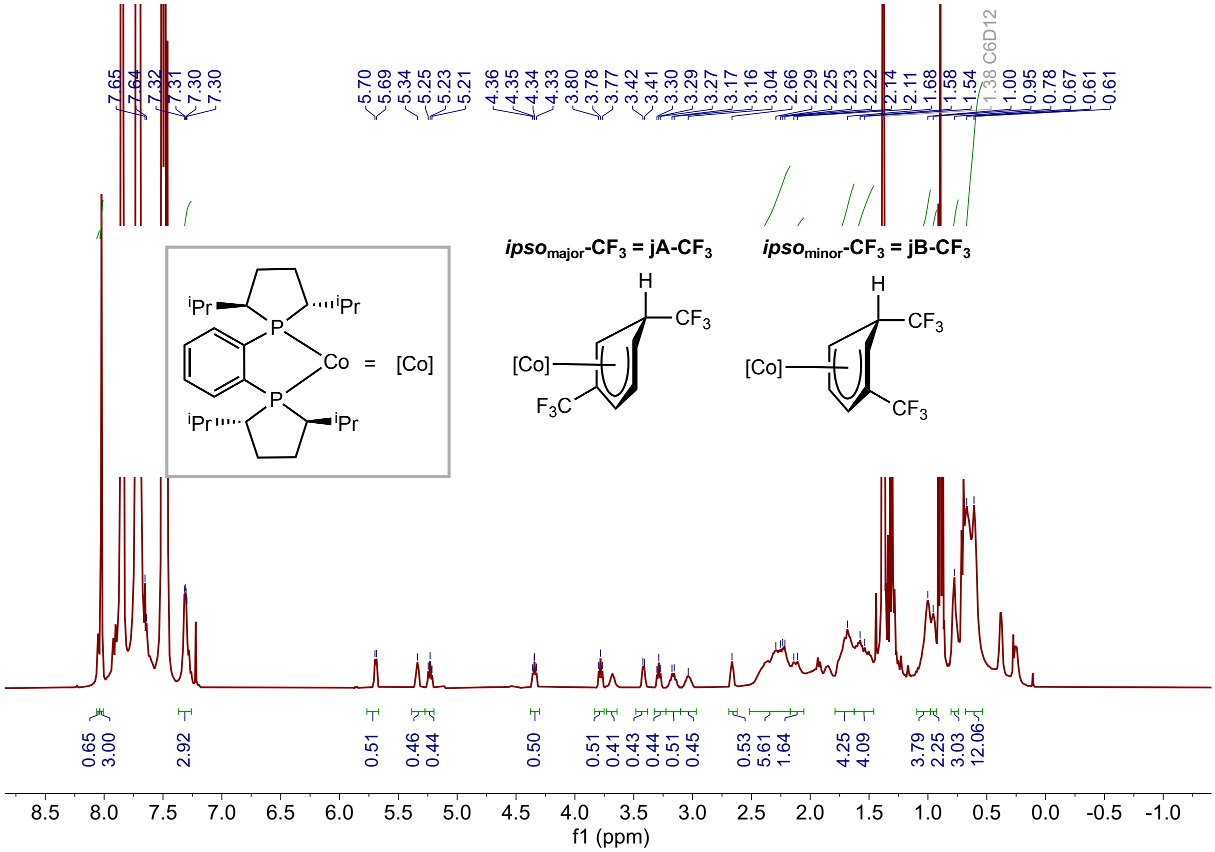

Figure S126. ^{1}H NMR spectrum (400 MHz, cyclohexane-d_{12}, 23 °C) of **Co1-j** with 1,3,5-tris(trifluoromethyl)benzene internal standard (δ 8.03 ppm) and excess arene.

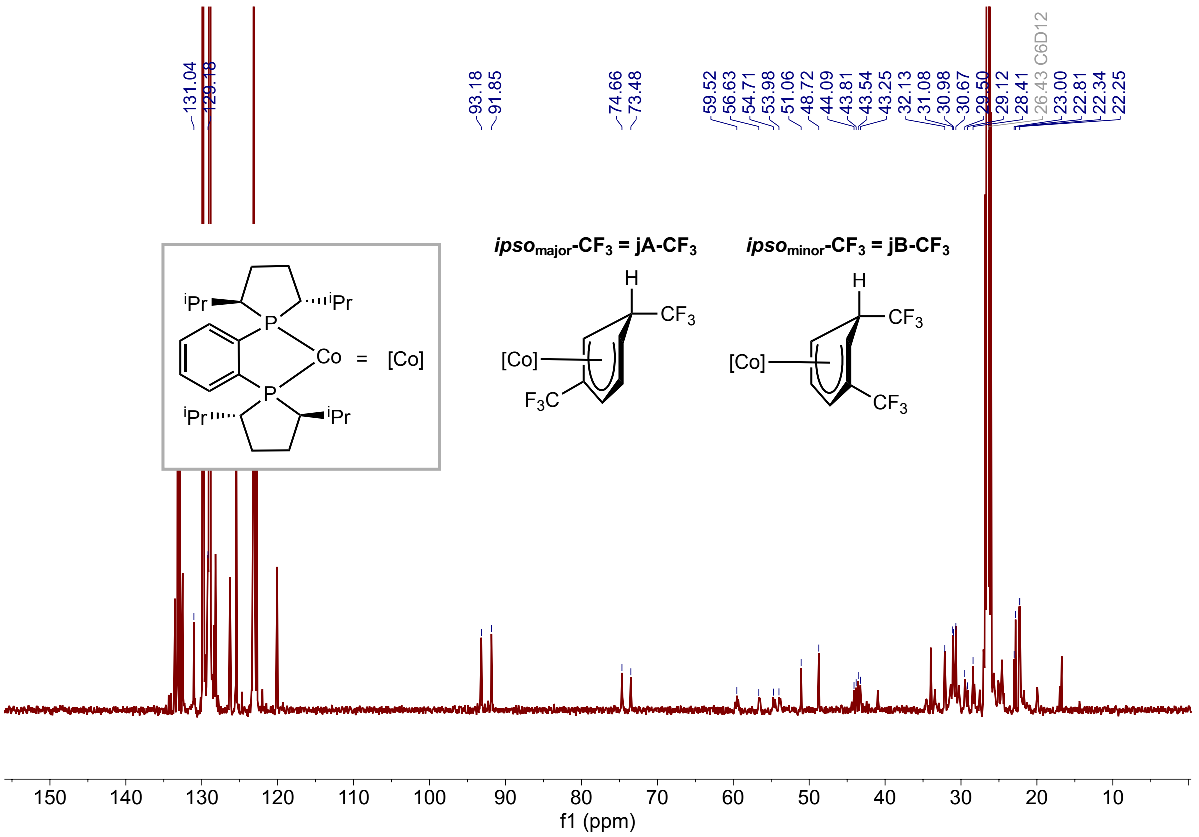

Figure S127. $^{13}C\{^{1}H\}$ NMR spectrum (101 MHz, cyclohexane-d_{12}, 23 °C) of **Co1-j** with 1,3,5-tris(trifluoromethyl)benzene and excess arene.

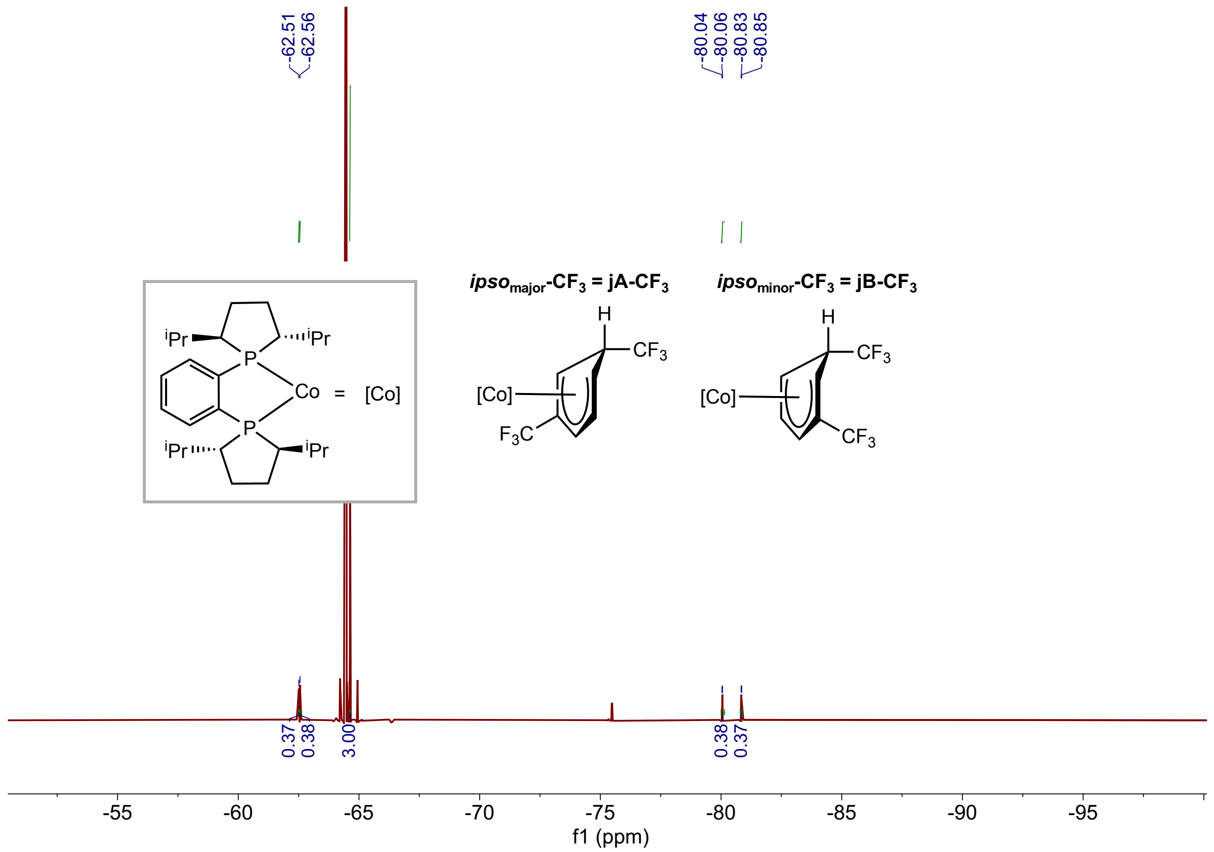

Figure S128. ^{19}F NMR spectrum (376 MHz, cyclohexane-d_{12}, 23 °C) of **Co1-j** with 1,3,5-tris(trifluoromethyl)benzene (δ -64.63 ppm) and excess arene (δ -64.45 ppm).

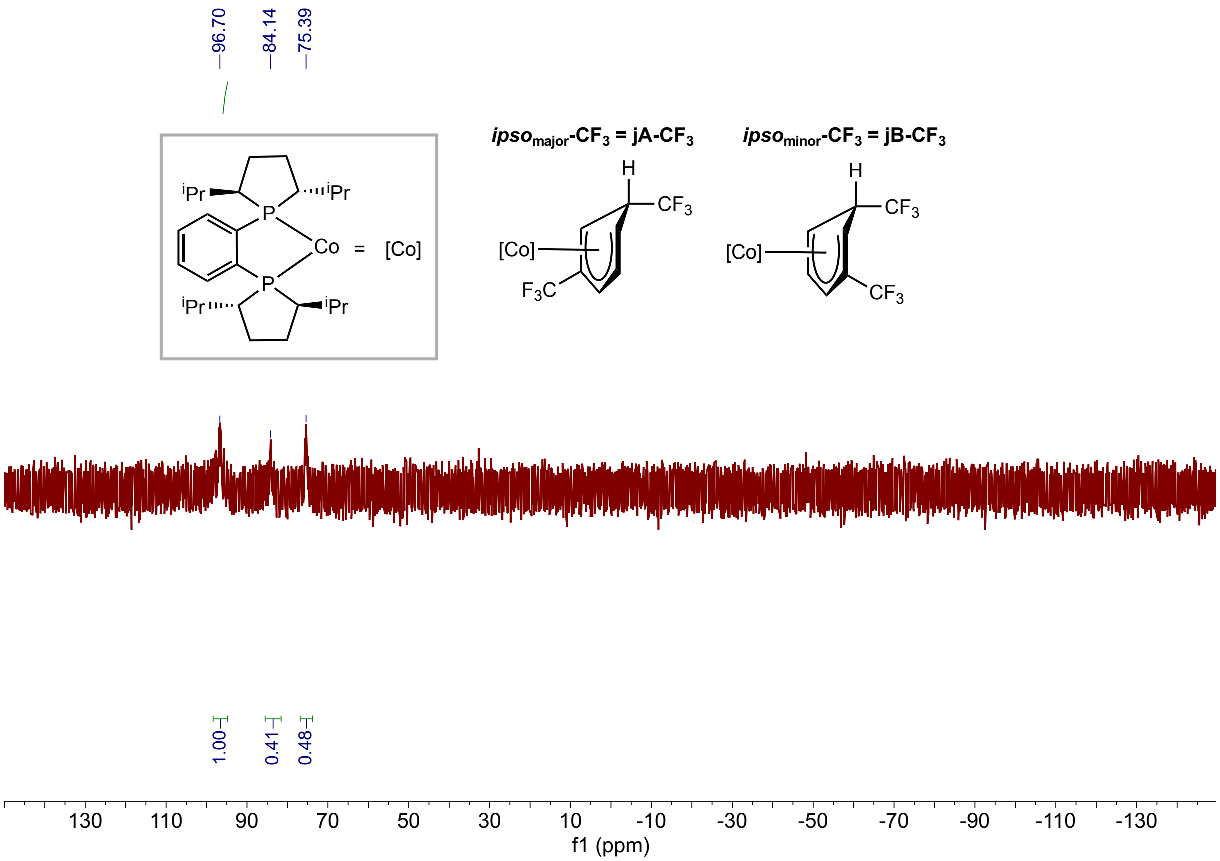

Figure S129. ^{31}P{^{1}H} NMR spectrum (162 MHz, cyclohexane-d_{12}, 23 °C) of **Co1-j**.

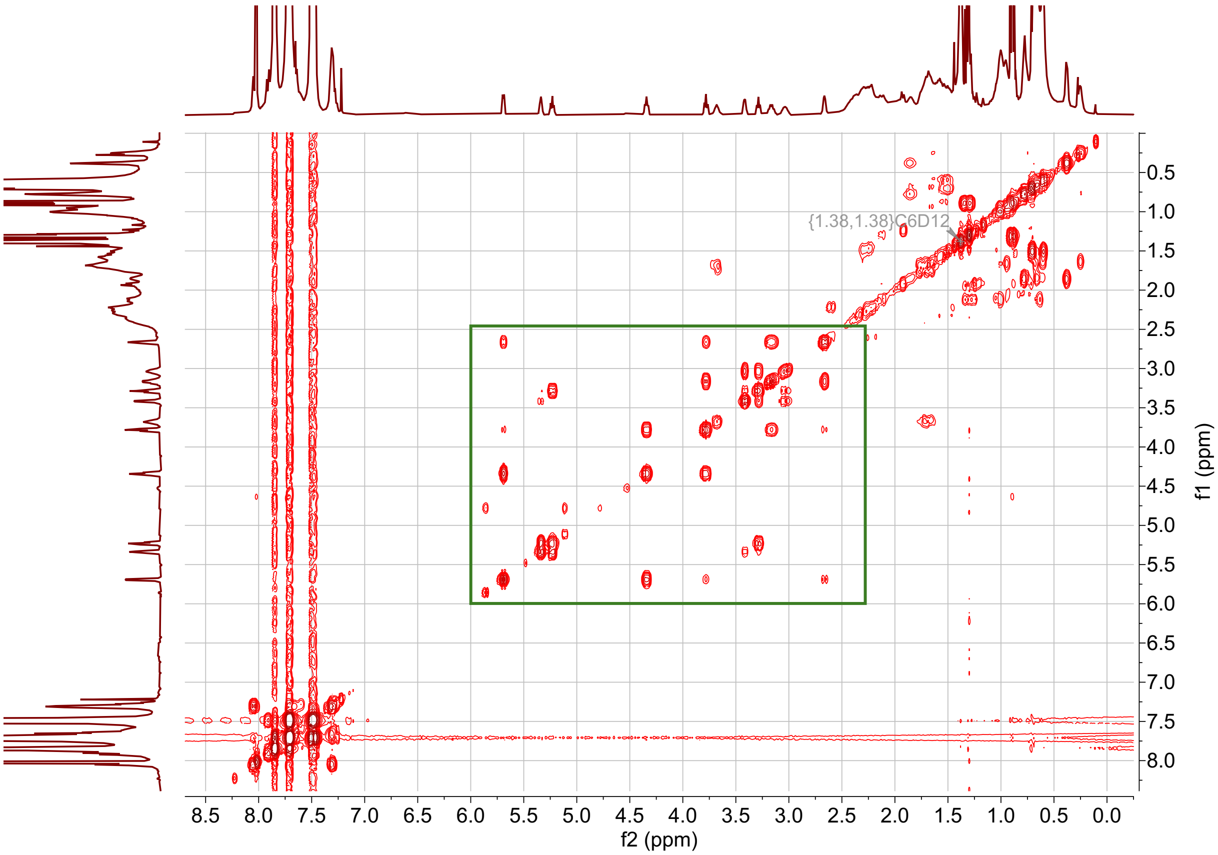

Figure S130. ^1H–^1H COSY NMR spectrum (cyclohexane-d_{12}, 23 °C) of **Co1-j** with 1,3,5-tris(trifluoromethyl)benzene and excess arene. Inset: ^1H–^1H correlation between η^5-cyclohexadienyl signals.

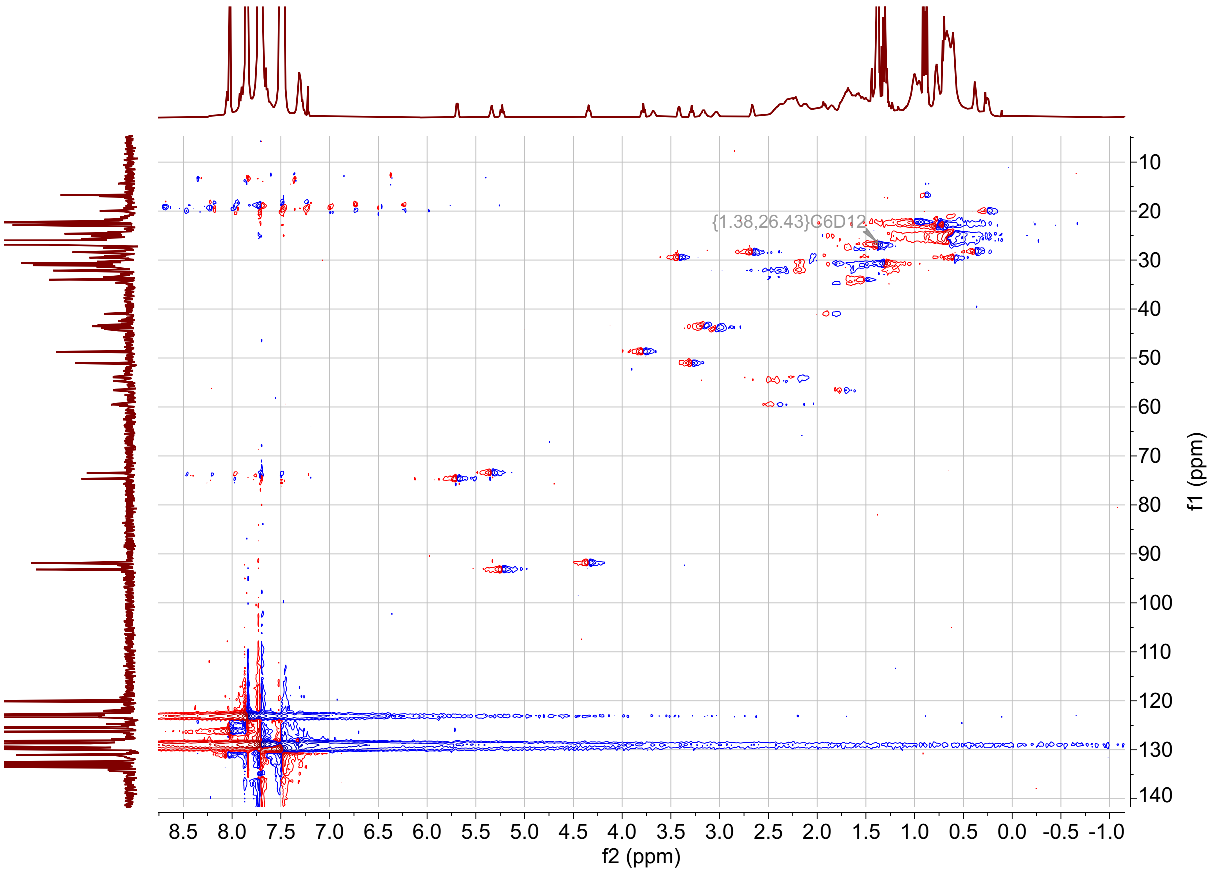

Figure S131. ^{1}H–$^{13}C\{^{1}H\}$ HSQC NMR spectrum (cyclohexane-d_{12}, 23 °C) of **Co1-j** with 1,3,5-tris(trifluoromethyl)benzene and excess arene.

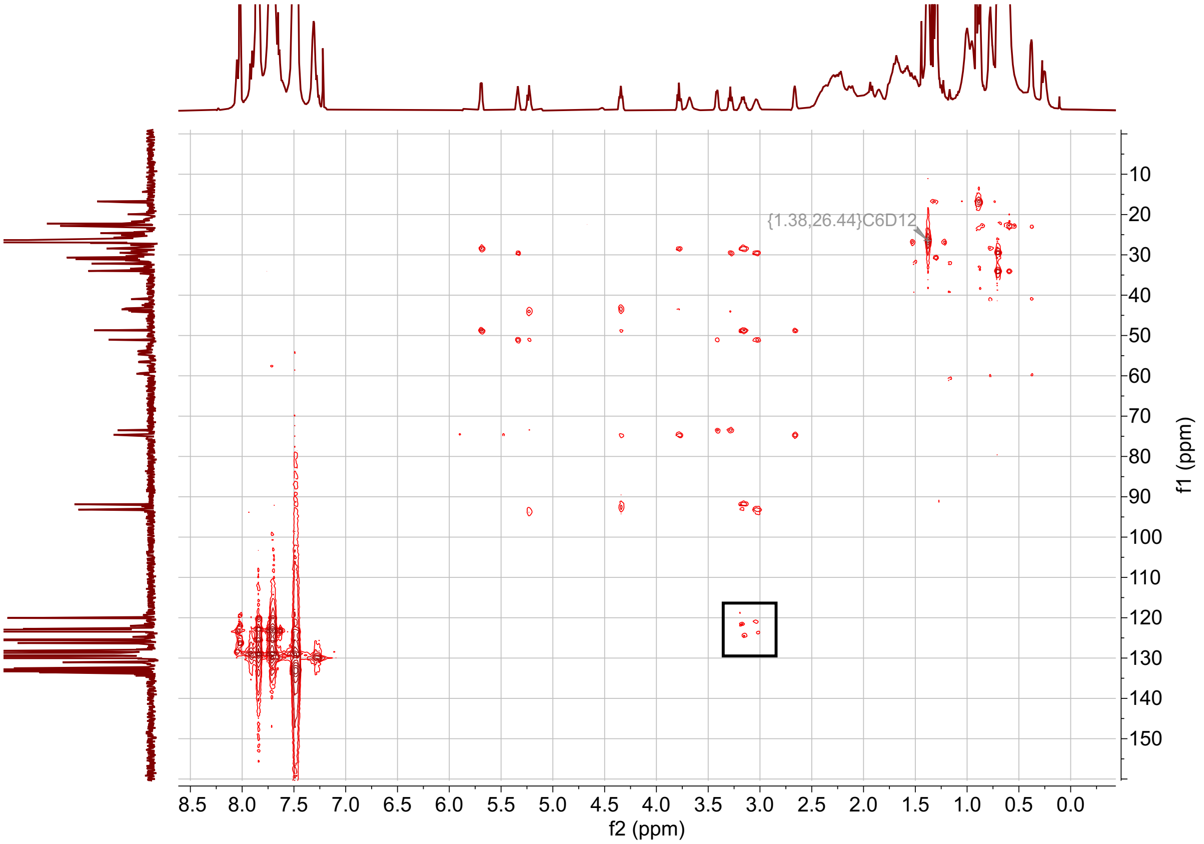

Figure S132. ^{1}H–$^{13}C\{^{1}H\}$ HMBC NMR spectrum (cyclohexane-d_{12}, 23 °C) of **Co1-j** with 1,3,5-tris(trifluoromethyl)benzene and excess arene. Inset: assignment of quaternary carbons.

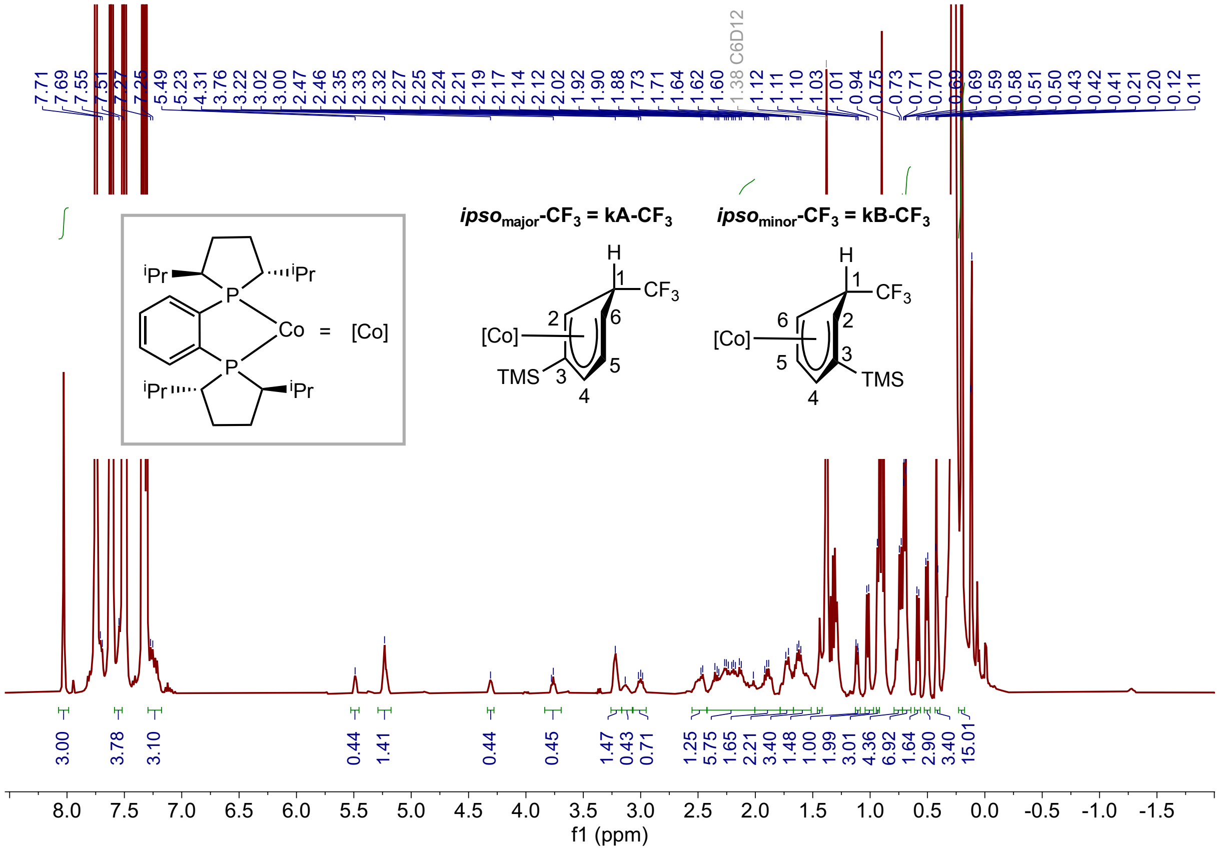

Figure S133. ^{1}H NMR spectrum (400 MHz, cyclohexane-d_{12}, 23 °C) of **Co1-k** with 1,3,5-tris(trifluoromethyl)benzene internal standard (δ 8.03 ppm) and excess arene.

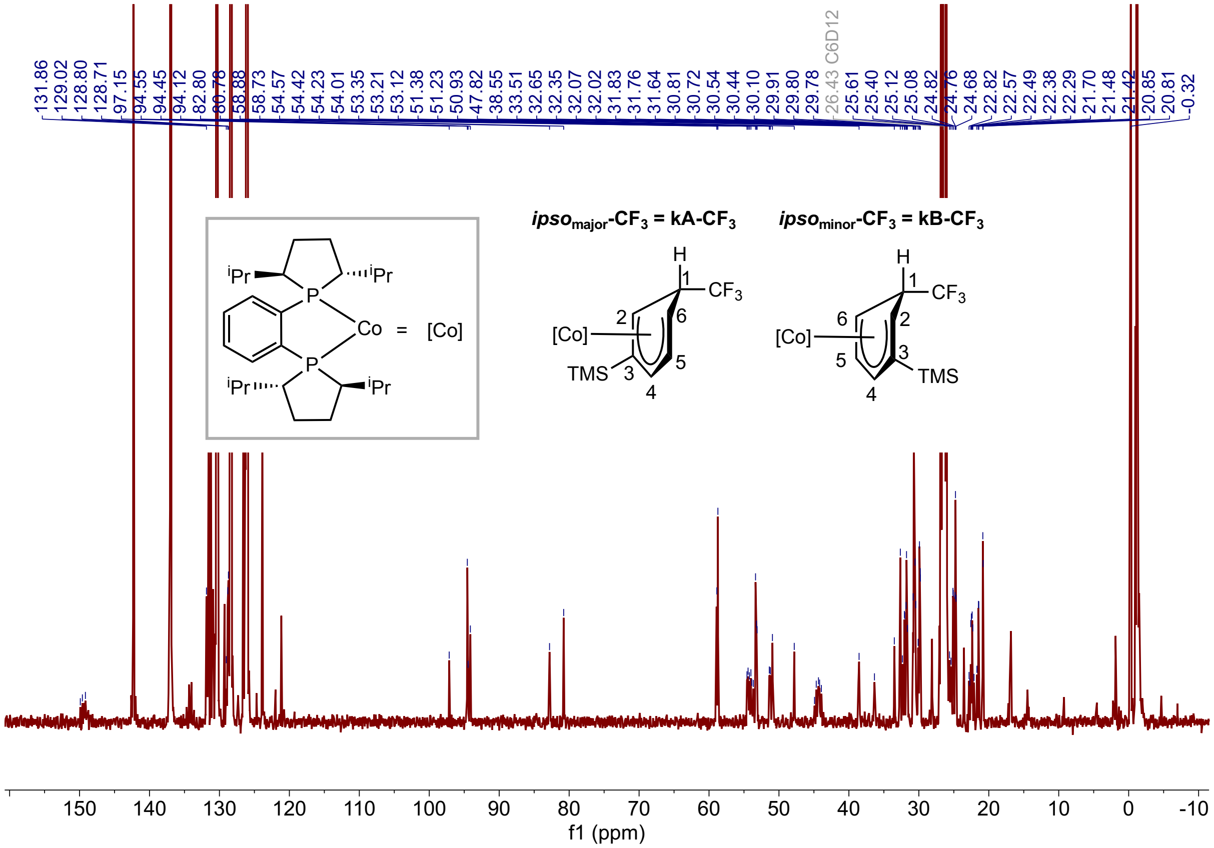

Figure S134. $^{13}C\{^1H\}$ NMR spectrum (101 MHz, cyclohexane-d_{12}, 23 °C) of **Co1-k** with 1,3,5-tris(trifluoromethyl)benzene and excess arene.

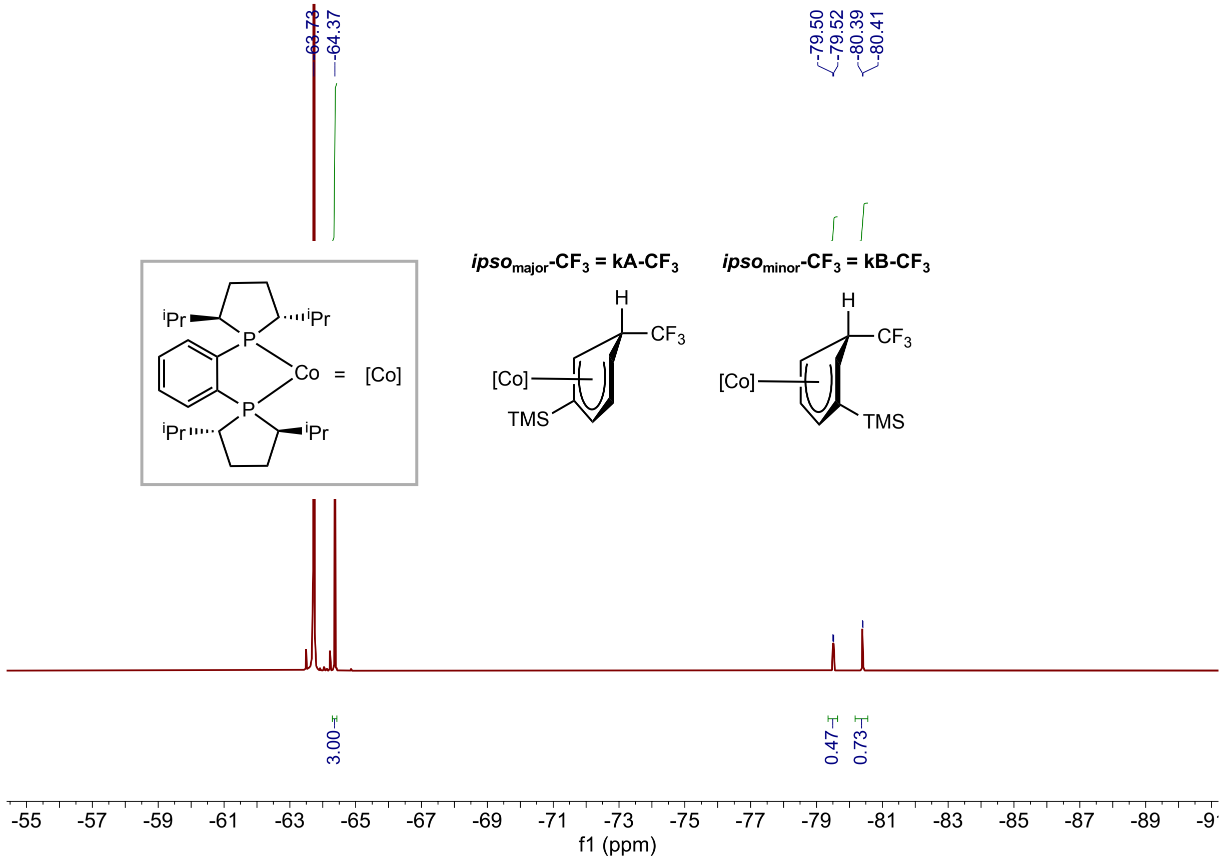

Figure S135. ^{19}F NMR spectrum (376 MHz, cyclohexane-d_{12}, 23 °C) of **Co1-k** with 1,3,5-tris(trifluoromethyl)benzene internal standard (δ -64.37 ppm) and excess arene (δ -63.73 ppm).

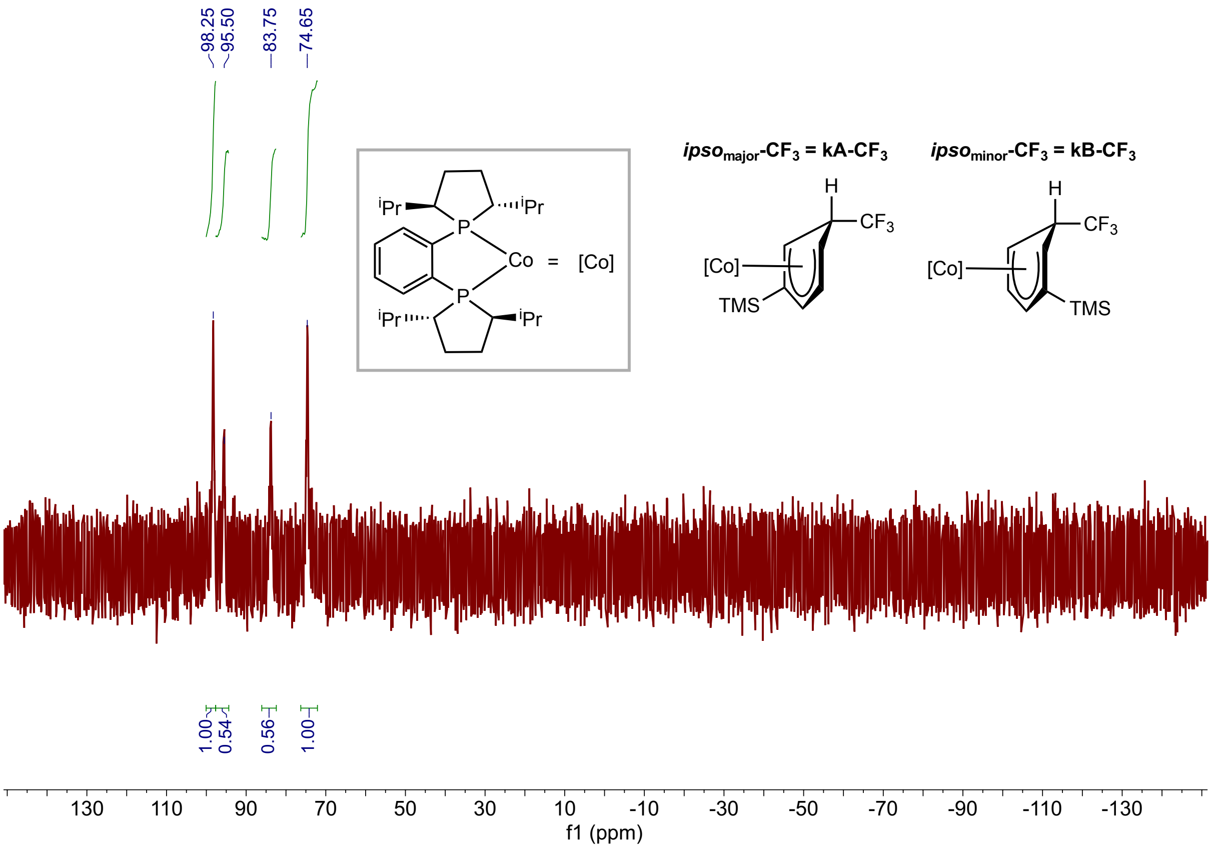

Figure S136. $^{31}P\{^1H\}$ NMR spectrum (162 MHz, cyclohexane-d_{12}, 23 °C) of **Co1-k**.

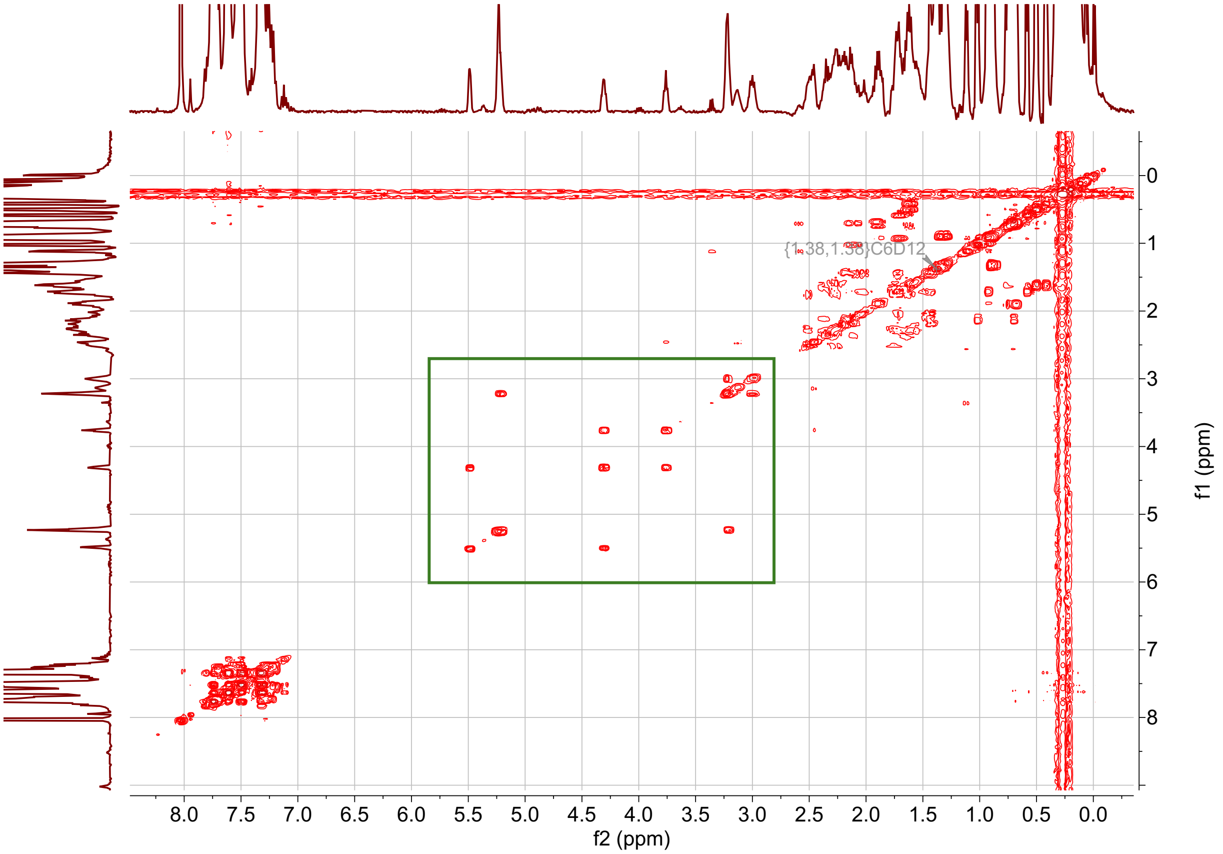

Figure S137. ^1H–^1H COSY NMR spectrum (cyclohexane-d_{12}, 23 °C) of **Co1-k** with 1,3,5-tris(trifluoromethyl)benzene and excess arene. Inset: ^1H–^1H correlation between η^5-cyclohexadienyl signals.

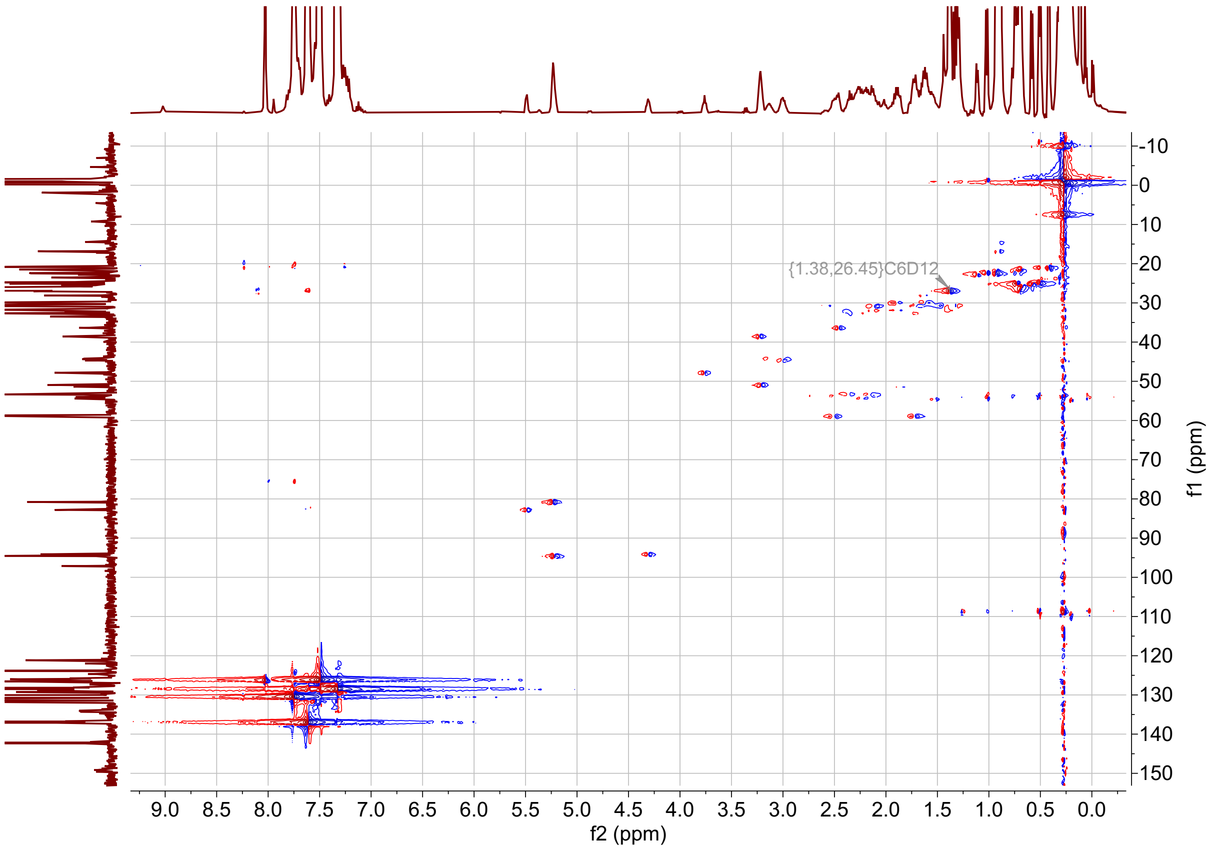

Figure S138. ^{1}H–$^{13}C\{^{1}H\}$ HSQC NMR spectrum (cyclohexane-d_{12}, 23 °C) of **Co1-k** with 1,3,5-tris(trifluoromethyl)benzene and excess arene.

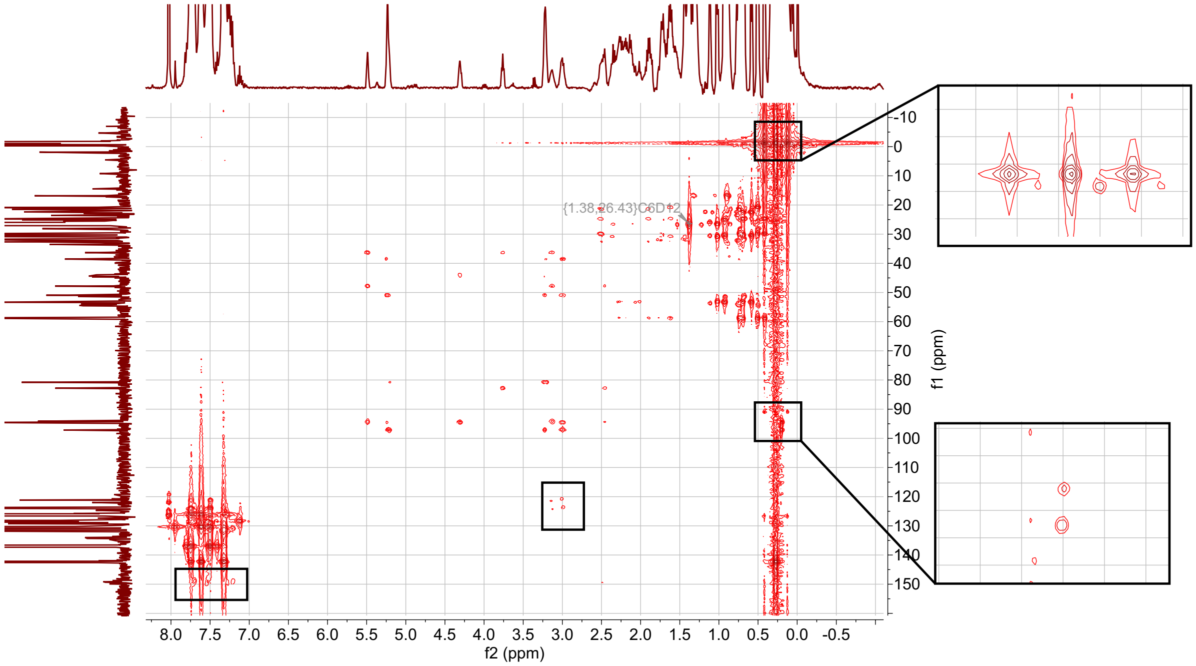

Figure S139. $^{1}H–^{13}C\{^{1}H\}$ HMBC NMR spectrum (cyclohexane-d_{12}, 23 °C) of **Co1-k** with 1,3,5-tris(trifluoromethyl)benzene and excess arene. Inset: assignment of quaternary carbons and C(sp^{2})-TMS correlation.

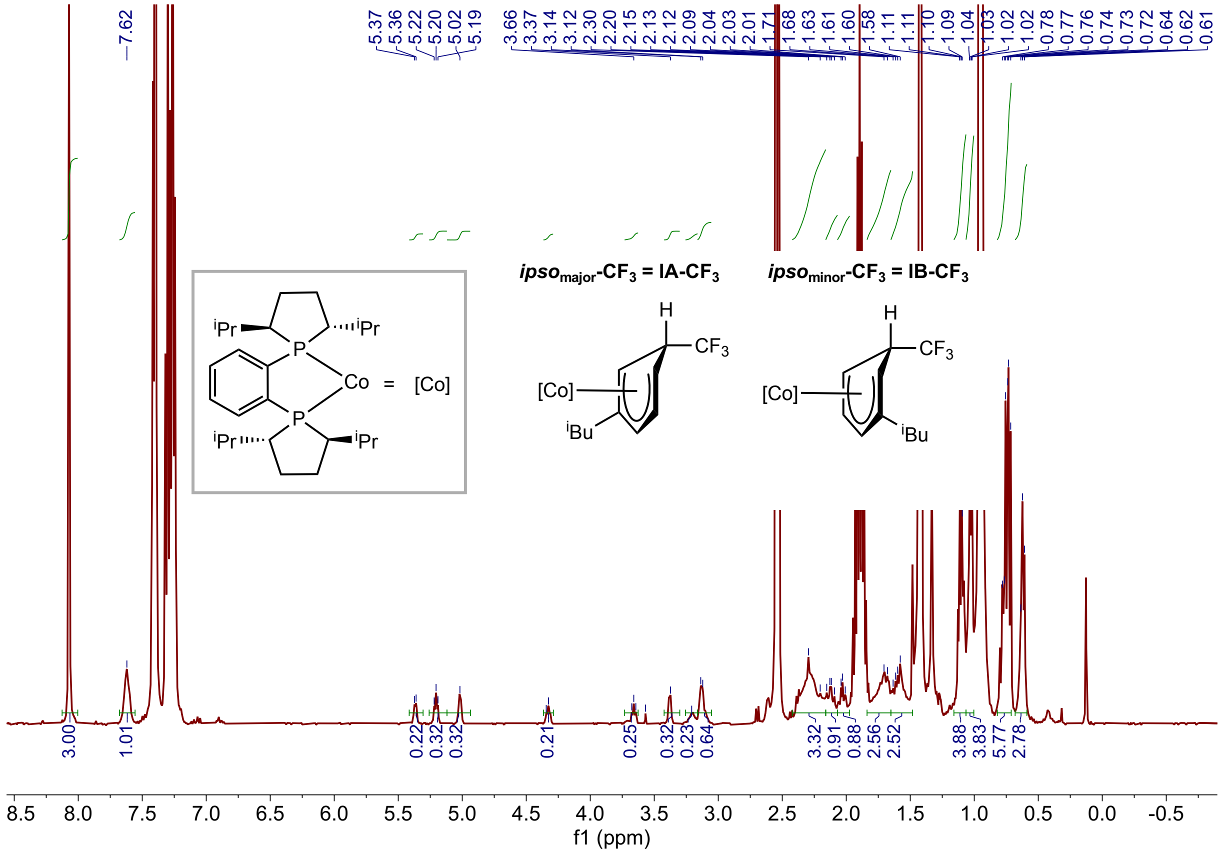

Figure S140. ^{1}H NMR spectrum (400 MHz, cyclohexane-d_{12}, 23 °C) of **Co1-I** with 1,3,5-tris(trifluoromethyl)benzene internal standard (δ 8.03 ppm) and excess arene.

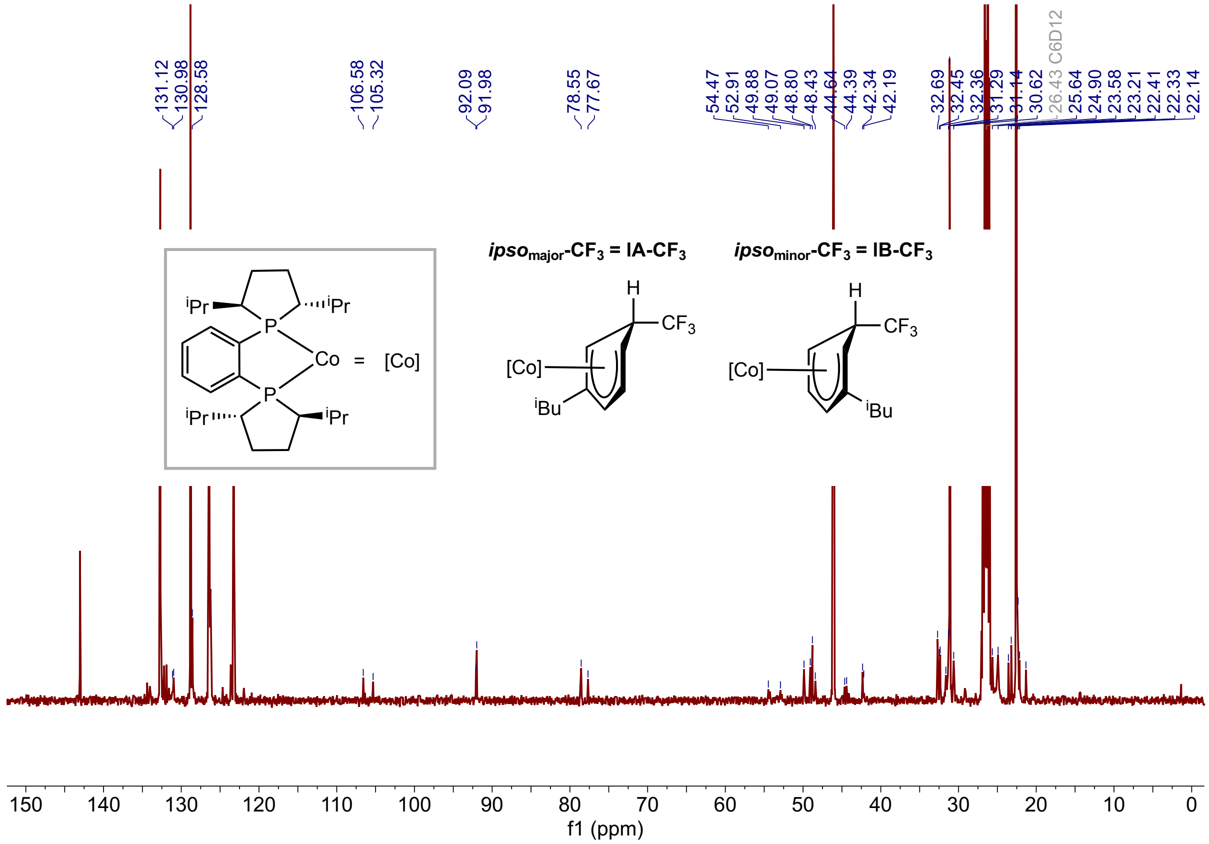

Figure S141. $^{13}C\{^{1}H\}$ NMR spectrum (101 MHz, cyclohexane-d_{12}, 23 °C) of **Co1-I** with 1,3,5-tris(trifluoromethyl)benzene and excess arene.

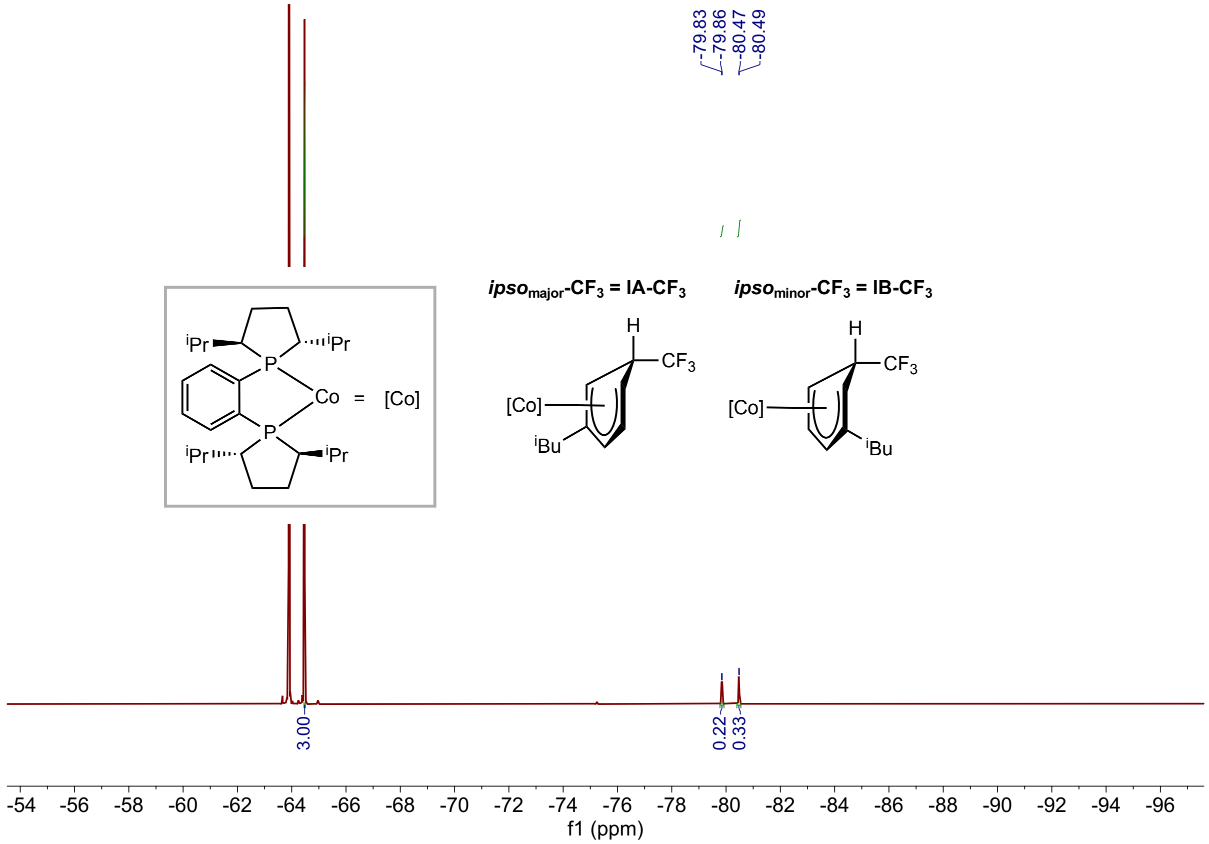

Figure S142. ^{19}F NMR spectrum (376 MHz, cyclohexane-d_{12}, 23 °C) of **Co1-I** with 1,3,5-tris(trifluoromethyl)benzene internal standard (δ -64.48 ppm) and excess arene (δ -63.89 ppm).

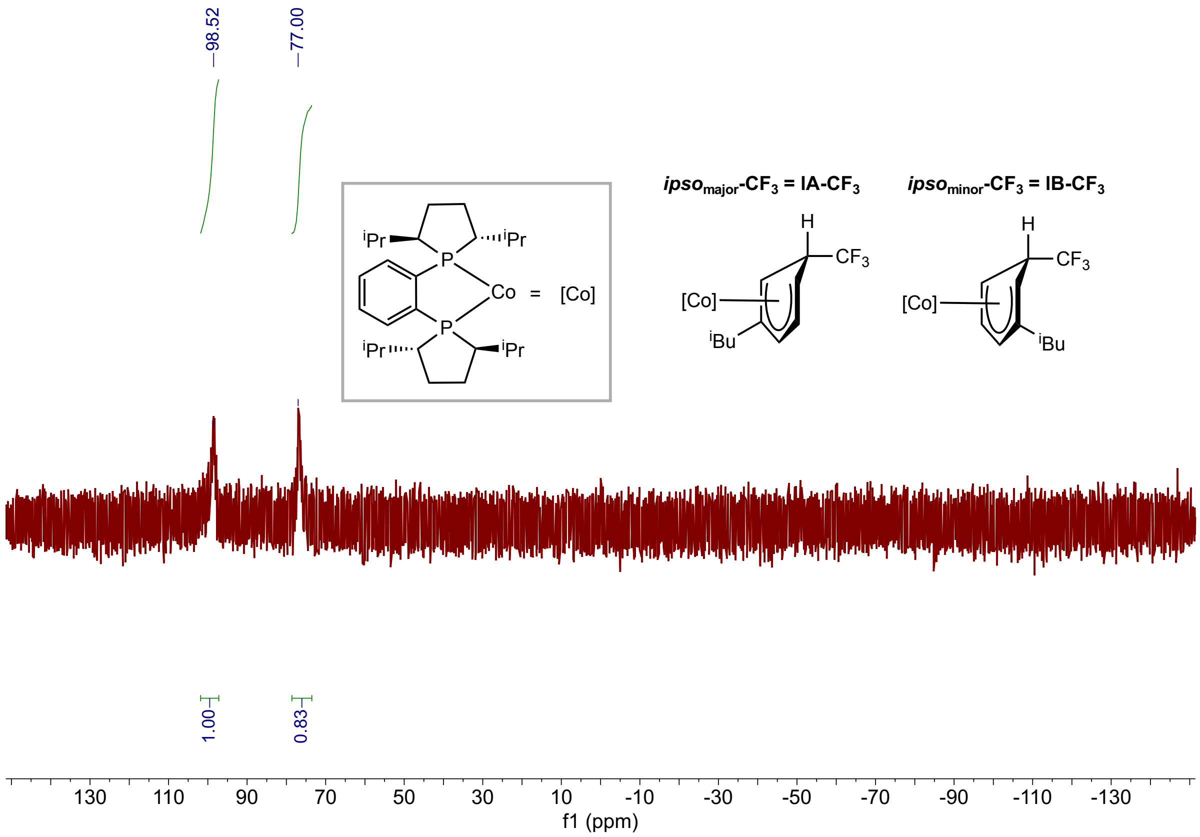

Figure S143. $^{31}P\{^1H\}$ NMR spectrum (162 MHz, cyclohexane-d_{12}, 23 °C) of **Co1-I**.

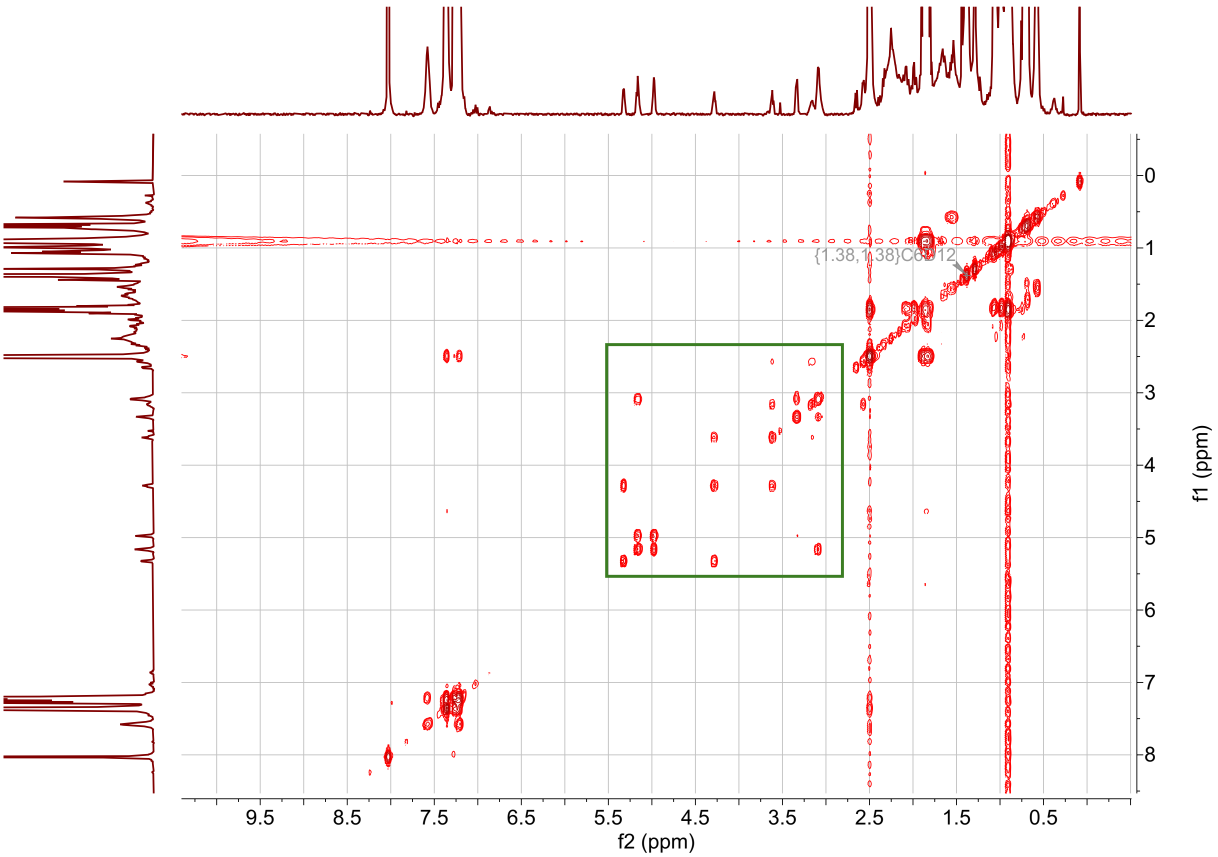

Figure S144. ^{1}H–^{1}H COSY NMR spectrum (cyclohexane-d_{12}, 23 °C) of **Co1-I** with 1,3,5-tris(trifluoromethyl)benzene and excess arene. Inset: ^{1}H–^{1}H correlation between η^{5}-cyclohexadienyl signals.

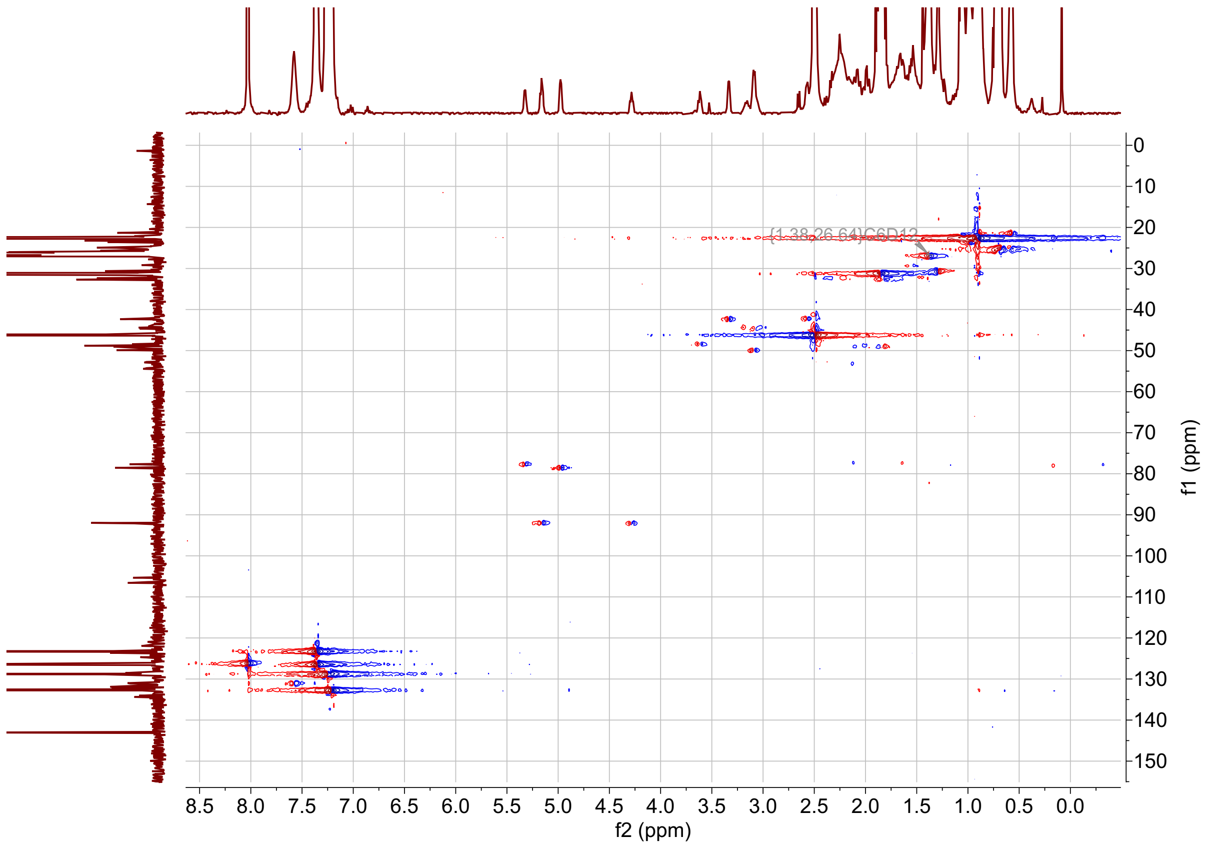

Figure S145. ^{1}H–$^{13}C\{^{1}H\}$ HSQC NMR spectrum (cyclohexane-d_{12}, 23 °C) of **Co1-I** with 1,3,5-tris(trifluoromethyl)benzene and excess arene.

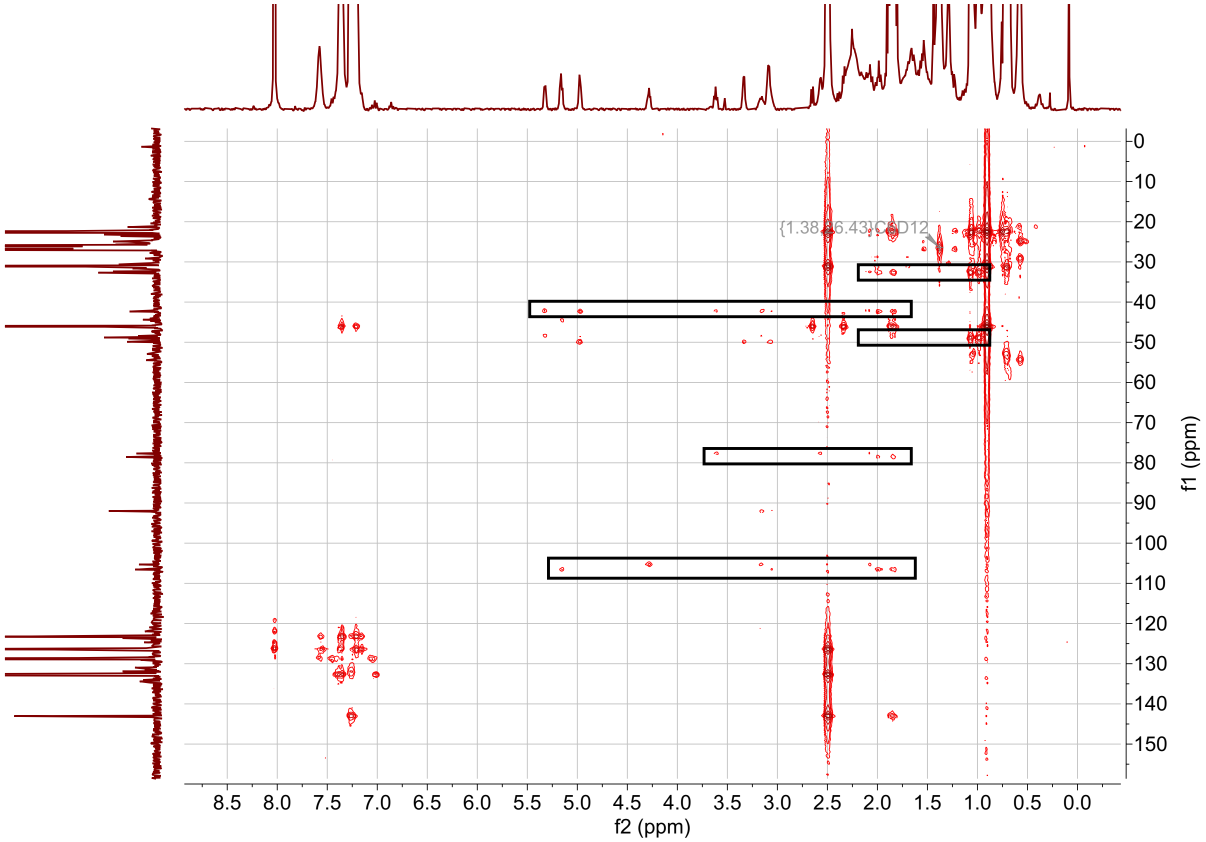

Figure S146. ^{1}H–$^{13}C\{^{1}H\}$ HMBC NMR spectrum (cyclohexane-d_{12}, 23 °C) of **Co1-I** with 1,3,5-tris(trifluoromethyl)benzene and excess arene. Inset: assignment of quaternary carbons and C(sp^{2})-^{i}Bu correlation.

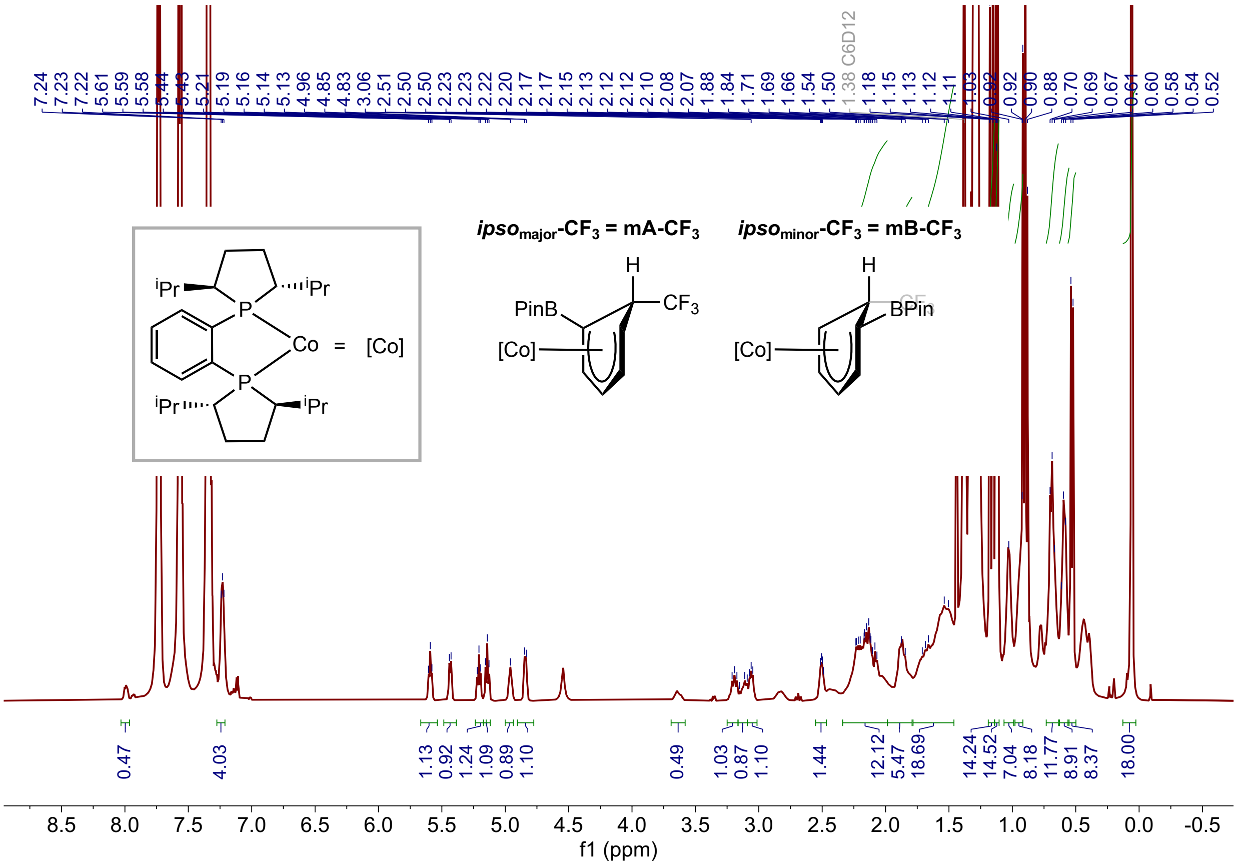

Figure S147. ^{1}H NMR spectrum (400 MHz, cyclohexane-d_{12}, 23 °C) of **Co1-m** with HMDSO internal standard (δ 0.10 ppm) and excess arene.

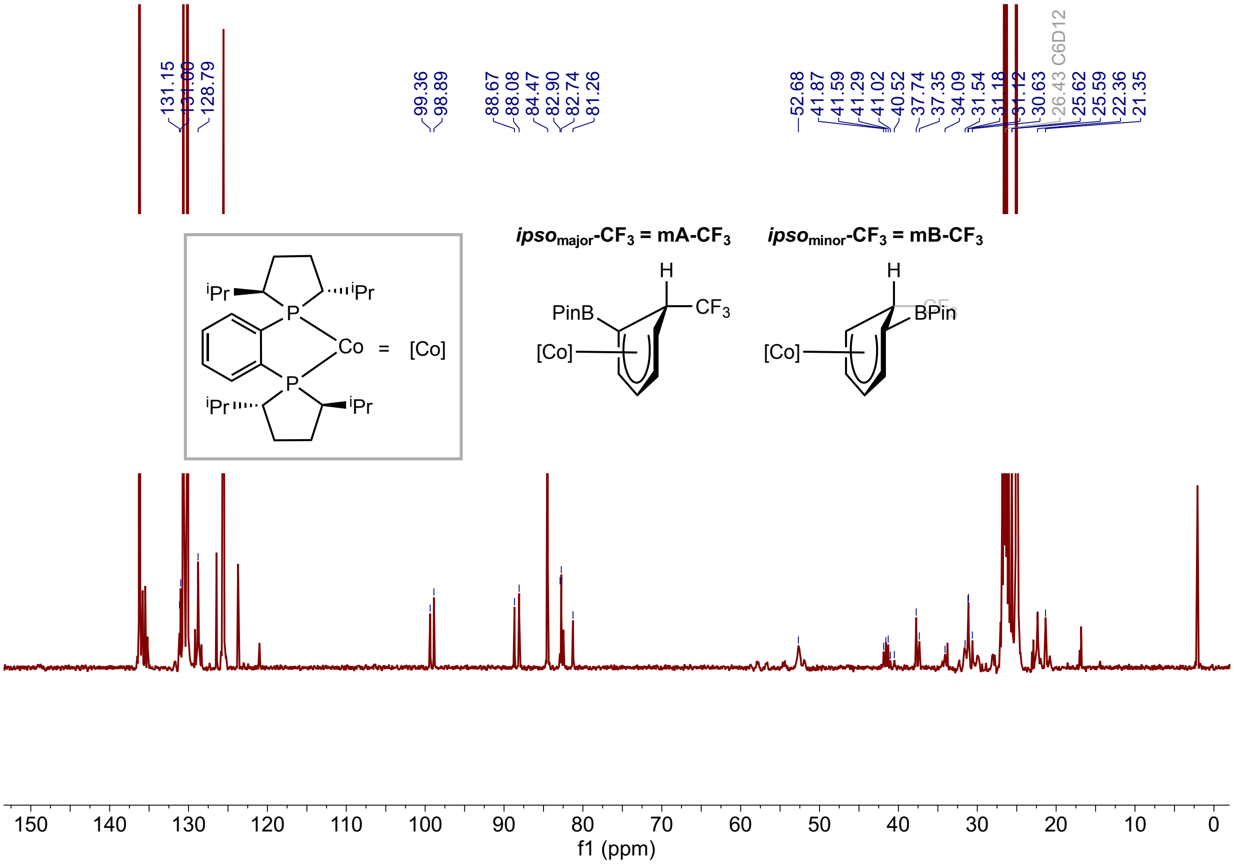

Figure S148. $^{13}C\{^{1}H\}$ NMR spectrum (101 MHz, cyclohexane-d_{12}, 23 °C) of **Co1-m** with HMDSO internal standard and excess arene.

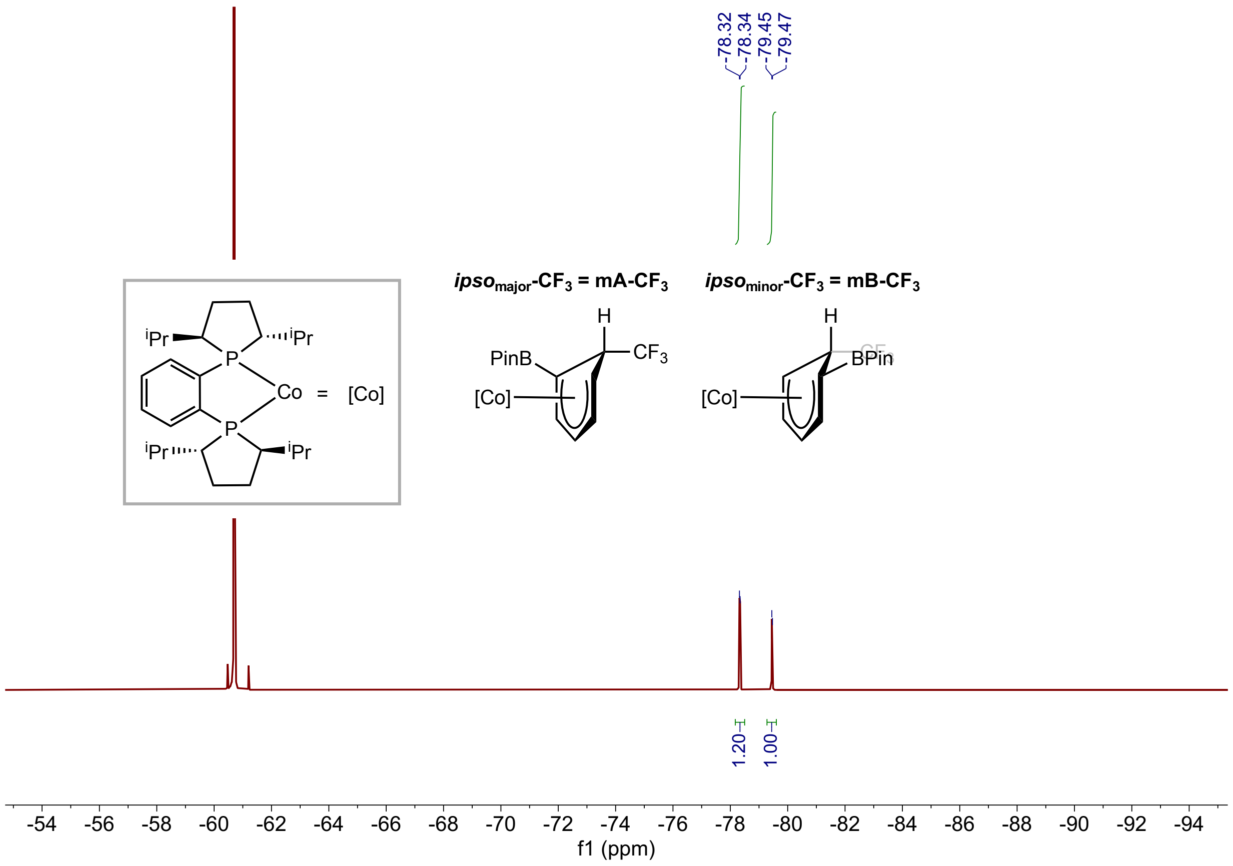

Figure S149. ^{19}F NMR spectrum (376 MHz, cyclohexane-d_{12}, 23 °C) of **Co1-m** in excess arene (δ -60.71 ppm).

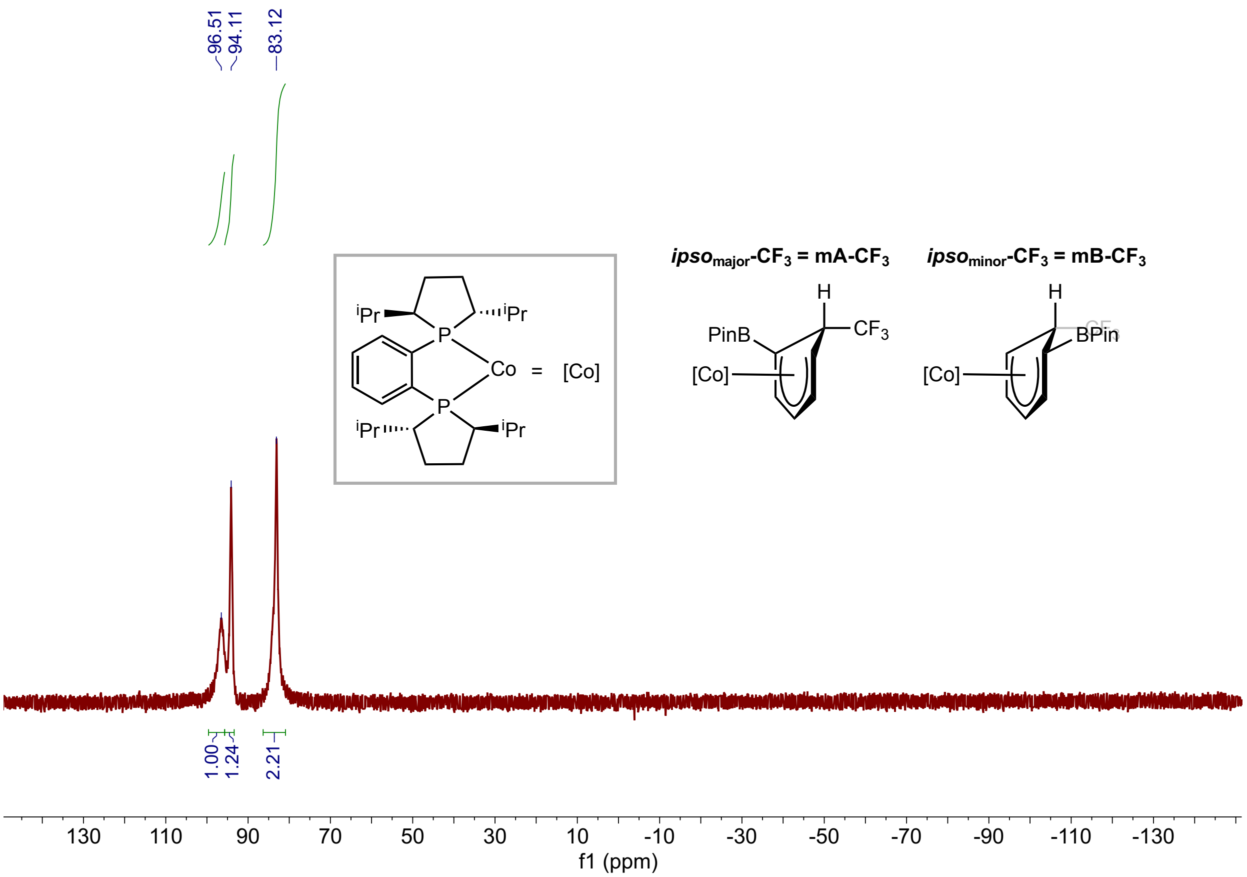

Figure S150. $^{31}P\{^{1}H\}$ NMR spectrum (162 MHz, cyclohexane-d_{12}, 23 °C) of **Co1-m**.

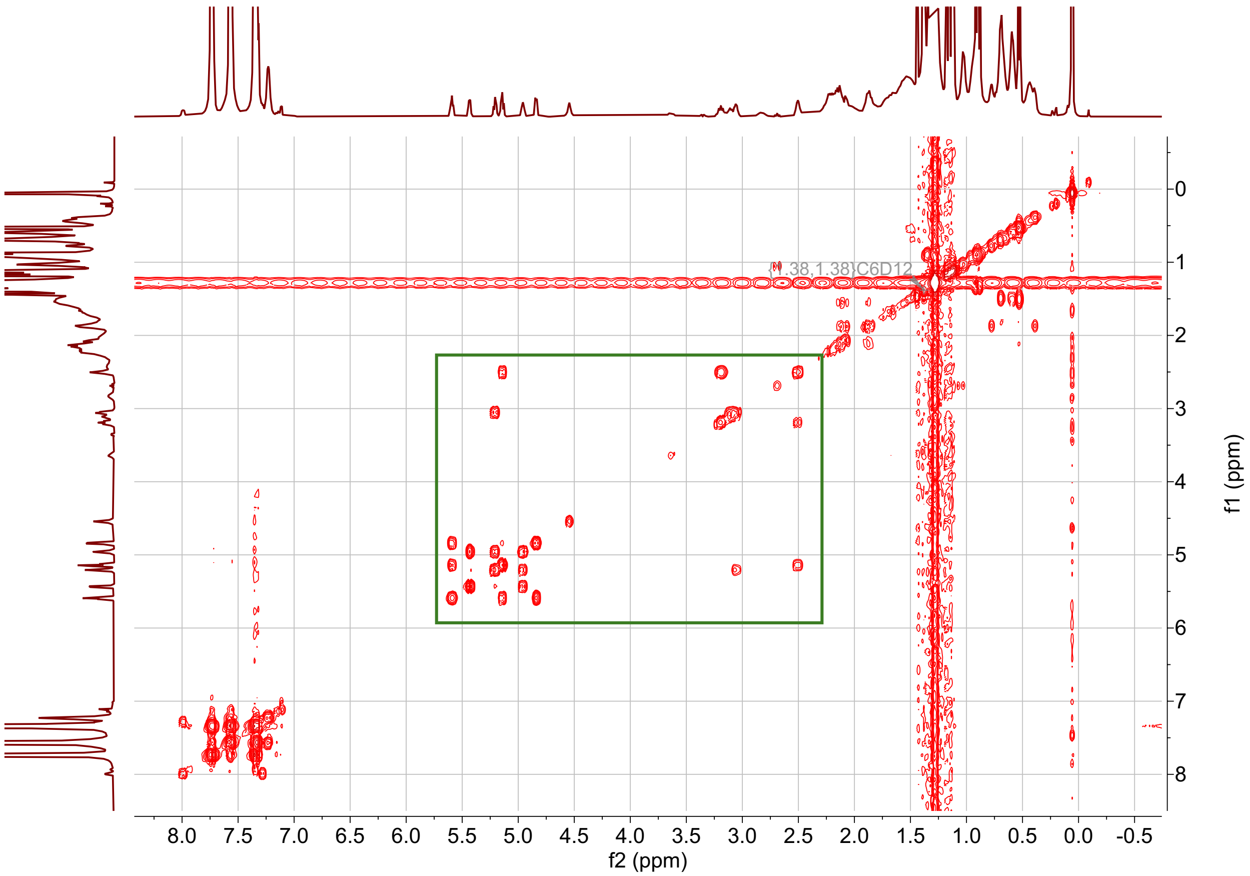

Figure S151. ^{1}H–^{1}H COSY NMR spectrum (cyclohexane-d_{12}, 23 °C) of **Co1-m** with HMDSO internal standard and excess arene. Inset: ^{1}H–^{1}H correlation between η^{5}-cyclohexadienyl signals.

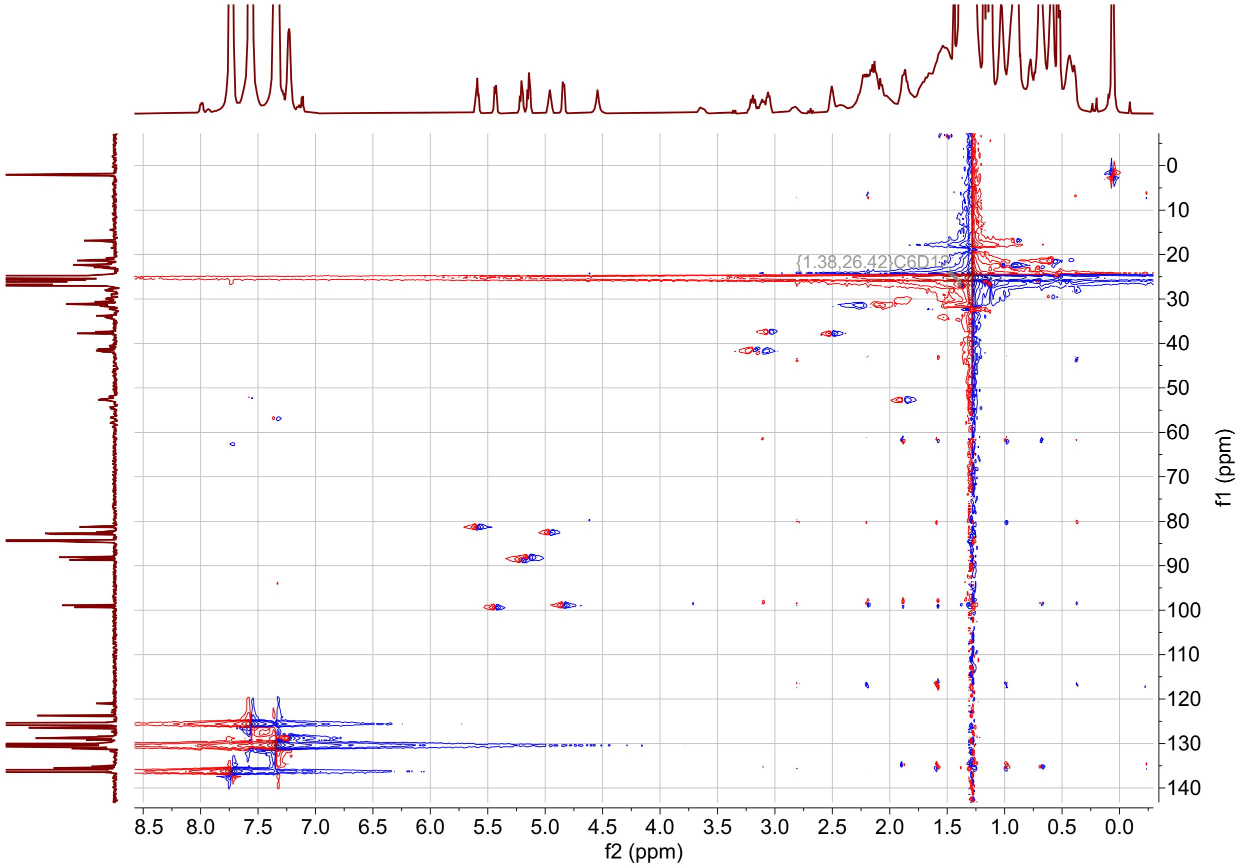

Figure S152. $^{1}H–^{13}C\{^{1}H\}$ HSQC NMR spectrum (cyclohexane-d_{12}, 23 °C) of **Co1-m** with HMDSO internal standard and excess arene.

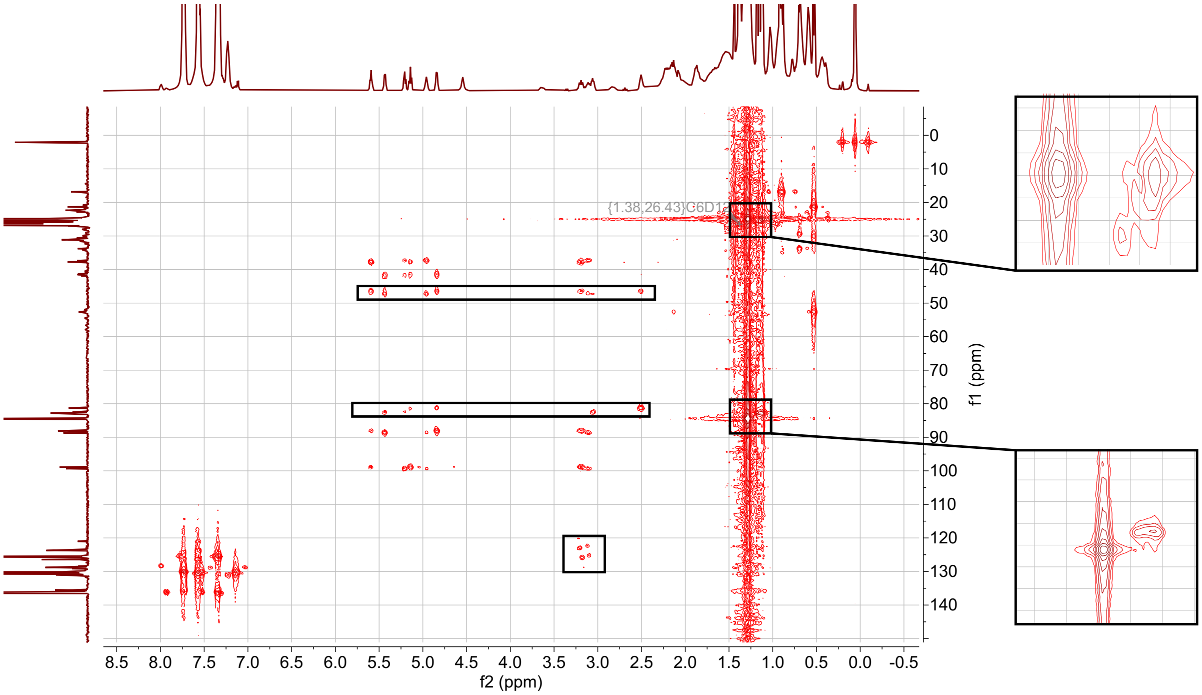

Figure S153. ^{1}H–$^{13}C\{^{1}H\}$ HMBC NMR spectrum (cyclohexane-d_{12}, 23 °C) of **Co1-m** with HMDSO internal standard (δ 0.10 ppm) and excess arene. Inset: assignment of quaternary carbons and C(sp^{2})–BPin correlation.

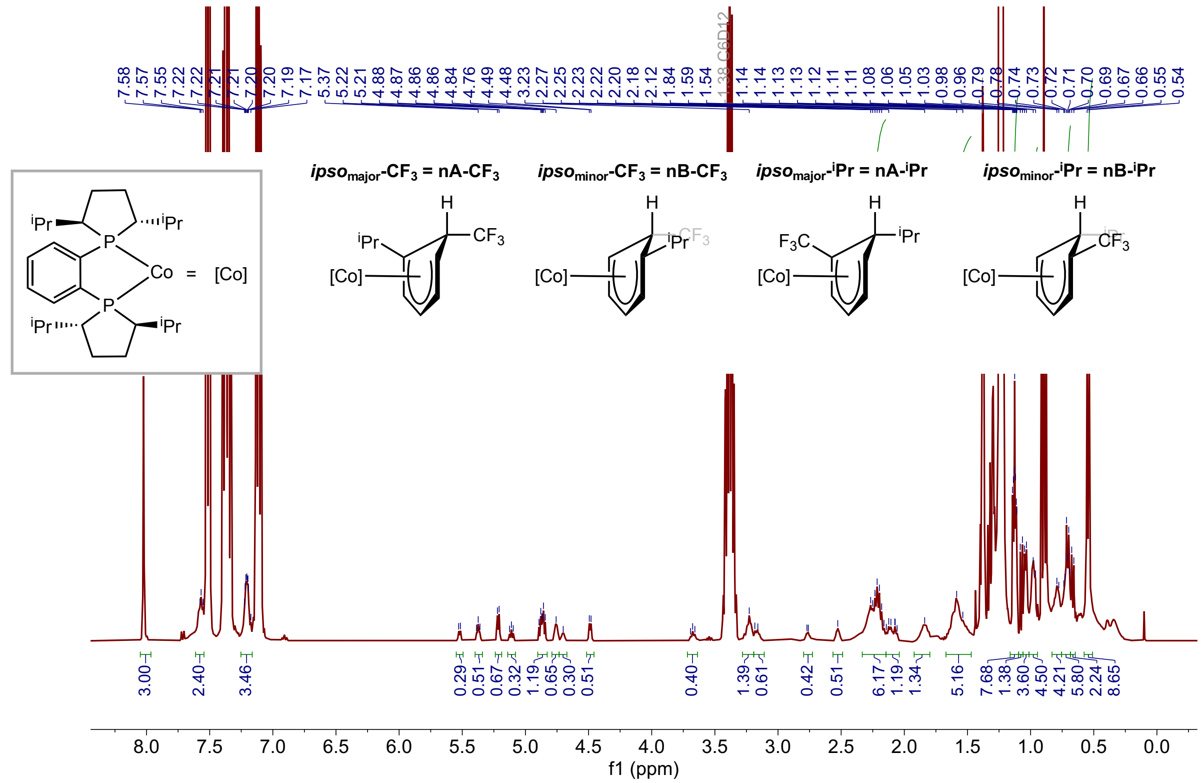

Figure S154. ^{1}H NMR spectrum (400 MHz, cyclohexane-d_{12}, 23 °C) of **Co1-n** with 1,3,5-tris(trifluoromethyl)benzene internal standard (δ 8.03 ppm) and excess arene.

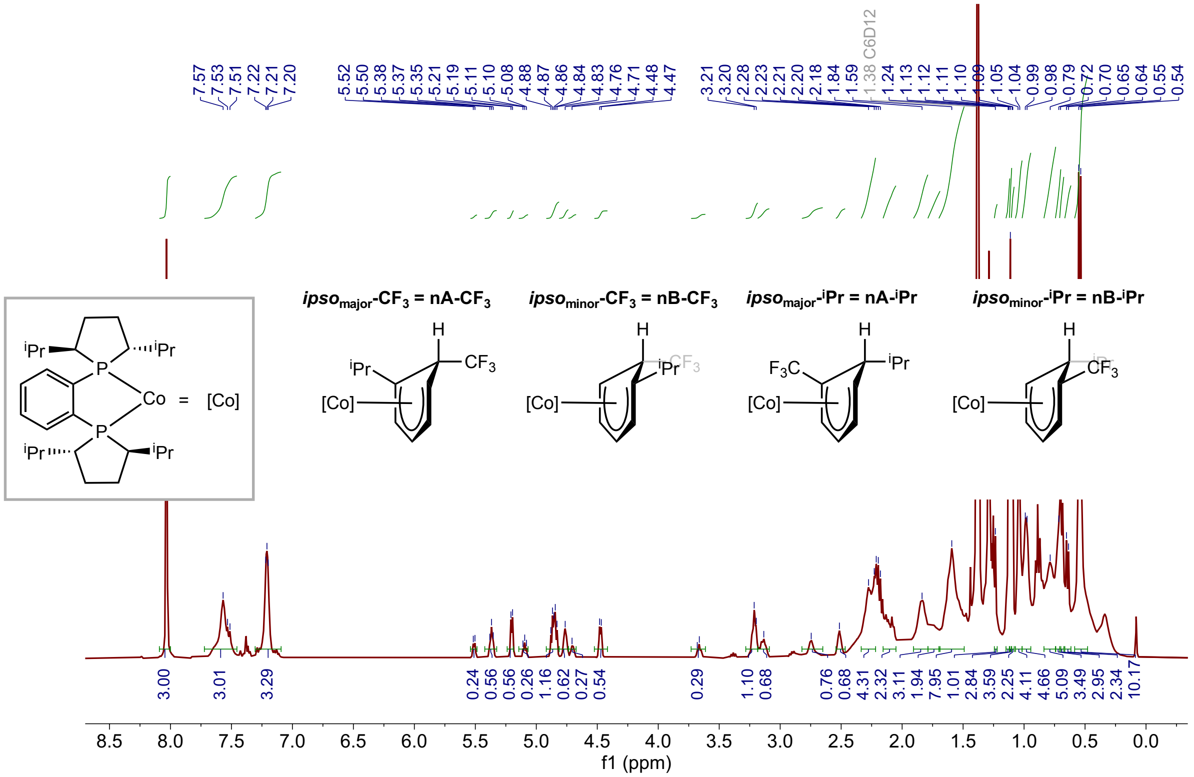

Figure S155. ^{1}H NMR spectrum (400 MHz, cyclohexane-d_{12}, 23 °C) of isolated **Co1-n** with 1,3,5-tris(trifluoromethyl)benzene internal standard (δ 8.03 ppm).

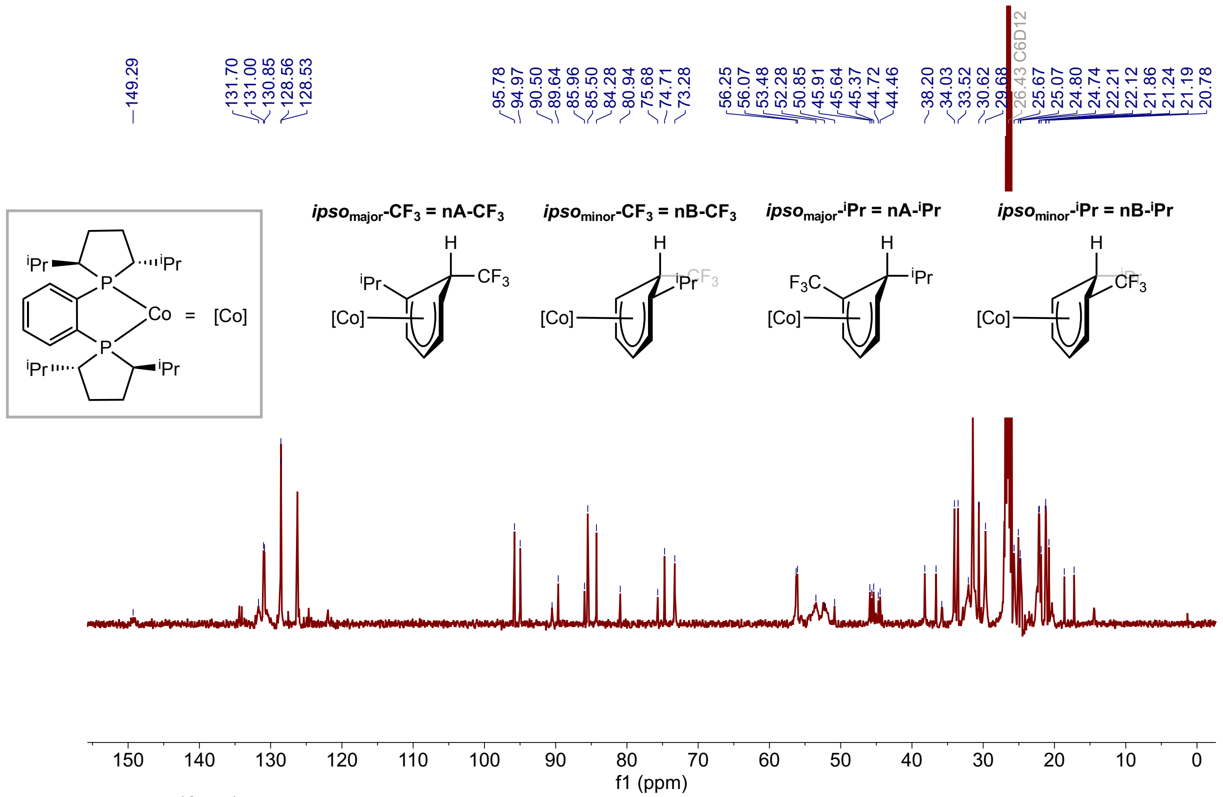

Figure S156. $^{13}C\{^{1}H\}$ NMR spectrum (101 MHz, cyclohexane-d_{12}, 23 °C) of isolated **Co1-n** with 1,3,5-tris(trifluoromethyl)benzene.

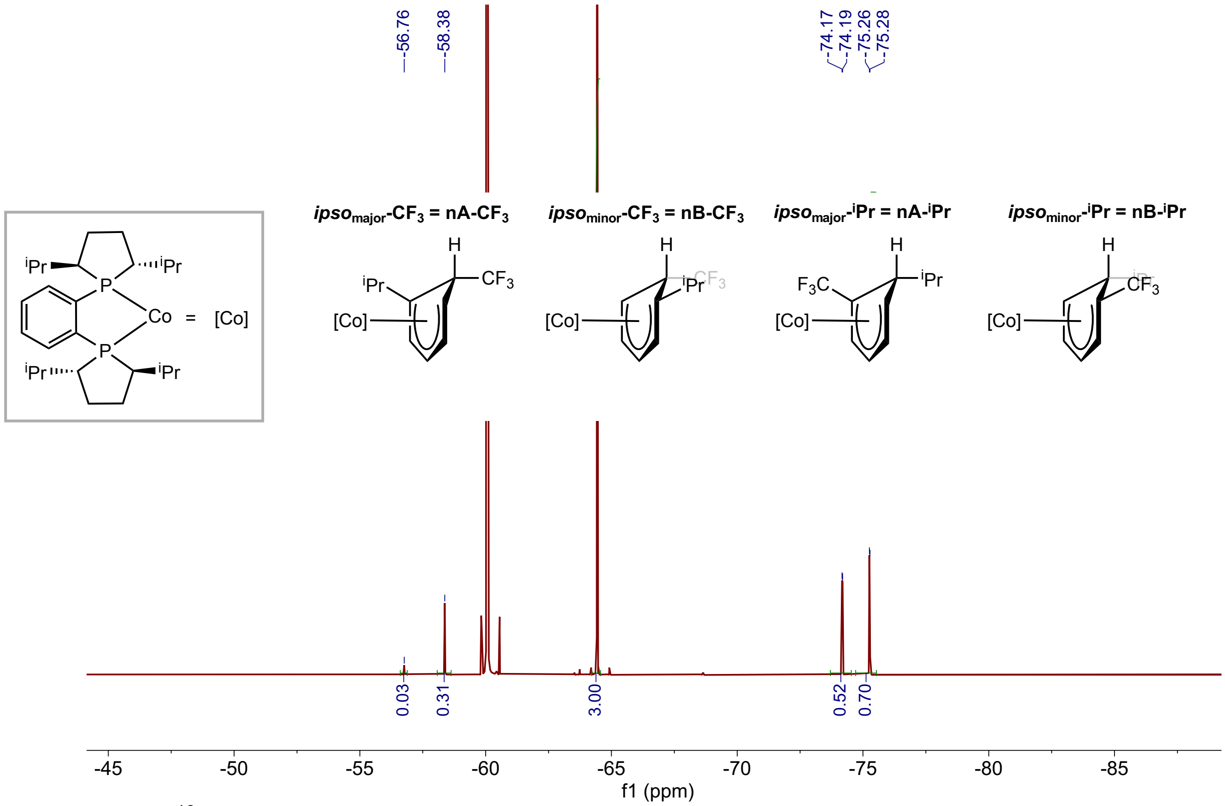

Figure S157. ^{19}F NMR spectrum (376 MHz, cyclohexane-d_{12}, 23 °C) of **Co1-n** with 1,3,5-tris(trifluoromethyl)benzene internal standard (δ -64.43 ppm) and excess arene (δ -60.06 ppm).

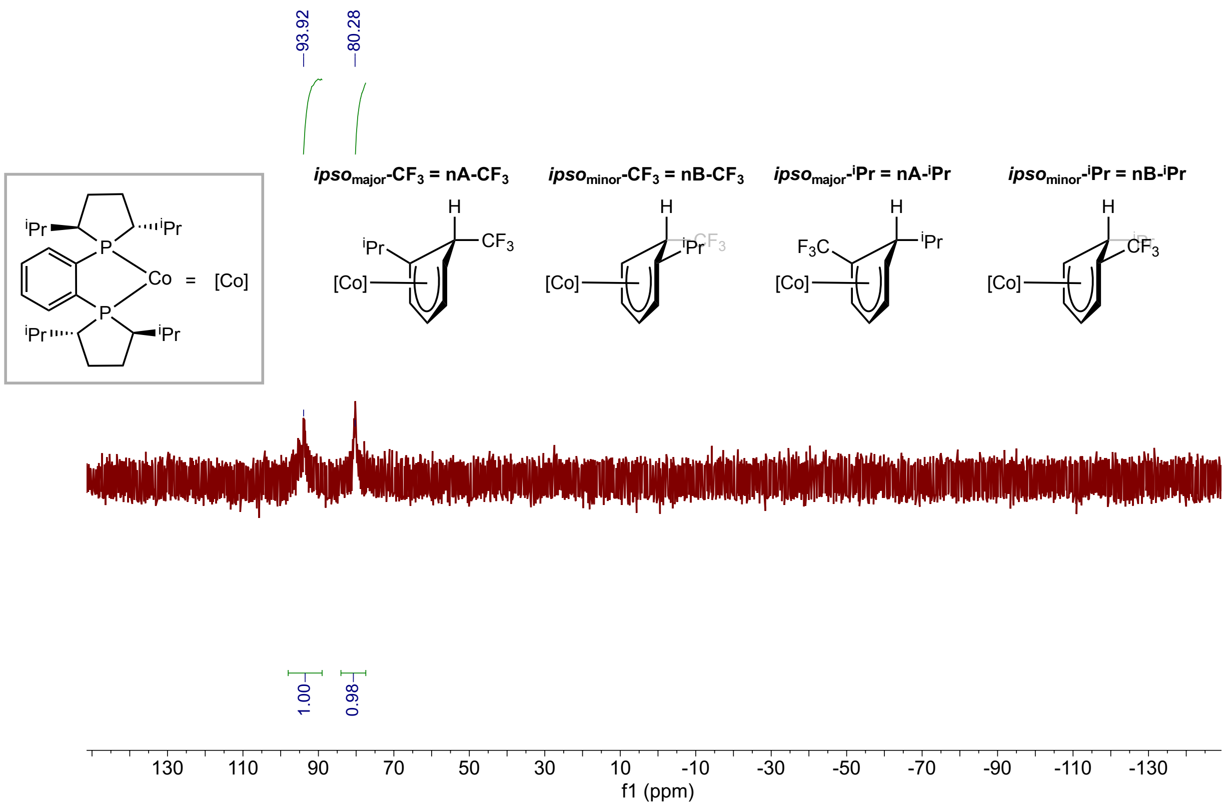

Figure S158. $^{31}P\{^{1}H\}$ NMR spectrum (162 MHz, cyclohexane-d_{12}, 23 °C) of isolated **Co1-n**.

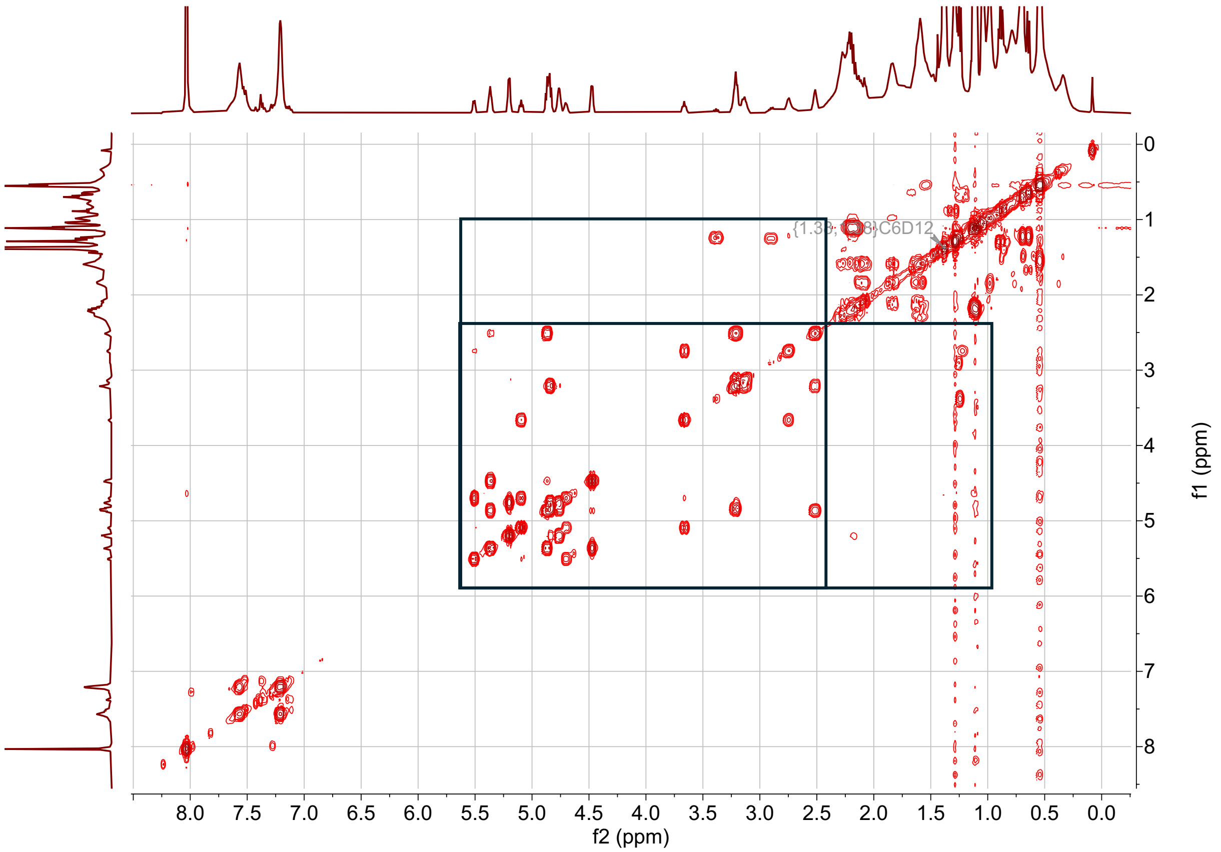

Figure S159. $^{1}H–^{1}H$ COSY NMR spectrum (cyclohexane-d_{12}, 23 °C) of isolated **Co1-n** with 1,3,5-tris(trifluoromethyl)benzene. Inset: $^{1}H–^{1}H$ correlation between η^{5}-cyclohexadienyl signals.

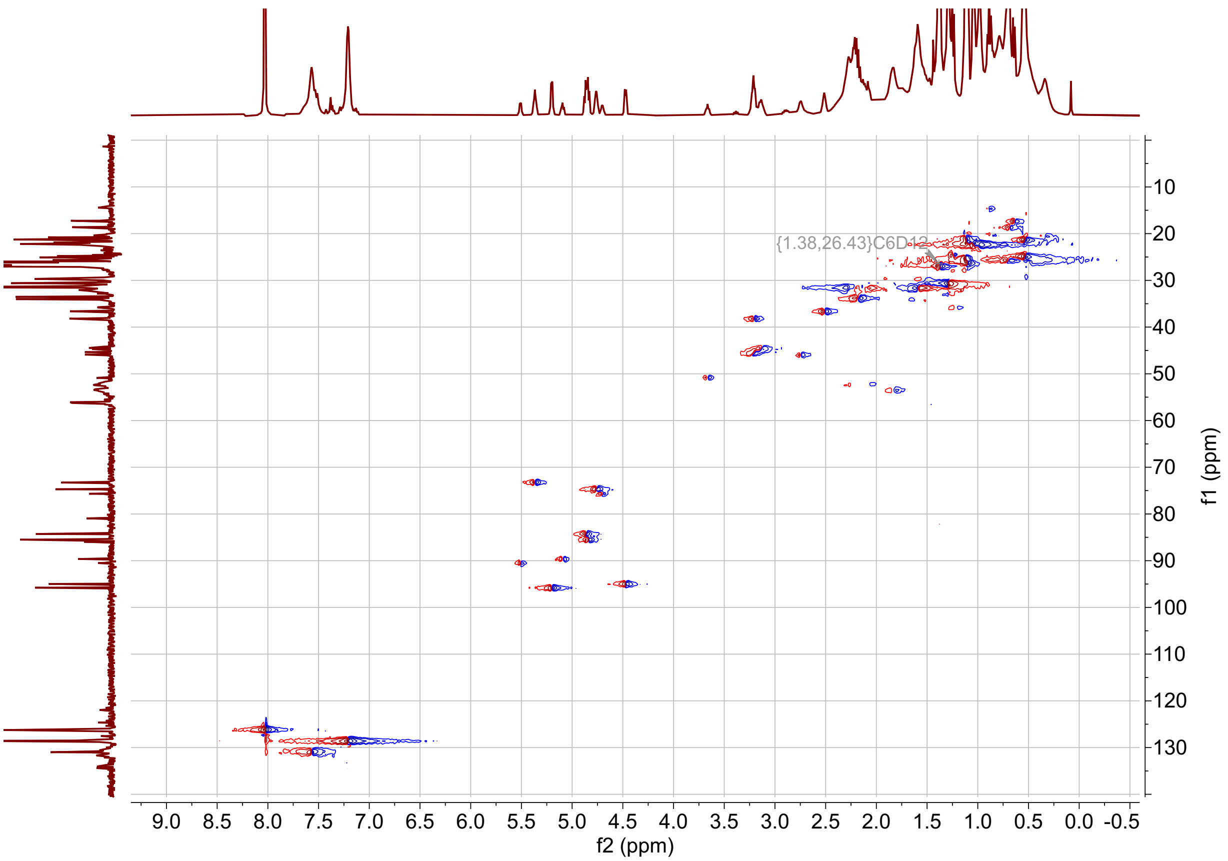

Figure S160. ^{1}H–$^{13}C\{^{1}H\}$ HSQC NMR spectrum (cyclohexane-d_{12}, 23 °C) of isolated **Co1-n** with 1,3,5-tris(trifluoromethyl)benzene.

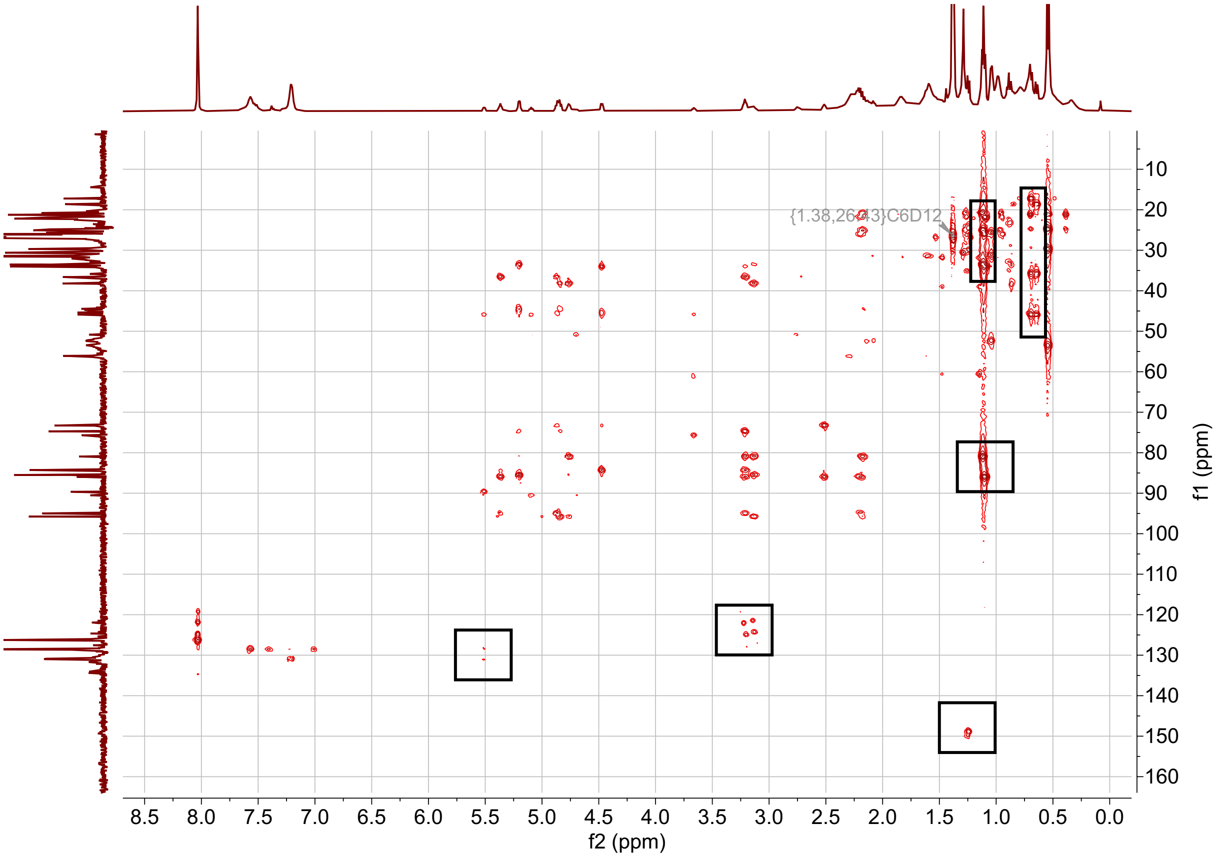

Figure S161. $^1H–^{13}C\{^1H\}$ HMBC NMR spectrum (cyclohexane-d_{12}, 23 °C) of isolated **Co1-n** with 1,3,5-tris(trifluoromethyl)benzene. Inset: assignment of quaternary carbons, $C(sp^2)–^iPr$, and $C(sp^3)–^iPr$ correlation.

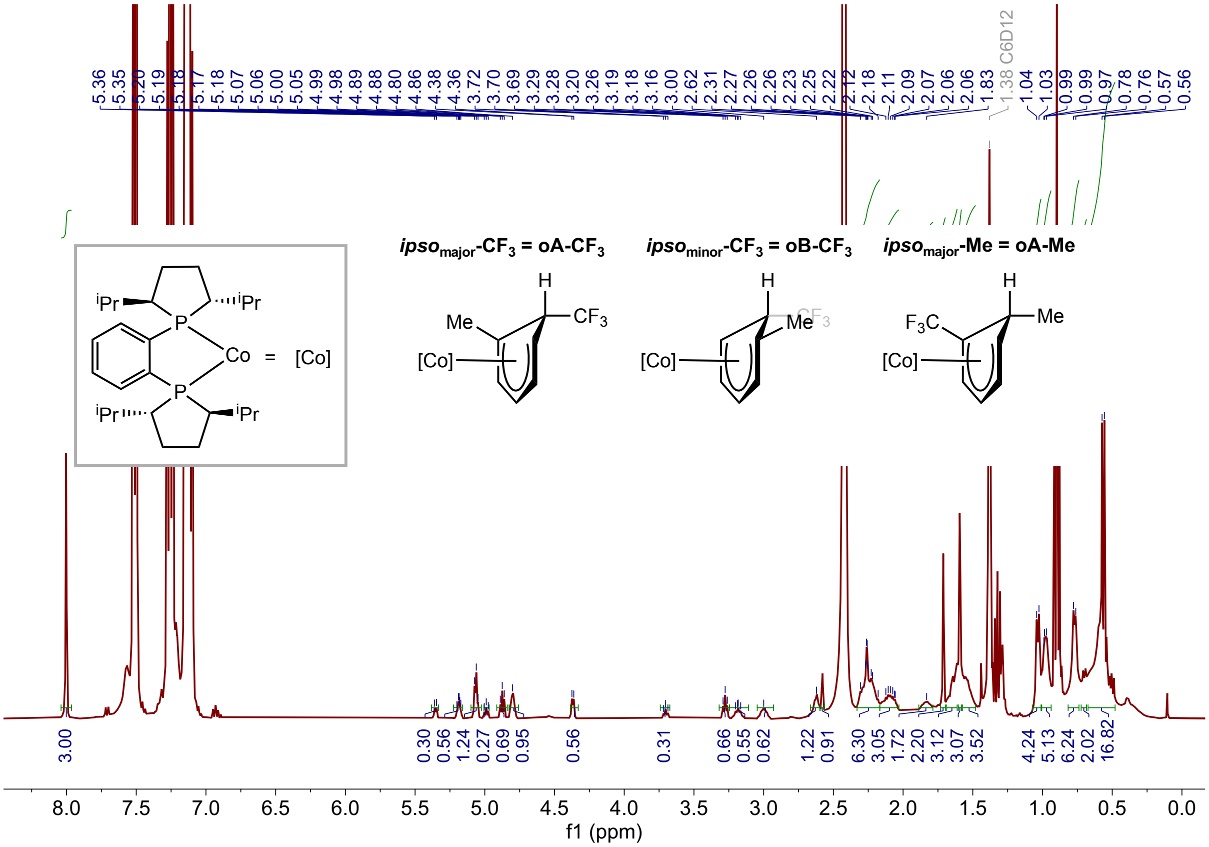

Figure S162. ^{1}H NMR spectrum (400 MHz, cyclohexane-d_{12}, 23 °C) of **Co1-o** with 1,3,5-tris(trifluoromethyl)benzene internal standard (δ 8.03 ppm) and excess arene.

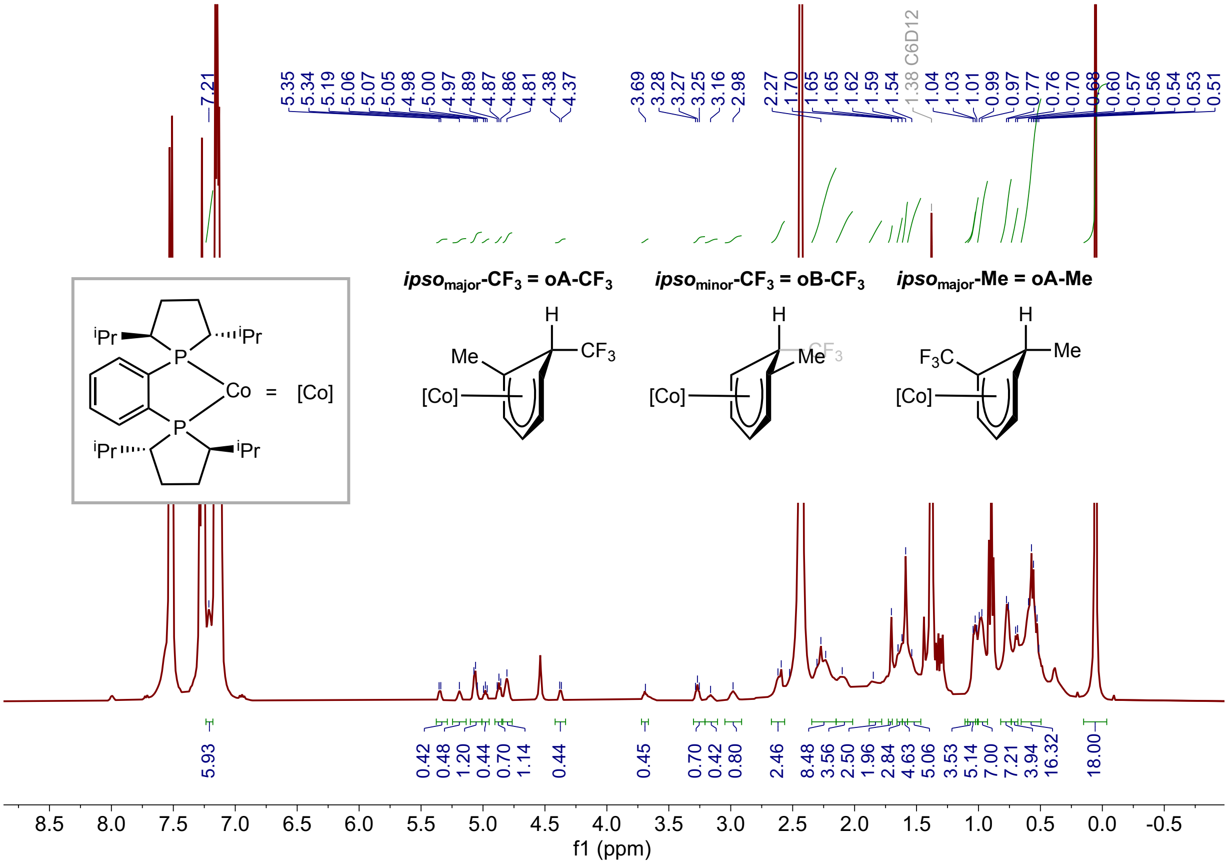

Figure S163. ^{1}H NMR spectrum (400 MHz, cyclohexane-d_{12}, 23 °C) of **Co1-o** with HMDSO internal standard (δ 0.10 ppm) and excess arene.

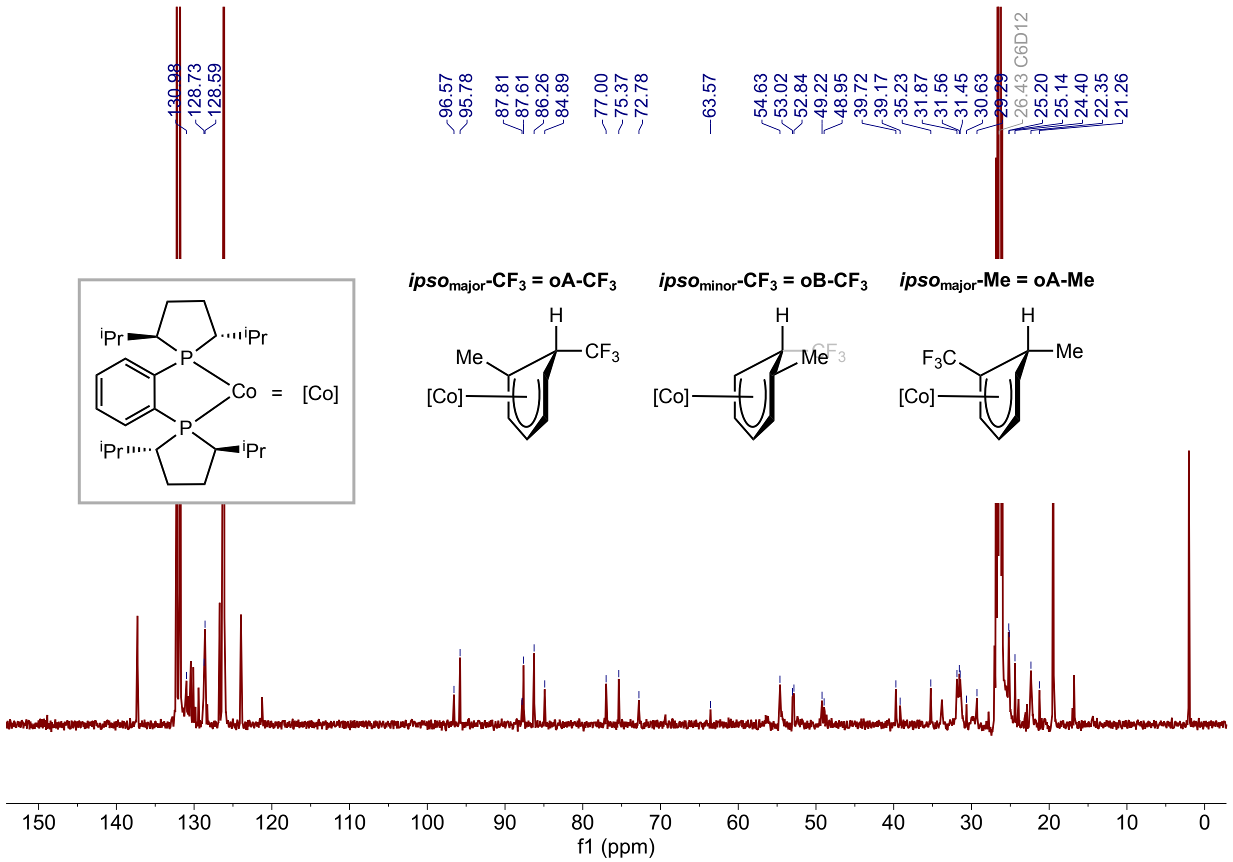

Figure S164. $^{13}C\{^1H\}$ NMR spectrum (101 MHz, cyclohexane-d_{12}, 23 °C) of **Co1-o** with HMDSO internal standard and excess arene.

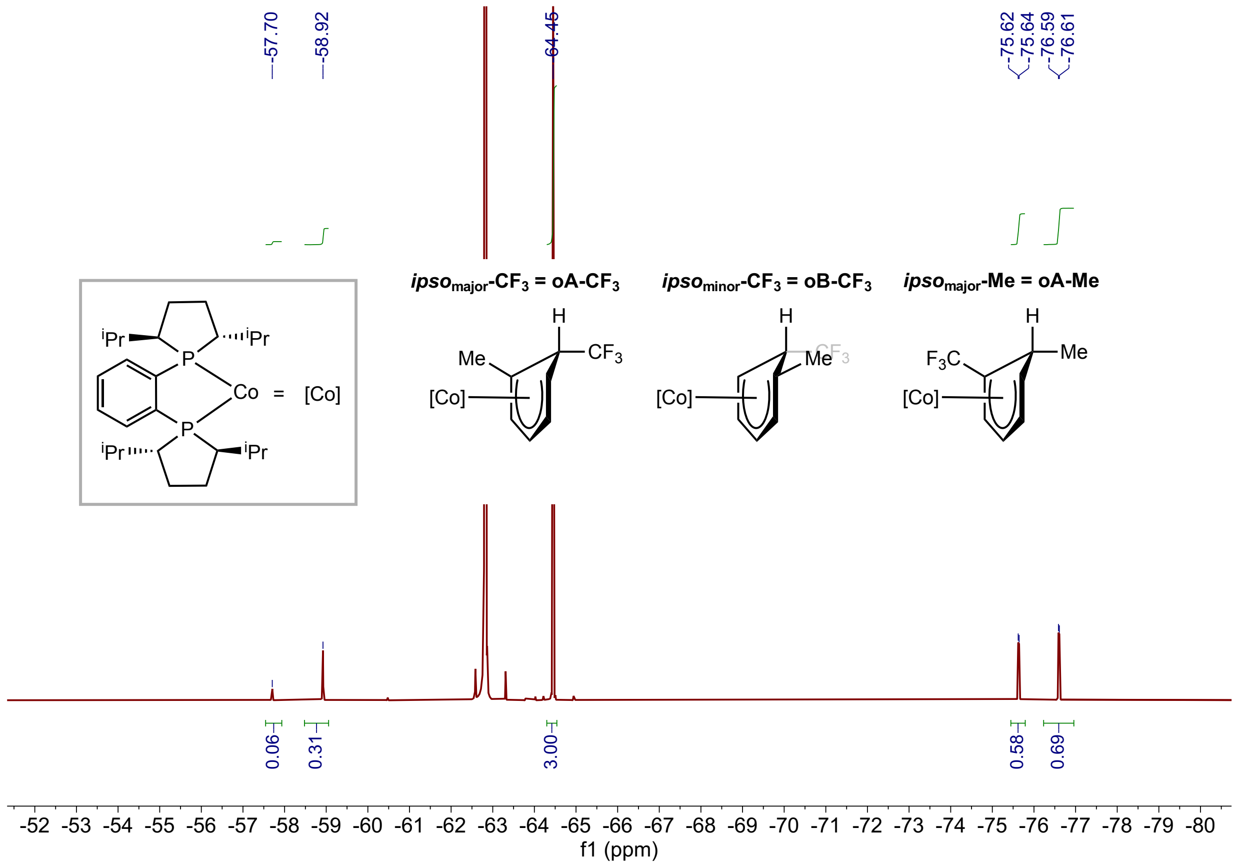

Figure S165. ^{19}F NMR spectrum (376 MHz, cyclohexane-d_{12}, 23 °C) of **Co1-o** with 1,3,5-tris(trifluoromethyl)benzene internal standard (δ -64.45 ppm) and excess arene (δ -62.82 ppm).

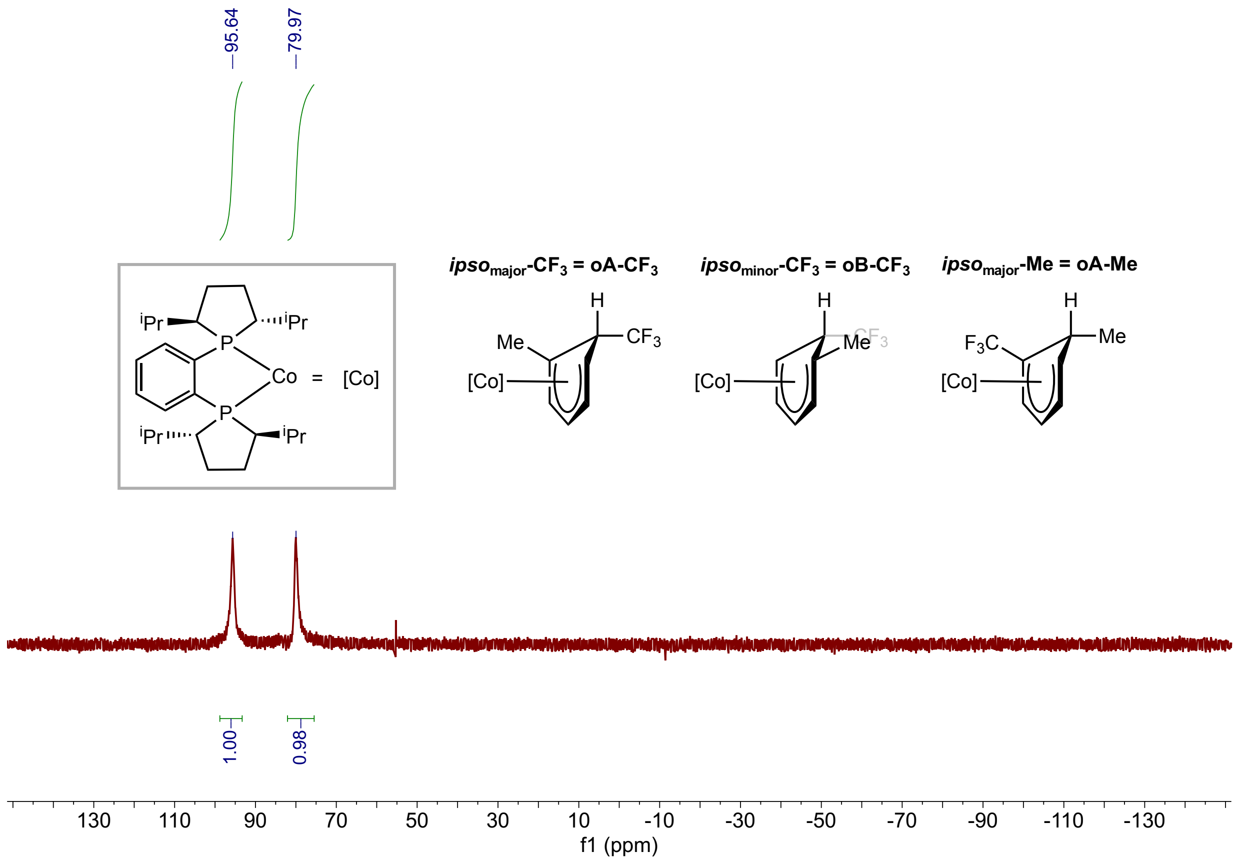

Figure S166. $^{31}P\{^1H\}$ NMR spectrum (162 MHz, cyclohexane-d_{12}, 23 °C) of **Co1-o**.

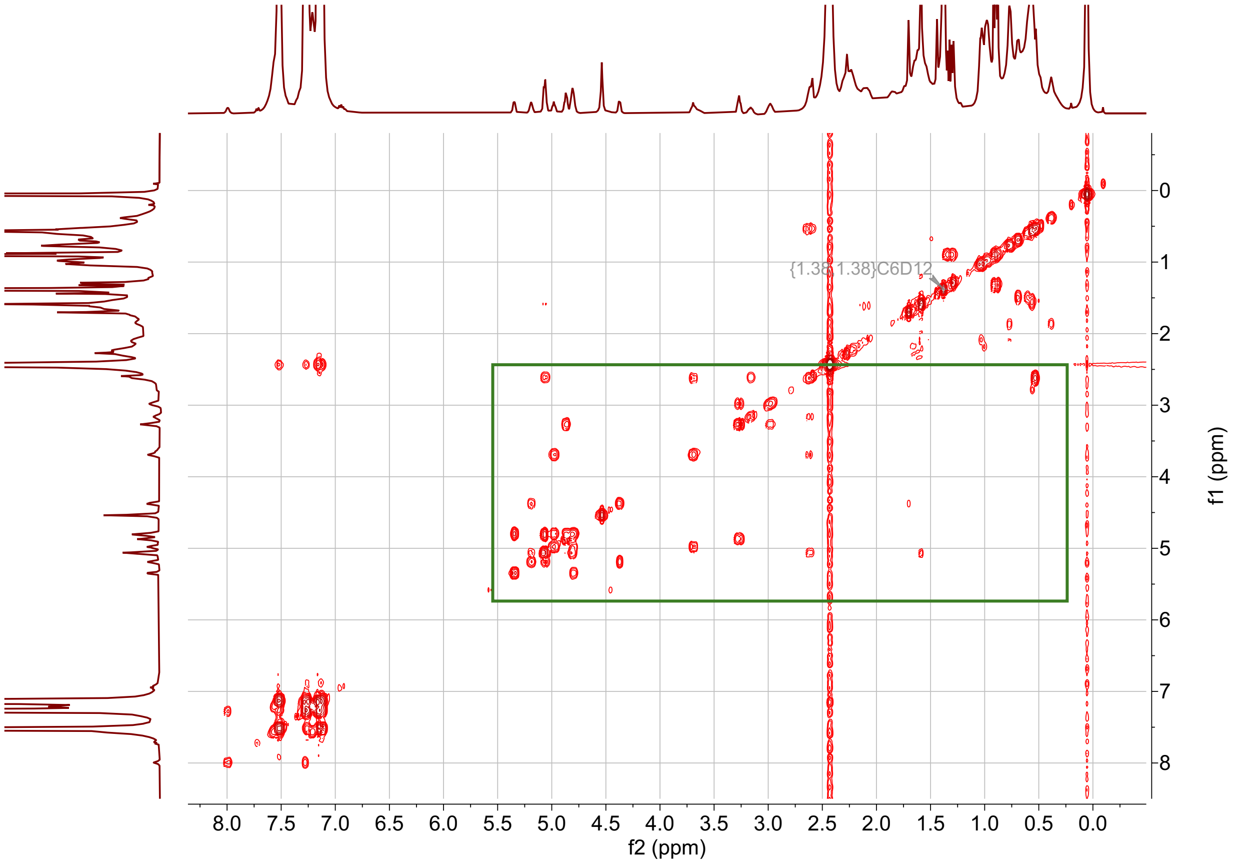

Figure S167. ^{1}H–^{1}H COSY NMR spectrum (cyclohexane-d_{12}, 23 °C) of **Co1-o** with HMDSO and excess arene. Inset: ^{1}H–^{1}H correlation between η^5-cyclohexadienyl signals.

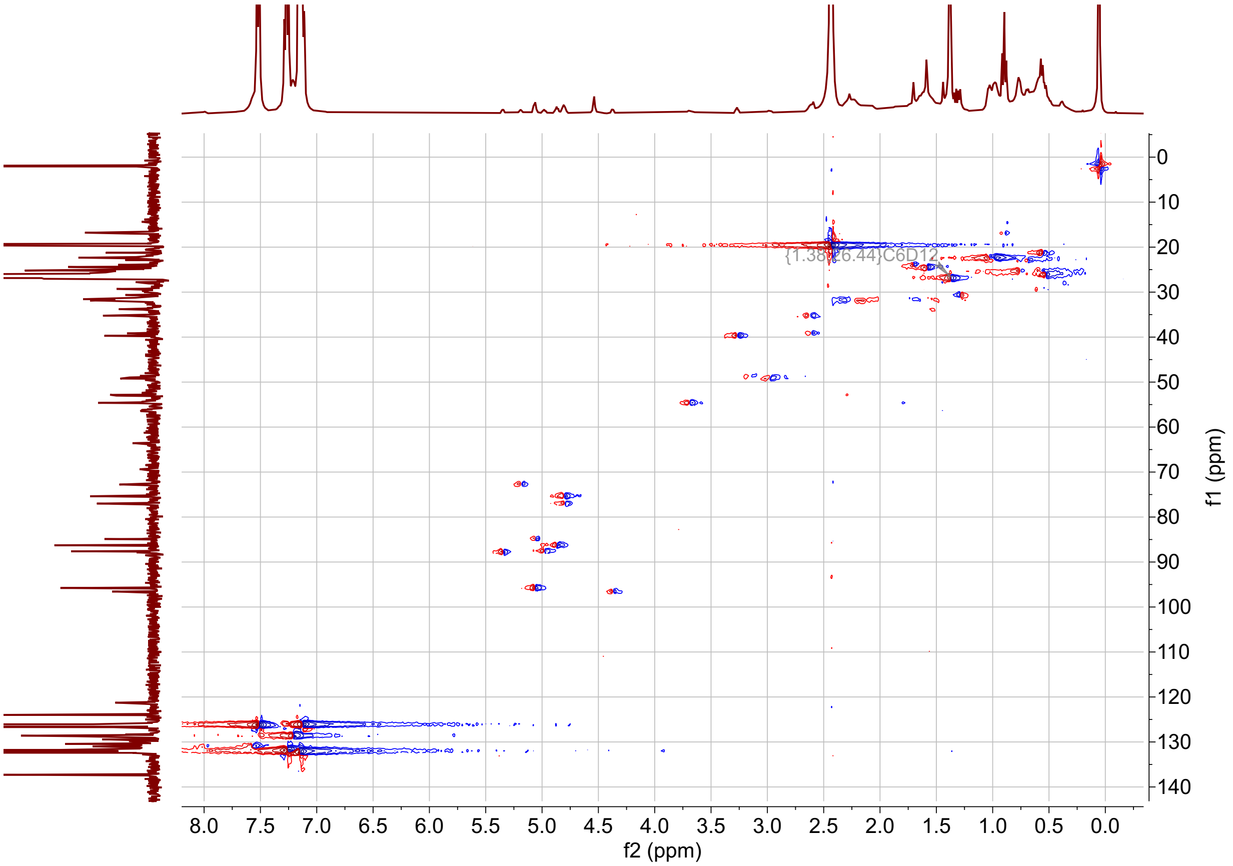

Figure S168. ^{1}H–$^{13}C\{^{1}H\}$ HSQC NMR spectrum (cyclohexane-d_{12}, 23 °C) of **Co1-o** with HMDSO and excess arene.

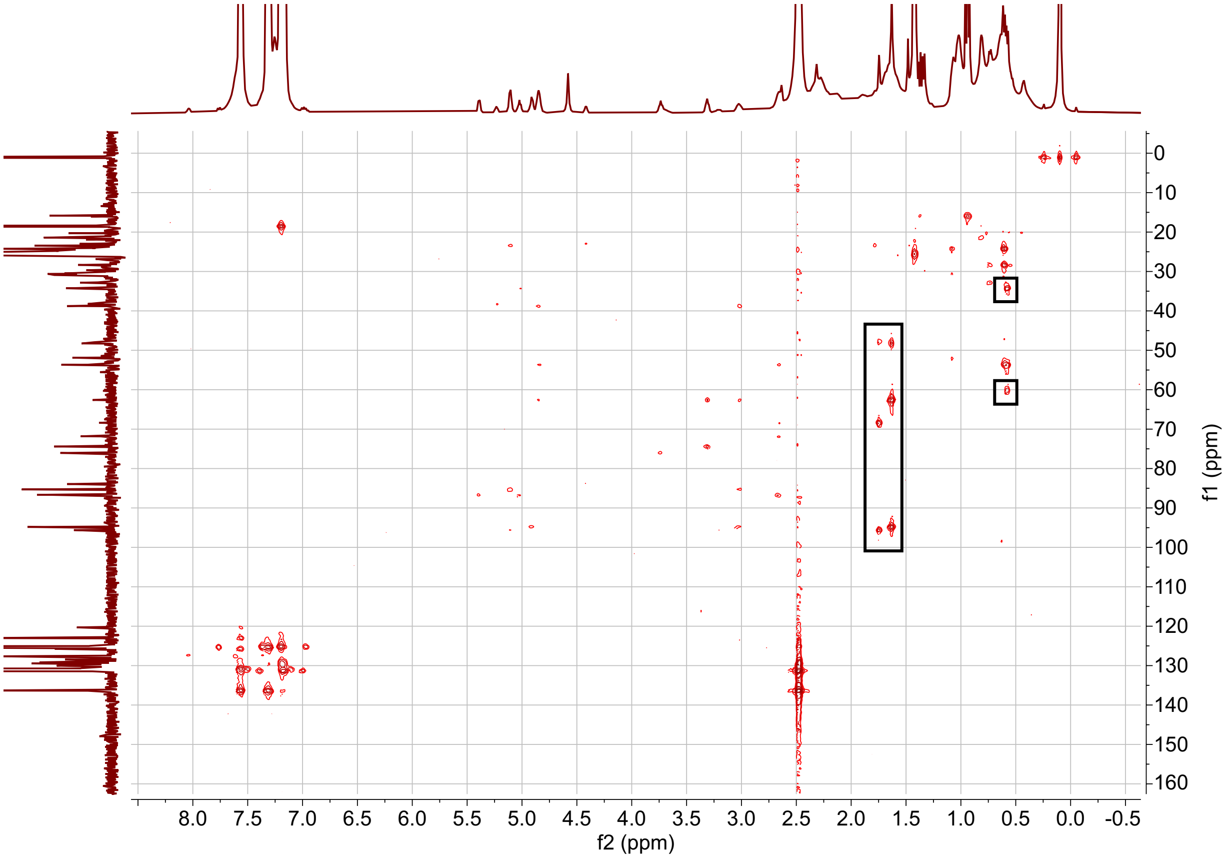

Figure S169. $^{1}H–^{13}C\{^{1}H\}$ HMBC NMR spectrum (cyclohexane-d_{12}, 23 °C) of **Co1-o** with HMDSO and excess arene. Inset: assignment of quaternary carbons, C(sp^2)–Me, and C(sp^3)–Me correlation.

VI. Preparation and Characterization of Cyclohexadienes

General Procedure A. A 20 mL scintillation vial was charged with **Co1-a, Co1-h,** or **Co1-l** (1 equiv.) in a benzene-d_6 solution. In a separate vial equipped with a Teflon-coated magnetic stir-bar, benzoic acid (2.5 equiv.) was weighed out. The cobalt solution was added to the vial containing benzoic acid and stirred at room temperature for 15 minutes to 1 hour, where a color change from orange-red to deep red was observed. The solution was added to a J. Young NMR tube to confirm full conversion by ^{1}H and ^{19}F NMR spectroscopy. The volatiles were then vacuum-transferred to another J. Young NMR tube with 50 μL of a 0.25 M 1,3,5-tris(trifluoromethyl)benzene cyclohexane-d_{12} solution as an internal standard. Benzoic acid-*d* was

purchased from Sigma Aldrich and used without further purification. The purity of benzoic acid-*d* was determined by relative integration in the ^{1}H NMR spectrum, which showed 93% deuterium incorporation.

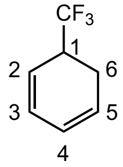

Preparation of 1. This compound was prepared using General Procedure A using 0.0256 mmol of **Co1-a.** The colorless liquid obtained following vacuum transfer was obtained in 55% yield (0.0141 mmol) of **1** relative to 1,3,5-tris(trifluoromethyl)benzene and obtained as the sole-isomer assigned using ^{1}H, $^{13}C\{^{1}H\}$, and ^{19}F NMR spectroscopy. ^{1}H NMR (400 MHz, benzene-d_6) δ 5.73 (dddt, J = 8.8, 4.4, 2.7, 0.9 Hz, 1H, $C^{3}H$), 5.66 – 5.55 (m, 1H, $C^{4}H$), 5.47 (dd, J = 9.9, 3.2 Hz, 1H, $C^{2}H$), 5.38 (dt, J = 9.3, 4.4 Hz, 1H, $C^{5}H$), 2.68 (dpt, J = 15.1, 9.2, 3.1 Hz, 1H, $C^{1}H$), 2.12 (ddt, J = 17.9, 14.8, 3.1 Hz, 1H, $C^{6}H$), 1.93 (dddd, J = 17.6, 9.1, 5.5, 1.4 Hz, 1H, $C^{6}H$). $^{13}C\{^{1}H\}$ NMR (101 MHz, benzene-d_6) δ 126.97 (s, C^{3}), 124.38 (s, C^{5}), 123.43 (s, C^{4}), 118.59 (q, J = 3.3 Hz, C^{2}), 38.39 (q, J = 27.0 Hz, C^{1}), 21.46 (q, J = 3.1 Hz, C^{6}). ^{19}F NMR (376 MHz, benzene-d_6) δ -72.57 (d, J = 9.3 Hz, 3F).

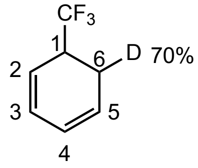

Preparation of 1D. This compound was prepared using General Procedure A using 0.0353 mmol of **Co1-a**, and 0.0706 mmol of benzoic acid-*d* (93% deuterated). The colorless liquid obtained following vacuum transfer was obtained in 59% yield (0.0206 mmol, 70% deuterium incorporation exclusively at C^{6}) relative to 1,3,5-tris(trifluoromethyl)benzene and obtained as the sole-isomer was quantified by ^{1}H, ^{2}H, and ^{19}F NMR spectroscopy. Separately, following general procedure A, using 0.0929 mmol of **Co1-a**, and 0.0232 mmol of benzoic acid-*d* (93% deuterated) in toluene-

d_8, a quantitative $^{13}C\{^1H\}$ NMR was collected to show $^1J_{C-D}$ and $^3J_{C-F}$ coupling. ^2H NMR (400 MHz, benzene-d_6) δ 1.89 (broad s, 1H, C^6H). $^{13}C\{^1H\}$ NMR (101 MHz, toluene-d_8) δ 21.92 (tq, $^1J_{C-D}$= 20.2 Hz; $^3J_{C-F}$ = 3.1 Hz).

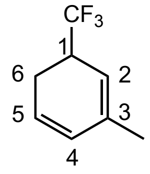

Preparation of 3-Me. This compound was prepared using General Procedure A using 0.027 mmol of **Co1-h.** The colorless liquid obtained following vacuum transfer was obtained in 50% yield (0.0135 mmol) relative to 1,3,5-tris(trifluoromethyl)benzene and was a mixture of 2-methyl-6-(trifluoromethyl)cyclohexa-1,3-diene (**3-Me**; 50% yield) and 1-methyl-5-(trifluoromethyl)cyclohexa-1,3-diene (**4-Me**; 32% yield, 0.00875 mmol) assigned using ^1H, $^{13}C\{^1H\}$, and ^{19}F NMR spectroscopy. ^1H NMR (400 MHz, toluene-d_8) δ 5.45 (m, 1H, C^5H), 5.35 (m, 1H, C^4H), 5.14 (m, 1H, C^2H), 2.62 (m, 1H, C^1H), 1.99 (m, 1H, C^6H), 1.90 – 1.84 (m, 1H, C^6H), 1.39 (app t, 3H, CH_3). ^{13}C NMR (101 MHz, toluene-d_8) δ 135.60 (s, C^3), 128.15 (s, C^5),125.18 (s, C^4), 113.99 (q, J = 2.9 Hz, C^2), 39.76 (q, J = 26.4 Hz, C^1), 22.45 (q, J = 2.8 Hz, C^6), 21.46 (s, CH_3). CF_3 resonance could not be identified due to signal broadening. ^{19}F NMR (376 MHz, toluene-d_8) δ -72.56 (d, J = 9.4 Hz, 3F).

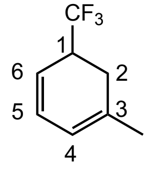

Preparation of 4-Me. This compound was prepared using General Procedure A using 0.027 mmol of **Co1-h.** The colorless liquid obtained following vacuum transfer was obtained in 32% yield (0.00875 mmol) relative to 1,3,5-tris(trifluoromethyl)benzene and was a mixture of 2-methyl-6-(trifluoromethyl)cyclohexa-1,3-diene (**3-Me**; 50% yield, 0.0135 mmol) and 1-methyl-5-(trifluoromethyl)cyclohexa-1,3-diene (**4-Me**; 32% yield, 0.00875 mmol) assigned using ^1H,

$^{13}C\{^1H\}$, and ^{19}F NMR spectroscopy. ^1H NMR (400 MHz, toluene-d_8) δ 5.67 (m, 1H, C^5H), 5.33-5.25 (m, 2H, C^6H + C^4H), 2.62 (m, 1H, C^1H), 2.05 -1.93 (m, 1H, C^2H), 1.80 -1.70 (m, 1H, C^2H), 1.36 (s, 3H, CH_3). $^{13}C\{^1H\}$ NMR (101 MHz, toluene-d_8) δ 134.69 (s, C^3), 128.37 (s, C^5), 119.42 (s, C^4), 116.25 (q, J = 3.1 Hz, C^6), 40.10 (q, J = 27.2 Hz, C^1), 27.79 (q, J = 2.8 Hz, C^2), 23.14 (s, CH_3). CF_3 resonance could not be identified due to signal broadening. ^{19}F NMR (376 MHz, toluene-d_8) δ -72.48 (d, J = 9.4 Hz, 3F).

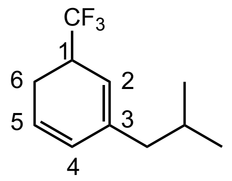

Preparation of 3-^{i}Bu. This compound was prepared using General Procedure A using 0.032 mmol of **Co1-I.** The colorless liquid obtained following vacuum transfer was obtained in 47% yield (0.015 mmol) relative to 1,3,5-tris(trifluoromethyl)benzene and was a mixture of 2-isobutyl-6-(trifluoromethyl)cyclohexa-1,3-diene (**3-^{i}Bu**; 47% yield) and 1-isobutyl-5-(trifluoromethyl)cyclohexa-1,3-diene (**4-^{i}Bu**; 20% yield, 0.00625 mmol) assigned using ^1H, $^{13}C\{^1H\}$, and ^{19}F NMR spectroscopy. ^1H NMR (400 MHz, benzene-d_6) 5.62 (ddd, J = 9.6, 2.8, 1.4 Hz, 1H, C^5H), 5.45 (m, 1H, C^4H), 5.27 (m, 1H, C^2H), 2.86 – 2.67 (m, 1H, C^1H), 2.20- 2.06 (m, 1H, C^6H + overlapping solvent), 2.02 – 1.90 (m, 1H, C^6H + overlapping solvent), 1.82 – 1.73 (m, 2H, CH_2 + overlapping solvent), 1.53 – 1.42 (m, 1H, CH + overlapping solvent), 0.81 – 0.76 (m, 6H, CH_3 + overlapping solvent). $^{13}C\{^1H\}$ NMR (101 MHz, benzene-d_6) δ 138.74 (s, C^3), 127.07 (s, C^5), 124.86 (s, C^4), 114.44 (q, J = 4.2 Hz, C^2), 45.13 (s, CH_2 + overlapping solvent), 39.10 (q, J = 27.7 Hz, C^1), 27.27 (s, CH), 26.21 (app br s, C^6), 23.09 (s, CH_3). CF_3 resonance could not be identified due to signal broadening. ^{19}F NMR (376 MHz, benzene-d_6) δ -72.48 (d, J = 9.4 Hz, 3F).

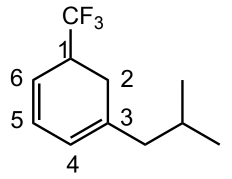

Preparation of 4-^{i}Bu. This compound was prepared using General Procedure A using 0.032 mmol of **Co1-I.** The colorless liquid obtained following vacuum transfer was obtained in 20% yield (0.00625 mmol) relative to 1,3,5-tris(trifluoromethyl)benzene and was a mixture of 2-isobutyl-6-(trifluoromethyl)cyclohexa-1,3-diene (**3-^{i}Bu**; 47% yield, 0.015 mmol) and 1-isobutyl-5-(trifluoromethyl)cyclohexa-1,3-diene (**4-^{i}Bu**; 20% yield, 0.00625 mmol) assigned using ^{1}H, ^{13}C{^{1}H}, and ^{19}F NMR spectroscopy. ^{1}H NMR (400 MHz, benzene-d_6) δ 5.81 (m, 1H, C^{4}H), 5.45 (m, 2H, C^{5}H + C^{6}H), 2.86 – 2.67 (m, 1H, C^{1}H), 2.20- 2.06 (m, 1H, C^{2}H + overlapping solvent), 2.02 – 1.90 (m, 1H, C^{2}H + overlapping solvent), 1.82 – 1.73 (m, 2H, CH_2 + overlapping solvent), 1.53 – 1.42 (m, 1H, CH + overlapping solvent), 0.81 – 0.76 (m, 6H, CH_3 + overlapping solvent). ^{13}C{^{1}H} NMR (101 MHz, benzene-d_6) δ 137.54 (s, C^3), 128.18 (s, C^4), 119.85 (s, C^5), 116.52 (q, *J* = 2.8 Hz, C^6), 46.72 (s, CH_2 + overlapping solvent), 39.70 (q, *J* = 28.5 Hz, C^1), 26.17 (app br s, C^2), 25.66 (s, CH), 22.71 (s, CH_3). CF_3 resonance could not be identified due to signal broadening. ^{19}F NMR (376 MHz, benzene-d_6 δ -72.22 (d, *J* = 9.3 Hz, 3F).

i. Preparation and Characterization of Diels-Alder Products

General Procedure A. A 20 mL scintillation vial was charged with 1 equivalent of N-phenyl maleimide and a 0.05 M benzene-d_6 solution was prepared. The solution was added to a thick-walled glass vessel equipped with a Teflon-coated magnetic stir bar with 1 equivalent of cyclohexadiene. The vessel was heated to 78 °C and stirred for 24 to 48 h. The solution was cooled to room temperature and concentrated to yield an off-white powder quantified by ^{1}H, ^{19}F, and ^{13}C{^{1}H} NMR spectroscopy relative to 0.25 M 1,3,5-tris(trifluoromethyl)benzene cyclohexane-d_{12} solution. Racemic standards were prepared via the protonation of insertion products generated from the achiral dcype-supported, **Co2**, and then subjected to the procedure described above.

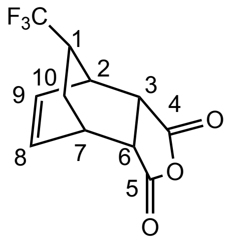

Preparation of 2. 7 mg of maleic anhydride (0.071 mmol, 1.05 equiv.) was brought into a 0.05 M toluene solution and added to a thick-walled glass vessel equipped with a Teflon-coated magnetic stir-bar with 10 mg of **1** (0.068 mmol, 1 equiv.). The vessel was heated to 95 °C and stirred for 24 h. The solution was cooled to room temperature and concentrated to yield 61% of **2** as crystalline white powder quantified through relative integration. Single-crystals suitable for X-ray diffraction were grown through a layered saturated solution of chloroform with pentane at room temperature, yielding 3 mg (17% yield) of **2** assigned by ^{1}H, ^{19}F, and ^{13}C{^{1}H} NMR spectroscopy relative to 0.25 M 1,3,5-tris(trifluoromethyl)benzene cyclohexane-d_{12} solution. ^{1}H NMR (400 MHz, benzene-d_6) δ 5.72 – 5.45 (m, 2H, $C^{8/9}$H), 2.96 (td, J = 3.5, 1.6 Hz, 1H, C^{2}H), 2.45 (dd, J = 5.7, 2.8 Hz, 1H, C^{7}H), 1.88 (qd, J = 9.0, 3.1 Hz, 2H, $C^{3/6}$H), 1.42 (ddt, J = 9.5, 6.3, 1.7 Hz, 1H, C^{1}H), 0.96 – 0.71 (m, 2H, C^{10}H). ^{13}C{^{1}H} NMR (101 MHz, benzene-d_6) δ 171.33 (s, $C^{4/5}$), 170.76 (s, $C^{4/5}$), 133.08 (s, C^{8}), 130.08 (C^{9}), 44.57 (s, C^{3}), 42.98 (C^{6}), 39.58 (q, J = 26.6 Hz, C^{1}), 31.64 (s, C^{7}), 30.90 (d, J = 2.8 Hz, C^{2}), 25.56 (s, C^{10} + overlapping cyclohexane-d_{12}). CF_3 resonance could not be identified due to signal broadening. ^{19}F NMR (376 MHz, benzene-d_6) δ -68.86 (d, J = 10.0 Hz, 3F, *exo*-minor), -70.11 (d, J = 9.3 Hz, 3F, *endo*-major).

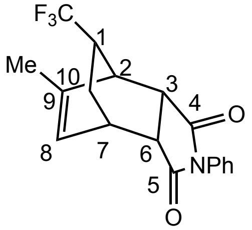

Preparation of 5-Me. This compound was prepared using General Procedure A using 0.0225 mmol combined mixture of **3-Me** and **4-Me.** The white solid obtained following concentration was

5-Me obtained in 59% yield (0.013 mmol) relative to 1,3,5-tris(trifluoromethyl)benzene and was a mixture of **5-Me** (59% yield, 0.013 mmol) and **6-Me** (35% yield, 0.008 mmol), as assigned using ^{19}F NMR spectroscopy. The compound was further purified by preparative HPLC and assigned using ^{1}H, ^{13}C{^{1}H}, and ^{19}F NMR spectroscopy. **HRMS** (ESI+) m/z calculated for [$C_{18}H_{16}F_3NO_2^+$] ([M+H]+) 336.1133 found m/z 336.1197. ^{1}H NMR (400 MHz, chloroform-*d*) δ 7.67 (dd, *J* = 8.7, 1.2 Hz, 1H, Ar), 7.49 – 7.21 (m, 2H, Ar), 7.18-7.09 (m, 2H, Ar), 5.97 (dt, *J* = 6.4, 1.7 Hz, 1H, C^{8}H), 3.37 (dt, *J* = 3.4, 1.7 Hz, 1H, C^{2}H), 3.28 (dq, *J* = 6.0, 2.9 Hz, 1H, C^{7}H), 3.09 (dd, *J* = 8.2, 3.4 Hz, 1H, C^{3}H), 2.99 (dd, *J* = 8.2, 2.9 Hz, 1H, C^{6}H), 2.63 – 2.44 (m, 1H, C^{1}H), 1.97 (ddd, *J* = 13.1, 10.1, 2.8 Hz, 1H, C^{10}H), 1.83 (d, *J* = 1.7 Hz, 3H, CH_3), 1.51 (m, 1H, C^{10}H). ^{13}C{^{1}H} NMR (101 MHz, chloroform-*d*) δ 177.34 (s, C^{4}), 176.33 (s, C^{5}), 139.40 (s, C^{9}), 129.33 (app d, Ar), 129.14 (app d, Ar), 126.56 (app d, Ar), 124.68 (s, C^{8}), 124.65 (app d, Ar), 120.22 (app d, Ar), 44.22 (s, C^{3}), 43.57 (s, C^{6}), 40.39 (q, *J* = 27.1 Hz, C^{1}), 36.49 (q, *J* = 2.8 Hz, C^{2}), 32.69 (s, C^{7}), 26.89 (s, C^{10}), 21.35 (s, CH_3). CF_3 and C(N) resonance could not be identified due to signal broadening. ^{19}F NMR (376 MHz, chloroform-*d*) δ -70.48 (d, *J* = 9.5 Hz, 3F).

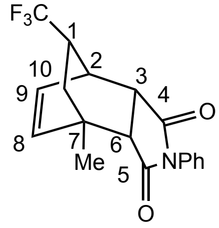

Preparation of 6-Me. This compound was prepared using General Procedure A using 0.0225 mmol of a mixture of **3-Me** and **4-Me.** The white solid obtained following concentration was **6-Me** obtained in 35% yield (0.008 mmol) relative to 1,3,5-tris(trifluoromethyl)benzene and was a mixture of **5-Me** (59% yield, 0.013 mmol) and **6-Me** (35% yield, 0.008 mmol), as assigned using ^{19}F NMR spectroscopy. The compound was further purified by preparative HPLC and assigned using ^{1}H, ^{13}C{^{1}H}, and ^{19}F NMR spectroscopy. **HRMS** (ESI+) m/z calculated for [$C_{18}H_{16}F_3NO_2^+$] ([M+H]+) 336.1133 found m/z 336.1197. ^{1}H NMR (400 MHz, chloroform-*d*) δ 7.67 (dd, *J* = 8.7, 1.2 Hz, 1H, Ar),

7.49 – 7.21 (m, 2H, Ar), 7.18-7.09 (m, 2H, Ar), 6.43 (d, *J* = 13.3 Hz, 1H, $C^{8/9}H$), 6.21 (d, *J* = 13.4 Hz, 1H, $C^{8/9}H$), 3.55 (ddt, *J* = 6.2, 3.1, 1.5 Hz, 1H, C^2H), 3.12 (d, *J* = 3.2 Hz, 1H, C^3H), 2.74 (d, *J* = 8.2 Hz, 1H, C^6H), 2.57 (m, 1H, C^1H), 1.83 (app s, 1H, $C^{10}H$), 1.57 (s, 3H, CH_3), 1.40 (app d, 1H, $C^{10}H$). $^{13}C\{^1H\}$ NMR (101 MHz, chloroform-*d*) δ 177.34 (s, C^4), 176.33 (s, C^5), 140.72 (s, $C^{8/9}$), 129.33 (app d, Ar), 129.14 (app d, Ar), 126.56 (app d, Ar), 125.43 (s, $C^{8/9}$), 124.65 (app d, Ar), 120.22 (app d, Ar), 47.39 (s, C^6), 45.73 (s, C^3), 41.91 (q, *J* = 28.2 Hz, C^1), 37.61 (s, C^7), 35.26 (s, C^{10}), 31.73 (s, C^2), 22.36 (s, CH_3). CF_3 and C(N) resonance could not be identified due to signal broadening. ^{19}F NMR (376 MHz, chloroform-*d*) δ -70.04 (d, *J* = 9.3 Hz, 3F).

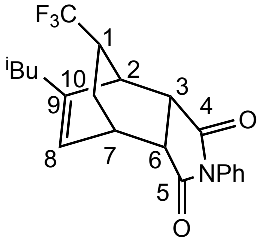

Preparation of 5-^{i}Bu. This compound was prepared using General Procedure A using 0.021 mmol of a mixture of **3-^{i}Bu** and **4-^{i}Bu.** The white solid obtained following concentration was **5-^{i}Bu** obtained in 65% yield (0.0137 mmol) relative to 1,3,5-tris(trifluoromethyl)benzene and was a mixture of **5-^{i}Bu** (65% yield, 0.0137 mmol) and **6-^{i}Bu** (18% yield, 0.0038 mmol), as assigned using ^{19}F NMR spectroscopy. The compound was further purified by preparative HPLC and assigned using ^1H, $^{13}C\{^1H\}$, and ^{19}F NMR spectroscopy. **HRMS** (ESI+) m/z calculated for $[C_{21}H_{22}F_3NO_2^+]$ ($[M+H]^+$) 378.1603 found m/z 378.1667. ^1H NMR (400 MHz, chloroform-*d*) δ 7.44 – 7.40 (m, 2H, Ar), 7.39 – 7.32 (m, 1H, Ar), 7.18 – 7.14 (m, 2H, Ar), 5.94 (dt, *J* = 6.5, 1.8 Hz, 1H, C^8H), 3.34 (dd, *J* = 6.5, 3.0 Hz, 1H, C^7H), 3.32 (dt, *J* = 3.4, 1.7 Hz, 1H, C^2H), 3.08 (dd, *J* = 8.2, 3.5 Hz, 1H, C^3H), 3.01 (dd, *J* = 8.4, 2.9 Hz, 1H, C^6H), 2.51 (m, 1H, C^1H), 1.99 (m, 3H, $C^{10}H + CH_2$), 1.81 (m, 1H, CH), 1.54 (m, 1H, $C^{10}H$), 0.85 (dd, *J* = 11.9, 6.5 Hz, 6H, CH_3). $^{13}C\{^1H\}$ NMR (101 MHz, chloroform-*d*) δ 177.26 (s, C^4), 176.21(s, C^5), 142.15 (s, C^9), 129.27 (app d, Ar), 128.85 (app d, Ar), 126.35 (s, Ar), 123.86 (s, C^8), 44.53 (app d, C^3), 43.79 (s, C^6), 40.71 (q, *J* = 27.1 Hz, C^1), 36.55 (q, *J* = 2.4

Hz, C^2), 32.47 (s, C^7), 27.04 (s, $C^{10}H$ + CH_2), 23.10 (s, CH_3), 22.62 (s, CH_3). CF_3 and C(N) resonance could not be identified due to signal broadening. ^{19}F NMR (376 MHz, chloroform-*d*) δ -70.09 (d, *J* = 9.5 Hz, 3F).

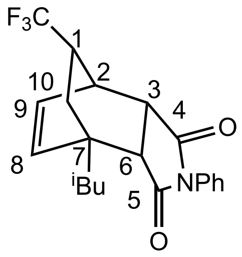

Preparation of 6-^{i}Bu. This compound was prepared using General Procedure A using 0.021 mmol of a mixture of **3-^{i}Bu** and **4-^{i}Bu.** The white solid obtained following concentration was **6-^{i}Bu** obtained in 18% yield (0.0038 mmol) relative to 1,3,5-tris(trifluoromethyl)benzene and was a mixture of **5-^{i}Bu** (65% yield, 0.0137 mmol) and **6-^{i}Bu** (18% yield, 0.0038 mmol), as assigned using ^{19}F NMR spectroscopy. The compound was further purified by preparative HPLC and assigned using ^1H, $^{13}C\{^1H\}$, and ^{19}F NMR spectroscopy. **HRMS** (ESI^+) m/z calculated for [$C_{21}H_{22}F_3NO_2^+$] ($[M+H]^+$) 378.1603 found m/z 378.1667. ^1H NMR (400 MHz, chloroform-*d*) δ 7.44 – 7.40 (m, 2H, Ar), 7.39 – 7.32 (m, 1H, Ar), 7.18 – 7.14 (m, 2H, Ar), 6.20 – 6.10 (m, 2H, $C^{8/9}H$), 3.55 (dd, *J* = 5.2, 2.8 Hz, 1H, C^2H), 3.10 (m, 1H, C^3H), 2.87 (d, *J* = 8.2 Hz, 1H, C^6H), 2.58 (d, *J* = 7.0 Hz, 1H, C^1H), 1.95 (m, 1H, $C^{10}H$), 1.54 – 1.48 (m, 1H, $C^{10}H$), 1.03 (dd, *J* = 19.5, 6.4 Hz, 6H, CH_3). $^{13}C\{^1H\}$ NMR (101 MHz, chloroform-*d*) δ 177.26 (s, C^4), 176.21 (s, C^5), 137.72 (s, $C^{8/9}$), 129.27 (app d, Ar), 128.85 (app d, Ar), 128.54 (s, $C^{8/9}$), 126.35 (s, Ar), 46.93 (s, C^6), 45.49 (s, C^3), 43.83 (s, C^7), 41.71 (q, *J* = 26.0 Hz, C^1), 31.80 (q, J = 2.9 Hz, C^2), 31.47 (s, C^{10} and CH_2), 25.19 (s, CH_3), 24.93 (s, CH), 24.34 (s, CH_3).^{19}F NMR (376 MHz, chloroform-*d*) δ -70.02 (d, *J* = 9.4 Hz, 3F).

VII. NMR Spectroscopic and SFC Data of Organic Products

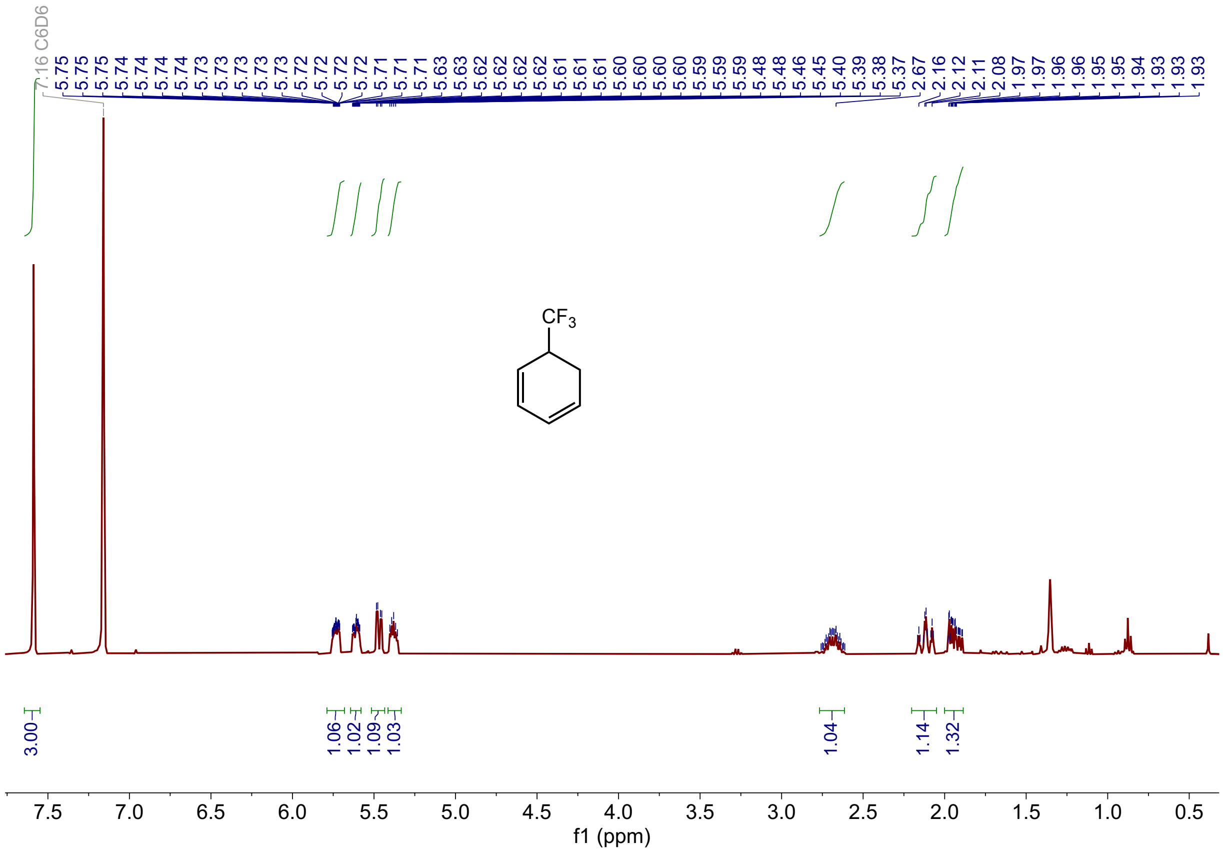

Figure S170. ^{1}H NMR spectrum (400 MHz, benzene-d_6, 23 °C) of **1** with 1,3,5-tris(trifluoromethyl)benzene internal standard (δ 7.59 ppm) in cyclohexane-d_{12} solution.

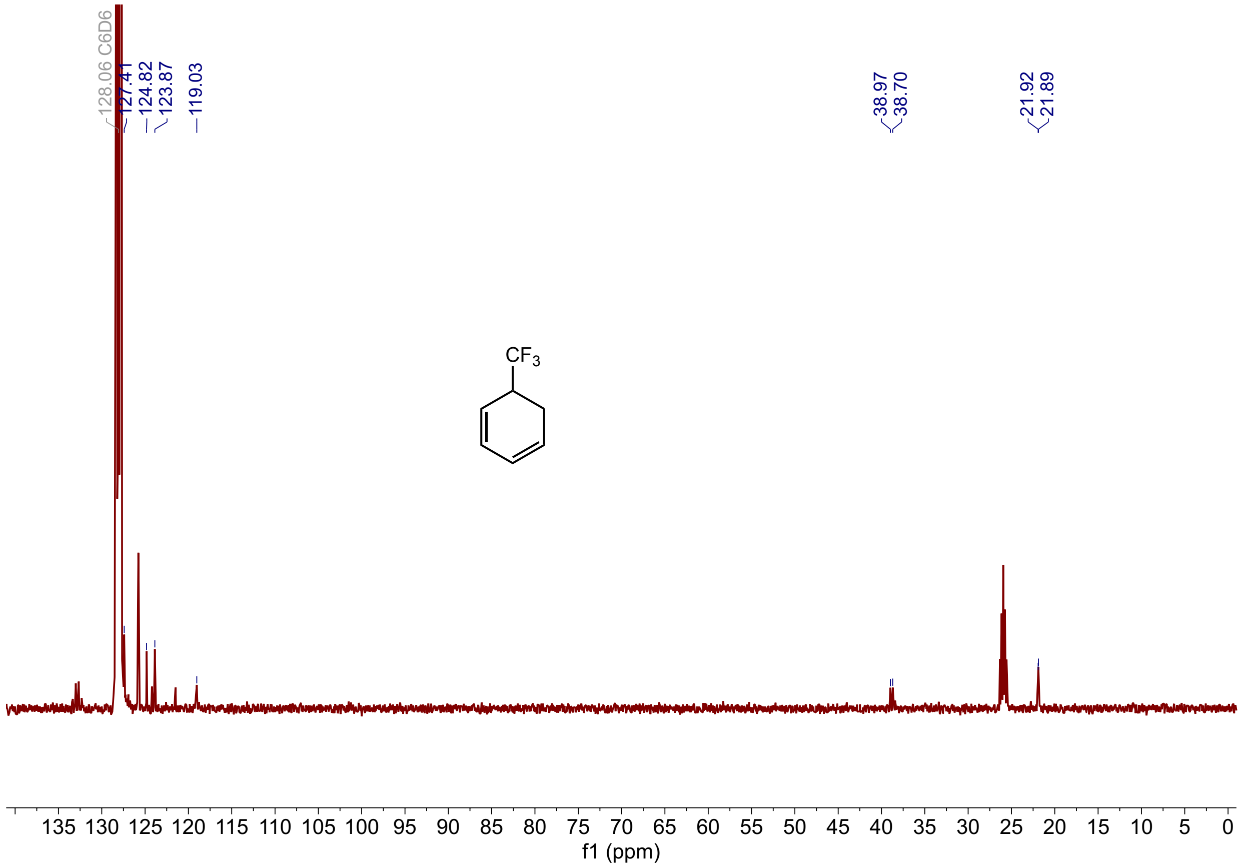

Figure S171. $^{13}C\{^{1}H\}$ NMR spectrum (101 MHz, benzene-d_6, 23 °C) of **1** with 1,3,5-tris(trifluoromethyl)benzene in cyclohexane-d_{12} solution.

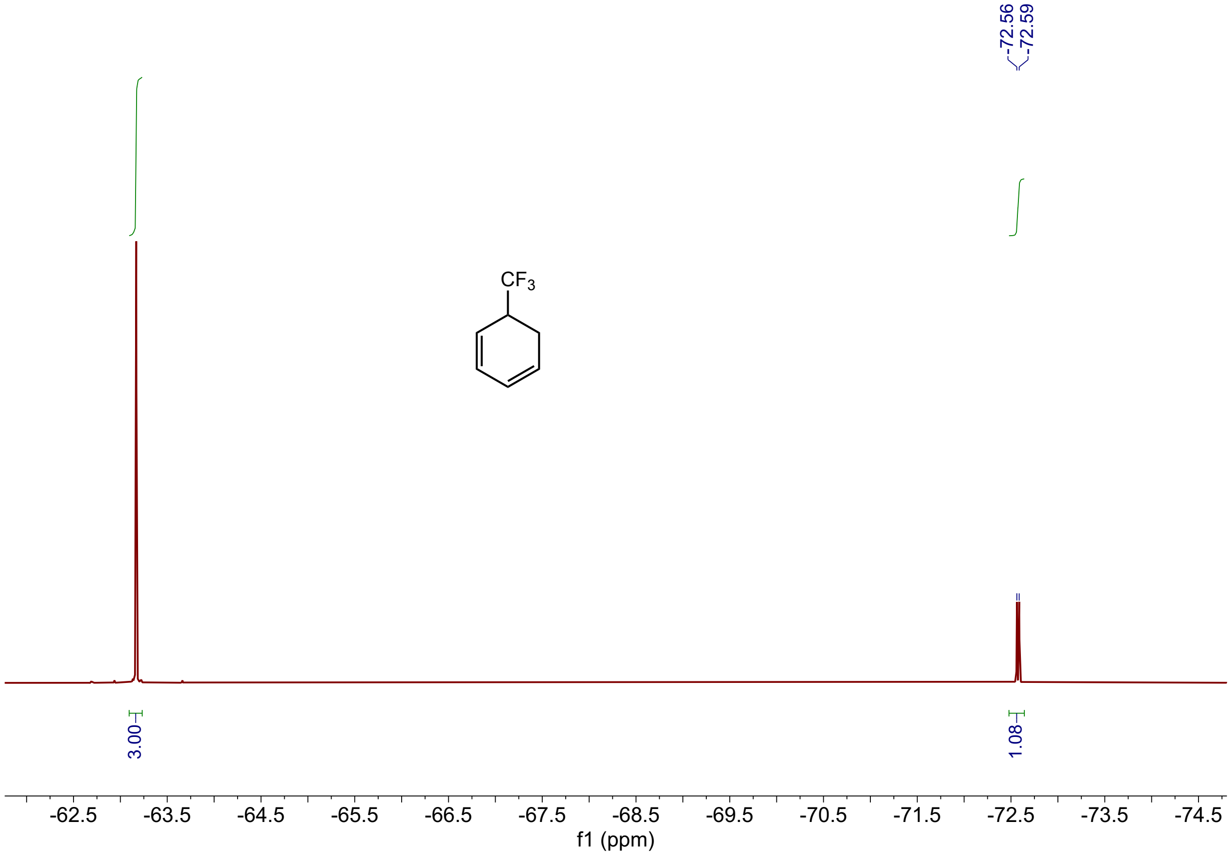

Figure S172. ^{19}F NMR spectrum (376 MHz, benzene-d_6, 23 °C) of **1** with 1,3,5-tris(trifluoromethyl)benzene internal standard (δ -63.17 ppm).

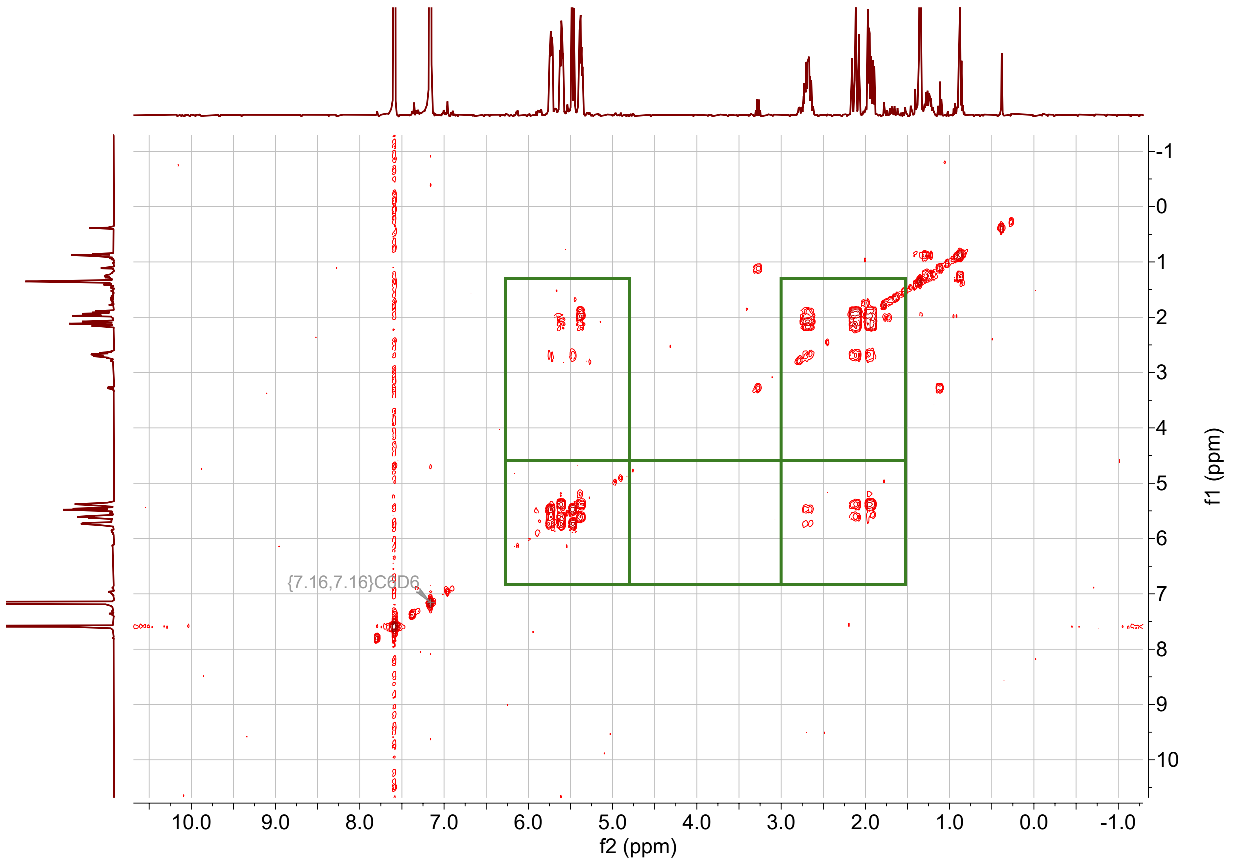

Figure S173. $^{1}H–^{1}H$ COSY NMR spectrum (benzene-d_6, 23 °C) of **1** with 1,3,5-tris(trifluoromethyl)benzene**.** Inset: $^{1}H–^{1}H$ correlation between cyclohexadiene signals.

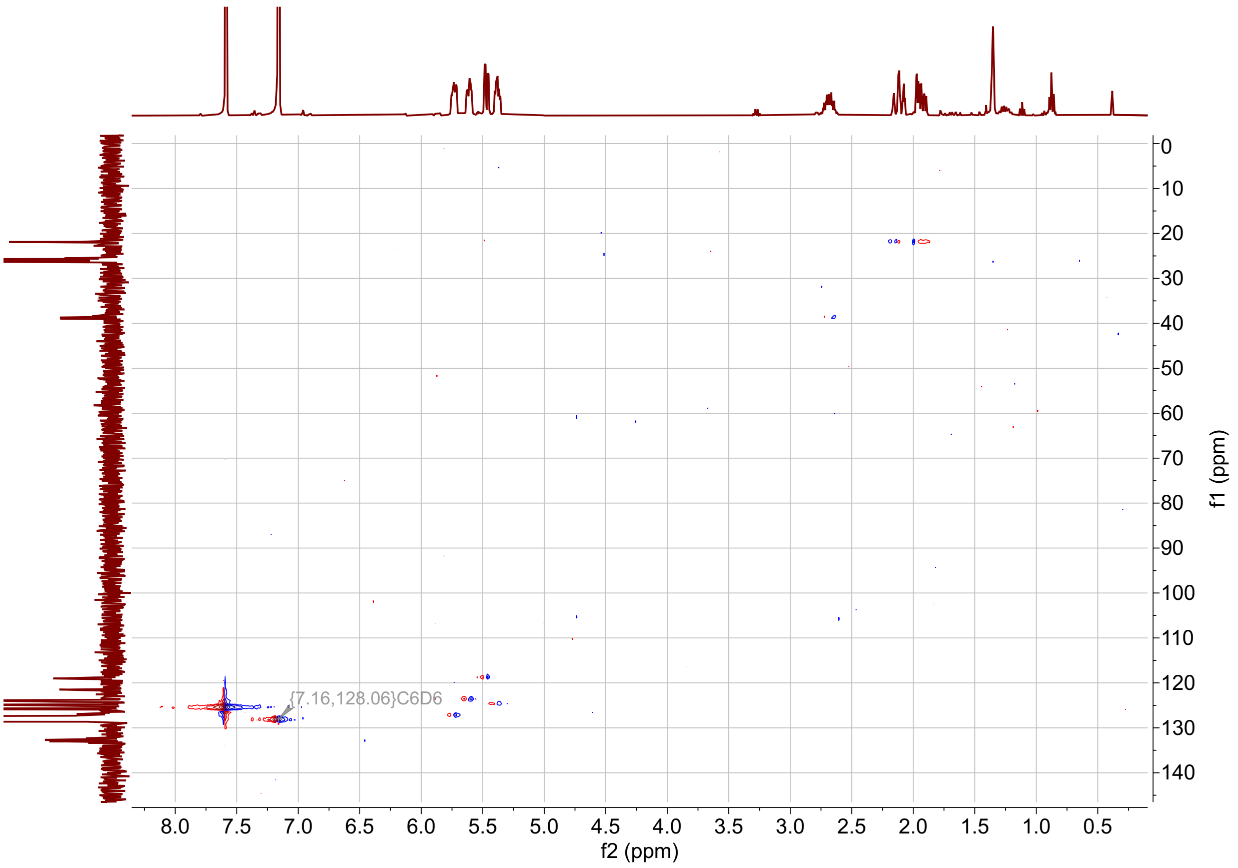

Figure S174. ^{1}H–^{13}C HSQC NMR spectrum (benzene-d_6, 23 °C) of **1** with 1,3,5-tris(trifluoromethyl)benzene**.**

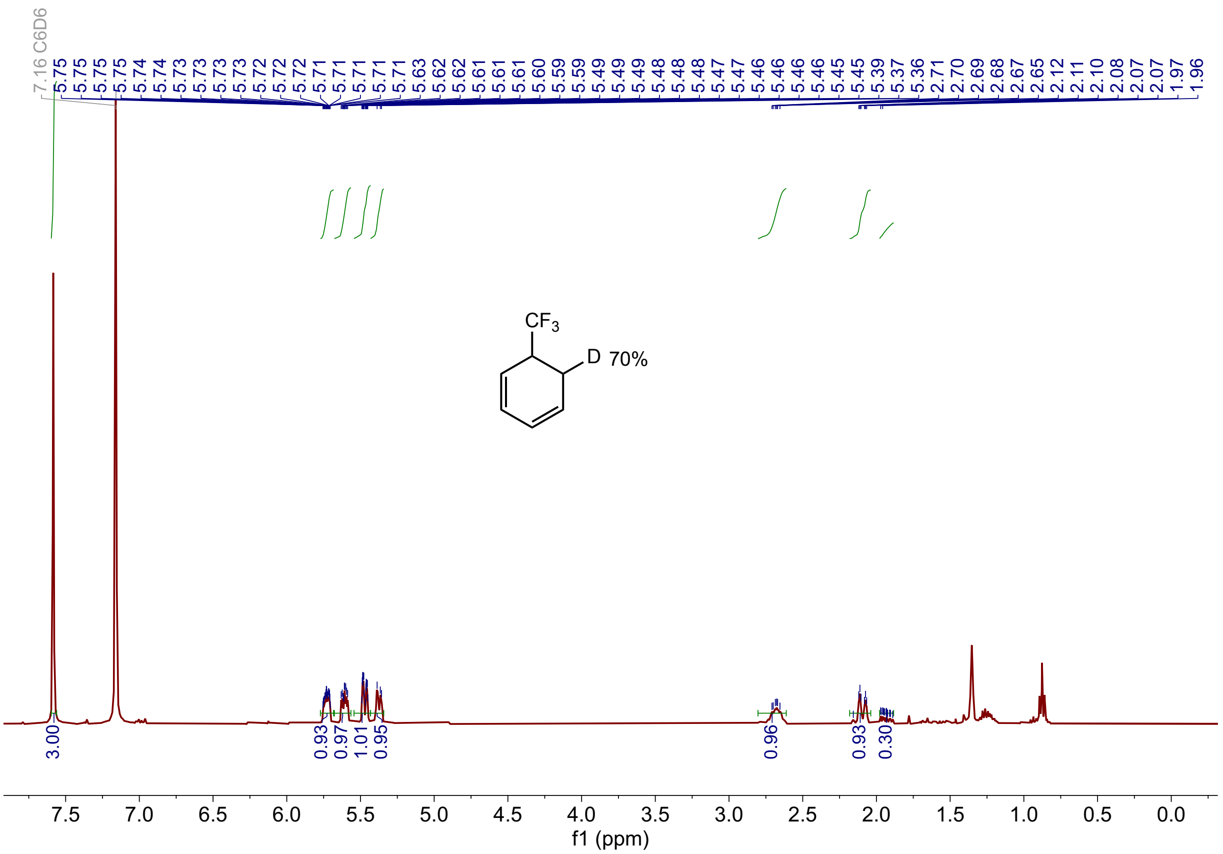

Figure S175. ^{1}H NMR spectrum (400 MHz, benzene-d_6, 23 °C) of **1D** with 1,3,5-tris(trifluoromethyl)benzene internal standard (δ 7.59 ppm) in cyclohexane-d_{12} solution.

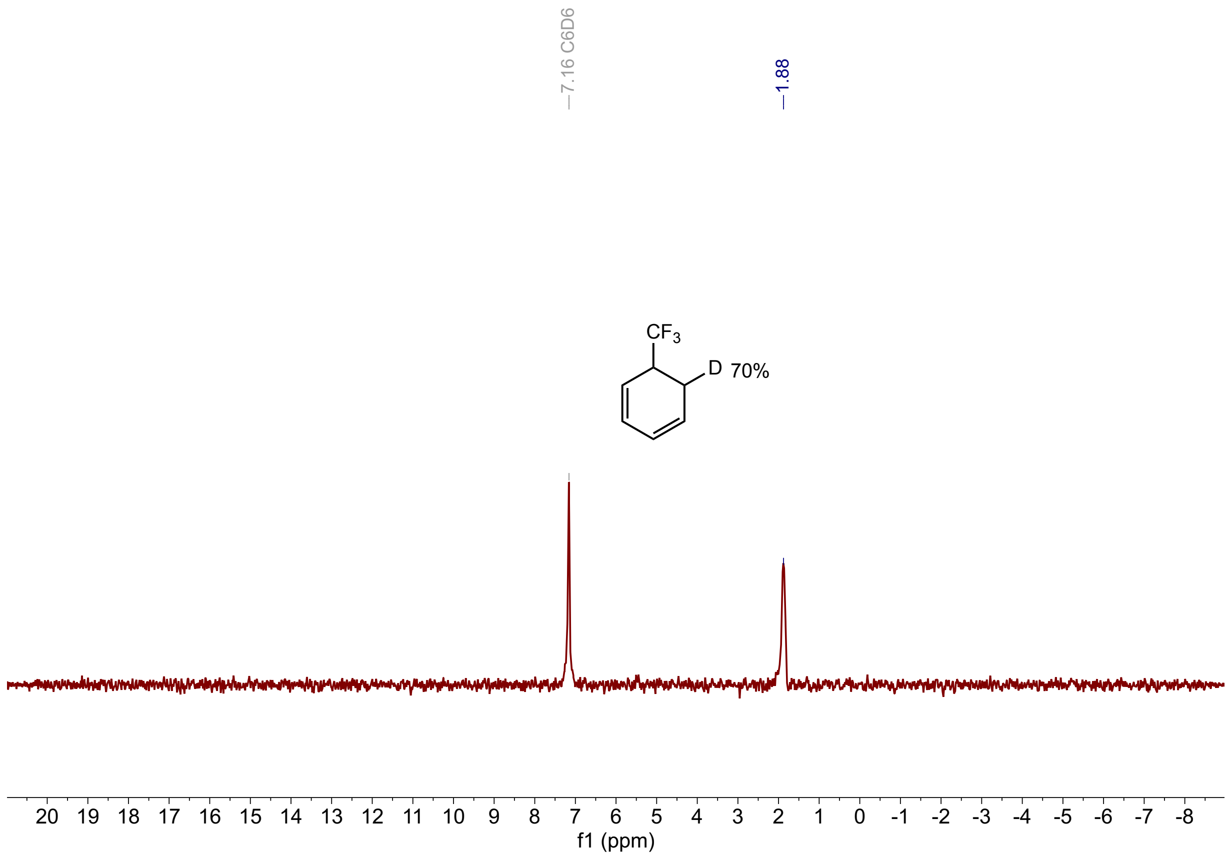

Figure S176. ^{2}H NMR spectrum (61 MHz, benzene, 23 °C) of **1D**.

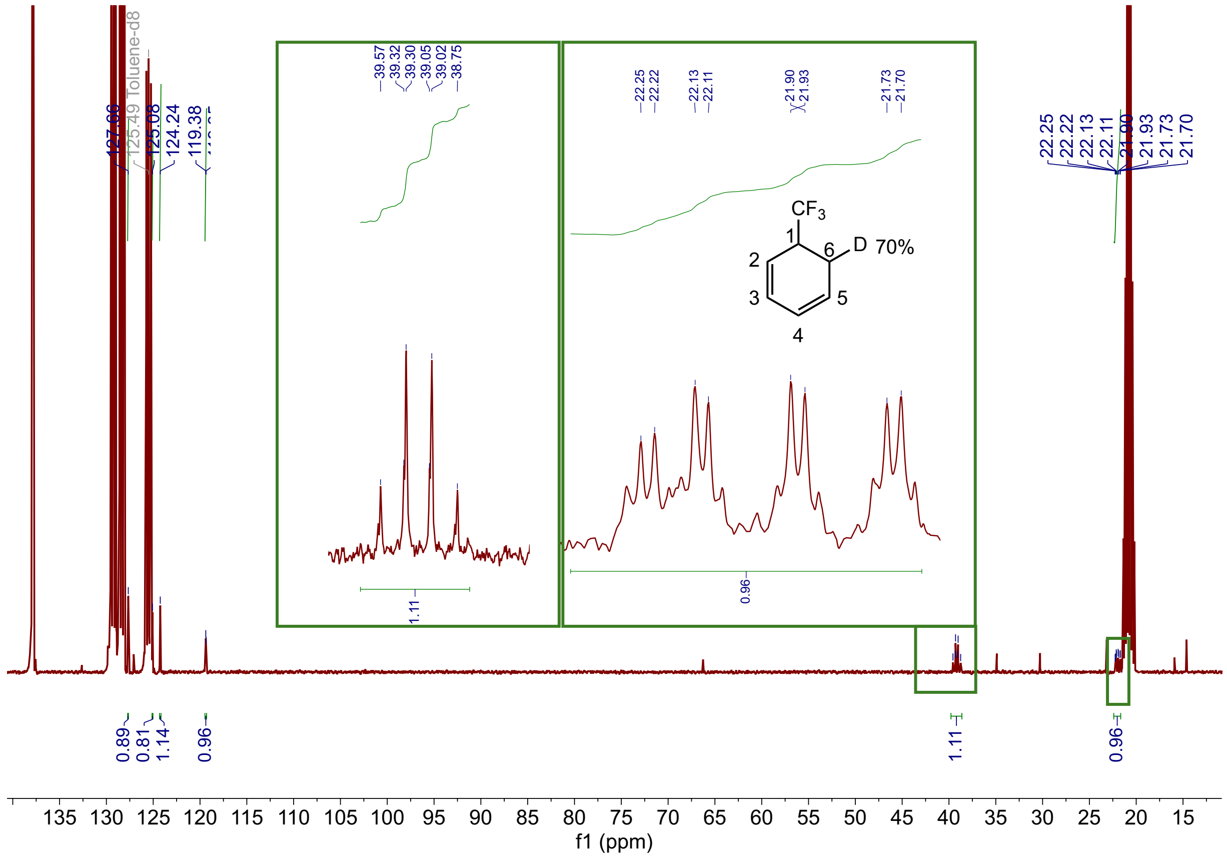

Figure S177. $^{13}C\{^1H\}$ NMR spectrum (101 MHz, toluene-d_8, 23 °C) of **1D**. Inset: C^1 (left) and C^6 (right).

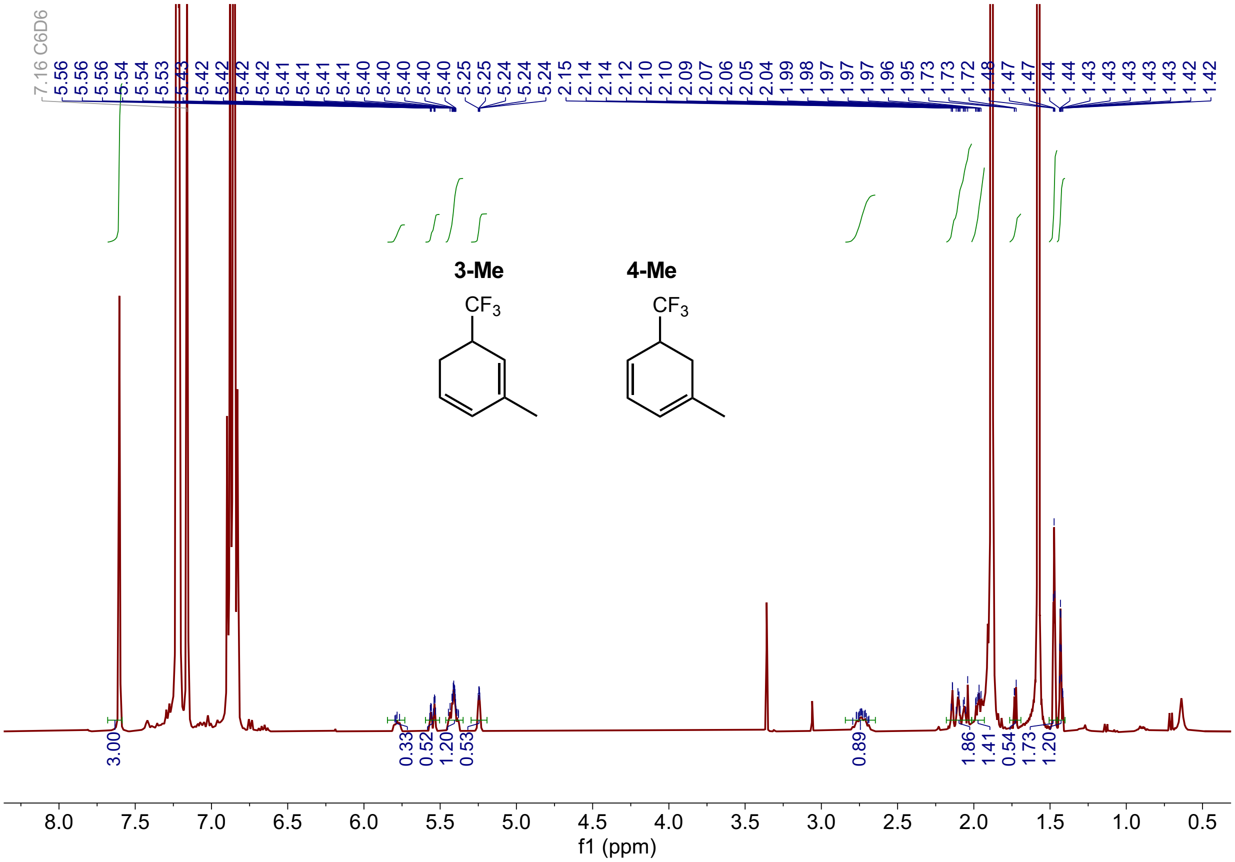

Figure S178. ^{1}H NMR spectrum (400 MHz, benzene-d_6, 23 °C) of **3-Me** and **4-Me** with 1,3,5-tris(trifluoromethyl)benzene internal standard (δ 7.59 ppm) in cyclohexane-d_{12} solution and excess arene.

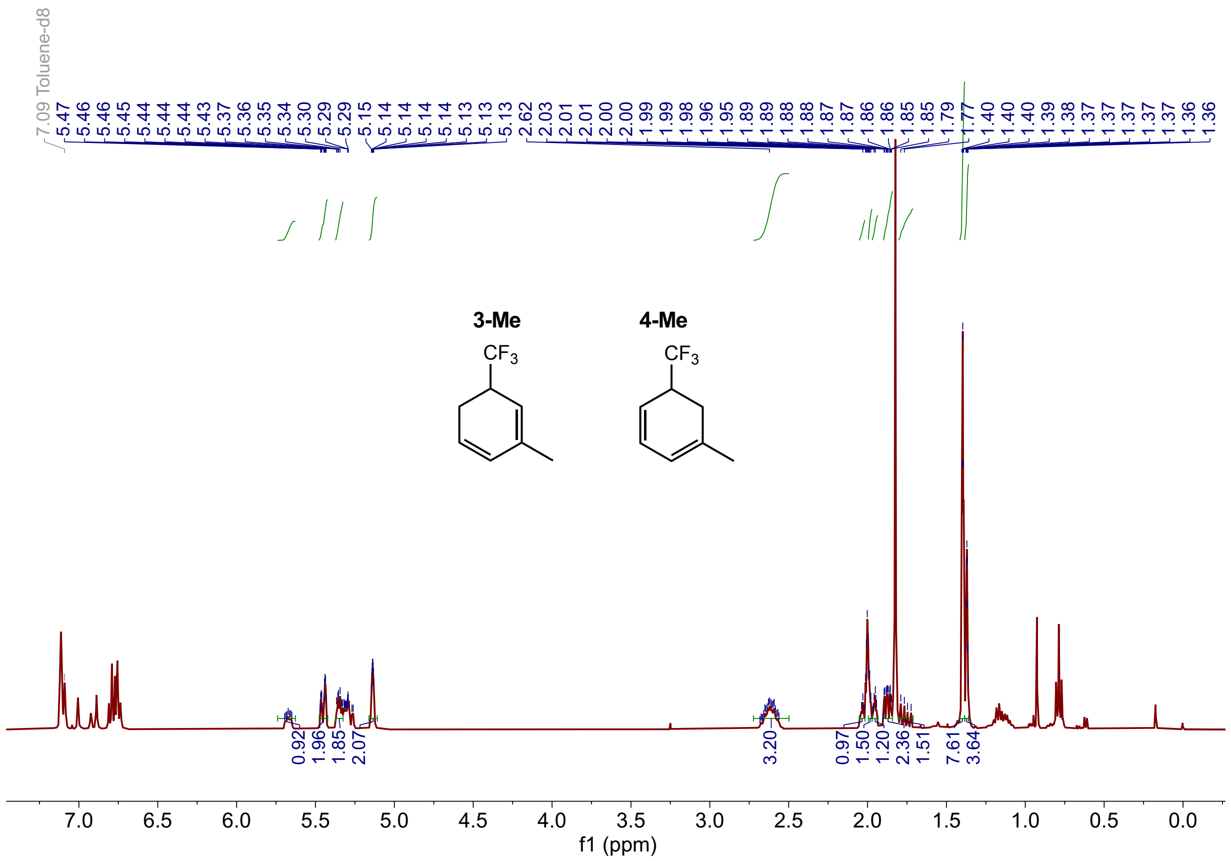

Figure S179. ^{1}H NMR spectrum (400 MHz, toluene-d_8, 23 °C) of **3-Me** and **4-Me** in excess arene.

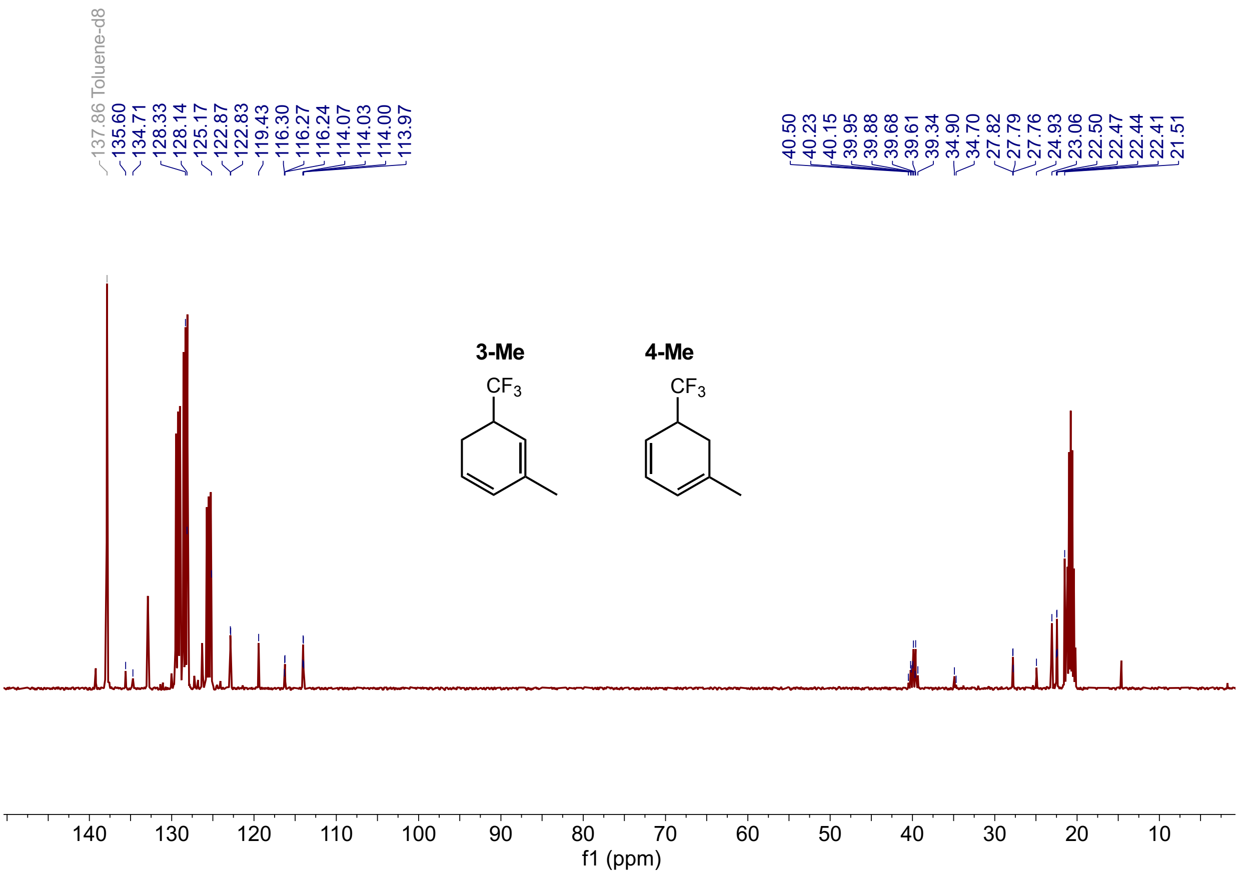

Figure S180. $^{13}C\{^{1}H\}$ NMR spectrum (101 MHz, toluene-d_8, 23 °C) of **3-Me** and **4-Me** in excess arene.

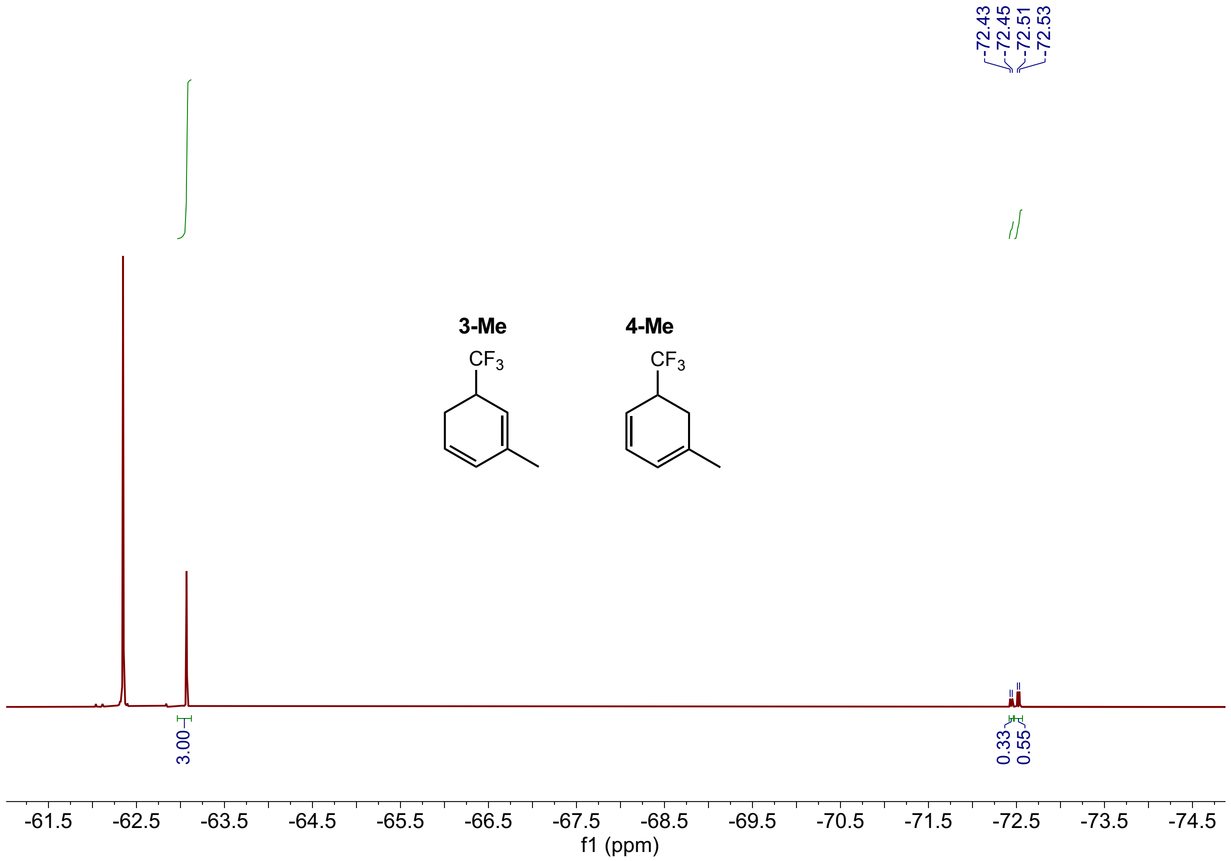

Figure S181. ^{19}F NMR spectrum (376 MHz, benzene-d_6, 23 °C) of **3-Me** and **4-Me** with 1,3,5-tris(trifluoromethyl)benzene internal standard (δ -63.07 ppm) and excess arene (δ -62.35 ppm).

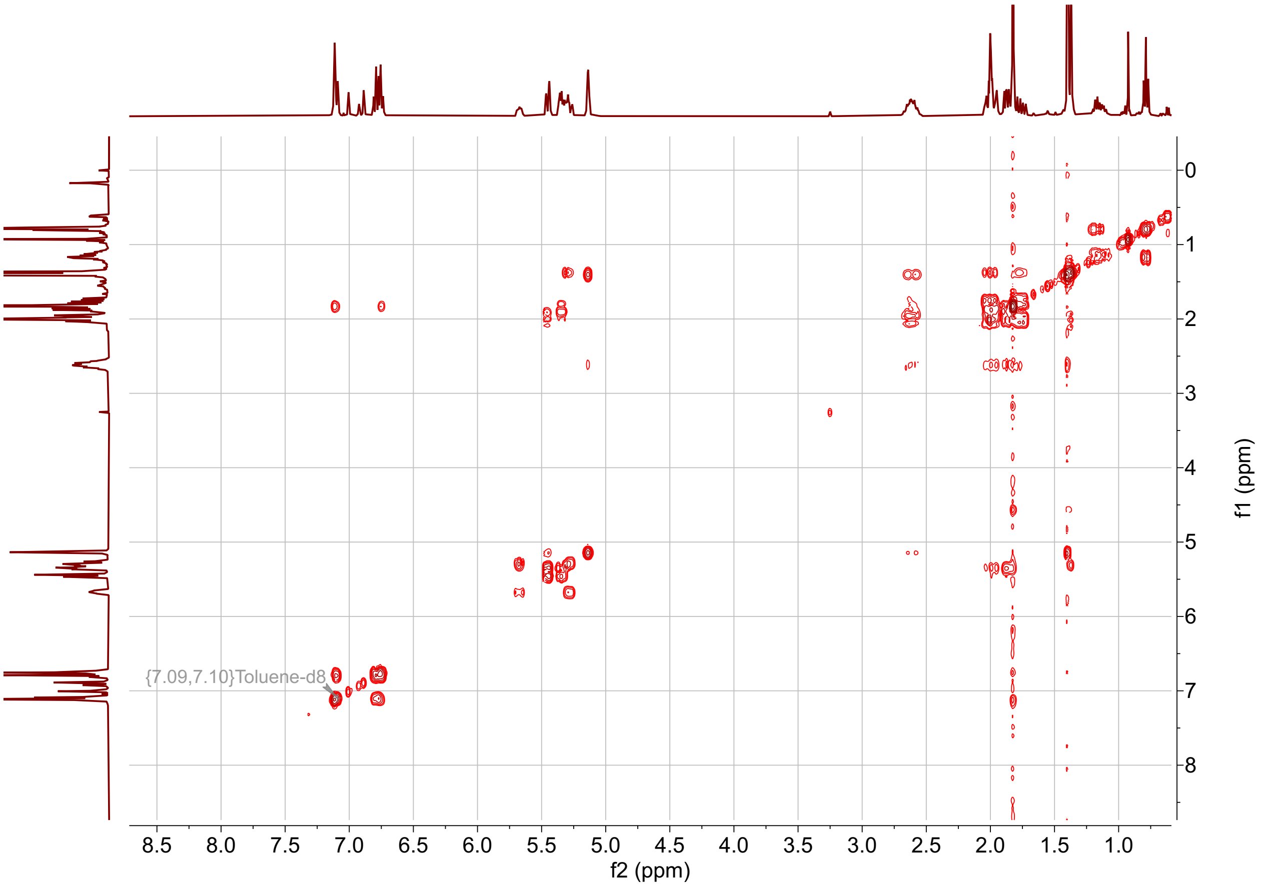

Figure S182. ^{1}H–^{1}H COSY NMR spectrum (toluene-d_8, 23 °C) of **3-Me** and **4-Me** in excess arene.

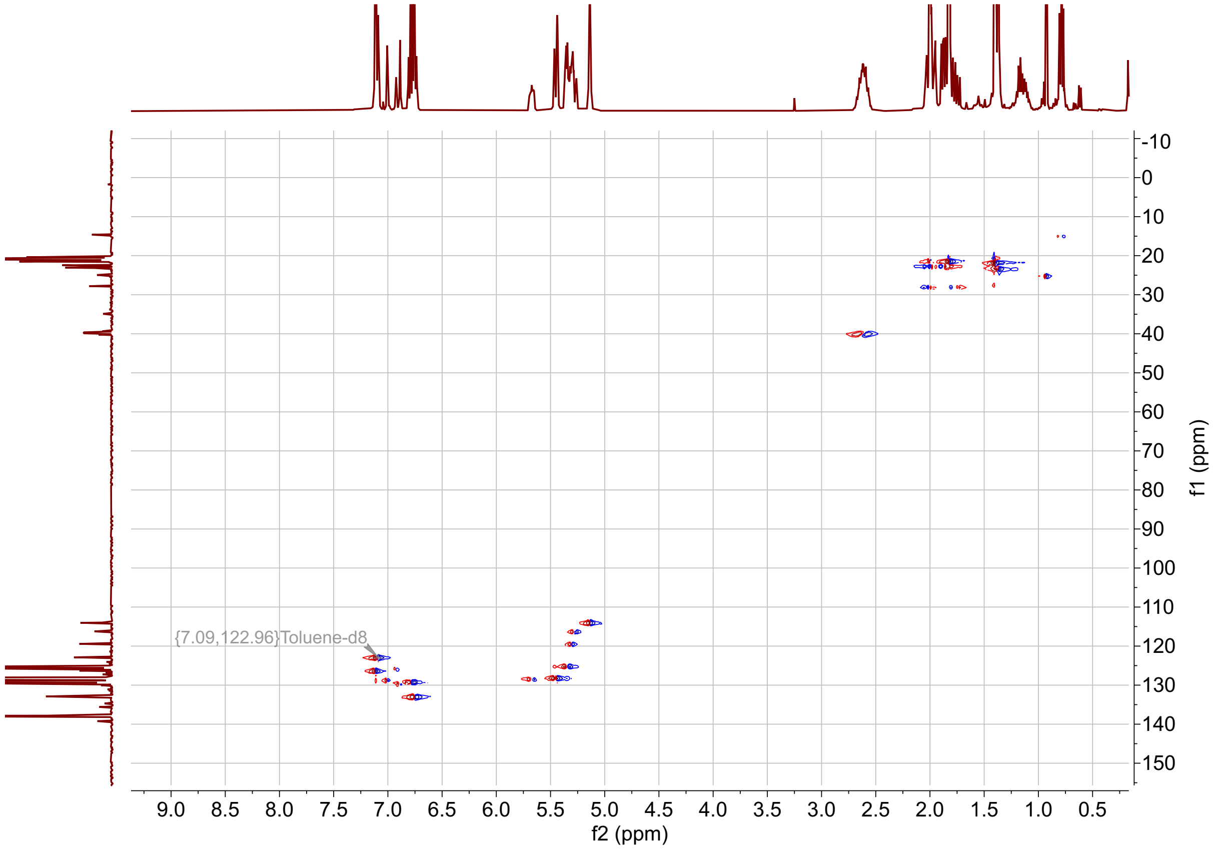

Figure S183. ^{1}H–$^{13}C\{^{1}H\}$ HSQC NMR spectrum (toluene-d_8, 23 °C) of **3-Me** and **4-Me** in excess arene.

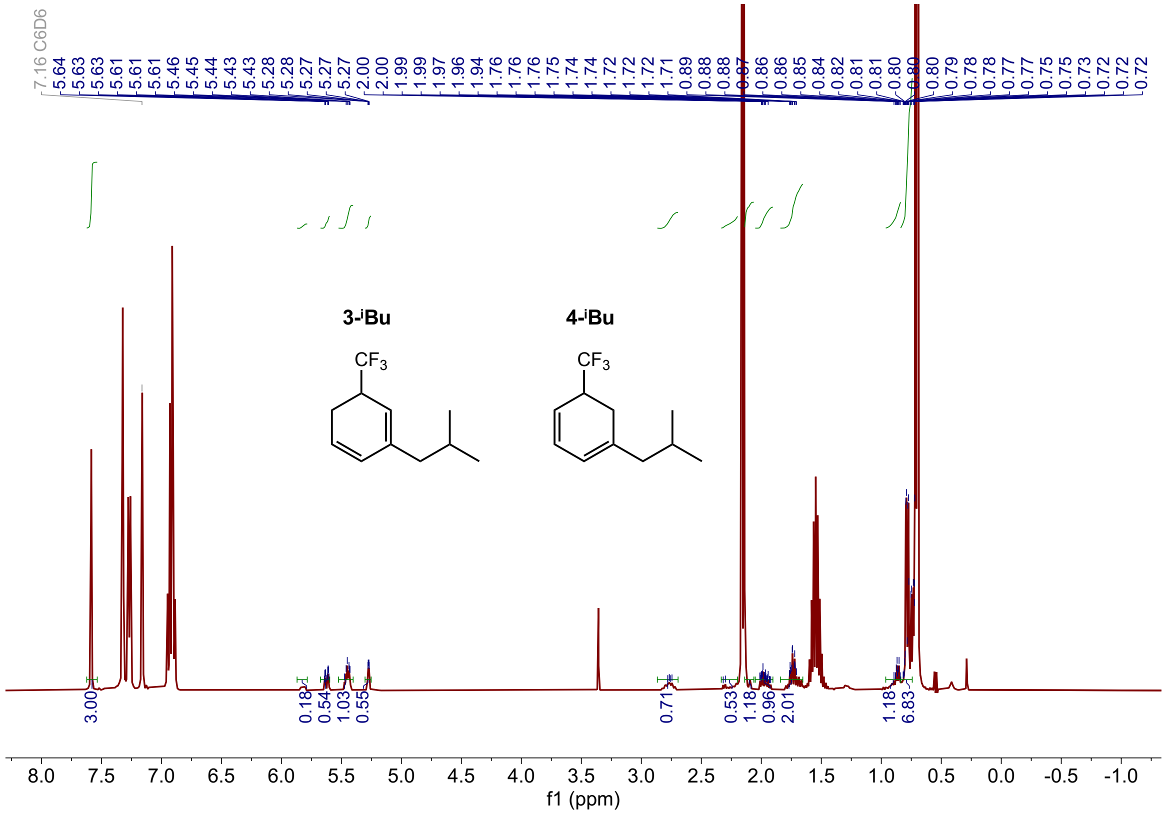

Figure S184. ^{1}H NMR spectrum (400 MHz, benzene-d_6, 23 °C) of **3-^{i}Bu** and **4-^{i}Bu** with 1,3,5-tris(trifluoromethyl)benzene internal standard (δ 7.59 ppm) and excess arene.

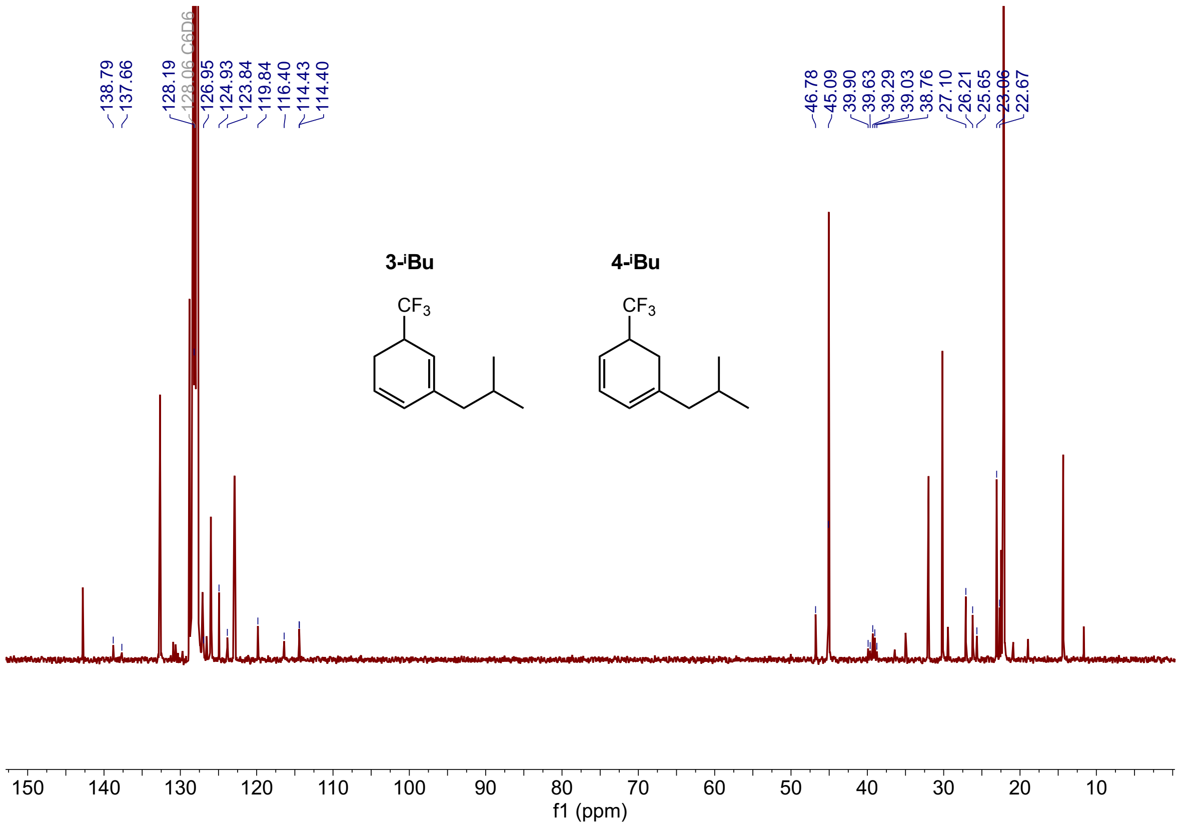

Figure S185. $^{13}C\{^{1}H\}$ NMR spectrum (101 MHz, benzene-d_6, 23 °C) of **3-^{i}Bu** and **4-^{i}Bu** with 1,3,5-tris(trifluoromethyl)benzene and excess arene.

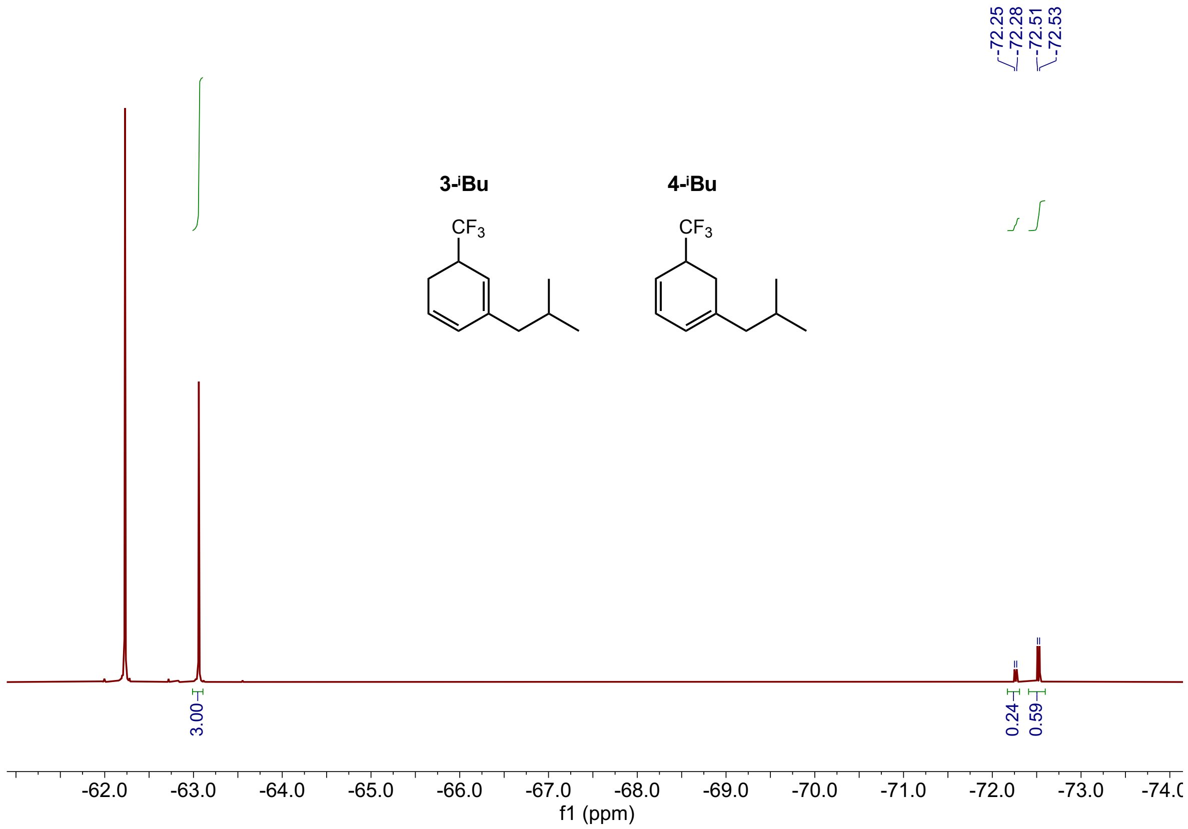

Figure S186. ^{19}F NMR spectrum (376 MHz, benzene-d_6, 23 °C) of **3-ⁱBu** and **4-ⁱBu** with 1,3,5-tris(trifluoromethyl)benzene (δ -63.06 ppm) and excess arene (δ -62.23 ppm).

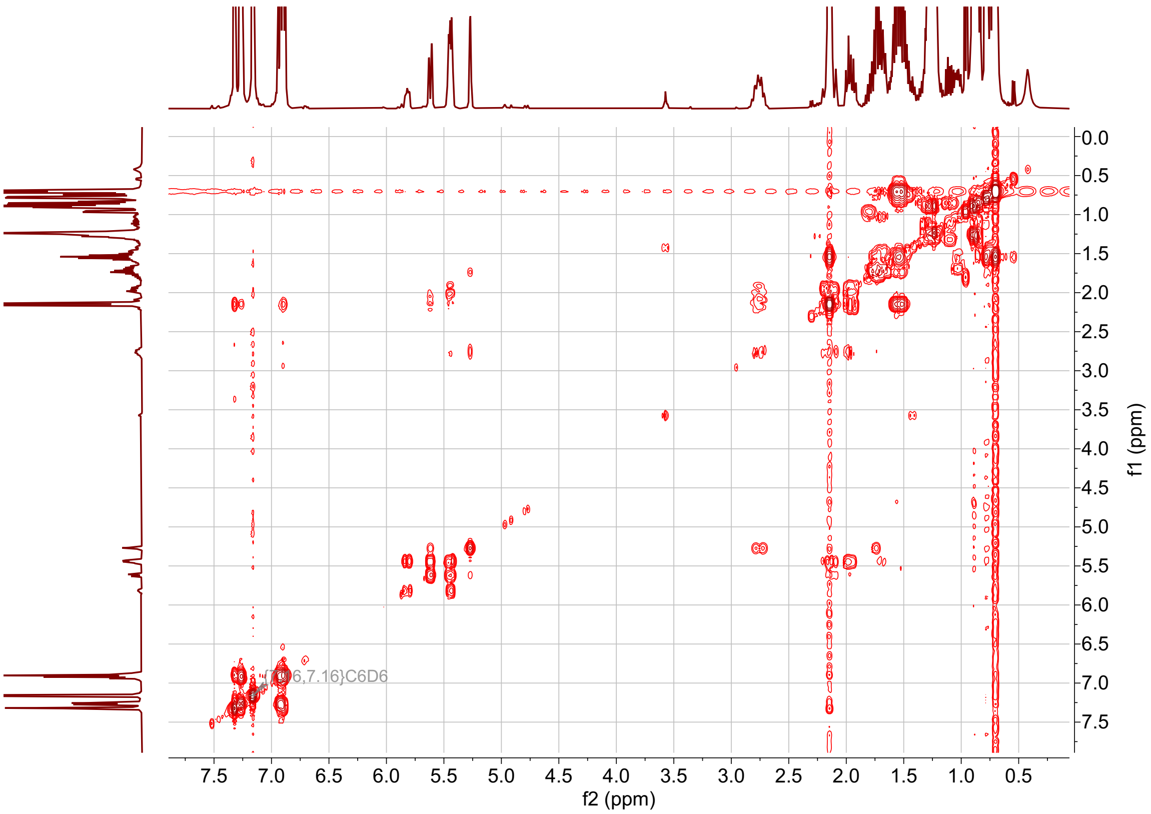

Figure S187. ^{1}H–^{1}H COSY NMR spectrum (benzene-d_6, 23 °C) of **3-^{i}Bu** and **4-^{i}Bu** with 1,3,5-tris(trifluoromethyl)benzene and excess arene**.**

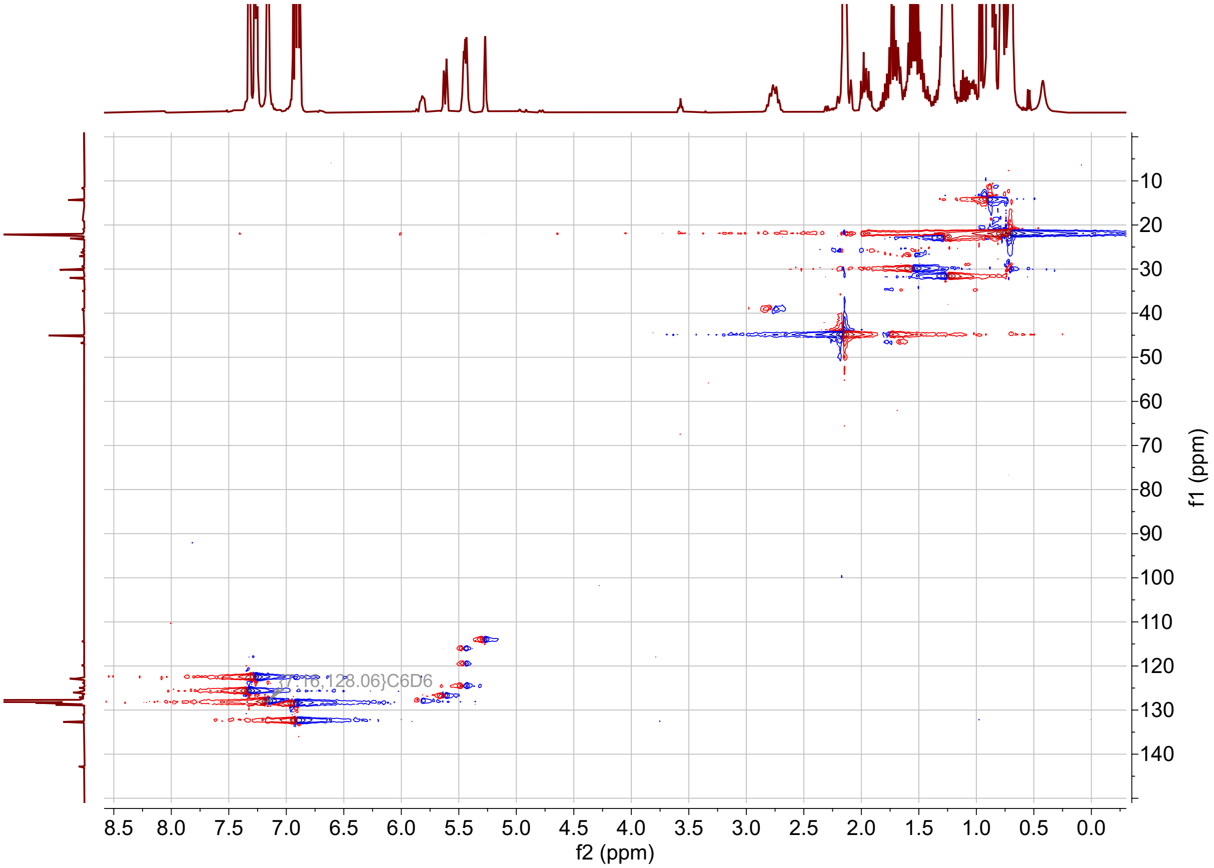

Figure S188. ^{1}H–^{13}C{^{1}H} HSQC NMR spectrum (benzene-d_6, 23 °C) of **3-^{i}Bu** and **4-^{i}Bu** with 1,3,5-tris(trifluoromethyl)benzene and excess arene.

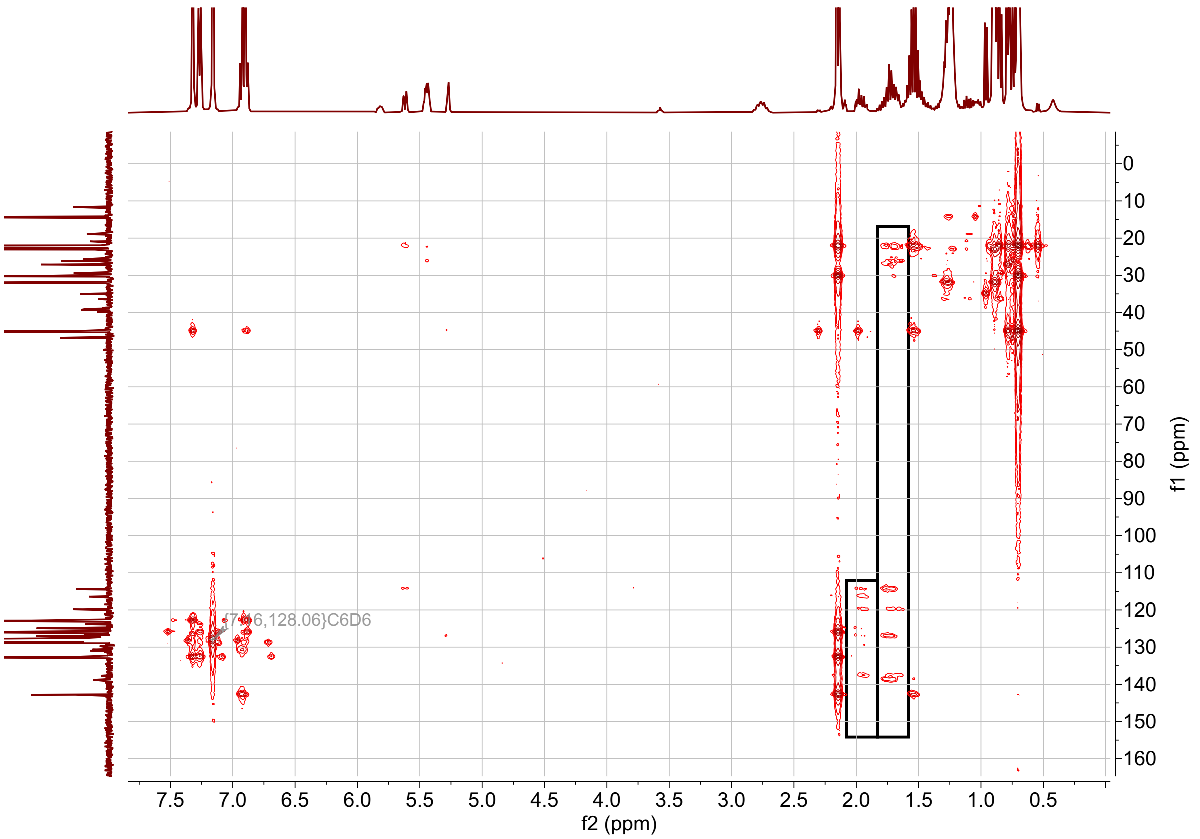

Figure S189. $^{1}H–^{13}C\{^{1}H\}$ HMBC NMR spectrum (benzene-d_6, 23 °C) of **3-^{i}Bu** and **4-^{i}Bu** with 1,3,5-tris(trifluoromethyl)benzene and excess arene. Inset: assignment of quaternary carbons and C(sp^2)-^{i}Bu correlation.

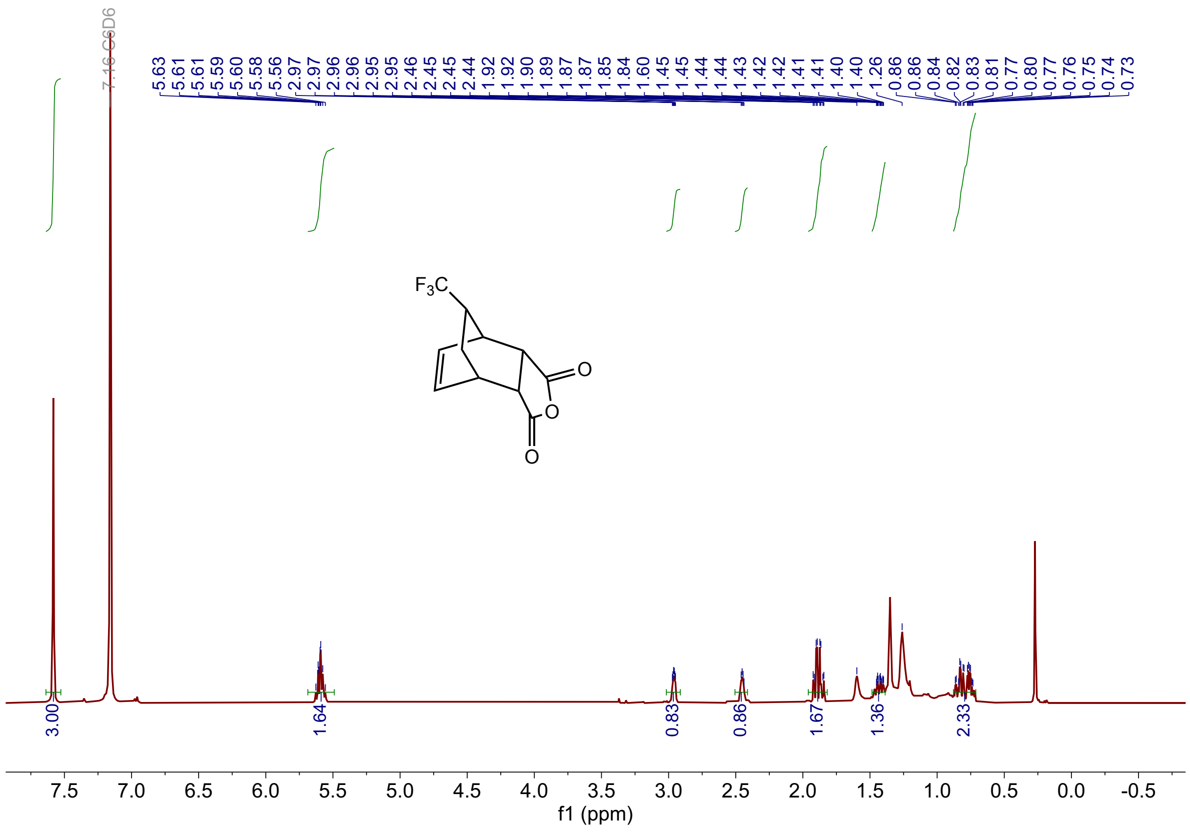

Figure S190. ^{1}H NMR spectrum (400 MHz, benzene-d_6, 23 °C) of isolated **2** with 1,3,5-tris(trifluoromethyl)benzene internal standard (δ 7.59 ppm) in cyclohexane-d_{12} solution.

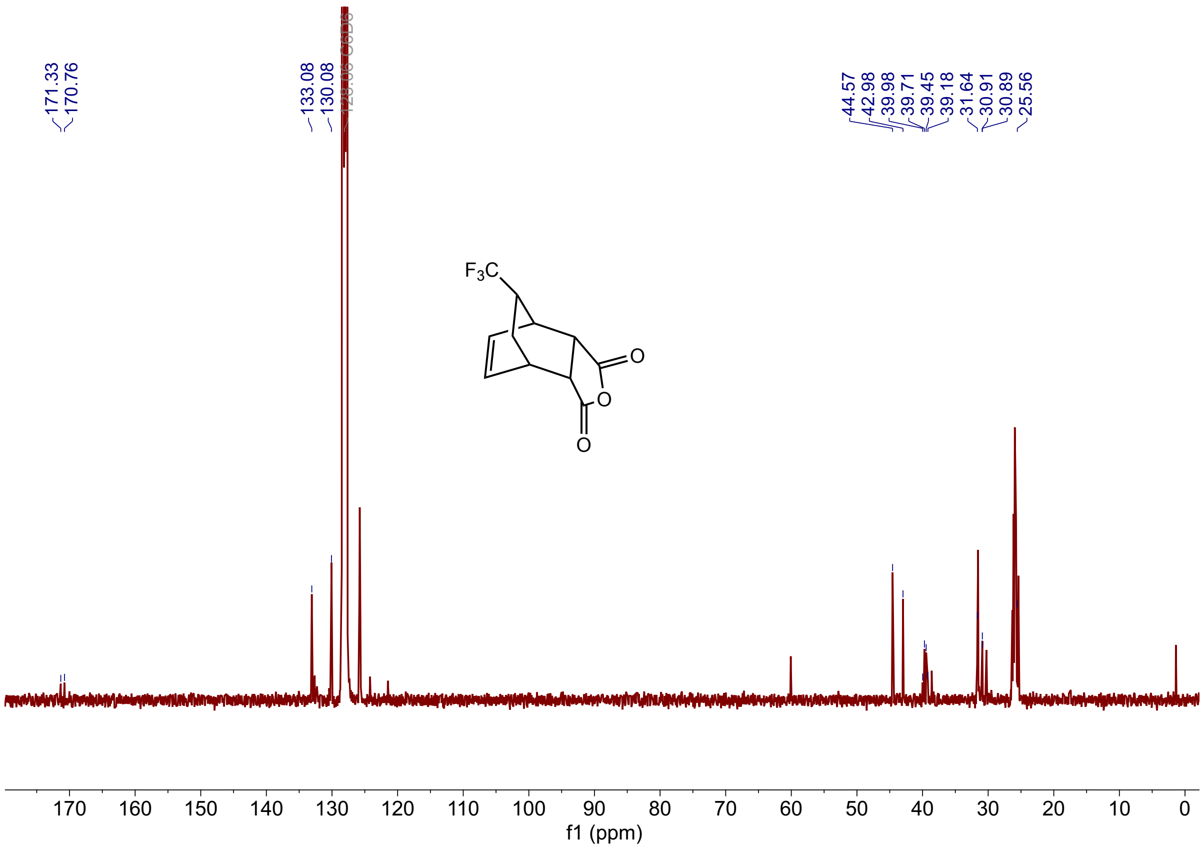

Figure S191. $^{13}C\{^1H\}$ NMR spectrum (101 MHz, benzene-d_6, 23 °C) of isolated **2** with 1,3,5-tris(trifluoromethyl)benzene internal standard in cyclohexane-d_{12} solution.

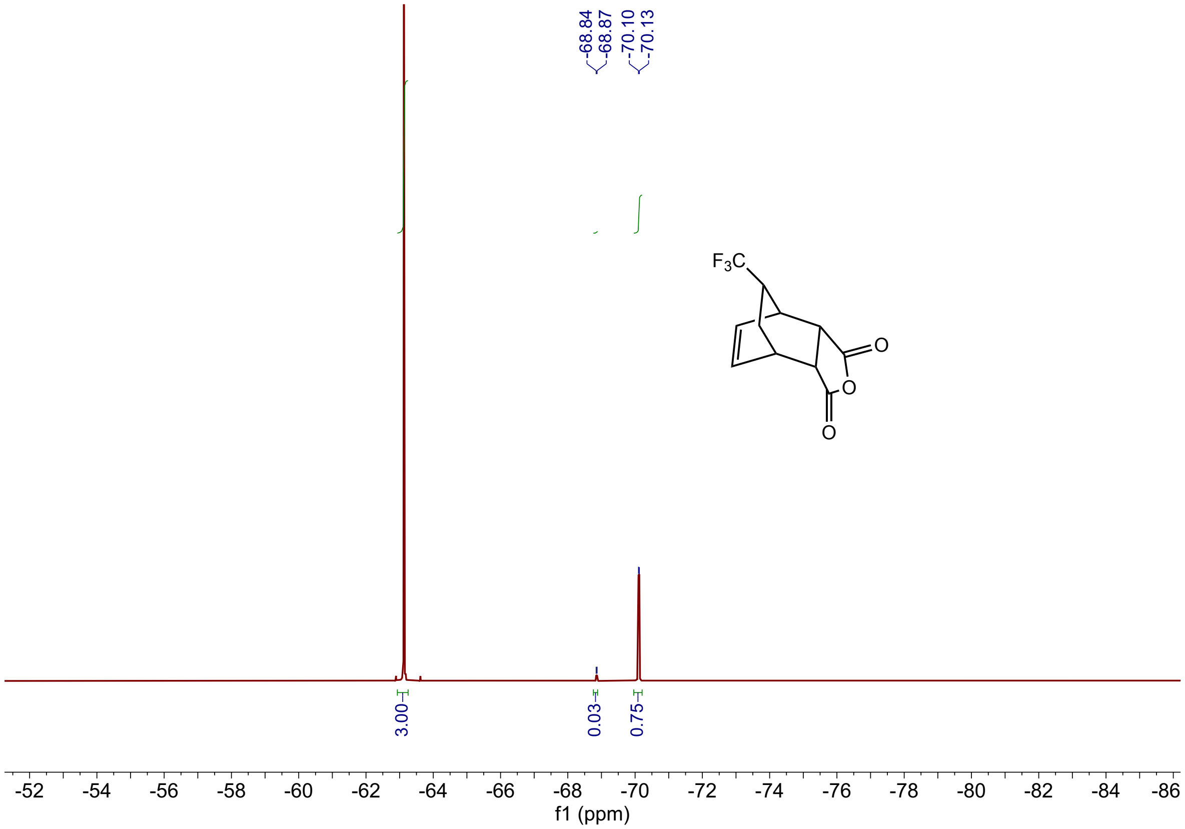

Figure S192. ^{19}F NMR spectrum (376 MHz, benzene-d_6, 23 °C) of isolated **2** with 1,3,5-tris(trifluoromethyl)benzene internal standard (δ -63.13 ppm).

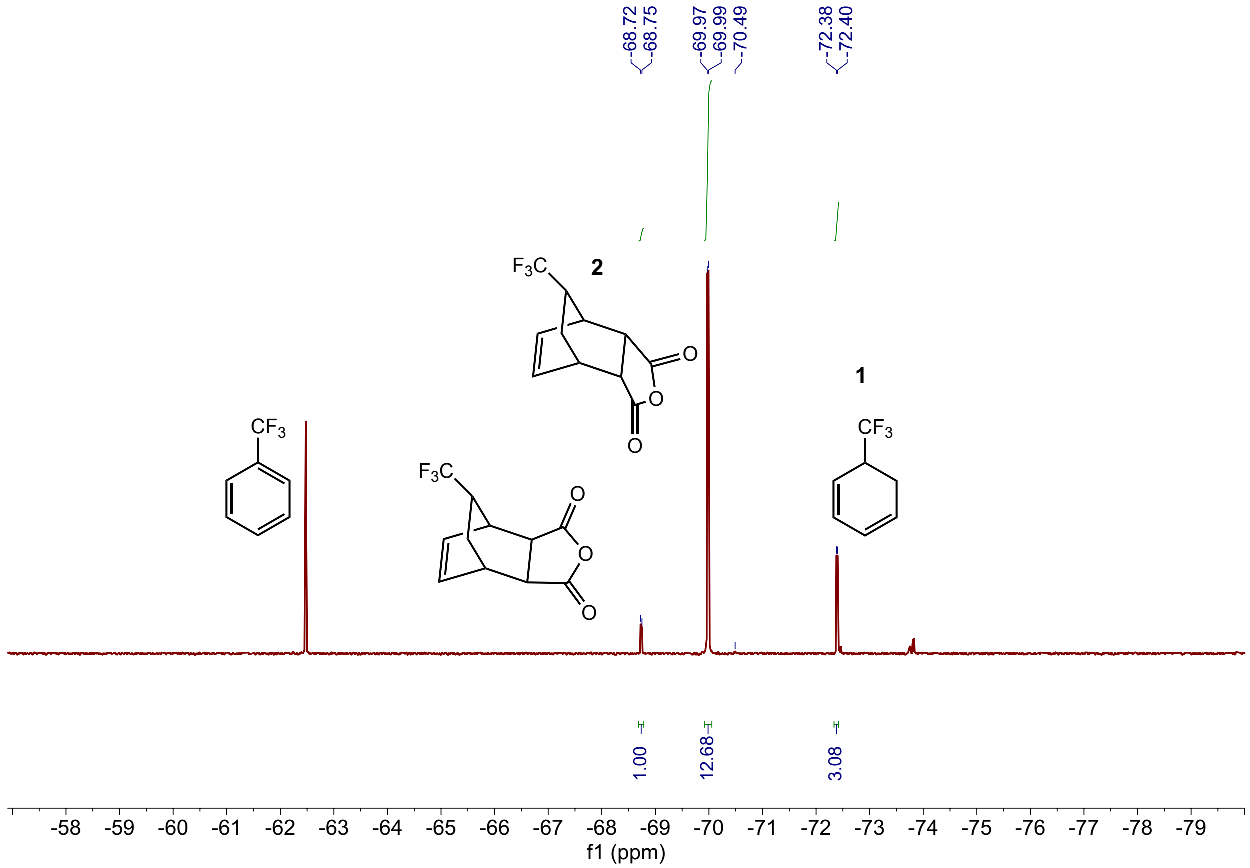

Figure S193. ^{19}F NMR spectrum (376 MHz, benzene-d_6, 23 °C) of conversion of **1** to **2**.

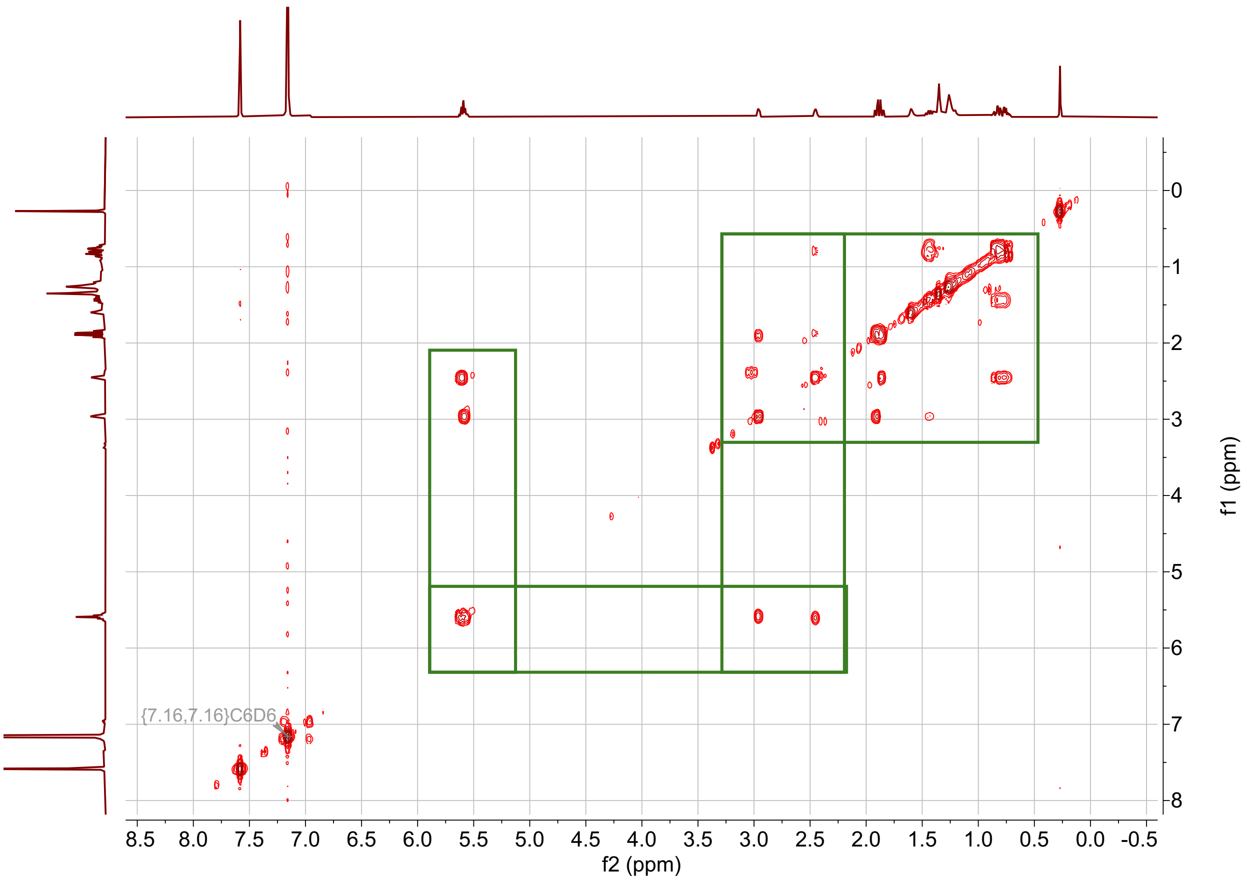

Figure S194. ^1H–^1H COSY NMR spectrum (benzene-d_6, 23 °C) of isolated 2 with 1,3,5-tris(trifluoromethyl)benzene. Inset: ^1H–^1H correlation between each signal.

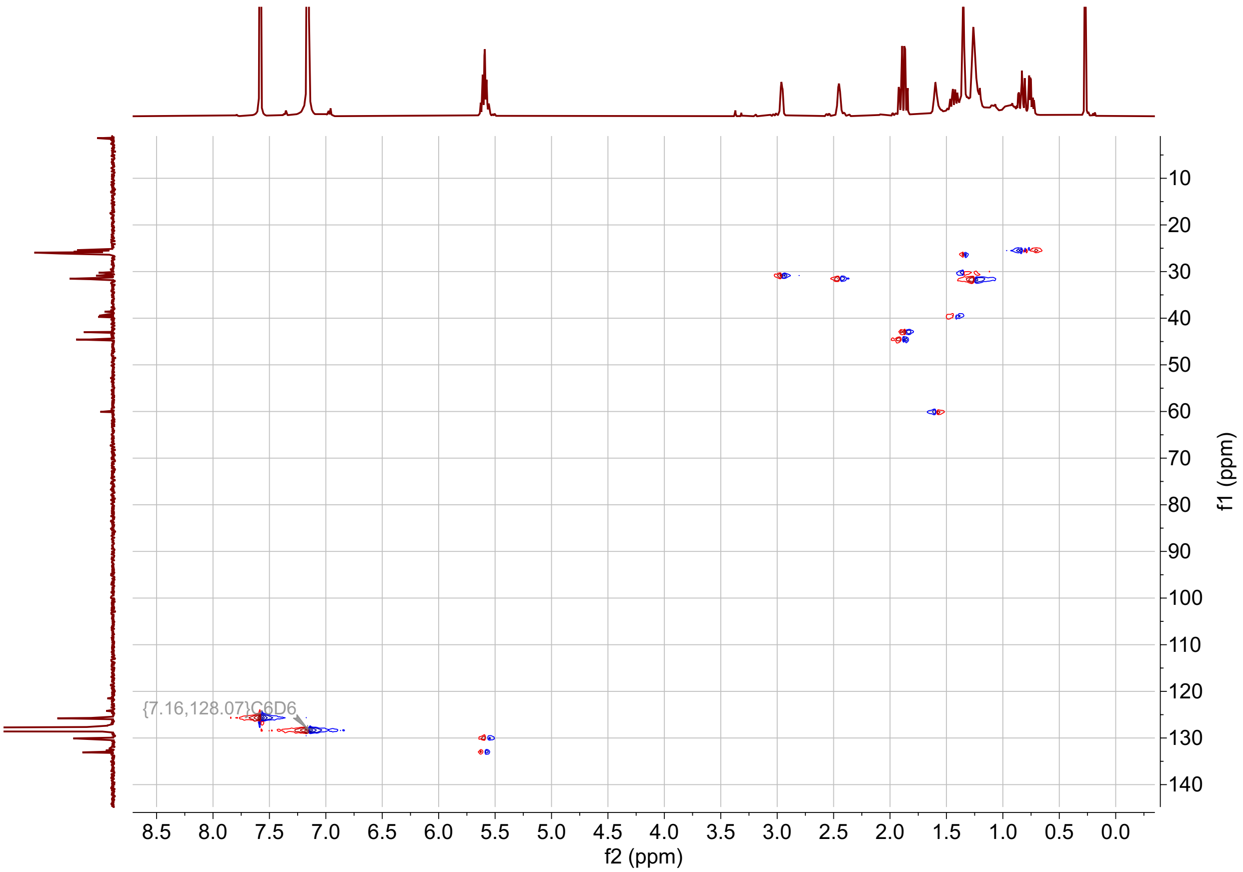

Figure S195. ^{1}H–$^{13}C\{^{1}H\}$ HSQC NMR spectrum (benzene-d_6, 23 °C) of isolated **2** with 1,3,5-tris(trifluoromethyl)benzene.

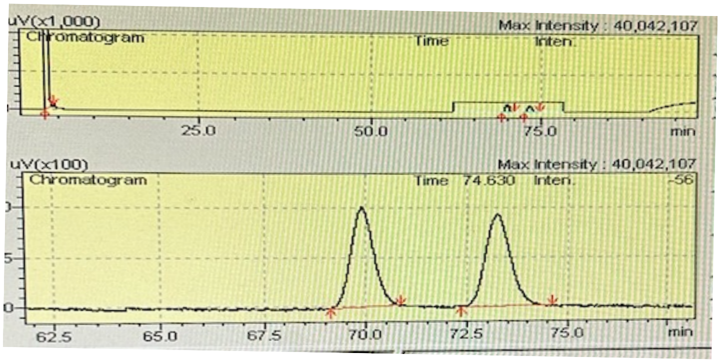

Peak#	Ret.Time	Area
1	3.276	383955669.5
2	69.883	19899.5
3	73.253	20949.8

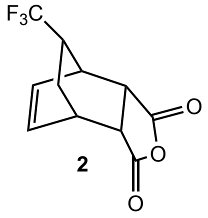

2

Figure S196. GC chromatogram of **2** generated from achiral dcype-supported **Co2**.

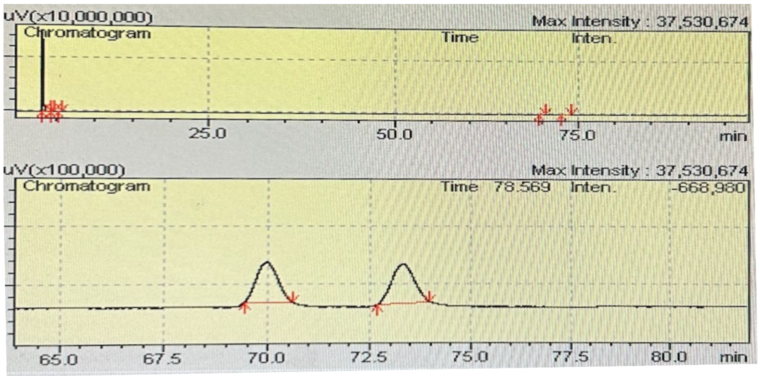

Peak#	Ret.Time	Area
1	3.277	353131459.5
2	4.601	12479.7
3	5.525	1191.9
4	69.968	24381.0
5	73.310	25698.8

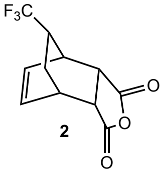

Figure S197. Chiral GC chromatogram of **2**. From (*R*,*R*)-^{iPr}DuPhos-supported **Co1**.

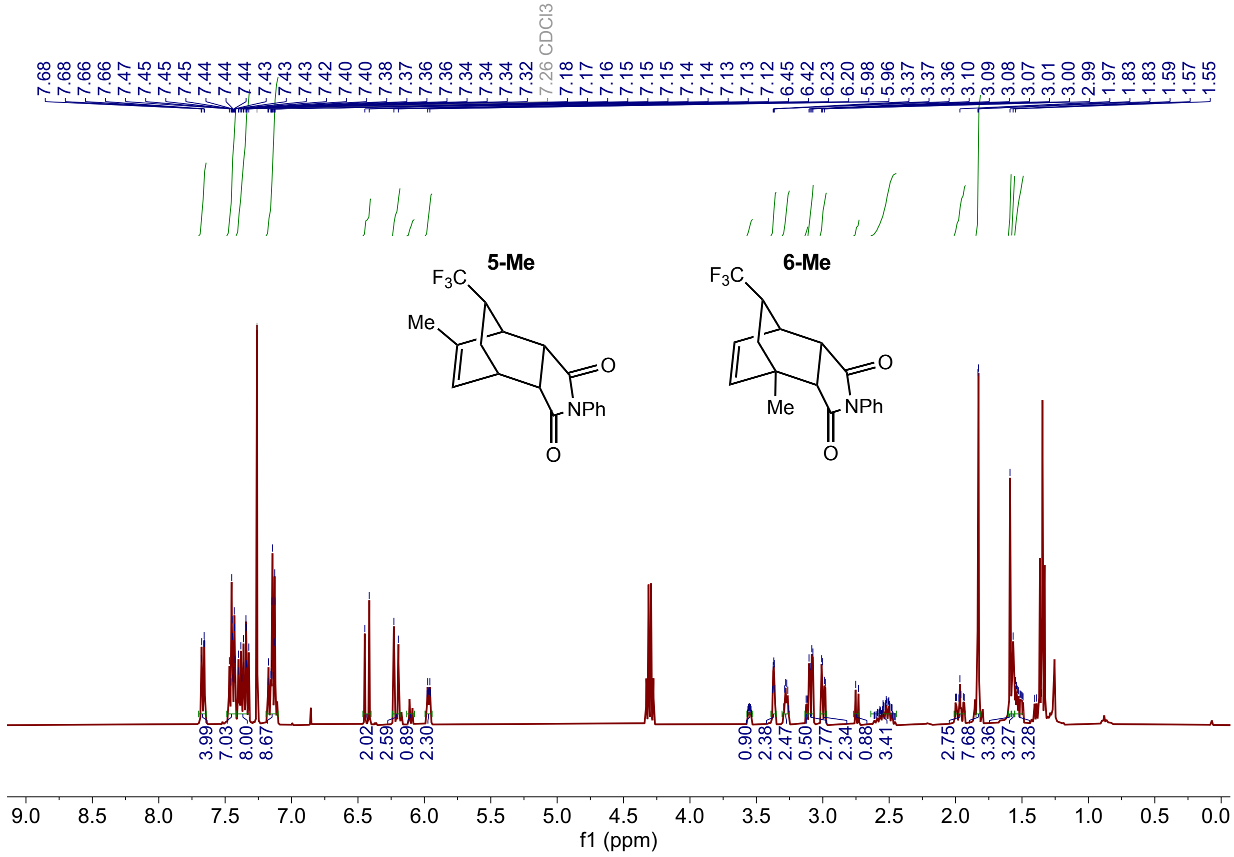

Figure S198. ^{1}H NMR spectrum (400 MHz, chloroform-*d*, 23 °C) of **5-Me** and **6-Me**.

Unassigned peaks associated with EtOAc.

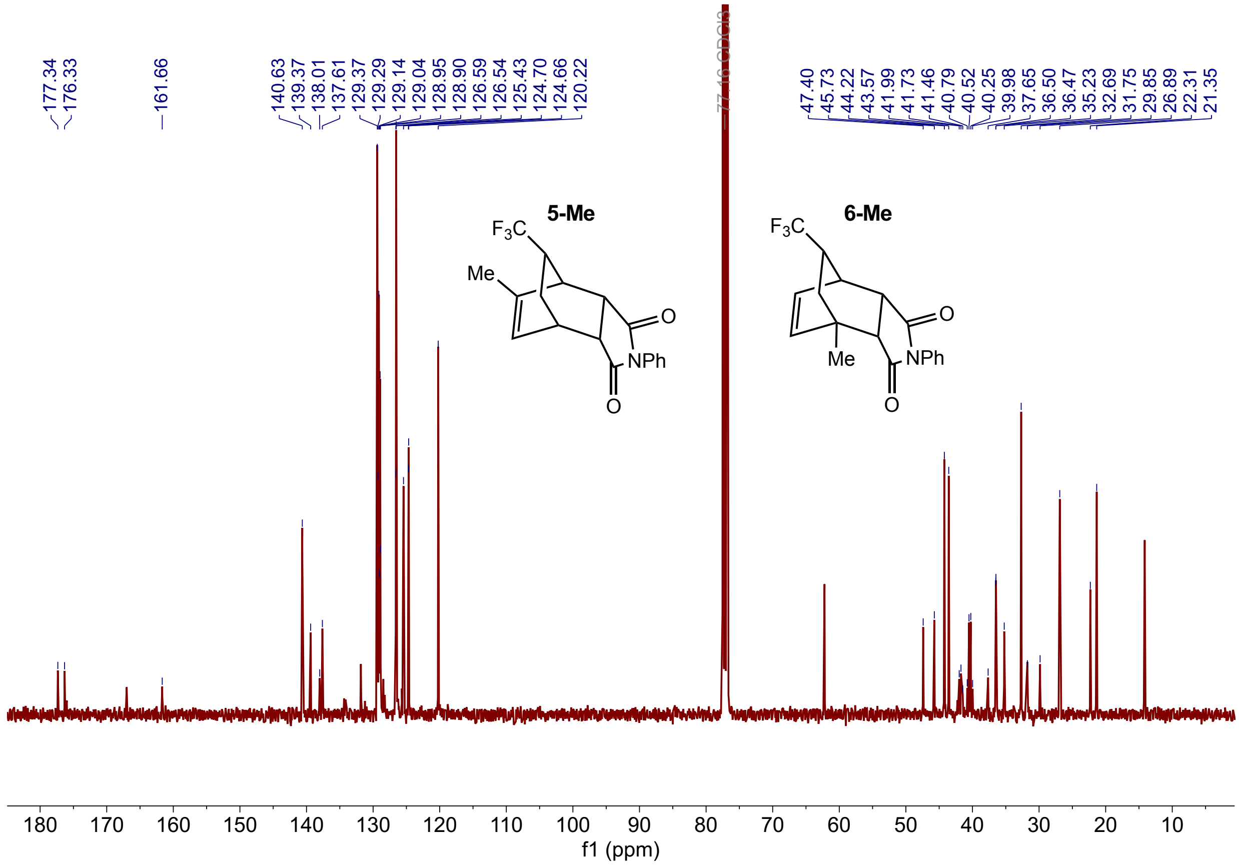

Figure S199. ^{13}C{^{1}H} NMR spectrum (101 MHz, chloroform-*d*, 23 °C) of **5-Me** and **6-Me**.

Unassigned peaks associated with EtOAc.

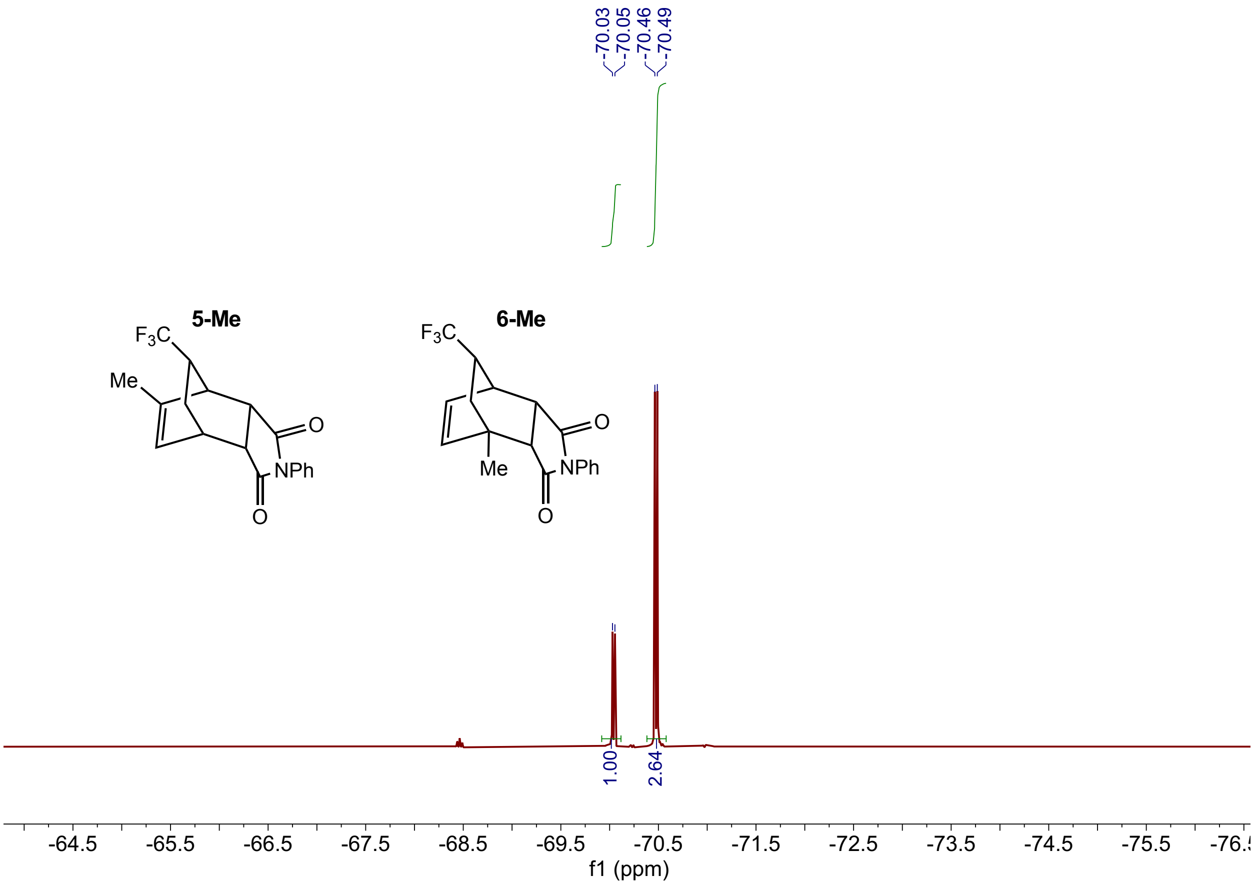

Figure S200. ^{19}F NMR spectrum (376 MHz, chloroform-*d*, 23 °C) of **5-Me** and **6-Me**.

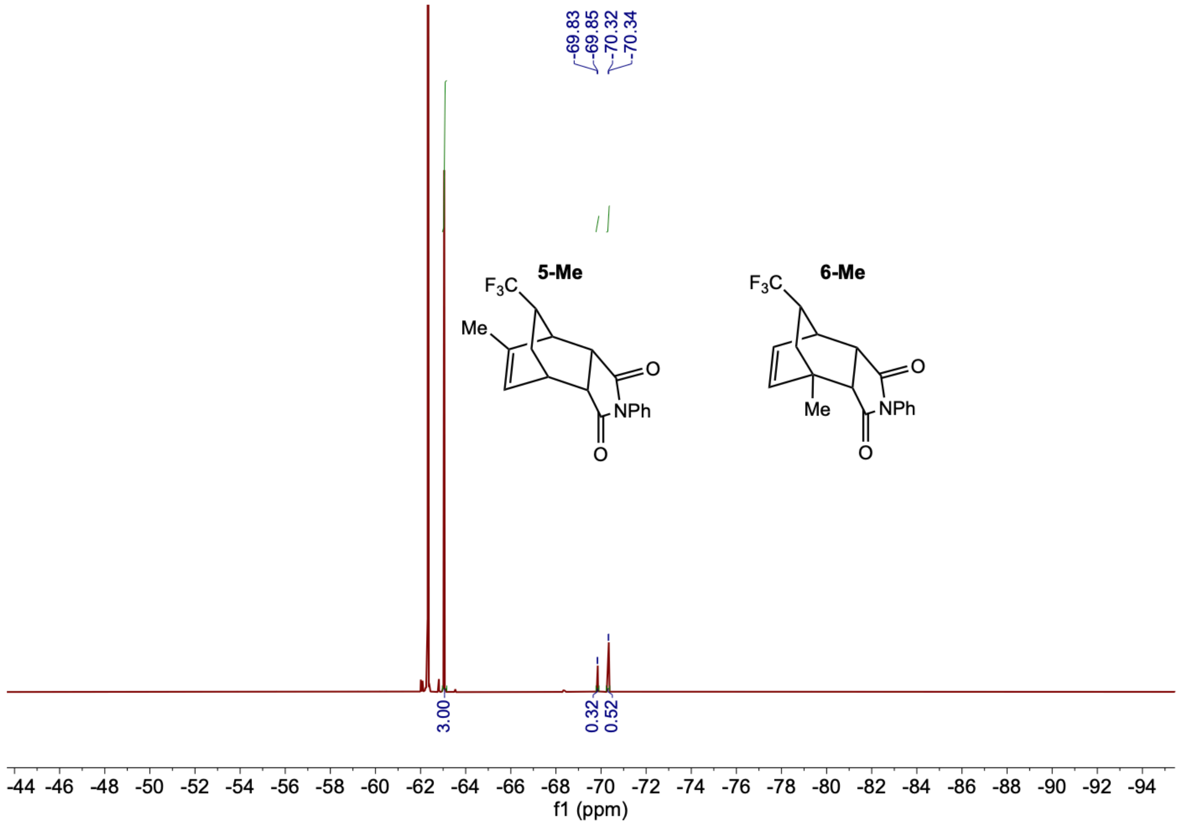

Figure S201. ^{19}F NMR spectrum (376 MHz, benzene-d_6, 23 °C) of **5-Me** and **6-Me** with 1,3,5-tris(trifluoromethyl)benzene internal standard (δ -63.05 ppm) and excess arene (δ -62.32 ppm).

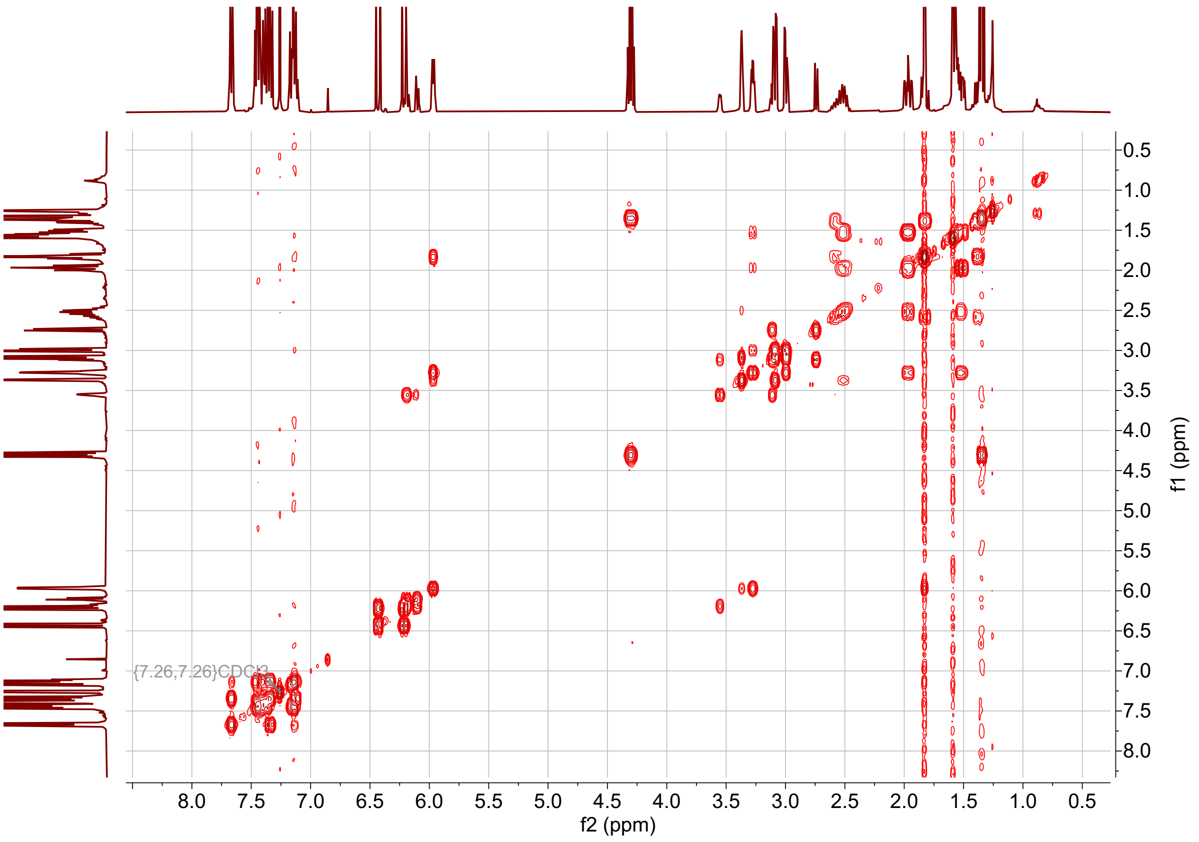

Figure S202. ^{1}H–^{1}H COSY NMR spectrum (chloroform-*d*, 23 °C) of **5-Me** and **6-Me**.

Unassigned resonances associated with EtOAc.

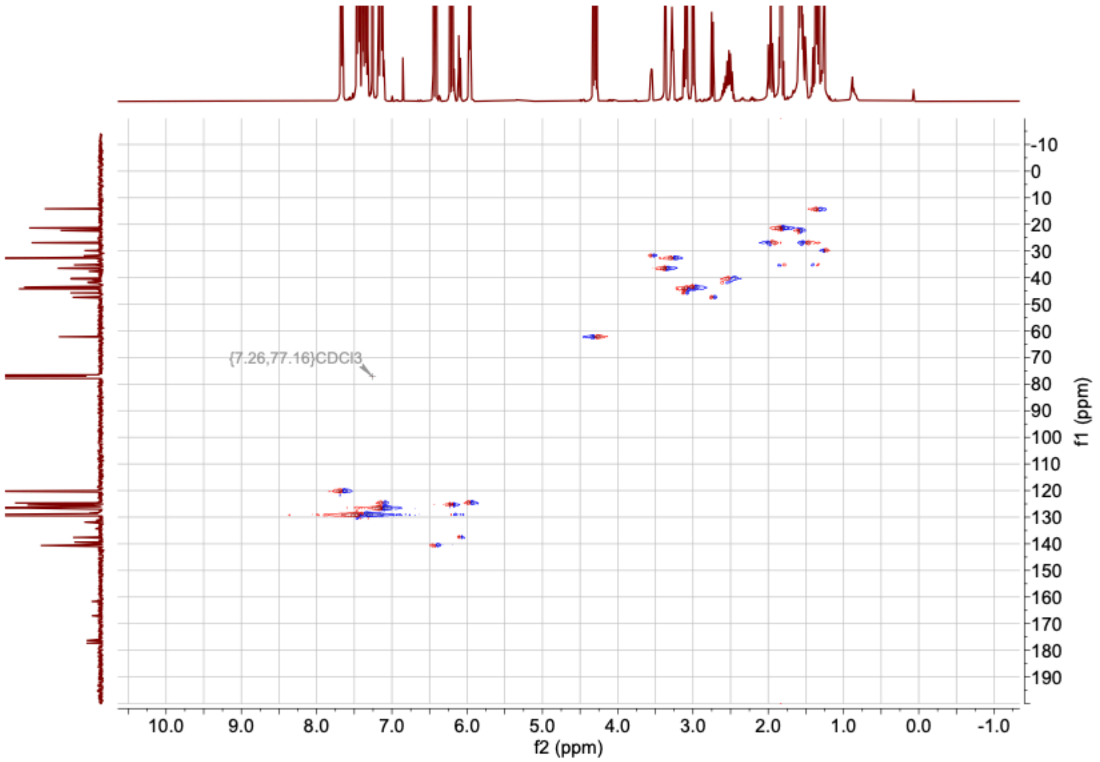

Figure S203. ^{1}H–$^{13}C\{^{1}H\}$ HSQC NMR spectrum (chloroform-*d*, 23 °C) of **5-Me** and **6-Me.**

Unassigned resonances associated with EtOAc.

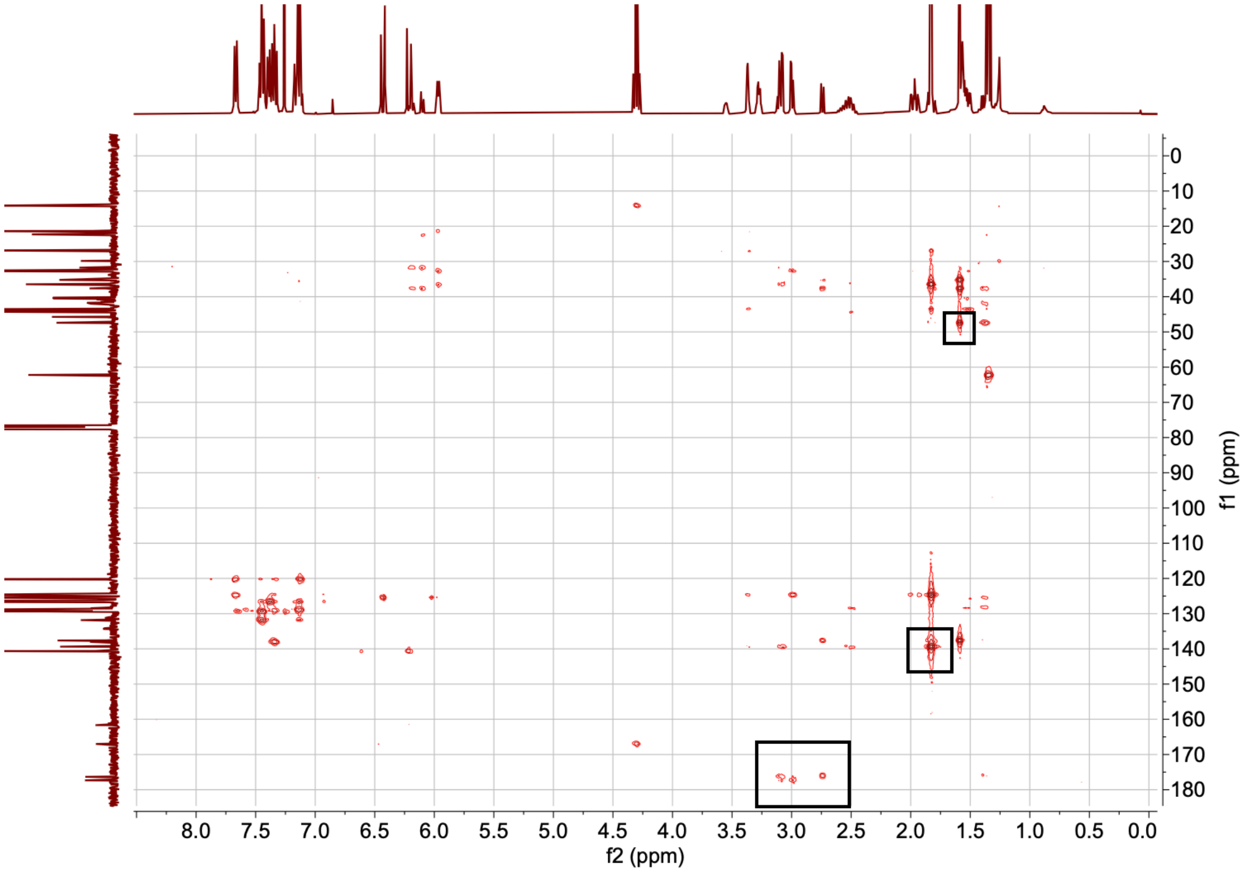

Figure S204. ^{1}H–$^{13}C\{^{1}H\}$ HMBC NMR spectrum (chloroform-*d*, 23 °C) of **5-Me** and **6-Me.**

Unassigned resonances associated with EtOAc.

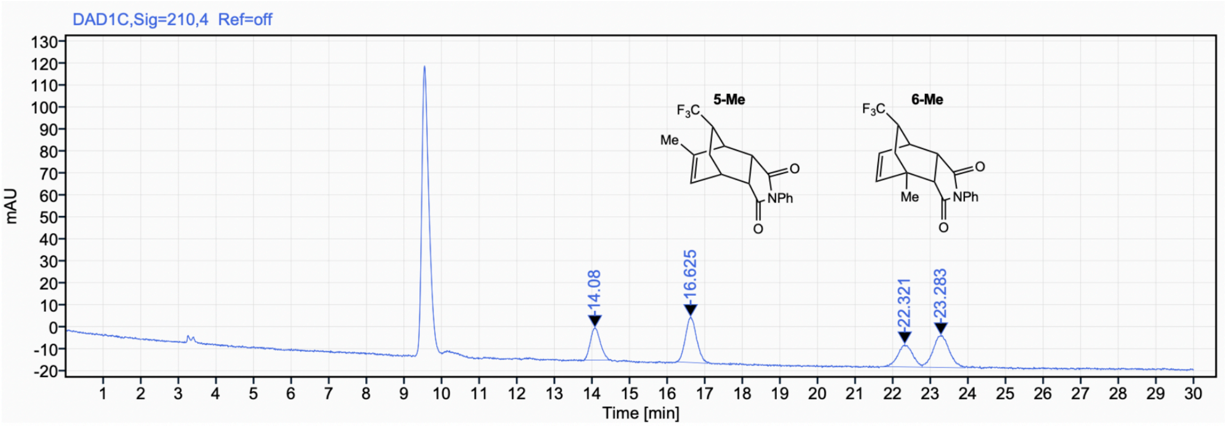

Signal:	DAD1C,Sig=210,4 Ref=off				
**RT [min]**	**Type**	**Width [min]**	**Area**	**Height**	**Area%**
14.080	MM m	0.67	270.28	14.63	19.25
16.625	MM m	0.82	423.51	20.67	30.17
22.321	MM m	1.03	275.27	10.27	19.61
23.283	MM m	1.14	434.64	14.63	30.96

Figure S205. HPLC chromatogram of racemic **5-Me** and **6-Me** (Chiralpak AD-H (250 x 4.6mm, 5μm); 10% IPA/ 90% hexane; 210 nm). Peak 1 + 3: **6-Me**; Peak 2 + 4 – **5-Me.** Unassigned signal corresponds to N-phenyl maleimide.

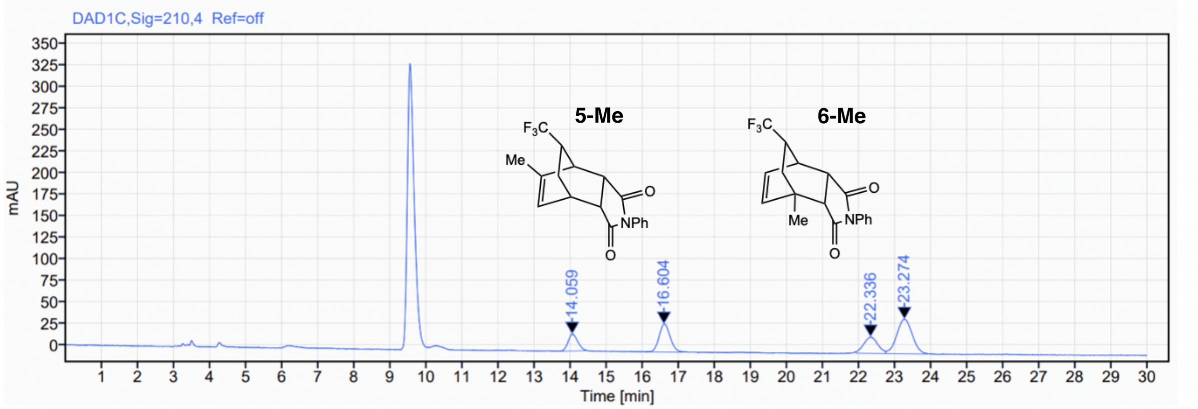

Signal: DAD1C,Sig=210,4 Ref=off

RT [min]	Type	Width [min]	Area	Height	Area%
14.059	MM m	0.80	366.68	19.62	13.24
16.604	BV R	0.99	699.09	32.56	25.24
22.336	VM m	0.83	510.41	19.04	18.43
23.274	MB m	1.15	1193.92	40.05	43.10

Figure S206. HPLC chromatogram of enantioenriched **5-Me** and **6-Me** (Chiralpak AD-H (250 x 4.6mm, 5µm); 10% IPA/ 90% hexane; 210 nm). Peak 1 + 3: **6-Me**; Peak 2 + 4 – **5-Me.** Unassigned signal corresponds to N-phenyl maleimide.

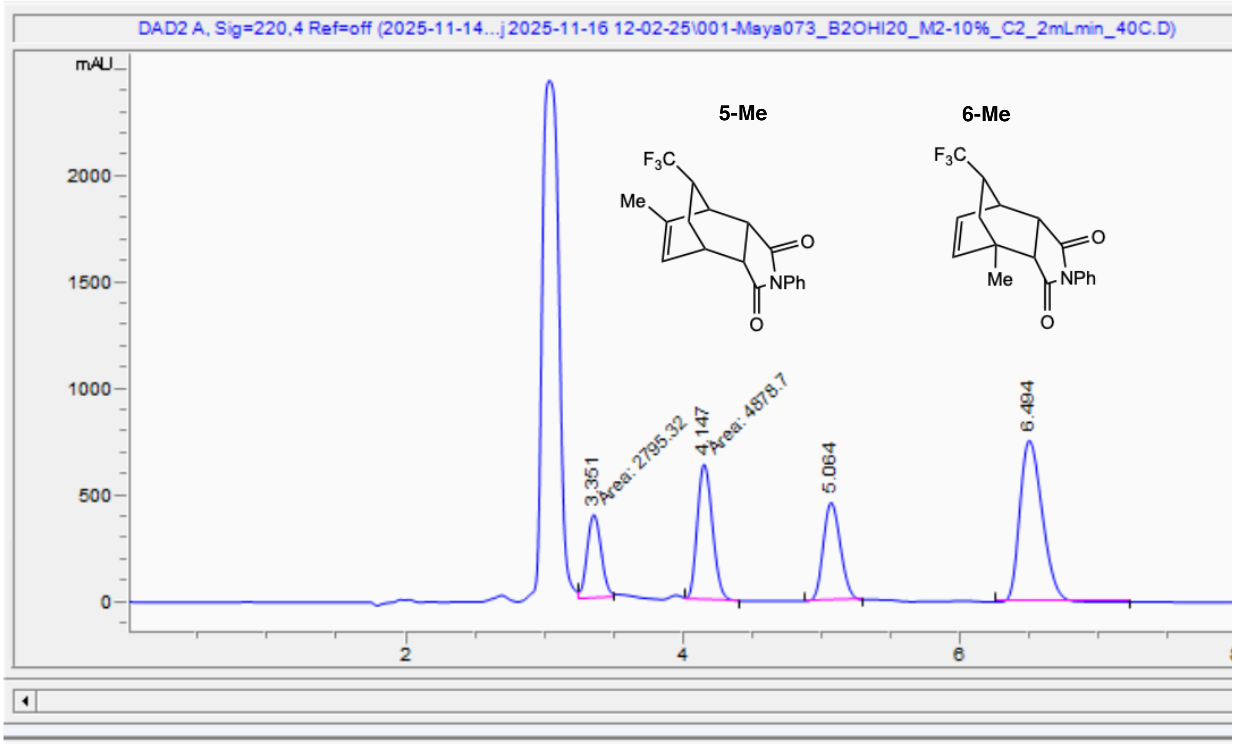

#	Time	Type	Area	Height	Width	Area%	Symmetry
1	3.351	MM	2795.3	392.6	0.1187	13.828	0.855
2	4.147	MM	4878.7	637.3	0.1276	24.134	0.804
3	5.064	BB	3952.1	458.9	0.1365	19.550	0.813
4	6.494	BB	8588.8	757.9	0.1782	42.487	0.69

Figure S207. SFC chromatogram of enantioenriched **5-Me** and **6-Me** (Enantiocel A3-5 (250 x 4.6 mm) 2 mL/min; 10% ethanol / 90% CO2 (100 bar); 220 nm). Peak 1 + 3: **6-Me**; Peak 2 + 4 – **5-Me.** Unassigned signal corresponds to N-phenyl maleimide.

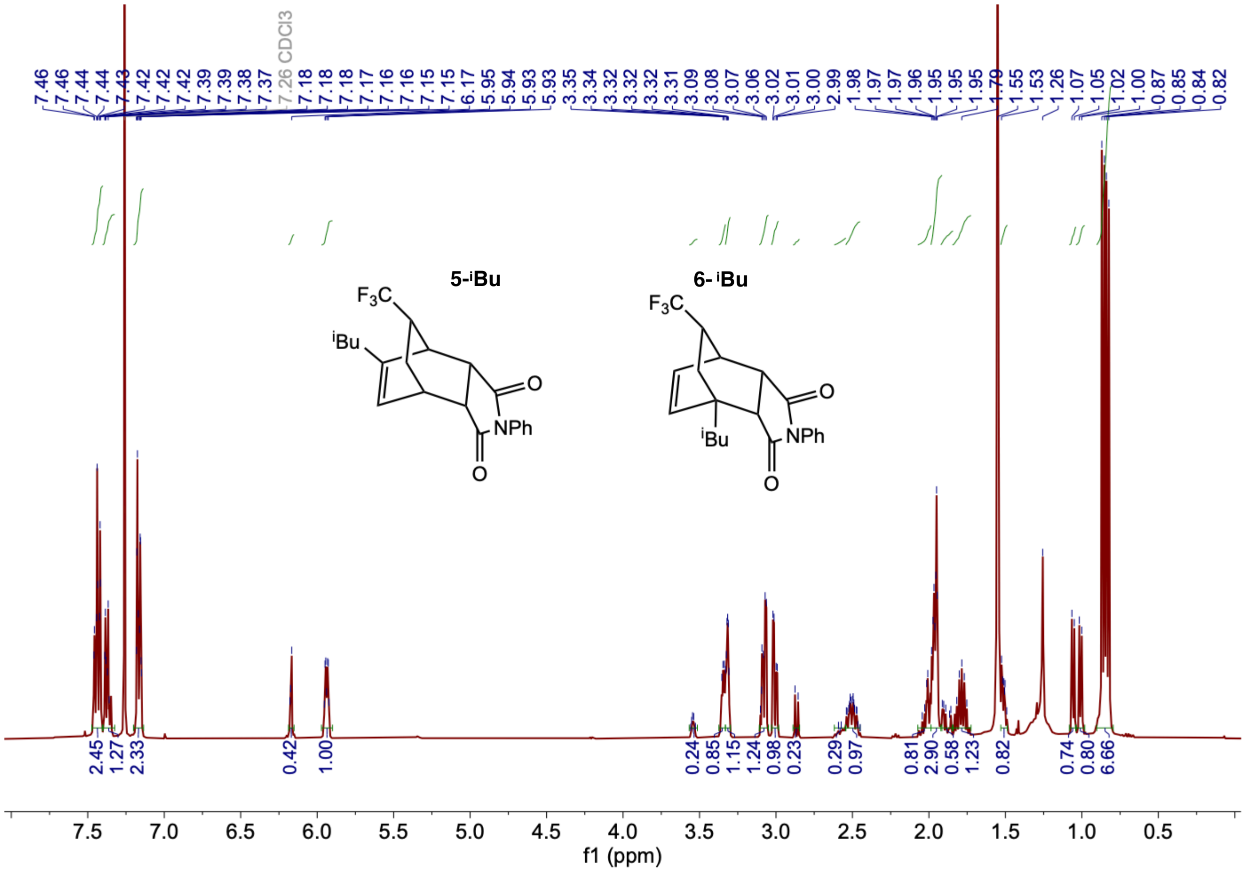

Figure S208. ^{1}H NMR spectrum (400 MHz, chloroform-*d*, 23 °C) of **5-^{i}Bu** and **6-^{i}Bu**.

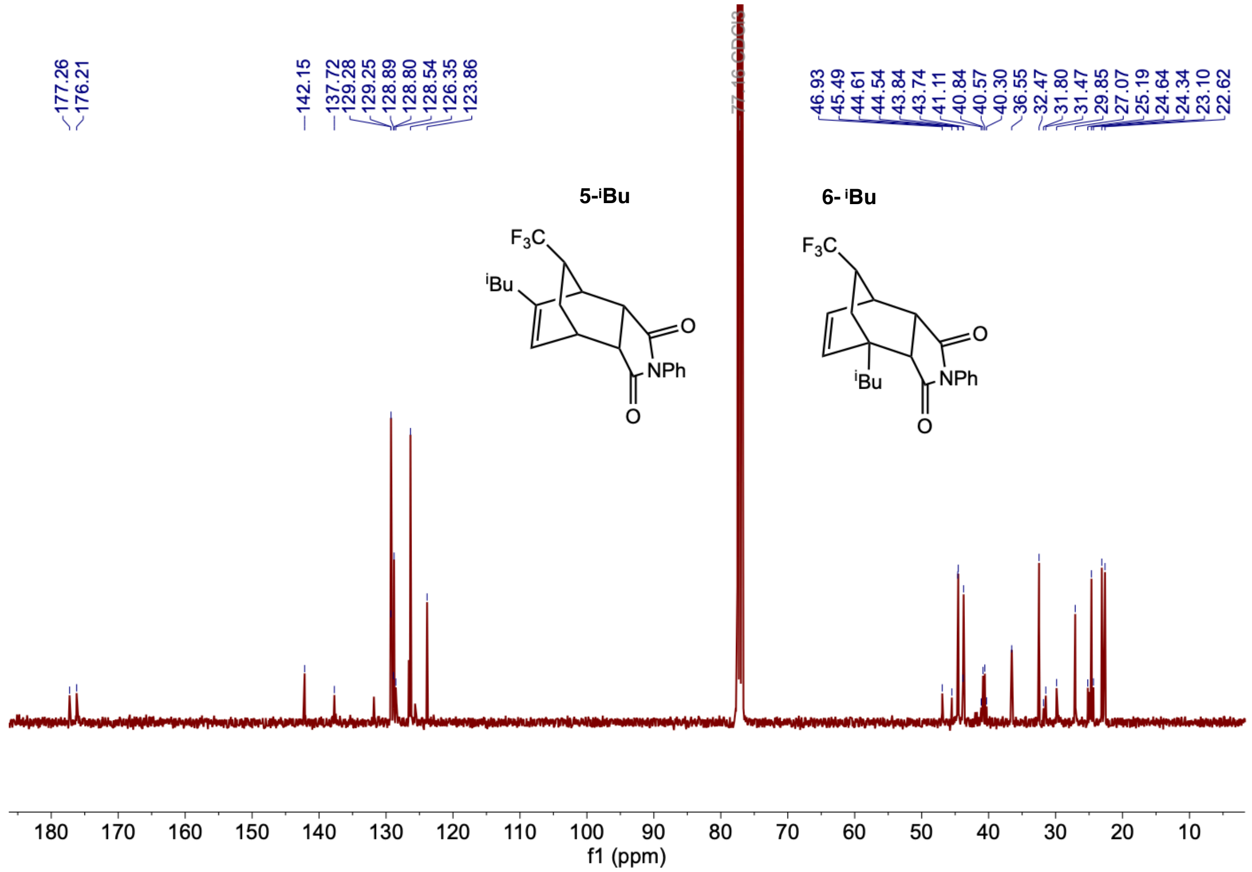

Figure S209. $^{13}C\{^{1}H\}$ NMR spectrum (101 MHz, chloroform-*d*, 23 °C) of **5-^{i}Bu** and **6-^{i}Bu**.

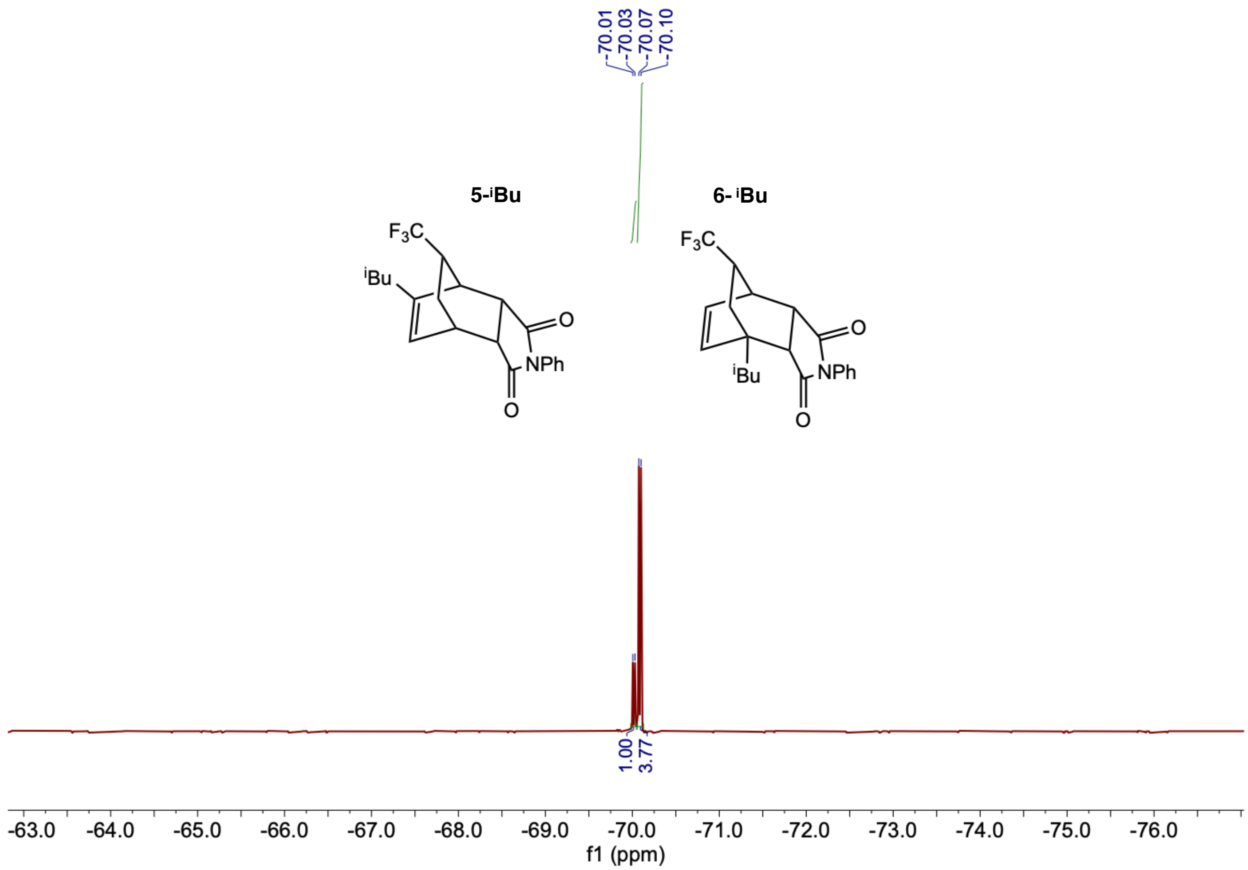

Figure S210. ^{19}F NMR spectrum (376 MHz, chloroform-*d*, 23 °C) of **5-^{i}Bu** and **6-^{i}Bu**.

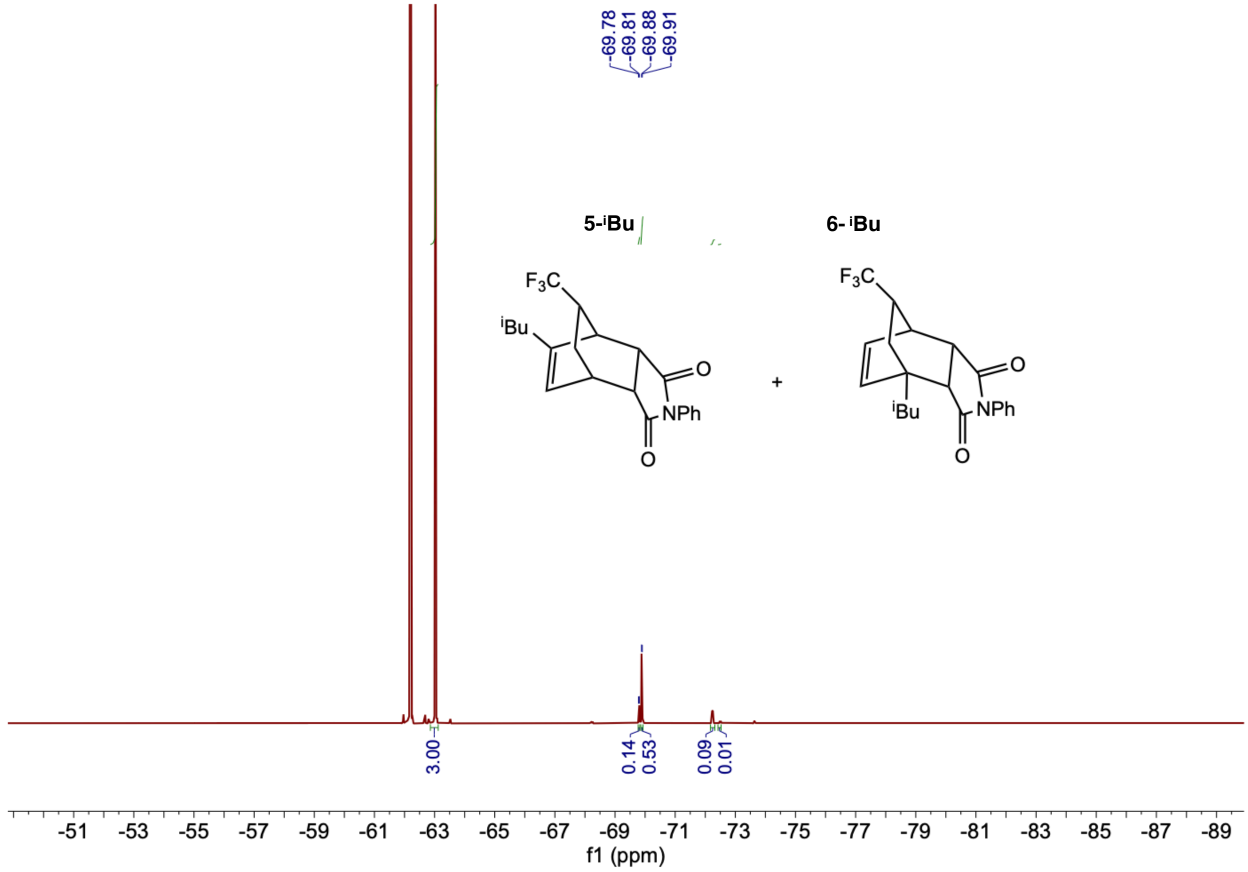

Figure S211. ^{19}F NMR spectrum (376 MHz, benzene-d_6, 23 °C) of **5-^{i}Bu** and **6-^{i}Bu** with 1,3,5-tris(trifluoromethyl)benzene internal standard (δ -63.03 ppm) and excess arene (δ -62.20 ppm).

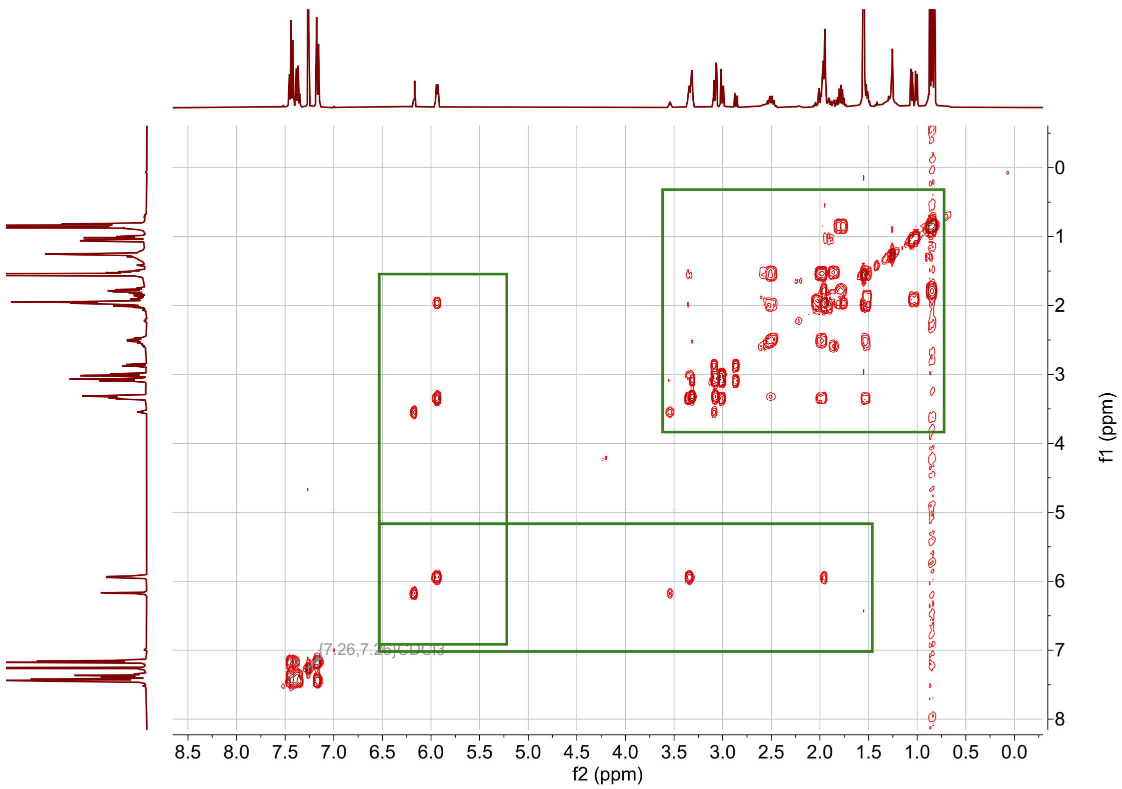

Figure S212. ^{1}H–^{1}H COSY NMR spectrum (chloroform-*d*, 23 °C) of **5-^{i}Bu** and **6-^{i}Bu.** Inset: ^{1}H–^{1}H correlation between each signal.

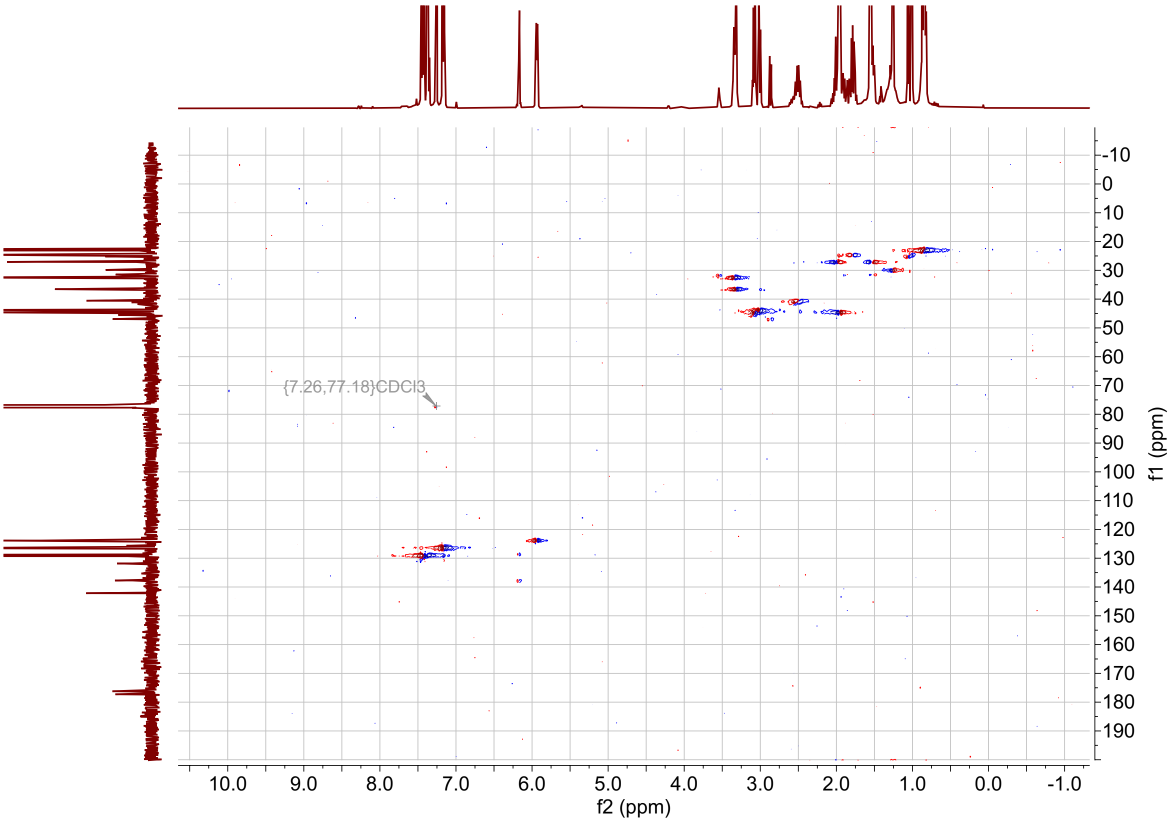

Figure S213. ^{1}H–$^{13}C\{^{1}H\}$ HSQC NMR spectrum (chloroform-*d*, 23 °C) of **5-^{i}Bu** and **6-^{i}Bu.**

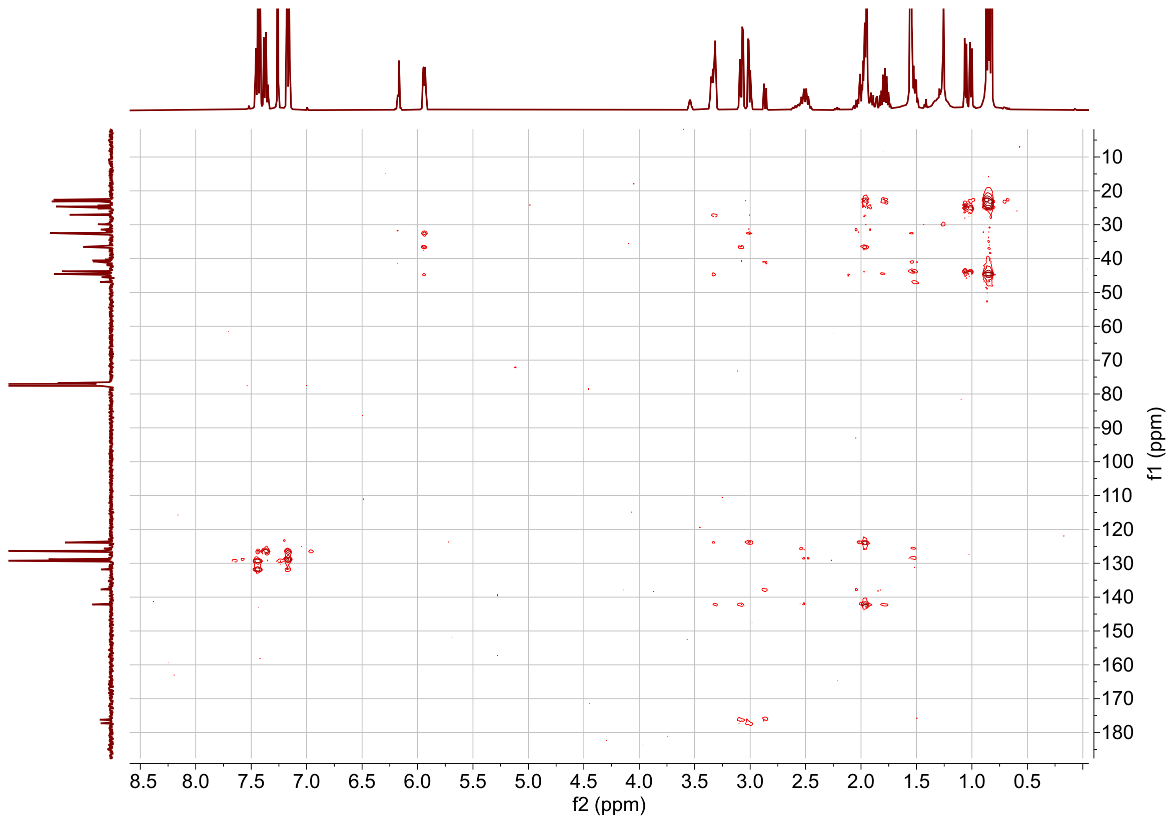

Figure S214. $^{1}H–^{13}C\{^{1}H\}$ HMBC NMR spectrum (chloroform-*d*, 23 °C) of **5-^{i}Bu** and **6-^{i}Bu.**

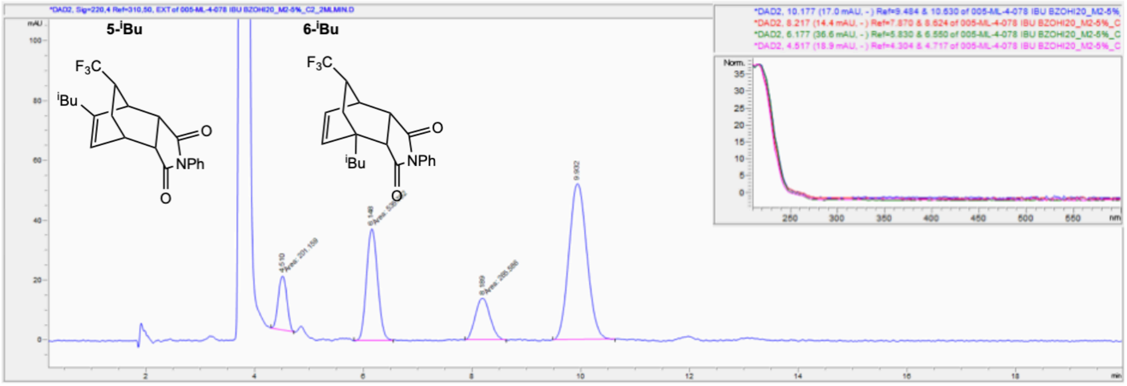

#	Time	Type	Area	Height	Width	Area%
1	4.51	MM	201.2	18.1	0.1855	9.144
2	6.148	MM	535.8	37.3	0.2391	24.353
3	8.189	MM	265.6	13.9	0.3184	12.072
4	9.932	BB	1197.5	52	0.3445	54.432

Instrument: Agilent 1260 SFC ("Aurora-2")
Column: Daicel ChiralPak AS-H (250 x 4.6 mm)
SFC Method: 2.0 mL/min; 5% ethanol / 95% CO_2 (100 bar)

Signal: DAD1C,Sig=210,4 Ref=off

RT [min]	Type	Width [min]	Area	Height	Area%
14.059	MM m	0.80	366.68	19.62	13.24
16.604	BV R	0.99	699.09	32.56	25.24
22.336	VM m	0.83	510.41	19.04	18.43
23.274	MB m	1.15	1193.92	40.05	43.10

Figure S215. SFC chromatogram of enantioenriched **5-^{i}Bu** and **6-^{i}Bu** (lDaicel ChiralPak AS-H 5% EtOH). Peak 2+ 4: **5-^{i}Bu**; Peak 1 + 3 – **6-^{i}Bu**. Unassigned signal corresponds to N-phenyl maleimide.

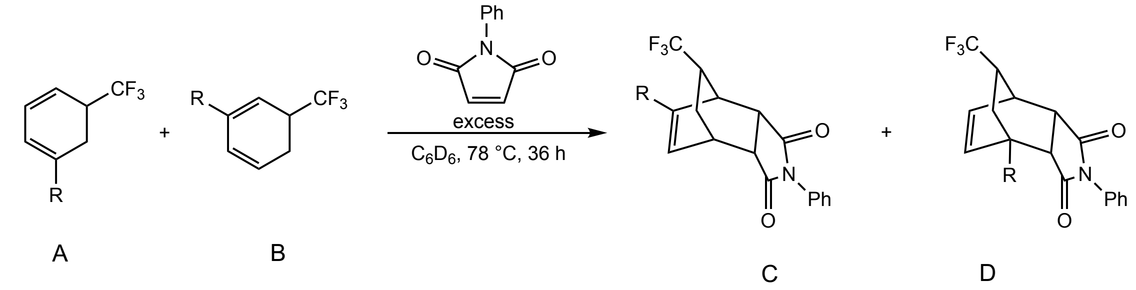

H^+ =	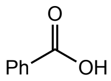	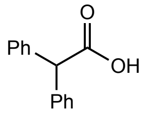	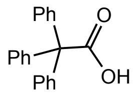	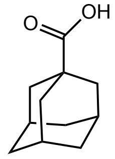
R = Me; % yield (C:D: combined)	59:35	67:31	74:18:92	59:33
R = Me; % ee (C:D)	27:18	25:16	18:23	11:10
R = ^{i}Bu; % yield (C:D: combined)	65:18	68:22	-	69:11:80
R = ^{i}Bu; % ee (C:D)	38:14	33:3	-	1:20

Figure S216. Yield and Enantiomeric Excess (ee) of **5-Me**, **6-Me**, **5-^{i}Bu**, and **6-^{i}Bu** using different carboxylic acids determined by ^{19}F NMR spectroscopy and SFC.

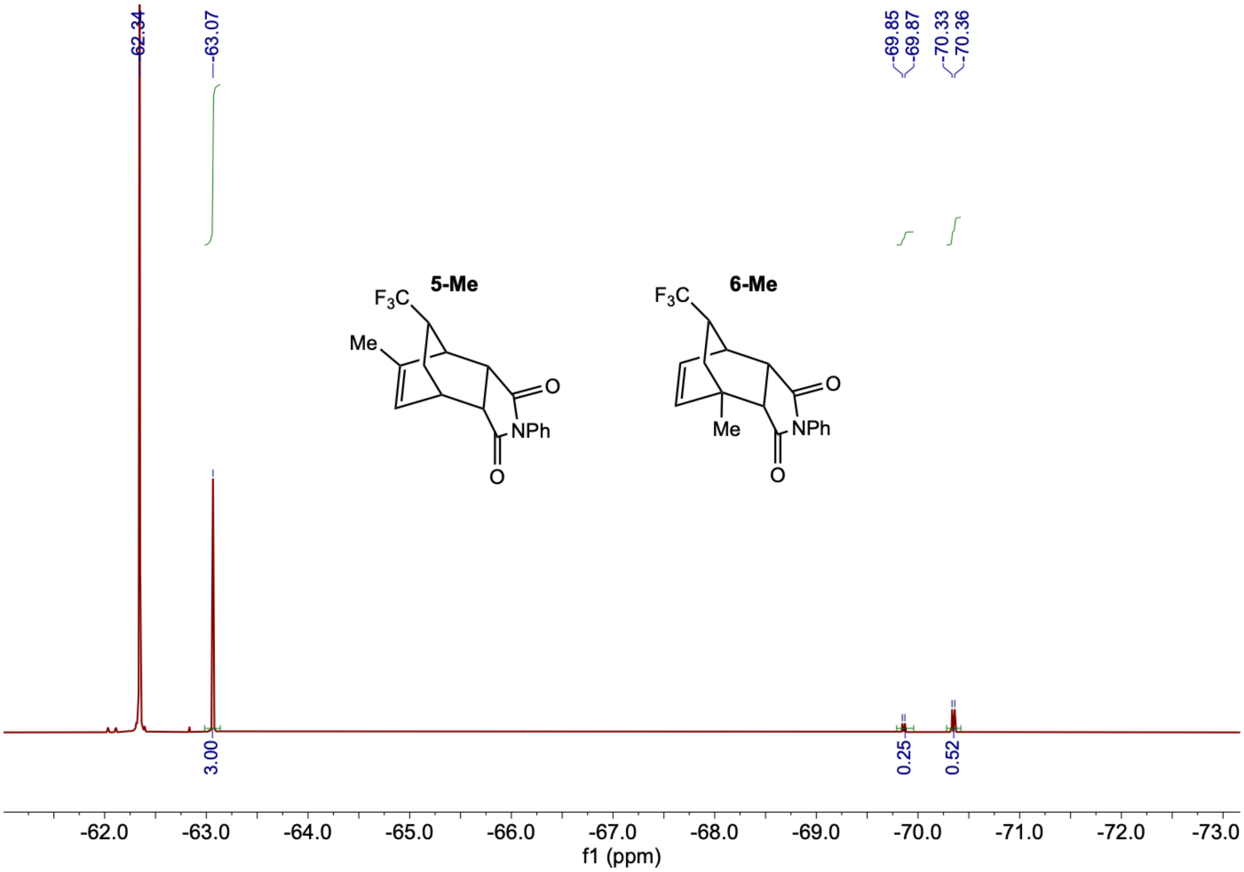

Figure S217. ^{19}F NMR spectrum (376 MHz, benzene-d_6, 23 °C) of **5-Me** and **6-Me** synthesized by general procedure A (0.013 mmol **3-Me**, 0.0065 mmol **4-Me**, 0.066 mmol 2,2-diphenyl acetic acid) with 1,3,5-tris(trifluoromethyl)benzene internal standard (δ -63.07 ppm) and excess arene (δ -62.34 ppm).

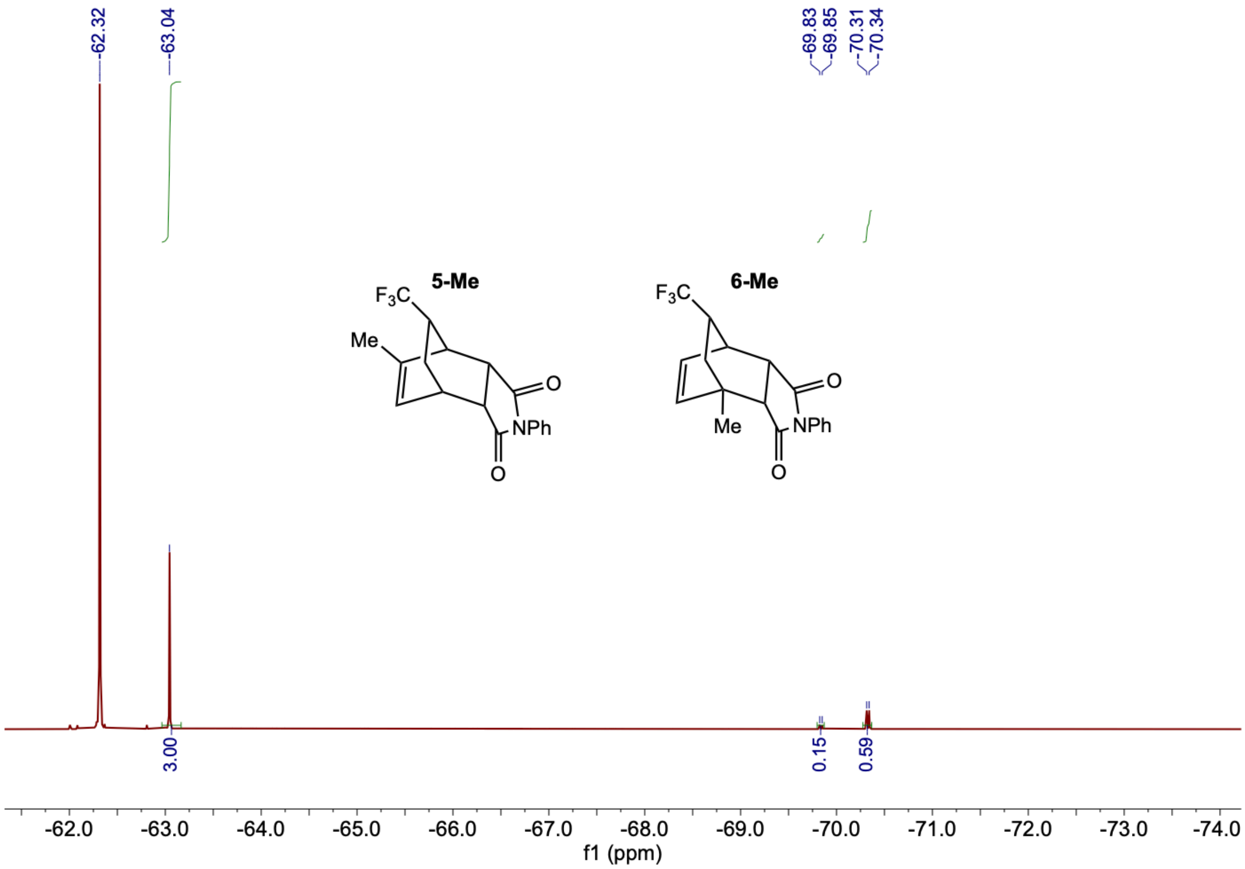

Figure S218. ^{19}F NMR spectrum (376 MHz, benzene-d_6, 23 °C) of **5-Me** and **6-Me** synthesized by general procedure A (0.0162 mmol **3-Me**, 0.004 mmol **4-Me**, 0.0694 mmol 2,2,2-triphenylacetic acid) with 1,3,5-tris(trifluoromethyl)benzene internal standard (δ -63.04 ppm) and excess arene (δ -62.32 ppm).

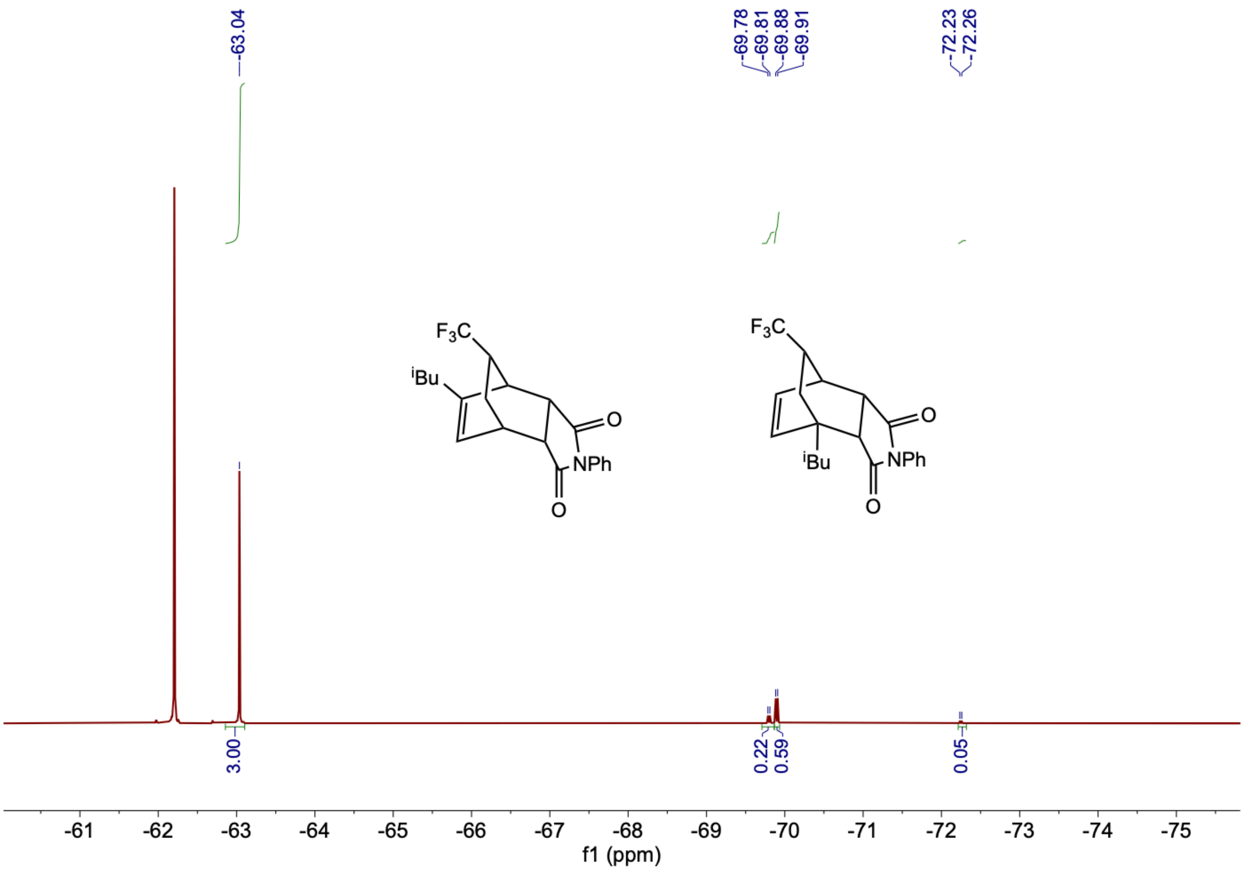

Figure S219. ^{19}F NMR spectrum (376 MHz, benzene-d_6, 23 °C) of **5-^{i}Bu** and **6-^{i}Bu** synthesized by general procedure A (0.0160 mmol **3-^{i}Bu**, 0.006 mmol **4-^{i}Bu**, 0.080 mmol 2,2-diphenylacetic acid) with 1,3,5-tris(trifluoromethyl)benzene internal standard (δ -63.04 ppm) and excess arene (δ -62.21 ppm).

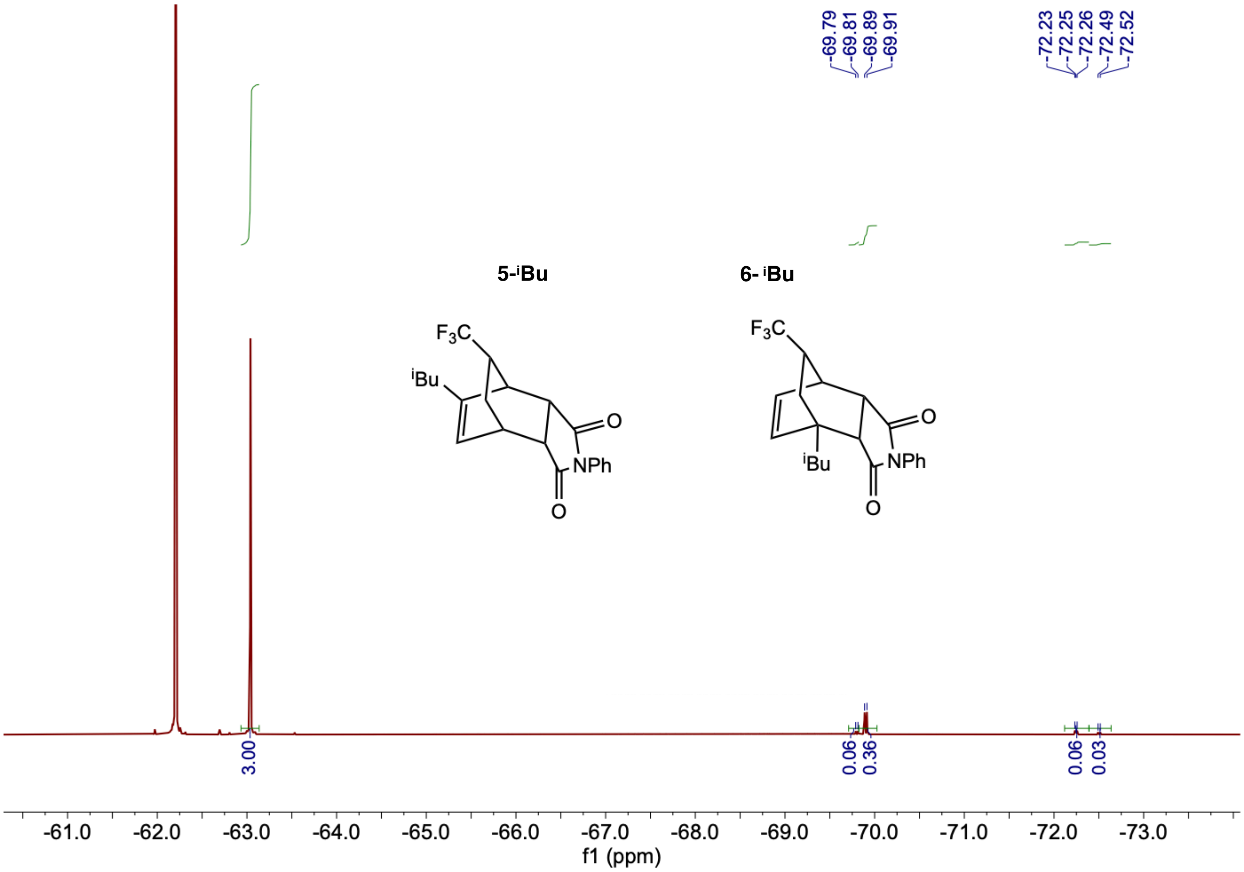

Figure S220. ^{19}F NMR spectrum (376 MHz, benzene-d_6, 23 °C) of **5-^{i}Bu** and **6-^{i}Bu** synthesized by general procedure A (0.010 mmol **3- ^{i}Bu**, 0.003 mmol **4- ^{i}Bu**, 0.077 mmol 1-adamantanecarboxylic acid) with 1,3,5-tris(trifluoromethyl)benzene internal standard (δ -63.04 ppm) and excess arene (δ -62.21 ppm).

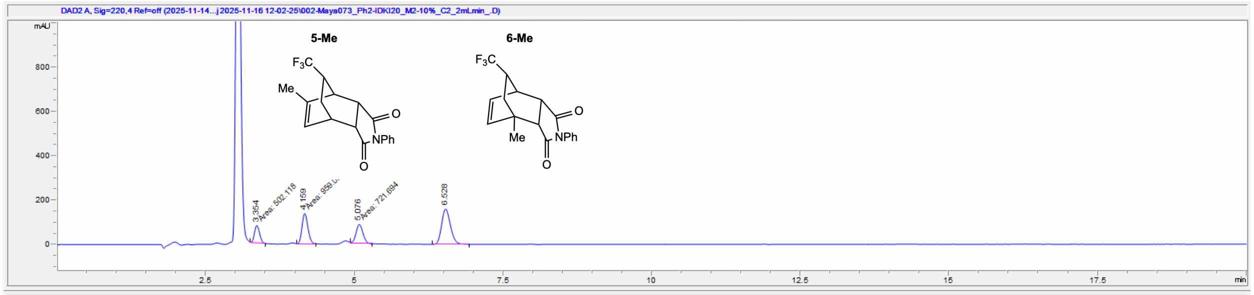

#	Time	Type	Area	Height	Width	Area%	Symmetry
1	3.354	MM T	502.1	80.5	0.104	13.122	0.815
2	4.159	MM	959.1	137	0.1166	25.064	0.814
3	5.076	MM	721.7	87.2	0.1379	18.861	0.87
4	6.528	BB	1643.6	158.7	0.1608	42.953	0.786

Figure S221. SFC chromatogram of corresponding to **Figure S217**. (Enantiocel A3-5 (250 x 4.6 mm) 2 mL/min; 10% ethanol / 90% CO_2 (100 bar); 220 nm). Peak 2+ 4: **5-Me**; Peak 1 + 3 – **6-Me.** Unassigned signal corresponds to N-phenyl maleimide.

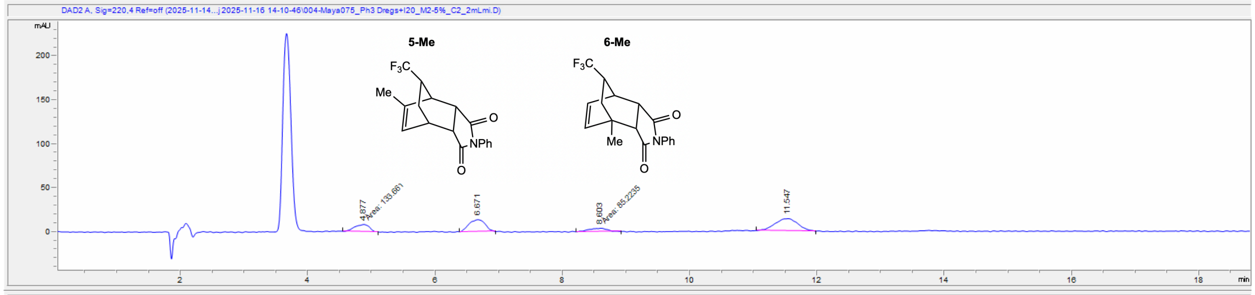

#	Time	Type	Area	Height	Width	Area%	Symmetry
1	4.877	MM	133.7	8.1	0.2734	16.273	1.62
2	6.671	BB	246.6	13.7	0.2693	30.018	1.187
3	8.603	MM	85.2	4.1	0.3498	10.376	1.707
4	11.547	BB	355.9	13.9	0.3218	43.333	1.325

Figure S222. SFC chromatogram of corresponding to **Figure S218**. (Enantiocel A3-5 (250 x 4.6 mm) 2 mL/min; 5% ethanol / 95% CO_2 (100 bar); 220 nm). Peak 2+ 4: **5-Me**; Peak 1 + 3 – **6-Me.** Unassigned signal corresponds to N-phenyl maleimide.

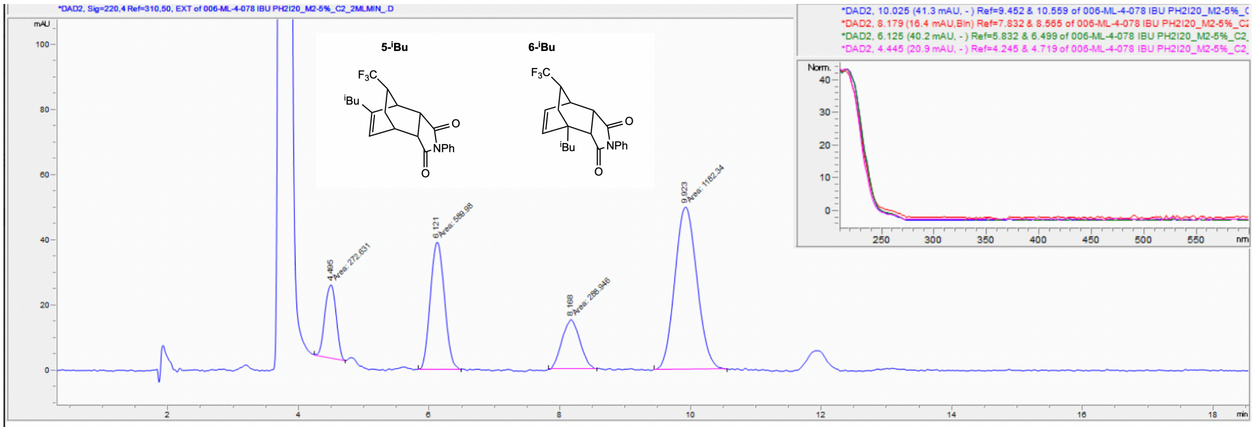

#	Time	Type	Area	Height	Width	Area%
1	4.495	MM	272.6	22.6	0.2011	11.681
2	6.121	MM	590	39.2	0.2511	25.279
3	8.168	MM	288.9	15.2	0.3168	12.380
4	9.923	MM	1182.3	49.9	0.3945	50.660

Instrument: Agilent 1260 SFC ("Aurora-2")
Column: Daicel ChiralPak AS-H (250 x 4.6 mm)
SFC Method: 2.0 mL/min; 5% ethanol / 95% CO_2 (100 bar)

Figure S223. SFC chromatogram of corresponding to **Figure S219**. Daicel ChiralPak AS-H (250 x 4.6 mm); 2.0 mL/min; 5% ethanol / 95% CO_2 (100 bar). Peak 2+ 4: **5-^{i}Bu**; Peak 1 + 3 – **6-^{i}Bu.** Unassigned signal corresponds to N-phenyl maleimide.

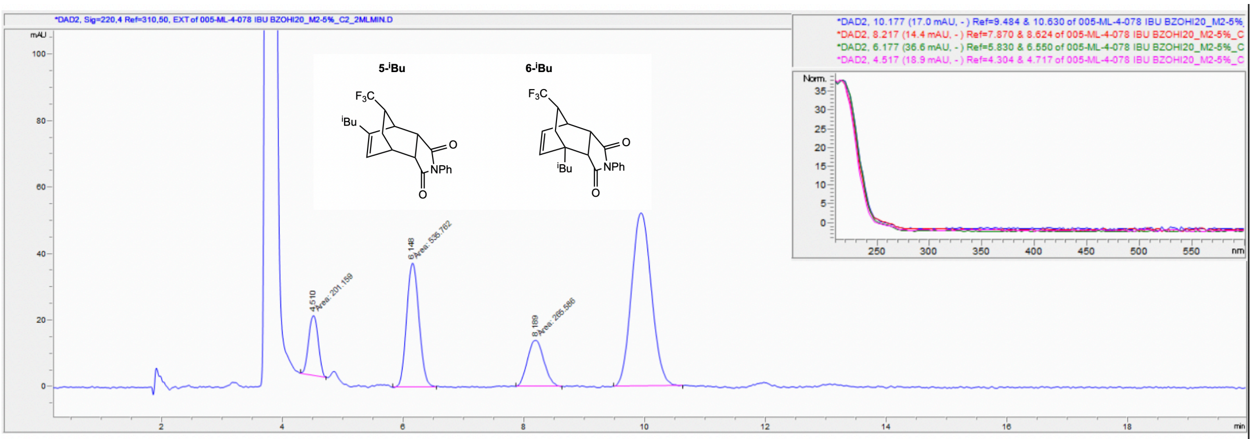

#	Time	Type	Area	Height	Width	Area%
1	4.51	MM	201.2	18.1	0.1855	9.144
2	6.148	MM	535.8	37.3	0.2391	24.353
3	8.189	MM	265.6	13.9	0.3184	12.072
4	9.932	BB	1197.5	52	0.3445	54.432

Instrument: Agilent 1260 SFC ("Aurora-2")
Column: Daicel ChiralPak AS-H (250 x 4.6 mm)
SFC Method: 2.0 mL/min; 5% ethanol / 95% CO_2 (100 bar)

Figure S224. SFC chromatogram of corresponding to **Figure S220**. Daicel ChiralPak AS-H (250 x 4.6 mm); 2.0 mL/min; 5% ethanol / 95% CO_2 (100 bar). Peak 2+ 4: **5-^{i}Bu**; Peak 1 + 3 – **6-^{i}Bu.** Unassigned signal corresponds to N-phenyl maleimide.

VIII. Crystallographic Data for Cobalt and Organic Complexes

Table S10. Crystal data and structure refinement for crystallographically characterized new compounds.[10,11]

	**Co1**	**Co4-N₂**	**Co4-H**	**Co1-a⁺**	**Co1-h⁺**	**1**
CCDC	2477307	2477308	2477309	2477310	2477311	2477312
Chemical formula	$C_{29}H_{48}CoP_2$	$C_{38}H_{66}Co_2N_2P_4$	$C_{48}H_{88}Co_3P_6 \cdot 1[C5H_{12}]$	$C_{33}H_{50}CoF_3P_2 \cdot C_6H_4F_2 \cdot F_6Sb$	$F_6Sb \cdot C_{34}H_{52}CoF_3P_2 \cdot 1[C4OH_8]$	$C_{11}H_9F_3O_3$
M_r	517.54	792.66	1027.79	974.44	946.48	246.18
Crystal system, space group	Orthorhombic, $P2_12_12_1$	Monoclinic, *C*2	Orthorhombic, $P2_12_12_1$	Orthorhombic, $P2_12_12_1$	Orthorhombic, $P2_12_12_1$	Orthorhombic, *Pbca*
Temperature (K)	105	100	100	110	100	100
a, *b*, *c* (Å)	11.0091 (6), 14.8778 (10), 17.3041 (9)	10.8617 (4), 17.5651 (6), 11.0308 (4)	12.8568 (2), 20.1002 (3), 22.4864 (3)	12.7858 (2), 15.8223 (2), 20.8314 (3)	11.9186 (5), 18.3790 (9), 18.8165 (6)	12.3758 (8), 10.2220 (7), 15.6537 (11)
V (Å^3)	2834.3 (3)	98.988 (2)	5811.03 (15)	4214.21 (10)	4121.8 (3)	1980.3 (2)
Z	4	2	4	4	4	8
Radiation type	Cu *K*a	Cu *K*a	Cu *K*a	Cu *K*a	Cu *K*α	Cu *K*α
m (mm^{-1})	5.90	7.90	8.39	9.56	9.70	1.37
Crystal size (mm)	0.21 × 0.11 × 0.04	0.24 × 0.22 × 0.16	0.18 × 0.12 × 0.06	0.17 × 0.16 × 0.12	0.21 × 0.13 × 0.10	0.10 × 0.07 × 0.05
Diffractometer	Bruker *APEX*-II CCD	Bruker *APEX*-II CCD	Bruker *APEX*-II CCD	Bruker *APEX*-II CCD	XtaLAB Synergy, Dualflex, HyPix-Arc 150	XtaLAB Synergy, Dualflex, HyPix-Arc 150

Absorption correction	Multi-scan *SADABS2016/2* (Bruker,2016/2) was used for absorption correction. wR2(int) was 0.1529 before and 0.0879 after correction. The Ratio of minimum to maximum transmission is 0.6500. The l/2 correction factor is Not present.	Multi-scan *SADABS2016/2* (Bruker,2016/2) was used for absorption correction. wR2(int) was 0.1348 before and 0.0868 after correction. The Ratio of minimum to maximum transmission is 0.6085. The l/2 correction factor is Not present.	Multi-scan *SADABS2016/2* (Bruker,2016/2) was used for absorption correction. wR2(int) was 0.1425 before and 0.1020 after correction. The Ratio of minimum to maximum transmission is 0.6877. The l/2 correction factor is Not present.	Multi-scan *SADABS2016/2* (Bruker,2016/2) was used for absorption correction. wR2(int) was 0.1440 before and 0.0925 after correction. The Ratio of minimum to maximum transmission is 0.6148. The l/2 correction factor is Not present.	Multi-scan *CrysAlis PRO* 1.171.43.143a (Rigaku Oxford Diffraction, 2024) Empirical absorption correction using spherical harmonics, implemented in SCALE3 ABSPACK scaling algorithm.	Multi-scan *CrysAlis PRO* 1.171.43.143a (Rigaku Oxford Diffraction, 2024) Empirical absorption correction using spherical harmonics, implemented in SCALE3 ABSPACK scaling algorithm.
T_{min}, T_{max}	0.489, 0.753	0.458, 0.753	0.518, 0.753	0.463, 0.753	0.689, 1.000	0.613, 1.000
No. of measured, independent and observed [$I > 2s(I)$] reflections	52286, 4989, 3803	18304, 3596, 3426	80754, 10215, 8733	41910, 7259, 6898	24301, 7772, 6225	10422, 1958, 1650
R_{int}	0.120	0.069	0.120	0.052	0.055	0.065
(sin q/l)$_{max}$ (Å^{-1})	0.596	0.597	0.596	0.596	0.629	0.629
$R[F^2 > 2s(F^2)]$, $wR(F^2)$, S	0.092, 0.263, 1.04	0.054, 0.157, 1.12	0.040, 0.095, 0.99	0.034, 0.080, 1.04	0.074, 0.206, 1.08	0.105, 0.255, 1.25
No. of reflections	4989	3596	10215	7259	7772	1958
No. of parameters	208	217	554	484	373	154
No. of restraints	13	1		30	18	
H-atom treatment	H-atom parameters constrained	H-atom parameters constrained	H atoms treated by a mixture of independent and constrained refinement	H-atom parameters constrained	H-atom parameters constrained	H-atom parameters constrained
Dρ$_{max}$, Dρ$_{min}$ (e Å^{-3})	0.89, -0.97	1.67, -0.44	0.43, -0.34	0.62, -0.73	1.15, -1.14	0.47, -0.33

Absolute structure	Refined as an inversion twin.	Refined as an inversion twin.	Flack x determined using 3241 quotients [(I+)-(I-)]/[(I+)+(I-)] (Parsons, Flack and Wagner, Acta Cryst. B69 (2013) 249-259).	Refined as an inversion twin.	Refined as an inversion twin.	
Absolute structure parameter	0.029 (17)	0.020 (8)	-0.014 (3)	0.009 (5)	0.038 (12)	

IX. References

1. Pangborn, A. B.; Giardello, M. A.; Grubbs, R. H.; Rosen, R. K.; Timmers, F. J. Safe and Convenient Procedure for Solvent Purification. *Organometallics* **1996**, *15*, 1518–1520.

2. Evans, D. F. 400. The Determination of the Paramagnetic Susceptibility of Substances in Solution by Nuclear Magnetic Resonance. *J. Chem. Soc.* **1959**, No. 0, 2003–2005.

3. Fischer, R.; Görls, H.; Meisinger, P. R.; Suxdorf, R.; Westerhausen, M. Structure–Solubility Relationship of 1,4-Dioxane Complexes of Di(Hydrocarbyl)Magnesium. *Chem. Eur. J.* **2019**, *25*, 12830–12841.

4. Kim, Y. B.; Kim, D.; Dighe, S. U.; Chang, S.; Park, J.-W. Cobalt-Hydride-Catalyzed Hydrosilylation of 3-Alkynes Accompanying π-Bond Migration. *ACS Catal.* **2021**, *11*, 1548–1553.

5. Mendelsohn, L. N.; Pavlovic, L.; Zhong, H.; Friedfeld, M. R.; Shevlin, M.; Hopmann, K. H.; Chirik, P. J. Mechanistic Investigations of the Asymmetric Hydrogenation of Enamides with Neutral Bis(Phosphine) Cobalt Precatalysts. *J. Am. Chem. Soc.* **2022**, *144*, 15764–15778.

6. Jonas, K. Reactive Organometallic Compounds from Metallocenes. *Pure Appl. Chem.* **1984**, *56*, 63–80.

7. Phan, D. H. T.; Kou, K. G. M.; Dong, V. M. Enantioselective Desymmetrization of Cyclopropenes by Hydroacylation. *J. Am. Chem. Soc.* **2010**, *132*, 16354–16355.

8. Seyferth, Dietmar.; Cheng, Y. Ming. Halomethyl-Metal Compounds. LXIII. Insertion of Phenyl(Bromodichloromethyl)Mercury-Derived Dichlorocarbene into Benzylic Carbon-Hydrogen Bonds. Stereochemistry and Mechanism. *J. Am. Chem. Soc.* **1973**, *95*, 6763–6770.

9. Wang, X.; Xu, Y.; Mo, F.; Ji, G.; Qiu, D.; Feng, J.; Ye, Y.; Zhang, S.; Zhang, Y.; Wang, J. Silver-Mediated Trifluoromethylation of Aryldiazonium Salts: Conversion of Amino Group into Trifluoromethyl Group. *J. Am. Chem. Soc.* **2013**, *135*, 10330–10333.

10. Dolomanov, O. V., Bourhis, L. J., Gildea, R. J., Howard, J. A. K. & Puschmann, H. (2009). *J. Appl. Cryst.* **42**, 339–341.

11. Sheldrick, G. M. (2015). *Acta Cryst.* C**71**, 3–8.

    a. Document origin: *publCIF* [Westrip, S. P. (2010). *J. Apply. Cryst.*, **43**, 920-925].
